# Proteomic characterization of post-mortem human brain tissue following ultracentrifugation-based subcellular fractionation

**DOI:** 10.1093/braincomms/fcac103

**Published:** 2022-04-21

**Authors:** Savannah E. Kandigian, Elizabeth C. Ethier, Robert R. Kitchen, Tukiet T. Lam, Steven E. Arnold, Becky C. Carlyle

**Affiliations:** 1 Harvard Medical School, Massachusetts General Hospital Department of Neurology, Charlestown, Boston, MA 02129, USA; 2 Harvard Medical School Department of Medicine, Charlestown, Boston, MA 02129, USA; 3 Yale University School of Medicine, Keck MS & Proteomics Resource, New Haven, CT 06511, USA; 4 Yale University School of Medicine, Department of Molecular Biophysics and Biochemistry, New Haven, CT 06511, USA; 5 Department of Physiology, Anatomy & Genetics, University of Oxford, New Biochemistry Building, South Parks Rd, Oxford OX1 3QU, UK

**Keywords:** spatial proteomics, mass spectrometry, Alzheimer’s disease, subcellular localization, neuroproteomics

## Abstract

Proteomic characterization of human brain tissue is increasingly utilized to identify potential novel biomarkers and drug targets for a variety of neurological diseases. In whole-tissue studies, results may be driven by changes in the proportion of the largest and most abundant organelles or tissue cell-type composition. Spatial proteomics approaches enhance our knowledge of disease mechanisms and changing signalling pathways at the subcellular level by taking into account the importance of cellular localization, which critically influences protein function. Density gradient-based ultracentrifugation methods allow for subcellular fractionation and have been utilized in cell lines, mouse and human brain tissue to quantify thousands of proteins in specific enriched organelles such as the pre- and post-synapse. Serial ultracentrifugation methods allow for the analysis of multiple cellular organelles from the same biological sample, and to our knowledge have not been previously applied to frozen post-mortem human brain tissue. The use of frozen human tissue for tissue fractionation faces two major challenges, the post-mortem interval, during which proteins may leach from their usual location into the cytosol, and freezing, which results in membrane breakdown. Despite these challenges, in this proof-of-concept study, we show that the majority of proteins segregate reproducibly into crude density-based centrifugation fractions, that the fractions are enriched for the appropriate organellar markers and that significant differences in protein localization can be observed between tissue from individuals with Alzheimer’s disease and control individuals.

## Teaser

Spatial proteomics accomplished by serial ultracentrifugation of frozen post-mortem brain tissue is capable of assigning thousands of brain proteins to large membranous cellular compartments, including the nucleus, mitochondria and synapse, and to the non-membranous cytosolic compartment.

## Graphical Abstract

**Graphical Abstract:**
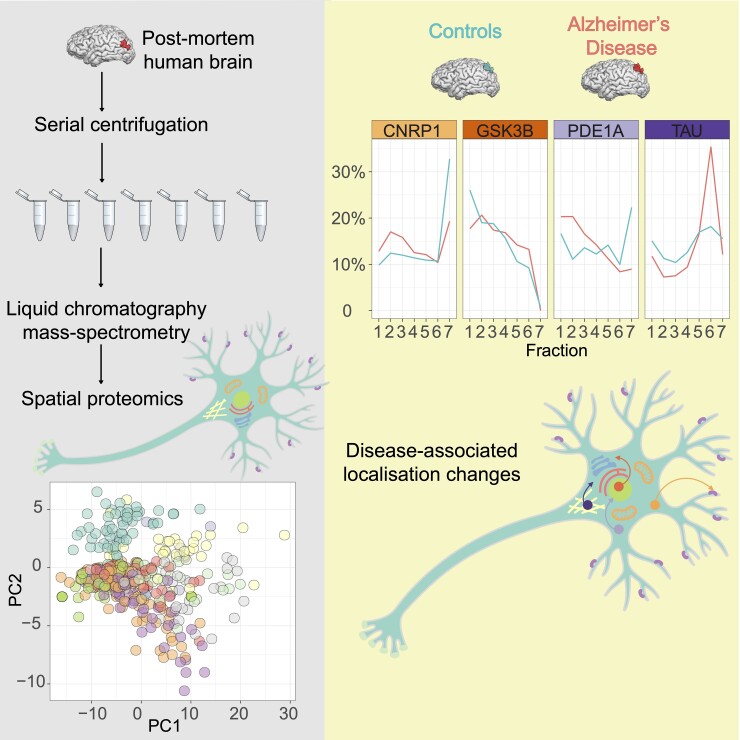

## Introduction

Proteomic characterization of brain tissue is increasingly utilized to investigate mechanisms and identify potential novel biomarker and drug targets for a variety of neurological diseases. Analysis of human brain tissue using liquid chromatography–mass spectrometry (LC-MS/MS) has revealed disease-related proteome differences beyond alterations found at the mRNA level in neurodegenerative diseases and neuropsychiatric disorders.^1–4^ Spatial proteomics approaches (reviewed in Lundberg and Borner^5^) may further enhance our knowledge of disease mechanisms and changing signalling pathways at the subcellular level by taking into account the importance of cellular localization for protein function. Through fractionation of a sample into its organellar compartments prior to LC-MS/MS analysis, patterns of protein mis-localization in disease states may be identified. Furthermore, spatial fractionation prior to analysis by LC-MS/MS simplifies the input sample, and may enable more sensitive detection of low abundance proteins that might remain undetected in whole-tissue LC-MS/MS.

Proteins can be active in multiple cellular localizations and the subcellular context critically influences protein function.^6^ Subcellular localization of a given protein dictates pH of the reaction environment, the availability of molecular interaction partners and the post-translational modification process. Dysregulation of protein localization can lead to the loss of protein function or gain of toxic functions, and such changes have been linked to a number of human diseases.^7,8^ Many neurodegenerative diseases are characterized by protein misfolding and accumulation in certain subcellular locations. Neumann *et al*.^7^ showed that in patients with Amyotrophic Lateral Sclerosis (ALS), diseased neurons show redistribution of TAR DNA-binding protein 43 (TDP-43) from the nucleus to the cytoplasm. In Alzheimer’s disease, numerous interacting pathways with varying subcellular localizations become dysregulated, and subcellular proteomics may enhance the specificity of our characterization of these pathways. For example, Shen *et al*.^8^ recently showed that enrichment of mitochondrial, myelin sheath and synaptosomal fractions from transgenic Alzheimer’s disease mouse model tissue was able to identify compartment-specific alterations in disease-relevant pathways such as metabolism and synaptic dysfunction. Carlyle *et al.*^4^ furthermore showed that enrichment of synaptosomes from post-mortem human brain tissue revealed a number of targets selectively associated with cognitive impairment in older individuals that were not revealed by whole-tissue studies. These studies indicate the potential value of broader subcellular profiling of the Alzheimer’s disease brain.

In addition to the mechanistic insights enabled by spatial proteomics, these methods avoid volume confounds inherent in whole-tissue proteomics. In whole-tissue studies, results may be driven by the largest and most abundant organelles, and changes identified between brain regions may in fact reflect healthy differences in organellar composition,^9^ while disease-related changes may refer to a difference in tissue cell-type composition. For example, the increased immune protein signal seen in a number of whole-tissue studies^1–3^ may reflect an increased volume of cortical tissue occupied by activated microglia or encroaching gliosis, rather than highlight the more subtle dysregulation of pathways and protein–protein networks. Spatial proteomics allows for the characterization of disease-associated changes in the proteomic makeup of an organelle type while controlling for the confound of changes in the total abundance of those organelles. Alzheimer’s disease, for example, is characterized by widespread synaptic loss.^10–12^ Whole-tissue proteomics may therefore show loss of synaptic proteins, but this does not lend insight into the makeup of remaining synapses and how synaptic dysfunction and degeneration progress.

Serial density gradient-based ultracentrifugation methods allow for subcellular fractionation and have been utilized in cell lines and primary cortical neurons to predict the subcellular location of thousands of proteins.^13,14^ Spatial proteomic analysis of brains from 10-month-old mice with a disease-relevant mutation in the Wash complex subunit 4 (SWIP) gene showed disruption of the Wiskott–Aldrich syndrome protein and scar homologue complex (WASH) complex resulting in altered abundance of hundreds of proteins across multiple subcellular fractions.^15^ To our knowledge, these serial fractionation techniques have not been used in frozen human tissue. The use of frozen human tissue for tissue fractionation faces three major challenges, the post-mortem interval (PMI), during which proteins may leach from their usual location into the cytosol, the complexity of the tissue which harbours hundreds of cell types, and freezing, which results in membrane breakdown. Despite these challenges, in this pilot study, we show that the majority of proteins segregate reproducibly into crude density-based centrifugation fractions, that the fractions are enriched for the appropriate organellar markers and that significant differences in protein localization can be detected between tissue from individuals with Alzheimer’s disease compared with control individuals.

## Materials and methods

### Samples

Frozen post-mortem human brain tissue was obtained from the Massachusetts Alzheimer’s Disease Research Center (MADRC). Paraffin-embedded post-mortem brain tissue slices were obtained from the Penn Memory Center. Experiments were conducted in accordance with the Partners Healthcare Institutional Review Board. Two hundred milligram sections of angular gyrus tissue were sectioned on dry ice from five subjects diagnosed with Alzheimer’s disease and five age, sex and PMI-matched controls. Demographic data for the sample are shown in Table 1.

**Table 1:** Demographics of the AD versus control sample set

	AD	Control	Overall
	(*N* = 5)	(*N* = 5)	(*N* = 10)
Age			
Mean (SD)	89.8 (2.77)	90.2 (3.42)	90.0 (2.94)
Median (Min, Max)	91.0 (86.0, 93.0)	91.0 (85.0, 94.0)	91.0 (85.0, 94.0)
PMI			
Mean (SD)	15.0 (4.8)	15.0 (5.96)	15.0 (5.10)
Median (Min, Max)	12.0 (12.0, 23.0)	13.0 (8.00, 23.0)	12.5 (8.00, 23.0)

PMI, post-mortem interval in hours.

### Fractionation

**Figure 1:**
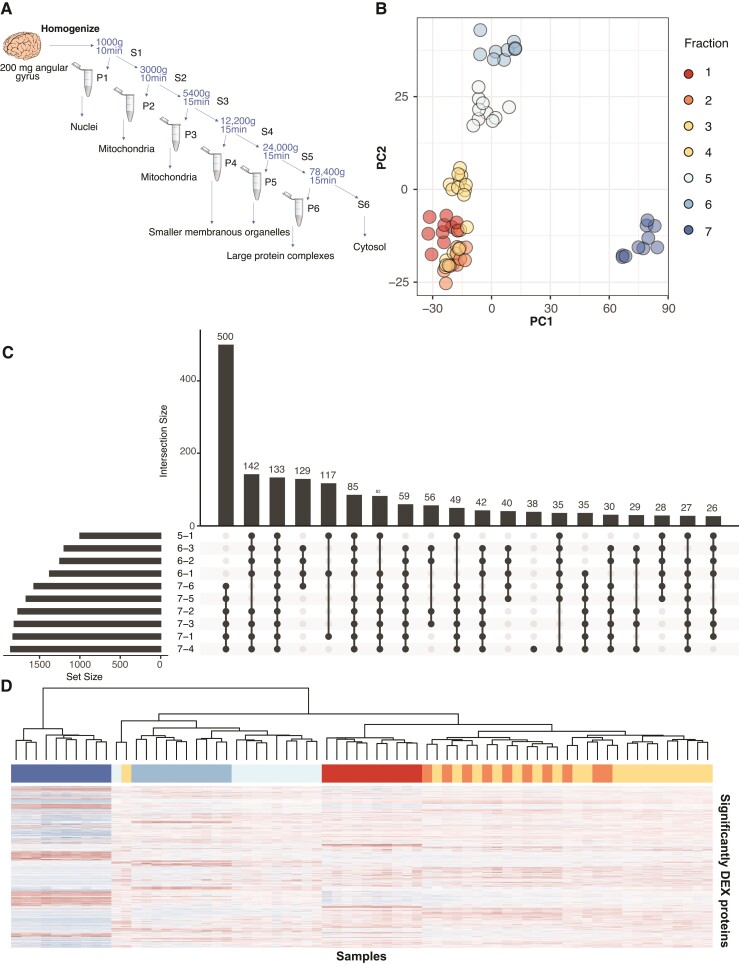
**Differential centrifugation can be used to separate proteins into consistent fractions in post-mortem human brain**. (**A**) Schematic of the centrifugation scheme used to prepare samples for this experiment. Centrifuge speeds and spin times are provided. All spins were performed at 4°C. (**B**) PCA shows good separation of samples by centrifugation fraction in the first two principal components. (**C**) An Upset plot shows that most proteins in the data set differentially expressed by ANOVA are differentially abundant between Fraction 7 (cytosolic fraction) and Fraction 6 (large protein complex fraction) and all other fractions. (**D**) A heatmap of differentially expressed proteins (ANOVA, see Supplementary Table 3 for test statistics) shows that samples generally cluster on the basis of centrifugation fraction. The exception is Fractions 2 and 3, where samples from the same individual cluster in pairs within the larger cluster. Colour coding of samples in the horizontal bar is identical to colour coding in **B**.

Methods were adapted from Itzhak *et al*.^13^ Samples were homogenized with a dounce homogenizer using 15 strokes at 800 rpm in lysis buffer (25 mM Tris–HCl, 50 mM sucrose, 0.5 mM MgCl_2_, 0.2 mM EGTA) with protease inhibitors. Following homogenization, sucrose concentration was readjusted to 250 mM. All centrifugation steps were carried out at 4^o^C in a benchtop microcentrifuge (<24 000×g) or a Beckman Coulter Ultracentrifuge (>24 000×g) using an MLA-50 rotor (Beckman Coulter) and 10 ml polypropylene tubes. Pellets and supernatants were handled on the ice at all times between spins. A schematic of the differential centrifugation protocol, including times and speeds, is shown in Fig. 1A. Following each centrifugation step, the supernatant was transferred to a new tube and pellets were frozen at −80°C. The final supernatant, representing the cytosolic fraction, underwent acetone precipitation as follows: 400 μl of supernatant was mixed with 1.6 ml of pre-cooled acetone and incubated for 1 h at −20°C. The resulting pellet was resuspended in solubilization buffer (8 M urea, 0.4 M ammonium bicarbonate) and frozen at −80°C.

### Sample preparation for mass spectrometry

Pellets were resuspended in solubilization buffer (8 M urea, 0.4 M ammonium bicarbonate) with protease inhibitors and briefly sonicated. Samples were cleared by centrifugation, total protein content was assessed by BCA and each sample was adjusted to 100 μg/50 μl of solubilization buffer. For samples with protein contents below this threshold, 50 μl of the straight sample was used. Supplementary Table 1 and Fig. 1 show protein yields for each individual sample fraction. Following reduction with dithiothreitol (45 mM at 1/10th sample volume) for 30 min at room temperature and alkylation with iodoacetamide (100 mM at 1/10th sample volume) for 30 min in the dark at room temperature, samples were trypsin digested overnight with a 1:20 protein:enzyme ratio. Samples were acidified to stop digestion, desalted on C18 Microspin columns (Nest Group) and dried in a SpeedVac. The resulting pellets were frozen at −80°C until transport. Eluted peptides were speed-vacced dried and dissolved in MS loading buffer (2% acetonitrile, 0.2% trifluoroacetic acid). A nanodrop (Thermo Scientific Nanodrop 2000 UV–Vis Spectrophotometer) was used to determine protein concentrations (A260/A280). Each sample was then further diluted with MS loading buffer to 0.08 µg/µl, with 0.4 µg (5 µl) injected for LC-MS/MS analysis.

### Liquid chromatography tandem mass spectrometry

LC-MS/MS analysis was performed on a Thermo Scientific Orbitrap Fusion equipped with a Waters nanoAcquity UPLC system utilizing a binary solvent system (Buffer A: 100% water, 0.1% formic acid; Buffer B: 100% acetonitrile, 0.1% formic acid). Trapping was performed at 5 µl/min, 97% Buffer A for 3 min using a Waters Symmetry^®^ C18 180 µm ×20 mm trap column. Peptides were separated using an ACQUITY UPLC PST (BEH) C18 nanoACQUITY Column 1.7 µm, 75 µm ×250 mm (37°C) and eluted at 300 nl/min with the following gradient: 3% Buffer B at initial conditions; 6% B at 5 min; 35% B at 170 min; 50% B at 175 min; 97% B at 180 min; 97% B at 185 min; return to initial conditions at 186–200 min. MS was acquired in the Orbitrap in the profile mode over the 350–1550 *m*/*z* range using wide quadrupole isolation, 1 microscan, 120 000 resolution, AGC target of 4E5 and a maximum injection time of 60 ms. Data-dependent MS/MS were collected in the top speed mode with a 3 s cycle time on species with an intensity threshold of 5E4, charge states 2–8, peptide monoisotopic precursor selection preferred. Dynamic exclusion was set to 30 s. MS/MS were acquired in the Orbitrap in the centroid mode using quadrupole isolation (window 1.6 *m*/*z*), HCD activation with a collision energy of 28%, 1 microscan, 60 000 resolution, AGC target of 1E5 and maximum injection time of 110 ms.

### Data analysis

Raw mass spectrometry data were processed in MaxQuant using default settings and the addition of the ‘match between runs’ feature. All downstream analyses were performed in R (v4.0.2), and the tidyverse (v1.3.0), UpSetR (v1.4.0), Caret (v6.0–86), Openxlsx (v.4.2.3) and TopGo (v2.40.0 with AnnotationDbi v1.50.3) packages. Filtering of this data set was minimal, as the fractions were expected to have quite different sets of proteins residing in them. Proteins with over 60% missing values were excluded from further analysis, with missing values set as NAs (Supplementary Fig. 2), zero [support vector machine (SVM) and entropy/within-fraction *t*-tests] or set as the minimum label-free quantification (LFQ) detected for that protein [principal component analysis (PCA), Heatmaps, analysis of variance (ANOVA) and Tukey’s honest significant difference test]. No further normalization was applied post-MaxQuant processing. PCA was performed using the prcomp function in base R. All plotting was performed using ggplot2 functions, except heatmaps which were produced with the heatmap.2 function and the UpSet plot (Fig. 1C) which was produced using UpSetR. Unless stated otherwise, all references to significance refer to *P*-values adjusted using the Benjamini–Hochberg method.^16^

SVM supervised learning was used to classify proteins to organelles and was performed on each biological sample individually. The training was performed on centred and scaled LFQ values with a radial basis function kernel and, to ensure balanced classes, marker proteins were randomly down-sampled to 35 proteins in each marker set. Ten-fold cross-validation was used to minimize over-fitting and we used 10 tuning levels for sigma (a.k.a. gamma) and the cost function, *C*. The performance of the training set was simplified to a single summary percentage of correctly assigned proteins per organelle set. The classification was performed on a sample-by-sample basis on all proteins that were not included in the training set for that sample.

Due to the limitations inherent in the organellar marker lists for brain tissue, the small number of markers observed in some categories that prevented them from being included in the SVM, and the overlapping patterns of segregation of some organelles, we chose a method of identifying differences between a diagnostic condition that was agnostic to the SVM classifications. Global entropy values were calculated on the proportions of total LFQ signal present in each fraction for each protein and subject. We used Shannon’s definition of entropy [calculated as: entropy = −sum(ratio × log2(ratio))/log2(*n*)] to measure the degree of disorder of each protein in each subject relative to the subcellular fractions. Low entropy values denote proteins highly ordered—i.e. those specific to a small number of fractions. For the purpose of these calculations, NA values were set as 0.0000000001. Global delta entropy was calculated as the difference between the mean entropy values for control subjects and the mean entropy values for the Alzheimer’s disease subjects.

### Immunohistochemistry

Tissue sections from the angular gyrus of individuals with Alzheimer’s disease and age-matched controls (*n* = 5/group, mean age Alzheimer’s disease = 77.6 ± 10.4 years, mean age Control = 69 ± 19.8 years, per cent female Alzheimer’s disease = 40%, per cent female Control = 60%) were labelled with anti-GSK3β and DAPI (4′,6-diamidino-2-phenylindole) nuclear stain. Mounted slices were dewaxed in an oven at 60°C for 30 min, followed by deparaffinization in 100% xylene. Sections were rehydrated in decreasing concentrations of ethanol in distilled water (100, 100, 95, 70, 50, 0%), then boiled in citrate buffer (10 mM citric acid, 0.05% Tween 20, pH 6) for 20 min to perform antigen retrieval. Sections were blocked and permeabilized in Tris-buffered saline (TBS) with 0.25% Triton X-100 and 5% bovine serum albumin for 1.5 h at room temperature, before incubation with anti-GSK3β (Thermo Fisher MA5-15597) diluted 1 in 200 in blocking solution overnight at 4°C. Following three TBS washes, sections were incubated with Alexa Fluor 568 secondary antibody (Thermo Fisher Donkey anti-mouse, A10037) diluted 1 in 200 in TBS with 0.25% Triton X-100 for 1.5 h at room temperature, then washed a further three times with TBS. To remove lipofuscin autofluorescence, sections were counter-stained with Trublack (Biotium) according to the manufacturer’s instructions. Following Trublack staining, sections were rinsed in TBS then coverslipped with Fluoromount G containing DAPI (Invitrogen). Two images were acquired from each stained section using an Olympus Fluoview confocal microscope with a 60× oil immersion objective. The investigator taking the images was blinded to the disease condition. Single-channel images were imported into ImageJ and analysed using the JACoP plugin^17^ by an investigator blinded to the subject disease condition. Manders M2 coefficients, quantifying the proportion of GSK3β signal overlapping with nuclear DAPI staining, were extracted and plotted for each individual image. The mean M2 coefficients were calculated for each subject, and a Student’s *t*-test was used to assess differences in the signal overlap between Alzheimer’s disease and control subjects.

### Data and code availability

The mass spectrometry proteomics data have been deposited to the ProteomeXchange Consortium via the PRIDE partner repository with the data set identifier PXD027456. Code for the analysis of this project is available at: https://github.com/ACTRU/becky-carlyle-fractionation-MS.

## Results

### Quality control

Post-mortem frozen cortical tissue from 10 individuals was subcellularly fractionated by progressive ultracentrifugation into six membranous organellar fractions and a final cytosolic supernatant fraction (Fig. 1A). Student’s *t*-test showed no significant differences in protein yield between the control and Alzheimer’s disease samples in any fraction (Supplementary Table 1, plotted in Supplementary Fig. 1). All 70 of the resulting samples were analysed by single-shot label-free LC-MS/MS. A total of 3843 proteins with at least two unique peptides were identified in at least one sample, and 850 proteins were identified in every sample (Supplementary Table 2). The distribution of LFQ values across samples were even and required no further normalization (Supplementary Fig. 2). Because of the nature of the progressive removal of centrifuged pellets, it was expected that not all proteins would be identified in all fractions, particularly those proteins which localize to certain organelles. Therefore, in order to conduct statistically rigorous analyses without disregarding proteins that are appropriately undetected in certain fractions, we did not remove proteins which were not detected across all samples from our analysis. Only samples with >60 missing values were filtered from the data set, allowing a protein to be detected in all replicates from only one fraction. Once this filter was applied, 3061 proteins remained for analysis.

### Sample clustering

A PCA was conducted to examine how samples clustered according to protein abundance (Fig. 1B). The PCA shows good stratification of samples by fraction, with clean clusters for Fractions 4–7, while Fractions 1–3 cluster more closely together. PC1 (accounting for 33.2% of the variance) mainly separated the cytosolic fraction (F7) from the organelle pellets (F1–6). PC2 (accounting for 20.6% of the variation) captures most of the separation between the remaining organelle fractions. This initial analysis showed that samples were grouped together based on centrifugation fraction rather than subject.

To further analyse protein distribution across the samples, a heatmap was generated using all proteins identified (Supplementary Fig. 3). As expected, similar to the PCA, the strongest effect was the separation of the cytosolic fraction (F7) from the organelle pellets (F1–6). Other fractions, most notably Fractions 1, 5 and 6, also showed good separation. In some instances, samples are clustered primarily by individual subject as opposed to fractions. This can be seen in Supplementary Fig. 3, where Fractions 2 and 3, and 5 and 6, are interspersed by subject. To improve clustering, we performed an ANOVA to identify proteins with significantly altered abundance between fractions. A total of 2701 proteins were significantly differentially expressed (adjusted *P* < 0.05; Supplementary Table 3). *Post hoc* testing (Tukey’s MSD) revealed that the highest number of significant pairwise comparisons arose from the comparisons between Fraction 7 and the other six fractions, again confirming that the cytosol is an outlier fraction (Fig. 1C). Clustering using only these significant proteins substantially improved the separation of the samples by a fraction (Fig. 1D), although the pairing of subjects remained in Fractions 2 and 3. The successful stratification of the samples based on these significant proteins indicates that the fractions had qualitatively different protein distributions.

### Marker protein distribution

**Figure 2:**
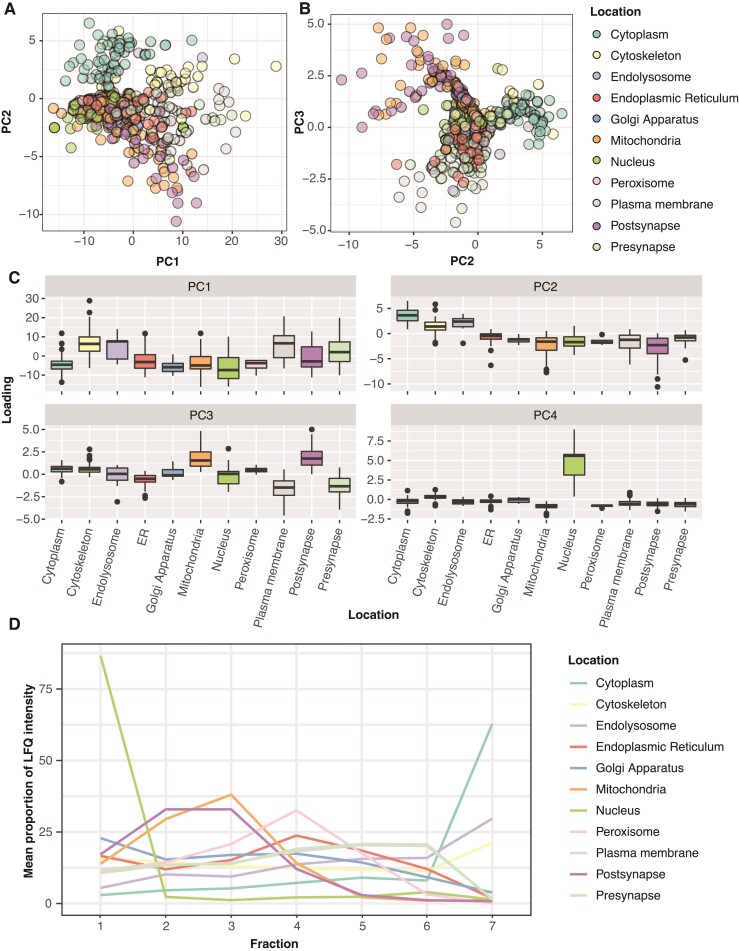
**Established markers proteins segregate according to reproducible patterns across fractions.** (**A**) Marker proteins plotted according to their locations along PC1 and PC2. Supplementary Figure 4 shows a faceted plot of each set of organellar markers alone, to inspect proteins in the crowded central region of the plot. The combination of PCs 1 and 2 shows good separation for cytoskeletal and cytosolic proteins in the lower part of PC2, and mitochondrial and post-synaptic proteins in the top half. (**B**) PC plot of PCs 2 and 3. PC3 nicely separates post-synaptic and mitochondrial proteins from pre-synaptic and plasma membrane proteins. (**C**) Box plots show the association of marker proteins from each organelle with the top 4 principal components. PC4 separates the nuclear markers from all other organelles. (**D**) Line plot of the average proportional distribution profile of the marker set for each organelle.

To assess whether our fractionation scheme successfully sorted organelles into the predicted fractions, we examined the behaviour of marker proteins known to localize to a singular subcellular organelle. Given the lack of neuron-specific organellar protein information available, we intersected the organelle lists used in Hela cells in Itzhak *et al*.^13^ and added pre- and post-synaptic terms from an inclusion list generated in Carlyle *et al*.^4^ (Supplementary Table 4). We generated an organelle marker map by performing a PCA on all proteins across all samples. Figure 2A represents the distribution of marker proteins along PC1 and PC2, with Fig. 2B showing PC2 and PC3. Due to the overlapping of points in both plots, Supplementary Fig. 4 shows each marker class independently on individual plots. In agreement with the sample PCA and protein clustering, proteins from the cytosolic fraction are most clearly separated by the first two principal components, with PC2 separating markers of the large membranous organelles from cytosolic and cytoskeletal markers (Fig. 2C). PC3 separates mitochondria and post-synaptic markers from plasma membrane and pre-synaptic markers, while PC4 clearly separates nuclear proteins from all other markers (Fig. 2C). However, the smaller membranous organelles, as expected, show substantially more overlap, indicating that the ultracentrifugation-based approach is less able to cleanly enrich these organelles. Assessment of these markers was also limited by a low number of markers from these organelles being detected in this single-shot proteomic experiment (Supplementary Fig. 4). Finally, we plotted the mean LFQ distribution between fractions of these marker proteins and showed patterns similar to those seen in Hela cells^13^ (Fig. 2D). Synaptic proteins, which are not present in Hela cells, showed patterns of segregation almost identical to two other organelles, with post-synaptic proteins co-segregating with mitochondria, and pre-synaptic proteins segregating with Hela cell plasma membrane markers.

### Localization prediction modelling

The organelle markers were used to predict the localization of unannotated proteins using an SVM model (R Caret package).^18^ The SVM was trained on a down-sampled set of marker proteins (*n* = 35 proteins per organelle), then used to classify all non-marker proteins to a likely cellular compartment. The SVM process was repeated 10 times, once for each experimental subject. The performance of the SVM model on the training set was high for the cytoplasm and nuclear fractions (mean percentage of proteins assigned to the appropriate organelle >75%), intermediate for the ER, mitochondria and cytoskeleton (> 50% appropriate assignment) and low for the pre- and post-synaptic fractions. Differences in SVM performance with reference to major covariates were quantified by a linear model; there was no difference in performance between control and Alzheimer’s disease samples (Supplementary Fig. 5A), and performance was not affected by PMI (Supplementary Fig. 5B). There were insufficient markers available for the Golgi apparatus and smaller membranous organelles (endolysosomes and peroxisomes) in this data set, and therefore, no categories for these organelles were generated by the model. Proteins were classified as high confidence (*n* = 1006) if the SVM placed them in the same organelles in at least four out of five control samples, medium confidence (*n* = 1257) if there was a dominant location assignment across the five samples and low confidence (*n* = 615) if organelle assignment was different in all five samples (Supplementary Table 5). The 1006 high-confidence proteins were then analysed for cellular compartment enrichment using the R package TopGo (Table 2).^19^

**Table 2:** The top five enriched GO terms for each set of SVM assigned organelles

GO ID	Term	Annotated	Significant	Expected	Classic Fisher’s *P*-value	SVM fraction
GO:0005829	Cytosol	1490	138	103.45	4.30E−07	Cytoplasm
GO:0072562	Blood microparticle	56	11	3.89	0.0013	Cytoplasm
GO:0120115	Lsm2–8 complex	5	3	0.35	0.003	Cytoplasm
GO:1902560	GMP reductase complex	2	2	0.14	0.0048	Cytoplasm
GO:0008537	Proteasome activator complex	2	2	0.14	0.0048	Cytoplasm
GO:0005622	Intracellular	2682	190	181.58	0.0035	Cytoskeleton
GO:0005829	Cytosol	1490	130	100.88	1.90E−06	Cytoskeleton
GO:0070062	Extracellular exosome	950	82	64.32	0.0038	Cytoskeleton
GO:1904813	Ficolin-1-rich granule lumen	84	14	5.69	0.0012	Cytoskeleton
GO:0031093	Platelet alpha granule lumen	22	6	1.49	0.0027	Cytoskeleton
GO:0016021	Integral component of membrane	586	114	70.04	1.20E−9	ER
GO:0005783	Endoplasmic reticulum	333	73	39.8	4.80E−7	ER
GO:0005789	Endoplasmic reticulum membrane	228	69	27.25	5.80E−14	ER
GO:0000139	Golgi membrane	156	38	18.64	0.00011	ER
GO:0030126	COPI vesicle coat	10	9	1.2	4.10E−8	ER
GO:0016021	Integral component of membrane	586	19	11.94	0.0044	Mitochondria
GO:0005739	Mitochondrion	564	47	11.49	0.0022	Mitochondria
GO:0005743	Mitochondrial inner membrane	196	22	3.99	4.70E−7	Mitochondria
GO:0005759	Mitochondrial matrix	185	21	3.77	1.70E−7	Mitochondria
GO:0005947	Mitochondrial alpha-ketoglutarate dehydr…	5	2	0.1	0.0039	Mitochondria
GO:0005654	Nucleoplasm	603	38	12.29	1.50E−12	Nucleus
GO:0000786	Nucleosome	43	15	0.88	4.50E−6	Nucleus
GO:0000788	Nuclear nucleosome	27	10	0.55	3.60E−11	Nucleus
GO:0005604	Basement membrane	23	11	0.47	5.00E−7	Nucleus
GO:0043260	Laminin-11 complex	3	3	0.06	8.00E−6	Nucleus
GO:0016020	Membrane	1868	72	55.49	4.70E−10	Plasma membrane
GO:0022627	Cytosolic small ribosomal subunit	37	8	1.1	8.40E−6	Plasma membrane
GO:0000784	Nuclear chromosome, telomeric region	33	14	0.98	7.30E−14	Plasma membrane
GO:0000788	Nuclear nucleosome	27	14	0.8	2.10E−15	Plasma membrane
GO:0042788	Polysomal ribosome	23	7	0.68	2.70E−6	Plasma membrane
GO:0005743	Mitochondrial inner membrane	196	15	2.03	5.90E−5	Post-synapse
GO:0005759	Mitochondrial matrix	185	8	1.92	0.0064	Post-synapse
GO:0005747	Mitochondrial respiratory chain complex …	39	5	0.4	3.80E−5	Post-synapse
GO:0031305	Integral component of mitochondrial inne…	17	3	0.18	0.0042	Post-synapse
GO:0098831	Pre-synaptic active zone cytoplasmic comp…	11	2	0.11	0.0054	Post-synapse
GO:0005887	Integral component of plasma membrane	185	9	1.79	0.0014	Pre-synapse
GO:0043025	Neuronal cell body	161	6	1.56	0.0051	Pre-synapse
GO:0035579	Specific granule membrane	31	3	0.3	0.003	Pre-synapse
GO:0017101	Aminoacyl-tRNA synthetase multienzyme co…	12	2	0.12	0.0056	Pre-synapse
GO:0070110	Ciliary neurotrophic factor receptor com…	1	1	0.01	0.0097	Pre-synapse

All terms are significantly enriched (classicFisher <0.05). The table shows proteins annotated (‘Annotated’) in that GO term, compared with proteins present in that organellar set (‘Significant’). For enrichment to be significant, the number of proteins in set will be greater than those expected by chance in a data set of this size (‘Expected’).

Fisher’s tests showed strong, appropriate enrichment for the nuclear (‘Nuclear nucleosome’, ‘Nucleoplasm’), Mitochondria (‘Mitochondrial matrix’, ‘Mitochondrial inner membrane’) and cytosol (‘cytosol’) assigned proteins. The endoplasmic reticulum (ER) protein set was strongly enriched for both ER (‘endoplasmic reticulum membrane’, ‘Endoplasmic reticulum’) and Golgi terms (‘COPI vesicle coat’, ‘Golgi membrane’). This overlap may be in part due to the lack of a dedicated Golgi compartment in the model. The cytoskeleton was the poorest performing category, showing enrichment for many proteins that failed to show a clear distribution pattern across fractions. Finally, unsurprisingly, the model also performed worse at identifying dedicated post- and pre-synaptic categories, given their distribution closely follows that of the mitochondria and plasma membrane, respectively (Fig. 2D). Despite this difficulty in formally assigning a compartment to synaptic fractions, the segregation pattern tends to be robust even for proteins with low confidence in SVM organelle assignment, with the distribution of root mean squares error centring on 0.05–0.06 for high-confidence proteins and 0.05–0.07 for low-confidence proteins (Supplementary Fig. 5C). This suggests that for an average high-confidence protein, the ratio across the fractions varies by ∼5–6% compared with the mean fractionation pattern, and by 5–7% in low-confidence proteins, which will still enable a change in the fractionation pattern due to disease condition to be detected.

### Disease-associated changes in protein localization

As a proof of concept, to define whether different protein segregation patterns were detectable between control (Braak Stages 1–3) and Alzheimer’s disease (Braak 4–6) tissue (Table 1), we ranked proteins by their difference in global delta entropy (see the ‘Materials and methods’ section) between Alzheimer’s disease and control and performed multiple corrected within-fraction *t*-tests to identify the fraction where differences in protein distribution were significant.

**Figure 3:**
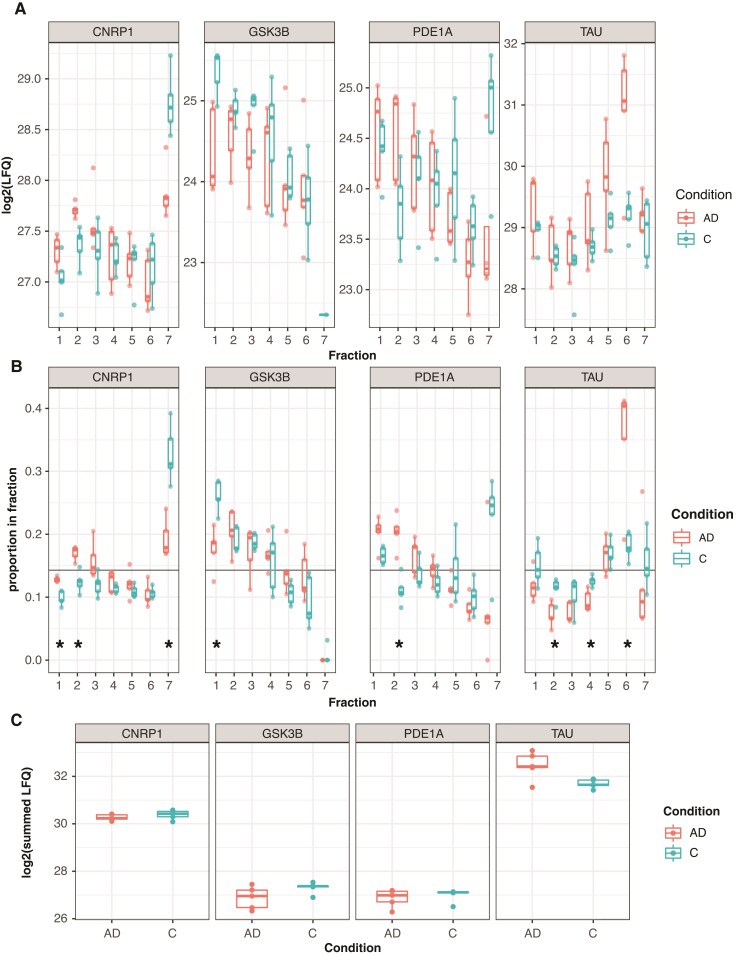
**Changes in protein distribution pattern between control and AD individuals can be detected in this data set**. (**A**) Boxplots of log2LFQ values for the four proteins of interest; raw LFQ values can be variable between samples in the same diagnostic group. Each point represents an individual sample, *n* = 5 per diagnostic condition. (**B**) Boxplots of proportional ratios of protein in each of the seven fractions; variation between samples is much lower when fractionation patterns are expressed as a proportion of total LFQ. Significant differences between AD and controls (within-fraction Student’s *t*-test, Supplementary Table 6, Benjamini–Hochberg-adjusted *P* < 0.05) in single fractions are noted with an asterisk. Individual samples are plotted as individual points. The horizontal black line shows the segregation pattern of a protein of zero entropy. (**C**) Summed LFQs across all fractions show a low likelihood of differential expression from these proteins across the whole tissue.

**Figure 4:**
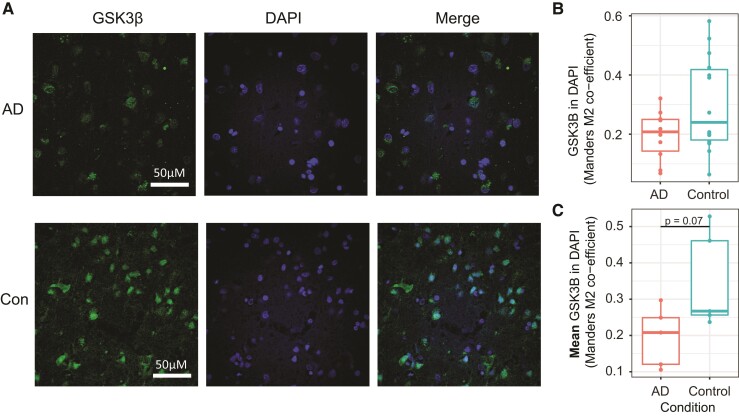
**Immunohistochemistry of angular gyrus sections with anti-GSK3β shows a trend towards increased nuclear GSK3β in controls**. (**A**) Representative images show increased colocalization of GSK3β signal with nuclear DAPI staining. Subjective visual analysis suggests increased presence of nuclear speckles in Control samples compared with AD. (**B**) Plot showing Mander’s M2 overlap coefficient for each individual image shows an enrichment for increased nuclear overlap of GSK3β staining with DAPI. (**C**) The mean Mander’s M2 coefficient for each subject shows a trend (Student’s *t*-test, *T* = −2.18, *P* = 0.07) towards increased nuclear overlap of GSK3β in controls compared with AD.

Eighty-five proteins had significant differences between Alzheimer’s disease and control in at least one fraction (Supplementary Table 6). From these lists, we identified several interesting candidates exhibiting likely altered subcellular localization (Fig. 3A and B). Cannabinoid Receptor Interacting Protein 1 (CNRIP) is high in the cytosol and low in the nucleus in control tissue, whereas in Alzheimer’s disease tissue, it is more strongly associated with the membrane Fractions 2 and 3. Glycogen Synthase Kinase 3-beta (GSK3β) is a kinase linked to tau phosphorylation and associated with psychiatric disease susceptibility that functions as a major point of integration for critical neuronal signalling pathways. It also has a function in the nucleus, where it acts to regulate the transcription of a number of genes, including those in the Wnt signalling pathway.^20,21^ In control samples, GSK3β was most strongly associated with the nuclear fraction, and in Alzheimer’s disease samples, this nuclear association was much smaller. To follow up the GSK3β finding, sections from the angular gyrus of five individuals with Alzheimer’s disease and five age-matched controls were labelled with DAPI nuclear stain and an antibody against GSK3β, and imaged by confocal microscopy (Fig. 4A). Two images from each subject were analysed for signal overlap between GSK3β and DAPI by calculating the Manders M2 coefficient^22^ (Fig. 4B). The mean Manders M2 coefficient was calculated for each subject, and Student’s *t*-test showed a trend (*T* = −2.18, *P* = 0.07) towards increased overlap of GSK3β signal with nuclear staining in Control versus Alzheimer’s disease (Fig. 4C), providing likely validation of the mass spectrometry results. Subjectively, GSK3β showed a nuclear speckle^23^ pattern in Control samples more frequently than in Alzheimer’s disease samples (Fig. 4A).

Phosphodiesterase 1A (PDE1A) localizes to the nucleus and large membrane fractions in both conditions, but there is a large cytosolic pool in controls absent in Alzheimer’s disease. In this example, we may not be seeing mis-localization of PDE1A, but a change in predominant PDE1A isoform species from a cytosolic to membrane-bound isoform of the protein. Finally, as a convincing proof of principle, Tau (MAPT) shifts from a general cytoskeletal pattern in control samples towards a large presence in Fraction 6 in Alzheimer’s disease tissue, which contains large protein complexes such as ribosomes. This likely represents the increasing pool of insoluble paired helical fragment tau in Alzheimer’s disease tissue. Of these four potentially interesting proteins, Tau is the only one that looks like it may have an abundance difference in total tissue, which we plotted as the summed LFQ intensities from each sample (Fig. 3C).

## Discussion

High-throughput subcellular profiling of the proteome of the post-mortem human brain is complicated by the difficulties in isolating organelle-enriched fractions from frozen brain tissue. Here, we show a robust density gradient-based centrifugal separation of seven fractions using frozen human angular gyrus tissue from subjects with Alzheimer’s disease as well as healthy controls. The extent of separation of lower density membranous organelles was less clear than in fresh mouse brain,^15^ although it is unclear whether this is a result of post-mortem tissue freezing, or the extended fractionation scheme used by these investigators. These data suggest that spatial proteomic techniques can be used to assign a high-confidence subcellular location to approximately one-third of the robustly quantified proteins in human post-mortem brain tissue, a medium-confidence location to a further 40%, and that this can be used to highlight changes in localization profiles in response to a change in a disease condition. While this particular fractionation scheme works well for the separation of nuclear, mitochondrial and synaptic proteins from cytosolic, an extended fractionation scheme may be better placed to focus on organelles involved in protein trafficking.^15^

Using an SVM model, we could assign high-quality organelle predictions to a number of proteins that are not clearly annotated in the human brain. As expected, SVM accuracy was generally lower across all organelles in frozen tissue than in previous reports from cultured cells, including primary neurons.^13,14^ However, the performance of the model was not equal for all organelles and suffered from some limitations. While the SVM performed particularly well for nuclear, mitochondrial and cytosolic assignments, differentiation between ER and Golgi-associated proteins was not possible due to the small number of Golgi annotated proteins being detected in this experiment, where we performed relatively low-resolution single-shot label-free mass spectrometry. Future experiments with higher resolution mass spectrometry will therefore be needed to define the extent to which tissue freezing affects our ability to differentiate between ER proteins and Golgi in the later fractions.

Related to this point, the ability to train a model to assign organelles in the brain is also affected by the quality of reference annotation. In this work, we used organellar references that were mostly generated from single cultured cell types, such as Hela cells. In a complex tissue like the brain, which is composed of hundreds of specialized differentiated cell types,^24–26^ it is possible that proteins localize to different subcellular compartments than annotated, and in different locations in different cell types. To refine these references for improved utility in brain tissue, focused proteomic studies on specific cellular compartments from human and murine brain tissue will need to be coupled with orthogonal, lower-throughput tissue staining and electron microscopy experiments. Finally, the overlap in centrifugation patterns between the synaptic compartments and other organelles also complicated the clean annotation of synaptic proteins.

Despite the limitations to the SVM classification, the groups of SVM assigned organellar classifications were generally enriched for Gene Ontology terms that closely aligned with predicted organelle location, with the exception of the previously mentioned synaptic proteins. Finally, and somewhat surprisingly given the clear separation of cytoskeletal marker proteins in the first principal components, no clear enrichment was observed in the cytoskeletal assigned proteins. This organelle assignment tended to act as a ‘catch all’ category for proteins with no clear distribution pattern between the organelles. Due to the reference quality, it is currently not possible for us to hypothesize whether this is a function of tissue freezing and protein leaching during the PMI, and that this compartment will always be difficult to define, or whether curation of a larger training set of brain annotated proteins would enable separation of a cytoskeletal signal from this background noise.

Despite the limitations of the SVM approach in this proof-of-concept study, and the modest power of five subjects per diagnostic group, we were able to identify interesting and statistically significant patterns of disease-associated alterations in localization assignments for a number of proteins. These findings will require immunostaining experiments to follow up on their potential shift in organellar localization, and western blotting to address potential changes in isoform profile. While this pilot is a methodological proof of principle and therefore underpowered as a disease discovery approach, this is a promising result that indicates that this method may be used in larger-scale studies of human tissue to identify proteins whose localizations become dysregulated as a result of neurological disease.

## Supplementary Material

fcac103_Supplementary_Data

## Abbreviations

AGC =: automatic gain control
ANOVA =: analysis of variance
BCA =: bicinchoninic acid
BSA =: bovine serum albumin
*C* =: cost function
CNRIP =: cannabinoid receptor interacting protein 1
DAPI =: 4′,6-diamidino-2-phenylindole
EGTA =: 3,12-Bis(carboxymethyl)-6,9-dioxa-3,12-diazatetradecane-1,14-dioic acid
ER =: endoplasmic reticulum
GSK3β =: glycogen synthase kinase 3-beta
HCD =: higher energy C-trap dissociation
JACoP =: just another colocalization plugin
LC-MS/MS =: liquid chromatography–mass spectrometry
LFQ =: label-free quantification
MADRC =: Massachusetts Alzheimer’s Disease Research Center
MAPT =: microtubule-associated protein tau
Max =: maximum
Min =: minimum
MS =: mass spectrometry/mass spectrometer
NIA =: National Institute on Aging
PC =: principal component
PCA =: principal component analysis
PDE1A =: phosphodiesterase 1A
PMI =: post-mortem interval
RMSE =: root mean squares error
SD =: standard deviation
SVM =: support vector machine
SWIP =: wash complex subunit 4
TBS =: Tris-buffered saline
TDP-43 =: TAR DNA-binding protein 43
Tris–HCl =: Tris hydrochloride
UPLC =: ultra-performance liquid chromatography
WASH =: Wiskott–Aldrich syndrome protein and scar homologue complex
