# Supplementary material for: Proteomic characterization of post-mortem human brain tissue following ultracentrifugation-based subcellular fractionation
Source: Brain Commun. 2022 Apr 21;4(3):fcac103. doi: 10.1093/braincomms/fcac103 (PMC9123841; doi:10.1093/braincomms/fcac103)
Supplement: fcac103_Supplementary_Data [file fcac103_supplementary_data.zip › Original_Submission_(1).pdf]

OXFORD UNIVERSITY PRESS | Brain Communications

Proteomic Characterization of Post-Mortem Human Brain Tissue Following Ultracentrifugation-based Subcellular Fractionation

Journal:	*Brain Communications*
Manuscript ID	BRAINCOM-2021-281
Manuscript Type:	Original Article
Date Submitted by the Author:	13-Aug-2021
Complete List of Authors:	Kandigian, Savannah; Harvard Medical School; Massachusetts General Hospital Department of Neurology<br>Kitchen, Robert; Harvard Medical School Department of Medicine<br>Lam, TuKiet; Yale University School of Medicine, Keck MS & Proteomics Resource; Yale University School of Medicine, Department of Molecular Biophysics and Biochemistry<br>Arnold, Steven; Harvard Medical School; Massachusetts General Hospital Department of Neurology<br>Carlyle , Becky; Harvard Medical School; Massachusetts General Hospital Department of Neurology
Keywords:	Proteomics, Spatial Fractionation, Human Brain Tissue, Alzheimer's Disease
Note: The following files were submitted by the author for peer review, but cannot be converted to PDF. You must view these files (e.g. movies) online.	
Table S1_Maxquant output.txt	

SCHOLARONE™
Manuscripts

Proteomic Characterization of Post-Mortem Human Brain Tissue Following Ultracentrifugation-based Subcellular Fractionation

Kandigian SE[1], Kitchen RR[2], Lam TT[3,4], Arnold SE[1], Carlyle BC[1]*

[1]Harvard Medical School, Massachusetts General Hospital Department of Neurology, Charlestown, Boston, MA, USA

[2]Harvard Medical School Department of Medicine, Charlestown, Boston, MA, USA

[3]Keck MS & Proteomics Resource, Yale University School of Medicine, New Haven, CT, 06511, USA

[4]Dept. of Molecular Biophysics and Biochemistry, Yale University School of Medicine, New Haven, CT, 06511, USA

*corresponding author

For Review Only

Abstract

Proteomic characterization of human brain tissue is increasingly utilized to identify potential novel biomarker and drug targets for a variety of neurological diseases. In whole tissue studies, results may be driven by changes in the proportion of the largest and most abundant organelles or tissue cell-type composition. Spatial proteomics approaches enhance our knowledge of disease mechanisms and changing signaling pathways at the subcellular level by taking into account the importance of cellular localization, which critically influences protein function. Density gradient-based ultracentrifugation methods allow for subcellular fractionation and have been utilized in cell lines and fresh mouse brain tissue to predict the subcellular location of thousands of proteins, but to our knowledge have not been used in frozen human brain tissue. The use of frozen human tissue for tissue fractionation faces two major challenges, the post-mortem interval, during which proteins may leach from their usual location into the cytosol, and freezing, which results in membrane breakdown. Despite these challenges, in this proof-of-concept study, we show that the majority of proteins segregate reproducibly into crude density-based centrifugation fractions, that the fractions are enriched for the appropriate organellar markers, and that significant differences in protein localization can be observed between tissue from individuals with Alzheimer's Disease and control individuals.

For Review Only

Background

Proteomic characterization of brain tissue is increasingly utilized to investigate mechanisms and identify potential novel biomarker and drug targets for a variety of neurological diseases. Analysis of human brain tissue using liquid chromatography mass spectrometry (LC-MS) has revealed disease-related proteome differences beyond alterations found at the mRNA level in neurodegenerative diseases and neuropsychiatric disorders [1–4]. Spatial proteomics approaches (reviewed Lundberg and Borner, 2019) may further enhance our knowledge of disease mechanisms and changing signaling pathways at the subcellular level by taking into account the importance of cellular localization for protein function. Through fractionation of a sample into its organellar compartments prior to LC-MS/MS analysis, patterns of protein mis-localization in disease states may be identified. Furthermore, spatial fractionation prior to analysis by LC-MS/MS simplifies the input sample, and may enable more sensitive detection of low abundance proteins that might remain undetected in whole tissue LC-MS/MS.

Proteins can be active in multiple cellular localizations and the subcellular context critically influences protein function [6]. Subcellular localization of a given protein dictates pH of the reaction environment, the availability of molecular interaction partners, and the post-translational modification process. Dysregulation of protein localization can lead to loss of protein function or gain of toxic functions, and such changes have been linked to a number of human diseases [7,8]. Many neurodegenerative diseases are characterized by protein misfolding and accumulation in certain subcellular locations. Neumann et al. [7] showed that in patients with ALS, diseased neurons show redistribution of TDP-43 from the nucleus to the cytoplasm. In Alzheimer's disease (AD), numerous interacting pathways with varying subcellular localizations become dysregulated, and subcellular proteomics may enhance the specificity of our characterization of these pathways. For example, Shen et al. [8] recently showed that enrichment of mitochondrial, myelin sheath, and synaptosomal fractions from transgenic AD mouse model tissue was able to

For Review Only

identify compartment-specific alterations in disease-relevant pathways such as metabolism and synaptic dysfunction. Carlyle et al. [4] furthermore showed that enrichment of synaptosomes from post-mortem human brain tissue revealed a number of targets selectively associated with cognitive impairment in older individuals that were not revealed by whole tissue studies. These studies indicate the potential value of broader subcellular profiling of the AD brain.

In addition to the mechanistic insights enabled by spatial proteomics, these methods avoid volume confounds inherent in whole tissue proteomics. In whole tissue studies, results may be driven by the largest and most abundant organelles, and changes identified between brain regions may in fact reflect healthy differences in organellar composition [9], while disease related changes may refer to a difference in tissue cell-type composition. For example, the increased immune protein signal seen in a number of whole tissue studies [1–3] may reflect an increased volume of cortical tissue occupied by activated microglia or encroaching gliosis, rather than highlight the more subtle dysregulation of pathways and protein-protein networks. Spatial proteomics allows for the characterization of disease-associated changes in the proteomic makeup of an organelle type while controlling for the confound of changes in total abundance of those organelles. Alzheimer's disease, for example, is characterized by widespread synaptic loss [10–12]. Whole tissue proteomics may therefore show loss of synaptic proteins, but this does not lend insight into the makeup of remaining synapses and how synaptic dysfunction and degeneration progress.

Density gradient-based ultracentrifugation methods allow for subcellular fractionation and have been utilized in cell lines and fresh mouse brain tissue to predict the subcellular location of thousands of proteins [13,14], but to our knowledge have not been used in frozen human tissue. The use of frozen human tissue for tissue fractionation faces two major challenges, the post-mortem interval, during which proteins may leach from their usual location into the cytosol, and freezing, which results in membrane breakdown. Despite these challenges, in this pilot study

For Review Only

we show that the majority of proteins segregate reproducibly into crude density-based centrifugation fractions, that the fractions are enriched for the appropriate organellar markers, and that significant differences in protein localization can be detected between tissue from individuals with Alzheimer's disease compared to control individuals.

Results

Quality Control

Post-mortem frozen cortical tissue from 10 individuals was subcellularly fractionated by progressive ultracentrifugation into 6 membranous organellar fractions and a final cytosolic supernatant fractions (Figure 1A). All 70 of the resulting samples were analyzed by single-shot label free LC-MS/MS. 4367 proteins with at least 2 unique peptides were identified in at least one sample, and 853 proteins were identified in every sample (Table S1). Distribution of label-free quantification (LFQ) values across samples was even and required no further normalization (Figure S1). Because of the nature of the progressive removal of centrifuged pellets, it was expected that not all proteins would be identified in all fractions, particularly those proteins which localize to certain organelles. Therefore, in order to conduct statistically rigorous analyses without disregarding proteins that are appropriately undetected in certain fractions, we did not remove proteins which were not detected across all samples from our analysis. Only samples with greater than 60 missing values were filtered from the dataset, allowing a protein to be detected in all replicates from only one fraction. Once this filter was applied, 3097 proteins remained for analysis. Where necessary for modeling, missing values were assigned the lowest LFQ across all samples in which that protein was quantified.

Sample Clustering

For Review Only

A Principal Components Analysis (PCA) was conducted to examine how samples clustered according to protein abundance (Figure 1B). The PCA shows good stratification of samples by fraction, with clean clusters for fractions 4-7, while fractions 1-3 cluster more closely together. PC1 (accounting for 33.2% of the variance) mainly separated the cytosolic fraction (F7) from the organelle pellets (F1-6). PC2 (accounting for 20.6% of the variation) captures most of the separation between the remaining organelle fractions. This initial analysis showed that samples grouped together based on centrifugation fraction rather than subject.

To further analyze protein distribution across the samples, a heatmap was generated using all proteins identified (Figure S2). As expected, similarly to the PCA, the strongest effect was separation of the cytosolic fraction (F7) from the organelle pellets (F1-6). Other fractions, most notably fractions 1, 5, and 6, also showed good separation. In some instances, samples clustered primarily by individual subject as opposed to fraction. This can be seen in Figure S2, where fractions 2 and 3, and 5 and 6, are interspersed by subject. To improve clustering, we performed an analysis of variance (ANOVA) to identify proteins with significantly altered abundance between fractions. 2679 proteins were significantly differentially expressed (adjusted $p < 0.05$; Table S2). Post-hoc testing (Tukey MSD) revealed that the highest number of significant pairwise comparisons arose from the comparisons between fraction 7 and the other 6 fractions, again confirming that the cytosol is an outlier fraction (Figure 1C). Clustering using only these significant proteins substantially improved separation of the samples by fraction (Figure 1D), although the pairing of subjects remained in fractions 2 and 3. The successful stratification of the samples based on these significant proteins indicates that the fractions had qualitatively different protein distributions.

Marker Protein Distribution

To assess whether our fractionation scheme successfully sorted organelles into the predicted fractions, we examined the behavior of marker proteins known to localize to a singular

For Review Only

subcellular organelle. Given the lack of neuron specific organellar protein information available, we intersected the organelle lists used in Hela cells in Itzhak *et al* [13], and added pre- and post-synaptic terms from an inclusion list generated in Carlyle *et al* [4]. We generated an organelle marker map by performing a PCA on all proteins across all samples. Figure 2A represents the distribution of marker proteins along PC1 and PC2, with Figure 2B showing PC2 and PC3. Due to the overlapping of points in both plots, Figure S3 shows each marker class independently on individual plots. In agreement with the sample PCA and protein clustering, proteins from the cytosolic fraction are most clearly separated by the first two principal components, with PC2 separating markers of the large membranous organelles from cytosolic and cytoskeletal markers (Figure 2C). PC3 separates Mitochondria and Post-Synaptic markers from Plasma membrane and Pre-Synaptic markers, while PC4 clearly separates nuclear proteins from all other markers (Figure 2C). However, the smaller membranous organelles, as expected, show substantially more overlap, indicating that the ultracentrifugation-based approach is less able to cleanly enrich for these organelles. Assessment of these markers was also limited by a low number of markers from these organelles being detected in this single-shot proteomic experiment (Figure S3). Finally, we plotted the mean LFQ distribution between fractions of these markers proteins, and showed patterns similar to those seen in Hela cells [13] (Figure 2D). Synaptic proteins, which are not present in Hela cells, showed patterns of segregation almost identical to two other organelles, with post-synaptic proteins co-segregating with mitochondria, and pre-synaptic proteins segregating with Hela cell plasma membrane markers.

Localization prediction modeling

The organelle markers were used to predict localization of unannotated proteins using a support vector machine (SVM) model (R Caret package, [15]). The SVM was trained on a down-sampled set of marker proteins (n = 36 proteins per organelle), then used to classify all non-marker proteins to a likely cellular compartment. Performance of the SVM model on the training set was

For Review Only

high for the cytoplasm and nuclear fractions (mean percentage of proteins assigned to the appropriate organelle greater than 75%), intermediate for the ER, mitochondria, and cytoskeleton (> 50% appropriate assignment), and low for the pre- and post-synaptic fractions. There was no difference in performance between control and AD samples (Figure S4A), and performance was not affected by post-mortem interval (PMI, Figure S4B). There were insufficient markers available for the Golgi Apparatus and smaller membranous organelles (endolysosomes and peroxisomes) in this dataset, and therefore no categories for these organelles were generated by the model. Proteins were classified as high confidence (n = 1529) if the SVM placed them in the same organelles in at least 4 out of 5 control samples, medium confidence (n = 740) if there was a dominant location assignment across the 5 samples, and low confidence (n = 638) if organelle assignment was different in all 5 samples (Table S3). The 1529 high confidence proteins were then analyzed for cellular compartment enrichment using the R package TopGo (Table 1, [16]).

Fisher tests showed strong, appropriate enrichment for the nuclear ("Nuclear nucleosome", "Nucleoplasm"), Mitochondria ("Mitochondrial matrix", "Mitochondrial inner membrane"), and cytosol ("cytosol") assigned proteins. The endoplasmic reticulum (ER) protein set was strongly enriched for both ER ("endoplasmic reticulum membrane", "Endoplasmic reticulum") and Golgi terms ("COPI vesicle coat", "Golgi membrane"). This overlap may be in part due to the lack of a dedicated Golgi compartment in the model. Cytoskeleton was the poorest performing category, showing enrichment for many proteins that failed to show a clear distribution pattern across fractions. Finally, unsurprisingly, the model also performed worse at identifying dedicated post- and presynaptic categories, given their distribution closely follows that of the mitochondria and plasma membrane respectively (Figure 2D). Despite this difficulty in formally assigning a compartment to synaptic fractions the segregation pattern tends to be robust even for proteins

For Review Only

with low confidence in SVM organelle assignment, with the distribution of root mean squares error (RMSE) centering on 0.05 for high confidence proteins and 0.07 for low confidence proteins (Figure S3C). This suggests that for an average high confidence protein the ratio of across the fractions varies by approximately 5 % compared to the mean fractionation pattern, and by 7 % in low confidence proteins, which will still enable a change in fractionation pattern due to disease condition to be detected.

GO.ID	Term	Annotated	Significant	Expected	classicFisher	SVM assigned fraction
GO:0005829	cytosol	1489	120	94.32	7.10E-05	Cytoplasm
GO:0072562	blood microparticle	58	13	3.67	4.40E-05	Cytoplasm
GO:0035578	azurophil granule lumen	42	9	2.66	0.00097	Cytoplasm
GO:0120115	Lsm2-8 complex	5	3	0.32	0.00227	Cytoplasm
GO:0008537	proteasome activator complex	2	2	0.13	0.00399	Cytoplasm
GO:0005829	cytosol	1489	514	431.91	1.30E-08	Cytoskeleton
GO:0005634	nucleus	1192	421	345.76	4.60E-08	Cytoskeleton
GO:0070062	extracellular exosome	932	416	270.34	< 1e-30	Cytoskeleton
GO:0005925	focal adhesion	212	113	61.49	1.10E-14	Cytoskeleton
GO:1904813	ficolin-1-rich granule lumen	84	50	24.37	3.30E-09	Cytoskeleton
GO:0016021	integral component of membrane	591	95	52.98	1.10E-07	ER
GO:0005783	endoplasmic reticulum	332	57	29.76	6.90E-07	ER
GO:0005789	endoplasmic reticulum membrane	224	55	20.08	4.00E-11	ER
GO:0000139	Golgi membrane	155	33	13.9	1.00E-05	ER
GO:0030126	COPI vesicle coat	10	7	0.9	4.10E-06	ER
GO:0005743	mitochondrial inner membrane	195	36	5.6	5.90E-10	Mitochondria
GO:0005759	mitochondrial matrix	188	31	5.4	8.50E-13	Mitochondria
GO:0005758	mitochondrial intermembrane space	31	6	0.89	0.00019	Mitochondria
GO:0031305	integral component of mitochondrial inne...	17	6	0.49	0.00011	Mitochondria
GO:0017146	NMDA selective glutamate receptor comple...	5	3	0.14	0.00022	Mitochondria
GO:0005654	nucleoplasm	589	35	12.84	7.50E-09	Nucleus
GO:0000786	nucleosome	29	15	0.63	6.60E-06	Nucleus
GO:0005604	basement membrane	23	11	0.5	7.80E-07	Nucleus
GO:0000788	nuclear nucleosome	13	10	0.28	3.20E-15	Nucleus
GO:0043260	laminin-11 complex	3	3	0.07	9.90E-06	Nucleus
GO:0016020	membrane	1853	43	31.43	0.0123	Plasma membrane
GO:0005886	plasma membrane	981	29	16.64	0.0024	Plasma membrane
GO:0055038	recycling endosome membrane	32	3	0.54	0.0161	Plasma membrane
GO:0101003	ficolin-1-rich granule membrane	22	3	0.37	0.0056	Plasma membrane
GO:0099092	postsynaptic density, intracellular comp...	15	3	0.25	0.0018	Plasma membrane
GO:0005739	mitochondrion	566	18	4.7	0.0018	Postsynapse
GO:0005743	mitochondrial inner membrane	195	9	1.62	6.70E-05	Postsynapse
GO:0005759	mitochondrial matrix	188	6	1.56	0.0126	Postsynapse
GO:0099629	postsynaptic specialization of symmetric...	2	1	0.02	0.0165	Postsynapse
GO:0002169	3-methylcrotonyl-CoA carboxylase complex...	2	1	0.02	0.0165	Postsynapse
GO:0005925	focal adhesion	212	7	1.39	0.00024	Presynapse
GO:0035579	specific granule membrane	33	3	0.22	0.00116	Presynapse
GO:0070821	tertiary granule membrane	24	3	0.16	0.00045	Presynapse
GO:0031209	SCAR complex	7	2	0.05	0.00084	Presynapse
GO:0032433	filopodium tip	7	2	0.05	0.00084	Presynapse

For Review Only

Table 1: The top five enriched GO terms for each set of SVM assigned organelles. *All terms are significantly enriched (classicFisher < 0.05). The table shows proteins annotated ("Annotated) in that GO term, compared to proteins present in that organellar set ("Significant"). For enrichment to be significant, the number of proteins in set will be greater than those expected by chance in a dataset of this size ("Expected").*

Disease-associated changes in protein localization

As a proof of concept, to define whether different protein segregation patterns were detectable between control (Braak stages 1-3) and AD (Braak 4-6) tissue (Table 2), we ranked proteins by their difference in global delta-entropy (see methods) between AD and control and performed multiple corrected within-fraction t-tests to identify the fraction where differences in protein distribution were significant.

	AD (N=5)	C (N=5)	Overall (N=10)
**Age**			
Mean (SD)	89.8 (2.77)	90.2 (3.42)	90.0 (2.94)
Median [Min, Max]	91.0 [86.0, 93.0]	91.0 [85.0, 94.0]	91.0 [85.0, 94.0]
**PMI**			
Mean (SD)	15.0 (4.80)	15.0 (5.96)	15.0 (5.10)
Median [Min, Max]	12.0 [12.0, 23.0]	13.0 [8.00, 23.0]	12.5 [8.00, 23.0]

Table 2: Demographics of the AD versus control sample set. *PMI = post-mortem interval in hours.*

Microtubule Associated Protein Tau was the 6th ranked protein for global entropy differences between conditions, and 85 proteins had significant differences between AD and control in at least one fraction. From these lists we identified several interesting candidates exhibiting likely altered subcellular localization (Fig 3A-B). Cannabinoid Receptor Interacting Protein 1 (CNRIP) is high in the cytosol and low in the nucleus in control tissue, whereas in AD tissue it is more strongly associated with the membrane fractions 2 and 3. GSK3β is a kinase linked to tau phosphorylation and associated with psychiatric disease susceptibility that functions as a major point of integration for critical neuronal signaling pathways. It also has a function in the nucleus, where it acts to regulate transcription of a number of genes, including those in the Wnt signaling pathway [17,18]. In control samples, GSK3β was most strongly associated with the nuclear fraction, and in AD samples this nuclear association was much smaller. Phosphodiesterase 1A (PDE1A) localizes to the nucleus and large membrane fractions in both conditions, but there is a large cytosolic pool in controls absent in AD. In this example we may not be seeing mis-localization of PDE1A, but a change in predominant PDE1A isoform species from a cytosolic to membrane bound isoform of the protein. Finally, as a convincing proof of principle, Tau (MAPT) shifts from a general cytoskeletal pattern in control samples towards a large presence in fraction 6 in AD tissue, which contains large protein complexes such as ribosomes. This likely represents the increasing pool of insoluble PHF-tau in AD tissue. Of these 4 potentially interesting proteins, Tau is the only one that looks like it may have an abundance difference in total tissue, which we plotted as the summed LFQ intensities from each sample (Figure 3C).

Discussion

For Review Only

High throughput subcellular profiling of the proteome of the post-mortem human brain is complicated by the difficulties in isolating organelle-enriched fractions from frozen brain tissue. Here, we show robust density-gradient based centrifugal separation of 7 fractions using frozen human angular gyrus tissue from subjects with AD as well as healthy controls. These data suggest that spatial proteomic techniques can be used to confidently assign a subcellular location to the majority of robustly quantified proteins in human post-mortem brain tissue, and that it can be used to highlight changes in localization profiles in response to a change in disease condition.

Using a SVM model, we could assign high-quality organelle predictions to a number of proteins that are not clearly annotated in human brain. However, the performance of the model was not equal for all organelles and suffered from some limitations. While the SVM performed particularly well for nuclear, mitochondrial, and cytosolic assignments, differentiation between ER and Golgi-associated proteins was not possible due to the small number of Golgi annotated proteins being detected in this experiment, where we performed relatively low-resolution single shot label free mass-spectrometry. Future experiments with higher resolution mass-spectrometry will therefore be needed to define the extent to which tissue freezing affects our ability to differentiate between ER proteins and Golgi in the later fractions.

Related to this point, the ability to train a model to assign organelles in brain is also affected by the quality of reference annotation. In this work we used organellar references that were mostly generated from single cultured cell types, such as Hela cells. In a complex tissue like the brain, which is composed of hundreds of specialized differentiated cell-types [19–21], it is possible that proteins localize to different subcellular compartments than annotated, and in different locations in different cell-types. To refine these references for improved utility in brain tissue, focused proteomic studies on specific cellular compartments from human and murine brain tissue will need to be coupled with orthogonal, lower-throughput tissue staining and electron-microscopy

For Review Only

experiments. Finally, the overlap in centrifugation patterns between the synaptic compartments and other organelles also complicated the clean annotation of synaptic proteins.

Despite the limitations to the SVM classification, the groups of SVM assigned organellar classifications were generally enriched for Gene Ontology terms that closely aligned with predicted organelle location, with the exception of the previously mentioned synaptic proteins. Finally, and somewhat surprisingly given the clear separation of cytoskeletal marker proteins in the first principal components, no clear enrichment was observed in the cytoskeletal assigned proteins. This organelle assignment tended to act as a “catch all” category for proteins with no clear distribution pattern between the organelles. Due to the reference quality, it is currently not possible for us to hypothesize whether this is a function of tissue freezing and protein leaching during the post-mortem interval, and that this compartment will always be difficult to define, or whether curation of a larger training set of brain annotated proteins would enable separation of a cytoskeletal signal from this background noise.

Despite the limitations of the SVM approach in this proof-of-concept study, and modest power of 5 subjects per diagnostic group, we were able to identify interesting and statistically significant patterns of disease-associated alterations in localization assignments for a number of proteins. These findings will require immunostaining experiments to follow up their potential shift in organellar localization, and western blotting to address potential changes in isoform profile. While this pilot is a methodological proof of principle and therefore underpowered as a disease discovery approach, this is a promising result that indicates that this method may be used in larger scale studies of human tissue to identify proteins whose localizations become dysregulated as a result of neurological disease.

For Review Only

Methods

Samples

Frozen post-mortem human brain tissue was obtained from the Massachusetts Alzheimer's Disease Research Center (MADRC). Experiments were conducted in accordance with the Partners Healthcare IRB. 200 mg sections of angular gyrus tissue were sectioned on dry ice from 5 subjects diagnosed with Alzheimer's disease and 5 age, sex, and post-mortem interval-matched controls. Demographic data for the sample is shown in Table 2.

Fractionation

Methods were adapted from Izthak et al. (2016). Samples were homogenized with a dounce homogenizer using 15 strokes at 800 rpm in lysis buffer (25 mM Tris-HCl, 50mM sucrose, 0.5 mM MgCl2, 0.2 mM EGTA) with protease inhibitors. Following homogenization, sucrose concentration was readjusted to 250mM. All centrifugation steps were carried out at 4 ^{o}C in a benchtop microcentrifuge (< 24,000xg) or a Beckman Coulter Ultracentrifuge (>24,000xg) using an MLA-50 rotor (Beckman Coulter) and 10ml polypropylene tubes. Pellets and supernatants were handled on ice at all times between spins. A schematic of the differential centrifugation protocol, including times and speeds, is shown in Figure 1A. Following each centrifugation step, the supernatant was transferred to a new tube and pellets were frozen at -80C. The final supernatant, representing the cytosolic fraction, underwent acetone precipitation as follows: 400μl of supernatant was mixed with 1.6 mls of pre-cooled acetone and incubated for one hour at -20 ^{o}C. The resulting pellet was resuspended in solubilization buffer (8 M urea , 0.4 M ammonium bicarbonate) and frozen at -80C.

Sample preparation for Mass Spectrometry

For Review Only

Pellets were resuspended in solubilization buffer (8 M urea, 0.4 M ammonium bicarbonate) with protease inhibitors and briefly sonicated. Samples were cleared by centrifugation, total protein content was assessed by BCA, and each sample was adjusted to 100 μg/50μl of solubilization buffer. For samples with protein contents below this threshold, 50 μl of the straight sample was used. Following reduction with dithiothreitol (45 mM at 1/10th sample volume) for 30 minutes at room temperature and alkylation with iodoacetamide (100 mM at 1/10th sample volume) for 30 minutes in the dark at room temperature, samples were trypsin digested overnight with a 1:20 protein:enzyme ratio. Samples were acidified to stop digestion, desalted on C18 Microspin columns (Nest Group) and dried in a SpeedVac. Resulting pellets were frozen at -80 ^{o}C until transport.

Liquid chromatography tandem mass spectrometry

LC-MS/MS analysis was performed on a Thermo Scientific Orbitrap Fusion equipped with a Waters nanoAcquity UPLC system utilizing a binary solvent system (Buffer A: 100% water, 0.1% formic acid; Buffer B: 100% acetonitrile, 0.1% formic acid). Trapping was performed at 5μl/min, 97% Buffer A for 3 min using a Waters Symmetry® C18 180μm x 20mm trap column. Peptides were separated using an ACQUITY UPLC PST (BEH) C18 nanoACQUITY Column 1.7 μm, 75 μm x 250 mm (37ºC) and eluted at 300 nl/min with the following gradient: 3% buffer B at initial conditions; 6% B at 5 minute; 35% B at 170 minute; 50% B at 175 minutes; 97% B at 180 minutes; 97% B at 185 minutes; return to initial conditions at 186-200 minutes. MS was acquired in the Orbitrap in profile mode over the 350-1,550 m/z range using wide quadrapole isolation, 1 microscan, 120,000 resolution, AGC target of 4E5, and a maximum injection time of 60 ms. Data dependent MS/MS were collected in top speed mode with a 3s cycle time on species with an intensity threshold of 5E4, charge states 2-8, peptide monoisotopic precursor selection preferred. Dynamic exclusion was set to 30 seconds. MS/MS were acquired in the Orbitrap in centroid mode using quadropole isolation (window 1.6m/z), HCD activation with a

For Review Only

collision energy of 28%, 1 microscan, 60,000 resolution, AGC target of 1E5, maximum injection time of 110 ms.

Data Analysis

Raw mass spectrometry data were processed in MaxQuant using default settings and the addition of the 'match between runs' feature. All downstream analyses were performed in R (v4.0.2), and the tidyverse (v1.3.0), UpSetR (v1.4.0) , Caret (v6.0-86), Openxlsx (v.4.2.3) and TopGo (v2.40.0 with AnnotationDbi v1.50.3) packages. Filtering of this dataset was minimal, as the fractions were expected to have quite different sets of proteins residing in them. Proteins with over 60% missing values were excluded from further analysis, with missing values set as NAs or set as the minimum LFQ detected for that protein depending on the plotting or statistical method to be employed. No further normalization was applied post MaxQuant processing. PCA was performed using the prcomp function in base R. All plotting was performed using ggplot2 functions, except from heatmaps which were produced with the heatmap.2 function, and the UpSet plot (Figure 1C) which was produced using UpSetR. Unless stated otherwise, all references to significance refer to p values adjusted using the Benjamini-Hochberg Method [22].

Support Vector Machine (SVM) supervised learning was used to classify proteins to organelles and was performed on each biological sample individually. Training was performed on centered and scaled LFQ values with a radial basis function kernel and, to ensure balanced classes, marker proteins were randomly down-sampled to 36 proteins in each marker set. 10-fold cross-validation was used to minimize over-fitting and we used 10 tuning levels for sigma (a.k.a. gamma) and the cost function, C. Performance of the training set was simplified to a single summary percentage of correctly assigned proteins per organelle set. Classification was

For Review Only

performed on a sample-by-sample basis on all proteins that were not included in the training set for that sample.

Global entropy values were calculated on the proportions of total LFQ signal present in each fraction for each protein and subject. We used Shannon's definition of entropy (calculated as: entropy = -sum(ratio x log2(ratio))/log2(n))) to measure the degree of disorder of each protein in each subject relative to the subcellular fractions. Low entropy values denote proteins highly ordered – i.e. those specific to a small number of fractions. For the purpose of these calculations NA values were set as 0.0000000001. Global delta entropy was calculated as the difference between the mean entropy values for control subjects and the mean entropy values for the AD subjects.

For Review Only

Competing interests

The authors declare that they have no competing interests.

Funding

This study was funded by a pilot award from NIA parent award AG062421.

Authors' contributions

SEK performed the fractionation experiments and initial data inspection in Perseus, and drafted the manuscript.

RRK supervised the analysis and contributed to writing the manuscript

TTL supervised and strategized the MS data collection, and reviewed the manuscript

SEA provided the lab space for this work and reviewed the manuscript

BCC obtained the funding, designed the experiments, performed the downstream analysis, and co-wrote the manuscript.

All authors read and approved the final manuscript.

Acknowledgements

Thank you to the individuals who donated their brains for scientific research, and thank you to the Massachusetts Alzheimer's Disease Research Center Neuropathology core who provided the tissue samples used. Thank you to Jean Kanyo at the Keck MS & Proteomics Resource at Yale for the MS data collection and initial MS analyses. The Orbitrap Fusion mass

For Review Only

spectrometer utilized was supported in part by NIH SIG grants S10OD018034 and Yale School of Medicine. The funders had no role in study design, data collection and analysis, decision to publish, or preparation of the manuscript. Funding for this project came from a pilot award from the Massachusetts Alzheimer's Disease Research Center, parent grant AG062421. BCC is supported by the Bright Focus Foundation.

The mass spectrometry proteomics data have been deposited to the ProteomeXchange Consortium via the PRIDE partner repository with the dataset identifier PXD027456.

Prior to publication these data can be accessed used the reviewer account details:

Username: reviewer_pxd027456@ebi.ac.uk

Password: jO9c5azj

Code for the analysis of this project is available at: https://github.com/ACTRU/becky-carlyle-fractionation-MS

References

1. Johnson, E. C. B. *et al.* Large-scale proteomic analysis of Alzheimer's disease brain and cerebrospinal fluid reveals early changes in energy metabolism associated with microglia and astrocyte activation. *Nat. Med.* **26**, 769–780 (2020).

2. Johnson, E. C. B. *et al.* Deep proteomic network analysis of Alzheimer's disease brain reveals alterations in RNA binding proteins and RNA splicing associated with disease. *Mol. Neurodegener.* **13**, 52 (2018).

For Review Only

3. Bai, B. *et al.* Deep Multilayer Brain Proteomics Identifies Molecular Networks in Alzheimer's Disease Progression. *Neuron* **105**, 975-991.e7 (2020).

4. Carlyle, B. C. *et al.* Synaptic proteins associated with cognitive performance and neuropathology in older humans revealed by multiplexed fractionated proteomics. *Neurobiol. Aging* (2021) doi:10.1016/j.neurobiolaging.2021.04.012.

5. Lundberg, E. & Borner, G. H. H. Spatial proteomics: a powerful discovery tool for cell biology. *Nature Reviews Molecular Cell Biology* vol. 20 285–302 (2019).

6. Hung, M. C. & Link, W. Protein localization in disease and therapy. *J. Cell Sci.* **124**, 3381–3392 (2011).

7. Neumann, M. *et al.* Ubiquitinated TDP-43 in frontotemporal lobar degeneration and amyotrophic lateral sclerosis. *Science (80-.).* **314**, 130–133 (2006).

8. Shen, L. *et al.* Proteomic Profiling of Cerebrum Mitochondria, Myelin Sheath, and Synaptosome Revealed Mitochondrial Damage and Synaptic Impairments in Association with 3 × Tg-AD Mice Model. *Cell. Mol. Neurobiol.* (2021) doi:10.1007/s10571-021-01052-z.

9. Carlyle, B. C. *et al.* A multiregional proteomic survey of the postnatal human brain. *Nat. Neurosci.* **20**, 1787–1795 (2017).

10. DeKosky, S. T. & Scheff, S. W. Synapse loss in frontal cortex biopsies in Alzheimer's disease: Correlation with cognitive severity. *Ann. Neurol.* **27**, 457–464 (1990).

11. Koffie, R. M., Hyman, B. T. & Spires-Jones, T. L. Alzheimer's disease: synapses gone cold. *Mol. Neurodegener.* **6**, 63 (2011).

12. Terry, R. D. *et al.* Physical basis of cognitive alterations in alzheimer's disease: Synapse loss is the major correlate of cognitive impairment. *Ann. Neurol.* **30**, 572–580 (1991).

For Review Only

13. Itzhak, D. N., Tyanova, S., Cox, J. & Borner, G. H. H. Global, quantitative and dynamic mapping of protein subcellular localization. *Elife* **5**, (2016).

14. Itzhak, D. N. *et al.* A Mass Spectrometry-Based Approach for Mapping Protein Subcellular Localization Reveals the Spatial Proteome of Mouse Primary Neurons. *Cell Rep.* **20**, 2706–2718 (2017).

15. Kuhn, M. Building predictive models in R using the caret package. *J. Stat. Softw.* **28**, 1–26 (2008).

16. Alexa, A. & Rahnenfuhrer, J. topGO: Enrichment Analysis for Gene Ontology. *R Packag. version 2.36.0* (2019).

17. Bijur, G. N. & Jope, R. S. Glycogen synthase kinase-3 beta is highly activated in nuclei and mitochondria. *Neuroreport* **14**, 2415–2419 (2003).

18. Caspi, M., Zilberberg, A., Eldar-Finkelman, H. & Rosin-Arbesfeld, R. Nuclear GSK-3β inhibits the canonical Wnt signalling pathway in a β-catenin phosphorylation-independent manner. *Oncogene* **27**, 3546–3555 (2008).

19. Zeisel, A. *et al.* Brain structure. Cell types in the mouse cortex and hippocampus revealed by single-cell RNA-seq. *Science* **347**, 1138–42 (2015).

20. Lake, B. B. *et al.* Integrative single-cell analysis of transcriptional and epigenetic states in the human adult brain. *Nat. Biotechnol.* **36**, 70–80 (2018).

21. Mathys, H. *et al.* Single-cell transcriptomic analysis of Alzheimer's disease. *Nature* **570**, 332–337 (2019).

22. Benjamini, Y. & Hochberg, Y. Controlling the False Discovery Rate: A Practical and Powerful Approach to Multiple Testing on JSTOR. *J. R. Stat. Soc. Ser. B* **57**, 289–300 (1995).

For Review Only

Figure legends

Main figures

Figure 1: Differential centrifugation can be used to separate proteins into consistent fractions in post-mortem human brain. A) Schematic of the centrifugation scheme used to prepare samples for this experiment. Centrifuge speeds and spin times are provided. All spins were performed at 4°C **B)** Principal Component analysis shows good separation of samples by centrifugation fraction in the first two principal components. **C)** An Upset plot shows that most proteins in the dataset differentially expressed by ANOVA are differentially abundant between fraction 7 (cytosolic fraction) and fraction 6 (large protein complex fraction) and all other fractions. **D)** A heatmap of differentially expressed proteins shows that samples generally cluster on the basis of centrifugation fraction. The exception is fractions 2 and 3, where samples from the same individual cluster in pairs within the larger cluster. Color coding of samples in the horizontal bar is identical to color coding in plot 1B.

Figure 2: Established markers proteins segregate according to reproducible patterns across fractions. A) Marker proteins plotted according to their locations along PC1 and PC2. Figure S3 shows a faceted plot of each set of organellar markers alone, to inspect proteins in the crowded central region of the plot. The combination of PC3 1 & 2 shows good separation

For Review Only

for cytoskeletal and cytosolic proteins in the lower part of PC2, and mitochondrial and post-synaptic proteins in the top half. **B)** PC plot of PCs 2 and 3. PC3 nicely separates post-synaptic and mitochondrial proteins from pre-synaptic and plasma membrane proteins. **C)** Box plots shows the association of marker proteins from each organelle with the top 4 principal components. PC4 separates the nuclear markers from all other organelles. **D)** Line plot of the average proportional distribution profile of the marker set for each organelle.

Figure 3: Changes in protein distribution pattern between control and AD individuals can be detected in this dataset. A) Boxplots of log2LFQ values for the 4 proteins of interest; raw LFQ values can be variable between samples in the same diagnostic group. **B)** Boxplots of proportional ratios of protein in each of the 7 fractions; variation between samples is much lower when fractionation patterns are expressed as a ratio. Significant differences between AD and controls (adjusted $p < 0.05$) in single fractions are noted with an asterisk. The horizontal black line shows the segregation pattern of a protein of zero entropy. **C)** Summed LFQs across all fractions show a low likelihood of differential expression from these proteins across the whole tissue.

Supplementary Figures

Figure S1: Per sample LFQ values show consistent LC-MS/MS results through the experiment. No further normalization was performed on these values.

For Review Only

Figure S2: All protein clustering shows reasonable clustering of samples by fraction. In fractions 2 & 3 and 5 & 6 samples are clustered by subject within the larger fraction cluster. Subject is denoted by the letter at the end of the sample name.

Figure S3: Faceted PCA plots from Figure 2A . A) Faceted PCA plots for PC1 and PC2 enables inspection of protein groups in crowded central region of plot. **B)** Faceted PCA plots for PC2 and PC3

Figure S4: SVM performance is not affected by sample demographics. A) Boxplots showing performance (% of proteins assigned to correct organelle) of the SVM model for each organelle, split by AD and control samples. **B)** SVM performance does not decrease as post-mortem interval increases. **C)** High confidence proteins segregate reproducibly in the same patterns regardless of diagnostic condition, as the RMSE distribution is centered at 0.05 (a 5% variation from the mean segregation pattern for that protein). Medium and low confidence proteins are slightly more variable.

For Review Only

For Review Only

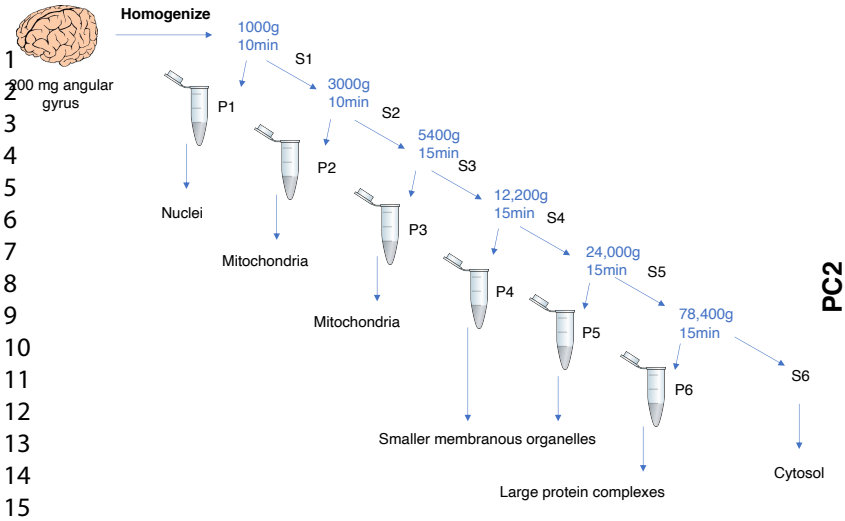
Homogenize
200 mg angular gyrus
1000g 10min
S1
P1
Nuclei
3000g 10min
S2
P2
Mitochondria
5400g 15min
S3
P3
Mitochondria
12,200g 15min
S4
P4
Smaller membranous organelles
24,000g 15min
S5
P5
Large protein complexes
78,400g 15min
S6
P6
Cytosol

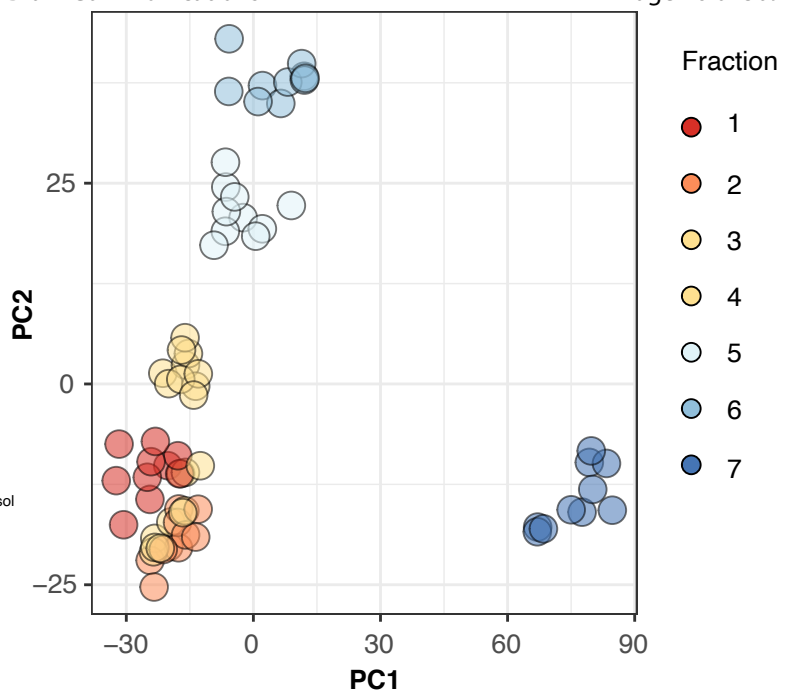
PC2
PC1
25
0
−25
−30
0
30
60
90
Fraction
1
2
3
4
5
6
7

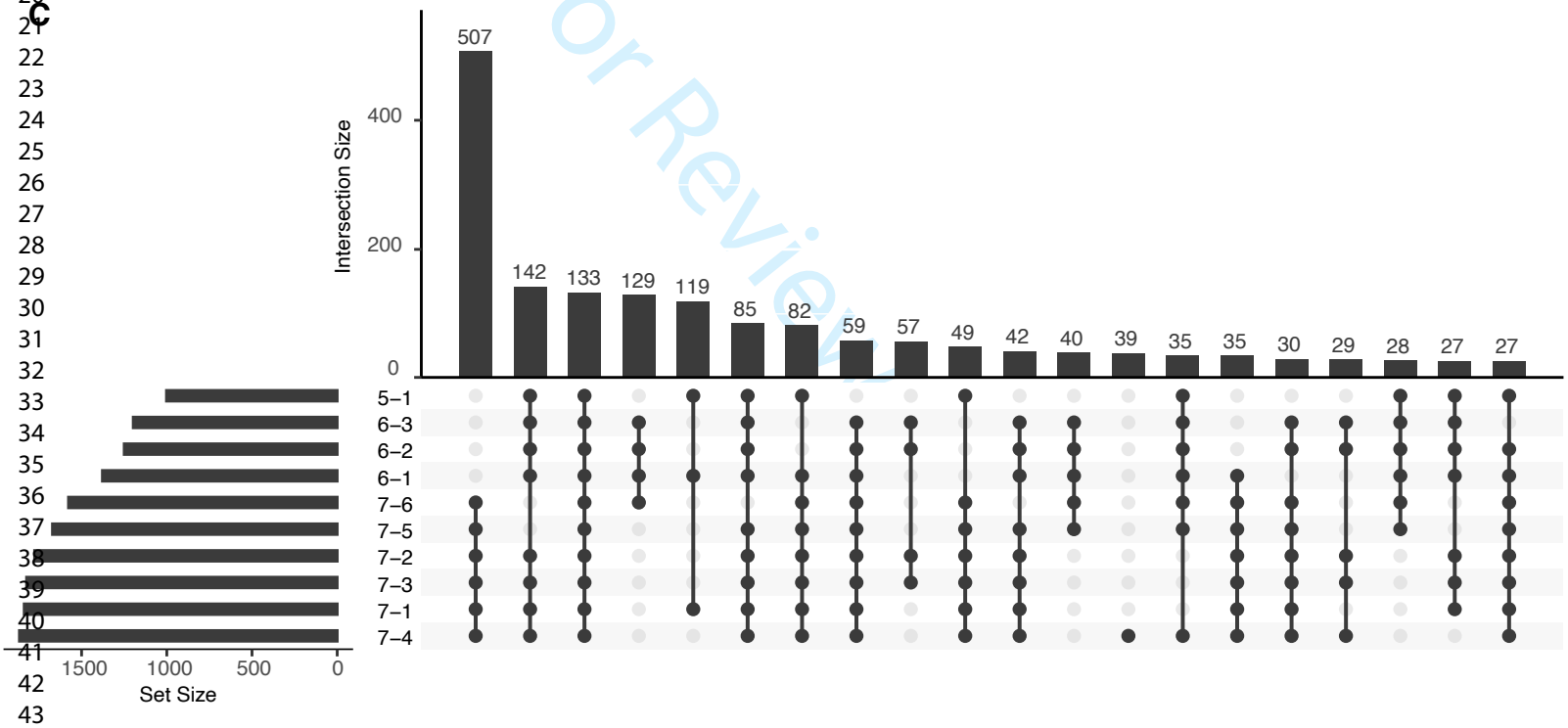
Intersection Size
507
142
133
129
119
85
82
59
57
49
42
40
39
35
35
30
29
28
27
27
400
200
0
5–1
6–3
6–2
6–1
7–6
7–5
7–2
7–3
7–1
7–4
1500
1000
500
0
Set Size

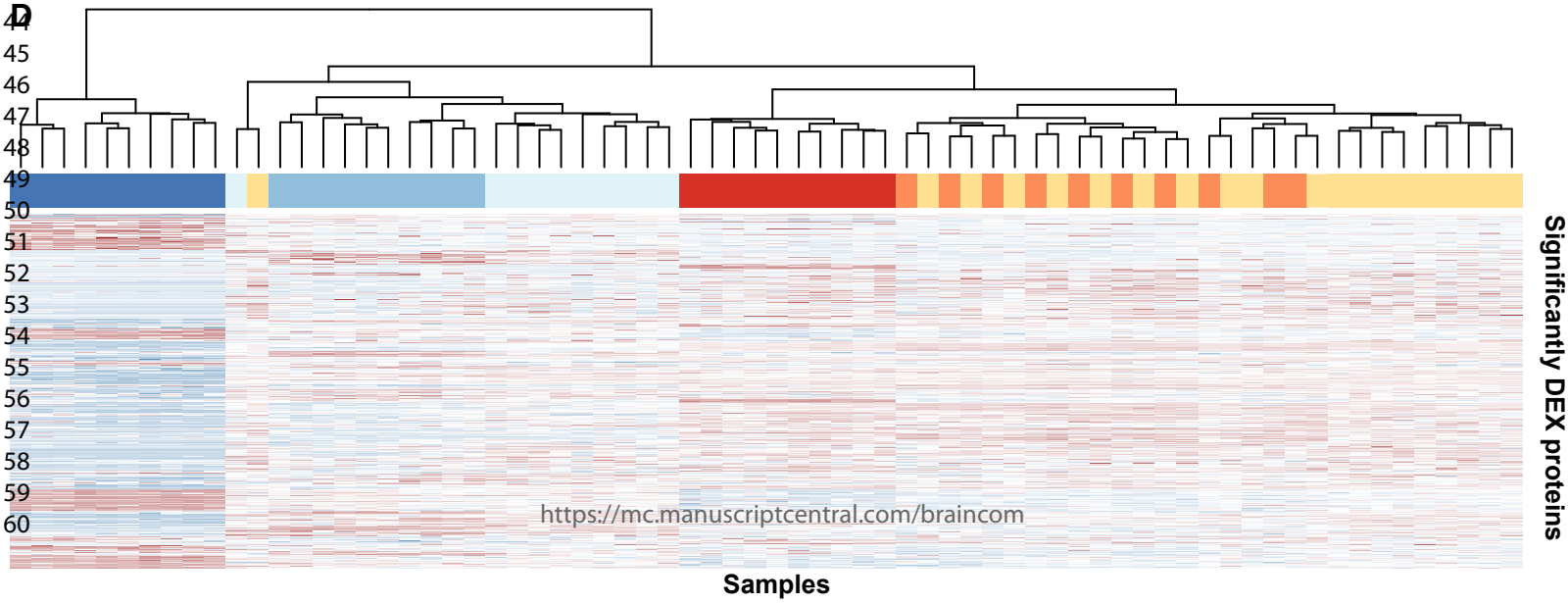
Significantly DEX proteins
Samples

For Review

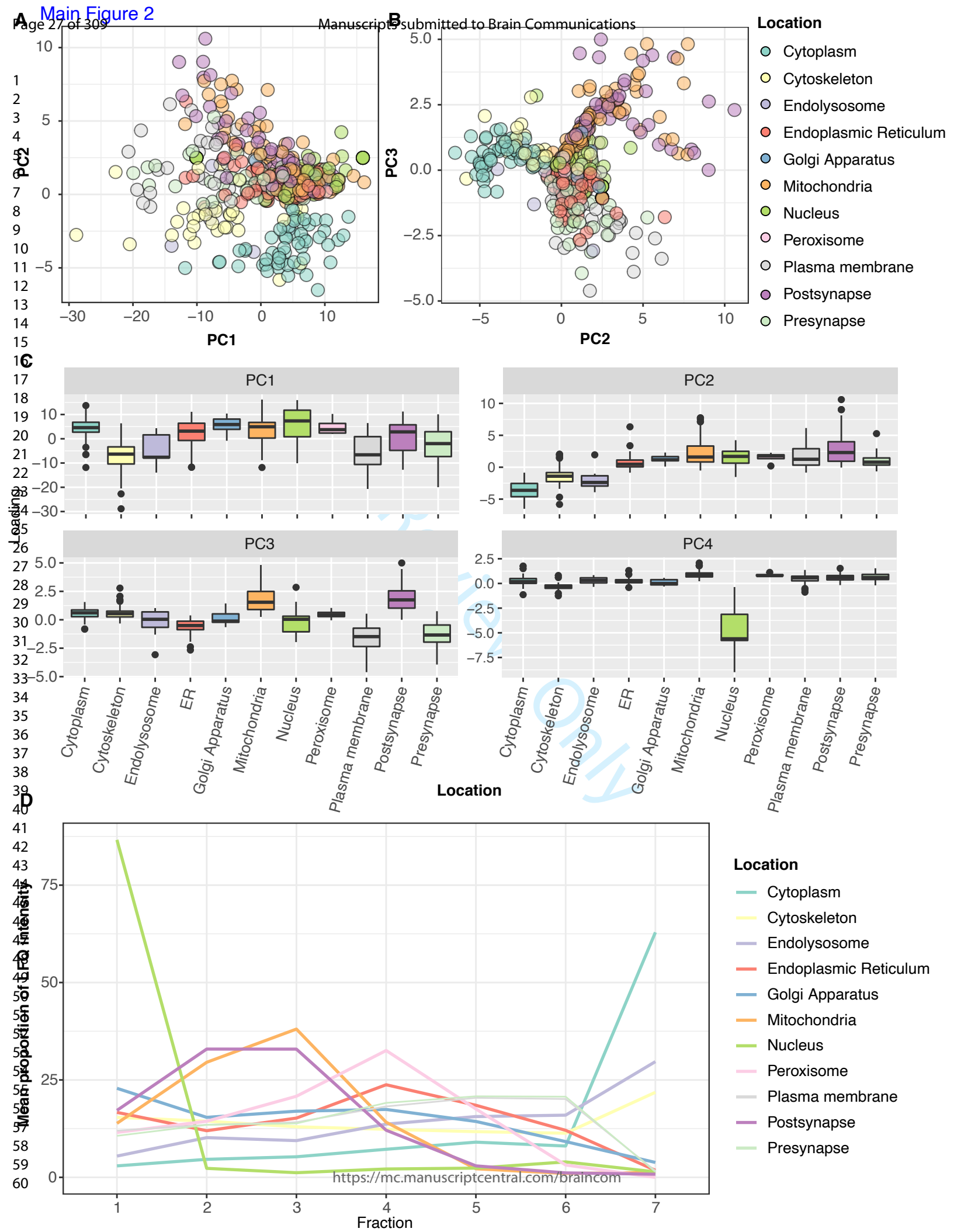

https://mc.manuscriptcentral.com/braincom
For Review Only

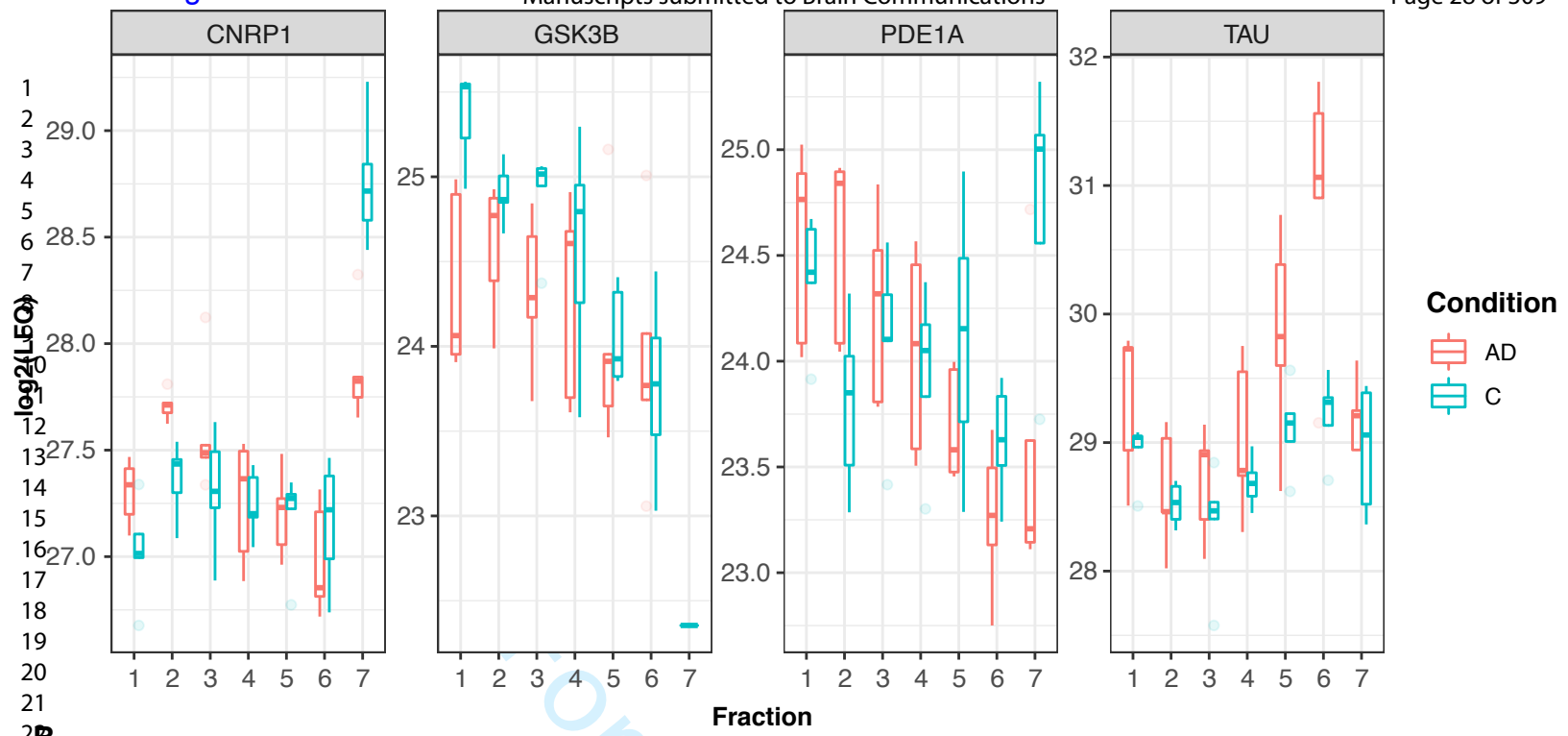

B

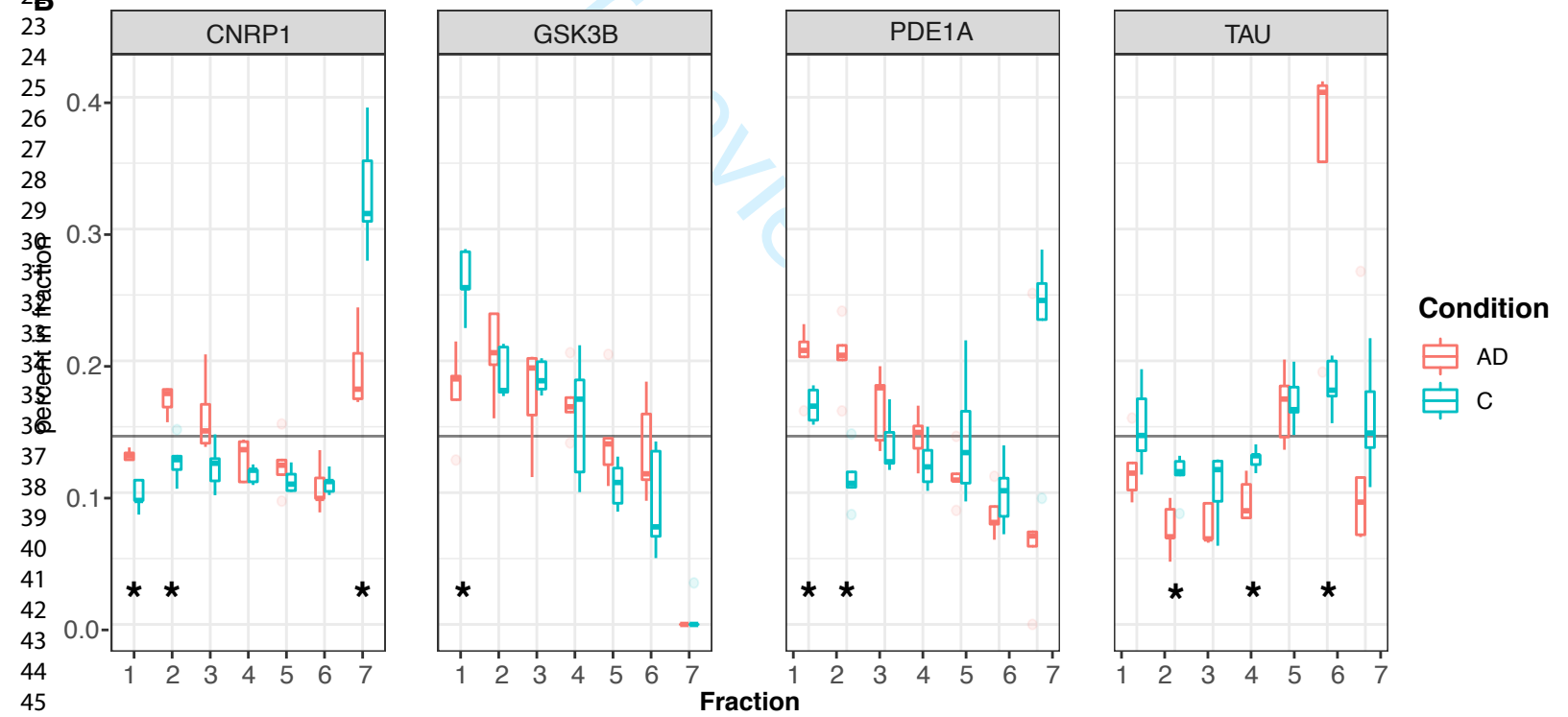

C

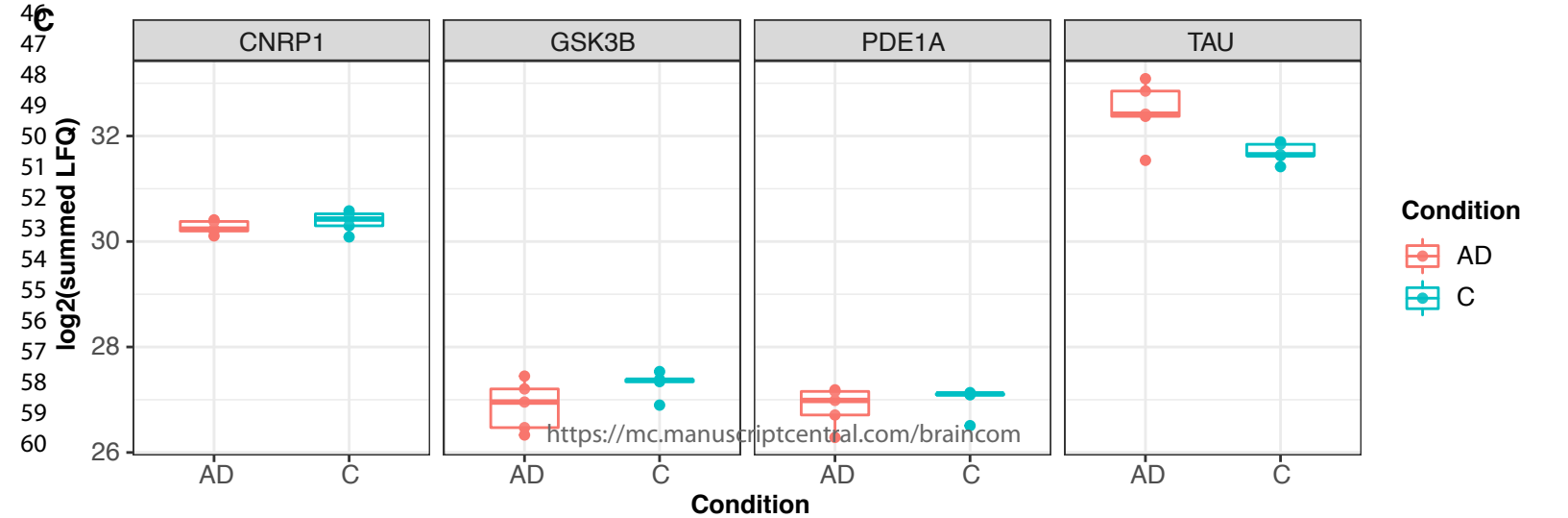

https://mc.manuscriptcentral.com/braincom

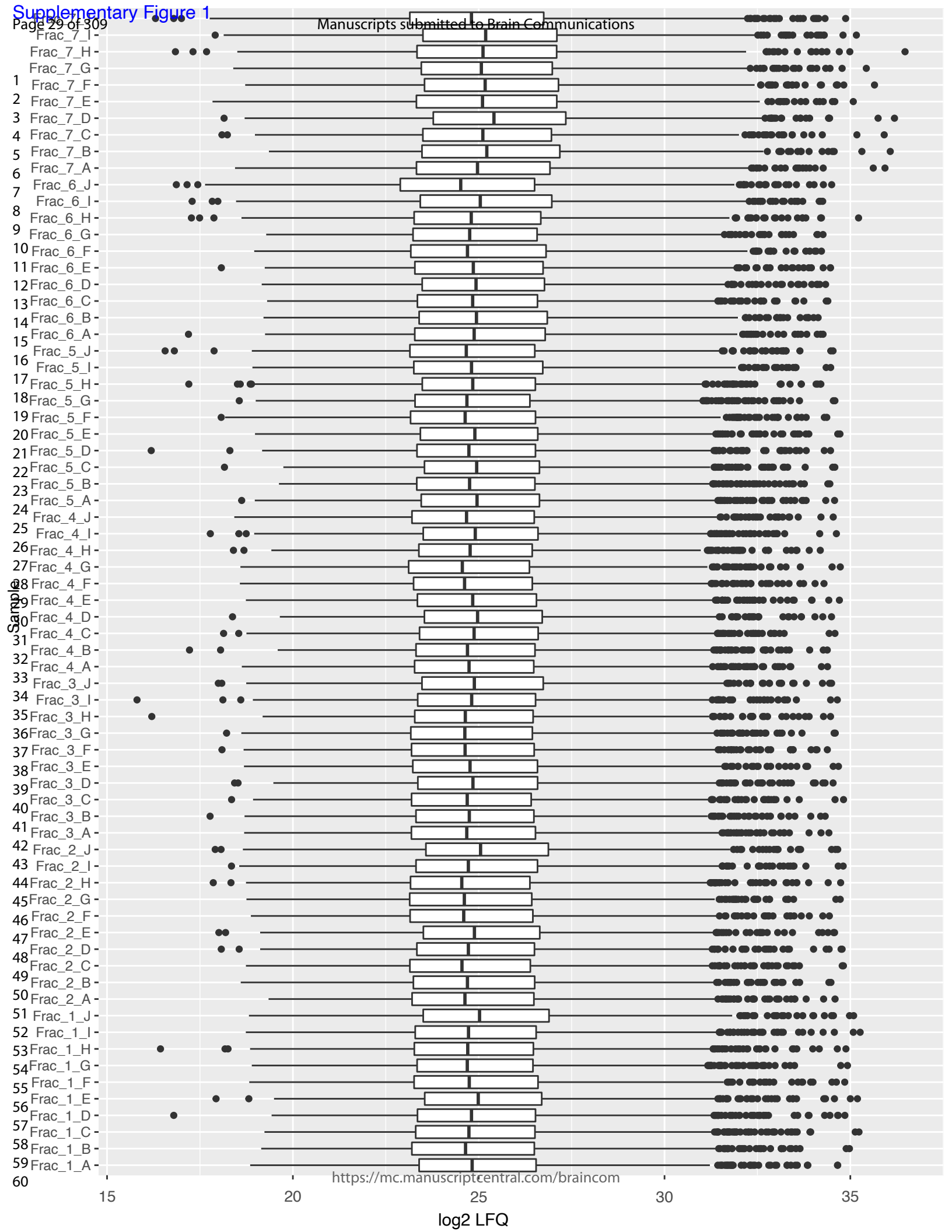
Frac_7_I
Frac_7_H
Frac_7_G
Frac_7_F
Frac_7_E
Frac_7_D
Frac_7_C
Frac_7_B
Frac_7_A
Frac_6_J
Frac_6_I
Frac_6_H
Frac_6_G
Frac_6_F
Frac_6_E
Frac_6_D
Frac_6_C
Frac_6_B
Frac_6_A
Frac_5_J
Frac_5_I
Frac_5_H
Frac_5_G
Frac_5_F
Frac_5_E
Frac_5_D
Frac_5_C
Frac_5_B
Frac_5_A
Frac_4_J
Frac_4_I
Frac_4_H
Frac_4_G
Frac_4_F
Frac_4_E
Frac_4_D
Frac_4_C
Frac_4_B
Frac_4_A
Frac_3_J
Frac_3_I
Frac_3_H
Frac_3_G
Frac_3_F
Frac_3_E
Frac_3_D
Frac_3_C
Frac_3_B
Frac_3_A
Frac_2_J
Frac_2_I
Frac_2_H
Frac_2_G
Frac_2_F
Frac_2_E
Frac_2_D
Frac_2_C
Frac_2_B
Frac_2_A
Frac_1_J
Frac_1_I
Frac_1_H
Frac_1_G
Frac_1_F
Frac_1_E
Frac_1_D
Frac_1_C
Frac_1_B
Frac_1_A
Sample
15
20
25
30
35
log2 LFQ

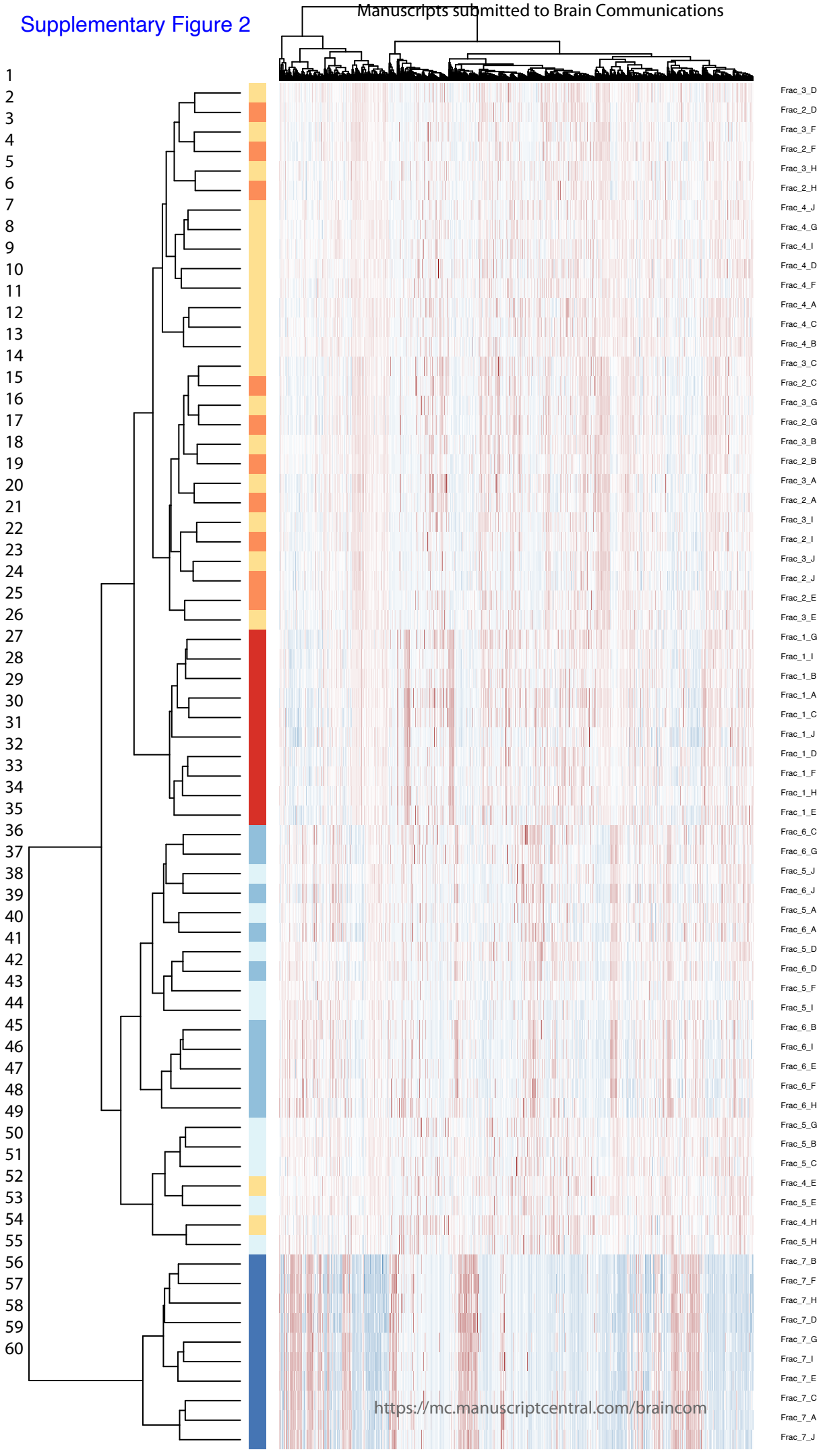
1
2
3
4
5
6
7
8
9
10
11
12
13
14
15
16
17
18
19
20
21
22
23
24
25
26
27
28
29
30
31
32
33
34
35
36
37
38
39
40
41
42
43
44
45
46
47
48
49
50
51
52
53
54
55
56
57
58
59
60
Frac_3_D
Frac_2_D
Frac_3_F
Frac_2_F
Frac_3_H
Frac_2_H
Frac_4_J
Frac_4_G
Frac_4_I
Frac_4_D
Frac_4_F
Frac_4_A
Frac_4_C
Frac_4_B
Frac_3_C
Frac_2_C
Frac_3_G
Frac_2_G
Frac_3_B
Frac_2_B
Frac_3_A
Frac_2_A
Frac_3_I
Frac_2_I
Frac_3_J
Frac_2_J
Frac_2_E
Frac_3_E
Frac_1_G
Frac_1_I
Frac_1_B
Frac_1_A
Frac_1_C
Frac_1_J
Frac_1_D
Frac_1_F
Frac_1_H
Frac_1_E
Frac_6_C
Frac_6_G
Frac_5_J
Frac_6_J
Frac_5_A
Frac_6_A
Frac_5_D
Frac_6_D
Frac_5_F
Frac_5_I
Frac_6_B
Frac_6_I
Frac_6_E
Frac_6_F
Frac_6_H
Frac_5_G
Frac_5_B
Frac_5_C
Frac_4_E
Frac_5_E
Frac_4_H
Frac_5_H
Frac_7_B
Frac_7_F
Frac_7_H
Frac_7_D
Frac_7_G
Frac_7_I
Frac_7_E
Frac_7_C
Frac_7_A
Frac_7_J

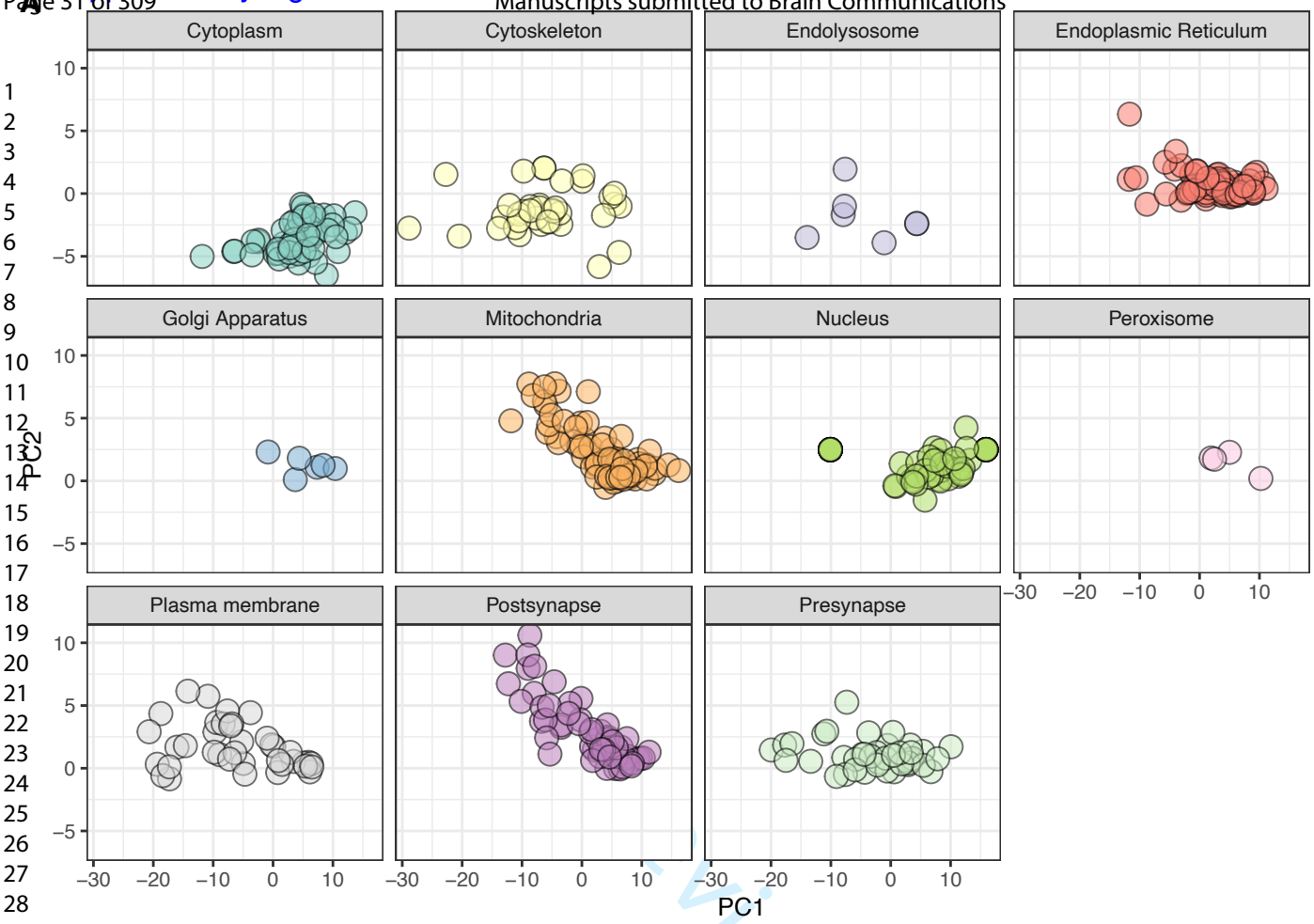

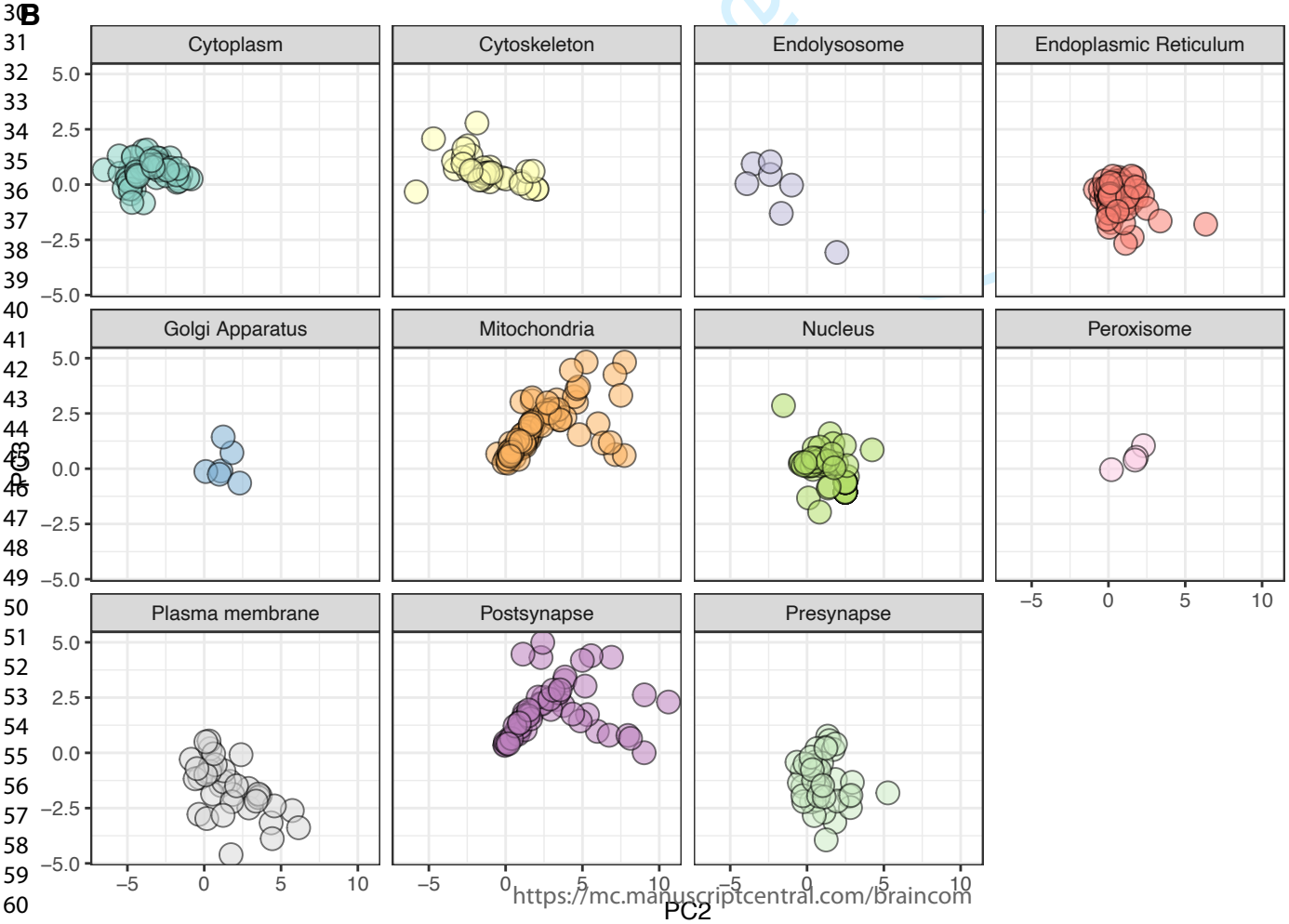

For Review Only

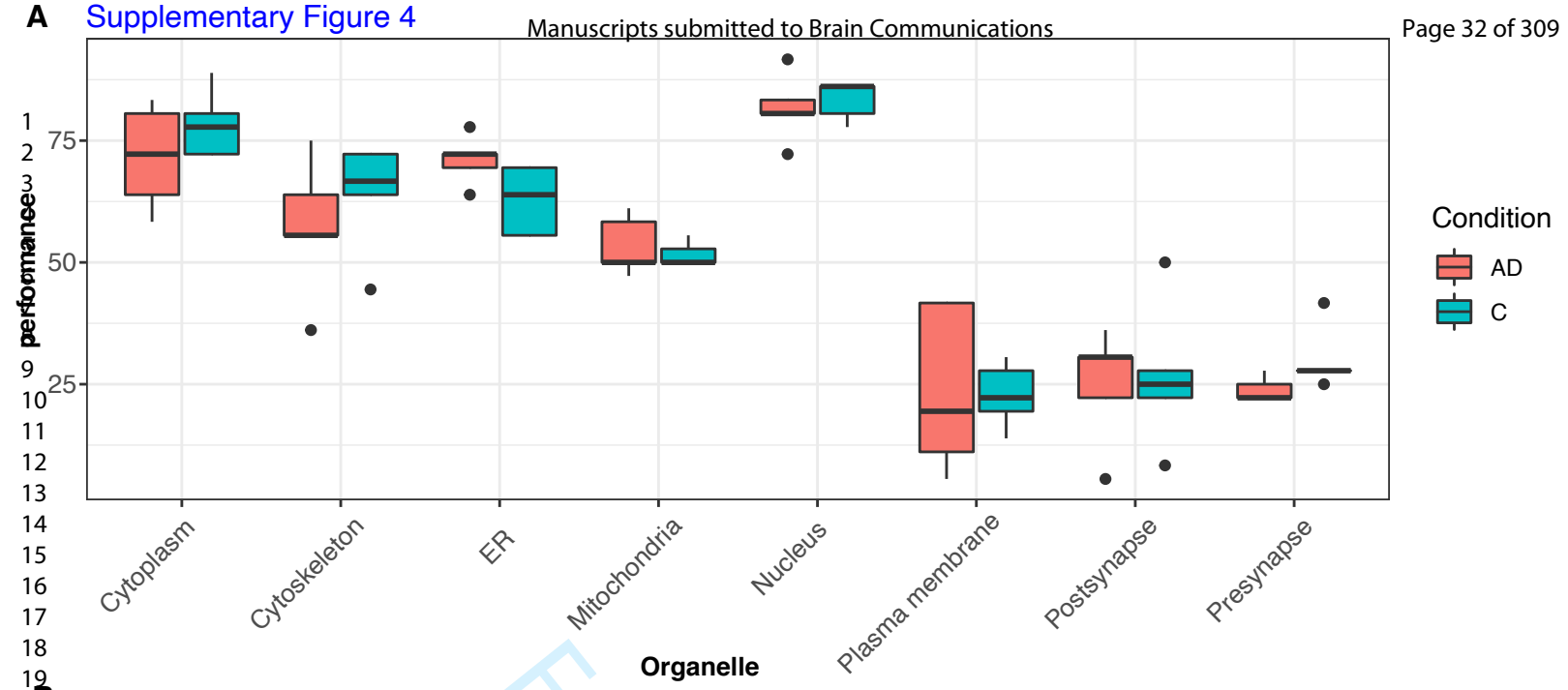

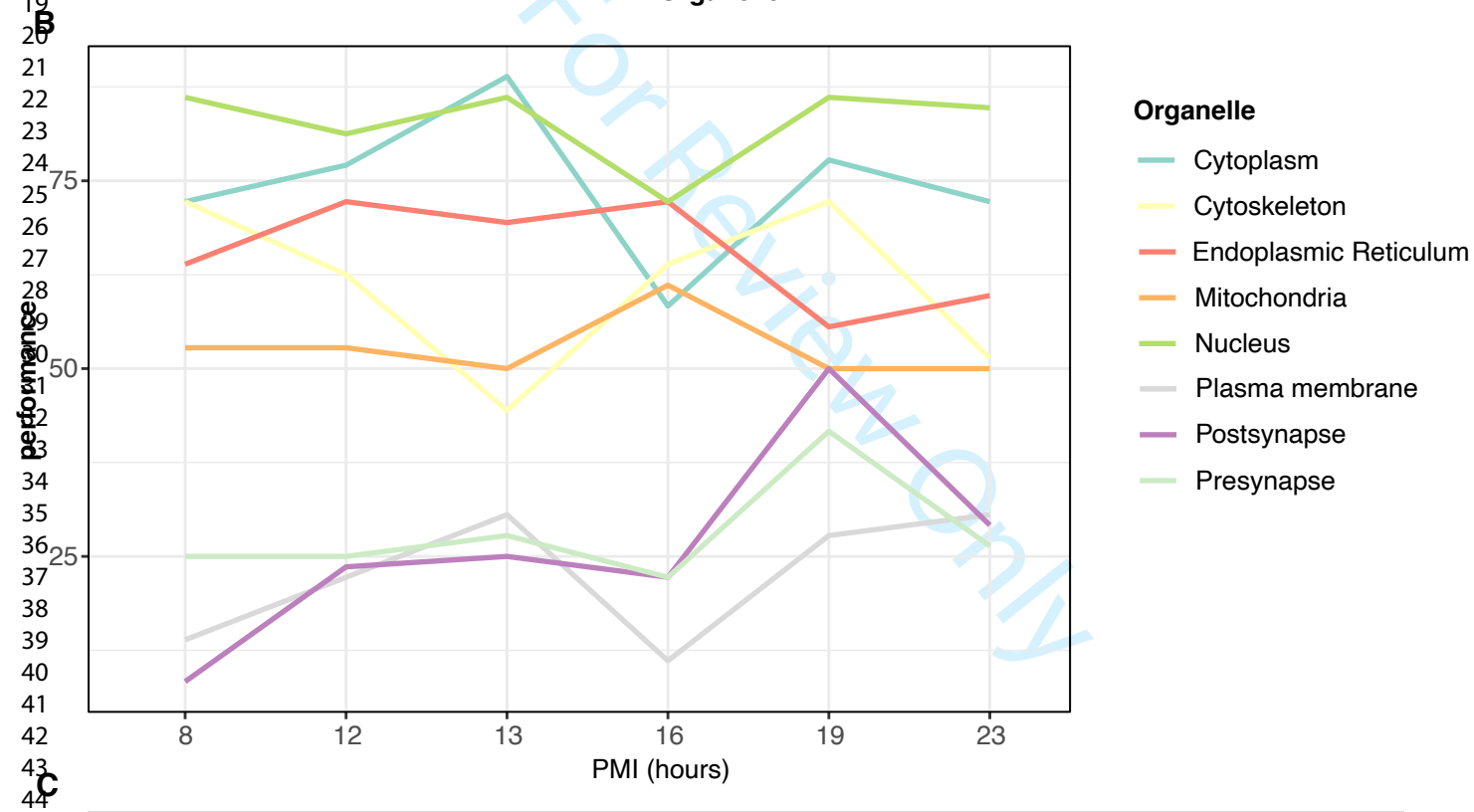

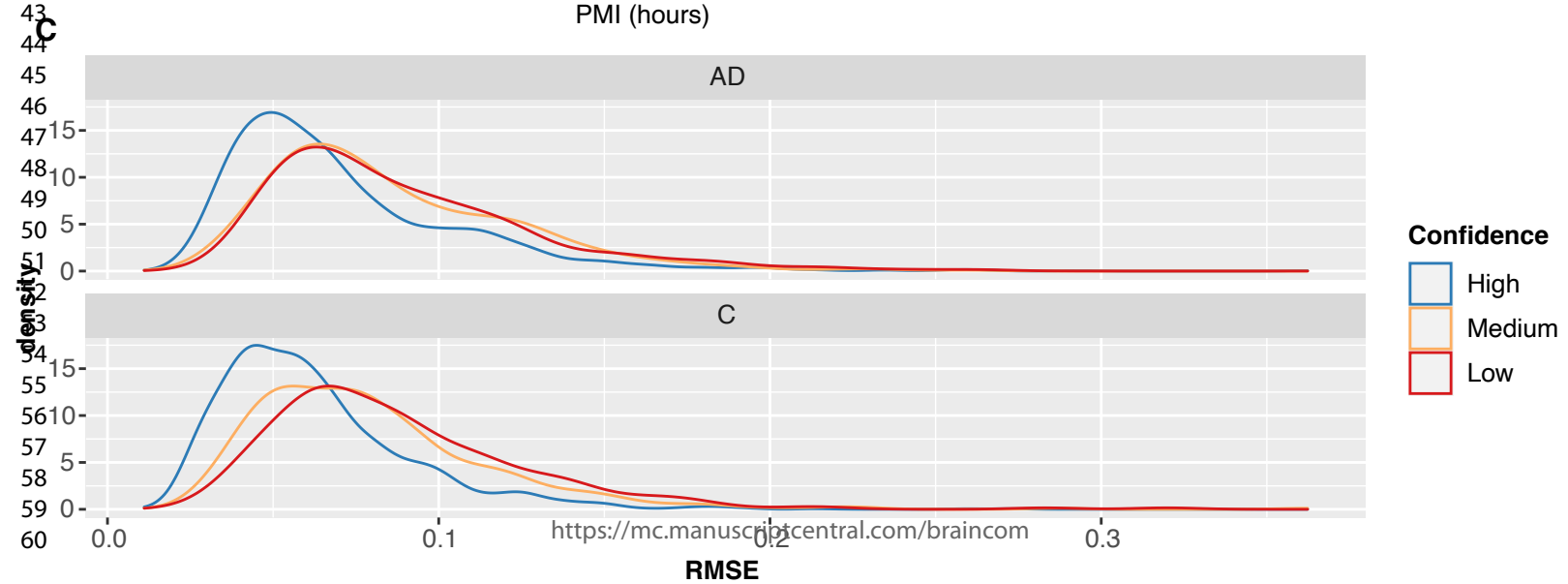

For Review Only

protein	Df1	Df2	F value1	Mean Sq1	Mean Sq2	Pr(>F)1	Sum Sq1	Sum Sq2
CON__P00	6	63	2.686373	9.6E+17	3.57E+17	########	6E+18	2E+19
CON__P02	6	63	########	8.14E+13	1.14E+14	########	4.88E+14	7.17E+15
CON__P13	6	63	1.355492	1.74E+14	1.28E+14	########	1.05E+15	8.1E+15
CON__Q3	6	63	11.66941	9.41E+13	8.07E+12	########	5.65E+14	5.08E+14
CON__Q3	6	63	6.268736	4.9E+15	7.82E+14	########	2.94E+16	4.93E+16
REV__sp|C	6	63	3.781005	3.56E+14	9.42E+13	########	2.14E+15	5.94E+15
REV__sp|C	6	63	8.039264	2.3E+14	2.86E+13	########	1.38E+15	1.8E+15
REV__sp|C	6	63	########	2.77E+14	3.05E+14	########	1.66E+15	2E+16
REV__sp|F	6	63	1.908449	1.1E+15	5.77E+14	########	7E+15	3.64E+16
REV__sp|F	6	63	7.906131	1.88E+15	2.38E+14	########	1.13E+16	1.5E+16
REV__sp|C	6	63	1.508392	1.53E+13	1.02E+13	########	9.21E+13	6.41E+14
REV__sp|C	6	63	9.204899	1.04E+17	1.13E+16	########	6E+17	########
REV__sp|C	6	63	27.06166	1.89E+15	6.98E+13	########	1.13E+16	4.4E+15
REV__sp|C	6	63	1.185598	6.63E+14	5.59E+14	########	3.98E+15	4E+16
REV__sp|C	6	63	3.840231	1.73E+14	4.5E+13	########	1.04E+15	2.83E+15
REV__sp|C	6	63	3.078344	1.03E+15	3.35E+14	########	6.19E+15	2E+16
REV__sp|C	6	63	1.888257	3.65E+15	1.93E+15	########	2.19E+16	1.22E+17
REV__sp|C	6	63	1.926356	1.62E+13	8.42E+12	########	9.73E+13	5.31E+14
REV__sp|C	6	63	10.44272	1.86E+15	1.78E+14	########	1.12E+16	1.12E+16
REV__sp|C	6	63	1.461871	1.65E+15	1.13E+15	########	9.89E+15	7.1E+16
REV__sp|C	6	63	1.865562	2.17E+15	1.16E+15	########	1.3E+16	7.33E+16
REV__sp|C	6	63	9.718062	4.98E+16	5.12E+15	########	2.99E+17	########
REV__sp|C	6	63	3.692137	4.03E+14	1.09E+14	########	2.42E+15	6.88E+15
REV__sp|C	6	63	########	7.21E+12	8.07E+12	########	4.33E+13	5.09E+14
REV__sp|C	6	63	1.37927	2.32E+14	1.68E+14	########	1.39E+15	1E+16
REV__sp|C	6	63	3.364018	3.11E+13	9.23E+12	########	1.86E+14	5.82E+14
REV__sp|C	6	63	1.599772	1.09E+15	6.82E+14	########	6.55E+15	4.3E+16
REV__sp|C	6	63	########	5.13E+13	1.12E+14	########	3.08E+14	7.06E+15
sp|A0A024	6	63	5.096095	9.67E+13	1.9E+13	########	5.8E+14	1.2E+15
sp|A0AVT1	6	63	8.745845	4.92E+14	5.63E+13	########	2.95E+15	3.55E+15
sp|A0FGR8	6	63	17.38213	5.79E+14	3.33E+13	########	3.48E+15	2E+15
sp|A0MZ66	6	63	8.023789	4.59E+14	5.73E+13	########	2.76E+15	3.61E+15
sp|A1L0T0	6	63	24.65864	4.49E+15	1.82E+14	########	2.7E+16	1E+16
sp|A2RRP1	6	63	5.362372	1.43E+14	2.66E+13	########	8.56E+14	1.68E+15
sp|A2RTX5	6	63	1.996084	2.72E+13	1.36E+13	########	1.63E+14	8.59E+14
sp|A2RU67	6	63	1.090889	7.35E+11	6.74E+11	########	4.41E+12	4.24E+13
sp|A5PKW4	6	63	3.188512	1.59E+12	4.99E+11	########	9.54E+12	3.14E+13
sp|A5YM72	6	63	########	6.49E+14	1.13E+15	########	4E+15	7E+16
sp|A6NDG6	6	63	74.4923	4.03E+15	5.4E+13	########	2.42E+16	3.4E+15
sp|A6NE02	6	63	2.521723	5.62E+14	2.23E+14	########	3.37E+15	1.4E+16
sp|A6NHX0	6	63	27.61477	3.42E+14	1.24E+13	########	2.05E+15	7.81E+14
sp|C9J069	6	63	10.40084	9.13E+13	8.78E+12	########	5.48E+14	5.53E+14
sp|E9PAV3	6	63	20.56538	1.54E+16	7.47E+14	########	9.22E+16	4.71E+16
sp|O00116	6	63	1.979113	8.92E+11	4.51E+11	########	5.35E+12	2.84E+13
sp|O00139	6	63	69.20765	3.3E+15	4.77E+13	########	1.98E+16	3E+15
sp|O00142	6	63	27.45619	1.88E+14	6.84E+12	########	1.13E+15	4.31E+14
sp|O00154	6	63	118.2107	2.44E+17	2.06E+15	########	1.46E+18	1.3E+17
sp|O00159	6	63	26.83147	1.52E+14	5.67E+12	########	9.12E+14	3.57E+14
sp|O00161	6	63	7.337935	3.25E+13	4.43E+12	########	1.95E+14	2.79E+14
sp|O00165	6	63	2.336932	1.16E+12	4.94E+11	########	6.93E+12	3.11E+13
sp|O00170	6	63	15.84295	2.64E+14	1.67E+13	########	1.58E+15	1.05E+15
sp|O00178	6	63	1.937646	2.56E+12	1.32E+12	########	1.54E+13	8.32E+13
sp|O00186	6	63	5.806891	9.27E+14	1.6E+14	########	5.56E+15	1.01E+16
sp|O00192	6	63	1.200105	4.25E+12	3.54E+12	########	2.55E+13	2.23E+14
sp|O00194	6	63	1.795242	5.79E+13	3.22E+13	########	3.47E+14	2.03E+15

For Review Only

sp|O00217|	6	63	76.5156	4.18E+16	5.47E+14	########	2.51E+17	3.44E+16
sp|O00231|	6	63	20.49569	8.93E+14	4.36E+13	########	5.36E+15	3E+15
sp|O00232|	6	63	21.88268	1.06E+15	4.86E+13	########	6.38E+15	3.06E+15
sp|O00244|	6	63	152.6637	1.99E+16	1.31E+14	########	1.2E+17	8.23E+15
sp|O00264|	6	63	27.04916	1.17E+16	4.33E+14	########	7.02E+16	2.72E+16
sp|O00291|	6	63	2.515698	2.93E+14	1.16E+14	########	1.76E+15	7.33E+15
sp|O00299|	6	63	5.855459	1.09E+15	1.87E+14	########	6.57E+15	1.18E+16
sp|O00303|	6	63	17.64659	2.05E+15	1.16E+14	########	1.23E+16	7.32E+15
sp|O00305|	6	63	6.542962	7.64E+13	1.17E+13	########	4.59E+14	7.36E+14
sp|O00330|	6	63	30.74803	7.68E+15	2.5E+14	########	4.61E+16	1.57E+16
sp|O00399|	6	63	########	2.36E+12	3.79E+12	########	1.42E+13	2.39E+14
sp|O00401|	6	63	10.14804	4.58E+14	4.52E+13	########	2.75E+15	2.85E+15
sp|O00408|	6	63	22.69093	1.26E+16	5.57E+14	########	7.58E+16	4E+16
sp|O00410|	6	63	9.16762	1.16E+16	1.27E+15	########	6.97E+16	7.99E+16
sp|O00429|	6	63	2.05858	3.38E+16	1.64E+16	########	2.03E+17	1.03E+18
sp|O00442|	6	63	34.26088	1.05E+15	3.06E+13	########	6.29E+15	1.93E+15
sp|O00445|	6	63	1.032677	5.96E+12	5.78E+12	########	3.58E+13	3.64E+14
sp|O00468|	6	63	15.24204	1.04E+15	6.83E+13	########	6.24E+15	4.3E+15
sp|O00471|	6	63	3.161701	2.07E+13	6.56E+12	########	1.24E+14	4.13E+14
sp|O00478|	6	63	3.873455	2.73E+13	7.04E+12	########	1.64E+14	4.44E+14
sp|O00483|	6	63	21.54485	9.25E+16	4.29E+15	########	5.55E+17	2.71E+17
sp|O00487|	6	63	18.5789	1.19E+15	6.4E+13	########	7.13E+15	4.03E+15
sp|O00499|	6	63	########	9.53E+15	2E+16	########	5.72E+16	1.26E+18
sp|O00505|	6	63	9.625813	9.15E+14	9.5E+13	########	5.49E+15	6E+15
sp|O00519|	6	63	12.91636	1.76E+14	1.36E+13	########	1.05E+15	8.58E+14
sp|O00533|	6	63	45.07453	1.56E+16	3.46E+14	########	9.35E+16	2.18E+16
sp|O00560|	6	63	26.68755	2.36E+15	8.86E+13	########	1.42E+16	5.58E+15
sp|O00567|	6	63	11.77228	7.22E+13	6.13E+12	########	4.33E+14	3.86E+14
sp|O00571|	6	63	38.79479	4.06E+15	1.05E+14	########	2.44E+16	7E+15
sp|O00584|	6	63	67.59755	1.91E+14	2.83E+12	########	1.15E+15	1.78E+14
sp|O00625|	6	63	7.213253	2.23E+14	3.09E+13	########	1.34E+15	2E+15
sp|O00629|	6	63	11.0787	1.53E+14	1.38E+13	########	9.19E+14	8.71E+14
sp|O00743|	6	63	2.420638	1.2E+13	4.95E+12	########	7.19E+13	3.12E+14
sp|O00764|	6	63	47.86533	2.68E+17	5.59E+15	########	1.61E+18	3.52E+17
sp|O14490|	6	63	17.20929	2.01E+14	1.17E+13	########	1.21E+15	7.37E+14
sp|O14495|	6	63	27.81479	1.21E+16	4.34E+14	########	7.24E+16	3E+16
sp|O14521|	6	63	10.08743	3.45E+13	3.42E+12	########	2.07E+14	2.16E+14
sp|O14523|	6	63	6.496893	7.12E+13	1.1E+13	########	4.27E+14	6.9E+14
sp|O14525|	6	63	8.942093	7.89E+13	8.82E+12	########	4.73E+14	5.56E+14
sp|O14531|	6	63	15.85224	7.42E+15	4.68E+14	########	4.45E+16	2.95E+16
sp|O14548|	6	63	35.56046	2.65E+16	7.46E+14	########	1.59E+17	4.7E+16
sp|O14558|	6	63	8.251788	7.27E+14	8.82E+13	########	4.36E+15	6E+15
sp|O14561|	6	63	76.42057	1E+17	1.31E+15	########	6E+17	8.25E+16
sp|O14576|	6	63	18.24587	3.9E+15	2.14E+14	########	2.34E+16	1.35E+16
sp|O14578|	6	63	23.3723	6.14E+14	2.63E+13	########	3.69E+15	1.66E+15
sp|O14579|	6	63	3.386695	3.02E+13	8.92E+12	########	1.81E+14	5.62E+14
sp|O14594|	6	63	24.9715	2E+17	8.01E+15	########	1.2E+18	5E+17
sp|O14617|	6	63	16.40104	1.73E+16	1.06E+15	########	1.04E+17	6.65E+16
sp|O14639|	6	63	9.121317	6.06E+14	6.64E+13	########	3.63E+15	4.18E+15
sp|O14653|	6	63	4.339854	3.68E+12	8.47E+11	########	2.21E+13	5.34E+13
sp|O14662|	6	63	########	1.62E+13	1.78E+13	########	9.73E+13	1.12E+15
sp|O14672|	6	63	21.49835	7.61E+15	3.54E+14	########	4.57E+16	2.23E+16
sp|O14734|	6	63	55.49447	2.4E+14	4.33E+12	########	1.44E+15	2.73E+14
sp|O14735|	6	63	11.76365	8.6E+13	7.31E+12	########	5.16E+14	4.61E+14
sp|O14737|	6	63	44.44237	2.07E+15	4.66E+13	########	1.24E+16	2.94E+15
sp|O14744|	6	63	5.442179	1.43E+13	2.63E+12	########	8.58E+13	1.66E+14

For Peer Review Only

sp|O14745|	6	63	45.40615	4.86E+16	1.07E+15	########	3E+17	7E+16
sp|O14773|	6	63	4.631057	5.38E+15	1.16E+15	########	3.23E+16	7E+16
sp|O14775|	6	63	4.016628	9.44E+14	2.35E+14	########	5.66E+15	1.48E+16
sp|O14787|	6	63	34.68094	1.96E+15	5.65E+13	########	1.18E+16	3.56E+15
sp|O14807|	6	63	9.996679	3.82E+14	3.82E+13	########	2.29E+15	2.4E+15
sp|O14810|	6	63	39.20709	9.81E+15	2.5E+14	########	5.88E+16	1.58E+16
sp|O14818|	6	63	75.10897	4.24E+16	5.65E+14	########	2.55E+17	3.56E+16
sp|O14827|	6	63	8.344226	1.07E+14	1.28E+13	########	6.39E+14	8.04E+14
sp|O14828|	6	63	6.602436	9.44E+13	1.43E+13	########	5.66E+14	9.01E+14
sp|O14841|	6	63	########	1.43E+14	1.82E+14	########	8.58E+14	1.15E+16
sp|O14874|	6	63	14.46185	5.38E+13	3.72E+12	########	3.23E+14	2.34E+14
sp|O14880|	6	63	24.59076	6E+17	2.43E+16	########	3.59E+18	1.53E+18
sp|O14907|	6	63	1.459328	1.78E+12	1.22E+12	########	1.07E+13	7.7E+13
sp|O14910|	6	63	9.151507	1.92E+14	2.1E+13	########	1.15E+15	1.32E+15
sp|O14936|	6	63	22.3271	6.34E+14	2.84E+13	########	3.8E+15	2E+15
sp|O14949|	6	63	45.73495	8.59E+15	1.88E+14	########	5.15E+16	1.18E+16
sp|O14950|	6	63	47.78294	1.91E+16	4E+14	########	1.15E+17	2.52E+16
sp|O14964|	6	63	1.083238	1.52E+14	1.41E+14	########	9.15E+14	9E+15
sp|O14974|	6	63	7.219401	2.36E+13	3.26E+12	########	1.41E+14	2.06E+14
sp|O14976|	6	63	14.21571	5.01E+14	3.53E+13	########	3.01E+15	2.22E+15
sp|O14979|	6	63	14.08541	1.49E+15	1.06E+14	########	8.92E+15	6.65E+15
sp|O14980|	6	63	67.16138	6.96E+15	1.04E+14	########	4.18E+16	6.53E+15
sp|O14994|	6	63	11.16272	8.32E+15	7.45E+14	########	4.99E+16	4.69E+16
sp|O15020|	6	63	41.4816	9.23E+16	2.23E+15	########	5.54E+17	1.4E+17
sp|O15027|	6	63	6.527031	5.29E+13	8.1E+12	########	3.17E+14	5.1E+14
sp|O15031|	6	63	3.160265	7.19E+13	2.27E+13	########	4.31E+14	1.43E+15
sp|O15034|	6	63	11.99571	2.17E+13	1.81E+12	########	1.3E+14	1.14E+14
sp|O15061|	6	63	3.700481	6.61E+13	1.79E+13	########	3.97E+14	1.13E+15
sp|O15066|	6	63	8.207795	3.67E+13	4.47E+12	########	2.2E+14	2.81E+14
sp|O15067|	6	63	96.85733	1.12E+16	1.16E+14	########	6.73E+16	7E+15
sp|O15068|	6	63	7.997638	3.97E+13	4.96E+12	########	2.38E+14	3.13E+14
sp|O15069|	6	63	9.099078	8.27E+13	9.09E+12	########	4.96E+14	5.73E+14
sp|O15075|	6	63	44.96297	3.65E+16	8.11E+14	########	########	5.11E+16
sp|O15079|	6	63	33.95758	6.02E+14	1.77E+13	########	3.61E+15	1.12E+15
sp|O15083|	6	63	34.77593	4.61E+14	1.32E+13	########	2.76E+15	8.34E+14
sp|O15118|	6	63	17.25355	2.14E+15	1.24E+14	########	1.28E+16	7.81E+15
sp|O15126|	6	63	3.102763	8.04E+17	########	########	5E+18	2E+19
sp|O15127|	6	63	3.610515	2.54E+13	7.05E+12	########	1.53E+14	4.44E+14
sp|O15144|	6	63	13.23818	1.97E+15	1.49E+14	########	1.18E+16	9E+15
sp|O15145|	6	63	6.010989	1.38E+15	2.3E+14	########	8.31E+15	1.45E+16
sp|O15173|	6	63	2.570035	1.07E+15	4.18E+14	########	6.45E+15	2.63E+16
sp|O15212|	6	63	19.74243	3.43E+14	1.74E+13	########	2.06E+15	1E+15
sp|O15228|	6	63	25.30134	6.21E+13	2.45E+12	########	3.72E+14	1.55E+14
sp|O15230|	6	63	17.9293	5.15E+14	2.87E+13	########	3.09E+15	1.81E+15
sp|O15240|	6	63	2.048669	1.47E+15	7.16E+14	########	8.8E+15	4.51E+16
sp|O15260|	6	63	11.70502	2.67E+14	2.28E+13	########	1.6E+15	1.44E+15
sp|O15294|	6	63	25.05926	4.06E+15	1.62E+14	########	2.44E+16	1E+16
sp|O15371|	6	63	10.62644	1.06E+15	9.95E+13	########	6.35E+15	6.27E+15
sp|O15372|	6	63	8.513476	3.95E+14	4.64E+13	########	2.37E+15	2.92E+15
sp|O15394|	6	63	39.18754	5.37E+16	1.37E+15	########	3.22E+17	8.64E+16
sp|O15400|	6	63	4.54807	2.65E+14	5.82E+13	########	1.59E+15	4E+15
sp|O15439|	6	63	2.101444	2.24E+13	1.07E+13	########	1.35E+14	6.73E+14
sp|O15484|	6	63	11.93244	8.37E+14	7.01E+13	########	5.02E+15	4.42E+15
sp|O15498|	6	63	5.600227	8.12E+13	1.45E+13	########	4.87E+14	9.14E+14
sp|O15523|	6	63	########	8.87E+10	1.01E+11	########	5.32E+11	6.35E+12
sp|O15530|	6	63	1.934613	6.83E+12	3.53E+12	########	4.1E+13	2.22E+14

For Peer Review Only

sp|O15540|	6	63	41.57341	5.98E+16	1.44E+15	########	3.59E+17	9.07E+16
sp|O43143|	6	63	25.48545	4.42E+14	1.73E+13	########	2.65E+15	1.09E+15
sp|O43149|	6	63	1.332068	3.61E+11	2.71E+11	########	2.17E+12	1.71E+13
sp|O43157|	6	63	6.232626	4.96E+14	7.96E+13	########	2.98E+15	5.01E+15
sp|O43169|	6	63	2.964636	3.95E+15	1.33E+15	########	2.37E+16	8.39E+16
sp|O43175|	6	63	3.834294	1.39E+17	3.63E+16	########	8.35E+17	2E+18
sp|O43181|	6	63	79.16971	1.3E+16	1.64E+14	########	7.79E+16	1.03E+16
sp|O43236|	6	63	24.47439	6.21E+16	2.54E+15	########	3.73E+17	1.6E+17
sp|O43237|	6	63	36.70817	3.2E+16	8.73E+14	########	########	5.5E+16
sp|O43242|	6	63	24.54479	2.93E+15	1.19E+14	########	1.76E+16	7.53E+15
sp|O43272|	6	63	14.03047	5.91E+14	4.21E+13	########	3.54E+15	3E+15
sp|O43295|	6	63	37.31025	5.05E+14	1.35E+13	########	3.03E+15	8.53E+14
sp|O43301|	6	63	91.45257	1.75E+18	1.91E+16	########	1E+19	1.2E+18
sp|O43324|	6	63	22.80612	3.85E+14	1.69E+13	########	2E+15	1.06E+15
sp|O43390|	6	63	62.12384	4.52E+16	7.27E+14	########	2.71E+17	4.58E+16
sp|O43396|	6	63	21.30457	7.68E+15	3.61E+14	########	4.61E+16	2.27E+16
sp|O43399|	6	63	22.62476	2.68E+15	1.19E+14	########	1.61E+16	7.47E+15
sp|O43414|	6	63	147.1762	3.31E+15	2.25E+13	########	1.98E+16	1E+15
sp|O43426|	6	63	13.96961	5.8E+16	4.15E+15	########	3.48E+17	2.62E+17
sp|O43427|	6	63	7.907285	2.13E+14	2.69E+13	########	1.28E+15	2E+15
sp|O43432|	6	63	9.4584	2.95E+13	3.12E+12	########	1.77E+14	1.97E+14
sp|O43464|	6	63	6.185229	3.55E+13	5.75E+12	########	2.13E+14	3.62E+14
sp|O43488|	6	63	59.54161	2.6E+16	4.36E+14	########	########	2.75E+16
sp|O43491|	6	63	5.526194	2.22E+16	4.02E+15	########	########	3E+17
sp|O43504|	6	63	17.64532	1.43E+15	8.08E+13	########	8.55E+15	5.09E+15
sp|O43505|	6	63	1.774298	6.52E+13	3.67E+13	########	3.91E+14	2.31E+15
sp|O43572|	6	63	1.708789	1.48E+12	8.65E+11	########	8.86E+12	5.45E+13
sp|O43581|	6	63	12.38426	2.62E+15	2.12E+14	########	1.57E+16	1.33E+16
sp|O43592|	6	63	3.124835	2.55E+13	8.15E+12	########	1.53E+14	5.13E+14
sp|O43598|	6	63	54.42568	8.02E+15	1.47E+14	########	4.81E+16	9.28E+15
sp|O43615|	6	63	54.80314	1.14E+15	2.09E+13	########	6.87E+15	1.32E+15
sp|O43674|	6	63	39.05346	4.15E+15	1.06E+14	########	2.49E+16	6.7E+15
sp|O43676|	6	63	22.55183	2.1E+15	9.29E+13	########	1.26E+16	5.85E+15
sp|O43678|	6	63	42.63937	1.3E+16	3.04E+14	########	7.79E+16	1.92E+16
sp|O43681|	6	63	7.945435	3.08E+15	3.87E+14	########	1.85E+16	2E+16
sp|O43687|	6	63	1.517971	9.44E+14	6.22E+14	########	5.66E+15	3.92E+16
sp|O43707|	6	63	84.83193	8.79E+16	1.04E+15	########	5E+17	6.53E+16
sp|O43708|	6	63	12.06113	2.13E+14	1.77E+13	########	1.28E+15	1.11E+15
sp|O43739|	6	63	1.633794	2.46E+12	1.51E+12	########	1.48E+13	9.5E+13
sp|O43747|	6	63	8.625967	3.21E+15	3.72E+14	########	1.93E+16	2.35E+16
sp|O43752|	6	63	2.288132	5.38E+12	2.35E+12	########	3.23E+13	1.48E+14
sp|O43759|	6	63	8.476525	1.04E+16	1.23E+15	########	6.24E+16	7.73E+16
sp|O43760|	6	63	1.419079	9.49E+11	6.69E+11	########	5.69E+12	4.21E+13
sp|O43761|	6	63	9.938349	1.13E+15	1.14E+14	########	6.81E+15	7.19E+15
sp|O43765|	6	63	2.117811	9.66E+13	4.56E+13	########	5.8E+14	2.87E+15
sp|O43768|	6	63	5.547366	4.67E+13	8.42E+12	########	2.8E+14	5.31E+14
sp|O43772|	6	63	3.903549	2.98E+13	7.64E+12	########	1.79E+14	4.82E+14
sp|O43776|	6	63	32.39511	1.4E+16	4.33E+14	########	8.42E+16	2.73E+16
sp|O43809|	6	63	23.38282	6.75E+14	2.89E+13	########	4.05E+15	1.82E+15
sp|O43813|	6	63	2.975524	3.77E+16	1.27E+16	########	2.26E+17	8E+17
sp|O43837|	6	63	51.50553	1.64E+17	3.18E+15	########	9.83E+17	2E+17
sp|O43852|	6	63	4.792496	2.85E+14	5.94E+13	########	1.71E+15	3.74E+15
sp|O43854|	6	63	########	9.65E+12	1.29E+13	########	5.79E+13	8.14E+14
sp|O43865|	6	63	58.90182	1.07E+17	1.81E+15	########	6.41E+17	1.14E+17
sp|O43920|	6	63	65.60881	3.45E+16	5.25E+14	########	2.07E+17	3.31E+16
sp|O60220|	6	63	18.80081	9.95E+14	5.29E+13	########	5.97E+15	3.34E+15

For Peer Review Only

sp|O60229|	6	63	7.881046	1.72E+13	2.18E+12	########	1.03E+14	1.37E+14
sp|O60245|	6	63	########	1.34E+12	1.51E+12	########	8.05E+12	9.5E+13
sp|O60256|	6	63	29.98876	8.85E+15	2.95E+14	########	5.31E+16	2E+16
sp|O60262|	6	63	30.87853	1.56E+16	5.05E+14	########	9.36E+16	3.18E+16
sp|O60268|	6	63	6.166473	1.87E+15	3.03E+14	########	1.12E+16	2E+16
sp|O60271|	6	63	2.976694	2.87E+13	9.65E+12	########	1.72E+14	6.08E+14
sp|O60282|	6	63	12.9859	4.45E+15	3.43E+14	########	2.67E+16	2.16E+16
sp|O60307|	6	63	2.935311	4.16E+12	1.42E+12	########	2.49E+13	8.92E+13
sp|O60313|	6	63	88.21909	1.73E+17	1.96E+15	########	1.04E+18	1.24E+17
sp|O60331|	6	63	19.07716	2.14E+15	1.12E+14	########	1.28E+16	7.07E+15
sp|O60346|	6	63	3.240613	2.64E+14	8.13E+13	########	1.58E+15	5.12E+15
sp|O60359|	6	63	4.20265	2.73E+13	6.49E+12	########	1.64E+14	4.09E+14
sp|O60493|	6	63	16.94866	1.64E+15	9.67E+13	########	9.83E+15	6.09E+15
sp|O60502|	6	63	2.674018	2.92E+14	1.09E+14	########	1.75E+15	7E+15
sp|O60503|	6	63	6.375878	1.19E+13	1.86E+12	########	7.11E+13	1.17E+14
sp|O60506|	6	63	17.78161	1.69E+15	9.51E+13	########	1.02E+16	5.99E+15
sp|O60613|	6	63	4.543487	3.29E+12	7.24E+11	########	1.97E+13	4.56E+13
sp|O60641|	6	63	33.67024	2.12E+17	6.31E+15	########	1.27E+18	3.97E+17
sp|O60645|	6	63	14.27243	2.14E+13	1.5E+12	########	1.28E+14	9.44E+13
sp|O60658|	6	63	2.019378	6.07E+12	3.01E+12	########	3.64E+13	1.89E+14
sp|O60664|	6	63	8.6929	2.17E+15	2.5E+14	########	1.3E+16	2E+16
sp|O60684|	6	63	104.2752	2.28E+15	2.19E+13	########	1.37E+16	1.38E+15
sp|O60716|	6	63	13.51013	1.59E+14	1.17E+13	########	9.52E+14	7.4E+14
sp|O60739|	6	63	4.606721	2.52E+13	5.48E+12	########	1.51E+14	3.45E+14
sp|O60741|	6	63	6.456639	4.91E+14	7.6E+13	########	2.94E+15	4.79E+15
sp|O60749|	6	63	########	7.88E+11	8.84E+11	########	4.73E+12	5.57E+13
sp|O60762|	6	63	6.922885	1.77E+14	2.56E+13	########	1.06E+15	1.61E+15
sp|O60763|	6	63	6.953477	4.38E+14	6.3E+13	########	2.63E+15	3.97E+15
sp|O60784|	6	63	2.794497	1.1E+15	3.93E+14	########	6.59E+15	2.47E+16
sp|O60825|	6	63	8.616487	3.21E+14	3.73E+13	########	1.93E+15	2E+15
sp|O60831|	6	63	37.98148	7.31E+14	1.93E+13	########	4.39E+15	1.21E+15
sp|O60841|	6	63	2.287427	5.51E+12	2.41E+12	########	3.31E+13	1.52E+14
sp|O60861|	6	63	12.48323	1.93E+15	1.55E+14	########	1.16E+16	9.74E+15
sp|O60869|	6	63	18.3805	5.09E+14	2.77E+13	########	3.05E+15	2E+15
sp|O60883|	6	63	65.31677	4.98E+15	7.63E+13	########	2.99E+16	4.8E+15
sp|O60884|	6	63	56.9156	5.16E+15	9.06E+13	########	3.1E+16	5.71E+15
sp|O60888|	6	63	47.37086	3.29E+16	6.94E+14	########	1.97E+17	4.37E+16
sp|O60925|	6	63	7.596602	1.46E+14	1.92E+13	########	8.76E+14	1E+15
sp|O60936|	6	63	17.13825	6.27E+13	3.66E+12	########	3.76E+14	2.3E+14
sp|O60939|	6	63	7.890526	1.31E+14	1.66E+13	########	7.84E+14	1.04E+15
sp|O75027|	6	63	3.479015	4.57E+12	1.31E+12	########	2.74E+13	8.28E+13
sp|O75044|	6	63	3.531772	4.59E+12	1.3E+12	########	2.76E+13	8.19E+13
sp|O75051|	6	63	4.083156	4.49E+13	1.1E+13	########	2.69E+14	6.93E+14
sp|O75061|	6	63	9.245905	1.97E+17	2.13E+16	########	1.18E+18	1E+18
sp|O75077|	6	63	14.44995	9.29E+15	6.43E+14	########	5.57E+16	4.05E+16
sp|O75078|	6	63	4.053628	1.44E+13	3.55E+12	########	8.63E+13	2.24E+14
sp|O75083|	6	63	15.76919	4.22E+16	2.68E+15	########	2.53E+17	2E+17
sp|O75110|	6	63	34.9868	7.46E+14	2.13E+13	########	4.48E+15	1.34E+15
sp|O75116|	6	63	1.139148	2.66E+14	2.33E+14	########	1.59E+15	1.47E+16
sp|O75122|	6	63	58.50415	6.35E+15	1.08E+14	########	3.81E+16	6.83E+15
sp|O75131|	6	63	19.08798	7.82E+14	4.09E+13	########	4.69E+15	3E+15
sp|O75145|	6	63	33.20594	1.56E+15	4.7E+13	########	9.36E+15	2.96E+15
sp|O75146|	6	63	7.882187	6.29E+14	7.98E+13	########	3.77E+15	5.03E+15
sp|O75155|	6	63	12.36201	2.41E+13	1.95E+12	########	1.45E+14	1.23E+14
sp|O75170|	6	63	1.743051	1.24E+13	7.14E+12	########	7.47E+13	4.5E+14
sp|O75208|	6	63	22.95711	4.62E+14	2.01E+13	########	2.77E+15	1E+15

For Peer Review Only

sp|O75223|	6	63	82.71499	6.02E+15	7.28E+13	########	3.61E+16	4.59E+15
sp|O75251|	6	63	44.0953	1.33E+16	3.02E+14	########	7.98E+16	2E+16
sp|O75306|	6	63	43.68141	########	4.8E+15	########	1.26E+18	3E+17
sp|O75323|	6	63	30.61882	1.41E+16	4.61E+14	########	8.48E+16	2.91E+16
sp|O75334|	6	63	7.069155	7.41E+12	1.05E+12	########	4.44E+13	6.6E+13
sp|O75340|	6	63	33.73139	8.24E+15	2.44E+14	########	4.95E+16	1.54E+16
sp|O75347|	6	63	172.8233	3.08E+16	1.78E+14	########	1.85E+17	1.12E+16
sp|O75348|	6	63	1.08393	2.57E+13	2.37E+13	########	1.54E+14	1.49E+15
sp|O75351|	6	63	3.66235	4.38E+13	1.2E+13	########	2.63E+14	7.53E+14
sp|O75352|	6	63	8.7657	7.15E+13	8.15E+12	########	4.29E+14	5.14E+14
sp|O75363|	6	63	11.9535	1.41E+16	1.18E+15	########	8.44E+16	7.41E+16
sp|O75367|	6	63	75.13015	1.07E+17	1.42E+15	########	6.42E+17	8.97E+16
sp|O75368|	6	63	111.0248	4.3E+16	3.87E+14	########	2.58E+17	2.44E+16
sp|O75369|	6	63	6.856267	9.28E+13	1.35E+13	########	5.57E+14	8.53E+14
sp|O75380|	6	63	12.4634	1.3E+16	1.04E+15	########	7.77E+16	6.55E+16
sp|O75381|	6	63	26.71043	5.82E+14	2.18E+13	########	3.49E+15	1.37E+15
sp|O75382|	6	63	29.15049	5.44E+14	1.87E+13	########	3.27E+15	1.18E+15
sp|O75390|	6	63	25.65133	1.07E+18	4.15E+16	########	6E+18	2.62E+18
sp|O75396|	6	63	38.86499	6.47E+15	1.66E+14	########	3.88E+16	1.05E+16
sp|O75431|	6	63	81.99532	1.27E+16	1.55E+14	########	7.61E+16	9.75E+15
sp|O75436|	6	63	3.562947	7.96E+13	2.23E+13	########	4.78E+14	1.41E+15
sp|O75438|	6	63	28.36213	3.89E+15	1.37E+14	########	2E+16	9E+15
sp|O75439|	6	63	7.776054	3.27E+13	4.2E+12	########	1.96E+14	2.65E+14
sp|O75475|	6	63	19.9325	6.79E+15	3.41E+14	########	4.07E+16	2.15E+16
sp|O75489|	6	63	85.49479	2.15E+17	2.51E+15	########	1.29E+18	########
sp|O75506|	6	63	37.79514	3.95E+15	1.04E+14	########	2.37E+16	6.58E+15
sp|O75508|	6	63	17.94135	3E+16	1.67E+15	########	1.8E+17	1.05E+17
sp|O75521|	6	63	2.210688	1.13E+13	5.1E+12	########	6.77E+13	3.22E+14
sp|O75531|	6	63	10.71999	3.68E+15	3.43E+14	########	2.21E+16	2.16E+16
sp|O75533|	6	63	122.2398	1.42E+15	1.16E+13	########	8.53E+15	7.33E+14
sp|O75534|	6	63	9.811036	2.35E+14	2.39E+13	########	1.41E+15	2E+15
sp|O75569|	6	63	12.47545	1.9E+15	1.52E+14	########	1.14E+16	9.58E+15
sp|O75592|	6	63	13.97957	5.13E+12	3.67E+11	########	3.08E+13	2.31E+13
sp|O75608|	6	63	14.20374	8.72E+14	6.14E+13	########	5.23E+15	3.87E+15
sp|O75643|	6	63	24.47027	5.9E+15	2.41E+14	########	3.54E+16	1.52E+16
sp|O75663|	6	63	20.00187	5.57E+14	2.78E+13	########	3.34E+15	1.75E+15
sp|O75689|	6	63	9.408706	3.42E+14	3.63E+13	########	2.05E+15	2.29E+15
sp|O75694|	6	63	5.688319	9.22E+12	1.62E+12	########	5.53E+13	1.02E+14
sp|O75746|	6	63	43.78394	2.26E+18	5.17E+16	########	1E+19	3E+18
sp|O75781|	6	63	19.51494	1.7E+16	8.72E+14	########	1.02E+17	5E+16
sp|O75821|	6	63	10.55716	3.33E+14	3.15E+13	########	2E+15	1.98E+15
sp|O75822|	6	63	25.60869	1.07E+15	4.16E+13	########	6.39E+15	2.62E+15
sp|O75828|	6	63	6.529271	1.28E+15	1.96E+14	########	7.69E+15	1.24E+16
sp|O75832|	6	63	3.177998	2.48E+13	7.8E+12	########	1.49E+14	4.92E+14
sp|O75844|	6	63	11.08329	4.2E+13	3.79E+12	########	2.52E+14	2.39E+14
sp|O75874|	6	63	57.31985	1.96E+16	3.42E+14	########	1.18E+17	2.15E+16
sp|O75879|	6	63	7.693007	1.62E+13	2.1E+12	########	9.69E+13	1.32E+14
sp|O75880|	6	63	16.05105	1.19E+14	7.42E+12	########	7.14E+14	4.67E+14
sp|O75884|	6	63	25.30348	3.6E+14	1.42E+13	########	2.16E+15	8.97E+14
sp|O75891|	6	63	4.091289	4.62E+16	1.13E+16	########	2.77E+17	7.11E+17
sp|O75896|	6	63	1.750109	2.06E+12	1.18E+12	########	1.24E+13	7.42E+13
sp|O75899|	6	63	4.868648	3.66E+14	7.52E+13	########	2.2E+15	4.74E+15
sp|O75914|	6	63	18.99015	2.55E+14	1.35E+13	########	1.53E+15	8.47E+14
sp|O75915|	6	63	40.16512	1.05E+16	2.62E+14	########	6.31E+16	1.65E+16
sp|O75923|	6	63	1.183991	7.13E+11	6.02E+11	########	4.28E+12	3.79E+13
sp|O75935|	6	63	4.319938	1.16E+14	2.67E+13	########	6.93E+14	2E+15

For Peer Review Only

sp|O75936|	6	63	########	6.38E+12	1.08E+13	########	3.83E+13	6.82E+14
sp|O75937|	6	63	20.3758	1.23E+14	6.05E+12	########	7.39E+14	3.81E+14
sp|O75947|	6	63	81.78384	1.14E+18	1.4E+16	########	7E+18	8.8E+17
sp|O75955|	6	63	22.82066	8.23E+16	3.61E+15	########	4.94E+17	2.27E+17
sp|O75962|	6	63	22.48827	3.87E+14	1.72E+13	########	2.32E+15	1.08E+15
sp|O75964|	6	63	45.77764	2.54E+16	5.55E+14	########	1.53E+17	3.5E+16
sp|O76003|	6	63	6.701973	1.46E+14	2.18E+13	########	8.76E+14	1.37E+15
sp|O76024|	6	63	8.261385	1.05E+15	1.27E+14	########	6.31E+15	8.02E+15
sp|O76041|	6	63	7.563126	3.45E+14	4.56E+13	########	2.07E+15	3E+15
sp|O76054|	6	63	########	3.9E+14	4.23E+14	########	2.34E+15	3E+16
sp|O76070|	6	63	59.20532	4.42E+17	7.46E+15	########	3E+18	5E+17
sp|O76094|	6	63	13.69732	2.09E+14	1.52E+13	########	1.25E+15	9.59E+14
sp|O94760|	6	63	246.6985	8E+18	3.13E+16	########	5E+19	2E+18
sp|O94772|	6	63	6.578659	3.24E+15	4.92E+14	########	2E+16	3E+16
sp|O94805|	6	63	2.141265	1.04E+13	4.84E+12	########	6.22E+13	3.05E+14
sp|O94811|	6	63	14.81406	3.77E+17	2.54E+16	########	2.26E+18	1.6E+18
sp|O94819|	6	63	17.94953	1.29E+16	7.16E+14	########	7.71E+16	4.51E+16
sp|O94826|	6	63	69.80384	1.25E+17	1.79E+15	########	7.51E+17	1.13E+17
sp|O94830|	6	63	10.25383	2.55E+13	2.49E+12	########	1.53E+14	1.57E+14
sp|O94832|	6	63	5.277186	2.3E+16	4.37E+15	########	1.38E+17	########
sp|O94856|	6	63	22.25161	4.44E+17	2E+16	########	3E+18	1.26E+18
sp|O94874|	6	63	6.42732	4.01E+14	6.25E+13	########	2.41E+15	3.94E+15
sp|O94875|	6	63	4.182399	3.51E+13	8.38E+12	########	2.1E+14	5.28E+14
sp|O94903|	6	63	9.070683	6.67E+14	7.35E+13	########	4E+15	4.63E+15
sp|O94905|	6	63	23.52317	9E+15	3.86E+14	########	5E+16	2.43E+16
sp|O94910|	6	63	5.970981	2.1E+15	3.51E+14	########	1.26E+16	2.21E+16
sp|O94911|	6	63	1.941882	1.76E+13	9.06E+12	########	1.06E+14	5.71E+14
sp|O94915|	6	63	8.104536	2.34E+15	2.88E+14	########	1.4E+16	2E+16
sp|O94919|	6	63	15.50653	4E+16	2.58E+15	########	2.4E+17	1.63E+17
sp|O94925|	6	63	117.7417	2E+18	1.6E+16	########	1E+19	1.01E+18
sp|O94966|	6	63	1.373104	2.73E+11	1.99E+11	########	1.64E+12	1.25E+13
sp|O94967|	6	63	37.83969	3.24E+15	8.57E+13	########	1.95E+16	5E+15
sp|O94973|	6	63	20.93109	5.6E+17	2.67E+16	########	3E+18	1.68E+18
sp|O94979|	6	63	16.6365	5.21E+14	3.13E+13	########	3.12E+15	1.97E+15
sp|O94985|	6	63	7.336547	5.5E+14	7.49E+13	########	3.3E+15	5E+15
sp|O95057|	6	63	10.84351	3.65E+14	3.37E+13	########	2.19E+15	2.12E+15
sp|O95139|	6	63	45.85898	7.03E+15	1.53E+14	########	4.22E+16	9.66E+15
sp|O95140|	6	63	33.10324	3.49E+15	1.05E+14	########	2.09E+16	6.64E+15
sp|O95167|	6	63	16.58451	4.31E+15	2.6E+14	########	2.59E+16	2E+16
sp|O95168|	6	63	51.43539	1.39E+16	2.7E+14	########	8.35E+16	1.7E+16
sp|O95169|	6	63	7.538196	2.23E+15	2.95E+14	########	1.34E+16	2E+16
sp|O95182|	6	63	38.7932	8.36E+15	2.16E+14	########	5.02E+16	1.36E+16
sp|O95196|	6	63	4.724023	6.47E+13	1.37E+13	########	3.88E+14	8.63E+14
sp|O95197|	6	63	9.980583	2.6E+16	2.61E+15	########	1.56E+17	1.64E+17
sp|O95202|	6	63	51.95792	6.32E+16	1.22E+15	########	3.79E+17	7.67E+16
sp|O95219|	6	63	18.69408	3E+14	1.6E+13	########	1.8E+15	1.01E+15
sp|O95248|	6	63	25.59317	1.42E+16	5.56E+14	########	8.54E+16	3.51E+16
sp|O95278|	6	63	14.85067	2.96E+14	1.99E+13	########	1.78E+15	1.26E+15
sp|O95292|	6	63	1.575933	2.98E+14	1.89E+14	########	1.79E+15	1.19E+16
sp|O95294|	6	63	3.60507	3.1E+13	8.59E+12	########	1.86E+14	5.41E+14
sp|O95295|	6	63	########	8.16E+12	8.37E+12	########	4.9E+13	5.27E+14
sp|O95298|	6	63	18.67627	3.07E+15	1.64E+14	########	1.84E+16	1.03E+16
sp|O95299|	6	63	74.08472	1.67E+17	2.25E+15	########	1E+18	########
sp|O95319|	6	63	1.672432	5.76E+13	3.44E+13	########	3.46E+14	2.17E+15
sp|O95336|	6	63	97.66985	1.05E+17	1.08E+15	########	6E+17	6.79E+16
sp|O95352|	6	63	30.86029	1.69E+15	5.48E+13	########	1.01E+16	3.45E+15

For Peer Review Only

sp|O95372|	6	63	10.70144	4.72E+14	4.41E+13	########	2.83E+15	2.78E+15
sp|O95373|	6	63	3.863039	4.8E+14	1.24E+14	########	2.88E+15	7.83E+15
sp|O95394|	6	63	27.3757	3.54E+15	1.29E+14	########	2.13E+16	8.16E+15
sp|O95433|	6	63	5.594884	3.08E+15	5.5E+14	########	1.85E+16	3.47E+16
sp|O95487|	6	63	14.74547	7.15E+13	4.85E+12	########	4.29E+14	3.05E+14
sp|O95502|	6	63	1.018225	1.2E+14	1.18E+14	########	7.21E+14	7E+15
sp|O95563|	6	63	23.8743	1.63E+15	6.81E+13	########	9.75E+15	4.29E+15
sp|O95571|	6	63	5.939067	7.74E+13	1.3E+13	########	4.65E+14	8.21E+14
sp|O95573|	6	63	16.35639	1.72E+15	1.05E+14	########	1.03E+16	6.64E+15
sp|O95670|	6	63	3.196337	4.02E+16	1.26E+16	########	2.41E+17	7.92E+17
sp|O95671|	6	63	########	2.7E+13	5.3E+13	########	1.62E+14	3.34E+15
sp|O95674|	6	63	41.53562	4.79E+15	1.15E+14	########	2.87E+16	7.26E+15
sp|O95716|	6	63	########	2.22E+14	2.32E+14	########	1.33E+15	1.46E+16
sp|O95741|	6	63	2.840327	2.13E+15	7.51E+14	########	1.28E+16	5E+16
sp|O95747|	6	63	11.39507	2.28E+14	2E+13	########	1.37E+15	1.26E+15
sp|O95757|	6	63	2.487656	6.27E+15	2.52E+15	########	3.76E+16	1.59E+17
sp|O95777|	6	63	39.43415	2.66E+14	6.76E+12	########	1.6E+15	4.26E+14
sp|O95782|	6	63	19.12149	2.15E+18	1.12E+17	########	1E+19	7E+18
sp|O95810|	6	63	6.592909	2.02E+13	3.06E+12	########	1.21E+14	1.93E+14
sp|O95817|	6	63	5.025581	1.13E+15	2.25E+14	########	6.77E+15	1.41E+16
sp|O95822|	6	63	5.713549	2.29E+14	4.01E+13	########	1.37E+15	2.53E+15
sp|O95825|	6	63	6.924866	2.27E+14	3.27E+13	########	1.36E+15	2E+15
sp|O95831|	6	63	35.89009	9.04E+15	2.52E+14	########	5.43E+16	1.59E+16
sp|O95837|	6	63	5.724271	3.97E+13	6.93E+12	########	2.38E+14	4.36E+14
sp|O95847|	6	63	51.50173	6.42E+14	1.25E+13	########	3.85E+15	7.85E+14
sp|O95858|	6	63	4.219417	4.87E+14	1.15E+14	########	2.92E+15	7.27E+15
sp|O95861|	6	63	98.5371	1.78E+15	1.81E+13	########	1.07E+16	1.14E+15
sp|O95865|	6	63	36.81742	1.36E+16	3.7E+14	########	8.16E+16	2.33E+16
sp|O95881|	6	63	5.257922	2.66E+13	5.06E+12	########	1.6E+14	3.19E+14
sp|O95886|	6	63	5.940082	6.28E+12	1.06E+12	########	3.77E+13	6.66E+13
sp|O95970|	6	63	18.82949	6.11E+15	3.25E+14	########	3.67E+16	2E+16
sp|O95989|	6	63	164.9124	1.42E+16	8.59E+13	########	8.5E+16	5.41E+15
sp|O96000|	6	63	52.27681	5.34E+16	1.02E+15	########	3.2E+17	6.44E+16
sp|O96005|	6	63	4.846608	4.06E+13	8.38E+12	########	2.44E+14	5.28E+14
sp|O96008|	6	63	14.57237	2.22E+14	1.53E+13	########	1.33E+15	9.62E+14
sp|P00338|	6	63	250.0391	1E+19	4.49E+16	########	7E+19	2.83E+18
sp|P00352|	6	63	100.1391	2.52E+17	2.52E+15	########	1.51E+18	2E+17
sp|P00367|	6	63	34.84856	3.81E+18	1.09E+17	########	2E+19	7E+18
sp|P00387|	6	63	14.27911	3.93E+16	2.76E+15	########	2.36E+17	1.74E+17
sp|P00390|	6	63	30.47845	4.56E+15	1.5E+14	########	2.73E+16	9.42E+15
sp|P00395|	6	63	14.19557	2.8E+16	1.97E+15	########	1.68E+17	1.24E+17
sp|P00403|	6	63	43.04241	########	4.44E+16	########	1E+19	2.8E+18
sp|P00441|	6	63	120.2456	2E+19	1.26E+17	########	9E+19	8E+18
sp|P00450|	6	63	8.175624	2.06E+16	2.52E+15	########	1.24E+17	1.59E+17
sp|P00488|	6	63	2.670345	3.1E+13	1.16E+13	########	1.86E+14	7.32E+14
sp|P00491|	6	63	32.74867	2.1E+16	6.41E+14	########	1.26E+17	4.04E+16
sp|P00492|	6	63	175.1507	1.21E+18	6.91E+15	########	7E+18	4.35E+17
sp|P00505|	6	63	33.34503	3.38E+18	1.01E+17	########	2E+19	6E+18
sp|P00533|	6	63	5.910065	6.39E+13	1.08E+13	########	3.84E+14	6.82E+14
sp|P00558|	6	63	341.986	9E+19	2.6E+17	########	5E+20	2E+19
sp|P00568|	6	63	########	2.64E+15	1.19E+16	########	1.59E+16	7.48E+17
sp|P00738|	6	63	16.15125	1.95E+18	1.21E+17	########	1E+19	8E+18
sp|P00751|	6	63	7.24961	4.46E+14	6.15E+13	########	2.67E+15	3.87E+15
sp|P00813|	6	63	21.6778	1.34E+14	6.19E+12	########	8.05E+14	3.9E+14
sp|P00846|	6	63	1.81579	2.33E+14	1.28E+14	########	1.4E+15	8E+15
sp|P00915|	6	63	40.99982	7.09E+17	1.73E+16	########	4.25E+18	1E+18

For Peer Review Only

sp|P00918|	6	63	75.86556	1.16E+18	1.53E+16	########	7E+18	1E+18
sp|P00966|	6	63	7.9144	5.34E+15	6.75E+14	########	3.21E+16	4E+16
sp|P01008|	6	63	13.19342	5.15E+13	3.9E+12	########	3.09E+14	2.46E+14
sp|P01009|	6	63	12.06209	1.17E+18	9.74E+16	########	7E+18	6E+18
sp|P01011|	6	63	4.70907	2.24E+16	4.76E+15	########	1.35E+17	########
sp|P01019|	6	63	10.16829	8.33E+15	8.2E+14	########	5E+16	5.16E+16
sp|P01023|	6	63	17.09292	8.28E+17	4.84E+16	########	5E+18	3.05E+18
sp|P01024|	6	63	12.27699	5.3E+17	4.32E+16	########	3.18E+18	3E+18
sp|P01034|	6	63	7.650914	3.85E+15	5.03E+14	########	2.31E+16	3.17E+16
sp|P01112|	6	63	21.85354	3.73E+16	1.71E+15	########	2.24E+17	1.08E+17
sp|P01116|	6	63	21.06483	1.82E+15	8.63E+13	########	1.09E+16	5.43E+15
sp|P01303|	6	63	1.761412	1.1E+13	6.26E+12	########	6.61E+13	3.94E+14
sp|P01591|	6	63	3.709395	7.48E+12	2.02E+12	########	4.49E+13	1.27E+14
sp|P01834|	6	63	8.500652	2.05E+17	2.41E+16	########	1.23E+18	1.52E+18
sp|P01857|	6	63	7.226232	1.51E+18	2.08E+17	########	9E+18	1E+19
sp|P01859|	6	63	18.39642	5.5E+16	2.99E+15	########	3.3E+17	1.88E+17
sp|P01860|	6	63	6.229324	5.04E+15	8.1E+14	########	3.03E+16	5.1E+16
sp|P01861|	6	63	3.369083	1.62E+16	4.8E+15	########	9.7E+16	3E+17
sp|P01871|	6	63	4.104823	1.04E+16	2.53E+15	########	6.24E+16	1.6E+17
sp|P01876|	6	63	5.946563	3.56E+17	5.99E+16	########	2.14E+18	3.77E+18
sp|P01903|	6	63	3.764173	1.13E+14	2.99E+13	########	6.75E+14	1.88E+15
sp|P02042|	6	63	27.88049	3E+18	1.08E+17	########	2E+19	7E+18
sp|P02462|	6	63	34.37265	2.61E+15	7.59E+13	########	1.56E+16	5E+15
sp|P02511|	6	63	12.57946	4E+18	3.21E+17	########	2E+19	2E+19
sp|P02545|	6	63	58.78544	3.85E+17	6.55E+15	########	2E+18	4E+17
sp|P02549|	6	63	6.175148	3.47E+13	5.61E+12	########	2.08E+14	3.54E+14
sp|P02647|	6	63	14.71642	4.82E+16	3.27E+15	########	2.89E+17	2.06E+17
sp|P02649|	6	63	3.34237	4.51E+16	1.35E+16	########	2.7E+17	8.49E+17
sp|P02652|	6	63	7.498019	3.48E+14	4.64E+13	########	2.09E+15	2.92E+15
sp|P02656|	6	63	8.16975	1.27E+14	1.55E+13	########	7.62E+14	9.79E+14
sp|P02671|	6	63	7.040314	1.28E+17	1.82E+16	########	7.67E+17	1.14E+18
sp|P02675|	6	63	7.487813	4.63E+17	6.19E+16	########	2.78E+18	3.9E+18
sp|P02679|	6	63	8.291226	2.47E+17	2.97E+16	########	1.48E+18	1.87E+18
sp|P02686|	6	63	13.93861	3E+20	2E+19	########	2E+21	1E+21
sp|P02689|	6	63	4.053368	4.52E+16	1E+16	########	2.71E+17	7E+17
sp|P02730|	6	63	3.664269	4.58E+15	1.25E+15	########	2.75E+16	7.87E+16
sp|P02745|	6	63	4.037494	1.27E+14	3.14E+13	########	7.61E+14	2E+15
sp|P02746|	6	63	8.633647	1.83E+15	2.12E+14	########	1.1E+16	1.34E+16
sp|P02747|	6	63	4.467009	1.93E+15	4.33E+14	########	1.16E+16	2.73E+16
sp|P02749|	6	63	########	1.35E+14	1.53E+14	########	8.09E+14	1E+16
sp|P02750|	6	63	4.148942	4.88E+14	1.18E+14	########	2.93E+15	7.41E+15
sp|P02763|	6	63	9.492604	9.88E+16	1.04E+16	########	5.93E+17	6.56E+17
sp|P02765|	6	63	4.850865	3.47E+13	7.15E+12	########	2.08E+14	4.5E+14
sp|P02766|	6	63	48.98775	1.73E+15	3.53E+13	########	1.04E+16	2.23E+15
sp|P02768|	6	63	35.1496	2E+20	6E+18	########	1E+21	4E+20
sp|P02774|	6	63	8.140029	1.97E+15	2.42E+14	########	1.18E+16	1.53E+16
sp|P02786|	6	63	21.17231	4.78E+13	2.26E+12	########	2.87E+14	1.42E+14
sp|P02787|	6	63	58.10348	5.19E+17	8.93E+15	########	3E+18	5.63E+17
sp|P02790|	6	63	14.2468	6.32E+16	4.43E+15	########	3.79E+17	2.79E+17
sp|P02792|	6	63	24.89603	3.5E+17	1.41E+16	########	2E+18	8.86E+17
sp|P02794|	6	63	35.68356	1.7E+18	4.77E+16	########	1E+19	3E+18
sp|P03886|	6	63	20.38831	3.19E+15	1.56E+14	########	1.91E+16	9.85E+15
sp|P03891|	6	63	22.72153	9.19E+15	4.05E+14	########	5.52E+16	2.55E+16
sp|P03905|	6	63	20.96122	7.02E+15	3.35E+14	########	4.21E+16	2.11E+16
sp|P03915|	6	63	44.91272	9.7E+15	2.16E+14	########	5.82E+16	1.36E+16
sp|P03928|	6	63	7.343841	1.74E+14	2.37E+13	########	1.04E+15	1.49E+15

For Peer Review Only

sp|P04003|	6	63	5.914357	9.18E+14	1.55E+14	########	5.51E+15	9.78E+15
sp|P04040|	6	63	28.09844	3.42E+16	1.22E+15	########	2.05E+17	8E+16
sp|P04062|	6	63	13.57618	1.12E+14	8.24E+12	########	6.71E+14	5.19E+14
sp|P04075|	6	63	79.08309	4E+19	5.54E+17	########	3E+20	3E+19
sp|P04080|	6	63	55.42331	2.11E+16	3.81E+14	########	1.27E+17	2.4E+16
sp|P04083|	6	63	2.299962	4.61E+16	2E+16	########	2.77E+17	1.26E+18
sp|P04114|	6	63	3.055801	6.55E+16	2.14E+16	########	3.93E+17	1.35E+18
sp|P04156|	6	63	11.11034	5.76E+15	5.19E+14	########	3.46E+16	3.27E+16
sp|P04179|	6	63	13.25686	2.67E+17	2.02E+16	########	1.6E+18	1.27E+18
sp|P04181|	6	63	40.83539	4.33E+15	1.06E+14	########	2.6E+16	6.68E+15
sp|P04216|	6	63	13.74266	1.2E+18	8.7E+16	########	7E+18	5E+18
sp|P04217|	6	63	2.781472	4.07E+13	1.46E+13	########	2.44E+14	9.22E+14
sp|P04264|	6	63	3.751601	1.45E+16	3.85E+15	########	8.68E+16	2.43E+17
sp|P04271|	6	63	13.91403	9E+18	6E+17	########	5E+19	4E+19
sp|P04350|	6	63	72.11001	2E+19	2.89E+17	########	1E+20	2E+19
sp|P04406|	6	63	77.17607	2E+20	3.16E+18	########	1E+21	2E+20
sp|P04632|	6	63	27.82925	8.84E+15	3.18E+14	########	5.3E+16	2E+16
sp|P04792|	6	63	13.99917	1.59E+17	1.13E+16	########	9.52E+17	7.14E+17
sp|P04839|	6	63	6.088417	9.32E+14	1.53E+14	########	5.59E+15	9.65E+15
sp|P04843|	6	63	44.83578	1.23E+16	2.74E+14	########	7.36E+16	1.72E+16
sp|P04844|	6	63	41.39692	5.25E+15	1.27E+14	########	3.15E+16	7.99E+15
sp|P04899|	6	63	62.99431	2E+17	2.96E+15	########	########	1.86E+17
sp|P05023|	6	63	29.12438	1E+19	4.1E+17	########	7E+19	3E+19
sp|P05026|	6	63	23.54291	2E+19	9.16E+17	########	1E+20	6E+19
sp|P05060|	6	63	3.748819	1.98E+14	5.27E+13	########	1.19E+15	3.32E+15
sp|P05067|	6	63	########	2.28E+17	2.9E+17	########	1E+18	2E+19
sp|P05090|	6	63	26.73156	2.24E+16	8.37E+14	########	1.34E+17	5.28E+16
sp|P05091|	6	63	108.3971	1.15E+18	1.06E+16	########	7E+18	6.68E+17
sp|P05107|	6	63	8.470053	3.6E+14	4.25E+13	########	2.16E+15	2.68E+15
sp|P05109|	6	63	1.427009	7.91E+14	5.54E+14	########	4.75E+15	3.49E+16
sp|P05129|	6	63	6.722712	9.6E+15	1.43E+15	########	5.76E+16	8.99E+16
sp|P05141|	6	63	28.15785	6.3E+16	2.24E+15	########	3.78E+17	1.41E+17
sp|P05155|	6	63	2.919584	3.34E+14	1.14E+14	########	2E+15	7.21E+15
sp|P05164|	6	63	########	4.74E+13	7.25E+13	########	2.84E+14	4.57E+15
sp|P05165|	6	63	23.2367	4.61E+15	1.98E+14	########	2.76E+16	1.25E+16
sp|P05166|	6	63	37.69298	9.65E+15	2.56E+14	########	5.79E+16	1.61E+16
sp|P05198|	6	63	30.73674	1.79E+15	5.82E+13	########	1.07E+16	3.67E+15
sp|P05230|	6	63	15.68516	1.27E+15	8.11E+13	########	7.63E+15	5.11E+15
sp|P05362|	6	63	1.727157	2.06E+13	1.2E+13	########	1.24E+14	7.53E+14
sp|P05386|	6	63	49.11063	4.12E+17	8.39E+15	########	2E+18	5.29E+17
sp|P05387|	6	63	48.03471	3.82E+17	7.96E+15	########	2.29E+18	5.01E+17
sp|P05388|	6	63	44.12412	########	8.2E+15	########	2E+18	########
sp|P05413|	6	63	104.1628	2.3E+17	2.21E+15	########	1.38E+18	1.39E+17
sp|P05452|	6	63	1.733561	2.28E+12	1.32E+12	########	1.37E+13	8.3E+13
sp|P05455|	6	63	9.013417	2.93E+15	3.25E+14	########	1.76E+16	2E+16
sp|P05556|	6	63	25.25675	1.67E+16	6.61E+14	########	1E+17	4.16E+16
sp|P05771|	6	63	44.65682	1.33E+17	2.98E+15	########	7.98E+17	1.88E+17
sp|P05937|	6	63	31.51229	1.71E+14	5.41E+12	########	1.02E+15	3.41E+14
sp|P06132|	6	63	58.45095	1.22E+15	2.09E+13	########	7.34E+15	1.32E+15
sp|P06241|	6	63	40.07202	3.19E+15	7.97E+13	########	1.92E+16	5.02E+15
sp|P06307|	6	63	6.716827	1.06E+14	1.58E+13	########	6.36E+14	9.94E+14
sp|P06396|	6	63	########	6.67E+16	8.97E+16	########	4E+17	6E+18
sp|P06576|	6	63	118.3742	2E+20	1.69E+18	########	1E+21	1E+20
sp|P06702|	6	63	4.58883	1.21E+16	2.64E+15	########	7.26E+16	1.66E+17
sp|P06703|	6	63	9.085861	6.69E+16	7.36E+15	########	4.01E+17	4.64E+17
sp|P06730|	6	63	1.25421	2.03E+12	1.62E+12	########	1.22E+13	1.02E+14

For Peer Review Only

sp|P06733|	6	63	157.105	1E+20	9.02E+17	########	9E+20	6E+19
sp|P06744|	6	63	4.564811	1.85E+18	4.05E+17	########	1E+19	3E+19
sp|P06748|	6	63	76.2014	3.59E+16	4.71E+14	########	2.15E+17	2.97E+16
sp|P06753|	6	63	55.02056	5.4E+16	9.81E+14	########	3.24E+17	6E+16
sp|P06756|	6	63	46.02234	2.65E+15	5.76E+13	########	1.59E+16	3.63E+15
sp|P07093|	6	63	1.873481	8.55E+11	4.56E+11	########	5.13E+12	2.88E+13
sp|P07099|	6	63	81.76121	6.35E+17	7.77E+15	########	4E+18	########
sp|P07108|	6	63	55.05465	8.19E+17	1.49E+16	########	5E+18	9.37E+17
sp|P07195|	6	63	99.90763	3E+19	2.63E+17	########	2E+20	2E+19
sp|P07196|	6	63	49.86076	2E+20	4.16E+18	########	1E+21	3E+20
sp|P07197|	6	63	61.56866	1E+20	2.29E+18	########	8E+20	1E+20
sp|P07203|	6	63	33.3749	1.95E+16	5.83E+14	########	1.17E+17	3.67E+16
sp|P07237|	6	63	18.76905	1.59E+16	8.49E+14	########	9.56E+16	5.35E+16
sp|P07305|	6	63	40.32853	7.96E+15	1.97E+14	########	4.78E+16	1.24E+16
sp|P07311|	6	63	12.5792	4.07E+14	3.24E+13	########	2.44E+15	2E+15
sp|P07339|	6	63	143.8241	1.82E+18	1.27E+16	########	1E+19	7.98E+17
sp|P07355|	6	63	9.032724	2E+17	2.45E+16	########	1E+18	1.55E+18
sp|P07384|	6	63	16.49355	8.35E+15	5.06E+14	########	5.01E+16	3.19E+16
sp|P07437|	6	63	56.34104	2E+18	3.64E+16	########	1E+19	2.29E+18
sp|P07602|	6	63	8.864282	3.38E+16	3.81E+15	########	2.03E+17	2.4E+17
sp|P07686|	6	63	34.6535	5.98E+14	1.73E+13	########	3.59E+15	1.09E+15
sp|P07737|	6	63	47.27372	2.95E+17	6.24E+15	########	1.77E+18	########
sp|P07738|	6	63	9.710293	1.05E+14	1.08E+13	########	6.28E+14	6.8E+14
sp|P07741|	6	63	4.054178	7.7E+14	1.9E+14	########	4.62E+15	1.2E+16
sp|P07814|	6	63	18.90784	6.85E+15	3.63E+14	########	4.11E+16	2.28E+16
sp|P07858|	6	63	52.43241	2.05E+16	3.91E+14	########	1.23E+17	2.46E+16
sp|P07900|	6	63	########	########	6.05E+17	########	4E+18	4E+19
sp|P07910|	6	63	180.8042	1.13E+17	6.25E+14	########	6.78E+17	3.93E+16
sp|P07919|	6	63	67.91843	2.52E+17	3.7E+15	########	1.51E+18	2.33E+17
sp|P07947|	6	63	5.276517	3.92E+13	7.43E+12	########	2.35E+14	4.68E+14
sp|P07954|	6	63	38.15911	4.19E+17	1.1E+16	########	2.52E+18	6.92E+17
sp|P08123|	6	63	1.651379	1.79E+13	1.08E+13	########	1.07E+14	6.82E+14
sp|P08133|	6	63	28.36603	4E+18	1.38E+17	########	2E+19	9E+18
sp|P08134|	6	63	1.409345	7.83E+13	5.55E+13	########	4.7E+14	3.5E+15
sp|P08183|	6	63	9.040204	2.52E+13	2.79E+12	########	1.51E+14	1.76E+14
sp|P08195|	6	63	108.0066	1.13E+18	1.04E+16	########	7E+18	6.56E+17
sp|P08237|	6	63	50.39023	4.78E+17	9.48E+15	########	2.87E+18	5.97E+17
sp|P08238|	6	63	5.99511	4.4E+17	7.33E+16	########	3E+18	5E+18
sp|P08240|	6	63	1.773787	1.34E+12	7.58E+11	########	8.07E+12	4.78E+13
sp|P08246|	6	63	########	7.36E+13	9.46E+13	########	4.41E+14	5.96E+15
sp|P08247|	6	63	8.962098	3.15E+17	3.52E+16	########	1.89E+18	2.22E+18
sp|P08311|	6	63	########	5.69E+13	6.07E+13	########	3.42E+14	3.83E+15
sp|P08397|	6	63	2.01102	3E+13	1.49E+13	########	1.8E+14	9.41E+14
sp|P08559|	6	63	34.76125	3.16E+17	9.1E+15	########	1.9E+18	5.73E+17
sp|P08571|	6	63	1.253205	7.77E+14	6.2E+14	########	4.66E+15	3.9E+16
sp|P08572|	6	63	14.66196	1.83E+15	1.25E+14	########	1.1E+16	7.85E+15
sp|P08574|	6	63	62.67903	3.86E+17	6.15E+15	########	2.31E+18	3.88E+17
sp|P08575|	6	63	3.212524	5.72E+13	1.78E+13	########	3.43E+14	1.12E+15
sp|P08603|	6	63	6.872503	1.03E+14	1.5E+13	########	6.21E+14	9.48E+14
sp|P08621|	6	63	24.06763	4.9E+14	2.04E+13	########	2.94E+15	1.28E+15
sp|P08670|	6	63	9.507025	2.08E+18	2.18E+17	########	1E+19	1E+19
sp|P08708|	6	63	64.8444	1.62E+17	2.5E+15	########	9.74E+17	1.58E+17
sp|P08754|	6	63	42.11697	1.59E+15	3.78E+13	########	9.54E+15	2.38E+15
sp|P08758|	6	63	11.1044	1.08E+18	9.73E+16	########	6E+18	6E+18
sp|P08865|	6	63	55.14284	1.27E+18	2.31E+16	########	8E+18	1.45E+18
sp|P09012|	6	63	10.63132	1.81E+14	1.7E+13	########	1.09E+15	1.07E+15

For Peer Review Only

sp|P09104|	6	63	151.9331	2E+20	1.19E+18	########	1E+21	8E+19
sp|P09110|	6	63	47.29995	6.96E+15	1.47E+14	########	4.17E+16	9E+15
sp|P09211|	6	63	126.132	3.7E+18	2.93E+16	########	2E+19	1.85E+18
sp|P09382|	6	63	131.6308	2E+18	1.78E+16	########	1E+19	1.12E+18
sp|P09417|	6	63	11.65209	2.17E+18	1.86E+17	########	1E+19	1E+19
sp|P09429|	6	63	21.20467	1.11E+16	5.25E+14	########	6.67E+16	3.31E+16
sp|P09455|	6	63	16.19927	2.44E+15	1.51E+14	########	1.47E+16	9.5E+15
sp|P09471|	6	63	50.19354	1E+19	2.29E+17	########	7E+19	1E+19
sp|P09488|	6	63	8.291662	9.7E+16	1.17E+16	########	5.82E+17	7.37E+17
sp|P09493|	6	63	45.15723	1.9E+16	4.21E+14	########	1.14E+17	2.65E+16
sp|P09496|	6	63	29.9691	6.74E+16	2.25E+15	########	4.04E+17	1.42E+17
sp|P09497|	6	63	16.82728	6.56E+16	3.9E+15	########	########	2.46E+17
sp|P09525|	6	63	24.07863	4.37E+15	1.82E+14	########	2.62E+16	1.14E+16
sp|P09543|	6	63	15.95227	2E+20	1E+19	########	1E+21	8E+20
sp|P09622|	6	63	29.67243	########	9.45E+15	########	1.68E+18	5.95E+17
sp|P09651|	6	63	93.06593	8.5E+16	9.13E+14	########	5.1E+17	6E+16
sp|P09669|	6	63	28.04941	5.88E+16	2.1E+15	########	3.53E+17	1.32E+17
sp|P09874|	6	63	67.07797	3.94E+16	5.88E+14	########	2.37E+17	3.71E+16
sp|P09936|	6	63	15.823	1E+19	6.14E+17	########	6E+19	4E+19
sp|P09960|	6	63	78.34693	4.81E+16	6.14E+14	########	2.89E+17	3.87E+16
sp|P09972|	6	63	116.2598	3E+19	2.48E+17	########	2E+20	2E+19
sp|P0C0L4	6	63	########	4.94E+13	2.15E+14	########	2.96E+14	1.35E+16
sp|P0C0L5	6	63	2.014295	5.75E+16	2.86E+16	########	3.45E+17	1.8E+18
sp|P0C7M8	6	63	2.416494	4.15E+13	1.72E+13	########	2.49E+14	1.08E+15
sp|P0CG3C	6	63	6.165475	3.88E+15	6.29E+14	########	2.33E+16	4E+16
sp|P0DJI8|S	6	63	3.068999	1.96E+15	6.38E+14	########	1.17E+16	4.02E+16
sp|P0DMV9	6	63	26.38835	2.95E+18	1.12E+17	########	2E+19	7E+18
sp|P0DOY3	6	63	6.691706	5.28E+16	7.89E+15	########	3.17E+17	4.97E+17
sp|P0DP25	6	63	200.6524	8E+19	4.12E+17	########	5E+20	3E+19
sp|P0DPI2|	6	63	68.45998	2.49E+17	3.64E+15	########	1.5E+18	2.29E+17
sp|P10114|	6	63	29.25241	1.24E+16	4.23E+14	########	7E+16	2.66E+16
sp|P10155|	6	63	57.69417	2.27E+15	3.94E+13	########	1.36E+16	2.48E+15
sp|P10253|	6	63	35.82032	7.05E+14	1.97E+13	########	4.23E+15	1.24E+15
sp|P10301|	6	63	23.05049	1.85E+14	8.03E+12	########	1E+15	5.06E+14
sp|P10412|	6	63	28.83954	2.27E+17	7.87E+15	########	1.36E+18	5E+17
sp|P10451|	6	63	1.750265	9.24E+15	5.28E+15	########	5.54E+16	3.33E+17
sp|P10515|	6	63	35.59309	5.9E+17	1.66E+16	########	3.54E+18	1.04E+18
sp|P10599|	6	63	65.46654	2.43E+17	3.71E+15	########	1.46E+18	2.34E+17
sp|P10606|	6	63	34.23921	3.02E+17	8.82E+15	########	1.81E+18	5.55E+17
sp|P10620|	6	63	3.385191	3.7E+13	1.09E+13	########	2.22E+14	6.89E+14
sp|P10636|	6	63	5.645668	1.46E+18	2.59E+17	########	9E+18	2E+19
sp|P10644|	6	63	9.820316	2.81E+15	2.86E+14	########	1.69E+16	2E+16
sp|P10645|	6	63	2.937404	1.77E+14	6.02E+13	########	1.06E+15	3.79E+15
sp|P10768|	6	63	64.25398	5.69E+17	8.85E+15	########	3.41E+18	5.57E+17
sp|P10809|	6	63	79.02666	3.88E+18	4.92E+16	########	2E+19	3.1E+18
sp|P10909|	6	63	6.678772	4.86E+16	7.28E+15	########	2.92E+17	5E+17
sp|P10915|	6	63	14.95045	5.33E+16	3.56E+15	########	3.2E+17	2.25E+17
sp|P11021|	6	63	14.67152	7.05E+16	4.8E+15	########	4.23E+17	3.03E+17
sp|P11047|	6	63	32.15307	4.03E+15	1.25E+14	########	2.42E+16	7.9E+15
sp|P11137|	6	63	3.713366	2.04E+17	5.49E+16	########	1E+18	3E+18
sp|P11142|	6	63	55.83185	2E+19	2.87E+17	########	1E+20	2E+19
sp|P11166|	6	63	35.27558	2.17E+15	6.16E+13	########	1.3E+16	3.88E+15
sp|P11169|	6	63	18.11996	3.01E+15	1.66E+14	########	1.81E+16	1.05E+16
sp|P11177|	6	63	31.25229	6.54E+17	2.09E+16	########	3.92E+18	1.32E+18
sp|P11182|	6	63	51.24036	5.27E+15	1.03E+14	########	3.16E+16	6.48E+15
sp|P11215|	6	63	23.68329	1.88E+14	7.93E+12	########	1.13E+15	4.99E+14

For Peer Review Only

sp|P11216|	6	63	14.80086	3.77E+17	2.55E+16	########	2.26E+18	1.61E+18
sp|P11217|	6	63	2.605577	3.15E+16	1.21E+16	########	1.89E+17	7.62E+17
sp|P11233|	6	63	94.27004	6.61E+16	7.01E+14	########	3.97E+17	4.42E+16
sp|P11234|	6	63	4.169665	9.78E+13	2.35E+13	########	5.87E+14	1.48E+15
sp|P11274|	6	63	4.106321	6.41E+12	1.56E+12	########	3.85E+13	9.84E+13
sp|P11277|	6	63	1.161347	1.75E+14	1.5E+14	########	1.05E+15	9E+15
sp|P11279|	6	63	65.92861	2.46E+16	3.74E+14	########	1.48E+17	2.36E+16
sp|P11310|	6	63	19.87495	4.99E+15	2.51E+14	########	3E+16	1.58E+16
sp|P11413|	6	63	103.8809	1.55E+16	1.49E+14	########	9.31E+16	9.41E+15
sp|P11498|	6	63	140.41	3.81E+17	2.71E+15	########	2.29E+18	1.71E+17
sp|P11532|	6	63	9.540092	1.78E+14	1.86E+13	########	1.07E+15	1.17E+15
sp|P11586|	6	63	96.16452	1.72E+16	1.79E+14	########	1.03E+17	1E+16
sp|P11766|	6	63	85.43764	1.59E+17	1.86E+15	########	9.55E+17	1.17E+17
sp|P11940|	6	63	50.1654	1.21E+16	2.41E+14	########	7.26E+16	1.52E+16
sp|P12036|	6	63	33.02455	9E+18	2.64E+17	########	5E+19	2E+19
sp|P12074|	6	63	24.48922	4.55E+16	1.86E+15	########	2.73E+17	1E+17
sp|P12081|	6	63	2.524666	1.46E+14	5.8E+13	########	8.78E+14	3.65E+15
sp|P12109|	6	63	11.17992	9.35E+14	8.36E+13	########	5.61E+15	5E+15
sp|P12110|	6	63	3.270853	7.56E+12	2.31E+12	########	4.53E+13	1.46E+14
sp|P12111|	6	63	13.29432	1.12E+16	8.42E+14	########	6.72E+16	5.3E+16
sp|P12235|	6	63	80.48932	4E+17	5.02E+15	########	2E+18	########
sp|P12236|	6	63	57.63174	8E+18	1.37E+17	########	5E+19	9E+18
sp|P12268|	6	63	3.226649	4.87E+13	1.51E+13	########	2.92E+14	9.5E+14
sp|P12270|	6	63	5.229484	2.29E+13	4.38E+12	########	1.38E+14	2.76E+14
sp|P12277|	6	63	50.37059	1E+20	2.57E+18	########	8E+20	2E+20
sp|P12429|	6	63	15.56752	5.17E+14	3.32E+13	########	3.1E+15	2E+15
sp|P12532|	6	63	23.42927	1.53E+18	6.55E+16	########	9E+18	4E+18
sp|P12694|	6	63	2.091376	1.93E+13	9.21E+12	########	1.16E+14	5.8E+14
sp|P12814|	6	63	38.92245	6.16E+17	2E+16	########	3.69E+18	1E+18
sp|P12931|	6	63	12.43279	5.83E+13	4.69E+12	########	3.5E+14	2.96E+14
sp|P12955|	6	63	48.06682	1.93E+16	4.01E+14	########	1.16E+17	2.53E+16
sp|P12956|	6	63	68.40513	9.07E+16	1.33E+15	########	5.44E+17	8.36E+16
sp|P13010|	6	63	58.69217	1.47E+17	2.51E+15	########	8.83E+17	1.58E+17
sp|P13073|	6	63	27.77275	2.72E+17	9.78E+15	########	2E+18	6.16E+17
sp|P13473|	6	63	11.93463	9.41E+14	7.89E+13	########	5.65E+15	4.97E+15
sp|P13489|	6	63	2.516079	2.92E+16	1.16E+16	########	1.75E+17	7E+17
sp|P13521|	6	63	6.244006	1.66E+15	2.65E+14	########	9.94E+15	1.67E+16
sp|P13591|	6	63	31.80297	3.77E+18	1.18E+17	########	2E+19	7E+18
sp|P13611|	6	63	11.14563	2E+19	1.96E+18	########	1E+20	1E+20
sp|P13637|	6	63	33.23691	3E+19	8.18E+17	########	2E+20	5E+19
sp|P13639|	6	63	12.32521	7.53E+16	6.11E+15	########	4.52E+17	4E+17
sp|P13667|	6	63	12.12048	3.58E+14	2.95E+13	########	2.15E+15	1.86E+15
sp|P13693|	6	63	47.89731	1.63E+16	3.4E+14	########	9.77E+16	2.14E+16
sp|P13716|	6	63	86.6586	1.57E+17	1.81E+15	########	9E+17	1.14E+17
sp|P13726|	6	63	13.4455	3.84E+15	2.85E+14	########	2.3E+16	1.8E+16
sp|P13796|	6	63	12.63491	6.25E+15	4.95E+14	########	3.75E+16	3.12E+16
sp|P13797|	6	63	26.49307	8.01E+15	3.02E+14	########	4.8E+16	1.9E+16
sp|P13798|	6	63	168.4671	4.42E+16	2.62E+14	########	2.65E+17	1.65E+16
sp|P13804|	6	63	35.24241	3.68E+16	1.04E+15	########	########	6.58E+16
sp|P13807|	6	63	5.223078	9.43E+14	1.81E+14	########	5.66E+15	1.14E+16
sp|P13861|	6	63	6.834846	1E+15	1.47E+14	########	6.02E+15	9.26E+15
sp|P13929|	6	63	19.81488	4.59E+14	2.32E+13	########	2.75E+15	1E+15
sp|P13987|	6	63	12.76945	9.69E+15	7.59E+14	########	5.82E+16	4.78E+16
sp|P14136|	6	63	6.907592	8E+20	1E+20	########	5E+21	7E+21
sp|P14174|	6	63	35.22859	1.08E+18	3.07E+16	########	6E+18	1.93E+18
sp|P14209|	6	63	14.81238	8.32E+14	5.62E+13	########	4.99E+15	3.54E+15

For Review Only

sp|P14314|	6	63	6.667077	1.25E+15	1.87E+14	########	7.47E+15	1.18E+16
sp|P14324|	6	63	62.674	2.13E+16	3.4E+14	########	1.28E+17	2.14E+16
sp|P14384|	6	63	4.039008	5.74E+14	1.42E+14	########	3.45E+15	8.96E+15
sp|P14406|	6	63	40.97451	1.87E+17	4.56E+15	########	1.12E+18	3E+17
sp|P14415|	6	63	55.87412	1.05E+18	1.88E+16	########	6E+18	1E+18
sp|P14543|	6	63	14.11249	1.55E+15	1.09E+14	########	9.27E+15	6.9E+15
sp|P14550|	6	63	1.560694	8.01E+15	5.13E+15	########	4.81E+16	3.23E+17
sp|P14618|	6	63	170.46	4E+20	2.22E+18	########	2E+21	1E+20
sp|P14621|	6	63	35.22393	6.38E+15	1.81E+14	########	3.83E+16	1E+16
sp|P14625|	6	63	29.81294	1.51E+17	5.06E+15	########	9.05E+17	3.19E+17
sp|P14649|	6	63	3.605301	8.82E+13	2.45E+13	########	5.29E+14	2E+15
sp|P14854|	6	63	47.13364	3.17E+17	6.73E+15	########	2E+18	4.24E+17
sp|P14866|	6	63	27.88728	3.88E+16	1.39E+15	########	########	8.76E+16
sp|P14867|	6	63	7.516748	1.91E+14	2.54E+13	########	1.15E+15	1.6E+15
sp|P14868|	6	63	28.8547	6.84E+15	2.37E+14	########	4.11E+16	1.49E+16
sp|P14923|	6	63	6.712845	3.25E+13	4.84E+12	########	1.95E+14	3.05E+14
sp|P14927|	6	63	54.58927	1.36E+17	2.48E+15	########	8.13E+17	1.56E+17
sp|P15056|	6	63	1.711059	3.29E+12	1.92E+12	########	1.97E+13	1.21E+14
sp|P15104|	6	63	26.82937	3E+18	1.3E+17	########	2E+19	8E+18
sp|P15121|	6	63	192.4583	5.62E+17	2.92E+15	########	3E+18	1.84E+17
sp|P15170|	6	63	########	1.88E+13	4.01E+13	########	1.13E+14	2.53E+15
sp|P15259|	6	63	4.287046	5.3E+15	1.24E+15	########	3.18E+16	7.78E+16
sp|P15311|	6	63	14.00048	2.01E+16	1.44E+15	########	1.21E+17	9.06E+16
sp|P15374|	6	63	1.19911	1.89E+13	1.58E+13	########	1.13E+14	9.93E+14
sp|P15531|	6	63	15.73535	1.13E+17	7.16E+15	########	7E+17	5E+17
sp|P15559|	6	63	5.333917	2.44E+14	4.57E+13	########	1.46E+15	2.88E+15
sp|P15880|	6	63	46.63097	7.59E+16	1.63E+15	########	########	1.03E+17
sp|P15882|	6	63	3.485271	1.66E+13	4.77E+12	########	9.97E+13	3E+14
sp|P15927|	6	63	########	7.91E+12	1.2E+13	########	4.75E+13	7.58E+14
sp|P15954|	6	63	18.67595	6.41E+15	3.43E+14	########	3.85E+16	2.16E+16
sp|P16070|	6	63	3.74994	4.01E+16	1.07E+16	########	2.4E+17	7E+17
sp|P16083|	6	63	22.40603	3.36E+16	1.5E+15	########	########	9.44E+16
sp|P16104|	6	63	40.34585	3.53E+17	8.74E+15	########	2.12E+18	5.5E+17
sp|P16112|	6	63	9.550113	5.49E+16	5.75E+15	########	3.3E+17	4E+17
sp|P16144|	6	63	7.251459	3.05E+14	4.21E+13	########	1.83E+15	2.65E+15
sp|P16152|	6	63	########	1.04E+17	1.75E+17	########	6.27E+17	1E+19
sp|P16157|	6	63	5.442047	4E+14	7.35E+13	########	2.4E+15	4.63E+15
sp|P16219|	6	63	8.941705	4.36E+16	4.88E+15	########	2.62E+17	3.07E+17
sp|P16298|	6	63	59.15465	1.12E+16	1.9E+14	########	6.75E+16	1E+16
sp|P16389|	6	63	4.317407	5.09E+15	1.18E+15	########	3.05E+16	7E+16
sp|P16403|	6	63	15.39313	3.15E+14	2.05E+13	########	1.89E+15	1.29E+15
sp|P16435|	6	63	28.94995	1.34E+15	4.62E+13	########	8.02E+15	2.91E+15
sp|P16615|	6	63	36.80034	4.48E+17	1.22E+16	########	2.69E+18	7.67E+17
sp|P16870|	6	63	1.808477	2.68E+14	1.48E+14	########	1.61E+15	9.35E+15
sp|P16930|	6	63	47.51826	8.94E+15	1.88E+14	########	5.36E+16	1.19E+16
sp|P16949|	6	63	180.2722	7E+17	4.02E+15	########	4E+18	2.53E+17
sp|P17066|	6	63	5.40816	6.87E+16	1.27E+16	########	4.12E+17	########
sp|P17096|	6	63	15.85725	5.71E+14	3.6E+13	########	3.42E+15	2.27E+15
sp|P17152|	6	63	3.395718	2.79E+12	8.21E+11	########	1.67E+13	5.17E+13
sp|P17174|	6	63	209.3398	3E+19	1.23E+17	########	2E+20	8E+18
sp|P17252|	6	63	33.61882	2E+16	5.94E+14	########	1.2E+17	4E+16
sp|P17301|	6	63	1.880913	6.42E+14	3.41E+14	########	3.85E+15	2.15E+16
sp|P17302|	6	63	20.98621	2E+16	9.53E+14	########	1.2E+17	6.01E+16
sp|P17540|	6	63	########	2.1E+14	3.78E+14	########	1.26E+15	2.38E+16
sp|P17568|	6	63	3.400795	3.32E+15	9.75E+14	########	1.99E+16	6.14E+16
sp|P17600|	6	63	26.32227	7E+18	2.7E+17	########	4E+19	2E+19

For Peer Review Only

sp|P17655|	6	63	54.89607	1.89E+16	3.44E+14	########	1.13E+17	2.17E+16
sp|P17677|	6	63	4.816652	2.37E+17	4.93E+16	########	1.42E+18	3.11E+18
sp|P17812|	6	63	32.98881	2.62E+14	7.95E+12	########	1.57E+15	5.01E+14
sp|P17844|	6	63	25.35259	7.76E+14	3.06E+13	########	4.65E+15	1.93E+15
sp|P17858|	6	63	29.79787	8.91E+16	2.99E+15	########	5.35E+17	1.88E+17
sp|P17900|	6	63	14.97671	1.01E+14	6.72E+12	########	6.03E+14	4.23E+14
sp|P17931|	6	63	13.08699	1.54E+15	1.18E+14	########	9.27E+15	7.44E+15
sp|P17980|	6	63	24.62974	2.98E+15	1.21E+14	########	1.79E+16	7.62E+15
sp|P17987|	6	63	31.2886	3.32E+16	1.06E+15	########	1.99E+17	6.68E+16
sp|P18077|	6	63	29.93851	1.97E+15	6.56E+13	########	1.18E+16	4.14E+15
sp|P18085|	6	63	65.77042	1.13E+16	1.72E+14	########	6.79E+16	1.08E+16
sp|P18124|	6	63	62.78244	3.17E+17	5.04E+15	########	1.9E+18	3.18E+17
sp|P18206|	6	63	31.46934	4.14E+15	1.32E+14	########	2.48E+16	8.29E+15
sp|P18433|	6	63	5.915769	7.64E+13	1.29E+13	########	4.59E+14	8.14E+14
sp|P18507|	6	63	32.4472	1.9E+15	5.84E+13	########	1.14E+16	3.68E+15
sp|P18621|	6	63	43.764	8.04E+16	1.84E+15	########	4.83E+17	1.16E+17
sp|P18669|	6	63	6.167713	8.9E+17	1.44E+17	########	5E+18	9E+18
sp|P18859|	6	63	68.51372	9.34E+16	1.36E+15	########	5.6E+17	8.58E+16
sp|P19022|	6	63	10.27118	3.67E+15	3.57E+14	########	2.2E+16	2.25E+16
sp|P19086|	6	63	30.22376	1.97E+16	6.51E+14	########	1.18E+17	4.1E+16
sp|P19174|	6	63	6.023693	1.54E+13	2.55E+12	########	9.23E+13	1.61E+14
sp|P19338|	6	63	25.12857	6.97E+16	2.77E+15	########	4.18E+17	1.75E+17
sp|P19367|	6	63	38.90143	6E+18	1.58E+17	########	4E+19	1E+19
sp|P19404|	6	63	58.15364	1E+17	2.56E+15	########	########	1.61E+17
sp|P19525|	6	63	14.16411	1.49E+14	1.05E+13	########	8.94E+14	6.63E+14
sp|P19623|	6	63	47.85817	7.48E+14	1.56E+13	########	4.49E+15	9.85E+14
sp|P19634|	6	63	3.835255	7.21E+14	1.88E+14	########	4.33E+15	1.18E+16
sp|P19652|	6	63	9.946315	5.8E+14	5.83E+13	########	3.48E+15	3.67E+15
sp|P19784|	6	63	2.741788	1.18E+13	4.29E+12	########	7.06E+13	2.7E+14
sp|P19971|	6	63	11.41183	1.33E+14	1.16E+13	########	7.96E+14	7.33E+14
sp|P20020|	6	63	11.57846	2.98E+17	2.57E+16	########	1.79E+18	1.62E+18
sp|P20073|	6	63	115.2601	1.56E+17	1.35E+15	########	9E+17	8.51E+16
sp|P20290|	6	63	31.83186	3.91E+15	1.23E+14	########	2.34E+16	7.74E+15
sp|P20336|	6	63	7.471301	5.26E+16	7.04E+15	########	3.16E+17	4.43E+17
sp|P20337|	6	63	3.794448	3.72E+13	9.8E+12	########	2.23E+14	6.17E+14
sp|P20338|	6	63	19.89801	2.47E+14	1.24E+13	########	1.48E+15	7.81E+14
sp|P20339|	6	63	4.05376	7.92E+14	1.95E+14	########	4.75E+15	1.23E+16
sp|P20340|	6	63	20.58073	1.02E+15	4.94E+13	########	6.1E+15	3E+15
sp|P20618|	6	63	107.3968	1.37E+16	1.28E+14	########	8.22E+16	8.04E+15
sp|P20645|	6	63	4.69963	1E+14	2.13E+13	########	6E+14	1.34E+15
sp|P20674|	6	63	52.42427	1.56E+18	2.98E+16	########	9E+18	2E+18
sp|P20700|	6	63	111.9567	1.57E+15	1.4E+13	########	9.42E+15	8.83E+14
sp|P20774|	6	63	1.800728	5.82E+13	3.23E+13	########	3.49E+14	2.04E+15
sp|P20810|	6	63	48.21419	4.16E+15	8.62E+13	########	2.49E+16	5.43E+15
sp|P20916|	6	63	3.610859	1.73E+17	4.79E+16	########	1.04E+18	3.01E+18
sp|P20936|	6	63	2.451163	2.79E+13	1.14E+13	########	1.67E+14	7.16E+14
sp|P21266|	6	63	1.002169	1.08E+16	1.08E+16	########	6.5E+16	7E+17
sp|P21281|	6	63	5.288082	6.44E+17	1.22E+17	########	4E+18	8E+18
sp|P21283|	6	63	9.300832	2.53E+16	2.72E+15	########	1.52E+17	1.71E+17
sp|P21291|	6	63	4.065948	2.22E+16	5.47E+15	########	1.33E+17	3.44E+17
sp|P21333|	6	63	5.782406	1.04E+16	1.79E+15	########	6.21E+16	1.13E+17
sp|P21359|	6	63	14.71205	1.8E+14	1.22E+13	########	1.08E+15	7.72E+14
sp|P21397|	6	63	24.01542	6.87E+15	2.86E+14	########	4.12E+16	1.8E+16
sp|P21399|	6	63	114.8135	3.02E+16	2.63E+14	########	1.81E+17	1.66E+16
sp|P21579|	6	63	13.36433	3E+18	2.07E+17	########	2E+19	1E+19
sp|P21589|	6	63	44.72412	3.4E+15	7.61E+13	########	2.04E+16	4.8E+15

For Peer Review Only

sp|P21695|	6	63	11.3027	2.54E+16	2.25E+15	########	1.53E+17	########
sp|P21796|	6	63	23.62342	2E+18	1.02E+17	########	1E+19	6E+18
sp|P21810|	6	63	3.606608	6.17E+15	1.71E+15	########	3.7E+16	1.08E+17
sp|P21912|	6	63	76.49899	4.52E+16	5.91E+14	########	2.71E+17	3.72E+16
sp|P21926|	6	63	9.362273	6E+18	7E+17	########	4E+19	4E+19
sp|P21953|	6	63	29.0145	1.5E+15	5.16E+13	########	8.99E+15	3.25E+15
sp|P21964|	6	63	15.63503	2.97E+16	1.9E+15	########	1.78E+17	1.2E+17
sp|P21980|	6	63	22.17977	2.75E+15	1.24E+14	########	1.65E+16	7.81E+15
sp|P22033|	6	63	25.2597	1.07E+15	4.25E+13	########	6.45E+15	3E+15
sp|P22059|	6	63	########	1.13E+12	2.53E+12	########	6.77E+12	1.59E+14
sp|P22061|	6	63	153.6772	3E+18	1.98E+16	########	2E+19	1.25E+18
sp|P22102|	6	63	3.236064	5.26E+14	1.63E+14	########	3.16E+15	1E+16
sp|P22234|	6	63	97.71605	1.34E+17	1.37E+15	########	8.04E+17	8.64E+16
sp|P22307|	6	63	11.17937	1.55E+14	1.39E+13	########	9.3E+14	8.74E+14
sp|P22314|	6	63	1.068199	1.46E+17	1.37E+17	########	8.78E+17	9E+18
sp|P22392|	6	63	8.170462	1.2E+16	1.47E+15	########	7.22E+16	9E+16
sp|P22570|	6	63	10.49845	4.23E+14	4.03E+13	########	2.54E+15	3E+15
sp|P22626|	6	63	211.7458	7.09E+17	3.35E+15	########	4.25E+18	2.11E+17
sp|P22676|	6	63	91.45246	3.34E+16	3.65E+14	########	2E+17	2.3E+16
sp|P22694|	6	63	3.72054	4.22E+15	1.13E+15	########	2.53E+16	7.14E+16
sp|P22695|	6	63	77.80805	2E+18	3.08E+16	########	1E+19	2E+18
sp|P22732|	6	63	2.030594	3.97E+13	1.95E+13	########	2.38E+14	1.23E+15
sp|P22748|	6	63	2.680868	2.55E+14	9.51E+13	########	1.53E+15	5.99E+15
sp|P22830|	6	63	9.64796	1.48E+15	1.53E+14	########	8.87E+15	9.65E+15
sp|P23141|	6	63	1.503516	4.87E+13	3.24E+13	########	2.92E+14	2E+15
sp|P23142|	6	63	5.717767	2.59E+14	4.53E+13	########	1.56E+15	2.86E+15
sp|P23246|	6	63	55.6249	2.74E+16	4.93E+14	########	1.64E+17	3.1E+16
sp|P23258|	6	63	4.429125	3.21E+13	7.24E+12	########	1.92E+14	4.56E+14
sp|P23284|	6	63	31.60754	1.57E+16	4.96E+14	########	9.41E+16	3.13E+16
sp|P23297|	6	63	127.1829	5E+18	3.78E+16	########	3E+19	2E+18
sp|P23368|	6	63	37.28876	8.48E+15	2.27E+14	########	5.09E+16	1.43E+16
sp|P23381|	6	63	147.7143	9.3E+16	6.29E+14	########	5.58E+17	3.97E+16
sp|P23396|	6	63	46.41884	3E+17	7.38E+15	########	2E+18	4.65E+17
sp|P23434|	6	63	40.83778	2.44E+16	5.99E+14	########	1.47E+17	4E+16
sp|P23468|	6	63	22.02654	6.6E+14	3E+13	########	3.96E+15	1.89E+15
sp|P23469|	6	63	1.484712	1.94E+12	1.31E+12	########	1.16E+13	8.23E+13
sp|P23471|	6	63	38.98344	8.11E+16	2.08E+15	########	########	1E+17
sp|P23515|	6	63	16.07105	1.74E+17	1.08E+16	########	1.04E+18	6.8E+17
sp|P23526|	6	63	92.02016	7.75E+16	8.42E+14	########	4.65E+17	5.31E+16
sp|P23528|	6	63	123.9564	1E+19	1.11E+17	########	8E+19	7E+18
sp|P23588|	6	63	28.18287	2.11E+15	7.49E+13	########	1.27E+16	4.72E+15
sp|P23610|	6	63	4.736324	8.13E+13	1.72E+13	########	4.88E+14	1.08E+15
sp|P23634|	6	63	39.47338	1.02E+18	2.58E+16	########	6E+18	1.63E+18
sp|P23677|	6	63	3.938162	1.18E+14	3.01E+13	########	7.11E+14	1.9E+15
sp|P23743|	6	63	4.021429	6.64E+11	1.65E+11	########	3.98E+12	1.04E+13
sp|P23763|	6	63	9.407451	2.46E+16	2.61E+15	########	1.47E+17	1.65E+17
sp|P23786|	6	63	8.994342	3.21E+14	3.57E+13	########	1.93E+15	2E+15
sp|P24043|	6	63	34.68191	7.73E+14	2.23E+13	########	4.64E+15	1.4E+15
sp|P24534|	6	63	1.567562	1.08E+15	6.87E+14	########	6.46E+15	4E+16
sp|P24539|	6	63	65.66038	7.79E+16	1.19E+15	########	4.67E+17	7.47E+16
sp|P24588|	6	63	9.907098	3.07E+13	3.1E+12	########	1.84E+14	1.95E+14
sp|P24666|	6	63	68.89559	2.64E+16	3.83E+14	########	1.58E+17	2.41E+16
sp|P24752|	6	63	16.18093	5.28E+16	3.26E+15	########	3.17E+17	2.06E+17
sp|P24821|	6	63	2.848884	2.2E+16	7.73E+15	########	1.32E+17	4.87E+17
sp|P25098|	6	63	8.244657	5.26E+13	6.38E+12	########	3.16E+14	4.02E+14
sp|P25325|	6	63	30.66324	5.01E+15	1.63E+14	########	3E+16	1.03E+16

For Peer Review Only

sp|P25398|	6	63	51.10278	6.92E+16	1.35E+15	########	4.15E+17	8.53E+16
sp|P25685|	6	63	19.21153	3.52E+14	1.83E+13	########	2.11E+15	1.15E+15
sp|P25686|	6	63	30.70752	1.18E+15	3.83E+13	########	7.06E+15	2.42E+15
sp|P25705|	6	63	122.7362	9E+19	7.12E+17	########	5E+20	4E+19
sp|P25713|	6	63	45.40175	2.16E+16	4.77E+14	########	########	3E+16
sp|P25786|	6	63	116.2348	3.29E+16	2.83E+14	########	1.98E+17	1.79E+16
sp|P25787|	6	63	171.6251	3.51E+16	2.04E+14	########	########	1E+16
sp|P25788|	6	63	32.377	7.7E+15	2.38E+14	########	4.62E+16	1.5E+16
sp|P25789|	6	63	103.915	4.87E+16	4.69E+14	########	2.92E+17	2.95E+16
sp|P26012|	6	63	4.928514	4.86E+13	9.87E+12	########	2.92E+14	6.22E+14
sp|P26038|	6	63	22.24096	1.52E+17	6.82E+15	########	9.1E+17	4.29E+17
sp|P26196|	6	63	5.388516	1.95E+14	3.61E+13	########	1.17E+15	2.27E+15
sp|P26232|	6	63	53.97774	1.24E+17	2.3E+15	########	7.46E+17	1.45E+17
sp|P26368|	6	63	7.988312	2.65E+14	3.32E+13	########	1.59E+15	2.09E+15
sp|P26373|	6	63	42.50399	1.85E+17	4.35E+15	########	1.11E+18	2.74E+17
sp|P26378|	6	63	6.488957	1.5E+15	2.31E+14	########	8.99E+15	1.45E+16
sp|P26440|	6	63	10.85224	1.08E+15	9.99E+13	########	6.5E+15	6.29E+15
sp|P26599|	6	63	25.14015	5.61E+14	2.23E+13	########	3.37E+15	1.41E+15
sp|P26639|	6	63	6.611952	1.67E+14	2.53E+13	########	1E+15	1.59E+15
sp|P26640|	6	63	5.880172	2.22E+15	3.78E+14	########	1.33E+16	2E+16
sp|P26641|	6	63	3.825954	1.35E+16	3.53E+15	########	8.11E+16	2.22E+17
sp|P26885|	6	63	27.04186	1.69E+15	6.26E+13	########	1.02E+16	3.94E+15
sp|P26992|	6	63	3.876867	6.44E+14	1.66E+14	########	3.86E+15	1E+16
sp|P27105|	6	63	33.59931	2.6E+16	7.72E+14	########	1.56E+17	4.87E+16
sp|P27144|	6	63	73.1183	1.42E+16	1.94E+14	########	8.51E+16	1.22E+16
sp|P27338|	6	63	4.896728	4.97E+17	1.02E+17	########	2.98E+18	6E+18
sp|P27348|	6	63	3.004783	8.19E+16	2.73E+16	########	4.91E+17	1.72E+18
sp|P27361|	6	63	2.735936	3.59E+15	1.31E+15	########	2.16E+16	8.27E+16
sp|P27449|	6	63	5.105172	1.13E+15	2.21E+14	########	6.76E+15	1.39E+16
sp|P27544|	6	63	3.301567	2.78E+13	8.42E+12	########	1.67E+14	5.3E+14
sp|P27635|	6	63	46.73053	1.83E+17	3.91E+15	########	1.1E+18	2.46E+17
sp|P27695|	6	63	67.25511	5.39E+15	8.02E+13	########	3.24E+16	5.05E+15
sp|P27701|	6	63	6.735871	8.04E+14	1.19E+14	########	4.82E+15	7.52E+15
sp|P27797|	6	63	28.19468	9.09E+16	3.22E+15	########	5.45E+17	2.03E+17
sp|P27816|	6	63	3.026093	4.77E+15	1.58E+15	########	2.86E+16	9.93E+16
sp|P27824|	6	63	69.80712	1.95E+17	2.79E+15	########	1E+18	1.76E+17
sp|P27986|	6	63	4.130098	2.63E+13	6.36E+12	########	1.58E+14	4.01E+14
sp|P28066|	6	63	104.2635	2.08E+16	1.99E+14	########	1.25E+17	1.26E+16
sp|P28070|	6	63	163.4134	5.91E+15	3.62E+13	########	3.55E+16	2.28E+15
sp|P28072|	6	63	45.53393	5.27E+15	1.16E+14	########	3.16E+16	7.3E+15
sp|P28074|	6	63	55.0089	4.62E+15	8.4E+13	########	2.77E+16	5.29E+15
sp|P28161|	6	63	28.21551	1.06E+17	3.75E+15	########	6.34E+17	2.36E+17
sp|P28288|	6	63	35.93582	7.68E+14	2.14E+13	########	4.61E+15	1.35E+15
sp|P28289|	6	63	3.050012	6.74E+12	2.21E+12	########	4.05E+13	1.39E+14
sp|P28331|	6	63	58.3753	5E+18	7.97E+16	########	3E+19	5E+18
sp|P28482|	6	63	25.9862	3.89E+17	1.5E+16	########	2.33E+18	9.43E+17
sp|P28676|	6	63	3.306536	8.66E+12	2.62E+12	########	5.19E+13	1.65E+14
sp|P28838|	6	63	21.93943	3.48E+16	1.59E+15	########	2.09E+17	9.99E+16
sp|P28907|	6	63	6.837163	1.69E+15	2.47E+14	########	1.01E+16	1.55E+16
sp|P29120|	6	63	1.830634	6.15E+12	3.36E+12	########	3.69E+13	2.12E+14
sp|P29144|	6	63	37.43004	4.65E+16	1.24E+15	########	2.79E+17	7.83E+16
sp|P29218|	6	63	126.3609	3.49E+17	2.76E+15	########	2.09E+18	1.74E+17
sp|P29401|	6	63	145.3858	3.67E+18	2.52E+16	########	2E+19	1.59E+18
sp|P29692|	6	63	7.168224	9.39E+14	1.31E+14	########	5.63E+15	8.25E+15
sp|P29762|	6	63	2.163135	7.94E+13	3.67E+13	########	4.77E+14	2.31E+15
sp|P29966|	6	63	6.426271	8.16E+15	1.27E+15	########	4.9E+16	8E+16

For Peer Review Only

sp|P29972|	6	63	4.763093	7.12E+16	1.49E+16	########	4.27E+17	9.42E+17
sp|P29992|	6	63	57.29431	5.72E+15	9.98E+13	########	3.43E+16	6.29E+15
sp|P30038|	6	63	35.46808	7.97E+16	2.25E+15	########	4.78E+17	1.42E+17
sp|P30040|	6	63	9.056167	1E+15	1.31E+14	########	7E+15	8.26E+15
sp|P30041|	6	63	75.75899	3E+19	3.59E+17	########	2E+20	2E+19
sp|P30043|	6	63	1.390426	1.21E+15	8.72E+14	########	7.28E+15	5E+16
sp|P30044|	6	63	14.36038	3.96E+17	2.76E+16	########	2E+18	########
sp|P30046|	6	63	94.78221	6.4E+16	6.76E+14	########	3.84E+17	4E+16
sp|P30048|	6	63	69.01503	3.64E+17	5.28E+15	########	2.18E+18	3.32E+17
sp|P30049|	6	63	54.2552	1.4E+17	2.59E+15	########	8.42E+17	1.63E+17
sp|P30050|	6	63	53.86446	6.42E+16	1.19E+15	########	3.85E+17	7.5E+16
sp|P30084|	6	63	36.67204	1.13E+17	3.07E+15	########	6.75E+17	1.93E+17
sp|P30085|	6	63	17.75168	1.66E+16	9.34E+14	########	9.94E+16	6E+16
sp|P30086|	6	63	186.6608	7E+19	3.87E+17	########	4E+20	2E+19
sp|P30101|	6	63	34.09668	2.87E+17	8.43E+15	########	2E+18	5.31E+17
sp|P30153|	6	63	5.919061	1.33E+17	2.24E+16	########	7.97E+17	########
sp|P30405|	6	63	7.511154	2.35E+14	3.13E+13	########	1.41E+15	1.97E+15
sp|P30419|	6	63	7.123266	1.73E+14	2.43E+13	########	1.04E+15	1.53E+15
sp|P30453|	6	63	7.127615	4.13E+14	5.79E+13	########	2.48E+15	3.65E+15
sp|P30484|	6	63	4.190441	1.55E+14	3.69E+13	########	9.28E+14	2E+15
sp|P30519|	6	63	27.99177	4.17E+15	1.49E+14	########	2.5E+16	9.39E+15
sp|P30520|	6	63	7.989097	1.19E+15	1.49E+14	########	7.12E+15	9.36E+15
sp|P30531|	6	63	17.582	1.29E+16	7.35E+14	########	7.76E+16	4.63E+16
sp|P30536|	6	63	2.678736	2.24E+14	8.37E+13	########	1.35E+15	5.27E+15
sp|P30566|	6	63	12.04011	5.72E+13	4.75E+12	########	3.43E+14	2.99E+14
sp|P30626|	6	63	56.99896	5.29E+17	9.27E+15	########	########	5.84E+17
sp|P30711|	6	63	3.734822	1.53E+15	4.11E+14	########	9.21E+15	3E+16
sp|P30740|	6	63	9.052732	2.04E+14	2.26E+13	########	1.23E+15	1.42E+15
sp|P30837|	6	63	9.441251	1.72E+14	1.82E+13	########	1.03E+15	1E+15
sp|P30876|	6	63	2.322035	5.19E+15	2.24E+15	########	3.12E+16	1E+17
sp|P31040|	6	63	67.41985	6.83E+17	1.01E+16	########	4E+18	6.39E+17
sp|P31146|	6	63	1.111617	4.68E+15	4.21E+15	########	2.81E+16	3E+17
sp|P31150|	6	63	325.193	4E+19	1.13E+17	########	2E+20	7E+18
sp|P31153|	6	63	55.57041	1.04E+16	1.87E+14	########	6.24E+16	1.18E+16
sp|P31323|	6	63	6.272456	9.41E+15	1.5E+15	########	5.65E+16	9.45E+16
sp|P31689|	6	63	18.739	3.42E+15	1.83E+14	########	2.05E+16	1.15E+16
sp|P31930|	6	63	91.14734	4E+18	4.04E+16	########	2E+19	3E+18
sp|P31937|	6	63	55.48728	7.96E+15	1.43E+14	########	4.78E+16	9.04E+15
sp|P31939|	6	63	8.036089	8.17E+15	1.02E+15	########	4.9E+16	6.41E+16
sp|P31942|	6	63	200.9208	8.65E+15	4.3E+13	########	5.19E+16	2.71E+15
sp|P31943|	6	63	118.6982	2.99E+15	2.52E+13	########	1.79E+16	1.59E+15
sp|P31946|	6	63	23.15361	4.26E+17	1.84E+16	########	2.56E+18	1.16E+18
sp|P31947|	6	63	########	7.16E+12	1.91E+13	########	4.3E+13	1.2E+15
sp|P31948|	6	63	110.6856	########	1.46E+15	########	1E+18	9.19E+16
sp|P31949|	6	63	25.08769	6E+15	2.39E+14	########	3.6E+16	2E+16
sp|P32004|	6	63	8.929737	5.55E+16	6.21E+15	########	3.33E+17	3.92E+17
sp|P32119|	6	63	132.7111	4E+18	2.95E+16	########	2E+19	1.86E+18
sp|P32189|	6	63	9.759715	4.98E+13	5.1E+12	########	2.99E+14	3.21E+14
sp|P32418|	6	63	10.32815	2.16E+15	2.1E+14	########	1.3E+16	1.32E+16
sp|P32455|	6	63	1.692062	9.57E+13	5.66E+13	########	5.74E+14	3.56E+15
sp|P32929|	6	63	10.39169	1.1E+13	1.06E+12	########	6.61E+13	6.68E+13
sp|P32969|	6	63	62.5969	6.99E+16	1.12E+15	########	4.19E+17	7.03E+16
sp|P33121|	6	63	3.456326	6.92E+12	2E+12	########	4.15E+13	1.26E+14
sp|P33176|	6	63	########	4.58E+13	7.12E+13	########	2.75E+14	4.49E+15
sp|P33316|	6	63	13.25528	2.6E+14	1.96E+13	########	1.56E+15	1.23E+15
sp|P33402|	6	63	########	6.37E+12	1.13E+13	########	3.82E+13	7.14E+14

For Peer Review Only

sp|P34897|	6	63	4.048004	3.2E+13	7.91E+12	########	1.92E+14	4.98E+14
sp|P34913|	6	63	11.89912	6.19E+14	5.2E+13	########	3.71E+15	3.27E+15
sp|P34932|	6	63	176.2464	6.38E+17	3.62E+15	########	4E+18	2E+17
sp|P34949|	6	63	55.41701	4.11E+16	7.43E+14	########	2.47E+17	4.68E+16
sp|P35080|	6	63	19.30862	1.33E+16	6.9E+14	########	8E+16	4.35E+16
sp|P35221|	6	63	4.442944	2.66E+14	5.99E+13	########	1.6E+15	3.78E+15
sp|P35222|	6	63	66.59042	9.92E+16	1.49E+15	########	5.95E+17	9E+16
sp|P35232|	6	63	187.3921	7.66E+17	4.09E+15	########	4.6E+18	2.58E+17
sp|P35237|	6	63	14.39102	5.43E+14	3.78E+13	########	3.26E+15	2E+15
sp|P35241|	6	63	7.982617	8.13E+15	1.02E+15	########	4.88E+16	6E+16
sp|P35244|	6	63	1.793425	8.79E+13	4.9E+13	########	5.27E+14	3.09E+15
sp|P35268|	6	63	39.6269	1.29E+17	3.26E+15	########	7.76E+17	2E+17
sp|P35270|	6	63	18.54239	5.29E+14	2.85E+13	########	3.18E+15	2E+15
sp|P35498|	6	63	2.040612	4.86E+12	2.38E+12	########	2.92E+13	1.5E+14
sp|P35520|	6	63	26.06475	5.09E+14	1.95E+13	########	3.06E+15	1.23E+15
sp|P35573|	6	63	36.36685	2.2E+16	6.06E+14	########	1E+17	3.82E+16
sp|P35579|	6	63	17.39245	2.92E+17	1.68E+16	########	2E+18	1.06E+18
sp|P35580|	6	63	61.88468	1.52E+17	2.46E+15	########	9.13E+17	1.55E+17
sp|P35606|	6	63	13.42616	2.96E+14	2.21E+13	########	1.78E+15	1.39E+15
sp|P35609|	6	63	10.84926	9.59E+15	8.84E+14	########	5.75E+16	5.57E+16
sp|P35611|	6	63	21.03247	6.94E+16	3.3E+15	########	4.17E+17	2.08E+17
sp|P35612|	6	63	28.98058	4.93E+16	1.7E+15	########	2.96E+17	1.07E+17
sp|P35613|	6	63	73.52494	5.04E+16	6.85E+14	########	3.02E+17	4.32E+16
sp|P35637|	6	63	4.062667	9.35E+13	2.3E+13	########	5.61E+14	1.45E+15
sp|P35749|	6	63	6.823171	3.03E+14	4.44E+13	########	1.82E+15	2.8E+15
sp|P35754|	6	63	72.2591	2.07E+16	2.86E+14	########	1.24E+17	1.8E+16
sp|P35813|	6	63	47.28048	1.8E+15	3.81E+13	########	1.08E+16	2E+15
sp|P35998|	6	63	29.33934	1.48E+15	5.06E+13	########	8.9E+15	3.19E+15
sp|P36222|	6	63	########	5.08E+14	9.42E+14	########	3.05E+15	5.94E+16
sp|P36269|	6	63	11.58633	5.02E+14	4.34E+13	########	3.01E+15	2.73E+15
sp|P36404|	6	63	4.308698	5.41E+13	1.25E+13	########	3.24E+14	7.9E+14
sp|P36405|	6	63	1.32137	3.77E+14	2.86E+14	########	2.26E+15	1.8E+16
sp|P36507|	6	63	18.60943	6.22E+14	3.35E+13	########	3.73E+15	2.11E+15
sp|P36542|	6	63	87.52884	7E+17	7.97E+15	########	4.18E+18	5.02E+17
sp|P36543|	6	63	7.632956	1.68E+17	2.2E+16	########	1.01E+18	1.39E+18
sp|P36578|	6	63	62.63471	4.88E+17	7.79E+15	########	2.93E+18	4.91E+17
sp|P36776|	6	63	126.1192	1.99E+17	1.58E+15	########	1.2E+18	9.96E+16
sp|P36871|	6	63	208.3132	7.51E+17	3.6E+15	########	4.5E+18	########
sp|P36873|	6	63	4.136064	8.94E+13	2.16E+13	########	5.36E+14	1.36E+15
sp|P36915|	6	63	15.7778	4.6E+15	2.92E+14	########	2.76E+16	1.84E+16
sp|P36955|	6	63	1.699834	2.79E+13	1.64E+13	########	1.67E+14	1.03E+15
sp|P36957|	6	63	52.81814	1.09E+17	2.06E+15	########	6.51E+17	1.29E+17
sp|P36959|	6	63	11.9672	2.84E+14	2.38E+13	########	1.71E+15	1.5E+15
sp|P36969|	6	63	16.97414	5.26E+15	3.1E+14	########	3.15E+16	1.95E+16
sp|P37108|	6	63	12.08881	1.83E+15	1.52E+14	########	1.1E+16	9.55E+15
sp|P37235|	6	63	2.30037	4.35E+13	1.89E+13	########	2.61E+14	1.19E+15
sp|P37802|	6	63	43.27829	6.33E+15	1.46E+14	########	3.8E+16	9.21E+15
sp|P37837|	6	63	77.85214	3E+17	4.4E+15	########	2.05E+18	2.77E+17
sp|P37840|	6	63	21.01761	2.35E+17	1.12E+16	########	1E+18	7.05E+17
sp|P38117|	6	63	34.12313	6.38E+15	1.87E+14	########	3.83E+16	1.18E+16
sp|P38159|	6	63	41.69898	4.03E+15	9.65E+13	########	2.42E+16	6.08E+15
sp|P38405|	6	63	1.479077	3.69E+11	2.5E+11	########	2.21E+12	1.57E+13
sp|P38606|	6	63	4.255139	1.25E+18	2.93E+17	########	7E+18	2E+19
sp|P38646|	6	63	64.31829	4.32E+17	6.72E+15	########	3E+18	4.23E+17
sp|P38919|	6	63	21.1076	3.72E+14	1.76E+13	########	2.23E+15	1.11E+15
sp|P39019|	6	63	47.51689	4.96E+16	1.04E+15	########	2.98E+17	6.58E+16

For Peer Review Only

sp|P39023|	6	63	77.30034	2.63E+17	3.4E+15	########	2E+18	2.14E+17
sp|P39060|	6	63	18.60276	1.76E+15	9.48E+13	########	1.06E+16	5.97E+15
sp|P39210|	6	63	26.44018	6E+14	2.27E+13	########	3.6E+15	1.43E+15
sp|P39656|	6	63	21.34768	1.53E+15	7.18E+13	########	9.2E+15	5E+15
sp|P39687|	6	63	45.11622	8.99E+15	1.99E+14	########	5.4E+16	1.26E+16
sp|P40121|	6	63	8.614736	7.83E+15	9.09E+14	########	4.7E+16	5.73E+16
sp|P40123|	6	63	17.76645	1.48E+17	8.35E+15	########	8.91E+17	5E+17
sp|P40145|	6	63	2.130383	1.31E+12	6.16E+11	########	7.88E+12	3.88E+13
sp|P40227|	6	63	47.79652	4.83E+16	1.01E+15	########	2.9E+17	6.37E+16
sp|P40429|	6	63	30.21227	1.88E+16	6.22E+14	########	1.13E+17	4E+16
sp|P40616|	6	63	13.94137	4.55E+13	3.26E+12	########	2.73E+14	2.06E+14
sp|P40763|	6	63	14.9103	1.11E+14	7.46E+12	########	6.67E+14	4.7E+14
sp|P40855|	6	63	1.284382	9.22E+12	7.18E+12	########	5.53E+13	4.52E+14
sp|P40925|	6	63	142.8969	3E+19	########	########	2E+20	1E+19
sp|P40926|	6	63	22.99598	1E+19	4.67E+17	########	6E+19	3E+19
sp|P40939|	6	63	33.96664	7.31E+17	2.15E+16	########	4E+18	1.36E+18
sp|P41091|	6	63	32.89588	1.9E+15	5.76E+13	########	1.14E+16	3.63E+15
sp|P41208|	6	63	5.133285	4.97E+13	9.69E+12	########	2.98E+14	6.1E+14
sp|P41217|	6	63	5.178056	1.49E+15	2.89E+14	########	8.97E+15	2E+16
sp|P41222|	6	63	26.3303	5.27E+16	2E+15	########	3.16E+17	1.26E+17
sp|P41240|	6	63	1.250025	9.92E+12	7.94E+12	########	5.95E+13	5E+14
sp|P41250|	6	63	59.32046	4.57E+16	7.7E+14	########	2.74E+17	4.85E+16
sp|P41252|	6	63	12.22039	1.18E+15	9.68E+13	########	7.1E+15	6E+15
sp|P41567|	6	63	9.227767	6.26E+14	6.78E+13	########	3.75E+15	4E+15
sp|P41586|	6	63	6.679016	5.46E+13	8.18E+12	########	3.28E+14	5.15E+14
sp|P41732|	6	63	2.509422	4.17E+13	1.66E+13	########	2.5E+14	1.05E+15
sp|P42025|	6	63	4.759124	2.24E+15	4.72E+14	########	1.35E+16	2.97E+16
sp|P42126|	6	63	69.26189	1.68E+15	2.43E+13	########	1.01E+16	1.53E+15
sp|P42167|	6	63	21.15393	1.92E+15	9.1E+13	########	1.15E+16	5.73E+15
sp|P42224|	6	63	8.052808	1.15E+15	1.43E+14	########	6.9E+15	9E+15
sp|P42262|	6	63	41.16071	1.93E+16	4.68E+14	########	1.16E+17	2.95E+16
sp|P42263|	6	63	14.70034	2.67E+15	1.81E+14	########	1.6E+16	1.14E+16
sp|P42330|	6	63	2.171924	2.78E+14	1.28E+14	########	1.67E+15	8.08E+15
sp|P42345|	6	63	22.28975	8.3E+14	3.72E+13	########	4.98E+15	2.35E+15
sp|P42356|	6	63	45.69618	5.03E+15	1.1E+14	########	3.02E+16	6.93E+15
sp|P42566|	6	63	2.643992	3.62E+13	1.37E+13	########	2.17E+14	8.63E+14
sp|P42658|	6	63	12.44342	8.16E+16	6.56E+15	########	4.89E+17	4.13E+17
sp|P42677|	6	63	33.16817	1.55E+16	4.67E+14	########	9.29E+16	2.94E+16
sp|P42704|	6	63	52.88637	2.04E+17	3.86E+15	########	1.23E+18	2.43E+17
sp|P42765|	6	63	28.29325	1.06E+16	3.73E+14	########	6.33E+16	2.35E+16
sp|P42766|	6	63	58.1314	1.88E+16	3.23E+14	########	1.13E+17	2.03E+16
sp|P42785|	6	63	33.25573	3.15E+15	9.49E+13	########	1.89E+16	5.98E+15
sp|P42858|	6	63	11.5069	1.06E+15	9.23E+13	########	6.38E+15	5.82E+15
sp|P43003|	6	63	35.51173	8E+18	########	########	5E+19	1E+19
sp|P43004|	6	63	31.02049	2E+19	########	########	1E+20	4E+19
sp|P43007|	6	63	39.35482	2.71E+16	6.89E+14	########	1.63E+17	4.34E+16
sp|P43034|	6	63	1.571161	9.55E+14	6.08E+14	########	5.73E+15	3.83E+16
sp|P43121|	6	63	30.87448	3.61E+15	1.17E+14	########	2.16E+16	7.36E+15
sp|P43155|	6	63	22.49923	1.86E+15	8.27E+13	########	1.12E+16	5E+15
sp|P43243|	6	63	91.76594	2.48E+17	2.7E+15	########	1.49E+18	1.7E+17
sp|P43304|	6	63	125.3606	1.74E+17	1.39E+15	########	1.04E+18	8.73E+16
sp|P43307|	6	63	1.659792	1.42E+13	8.53E+12	########	8.49E+13	5.37E+14
sp|P43487|	6	63	3.256445	7.49E+14	2.3E+14	########	4.49E+15	1E+16
sp|P43490|	6	63	2.324684	4.27E+15	1.84E+15	########	2.56E+16	1E+17
sp|P43686|	6	63	41.27124	6.78E+15	1.64E+14	########	4.07E+16	1.04E+16
sp|P43897|	6	63	51.18995	3.5E+15	6.84E+13	########	2.1E+16	4.31E+15

For Peer Review Only

sp|P45381|	6	63	16.07232	2.62E+16	1.63E+15	########	########	1.03E+17
sp|P45880|	6	63	37.91867	8.92E+17	2.35E+16	########	5E+18	1.48E+18
sp|P45954|	6	63	9.129441	3.12E+14	3.42E+13	########	1.87E+15	2.16E+15
sp|P45974|	6	63	116.0668	3.21E+17	2.77E+15	########	########	1.74E+17
sp|P45985|	6	63	23.53712	4.61E+15	1.96E+14	########	2.76E+16	1E+16
sp|P46060|	6	63	13.69404	3.57E+14	2.61E+13	########	2.14E+15	1.64E+15
sp|P46108|	6	63	5.606771	2.59E+14	4.62E+13	########	1.55E+15	3E+15
sp|P46109|	6	63	7.358789	1.18E+15	1.6E+14	########	7.06E+15	1.01E+16
sp|P46379|	6	63	20.00894	5.36E+14	2.68E+13	########	3.21E+15	2E+15
sp|P46439|	6	63	1.745903	3.45E+13	1.97E+13	########	2.07E+14	1.24E+15
sp|P46459|	6	63	78.90535	6E+18	7.25E+16	########	3E+19	4.57E+18
sp|P46776|	6	63	36.92561	8.11E+16	2.2E+15	########	4.86E+17	1.38E+17
sp|P46777|	6	63	55.81548	9.41E+16	1.69E+15	########	5.65E+17	1.06E+17
sp|P46778|	6	63	30.88363	4.11E+16	1.33E+15	########	2.47E+17	8.39E+16
sp|P46779|	6	63	38.2303	4.07E+15	1.07E+14	########	2.44E+16	6.71E+15
sp|P46781|	6	63	52.04568	7.94E+16	1.53E+15	########	4.77E+17	9.62E+16
sp|P46782|	6	63	67.43212	1.1E+17	1.64E+15	########	6.63E+17	1.03E+17
sp|P46783|	6	63	37.54288	7.32E+16	1.95E+15	########	4.39E+17	1.23E+17
sp|P46821|	6	63	18.82701	5E+18	2.87E+17	########	3E+19	2E+19
sp|P46926|	6	63	158.2561	6.39E+16	4.04E+14	########	3.83E+17	2.54E+16
sp|P46939|	6	63	2.462622	6.08E+13	2.47E+13	########	3.65E+14	1.55E+15
sp|P46940|	6	63	3.532616	7.57E+14	2.14E+14	########	4.54E+15	1E+16
sp|P46952|	6	63	4.879868	4.45E+17	9.12E+16	########	3E+18	6E+18
sp|P46976|	6	63	33.99781	2E+15	5.88E+13	########	1.2E+16	3.71E+15
sp|P47736|	6	63	35.29801	2.35E+15	6.65E+13	########	1.41E+16	4.19E+15
sp|P47755|	6	63	15.57196	1.24E+16	7.93E+14	########	7.41E+16	5E+16
sp|P47756|	6	63	5.513623	4.62E+15	8.37E+14	########	2.77E+16	5.27E+16
sp|P47870|	6	63	13.13369	2.45E+15	1.87E+14	########	1.47E+16	1E+16
sp|P47897|	6	63	13.20789	1.54E+14	1.16E+13	########	9.22E+14	7.33E+14
sp|P47914|	6	63	20.18423	3.01E+15	1.49E+14	########	1.8E+16	9E+15
sp|P47985|	6	63	62.05826	4.99E+17	8.05E+15	########	3E+18	5.07E+17
sp|P48047|	6	63	114.5641	1E+18	1.29E+16	########	9E+18	8.12E+17
sp|P48066|	6	63	9.213385	9.16E+14	9.94E+13	########	5.5E+15	6.26E+15
sp|P48147|	6	63	5.180025	1.17E+16	2.27E+15	########	7.05E+16	1E+17
sp|P48163|	6	63	92.4954	4.15E+16	4.49E+14	########	2.49E+17	2.83E+16
sp|P48426|	6	63	1.675308	1.88E+15	1.12E+15	########	1.13E+16	7.07E+16
sp|P48444|	6	63	5.911731	2.16E+14	3.65E+13	########	1.29E+15	2.3E+15
sp|P48506|	6	63	########	7.59E+13	1.11E+14	########	4.55E+14	7E+15
sp|P48539|	6	63	26.29333	1.55E+15	5.91E+13	########	9.32E+15	3.72E+15
sp|P48553|	6	63	1.46233	2.02E+12	1.38E+12	########	1.21E+13	8.7E+13
sp|P48556|	6	63	13.13016	2.75E+14	2.1E+13	########	1.65E+15	1.32E+15
sp|P48637|	6	63	56.43471	1.32E+16	2.33E+14	########	7.9E+16	1.47E+16
sp|P48643|	6	63	20.43459	7.44E+16	3.64E+15	########	4.47E+17	2.29E+17
sp|P48723|	6	63	5.309069	3.74E+13	7.05E+12	########	2.25E+14	4.44E+14
sp|P48735|	6	63	41.76808	7.1E+17	1.7E+16	########	4.26E+18	1.07E+18
sp|P48739|	6	63	4.344729	1.1E+14	2.53E+13	########	6.61E+14	1.6E+15
sp|P49006|	6	63	6.585344	4.13E+14	6.26E+13	########	2.48E+15	3.95E+15
sp|P49189|	6	63	77.40621	3.2E+17	4.13E+15	########	1.92E+18	3E+17
sp|P49207|	6	63	24.43975	5.09E+15	2.08E+14	########	3.05E+16	1.31E+16
sp|P49257|	6	63	2.824437	7.41E+13	2.62E+13	########	4.45E+14	1.65E+15
sp|P49327|	6	63	5.688965	1.03E+17	1.81E+16	########	6.17E+17	1.14E+18
sp|P49354|	6	63	2.998386	7.05E+13	2.35E+13	########	4.23E+14	1E+15
sp|P49368|	6	63	21.58535	5.45E+16	2.53E+15	########	3.27E+17	1.59E+17
sp|P49407|	6	63	35.66104	7.05E+15	1.98E+14	########	4.23E+16	1.25E+16
sp|P49411|	6	63	67.49193	1.67E+18	2.48E+16	########	1E+19	2E+18
sp|P49418|	6	63	3.957509	2.92E+16	7.38E+15	########	1.75E+17	4.65E+17

For Peer Review Only

sp|P49419|	6	63	111.8279	1.06E+18	9.44E+15	########	6E+18	5.95E+17
sp|P49441|	6	63	16.65694	2.6E+14	1.56E+13	########	1.56E+15	9.85E+14
sp|P49588|	6	63	35.59258	7.9E+16	2.22E+15	########	4.74E+17	1.4E+17
sp|P49589|	6	63	68.77234	3.97E+15	5.78E+13	########	2.38E+16	3.64E+15
sp|P49591|	6	63	170.2213	7.85E+16	4.61E+14	########	4.71E+17	2.9E+16
sp|P49593|	6	63	3.256858	1.36E+14	4.17E+13	########	8.14E+14	2.63E+15
sp|P49720|	6	63	70.93551	1.07E+16	1.51E+14	########	6.44E+16	9.53E+15
sp|P49721|	6	63	82.18813	6.3E+15	7.67E+13	########	3.78E+16	4.83E+15
sp|P49748|	6	63	39.25688	2.22E+16	5.66E+14	########	1.33E+17	3.57E+16
sp|P49750|	6	63	13.53535	6.86E+12	5.07E+11	########	4.12E+13	3.19E+13
sp|P49755|	6	63	16.45476	1.6E+15	9.74E+13	########	9.61E+15	6.13E+15
sp|P49758|	6	63	6.836952	2.2E+14	3.22E+13	########	1.32E+15	2.03E+15
sp|P49773|	6	63	83.31733	1.34E+18	1.6E+16	########	8E+18	1.01E+18
sp|P49802|	6	63	18.45105	7.1E+15	3.85E+14	########	4.26E+16	2.43E+16
sp|P49815|	6	63	28.22333	1.08E+14	3.84E+12	########	6.5E+14	2.42E+14
sp|P49821|	6	63	71.90741	7.93E+17	1.1E+16	########	5E+18	6.95E+17
sp|P49840|	6	63	2.014696	6.97E+13	3.46E+13	########	4.18E+14	2.18E+15
sp|P49841|	6	63	12.75433	8.73E+14	6.84E+13	########	5.24E+15	4.31E+15
sp|P49902|	6	63	33.75097	5.07E+14	1.5E+13	########	3E+15	9.46E+14
sp|P49915|	6	63	7.048516	1.21E+15	1.72E+14	########	7.26E+15	1E+16
sp|P50135|	6	63	23.24336	5.28E+14	2.27E+13	########	3.17E+15	1.43E+15
sp|P50148|	6	63	49.13771	########	3.42E+15	########	1.01E+18	2E+17
sp|P50150|	6	63	4.861176	3.18E+14	6.55E+13	########	1.91E+15	4.12E+15
sp|P50151|	6	63	11.51637	1.97E+15	1.71E+14	########	1.18E+16	1E+16
sp|P50213|	6	63	53.0534	7.43E+17	1.4E+16	########	4E+18	8.82E+17
sp|P50226|	6	63	1.320493	8.89E+11	6.73E+11	########	5.33E+12	4.24E+13
sp|P50336|	6	63	26.47416	2.15E+14	8.11E+12	########	1.29E+15	5.11E+14
sp|P50395|	6	63	108.3536	7.8E+17	7.2E+15	########	5E+18	4.54E+17
sp|P50402|	6	63	3.403089	6.69E+12	1.97E+12	########	4.01E+13	1.24E+14
sp|P50416|	6	63	10.9092	1.95E+14	1.79E+13	########	1.17E+15	1.13E+15
sp|P50440|	6	63	28.13552	7E+15	2.35E+14	########	4E+16	1.48E+16
sp|P50453|	6	63	30.55624	9.82E+14	3.21E+13	########	5.89E+15	2.03E+15
sp|P50454|	6	63	9.401095	1.96E+14	2.09E+13	########	1.18E+15	1E+15
sp|P50502|	6	63	75.83951	5.71E+16	7.52E+14	########	3.42E+17	4.74E+16
sp|P50570|	6	63	24.59513	1.43E+16	5.82E+14	########	8.58E+16	3.66E+16
sp|P50579|	6	63	14.94149	2.73E+13	1.83E+12	########	1.64E+14	1.15E+14
sp|P50583|	6	63	########	9.78E+12	2.2E+13	########	5.87E+13	1.38E+15
sp|P50897|	6	63	29.47795	1.54E+16	5.24E+14	########	9.26E+16	3.3E+16
sp|P50914|	6	63	47.54633	4.16E+16	8.75E+14	########	2.5E+17	5.51E+16
sp|P50990|	6	63	13.24841	1.19E+16	9E+14	########	7.16E+16	5.67E+16
sp|P50991|	6	63	35.38927	6.12E+16	1.73E+15	########	3.67E+17	1.09E+17
sp|P50993|	6	63	45.65812	1E+20	3E+18	########	8E+20	2E+20
sp|P50995|	6	63	35.21071	########	3.93E+15	########	8.3E+17	2.48E+17
sp|P51114|	6	63	15.42902	2.16E+14	1.4E+13	########	1.3E+15	8.84E+14
sp|P51148|	6	63	4.728552	2.54E+15	5.36E+14	########	1.52E+16	3E+16
sp|P51149|	6	63	6.464607	8.28E+15	1.28E+15	########	4.97E+16	8E+16
sp|P51178|	6	63	10.85294	8.54E+15	7.87E+14	########	5.12E+16	4.96E+16
sp|P51398|	6	63	2.3397	3.84E+12	1.64E+12	########	2.3E+13	1.03E+14
sp|P51452|	6	63	3.016748	8.04E+14	2.67E+14	########	4.83E+15	1.68E+16
sp|P51513|	6	63	4.738802	1.47E+14	3.11E+13	########	8.83E+14	1.96E+15
sp|P51553|	6	63	50.37839	2.23E+17	4.42E+15	########	1.34E+18	2.78E+17
sp|P51570|	6	63	3.716442	1.84E+14	4.94E+13	########	1.1E+15	3.11E+15
sp|P51571|	6	63	5.466434	8.13E+13	1.49E+13	########	4.88E+14	9.37E+14
sp|P51572|	6	63	14.37476	1.8E+15	1.25E+14	########	1.08E+16	7.87E+15
sp|P51608|	6	63	38.15611	1.9E+15	4.99E+13	########	1.14E+16	3.14E+15
sp|P51610|	6	63	2.588863	4.1E+12	1.58E+12	########	2.46E+13	9.97E+13

For Peer Review Only

sp|P51648|	6	63	19.93646	9.72E+14	4.87E+13	########	5.83E+15	3.07E+15
sp|P51649|	6	63	37.4071	1.95E+17	5.21E+15	########	1.17E+18	3.28E+17
sp|P51659|	6	63	84.54225	5.95E+16	7.04E+14	########	########	4.43E+16
sp|P51665|	6	63	14.97174	1.23E+15	8.18E+13	########	7.35E+15	5.16E+15
sp|P51674|	6	63	29.76913	1.46E+18	4.91E+16	########	9E+18	3.09E+18
sp|P51693|	6	63	65.18997	8.65E+14	1.33E+13	########	5.19E+15	8.36E+14
sp|P51784|	6	63	9.597973	4.84E+13	5.04E+12	########	2.9E+14	3.17E+14
sp|P51790|	6	63	1.990933	2.04E+12	1.03E+12	########	1.22E+13	6.46E+13
sp|P51808|	6	63	22.13092	1.18E+15	5.33E+13	########	7.07E+15	3.36E+15
sp|P51858|	6	63	27.59461	3.7E+15	1.34E+14	########	2.22E+16	8.44E+15
sp|P51888|	6	63	3.298835	9.19E+14	2.78E+14	########	5.51E+15	1.75E+16
sp|P51911|	6	63	1.124428	2.8E+13	2.49E+13	########	1.68E+14	1.57E+15
sp|P51970|	6	63	52.37738	1.44E+17	2.76E+15	########	8.66E+17	1.74E+17
sp|P51991|	6	63	71.70437	1.4E+17	1.95E+15	########	8.38E+17	1.23E+17
sp|P52209|	6	63	48.8565	3.55E+17	7.27E+15	########	2.13E+18	########
sp|P52272|	6	63	58.91025	6.58E+16	1.12E+15	########	3.95E+17	7.04E+16
sp|P52294|	6	63	54.78571	3.03E+14	5.53E+12	########	1.82E+15	3.48E+14
sp|P52306|	6	63	4.218476	3.77E+16	8.93E+15	########	2.26E+17	5.63E+17
sp|P52429|	6	63	4.412971	1.79E+13	4.06E+12	########	1.07E+14	2.56E+14
sp|P52565|	6	63	43.57755	3.29E+17	7.54E+15	########	2E+18	4.75E+17
sp|P52566|	6	63	15.44397	2.91E+15	1.89E+14	########	1.75E+16	1.19E+16
sp|P52594|	6	63	2.987518	4.75E+13	1.59E+13	########	2.85E+14	1E+15
sp|P52597|	6	63	18.91168	2.23E+14	1.18E+13	########	1.34E+15	7.41E+14
sp|P52758|	6	63	115.3546	4.99E+16	4.32E+14	########	3E+17	3E+16
sp|P52788|	6	63	4.51618	5.22E+14	1.16E+14	########	3.13E+15	7.29E+15
sp|P52815|	6	63	14.31762	4.33E+15	3.02E+14	########	2.6E+16	1.9E+16
sp|P52888|	6	63	30.49071	2.29E+14	7.49E+12	########	1E+15	4.72E+14
sp|P52907|	6	63	7.377683	1.02E+15	1.38E+14	########	6.13E+15	8.72E+15
sp|P52943|	6	63	2.02633	1.99E+15	9.8E+14	########	1.19E+16	6.17E+16
sp|P53004|	6	63	211.1131	1.34E+16	6.33E+13	########	8.02E+16	3.99E+15
sp|P53007|	6	63	20.95399	1.19E+15	5.69E+13	########	7.15E+15	3.58E+15
sp|P53041|	6	63	22.40476	2.59E+15	1.15E+14	########	1.55E+16	7.28E+15
sp|P53365|	6	63	3.833572	3.48E+13	9.07E+12	########	2.09E+14	5.71E+14
sp|P53367|	6	63	########	4.66E+12	5.75E+12	########	2.8E+13	3.62E+14
sp|P53396|	6	63	3.297374	4.14E+15	1.26E+15	########	2.49E+16	7.92E+16
sp|P53582|	6	63	3.099924	6.55E+14	2.11E+14	########	3.93E+15	1.33E+16
sp|P53597|	6	63	94.35046	4.72E+16	5.01E+14	########	2.83E+17	3.15E+16
sp|P53602|	6	63	########	8.5E+13	1.34E+14	########	5.1E+14	8.44E+15
sp|P53618|	6	63	25.01999	1.47E+15	5.89E+13	########	8.84E+15	3.71E+15
sp|P53621|	6	63	40.00005	2.28E+15	5.71E+13	########	1.37E+16	3.6E+15
sp|P53677|	6	63	12.8756	2.39E+15	1.86E+14	########	1.43E+16	1E+16
sp|P53680|	6	63	15.16234	1.21E+16	8E+14	########	7.28E+16	5.04E+16
sp|P53779|	6	63	49.6916	9.01E+15	1.81E+14	########	5.41E+16	1.14E+16
sp|P53985|	6	63	18.55087	5.07E+14	2.73E+13	########	3.04E+15	1.72E+15
sp|P53990|	6	63	4.123924	6.29E+14	1.52E+14	########	3.77E+15	9.6E+15
sp|P53992|	6	63	9.044314	9.56E+13	1.06E+13	########	5.74E+14	6.66E+14
sp|P53999|	6	63	12.82045	2.1E+15	1.64E+14	########	1.26E+16	1E+16
sp|P54136|	6	63	6.71787	1.36E+15	2.03E+14	########	8.17E+15	1.28E+16
sp|P54284|	6	63	3.639184	9.81E+13	2.7E+13	########	5.89E+14	1.7E+15
sp|P54289|	6	63	17.04605	3.1E+16	1.82E+15	########	1.86E+17	1.15E+17
sp|P54577|	6	63	########	1.73E+14	1.88E+14	########	1.04E+15	1.19E+16
sp|P54578|	6	63	########	7.15E+14	7.89E+14	########	4.29E+15	4.97E+16
sp|P54619|	6	63	33.81276	3E+14	8.88E+12	########	1.8E+15	5.59E+14
sp|P54652|	6	63	3.460959	4.72E+16	1.36E+16	########	2.83E+17	8.6E+17
sp|P54687|	6	63	23.21866	1.1E+15	4.75E+13	########	6.61E+15	2.99E+15
sp|P54709|	6	63	60.52261	1.01E+16	1.67E+14	########	6.08E+16	1.05E+16

For Peer Review Only

sp|P54725|	6	63	28.44883	3.48E+14	1.22E+13	########	2.09E+15	7.7E+14
sp|P54727|	6	63	104.5129	1.86E+16	1.78E+14	########	1.11E+17	1E+16
sp|P54750|	6	63	2.940524	1.57E+14	5.33E+13	########	9.41E+14	3.36E+15
sp|P54764|	6	63	6.278174	8.26E+14	1.32E+14	########	4.96E+15	8.29E+15
sp|P54803|	6	63	########	2.22E+13	3.05E+13	########	1.33E+14	1.92E+15
sp|P54819|	6	63	34.39482	5.71E+14	1.66E+13	########	3.42E+15	1.05E+15
sp|P54829|	6	63	2.031985	6.9E+12	3.4E+12	########	4.14E+13	2.14E+14
sp|P54886|	6	63	7.651981	3.06E+12	4E+11	########	1.84E+13	2.52E+13
sp|P54920|	6	63	24.82794	1.47E+17	5.91E+15	########	8.8E+17	3.72E+17
sp|P55010|	6	63	13.08381	5.91E+14	4.51E+13	########	3.54E+15	2.84E+15
sp|P55011|	6	63	12.00432	4.64E+15	3.86E+14	########	2.78E+16	2.43E+16
sp|P55036|	6	63	13.74512	2.95E+14	2.15E+13	########	1.77E+15	1E+15
sp|P55060|	6	63	30.50502	3.53E+16	1.16E+15	########	2.12E+17	7.3E+16
sp|P55072|	6	63	54.35732	1.15E+18	2.11E+16	########	7E+18	1E+18
sp|P55084|	6	63	37.37436	########	5.32E+15	########	1.19E+18	3E+17
sp|P55087|	6	63	7.286648	4.45E+17	6.11E+16	########	2.67E+18	3.85E+18
sp|P55160|	6	63	3.923312	5.08E+11	1.3E+11	########	3.05E+12	8.16E+12
sp|P55196|	6	63	4.477474	3.34E+14	7.46E+13	########	2E+15	5E+15
sp|P55209|	6	63	2.570827	4.07E+15	1.59E+15	########	2.44E+16	9.99E+16
sp|P55263|	6	63	26.21171	1.2E+14	4.58E+12	########	7.2E+14	2.88E+14
sp|P55268|	6	63	43.48531	1.9E+16	4.37E+14	########	########	2.76E+16
sp|P55290|	6	63	7.681822	5.88E+15	7.65E+14	########	3.53E+16	4.82E+16
sp|P55327|	6	63	39.9206	1.87E+16	4.69E+14	########	1.12E+17	2.95E+16
sp|P55735|	6	63	7.007385	4.37E+14	6.24E+13	########	2.62E+15	3.93E+15
sp|P55769|	6	63	29.5809	1.84E+14	6.23E+12	########	1.11E+15	3.92E+14
sp|P55786|	6	63	176.464	2E+18	9.84E+15	########	1E+19	6E+17
sp|P55795|	6	63	106.6923	3.77E+16	3.54E+14	########	2.26E+17	2.23E+16
sp|P55809|	6	63	75.55273	3.59E+17	4.75E+15	########	2.15E+18	2.99E+17
sp|P55854|	6	63	4.156379	3.25E+14	7.81E+13	########	1.95E+15	4.92E+15
sp|P55884|	6	63	7.657002	1.24E+15	1.62E+14	########	7.44E+15	1E+16
sp|P55957|	6	63	25.59883	1.84E+14	7.2E+12	########	1.11E+15	4.54E+14
sp|P56134|	6	63	40.31255	1.8E+17	4.46E+15	########	1.08E+18	2.81E+17
sp|P56181|	6	63	14.5773	4.74E+14	3.25E+13	########	2.84E+15	2.05E+15
sp|P56192|	6	63	34.39235	7.46E+15	2.17E+14	########	4.48E+16	1.37E+16
sp|P56378|	6	63	9.661338	1.2E+15	1.24E+14	########	7.2E+15	7.83E+15
sp|P56385|	6	63	45.38472	4.03E+16	8.87E+14	########	2.42E+17	5.59E+16
sp|P56537|	6	63	2.904147	7.99E+13	2.75E+13	########	4.79E+14	1.73E+15
sp|P56556|	6	63	58.06255	1.72E+16	2.96E+14	########	1.03E+17	1.86E+16
sp|P57087|	6	63	5.691677	5.2E+13	9.14E+12	########	3.12E+14	5.76E+14
sp|P57088|	6	63	2.442512	1.17E+14	4.79E+13	########	7.02E+14	3.02E+15
sp|P57737|	6	63	12.6698	1.4E+14	1.11E+13	########	8.42E+14	6.98E+14
sp|P58546|	6	63	56.14577	5.58E+16	9.95E+14	########	3.35E+17	6E+16
sp|P58549|	6	63	23.38287	9.62E+15	4.11E+14	########	5.77E+16	2.59E+16
sp|P59666|	6	63	########	5.55E+14	9.87E+14	########	3.33E+15	6.22E+16
sp|P59768|	6	63	11.20617	3.77E+16	3.37E+15	########	2.26E+17	2.12E+17
sp|P59998|	6	63	9.251573	8.8E+14	9.52E+13	########	5.28E+15	5.99E+15
sp|P60033|	6	63	38.7902	5E+18	1.42E+17	########	3E+19	9E+18
sp|P60174|	6	63	282.3153	9E+19	3.23E+17	########	5E+20	2E+19
sp|P60201|	6	63	13.1085	4E+20	3E+19	########	2E+21	2E+21
sp|P60228|	6	63	9.880273	3.02E+14	3.06E+13	########	1.81E+15	1.93E+15
sp|P60520|	6	63	30.21671	9.99E+14	3.31E+13	########	5.99E+15	2.08E+15
sp|P60660|	6	63	19.37759	1.57E+16	8.11E+14	########	9.43E+16	5.11E+16
sp|P60709|	6	63	########	1.26E+16	1.38E+16	########	7.58E+16	8.72E+17
sp|P60842|	6	63	10.61786	4.3E+14	4.05E+13	########	2.58E+15	3E+15
sp|P60866|	6	63	48.13931	3.26E+16	6.77E+14	########	1.95E+17	4.26E+16
sp|P60880|	6	63	20.97884	3.28E+18	1.56E+17	########	2E+19	1E+19

For Peer Review Only

sp|P60891|	6	63	35.93893	2.14E+16	5.96E+14	########	1.29E+17	3.76E+16
sp|P60900|	6	63	81.3824	2.12E+16	2.61E+14	########	1.27E+17	1.64E+16
sp|P60903|	6	63	6.725803	1.57E+15	2.33E+14	########	9.41E+15	1.47E+16
sp|P60953|	6	63	26.19952	5.76E+16	2.2E+15	########	########	1.38E+17
sp|P60981|	6	63	9.336398	1.42E+16	1.52E+15	########	8.54E+16	9.6E+16
sp|P60983|	6	63	111.1357	1.73E+17	1.56E+15	########	1.04E+18	9.81E+16
sp|P61006|	6	63	1.655179	4.13E+13	2.49E+13	########	2.48E+14	1.57E+15
sp|P61011|	6	63	4.113909	6.03E+13	1.47E+13	########	3.62E+14	9.24E+14
sp|P61018|	6	63	59.38206	1.68E+15	2.83E+13	########	1.01E+16	1.78E+15
sp|P61019|	6	63	########	1.69E+15	2.09E+15	########	1.01E+16	1.32E+17
sp|P61020|	6	63	3.43289	4.97E+14	1.45E+14	########	2.98E+15	9.12E+15
sp|P61026|	6	63	4.658641	2.71E+15	5.81E+14	########	1.62E+16	3.66E+16
sp|P61077|	6	63	18.80412	1.99E+15	1.06E+14	########	1.2E+16	6.68E+15
sp|P61081|	6	63	26.1933	4.73E+15	1.81E+14	########	2.84E+16	1E+16
sp|P61086|	6	63	68.26448	7.69E+14	1.13E+13	########	4.61E+15	7.1E+14
sp|P61088|	6	63	47.62996	7.8E+16	1.64E+15	########	4.68E+17	1.03E+17
sp|P61106|	6	63	25.38419	1.32E+16	5.21E+14	########	7.94E+16	3.28E+16
sp|P61158|	6	63	7.348656	5.91E+15	8.04E+14	########	3.55E+16	5.07E+16
sp|P61160|	6	63	4.811139	1E+15	2.24E+14	########	6E+15	1.41E+16
sp|P61163|	6	63	12.18778	6.01E+16	4.93E+15	########	3.6E+17	3.1E+17
sp|P61201|	6	63	2.737108	6.85E+14	2.5E+14	########	4.11E+15	1.58E+16
sp|P61204|	6	63	1.345543	1.11E+13	8.26E+12	########	6.67E+13	5.2E+14
sp|P61221|	6	63	8.309765	3.57E+14	4.29E+13	########	2.14E+15	2.7E+15
sp|P61224|	6	63	15.59916	3.92E+15	2.52E+14	########	2.35E+16	1.58E+16
sp|P61225|	6	63	4.243681	1.44E+14	3.39E+13	########	8.63E+14	2.14E+15
sp|P61247|	6	63	62.54606	2.43E+17	3.89E+15	########	1.46E+18	2.45E+17
sp|P61254|	6	63	51.4321	3E+16	5.58E+14	########	2E+17	3.52E+16
sp|P61266|	6	63	13.50456	1.69E+18	1.25E+17	########	1E+19	8E+18
sp|P61313|	6	63	31.54439	2.4E+16	7.6E+14	########	1.44E+17	4.79E+16
sp|P61328|	6	63	7.66772	4.55E+14	5.93E+13	########	2.73E+15	3.74E+15
sp|P61353|	6	63	59.90373	7.13E+16	1.19E+15	########	4.28E+17	7.5E+16
sp|P61421|	6	63	13.73878	6.06E+17	4.41E+16	########	4E+18	3E+18
sp|P61457|	6	63	3.457592	1.24E+13	3.59E+12	########	7.45E+13	2.26E+14
sp|P61513|	6	63	11.43877	1.15E+14	1.01E+13	########	6.9E+14	6.33E+14
sp|P61586|	6	63	20.29085	1.37E+17	6.77E+15	########	8E+17	4.26E+17
sp|P61601|	6	63	6.152235	2.92E+15	4.74E+14	########	1.75E+16	2.99E+16
sp|P61604|	6	63	26.68514	2.37E+17	8.89E+15	########	1E+18	5.6E+17
sp|P61619|	6	63	2.872311	4.45E+13	1.55E+13	########	2.67E+14	9.76E+14
sp|P61758|	6	63	20.45031	2.85E+15	1.39E+14	########	1.71E+16	8.79E+15
sp|P61764|	6	63	21.45275	7E+19	3.25E+18	########	4E+20	2E+20
sp|P61769|	6	63	5.846434	2.56E+15	4.37E+14	########	1.53E+16	2.76E+16
sp|P61916|	6	63	48.23843	2.64E+15	5.47E+13	########	1.58E+16	3E+15
sp|P61923|	6	63	4.123827	5.17E+13	1.25E+13	########	3.1E+14	7.9E+14
sp|P61925|	6	63	43.19183	8.03E+13	1.86E+12	########	4.82E+14	1.17E+14
sp|P61960|	6	63	225.4112	2.15E+16	9.55E+13	########	1.29E+17	6.02E+15
sp|P61970|	6	63	190.6043	1.41E+17	7.38E+14	########	8.44E+17	4.65E+16
sp|P61978|	6	63	44.99399	1.7E+17	3.78E+15	########	1.02E+18	2.38E+17
sp|P61981|	6	63	19.64127	4.23E+18	########	########	3E+19	1E+19
sp|P62068|	6	63	3.185909	7.75E+12	2.43E+12	########	4.65E+13	1.53E+14
sp|P62070|	6	63	1.700194	4.67E+11	2.75E+11	########	2.8E+12	1.73E+13
sp|P62081|	6	63	89.84037	3.7E+17	4.12E+15	########	2E+18	2.6E+17
sp|P62140|	6	63	8.029013	8.14E+15	1.01E+15	########	4.89E+16	6.39E+16
sp|P62166|	6	63	7.855326	3.16E+14	4.02E+13	########	1.9E+15	2.53E+15
sp|P62191|	6	63	12.0846	6.36E+14	5.26E+13	########	3.81E+15	3.31E+15
sp|P62195|	6	63	30.31113	3.63E+15	1.2E+14	########	2.18E+16	7.54E+15
sp|P62241|	6	63	39.12423	1.45E+17	3.72E+15	########	8.73E+17	2E+17

For Peer Review Only

sp|P62244|	6	63	49.31237	4E+16	8.11E+14	########	2.4E+17	5.11E+16
sp|P62249|	6	63	61.5278	1.43E+17	2.32E+15	########	9E+17	1.46E+17
sp|P62258|	6	63	8.682977	2E+18	2.11E+17	########	1E+19	1E+19
sp|P62263|	6	63	59.89286	4.4E+16	7.34E+14	########	2.64E+17	4.63E+16
sp|P62266|	6	63	71.037	2.72E+16	3.83E+14	########	1.63E+17	2E+16
sp|P62269|	6	63	55.83886	4.75E+16	8.5E+14	########	2.85E+17	5.36E+16
sp|P62273|	6	63	17.52523	3.48E+15	1.99E+14	########	2.09E+16	1.25E+16
sp|P62277|	6	63	53.03367	1.6E+17	3.01E+15	########	9.58E+17	2E+17
sp|P62280|	6	63	42.97828	8.02E+16	1.87E+15	########	4.81E+17	1.18E+17
sp|P62304|	6	63	24.79103	3.17E+15	1.28E+14	########	1.9E+16	8.06E+15
sp|P62306|	6	63	2.952579	6.46E+12	2.19E+12	########	3.87E+13	1.38E+14
sp|P62308|	6	63	3.892813	4.35E+14	1.12E+14	########	2.61E+15	7.04E+15
sp|P62310|	6	63	8.05971	6.93E+13	8.6E+12	########	4.16E+14	5.42E+14
sp|P62314|	6	63	38.33952	4.84E+15	1.26E+14	########	2.9E+16	7.95E+15
sp|P62316|	6	63	18.42209	2.3E+15	1.25E+14	########	1.38E+16	8E+15
sp|P62318|	6	63	26.20752	9.65E+15	3.68E+14	########	5.79E+16	2.32E+16
sp|P62328|	6	63	108.6166	4.02E+18	3.71E+16	########	2E+19	2.33E+18
sp|P62330|	6	63	26.61374	6.16E+14	2.31E+13	########	3.69E+15	1E+15
sp|P62333|	6	63	18.6533	3.1E+15	1.66E+14	########	1.86E+16	1.05E+16
sp|P62424|	6	63	44.9009	2.67E+17	5.94E+15	########	1.6E+18	3.74E+17
sp|P62495|	6	63	4.322855	2.42E+12	5.6E+11	########	1.45E+13	3.53E+13
sp|P62701|	6	63	63.08708	2.32E+17	3.68E+15	########	1.39E+18	2.32E+17
sp|P62745|	6	63	57.85889	3.33E+16	5.76E+14	########	2E+17	3.63E+16
sp|P62750|	6	63	31.34147	4.99E+16	1.59E+15	########	3E+17	1E+17
sp|P62753|	6	63	43.35798	1.36E+17	3.14E+15	########	8.18E+17	1.98E+17
sp|P62760|	6	63	57.85417	3E+18	4.43E+16	########	2E+19	3E+18
sp|P62805|	6	63	148.6164	2E+19	1.42E+17	########	1E+20	9E+18
sp|P62820|	6	63	4.938091	1.24E+16	2.51E+15	########	7.44E+16	2E+17
sp|P62826|	6	63	140.0602	1.32E+17	9.41E+14	########	8E+17	5.93E+16
sp|P62829|	6	63	21.1684	1.13E+17	5.35E+15	########	6.8E+17	3.37E+17
sp|P62834|	6	63	26.24705	1.46E+17	5.56E+15	########	8.76E+17	3.5E+17
sp|P62837|	6	63	6.574284	7.2E+12	1.1E+12	########	4.32E+13	6.9E+13
sp|P62841|	6	63	64.67232	3.11E+16	4.8E+14	########	1.86E+17	3.03E+16
sp|P62847|	6	63	45.21129	6.12E+16	1.35E+15	########	3.67E+17	8.53E+16
sp|P62851|	6	63	46.83783	3E+16	6.41E+14	########	1.8E+17	4.04E+16
sp|P62854|	6	63	46.28307	2.97E+16	6.41E+14	########	1.78E+17	4.04E+16
sp|P62857|	6	63	55.91983	2.26E+16	4.05E+14	########	1.36E+17	2.55E+16
sp|P62861|	6	63	36.49246	4.95E+15	1.36E+14	########	2.97E+16	9E+15
sp|P62873|	6	63	70.0735	4E+19	5.02E+17	########	2E+20	3E+19
sp|P62879|	6	63	39.14546	1.93E+18	4.94E+16	########	1E+19	3.11E+18
sp|P62888|	6	63	51.65479	8.36E+16	1.62E+15	########	5.02E+17	1.02E+17
sp|P62899|	6	63	48.8899	3.63E+16	7.43E+14	########	########	4.68E+16
sp|P62906|	6	63	43.5363	9.39E+16	2.16E+15	########	5.64E+17	1.36E+17
sp|P62910|	6	63	45.7442	2.18E+16	4.76E+14	########	1.31E+17	3E+16
sp|P62913|	6	63	46.9689	3.87E+16	8.25E+14	########	2.32E+17	5.2E+16
sp|P62917|	6	63	102.472	2.93E+17	2.86E+15	########	1.76E+18	1.8E+17
sp|P62937|	6	63	16.53293	6E+18	########	########	4E+19	2E+19
sp|P62942|	6	63	98.09436	2.8E+16	2.85E+14	########	1.68E+17	1.8E+16
sp|P62979|	6	63	1.99526	3.2E+16	1.6E+16	########	########	1.01E+18
sp|P62993|	6	63	139.43	2.1E+16	1.51E+14	########	1.26E+17	9.49E+15
sp|P62995|	6	63	26.44944	2.15E+15	8.13E+13	########	1.29E+16	5.12E+15
sp|P63000|	6	63	25.19543	2.06E+17	8.16E+15	########	1.23E+18	5E+17
sp|P63010|	6	63	13.10622	2E+18	1.34E+17	########	1E+19	8E+18
sp|P63027|	6	63	15.03757	6.31E+17	4.2E+16	########	4E+18	3E+18
sp|P63092|	6	63	97.49621	8.96E+16	9.19E+14	########	5.38E+17	6E+16
sp|P63096|	6	63	128.7382	2.12E+18	1.65E+16	########	1E+19	1.04E+18

For Peer Review Only

sp|P63098|	6	63	57.24062	4.03E+17	7.04E+15	########	2E+18	4.44E+17
sp|P63104|	6	63	9.978894	1.74E+18	1.74E+17	########	1E+19	1E+19
sp|P63151|	6	63	2.75481	1.73E+15	6.29E+14	########	1.04E+16	4E+16
sp|P63162|	6	63	23.09159	2.48E+15	1.08E+14	########	1.49E+16	6.78E+15
sp|P63167|	6	63	3.012867	8.24E+14	2.73E+14	########	4.94E+15	2E+16
sp|P63172|	6	63	5.488584	5.02E+13	9.14E+12	########	3.01E+14	5.76E+14
sp|P63173|	6	63	54.59815	1.5E+16	2.76E+14	########	9.03E+16	2E+16
sp|P63208|	6	63	78.85193	1.15E+17	1.46E+15	########	6.93E+17	9.23E+16
sp|P63215|	6	63	7.490634	2.12E+16	2.82E+15	########	1.27E+17	1.78E+17
sp|P63218|	6	63	12.42742	2.47E+13	1.99E+12	########	1.48E+14	1.25E+14
sp|P63220|	6	63	47.67324	1.51E+16	3.16E+14	########	9.04E+16	1.99E+16
sp|P63241|	6	63	1.468285	1.13E+15	7.68E+14	########	6.77E+15	5E+16
sp|P63244|	6	63	77.61935	4.04E+17	5.21E+15	########	2.43E+18	3E+17
sp|P63261|	6	63	35.63939	6E+19	########	########	4E+20	1E+20
sp|P63313|	6	63	103.3453	4.41E+17	4.27E+15	########	2.65E+18	2.69E+17
sp|P67775|	6	63	6.657787	1.02E+16	1.53E+15	########	6.11E+16	9.64E+16
sp|P67870|	6	63	5.590514	4.26E+14	7.61E+13	########	2.55E+15	5E+15
sp|P67936|	6	63	81.78357	3.56E+17	4.35E+15	########	2.14E+18	2.74E+17
sp|P68032|	6	63	2.075077	########	########	########	2.85E+18	1E+19
sp|P68036|	6	63	105.0718	6.13E+16	5.83E+14	########	########	3.68E+16
sp|P68104|	6	63	52.2693	1.87E+18	3.59E+16	########	1E+19	2.26E+18
sp|P68363|	6	63	46.68019	6E+20	1E+19	########	3E+21	8E+20
sp|P68366|	6	63	26.98223	4E+18	1.59E+17	########	3E+19	1E+19
sp|P68371|	6	63	29.93955	3E+19	9.48E+17	########	2E+20	6E+19
sp|P68400|	6	63	7.724663	2.66E+15	3.44E+14	########	1.6E+16	2.17E+16
sp|P68402|	6	63	43.36307	2.09E+17	4.82E+15	########	1.25E+18	########
sp|P68431|	6	63	38.71207	6.15E+14	1.59E+13	########	3.69E+15	1E+15
sp|P68871|	6	63	57.12826	3E+21	5E+19	########	2E+22	3E+21
sp|P69849|	6	63	31.18657	1.73E+14	5.55E+12	########	1.04E+15	3.5E+14
sp|P69891|	6	63	1.979341	7.02E+16	3.55E+16	########	4.21E+17	2.24E+18
sp|P69905|	6	63	33.04353	1E+21	3E+19	########	7E+21	2E+21
sp|P78324|	6	63	11.39741	2E+17	1.69E+16	########	1E+18	1.06E+18
sp|P78344|	6	63	33.34776	2.25E+14	6.74E+12	########	1.35E+15	4.24E+14
sp|P78347|	6	63	33.31768	3.1E+14	9.3E+12	########	1.86E+15	5.86E+14
sp|P78352|	6	63	67.90452	1.91E+17	2.82E+15	########	1.15E+18	########
sp|P78356|	6	63	12.01625	3.75E+15	3.12E+14	########	2.25E+16	1.96E+16
sp|P78357|	6	63	32.67631	5.46E+17	1.67E+16	########	3E+18	1.05E+18
sp|P78362|	6	63	31.95245	1.85E+15	5.79E+13	########	1.11E+16	3.65E+15
sp|P78369|	6	63	1.5587	1.74E+12	1.11E+12	########	1.04E+13	7.02E+13
sp|P78371|	6	63	26.23876	7.13E+16	2.72E+15	########	4.28E+17	1.71E+17
sp|P78417|	6	63	153.8465	1.61E+17	1.04E+15	########	9.64E+17	6.58E+16
sp|P78508|	6	63	3.363751	2.73E+13	8.11E+12	########	1.64E+14	5.11E+14
sp|P78527|	6	63	87.02653	3.77E+16	4.33E+14	########	2.26E+17	2.73E+16
sp|P78559|	6	63	19.79622	1.4E+18	7.08E+16	########	8E+18	4.46E+18
sp|P80404|	6	63	40.12974	7.24E+17	1.8E+16	########	4.34E+18	1E+18
sp|P80723|	6	63	13.93398	3.84E+18	2.76E+17	########	2E+19	2E+19
sp|P82909|	6	63	13.07598	1.57E+15	1.2E+14	########	9.41E+15	7.56E+15
sp|P83731|	6	63	43.39385	2.67E+16	6.16E+14	########	########	3.88E+16
sp|P83916|	6	63	29.36223	3.84E+14	1.31E+13	########	2.3E+15	8.24E+14
sp|P84074|	6	63	7.685824	2.37E+15	3.08E+14	########	1.42E+16	1.94E+16
sp|P84077|	6	63	27.79067	2.14E+17	7.71E+15	########	1.29E+18	4.86E+17
sp|P84085|	6	63	1.473008	1.48E+14	1.01E+14	########	8.89E+14	6.34E+15
sp|P84090|	6	63	13.56695	2.28E+14	1.68E+13	########	1.37E+15	1.06E+15
sp|P84095|	6	63	9.241033	9.99E+15	1.08E+15	########	5.99E+16	6.81E+16
sp|P84098|	6	63	32.0268	5.57E+14	1.74E+13	########	3.34E+15	1.1E+15
sp|P84103|	6	63	18.41408	1.55E+16	8.44E+14	########	9.33E+16	5.32E+16

For Peer Review Only

sp|P84243|	6	63	119.8409	2E+19	1.49E+17	########	1E+20	9E+18
sp|P98160|	6	63	27.58405	4.33E+16	1.57E+15	########	2.6E+17	1E+17
sp|P98179|	6	63	1.054518	5.11E+13	4.84E+13	########	3.06E+14	3E+15
sp|P98194|	6	63	2.204586	2.3E+12	1.05E+12	########	1.38E+13	6.58E+13
sp|P99999|	6	63	11.58582	2.4E+17	2.07E+16	########	1.44E+18	1.3E+18
sp|Q00013|	6	63	15.51136	9.31E+14	6E+13	########	5.58E+15	3.78E+15
sp|Q00059|	6	63	24.80447	1.41E+15	5.68E+13	########	8.45E+15	3.58E+15
sp|Q00169|	6	63	31.78925	3.48E+15	1.1E+14	########	2.09E+16	7E+15
sp|Q00325|	6	63	66.6424	4.3E+17	6.46E+15	########	2.58E+18	########
sp|Q00341|	6	63	18.82845	3.65E+14	1.94E+13	########	2.19E+15	1.22E+15
sp|Q00535|	6	63	30.31581	1.14E+16	3.76E+14	########	6.84E+16	2.37E+16
sp|Q00577|	6	63	5.420593	1.86E+16	3.43E+15	########	1.12E+17	2.16E+17
sp|Q00587|	6	63	1.768965	1.74E+13	9.84E+12	########	1.04E+14	6.2E+14
sp|Q00610|	6	63	31.81625	9E+19	3E+18	########	5E+20	2E+20
sp|Q00688|	6	63	23.54933	5.18E+15	2.2E+14	########	3.11E+16	1.39E+16
sp|Q00765|	6	63	2.515671	2.24E+14	8.9E+13	########	1.34E+15	5.61E+15
sp|Q00796|	6	63	63.59012	3.65E+16	5.74E+14	########	2.19E+17	3.61E+16
sp|Q00839|	6	63	251.9836	5.19E+17	2.06E+15	########	3E+18	1.3E+17
sp|Q01064|	6	63	19.52134	3.7E+14	1.89E+13	########	2E+15	1E+15
sp|Q01082|	6	63	50.58843	2E+19	3.36E+17	########	1E+20	2E+19
sp|Q01105|	6	63	24.24721	3.02E+15	1.25E+14	########	1.81E+16	8E+15
sp|Q01130|	6	63	36.52077	2.14E+15	5.85E+13	########	1.28E+16	3.68E+15
sp|Q01432|	6	63	1.366269	1.74E+12	1.27E+12	########	1.04E+13	8.01E+13
sp|Q01433|	6	63	3.28441	6.17E+13	1.88E+13	########	3.7E+14	1.18E+15
sp|Q01469|	6	63	68.52467	1.57E+17	2.29E+15	########	9.39E+17	########
sp|Q01484|	6	63	71.95485	2.58E+18	3.59E+16	########	2E+19	2E+18
sp|Q01518|	6	63	27.37799	4.09E+16	1.49E+15	########	2.45E+17	9.41E+16
sp|Q01650|	6	63	7.204569	4.51E+14	6.26E+13	########	2.71E+15	3.94E+15
sp|Q01813|	6	63	39.9153	6.61E+17	1.66E+16	########	3.97E+18	1.04E+18
sp|Q01814|	6	63	18.68466	1.68E+18	8.98E+16	########	1E+19	6E+18
sp|Q01844|	6	63	1.347554	1.62E+12	1.21E+12	########	9.74E+12	7.59E+13
sp|Q01995|	6	63	6.420977	4.09E+16	6.38E+15	########	2.46E+17	4.02E+17
sp|Q02108|	6	63	########	3.4E+11	3.97E+11	########	2.04E+12	2.5E+13
sp|Q02153|	6	63	5.999408	1.69E+14	2.82E+13	########	1.01E+15	1.78E+15
sp|Q02156|	6	63	23.45937	2.83E+15	1.21E+14	########	1.7E+16	7.59E+15
sp|Q02218|	6	63	157.6974	4.9E+17	3.1E+15	########	3E+18	1.96E+17
sp|Q02246|	6	63	4.726278	1.37E+15	2.9E+14	########	8.23E+15	1.83E+16
sp|Q02252|	6	63	14.56333	4.09E+17	2.81E+16	########	2.46E+18	1.77E+18
sp|Q02338|	6	63	67.96673	3.09E+16	4.55E+14	########	1.86E+17	3E+16
sp|Q02543|	6	63	42.23514	8.72E+15	2.06E+14	########	5.23E+16	1.3E+16
sp|Q02750|	6	63	25.0575	4.64E+16	1.85E+15	########	2.79E+17	1.17E+17
sp|Q02790|	6	63	########	4.52E+14	6.27E+14	########	2.71E+15	4E+16
sp|Q02818|	6	63	11.95488	4.27E+14	3.57E+13	########	2.56E+15	2.25E+15
sp|Q02878|	6	63	61.21585	8.54E+16	1.39E+15	########	5.12E+17	8.78E+16
sp|Q02880|	6	63	19.58024	1.17E+14	6E+12	########	7.05E+14	3.78E+14
sp|Q02952|	6	63	24.89762	2.29E+16	9.21E+14	########	1.38E+17	5.8E+16
sp|Q02978|	6	63	85.77188	3.26E+17	3.8E+15	########	1.95E+18	2.39E+17
sp|Q03001|	6	63	7.619806	5.08E+15	6.67E+14	########	3.05E+16	4.2E+16
sp|Q03013|	6	63	1.567977	1.33E+15	8.5E+14	########	7.99E+15	5E+16
sp|Q03135|	6	63	10.88514	6.91E+14	6.35E+13	########	4.15E+15	4E+15
sp|Q03154|	6	63	24.26423	5.31E+14	2.19E+13	########	3.18E+15	1.38E+15
sp|Q03252|	6	63	133.4294	8.57E+16	6.43E+14	########	5.14E+17	4.05E+16
sp|Q03426|	6	63	########	2.55E+11	2.65E+11	########	1.53E+12	1.67E+13
sp|Q04323|	6	63	6.143686	2.27E+14	3.69E+13	########	1.36E+15	2E+15
sp|Q04446|	6	63	6.477829	3.71E+14	5.73E+13	########	2.23E+15	3.61E+15
sp|Q04609|	6	63	3.33606	1.75E+15	5.24E+14	########	1.05E+16	3.3E+16

For Peer Review Only

sp|Q04637|	6	63	4.564238	5.21E+13	1.14E+13	########	3.13E+14	7.2E+14
sp|Q04760|	6	63	213.6298	########	1.12E+15	########	1E+18	7.05E+16
sp|Q04828|	6	63	5.79062	4.18E+14	7.21E+13	########	2.51E+15	4.54E+15
sp|Q04837|	6	63	25.20949	3.63E+15	1.44E+14	########	2.18E+16	9.07E+15
sp|Q04917|	6	63	12.13806	1.31E+17	1.08E+16	########	7.87E+17	6.81E+17
sp|Q05193|	6	63	64.90307	1E+19	2.02E+17	########	8E+19	1E+19
sp|Q05329|	6	63	10.624	2.62E+15	2.46E+14	########	1.57E+16	2E+16
sp|Q05469|	6	63	4.827441	1.69E+15	3.49E+14	########	1.01E+16	2E+16
sp|Q05586|	6	63	9.102244	7.43E+13	8.16E+12	########	4.46E+14	5.14E+14
sp|Q05639|	6	63	4.359226	1.14E+17	2.62E+16	########	6.84E+17	1.65E+18
sp|Q06124|	6	63	37.83504	2.24E+16	5.91E+14	########	1.34E+17	3.73E+16
sp|Q06136|	6	63	7.262659	2.71E+14	3.74E+13	########	1.63E+15	2.35E+15
sp|Q06323|	6	63	43.69465	2.67E+15	6.12E+13	########	1.6E+16	3.85E+15
sp|Q06481|	6	63	1.12838	3.24E+11	2.87E+11	########	1.94E+12	1.81E+13
sp|Q06830|	6	63	74.47335	3.15E+18	4.23E+16	########	2E+19	3E+18
sp|Q07002|	6	63	7.278043	3.05E+14	4.19E+13	########	1.83E+15	2.64E+15
sp|Q07020|	6	63	40.86818	6.43E+16	1.57E+15	########	3.86E+17	9.91E+16
sp|Q07021|	6	63	77.26079	1.93E+17	2.5E+15	########	1.16E+18	1.57E+17
sp|Q07065|	6	63	27.22291	3.84E+14	1.41E+13	########	2.3E+15	8.88E+14
sp|Q07157|	6	63	6.714544	3.67E+14	5.47E+13	########	2.2E+15	3.45E+15
sp|Q07666|	6	63	44.88257	2.94E+15	6.55E+13	########	1.76E+16	4.12E+15
sp|Q07866|	6	63	8.671053	1.4E+15	1.61E+14	########	8.39E+15	1.02E+16
sp|Q07954|	6	63	15.92777	6.49E+15	4.07E+14	########	3.89E+16	2.57E+16
sp|Q07955|	6	63	53.39964	1.39E+16	2.6E+14	########	8.34E+16	2E+16
sp|Q07960|	6	63	64.42519	3.89E+16	6.04E+14	########	2.34E+17	4E+16
sp|Q08174|	6	63	5.195206	1.13E+15	2.18E+14	########	6.79E+15	1.37E+16
sp|Q08209|	6	63	106.509	1.88E+18	1.77E+16	########	1E+19	1.11E+18
sp|Q08211|	6	63	218.5137	9.49E+16	4.34E+14	########	5.69E+17	2.74E+16
sp|Q08257|	6	63	4.245609	2.65E+15	6.23E+14	########	1.59E+16	3.93E+16
sp|Q08380|	6	63	13.09437	2.92E+15	2.23E+14	########	1.75E+16	1E+16
sp|Q08431|	6	63	3.651234	2.82E+14	7.71E+13	########	1.69E+15	4.86E+15
sp|Q08462|	6	63	34.55555	1.94E+14	5.62E+12	########	1.16E+15	3.54E+14
sp|Q08495|	6	63	15.58934	2.87E+15	1.84E+14	########	1.72E+16	1.16E+16
sp|Q08499|	6	63	1.207894	2.25E+12	1.86E+12	########	1.35E+13	1.17E+14
sp|Q08623|	6	63	57.57433	1.62E+15	2.82E+13	########	9.75E+15	1.78E+15
sp|Q08722|	6	63	16.1829	6.53E+16	4.03E+15	########	3.92E+17	2.54E+17
sp|Q08752|	6	63	4.604391	1.85E+13	4.03E+12	########	1.11E+14	2.54E+14
sp|Q08828|	6	63	2.87021	4.6E+13	1.6E+13	########	2.76E+14	1E+15
sp|Q08AD1|	6	63	2.687777	8.82E+12	3.28E+12	########	5.29E+13	2.07E+14
sp|Q08AE8|	6	63	########	2.87E+11	2.88E+11	########	1.72E+12	1.82E+13
sp|Q08AM6|	6	63	27.22017	8.83E+14	3.24E+13	########	5.3E+15	2.04E+15
sp|Q09028|	6	63	12.80006	4.37E+14	3.42E+13	########	2.62E+15	2.15E+15
sp|Q09666|	6	63	7.127614	2.25E+16	3.16E+15	########	1.35E+17	2E+17
sp|Q0VDG4|	6	63	28.94118	7.8E+14	2.7E+13	########	4.68E+15	1.7E+15
sp|Q10567|	6	63	2.513491	1.59E+15	6.34E+14	########	9.57E+15	4E+16
sp|Q10713|	6	63	23.17508	6.46E+14	2.79E+13	########	3.88E+15	1.76E+15
sp|Q12756|	6	63	27.12026	9.26E+14	3.41E+13	########	5.55E+15	2.15E+15
sp|Q12765|	6	63	12.49788	2.31E+17	1.85E+16	########	1.39E+18	########
sp|Q12768|	6	63	9.133432	1.64E+14	1.79E+13	########	9.82E+14	1.13E+15
sp|Q12791|	6	63	9.585385	8.04E+13	8.38E+12	########	4.82E+14	5.28E+14
sp|Q12792|	6	63	1.36999	7.08E+12	5.17E+12	########	4.25E+13	3.26E+14
sp|Q12797|	6	63	33.79655	1.49E+15	4.41E+13	########	8.94E+15	2.78E+15
sp|Q12846|	6	63	5.035227	2.27E+13	4.5E+12	########	1.36E+14	2.84E+14
sp|Q12849|	6	63	43.34547	3.37E+14	7.77E+12	########	2.02E+15	4.9E+14
sp|Q12860|	6	63	21.25205	2E+18	9.92E+16	########	1E+19	6E+18
sp|Q12879|	6	63	8.475933	1.65E+13	1.95E+12	########	9.91E+13	1.23E+14

For Peer Review Only

sp|Q12904|	6	63	6.778515	3.95E+14	5.83E+13	########	2.37E+15	3.67E+15
sp|Q12905|	6	63	23.12691	3.07E+16	1.33E+15	########	########	8E+16
sp|Q12906|	6	63	11.94239	1.11E+16	9.31E+14	########	6.67E+16	5.87E+16
sp|Q12907|	6	63	9.774882	4.55E+14	4.66E+13	########	2.73E+15	3E+15
sp|Q12931|	6	63	58.15685	2.86E+16	4.92E+14	########	1.72E+17	3.1E+16
sp|Q12955|	6	63	90.79231	6.85E+16	7.54E+14	########	4.11E+17	4.75E+16
sp|Q12959|	6	63	6.910503	7.21E+14	1.04E+14	########	4.33E+15	7E+15
sp|Q12965|	6	63	4.181544	1.64E+13	3.93E+12	########	9.86E+13	2.48E+14
sp|Q12974|	6	63	7.454529	9.41E+12	1.26E+12	########	5.65E+13	7.95E+13
sp|Q12979|	6	63	39.14111	8.5E+15	2.17E+14	########	5.1E+16	1E+16
sp|Q13011|	6	63	9.620633	1.85E+15	1.92E+14	########	1.11E+16	1E+16
sp|Q13015|	6	63	1.251537	6.55E+11	5.24E+11	########	3.93E+12	3.3E+13
sp|Q13017|	6	63	2.948701	3.72E+13	1.26E+13	########	2.23E+14	7.94E+14
sp|Q13045|	6	63	5.380643	4.34E+13	8.07E+12	########	2.61E+14	5.09E+14
sp|Q13057|	6	63	35.295	3.51E+15	9.95E+13	########	2.11E+16	6.27E+15
sp|Q13085|	6	63	5.061703	2.24E+14	4.42E+13	########	1.34E+15	2.79E+15
sp|Q13098|	6	63	1.481004	2.29E+14	1.55E+14	########	1.37E+15	9.75E+15
sp|Q13099|	6	63	1.540179	3.03E+14	1.97E+14	########	1.82E+15	1.24E+16
sp|Q13126|	6	63	34.91418	8.4E+15	2.41E+14	########	5.04E+16	2E+16
sp|Q13131|	6	63	14.34279	1.98E+14	1.38E+13	########	1.19E+15	8.71E+14
sp|Q13148|	6	63	60.3539	2.03E+15	3.36E+13	########	1.22E+16	2.11E+15
sp|Q13151|	6	63	84.32236	3.78E+15	4.48E+13	########	2.27E+16	2.82E+15
sp|Q13153|	6	63	35.27233	4.28E+16	1.21E+15	########	2.57E+17	8E+16
sp|Q13155|	6	63	19.21537	3.94E+14	2.05E+13	########	2.36E+15	1E+15
sp|Q13162|	6	63	5.257263	7.64E+13	1.45E+13	########	4.59E+14	9.16E+14
sp|Q13177|	6	63	18.09963	1.75E+15	9.68E+13	########	1.05E+16	6.1E+15
sp|Q13185|	6	63	4.949146	3.11E+13	6.29E+12	########	1.87E+14	3.96E+14
sp|Q13200|	6	63	35.53731	6.11E+15	1.72E+14	########	3.67E+16	1.08E+16
sp|Q13217|	6	63	4.873017	1.21E+13	2.48E+12	########	7.26E+13	1.56E+14
sp|Q13224|	6	63	6.119203	2.6E+13	4.25E+12	########	1.56E+14	2.68E+14
sp|Q13228|	6	63	79.45006	4.03E+17	5.08E+15	########	2E+18	3.2E+17
sp|Q13232|	6	63	########	1.31E+13	1.34E+13	########	7.84E+13	8.47E+14
sp|Q13243|	6	63	6.164782	1.69E+14	2.74E+13	########	1.01E+15	1.73E+15
sp|Q13263|	6	63	17.12111	1.51E+14	8.83E+12	########	9.07E+14	5.56E+14
sp|Q13303|	6	63	16.78915	1.06E+17	6.32E+15	########	6.37E+17	3.98E+17
sp|Q13332|	6	63	30.56056	7.5E+14	2.45E+13	########	4.5E+15	2E+15
sp|Q13336|	6	63	14.7024	4.48E+14	3.05E+13	########	2.69E+15	1.92E+15
sp|Q13347|	6	63	12.43867	1.33E+15	1.07E+14	########	7.98E+15	6.73E+15
sp|Q13362|	6	63	14.17114	2E+14	1.41E+13	########	1.2E+15	8.9E+14
sp|Q13363|	6	63	26.32266	1.53E+16	5.83E+14	########	9.21E+16	3.67E+16
sp|Q13367|	6	63	13.35693	8.62E+15	6.45E+14	########	5.17E+16	4.06E+16
sp|Q13404|	6	63	103.4227	1.54E+15	1.49E+13	########	9.27E+15	9.41E+14
sp|Q13409|	6	63	10.18913	2.61E+15	2.57E+14	########	1.57E+16	1.62E+16
sp|Q13418|	6	63	26.34309	1.06E+14	4.02E+12	########	6.35E+14	2.53E+14
sp|Q13423|	6	63	87.0735	9.21E+17	1.06E+16	########	6E+18	7E+17
sp|Q13424|	6	63	23.23783	1.15E+16	4.94E+14	########	6.89E+16	3.11E+16
sp|Q13442|	6	63	43.44973	2.55E+15	5.87E+13	########	1.53E+16	3.7E+15
sp|Q13449|	6	63	21.4191	2.15E+16	1E+15	########	1.29E+17	6.33E+16
sp|Q13491|	6	63	32.5025	9.63E+16	2.96E+15	########	5.78E+17	2E+17
sp|Q13492|	6	63	13.09158	2.67E+15	2.04E+14	########	1.6E+16	1.28E+16
sp|Q13496|	6	63	3.14957	1.38E+13	4.39E+12	########	8.3E+13	2.77E+14
sp|Q13505|	6	63	17.29444	1.62E+15	9.34E+13	########	9.69E+15	6E+15
sp|Q13509|	6	63	########	3.87E+17	4.04E+17	########	2.32E+18	3E+19
sp|Q13510|	6	63	2.514442	2.29E+15	9.12E+14	########	1.38E+16	5.75E+16
sp|Q13526|	6	63	38.71448	5.14E+16	1.33E+15	########	3.09E+17	8.37E+16
sp|Q13554|	6	63	32.50965	3.45E+17	1.06E+16	########	2.07E+18	6.68E+17

For Peer Review Only

sp|Q13555|	6	63	27.3179	4.67E+17	1.71E+16	########	2.8E+18	1E+18
sp|Q13557|	6	63	17.37748	7.92E+16	4.56E+15	########	4.75E+17	3E+17
sp|Q13561|	6	63	6.02247	1.47E+16	2.44E+15	########	8.83E+16	1.54E+17
sp|Q13564|	6	63	26.36929	9.16E+14	3.47E+13	########	5.5E+15	2.19E+15
sp|Q13574|	6	63	2.178038	4.99E+11	2.29E+11	########	2.99E+12	1.44E+13
sp|Q13586|	6	63	4.25248	3.29E+13	7.73E+12	########	1.97E+14	4.87E+14
sp|Q13596|	6	63	9.720991	2.91E+14	2.99E+13	########	1.75E+15	1.89E+15
sp|Q13613|	6	63	10.89439	1.87E+14	1.71E+13	########	1.12E+15	1.08E+15
sp|Q13616|	6	63	4.166407	8.24E+13	1.98E+13	########	4.94E+14	1.25E+15
sp|Q13617|	6	63	3.427728	1.38E+14	4.02E+13	########	8.28E+14	2.54E+15
sp|Q13618|	6	63	47.70657	2.03E+15	4.26E+13	########	1.22E+16	2.68E+15
sp|Q13620|	6	63	21.48609	2.46E+14	1.15E+13	########	1.48E+15	7.22E+14
sp|Q13630|	6	63	31.59435	4.39E+14	1.39E+13	########	2.63E+15	8.75E+14
sp|Q13642|	6	63	2.922617	1.14E+15	3.89E+14	########	6.82E+15	2.45E+16
sp|Q13683|	6	63	11.15995	5.56E+14	4.98E+13	########	3.33E+15	3.14E+15
sp|Q13724|	6	63	14.76572	1.55E+14	1.05E+13	########	9.32E+14	6.62E+14
sp|Q13740|	6	63	21.95199	3.03E+16	1.38E+15	########	1.82E+17	9E+16
sp|Q13813|	6	63	52.87035	3E+19	5.19E+17	########	2E+20	3E+19
sp|Q13825|	6	63	43.95964	4.49E+15	1.02E+14	########	2.7E+16	6.44E+15
sp|Q13838|	6	63	7.344067	3.15E+15	4.28E+14	########	1.89E+16	3E+16
sp|Q13867|	6	63	18.18445	2.71E+15	1.49E+14	########	1.62E+16	9.37E+15
sp|Q13875|	6	63	3.399125	8.55E+14	2.51E+14	########	5.13E+15	1.58E+16
sp|Q13884|	6	63	3.582512	2.27E+14	6.32E+13	########	1.36E+15	3.98E+15
sp|Q13885|	6	63	44.23533	5E+20	1E+19	########	3E+21	7E+20
sp|Q13907|	6	63	19.88054	6.94E+14	3.49E+13	########	4.17E+15	2.2E+15
sp|Q13938|	6	63	1.893487	4.82E+14	2.55E+14	########	2.89E+15	1.6E+16
sp|Q14008|	6	63	50.3469	8.67E+15	1.72E+14	########	5.2E+16	1.09E+16
sp|Q14011|	6	63	2.531307	1.14E+14	4.49E+13	########	6.81E+14	2.83E+15
sp|Q14019|	6	63	101.4353	1.09E+17	1.07E+15	########	6.53E+17	6.76E+16
sp|Q14103|	6	63	14.87797	3.26E+16	2.19E+15	########	1.96E+17	1.38E+17
sp|Q14108|	6	63	56.08167	1.2E+17	2.14E+15	########	7.2E+17	1.35E+17
sp|Q14112|	6	63	139.4931	8.38E+14	6.01E+12	########	5.03E+15	3.78E+14
sp|Q14118|	6	63	6.427654	1.6E+14	2.48E+13	########	9.58E+14	1.56E+15
sp|Q14123|	6	63	3.021817	2.92E+13	9.67E+12	########	1.75E+14	6.09E+14
sp|Q14139|	6	63	15.21779	3.33E+14	2.19E+13	########	2E+15	1.38E+15
sp|Q14141|	6	63	5.554353	6.66E+15	1.2E+15	########	3.99E+16	7.55E+16
sp|Q14152|	6	63	18.95535	5.65E+15	2.98E+14	########	3.39E+16	2E+16
sp|Q14155|	6	63	55.44965	7.74E+15	1.39E+14	########	4.64E+16	8.79E+15
sp|Q14156|	6	63	17.23596	1.47E+13	8.54E+11	########	8.84E+13	5.38E+13
sp|Q14157|	6	63	6.661736	2.12E+13	3.18E+12	########	1.27E+14	2E+14
sp|Q14160|	6	63	8.773393	7.09E+13	8.08E+12	########	4.26E+14	5.09E+14
sp|Q14161|	6	63	1.514555	9.12E+11	6.02E+11	########	5.47E+12	3.79E+13
sp|Q14165|	6	63	6.40417	3.35E+13	5.24E+12	########	2.01E+14	3.3E+14
sp|Q14166|	6	63	3.121618	1.05E+14	3.37E+13	########	6.3E+14	2.12E+15
sp|Q14168|	6	63	34.61482	9.46E+15	2.73E+14	########	5.68E+16	1.72E+16
sp|Q14185|	6	63	3.186202	2.09E+14	6.55E+13	########	1.25E+15	4E+15
sp|Q14194|	6	63	64.2529	3E+18	4.69E+16	########	2E+19	3E+18
sp|Q14195|	6	63	81.92274	1E+18	1.5E+16	########	7E+18	9.42E+17
sp|Q14203|	6	63	12.10078	2.52E+16	2.08E+15	########	1.51E+17	1.31E+17
sp|Q14204|	6	63	80.67017	1E+19	1.34E+17	########	6E+19	8E+18
sp|Q14232|	6	63	5.890424	1.06E+13	1.79E+12	########	6.34E+13	1.13E+14
sp|Q14240|	6	63	1.895375	6.03E+15	3.18E+15	########	3.62E+16	2E+17
sp|Q14247|	6	63	13.51337	2.62E+14	1.94E+13	########	1.57E+15	1.22E+15
sp|Q14254|	6	63	19.59778	5.31E+16	2.71E+15	########	########	1.71E+17
sp|Q14257|	6	63	10.60244	8.02E+14	7.57E+13	########	4.81E+15	4.77E+15
sp|Q14289|	6	63	9.900306	1.57E+15	1.58E+14	########	9.4E+15	9.97E+15

For Peer Review Only

sp|Q14315|	6	63	########	8.01E+13	2.82E+14	########	4.81E+14	1.78E+16
sp|Q14318|	6	63	52.52628	8.38E+14	1.6E+13	########	5.03E+15	1E+15
sp|Q14344|	6	63	27.89229	2.34E+16	8.39E+14	########	1.4E+17	5.29E+16
sp|Q14353|	6	63	15.029	1.47E+15	9.8E+13	########	8.84E+15	6.18E+15
sp|Q14376|	6	63	3.090666	1.22E+13	3.94E+12	########	7.31E+13	2.48E+14
sp|Q14416|	6	63	########	3.13E+13	5.39E+13	########	1.88E+14	3.39E+15
sp|Q14444|	6	63	18.07122	1.14E+15	6.33E+13	########	6.86E+15	3.99E+15
sp|Q14515|	6	63	6.71879	1.14E+15	1.7E+14	########	6.85E+15	1.07E+16
sp|Q14558|	6	63	22.08136	8.09E+15	3.67E+14	########	4.86E+16	2E+16
sp|Q14571|	6	63	13.20396	3.84E+14	2.91E+13	########	2.3E+15	2E+15
sp|Q14624|	6	63	3.146084	2.21E+15	7.03E+14	########	1.33E+16	4.43E+16
sp|Q14642|	6	63	13.60449	2.46E+14	1.81E+13	########	1.48E+15	1E+15
sp|Q14643|	6	63	22.77839	1.34E+15	5.88E+13	########	8.03E+15	3.7E+15
sp|Q14677|	6	63	11.05034	4.8E+13	4.35E+12	########	2.88E+14	2.74E+14
sp|Q14683|	6	63	8.374758	3.37E+12	4.02E+11	########	2.02E+13	2.53E+13
sp|Q14696|	6	63	10.08628	1.88E+13	1.86E+12	########	1.13E+14	1.17E+14
sp|Q14697|	6	63	36.63704	9.02E+16	2.46E+15	########	5.41E+17	1.55E+17
sp|Q14699|	6	63	2.353652	4.66E+13	1.98E+13	########	2.8E+14	1E+15
sp|Q14721|	6	63	1.466807	6.16E+12	4.2E+12	########	3.7E+13	2.65E+14
sp|Q14738|	6	63	81.15046	1.31E+16	1.61E+14	########	7.85E+16	1E+16
sp|Q14764|	6	63	45.41149	4.78E+17	1.05E+16	########	3E+18	6.63E+17
sp|Q14832|	6	63	6.087118	2.26E+14	3.71E+13	########	1.35E+15	2E+15
sp|Q14847|	6	63	145.4788	1.05E+16	7.21E+13	########	6.29E+16	4.54E+15
sp|Q14894|	6	63	111.909	########	3.37E+15	########	2E+18	2.12E+17
sp|Q14914|	6	63	26.29134	2.07E+16	7.88E+14	########	1.24E+17	4.97E+16
sp|Q14956|	6	63	3.26132	2.56E+14	7.84E+13	########	1.54E+15	4.94E+15
sp|Q14964|	6	63	21.06099	5.85E+14	2.78E+13	########	3.51E+15	1.75E+15
sp|Q14974|	6	63	9.654882	5.23E+16	5.42E+15	########	3.14E+17	########
sp|Q14978|	6	63	2.673676	1.32E+12	4.93E+11	########	7.91E+12	3.11E+13
sp|Q14980|	6	63	49.30621	1.61E+15	3.27E+13	########	9.68E+15	2.06E+15
sp|Q14982|	6	63	10.37545	6.38E+16	6.15E+15	########	3.83E+17	########
sp|Q14C86	6	63	1.355046	9.3E+11	6.86E+11	########	5.58E+12	4.32E+13
sp|Q14CZ8	6	63	7.615009	6.73E+16	8.83E+15	########	4.04E+17	5.56E+17
sp|Q15008|	6	63	9.75099	3.94E+14	4.04E+13	########	2.36E+15	3E+15
sp|Q15019|	6	63	3.560432	6.92E+16	1.94E+16	########	4.15E+17	########
sp|Q15029|	6	63	25.85715	5.1E+14	1.97E+13	########	3.06E+15	1.24E+15
sp|Q15031|	6	63	38.05298	3.11E+14	8.16E+12	########	1.86E+15	5.14E+14
sp|Q15041|	6	63	12.87104	2.2E+15	1.71E+14	########	1.32E+16	1.07E+16
sp|Q15042|	6	63	32.11971	3.4E+14	1.06E+13	########	2.04E+15	6.67E+14
sp|Q15046|	6	63	9.162231	4.21E+14	4.6E+13	########	2.53E+15	2.9E+15
sp|Q15049|	6	63	5.605528	5.95E+15	1.06E+15	########	3.57E+16	6.68E+16
sp|Q15056|	6	63	31.20767	6.91E+15	2.21E+14	########	4.15E+16	1.39E+16
sp|Q15067|	6	63	22.00548	2.39E+15	1.09E+14	########	1.44E+16	6.85E+15
sp|Q15075|	6	63	37.50982	2.24E+16	5.96E+14	########	1.34E+17	4E+16
sp|Q15084|	6	63	19.20737	6.96E+15	3.63E+14	########	4.18E+16	2E+16
sp|Q15102|	6	63	1.326595	4.1E+14	3.09E+14	########	2.46E+15	1.95E+16
sp|Q15111|	6	63	########	6.56E+13	6.72E+13	########	3.93E+14	4.23E+15
sp|Q15119|	6	63	29.41871	8.51E+14	2.89E+13	########	5.11E+15	1.82E+15
sp|Q15120|	6	63	49.13464	1.52E+15	3.1E+13	########	9.15E+15	1.95E+15
sp|Q15121|	6	63	4.490618	5.35E+16	1.19E+16	########	3.21E+17	7.51E+17
sp|Q15126|	6	63	8.395294	2.1E+14	2.5E+13	########	1.26E+15	2E+15
sp|Q15149|	6	63	4.865486	2E+18	3.16E+17	########	9E+18	2E+19
sp|Q15165|	6	63	29.37202	4.42E+16	1.5E+15	########	2.65E+17	9.48E+16
sp|Q15181|	6	63	167.3871	1.04E+17	6.19E+14	########	6.21E+17	3.9E+16
sp|Q15185|	6	63	20.80293	1.78E+16	8.55E+14	########	1.07E+17	5.39E+16
sp|Q15223|	6	63	1.766722	1.14E+14	6.46E+13	########	6.85E+14	4E+15

For Peer Review Only

sp|Q15233|	6	63	114.3137	1.01E+16	8.82E+13	########	6.05E+16	5.55E+15
sp|Q15257|	6	63	1.882009	3.66E+15	1.95E+15	########	2.2E+16	1.23E+17
sp|Q15274|	6	63	14.84178	1.1E+15	7.39E+13	########	6.58E+15	5E+15
sp|Q15276|	6	63	8.266602	8.63E+13	1.04E+13	########	5.18E+14	6.58E+14
sp|Q15286|	6	63	10.22338	1.43E+15	1.4E+14	########	8.56E+15	8.79E+15
sp|Q15293|	6	63	9.125624	2.54E+14	2.78E+13	########	1.52E+15	1.75E+15
sp|Q15334|	6	63	8.640322	1.33E+16	1.54E+15	########	8.01E+16	9.73E+16
sp|Q15349|	6	63	6.564235	1.16E+14	1.76E+13	########	6.93E+14	1E+15
sp|Q15363|	6	63	2.048635	1.35E+14	6.59E+13	########	8.11E+14	4E+15
sp|Q15365|	6	63	8.821517	5.98E+15	6.77E+14	########	3.59E+16	4.27E+16
sp|Q15366|	6	63	10.06472	6.05E+15	6.01E+14	########	3.63E+16	3.78E+16
sp|Q15369|	6	63	14.54039	7.7E+14	5.3E+13	########	4.62E+15	3.34E+15
sp|Q15370|	6	63	35.89513	2.39E+15	6.67E+13	########	1.44E+16	4.2E+15
sp|Q15382|	6	63	8.916657	4.53E+13	5.08E+12	########	2.72E+14	3.2E+14
sp|Q15388|	6	63	10.63698	1.95E+14	1.84E+13	########	1.17E+15	1.16E+15
sp|Q15404|	6	63	5.385481	1.1E+14	2.05E+13	########	6.62E+14	1.29E+15
sp|Q15417|	6	63	########	1.09E+14	2.6E+14	########	6.54E+14	1.64E+16
sp|Q15435|	6	63	126.8325	6.33E+16	4.99E+14	########	3.8E+17	3E+16
sp|Q15436|	6	63	11.5166	9.39E+14	8.15E+13	########	5.63E+15	5.13E+15
sp|Q15493|	6	63	36.54209	8.82E+14	2.41E+13	########	5.29E+15	1.52E+15
sp|Q15555|	6	63	60.345	2.97E+16	4.92E+14	########	1.78E+17	3.1E+16
sp|Q15582|	6	63	1.14481	2.29E+12	2E+12	########	1.38E+13	1.26E+14
sp|Q15599|	6	63	9.300364	4.42E+13	4.76E+12	########	2.65E+14	3E+14
sp|Q15631|	6	63	96.07545	1.38E+16	1.44E+14	########	8.29E+16	9.06E+15
sp|Q15691|	6	63	18.73549	1.47E+15	7.86E+13	########	8.84E+15	4.95E+15
sp|Q15700|	6	63	21.05593	2.25E+15	1.07E+14	########	1.35E+16	6.72E+15
sp|Q15714|	6	63	37.52448	1.84E+16	4.89E+14	########	1.1E+17	3.08E+16
sp|Q15738|	6	63	3.119914	1.29E+13	4.13E+12	########	7.74E+13	2.6E+14
sp|Q15746|	6	63	8.492708	1.51E+14	1.78E+13	########	9.07E+14	1.12E+15
sp|Q15750|	6	63	########	5.79E+11	5.85E+11	########	3.47E+12	3.68E+13
sp|Q15751|	6	63	2.528522	4.03E+12	1.59E+12	########	2.42E+13	1E+14
sp|Q15773|	6	63	7.266778	1.39E+15	1.92E+14	########	8.36E+15	1.21E+16
sp|Q15811|	6	63	10.54085	1.05E+15	9.99E+13	########	6.32E+15	6.3E+15
sp|Q15813|	6	63	3.011733	2.39E+13	7.93E+12	########	1.43E+14	4.99E+14
sp|Q15814|	6	63	########	1.15E+12	3.16E+12	########	6.9E+12	1.99E+14
sp|Q15818|	6	63	5.098999	1.38E+15	2.71E+14	########	8.3E+15	1.71E+16
sp|Q15819|	6	63	120.5181	5.19E+16	4.3E+14	########	3.11E+17	2.71E+16
sp|Q15843|	6	63	38.82724	2.28E+16	5.87E+14	########	1.37E+17	3.7E+16
sp|Q15847|	6	63	26.27887	1.61E+15	6.11E+13	########	9.63E+15	3.85E+15
sp|Q15904|	6	63	1.375933	1.22E+14	8.86E+13	########	7.32E+14	5.58E+15
sp|Q15907|	6	63	41.35064	2.73E+16	6.6E+14	########	1.64E+17	4.16E+16
sp|Q15942|	6	63	12.99779	1.86E+14	1.43E+13	########	1.11E+15	9E+14
sp|Q16134|	6	63	26.57004	1.35E+15	5.07E+13	########	8.08E+15	3.19E+15
sp|Q16143|	6	63	81.86977	6.14E+17	7.49E+15	########	4E+18	4.72E+17
sp|Q16181|	6	63	17.70093	7.41E+17	4.18E+16	########	4E+18	2.64E+18
sp|Q16204|	6	63	4.643493	9.72E+13	2.09E+13	########	5.83E+14	1.32E+15
sp|Q16222|	6	63	33.45444	5.85E+13	1.75E+12	########	3.51E+14	1.1E+14
sp|Q16352|	6	63	66.538	4E+19	6.68E+17	########	3E+20	4E+19
sp|Q16401|	6	63	8.974889	1.74E+14	1.93E+13	########	1.04E+15	1.22E+15
sp|Q16531|	6	63	195.3367	9.05E+16	4.63E+14	########	5.43E+17	2.92E+16
sp|Q16537|	6	63	7.82943	3.03E+14	3.87E+13	########	2E+15	2E+15
sp|Q16543|	6	63	54.38372	8.68E+15	1.6E+14	########	5.21E+16	1.01E+16
sp|Q16555|	6	63	124.7791	5E+20	3.62E+18	########	3E+21	2E+20
sp|Q16566|	6	63	3.810552	2.19E+13	5.75E+12	########	1.31E+14	3.62E+14
sp|Q16620|	6	63	2.062358	2.54E+14	1.23E+14	########	1.52E+15	7.75E+15
sp|Q16623|	6	63	6.409022	2.12E+17	3.31E+16	########	1.27E+18	2.08E+18

For Peer Review Only

sp|Q16629|	6	63	18.13682	4.2E+15	2.31E+14	########	2.52E+16	1.46E+16
sp|Q16630|	6	63	22.57361	1.68E+15	7.42E+13	########	1.01E+16	5E+15
sp|Q16643|	6	63	8.171945	1.48E+15	1.81E+14	########	8.87E+15	1.14E+16
sp|Q16653|	6	63	9.54427	2.03E+18	2.13E+17	########	1E+19	1E+19
sp|Q16658|	6	63	31.57681	4.82E+17	1.53E+16	########	3E+18	9.61E+17
sp|Q16698|	6	63	21.87446	3.13E+16	1.43E+15	########	1.88E+17	9E+16
sp|Q16718|	6	63	57.74623	1.61E+17	2.78E+15	########	9.65E+17	2E+17
sp|Q16720|	6	63	14.17674	3.47E+16	2.45E+15	########	2.08E+17	1.54E+17
sp|Q16762|	6	63	15.70796	2.46E+15	1.57E+14	########	1.48E+16	1E+16
sp|Q16773|	6	63	6.635444	2.14E+14	3.23E+13	########	1.29E+15	2.04E+15
sp|Q16774|	6	63	31.82134	2.81E+15	8.84E+13	########	1.69E+16	5.57E+15
sp|Q16775|	6	63	138.7455	4.51E+16	3.25E+14	########	3E+17	2.05E+16
sp|Q16777|	6	63	141.5315	1E+19	6.88E+16	########	########	4.33E+18
sp|Q16778|	6	63	192.0347	2E+19	9.32E+16	########	1E+20	6E+18
sp|Q16795|	6	63	74.52725	5.12E+17	6.87E+15	########	3E+18	4.33E+17
sp|Q16798|	6	63	45.03545	1.68E+17	3.72E+15	########	1.01E+18	2.34E+17
sp|Q16799|	6	63	3.367702	9.99E+15	2.97E+15	########	5.99E+16	2E+17
sp|Q16836|	6	63	45.34186	3.18E+16	7.02E+14	########	1.91E+17	4.42E+16
sp|Q16849|	6	63	4.990819	2.68E+14	5.37E+13	########	1.61E+15	3.38E+15
sp|Q16851|	6	63	74.01459	1.89E+17	2.55E+15	########	1.13E+18	1.61E+17
sp|Q16853|	6	63	3.789988	3.68E+14	9.7E+13	########	2.21E+15	6.11E+15
sp|Q16864|	6	63	2.521622	1.78E+15	7.04E+14	########	1.07E+16	4.44E+16
sp|Q16880|	6	63	4.966396	1.38E+14	2.79E+13	########	8.31E+14	1.76E+15
sp|Q16881|	6	63	5.24009	1E+15	2.05E+14	########	6E+15	1.29E+16
sp|Q16891|	6	63	95.11235	4.56E+17	4.79E+15	########	3E+18	3.02E+17
sp|Q17R89	6	63	45.72288	4.98E+14	1.09E+13	########	2.99E+15	6.86E+14
sp|Q1KMD3	6	63	39.60896	2.16E+16	5.47E+14	########	1.3E+17	3.44E+16
sp|Q27J81|	6	63	10.66455	6.03E+15	5.66E+14	########	3.62E+16	3.56E+16
sp|Q2M2I8|	6	63	11.07354	8.2E+16	7.4E+15	########	4.92E+17	4.66E+17
sp|Q2M389	6	63	5.580054	1.06E+14	1.89E+13	########	6.33E+14	1.19E+15
sp|Q2NKQ1	6	63	5.273661	9.9E+12	1.88E+12	########	5.94E+13	1.18E+14
sp|Q2TAA2	6	63	90.11995	1.07E+16	1.19E+14	########	6.42E+16	7.48E+15
sp|Q330K2	6	63	1.135434	8.65E+11	7.62E+11	########	5.19E+12	4.8E+13
sp|Q3KQU3	6	63	6.50605	1.38E+14	2.12E+13	########	8.29E+14	1E+15
sp|Q3KQV9	6	63	1.480384	5.55E+12	3.75E+12	########	3.33E+13	2.36E+14
sp|Q3LXA3	6	63	62.28531	6.92E+15	1.11E+14	########	4.15E+16	7E+15
sp|Q3SXM5	6	63	44.1334	1.18E+15	2.67E+13	########	7.07E+15	1.68E+15
sp|Q3YEC7	6	63	8.038217	1.81E+14	2.25E+13	########	1.09E+15	1.42E+15
sp|Q3ZCM7	6	63	14.99623	5E+18	3.66E+17	########	3E+19	2E+19
sp|Q3ZCQ8	6	63	41.9078	2.09E+15	4.99E+13	########	1.25E+16	3.14E+15
sp|Q3ZCW2	6	63	93.28595	1E+16	1.08E+14	########	6.03E+16	6.78E+15
sp|Q49A26	6	63	29.05863	8.01E+13	2.76E+12	########	4.81E+14	1.74E+14
sp|Q4G0F5	6	63	3.169504	5.1E+14	1.61E+14	########	3.06E+15	1E+16
sp|Q4G0N4	6	63	58.12067	3.25E+15	5.59E+13	########	1.95E+16	3.52E+15
sp|Q4G0P3	6	63	2.770563	6.83E+14	2.46E+14	########	4.1E+15	1.55E+16
sp|Q4G176	6	63	18.95921	1.81E+14	9.53E+12	########	1.08E+15	6E+14
sp|Q4J6C6	6	63	12.74101	2.15E+16	1.69E+15	########	1.29E+17	1E+17
sp|Q4KMP7	6	63	9.210373	1.23E+14	1.34E+13	########	7.41E+14	8.45E+14
sp|Q4KMQ2	6	63	7.911639	1.18E+15	1.5E+14	########	7.1E+15	9.43E+15
sp|Q4V328	6	63	32.52462	8.58E+14	2.64E+13	########	5.15E+15	2E+15
sp|Q504Y0	6	63	3.253813	2.57E+13	7.91E+12	########	1.54E+14	4.98E+14
sp|Q52LD8	6	63	########	2.23E+12	3.65E+12	########	1.34E+13	2.3E+14
sp|Q52LJ0|	6	63	23.2988	8.45E+14	3.63E+13	########	5.07E+15	2.28E+15
sp|Q53GQ0	6	63	61.7878	7.21E+16	1.17E+15	########	4.33E+17	7.35E+16
sp|Q53H12	6	63	30.88247	5.25E+16	1.7E+15	########	3.15E+17	1.07E+17
sp|Q53HC9	6	63	1.623936	1.24E+13	7.67E+12	########	7.47E+13	4.83E+14

For Peer Review Only

sp|Q562R1	6	63	########	4.32E+13	4.42E+13	########	2.59E+14	3E+15
sp|Q58FF8	6	63	########	4.87E+14	9.25E+14	########	2.92E+15	5.83E+16
sp|Q59EK9	6	63	11.1898	2.69E+14	2.4E+13	########	1.61E+15	2E+15
sp|Q5FWE	6	63	########	2.01E+13	2.08E+13	########	1.21E+14	1.31E+15
sp|Q5H9L2	6	63	11.87114	1.73E+15	1.46E+14	########	1.04E+16	9.19E+15
sp|Q5H9R7	6	63	1.309432	6.15E+10	4.7E+10	########	3.69E+11	2.96E+12
sp|Q5HYK	6	63	8.186514	2.82E+13	3.44E+12	########	1.69E+14	2.17E+14
sp|Q5JPH6	6	63	4.818616	1.97E+13	4.09E+12	########	1.18E+14	2.58E+14
sp|Q5JRX3	6	63	11.63942	7.04E+15	6.05E+14	########	4.22E+16	3.81E+16
sp|Q5JSH3	6	63	14.02284	1.55E+14	1.11E+13	########	9.33E+14	6.98E+14
sp|Q5JTD7	6	63	3.600963	1.32E+13	3.68E+12	########	7.95E+13	2.32E+14
sp|Q5JTJ3|	6	63	8.978377	4.97E+13	5.54E+12	########	2.98E+14	3.49E+14
sp|Q5JTZ9	6	63	3.155053	4.35E+12	1.38E+12	########	2.61E+13	8.68E+13
sp|Q5JU85	6	63	42.13771	1.2E+15	2.85E+13	########	7.2E+15	2E+15
sp|Q5RI15|	6	63	3.524938	1.6E+13	4.55E+12	########	9.63E+13	2.87E+14
sp|Q5SQI0	6	63	28.72182	1.84E+14	6.39E+12	########	1.1E+15	4.03E+14
sp|Q5SRE7	6	63	1.035711	1.04E+13	1E+13	########	6.23E+13	6.32E+14
sp|Q5SSJ5	6	63	35.3984	3.2E+16	9.03E+14	########	########	5.69E+16
sp|Q5SYC	6	63	5.254	3.42E+13	6.5E+12	########	2.05E+14	4.1E+14
sp|Q5T0D9	6	63	5.628724	6.53E+15	1.16E+15	########	3.92E+16	7.31E+16
sp|Q5T440	6	63	14.0387	1.03E+14	7.37E+12	########	6.21E+14	4.64E+14
sp|Q5T4S7	6	63	59.90751	6.7E+15	1.12E+14	########	4.02E+16	7.04E+15
sp|Q5T5C0	6	63	8.228981	7.26E+14	8.82E+13	########	4.35E+15	5.56E+15
sp|Q5T5P2	6	63	4.011419	3.53E+13	8.81E+12	########	2.12E+14	5.55E+14
sp|Q5T848	6	63	4.067909	3.67E+14	9.03E+13	########	2.2E+15	5.69E+15
sp|Q5TC12	6	63	8.25238	1.43E+14	1.73E+13	########	8.55E+14	1.09E+15
sp|Q5TCZ1	6	63	2.327117	1.77E+12	7.61E+11	########	1.06E+13	4.79E+13
sp|Q5TF21	6	63	21.6709	1.06E+15	4.9E+13	########	6.37E+15	3.09E+15
sp|Q5TFE4	6	63	40.93425	4.9E+14	1.2E+13	########	2.94E+15	7.54E+14
sp|Q5TGZ0	6	63	8.660041	3.29E+14	3.8E+13	########	1.98E+15	2.4E+15
sp|Q5TH69	6	63	46.70711	6.81E+14	1.46E+13	########	4.09E+15	9.19E+14
sp|Q5TZA2	6	63	1.120046	1.2E+12	1.07E+12	########	7.19E+12	6.74E+13
sp|Q5U4P2	6	63	6.234166	8.17E+12	1.31E+12	########	4.9E+13	8.26E+13
sp|Q5VIR6|	6	63	19.108	1.17E+14	6.1E+12	########	6.99E+14	3.84E+14
sp|Q5VSL9	6	63	9.623388	2.14E+13	2.22E+12	########	1.28E+14	1.4E+14
sp|Q5VST6	6	63	5.9974	6.94E+13	1.16E+13	########	4.16E+14	7.29E+14
sp|Q5VT25	6	63	3.301144	9.3E+12	2.82E+12	########	5.58E+13	1.78E+14
sp|Q5VT66	6	63	1.205977	9.31E+11	7.72E+11	########	5.59E+12	4.86E+13
sp|Q5VUB5	6	63	1.324238	1.32E+13	1E+13	########	7.94E+13	6.3E+14
sp|Q5VW32	6	63	1.2685	4.7E+13	3.71E+13	########	2.82E+14	2.34E+15
sp|Q5VWJ9	6	63	7.393919	1.04E+14	1.41E+13	########	6.25E+14	8.88E+14
sp|Q5VWQ	6	63	5.520902	9.08E+11	1.65E+11	########	5.45E+12	1.04E+13
sp|Q5VWZ2	6	63	6.242395	4.34E+13	6.95E+12	########	2.6E+14	4.38E+14
sp|Q5VYK3	6	63	17.25745	2.37E+14	1.37E+13	########	1.42E+15	8.66E+14
sp|Q5VZ66	6	63	2.266804	9.75E+09	4.3E+09	########	5.85E+10	2.71E+11
sp|Q5VZK9	6	63	3.449885	1.73E+12	5.02E+11	########	1.04E+13	3.16E+13
sp|Q5XKP0	6	63	34.52534	6.06E+15	1.76E+14	########	3.64E+16	1.11E+16
sp|Q63HM9	6	63	8.673733	6.08E+14	7.01E+13	########	3.65E+15	4.42E+15
sp|Q643R3	6	63	8.792639	2.81E+14	3.19E+13	########	1.68E+15	2.01E+15
sp|Q66K14	6	63	14.4086	5.08E+13	3.53E+12	########	3.05E+14	2.22E+14
sp|Q66K74	6	63	24.45407	8.05E+15	3.29E+14	########	4.83E+16	2.07E+16
sp|Q684P5	6	63	11.98981	1.76E+14	1.47E+13	########	1.06E+15	9.24E+14
sp|Q68CZ2	6	63	1.502996	1.95E+13	1.3E+13	########	1.17E+14	8.17E+14
sp|Q68D91	6	63	9.159356	3.99E+14	4.35E+13	########	2.39E+15	2.74E+15
sp|Q68DH5	6	63	5.390105	5E+13	9.27E+12	########	3E+14	5.84E+14
sp|Q68DU8	6	63	1.744408	4.33E+13	2.48E+13	########	2.6E+14	1.56E+15

For Peer Review Only

sp|Q6BCY4	6	63	19.23185	1.01E+16	5.26E+14	########	6.07E+16	3.31E+16
sp|Q6DD88	6	63	5.245308	4.34E+14	8.26E+13	########	2.6E+15	5.21E+15
sp|Q6DN90	6	63	77.62024	8.41E+15	1.08E+14	########	5.05E+16	6.83E+15
sp|Q6FI81|	6	63	21.71858	7.3E+13	3.36E+12	########	4.38E+14	2.12E+14
sp|Q6GMV	6	63	10.35791	2.92E+15	2.81E+14	########	1.75E+16	1.77E+16
sp|Q6H8Q1	6	63	16.92583	4.37E+14	2.58E+13	########	2.62E+15	2E+15
sp|Q6IAA8|	6	63	28.5595	3.12E+15	1.09E+14	########	1.87E+16	6.89E+15
sp|Q6IAN0|	6	63	9.122829	6.26E+12	6.86E+11	########	3.76E+13	4.32E+13
sp|Q6IBS0|	6	63	8.440584	1.23E+15	1.46E+14	########	7.38E+15	9.19E+15
sp|Q6IQ20|	6	63	2.580104	4.51E+13	1.75E+13	########	2.71E+14	1.1E+15
sp|Q6IQ22|	6	63	########	4.15E+11	9.46E+11	########	2.49E+12	5.96E+13
sp|Q6KCM1	6	63	29.55259	8.53E+14	2.89E+13	########	5.12E+15	1.82E+15
sp|Q6L8Q7	6	63	6.108849	7.69E+13	1.26E+13	########	4.61E+14	7.93E+14
sp|Q6NUK1	6	63	53.8005	1.29E+14	2.4E+12	########	7.74E+14	1.51E+14
sp|Q6NV74	6	63	########	9.98E+10	2.17E+11	########	5.99E+11	1.37E+13
sp|Q6NVY1	6	63	24.82427	2.55E+15	1.03E+14	########	1.53E+16	6E+15
sp|Q6NXS1	6	63	156.7382	5.43E+13	3.47E+11	########	3.26E+14	2.18E+13
sp|Q6NZI2|	6	63	49.40195	1.37E+15	2.78E+13	########	8.25E+15	1.75E+15
sp|Q6P1X6	6	63	7.985182	6.81E+12	8.53E+11	########	4.09E+13	5.37E+13
sp|Q6P2E9	6	63	2.455932	1.79E+14	7.3E+13	########	1.08E+15	4.6E+15
sp|Q6P2Q9	6	63	43.57084	4.43E+15	1.02E+14	########	2.66E+16	6.41E+15
sp|Q6P3W7	6	63	8.007944	3.71E+14	4.64E+13	########	2.23E+15	2.92E+15
sp|Q6P4A7	6	63	8.049122	4.02E+12	5E+11	########	2.41E+13	3.15E+13
sp|Q6P587	6	63	11.42102	1.07E+16	9.41E+14	########	6.45E+16	5.93E+16
sp|Q6P995	6	63	2.665722	9.78E+12	3.67E+12	########	5.87E+13	2.31E+14
sp|Q6P9B6	6	63	3.812603	4.79E+13	1.26E+13	########	2.87E+14	7.91E+14
sp|Q6PCB7	6	63	7.31218	5.57E+14	7.62E+13	########	3.34E+15	5E+15
sp|Q6PCE3	6	63	59.83717	4.61E+16	7.71E+14	########	2.77E+17	4.86E+16
sp|Q6PFW	6	63	7.270824	4.84E+12	6.65E+11	########	2.9E+13	4.19E+13
sp|Q6PGP7	6	63	1.66532	1.22E+13	7.3E+12	########	7.29E+13	4.6E+14
sp|Q6PI78|	6	63	14.15243	1.29E+14	9.1E+12	########	7.72E+14	5.73E+14
sp|Q6PIU2|	6	63	31.02235	6.32E+15	2.04E+14	########	3.79E+16	1.28E+16
sp|Q6PJW8	6	63	########	3.95E+12	4.58E+12	########	2.37E+13	2.88E+14
sp|Q6PKG0	6	63	10.6336	1.26E+14	1.19E+13	########	7.56E+14	7.47E+14
sp|Q6PUV4	6	63	1.950431	1.5E+16	7.68E+15	########	8.99E+16	4.84E+17
sp|Q6U841	6	63	11.31326	4.86E+15	4.29E+14	########	2.91E+16	2.7E+16
sp|Q6UW68	6	63	7.087837	4.98E+13	7.03E+12	########	2.99E+14	4.43E+14
sp|Q6UWE	6	63	10.85763	9.04E+13	8.33E+12	########	5.42E+14	5.25E+14
sp|Q6UWP	6	63	16.66277	6.52E+13	3.91E+12	########	3.91E+14	2.46E+14
sp|Q6UWR	6	63	9.320305	3.17E+17	3.4E+16	########	1.9E+18	2.14E+18
sp|Q6UXD9	6	63	2.256235	2.13E+13	9.45E+12	########	1.28E+14	5.95E+14
sp|Q6UXV4	6	63	13.75035	1.31E+14	9.52E+12	########	7.86E+14	6E+14
sp|Q6VY07	6	63	7.416088	2.18E+14	2.95E+13	########	1.31E+15	1.86E+15
sp|Q6WCQ1	6	63	2.769245	3.35E+13	1.21E+13	########	2.01E+14	7.62E+14
sp|Q6XQN6	6	63	13.15383	1.39E+14	1.06E+13	########	8.36E+14	6.68E+14
sp|Q6Y7W6	6	63	5.358639	1.08E+13	2.02E+12	########	6.51E+13	1.28E+14
sp|Q6YN16	6	63	16.29225	3.89E+15	2.39E+14	########	2.34E+16	1.5E+16
sp|Q6YP21	6	63	8.966195	6.27E+14	6.99E+13	########	3.76E+15	4.41E+15
sp|Q6ZMI0	6	63	2.319219	1.04E+13	4.5E+12	########	6.27E+13	2.84E+14
sp|Q6ZSS7	6	63	8.181564	1.68E+14	2.06E+13	########	1.01E+15	1.3E+15
sp|Q6ZT12	6	63	3.550463	3.89E+12	1.1E+12	########	2.33E+13	6.9E+13
sp|Q6ZVL6	6	63	4.122489	5.25E+12	1.27E+12	########	3.15E+13	8.02E+13
sp|Q6ZVM7	6	63	2.643975	2.5E+15	9.45E+14	########	1.5E+16	5.96E+16
sp|Q709C8	6	63	17.68576	5.97E+14	3.38E+13	########	3.58E+15	2.13E+15
sp|Q71DI3|	6	63	63.16746	9.31E+13	1.47E+12	########	5.59E+14	9.29E+13
sp|Q71U36	6	63	16.30749	1.37E+18	8.41E+16	########	8E+18	5E+18

For Peer Review Only

sp|Q71UI9|	6	63	33.16468	1.24E+17	3.73E+15	########	7E+17	2.35E+17
sp|Q71UM5	6	63	7.906988	1.06E+14	1.34E+13	########	6.34E+14	8.41E+14
sp|Q765P7	6	63	9.829617	1.71E+14	1.74E+13	########	1.02E+15	1.09E+15
sp|Q7KZF4	6	63	9.928308	3.02E+15	3.04E+14	########	1.81E+16	2E+16
sp|Q7KZN9	6	63	2.056576	8.61E+12	4.19E+12	########	5.17E+13	2.64E+14
sp|Q7L099|	6	63	50.83456	4.75E+16	9.35E+14	########	2.85E+17	5.89E+16
sp|Q7L0J3|	6	63	10.06923	9.45E+16	9.39E+15	########	6E+17	5.91E+17
sp|Q7L1I2|	6	63	11.22947	1.72E+16	1.53E+15	########	########	9.64E+16
sp|Q7L1Q6	6	63	1.988007	1.61E+13	8.1E+12	########	9.66E+13	5.1E+14
sp|Q7L1S5	6	63	6.38122	4.77E+14	7.47E+13	########	2.86E+15	4.71E+15
sp|Q7L266|	6	63	95.39489	4.24E+16	4.45E+14	########	2.55E+17	2.8E+16
sp|Q7L2E3	6	63	6.902991	5.28E+13	7.65E+12	########	3.17E+14	4.82E+14
sp|Q7L2H7	6	63	4.651458	3.91E+14	8.42E+13	########	2.35E+15	5.3E+15
sp|Q7L3T8	6	63	6.554115	1.21E+12	1.84E+11	########	7.25E+12	1.16E+13
sp|Q7L523|	6	63	5.435259	2.77E+13	5.1E+12	########	1.66E+14	3.21E+14
sp|Q7L576|	6	63	13.19518	5.78E+14	4.38E+13	########	3.47E+15	3E+15
sp|Q7L5N1	6	63	4.334544	6.32E+14	1.46E+14	########	3.79E+15	9E+15
sp|Q7L5N7	6	63	1.626488	1.83E+13	1.12E+13	########	1.1E+14	7.08E+14
sp|Q7L775|	6	63	17.91339	2.08E+14	1.16E+13	########	1.25E+15	7.32E+14
sp|Q7Z2K8	6	63	8.161384	2.2E+13	2.7E+12	########	1.32E+14	1.7E+14
sp|Q7Z392	6	63	2.368417	8.71E+11	3.68E+11	########	5.22E+12	2.32E+13
sp|Q7Z3B1	6	63	8.979771	1.24E+16	1.39E+15	########	7.47E+16	9E+16
sp|Q7Z3D6	6	63	6.842538	7.93E+15	1.16E+15	########	4.76E+16	7E+16
sp|Q7Z3J2|	6	63	15.17355	2.75E+13	1.81E+12	########	1.65E+14	1.14E+14
sp|Q7Z3U7	6	63	3.901905	1.04E+13	2.66E+12	########	6.23E+13	1.68E+14
sp|Q7Z406	6	63	11.55424	1.2E+15	1.04E+14	########	7.18E+15	6.53E+15
sp|Q7Z460	6	63	22.93903	1.08E+15	4.71E+13	########	6.49E+15	2.97E+15
sp|Q7Z4H3	6	63	16.6084	1.21E+14	7.28E+12	########	7.25E+14	4.58E+14
sp|Q7Z4S6	6	63	45.57323	2.91E+16	6.38E+14	########	########	4.02E+16
sp|Q7Z4W1	6	63	15.69607	3.39E+15	2.16E+14	########	2.03E+16	1.36E+16
sp|Q7Z6G3	6	63	2.222141	8.77E+14	3.95E+14	########	5.26E+15	2E+16
sp|Q7Z6G8	6	63	22.44624	9.24E+14	4.12E+13	########	5.54E+15	2.59E+15
sp|Q7Z6L0	6	63	4.73159	1.76E+16	3.71E+15	########	1.05E+17	2.34E+17
sp|Q7Z6Z7	6	63	13.55601	2.35E+16	1.73E+15	########	1.41E+17	1.09E+17
sp|Q7Z7A4	6	63	3.60641	2.41E+13	6.67E+12	########	1.44E+14	4.2E+14
sp|Q7Z7H8	6	63	7.603228	7.46E+12	9.82E+11	########	4.48E+13	6.18E+13
sp|Q7Z7L7	6	63	3.258625	3.07E+12	9.43E+11	########	1.84E+13	5.94E+13
sp|Q86SK9	6	63	3.398168	8.11E+12	2.39E+12	########	4.86E+13	1.5E+14
sp|Q86SX6	6	63	30.00489	7.92E+14	2.64E+13	########	4.75E+15	1.66E+15
sp|Q86SZ2	6	63	########	7.68E+12	1.02E+13	########	4.61E+13	6.43E+14
sp|Q86T65	6	63	7.203872	1.77E+15	2.46E+14	########	1.06E+16	1.55E+16
sp|Q86TP1	6	63	11.76976	1.08E+14	9.18E+12	########	6.48E+14	5.78E+14
sp|Q86TV6	6	63	19.49801	2.33E+14	1.19E+13	########	1.4E+15	7.52E+14
sp|Q86TX2	6	63	52.77686	2.93E+15	5.56E+13	########	1.76E+16	3.5E+15
sp|Q86UP2	6	63	3.308062	3.85E+14	1.16E+14	########	2.31E+15	7.33E+15
sp|Q86UR5	6	63	2.152499	3.12E+12	1.45E+12	########	1.87E+13	9.12E+13
sp|Q86UW1	6	63	9.455797	9.16E+13	9.69E+12	########	5.5E+14	6.1E+14
sp|Q86UW8	6	63	3.720906	3.9E+15	1.05E+15	########	2.34E+16	6.61E+16
sp|Q86UX6	6	63	12.49549	2.37E+14	1.9E+13	########	1.42E+15	1E+15
sp|Q86UY8	6	63	11.46454	5.97E+14	5.21E+13	########	3.58E+15	3E+15
sp|Q86V81	6	63	9.261365	8.24E+13	8.89E+12	########	4.94E+14	5.6E+14
sp|Q86V88	6	63	11.7465	4.49E+13	3.82E+12	########	2.69E+14	2.41E+14
sp|Q86VP6	6	63	194.2483	9.62E+17	4.95E+15	########	6E+18	3.12E+17
sp|Q86VS8	6	63	3.28235	3.48E+12	1.06E+12	########	2.09E+13	6.68E+13
sp|Q86VU5	6	63	11.30888	1.44E+14	1.27E+13	########	8.63E+14	8.02E+14
sp|Q86VW0	6	63	10.99864	6.83E+14	6.21E+13	########	4.1E+15	3.91E+15

For Peer Review Only

sp|Q86WA(	6	63	2.93439	3.98E+13	1.36E+13	########	2.39E+14	8.55E+14
sp|Q86WG	6	63	11.76034	1.02E+15	8.65E+13	########	6.1E+15	5E+15
sp|Q86WU	6	63	11.07247	1.02E+14	9.21E+12	########	6.12E+14	5.8E+14
sp|Q86X10	6	63	8.706222	3.89E+14	4.47E+13	########	2.33E+15	2.82E+15
sp|Q86X55	6	63	########	2.74E+12	4.11E+12	########	1.64E+13	2.59E+14
sp|Q86X76	6	63	16.10872	7.91E+14	4.91E+13	########	4.75E+15	3.09E+15
sp|Q86XE3	6	63	11.65963	1.64E+13	1.41E+12	########	9.83E+13	8.85E+13
sp|Q86Y39	6	63	21.0633	1.45E+16	6.9E+14	########	8.71E+16	4.34E+16
sp|Q86Y82	6	63	7.033098	2.59E+14	3.68E+13	########	1.55E+15	2.32E+15
sp|Q86YM7	6	63	20.54918	7.64E+14	3.72E+13	########	4.58E+15	2.34E+15
sp|Q86YN1	6	63	6.694105	2.82E+12	4.22E+11	########	1.69E+13	2.66E+13
sp|Q86YQ8	6	63	1.421751	7.3E+11	5.14E+11	########	4.38E+12	3.24E+13
sp|Q86YS7	6	63	1.274584	3.17E+12	2.49E+12	########	1.9E+13	1.57E+14
sp|Q8IU85|	6	63	26.89951	2.46E+15	9.15E+13	########	1.48E+16	5.77E+15
sp|Q8IUR0	6	63	5.710277	2.02E+13	3.53E+12	########	1.21E+14	2.22E+14
sp|Q8IUR7	6	63	14.04185	6.19E+13	4.41E+12	########	3.71E+14	2.78E+14
sp|Q8IUS5|	6	63	1.124276	1.03E+11	9.19E+10	########	6.2E+11	5.79E+12
sp|Q8IV01|	6	63	6.647209	1.06E+15	1.6E+14	########	6.39E+15	1.01E+16
sp|Q8IV08|	6	63	5.636486	1.86E+14	3.31E+13	########	1.12E+15	2E+15
sp|Q8IV38|	6	63	1.421833	5.72E+12	4.02E+12	########	3.43E+13	2.53E+14
sp|Q8IVD9|	6	63	7.047332	1.66E+14	2.36E+13	########	9.97E+14	1.49E+15
sp|Q8IVF2|	6	63	3.560054	9.37E+13	2.63E+13	########	5.62E+14	1.66E+15
sp|Q8IVP5|	6	63	2.968948	7.77E+12	2.62E+12	########	4.66E+13	1.65E+14
sp|Q8IW45	6	63	49.62702	1.11E+16	2.25E+14	########	6.69E+16	1.42E+16
sp|Q8IWA5	6	63	51.35167	1.29E+16	2.52E+14	########	7.77E+16	1.59E+16
sp|Q8IWB7	6	63	3.759661	5.03E+13	1.34E+13	########	3.02E+14	8.43E+14
sp|Q8IWQ3	6	63	23.11434	3.89E+14	1.68E+13	########	2.33E+15	1.06E+15
sp|Q8IWT6	6	63	27.76291	2.04E+15	7.35E+13	########	1.22E+16	4.63E+15
sp|Q8IXI2|M	6	63	45.9299	1.09E+15	2.36E+13	########	6.52E+15	1.49E+15
sp|Q8IXJ6|	6	63	7.820204	7.22E+17	9.23E+16	########	4E+18	6E+18
sp|Q8IXS8|	6	63	6.089937	1.22E+14	2E+13	########	7.31E+14	1.26E+15
sp|Q8IY17|	6	63	16.70072	3.06E+14	1.83E+13	########	1.83E+15	1.15E+15
sp|Q8IY22|	6	63	3.158313	1.84E+13	5.82E+12	########	1.1E+14	3.67E+14
sp|Q8IYB4|	6	63	12.38382	6.43E+14	5.19E+13	########	3.86E+15	3.27E+15
sp|Q8IYB5|	6	63	17.5266	2.44E+15	1.39E+14	########	1.46E+16	8.78E+15
sp|Q8IYI6|E	6	63	4.839015	7.87E+13	1.63E+13	########	4.72E+14	1.02E+15
sp|Q8IYJ1|	6	63	########	1.29E+11	2.72E+11	########	7.75E+11	1.72E+13
sp|Q8IYQ7	6	63	5.282625	4.5E+13	8.53E+12	########	2.7E+14	5.37E+14
sp|Q8IZ83|	6	63	8.436672	5.34E+13	6.32E+12	########	3.2E+14	3.98E+14
sp|Q8IZD9|	6	63	12.34681	1.09E+14	8.81E+12	########	6.53E+14	5.55E+14
sp|Q8IZP0|	6	63	41.1761	2.28E+15	5.53E+13	########	1.37E+16	3.49E+15
sp|Q8IZS8|	6	63	3.819493	1.37E+14	3.58E+13	########	8.2E+14	2.25E+15
sp|Q8IZU2|	6	63	2.888262	1.16E+14	4.02E+13	########	6.97E+14	2.53E+15
sp|Q8N0X7	6	63	11.39024	2.39E+14	2.1E+13	########	1.43E+15	1.32E+15
sp|Q8N111	6	63	18.79159	2.23E+16	1.19E+15	########	1.34E+17	7.47E+16
sp|Q8N122	6	63	5.489987	7.52E+12	1.37E+12	########	4.51E+13	8.63E+13
sp|Q8N126	6	63	15.88267	1.41E+17	8.86E+15	########	8.45E+17	5.58E+17
sp|Q8N135	6	63	7.192232	1.32E+14	1.83E+13	########	7.9E+14	1.15E+15
sp|Q8N145	6	63	7.524618	1.15E+16	1.53E+15	########	6.91E+16	9.64E+16
sp|Q8N163	6	63	16.10634	2.41E+14	1.5E+13	########	1.45E+15	9.44E+14
sp|Q8N183	6	63	19.71752	6.2E+14	3.14E+13	########	3.72E+15	1.98E+15
sp|Q8N1AC	6	63	12.8612	1.63E+13	1.26E+12	########	9.76E+13	7.97E+13
sp|Q8N1B4	6	63	8.317263	2.23E+14	2.68E+13	########	1.34E+15	1.69E+15
sp|Q8N1F7	6	63	6.979179	9.63E+12	1.38E+12	########	5.78E+13	8.69E+13
sp|Q8N1G4	6	63	14.53567	4.85E+15	3.34E+14	########	2.91E+16	2E+16
sp|Q8N1I0|	6	63	18.2071	1.76E+14	9.69E+12	########	1.06E+15	6.1E+14

For Peer Review Only

sp|Q8N2F6	6	63	19.55243	5.7E+14	2.91E+13	########	3.42E+15	1.84E+15
sp|Q8N2K0	6	63	13.49134	4.22E+14	3.13E+13	########	2.53E+15	1.97E+15
sp|Q8N335	6	63	32.89505	5.52E+15	1.68E+14	########	3.31E+16	1E+16
sp|Q8N3E9	6	63	16.07415	1.47E+15	9.16E+13	########	8.84E+15	5.77E+15
sp|Q8N3F0	6	63	7.699763	1.57E+15	2.03E+14	########	9.39E+15	1.28E+16
sp|Q8N3J6	6	63	26.58486	4.03E+17	1.52E+16	########	2.42E+18	9.55E+17
sp|Q8N3P4	6	63	9.311077	3.91E+12	4.19E+11	########	2.34E+13	2.64E+13
sp|Q8N3V7	6	63	9.405732	7.75E+15	8.24E+14	########	4.65E+16	5E+16
sp|Q8N414	6	63	2.876549	4.07E+12	1.42E+12	########	2.44E+13	8.92E+13
sp|Q8N461	6	63	5.514331	1.19E+14	2.15E+13	########	7.13E+14	1.36E+15
sp|Q8N465	6	63	1.645768	8.33E+13	5.06E+13	########	5E+14	3.19E+15
sp|Q8N468	6	63	2.183732	5.63E+11	2.58E+11	########	3.38E+12	1.62E+13
sp|Q8N4C8	6	63	14.82881	2.44E+14	1.65E+13	########	1.47E+15	1.04E+15
sp|Q8N4P6	6	63	1.667156	4.8E+14	2.88E+14	########	2.88E+15	1.81E+16
sp|Q8N4Q0	6	63	14.18343	7.41E+13	5.23E+12	########	4.45E+14	3.29E+14
sp|Q8N4V2	6	63	2.72577	2.92E+12	1.07E+12	########	1.75E+13	6.74E+13
sp|Q8N568	6	63	27.06182	2.56E+15	9.45E+13	########	1.53E+16	5.95E+15
sp|Q8N573	6	63	60.35473	9.65E+16	1.6E+15	########	5.79E+17	1.01E+17
sp|Q8N5H7	6	63	1.56681	6.82E+12	4.35E+12	########	4.09E+13	2.74E+14
sp|Q8N5J2	6	63	2.198763	8.07E+13	3.67E+13	########	4.84E+14	2.31E+15
sp|Q8N5K1	6	63	1.91728	2.63E+13	1.37E+13	########	1.58E+14	8.65E+14
sp|Q8N5R6	6	63	1.206084	1.64E+14	1.36E+14	########	9.83E+14	8.56E+15
sp|Q8N5S9	6	63	26.50795	1.22E+15	4.6E+13	########	7.31E+15	2.9E+15
sp|Q8N5V2	6	63	5.20774	8.37E+13	1.61E+13	########	5.02E+14	1.01E+15
sp|Q8N608	6	63	12.76644	2.42E+15	1.9E+14	########	1.45E+16	1.2E+16
sp|Q8N668	6	63	3.015494	9.79E+12	3.25E+12	########	5.87E+13	2.04E+14
sp|Q8N684	6	63	8.006264	1.21E+13	1.51E+12	########	7.26E+13	9.52E+13
sp|Q8N6C5	6	63	4.679579	1.35E+14	2.88E+13	########	8.07E+14	1.81E+15
sp|Q8N6M0	6	63	5.428249	7.34E+12	1.35E+12	########	4.4E+13	8.51E+13
sp|Q8N6N7	6	63	27.9665	4.9E+15	1.75E+14	########	2.94E+16	1.1E+16
sp|Q8N6T3	6	63	5.516191	2.26E+14	4.09E+13	########	1.35E+15	2.58E+15
sp|Q8N766	6	63	15.97037	3.84E+14	2.4E+13	########	2.3E+15	1.51E+15
sp|Q8N7J2	6	63	3.700323	2.24E+14	6.06E+13	########	1.35E+15	3.82E+15
sp|Q8N8N7	6	63	75.19811	3.97E+15	5.27E+13	########	2.38E+16	3.32E+15
sp|Q8N8S7	6	63	12.27575	3.09E+13	2.52E+12	########	1.86E+14	1.59E+14
sp|Q8N8Y2	6	63	3.729533	5.57E+15	1.49E+15	########	3.34E+16	9E+16
sp|Q8N987	6	63	1.167228	1.63E+14	1.4E+14	########	9.78E+14	9E+15
sp|Q8N9F0	6	63	4.543791	1.11E+12	2.44E+11	########	6.66E+12	1.54E+13
sp|Q8N9F7	6	63	4.840729	4.62E+13	9.54E+12	########	2.77E+14	6.01E+14
sp|Q8N9I0	6	63	2.830153	1.22E+16	4.31E+15	########	7.33E+16	2.72E+17
sp|Q8N9N7	6	63	11.96673	9.28E+14	7.75E+13	########	5.57E+15	5E+15
sp|Q8N9R8	6	63	12.28626	1.55E+15	1.26E+14	########	9.3E+15	7.95E+15
sp|Q8NB37	6	63	2.468106	1.26E+14	5.09E+13	########	7.54E+14	3.21E+15
sp|Q8NBF2	6	63	3.814734	1.8E+12	4.71E+11	########	1.08E+13	2.97E+13
sp|Q8NBF6	6	63	1.060476	3.92E+12	3.7E+12	########	2.35E+13	2.33E+14
sp|Q8NBM8	6	63	6.24126	1.28E+14	2.05E+13	########	7.68E+14	1.29E+15
sp|Q8NBN7	6	63	29.07075	1.6E+14	5.49E+12	########	9.57E+14	3.46E+14
sp|Q8NBQ5	6	63	7.961338	9.69E+12	1.22E+12	########	5.82E+13	7.67E+13
sp|Q8NBS9	6	63	9.998662	4.41E+14	4.41E+13	########	2.65E+15	2.78E+15
sp|Q8NBU5	6	63	27.27947	3.87E+14	1.42E+13	########	2.32E+15	8.94E+14
sp|Q8NBX0	6	63	46.57155	6.18E+16	1.33E+15	########	3.71E+17	8.36E+16
sp|Q8NC51	6	63	14.15317	1.1E+15	7.8E+13	########	6.62E+15	4.91E+15
sp|Q8NC96	6	63	5.320764	2.27E+15	4.27E+14	########	1.36E+16	2.69E+16
sp|Q8NCA5	6	63	2.643021	3.55E+13	1.34E+13	########	2.13E+14	8.45E+14
sp|Q8NCB2	6	63	24.42543	6.75E+16	2.76E+15	########	4.05E+17	1.74E+17
sp|Q8NCG7	6	63	10.45951	1.28E+15	1.22E+14	########	7.68E+15	7.71E+15

For Peer Review Only

sp|Q8NCW	6	63	170.6857	4.17E+16	2.44E+14	########	2.5E+17	1.54E+16
sp|Q8ND24	6	63	1.125964	3.78E+11	3.36E+11	########	2.27E+12	2.11E+13
sp|Q8ND76	6	63	1.999449	1.24E+14	6.19E+13	########	7.43E+14	3.9E+15
sp|Q8NDA8	6	63	8.170923	3.75E+14	4.59E+13	########	2.25E+15	2.89E+15
sp|Q8NDH3	6	63	3.346238	7.64E+12	2.28E+12	########	4.58E+13	1.44E+14
sp|Q8NE01	6	63	4.103899	6.25E+11	1.52E+11	########	3.75E+12	9.6E+12
sp|Q8NE62	6	63	9.664867	4.8E+14	4.97E+13	########	2.88E+15	3E+15
sp|Q8NE71	6	63	7.597487	5.73E+13	7.54E+12	########	3.44E+14	4.75E+14
sp|Q8NE86	6	63	3.211266	7.6E+13	2.37E+13	########	4.56E+14	1.49E+15
sp|Q8NEB9	6	63	2.587079	9.9E+11	3.83E+11	########	5.94E+12	2.41E+13
sp|Q8NEU8	6	63	8.110432	9.03E+13	1.11E+13	########	5.42E+14	7.02E+14
sp|Q8NF37	6	63	3.554581	1.56E+13	4.39E+12	########	9.35E+13	2.76E+14
sp|Q8NF91	6	63	7.88602	8.94E+14	1.13E+14	########	5.36E+15	7.14E+15
sp|Q8NFJ9	6	63	5.792758	1.48E+13	2.56E+12	########	8.9E+13	1.61E+14
sp|Q8NFP9	6	63	14.61787	1.16E+14	7.91E+12	########	6.94E+14	4.98E+14
sp|Q8NFU3	6	63	4.578472	3.43E+13	7.5E+12	########	2.06E+14	4.73E+14
sp|Q8NFV4	6	63	19.62001	1.04E+15	5.31E+13	########	6.25E+15	3.34E+15
sp|Q8NFW1	6	63	31.54963	3.01E+15	9.53E+13	########	1.8E+16	6E+15
sp|Q8NFX7	6	63	16.74347	1.96E+14	1.17E+13	########	1.18E+15	7.37E+14
sp|Q8NFZ4	6	63	4.768207	1.05E+13	2.2E+12	########	6.28E+13	1.38E+14
sp|Q8NFZ8	6	63	8.752598	2.57E+16	2.94E+15	########	1.54E+17	2E+17
sp|Q8NHG7	6	63	2.361367	2.28E+14	9.67E+13	########	1.37E+15	6.09E+15
sp|Q8NHH9	6	63	2.150694	9.59E+12	4.46E+12	########	5.75E+13	2.81E+14
sp|Q8NHM1	6	63	2.049606	1.75E+15	8.52E+14	########	1.05E+16	5.37E+16
sp|Q8NHP6	6	63	3.734815	1.7E+13	4.54E+12	########	1.02E+14	2.86E+14
sp|Q8TAC2	6	63	########	1.47E+12	1.5E+12	########	8.84E+12	9.45E+13
sp|Q8TAC9	6	63	6.10152	1.42E+15	2.33E+14	########	8.53E+15	1.47E+16
sp|Q8TAF3	6	63	3.271021	1.74E+13	5.31E+12	########	1.04E+14	3.34E+14
sp|Q8TAM6	6	63	1.966617	1.71E+15	8.71E+14	########	1.03E+16	5.49E+16
sp|Q8TAT6	6	63	1.368337	7.22E+13	5.27E+13	########	4.33E+14	3E+15
sp|Q8TB36	6	63	44.87421	########	2.34E+15	########	6.31E+17	1E+17
sp|Q8TB96	6	63	7.443589	2.04E+14	2.73E+13	########	1.22E+15	1.72E+15
sp|Q8TBB6	6	63	1.959771	8.67E+12	4.42E+12	########	5.2E+13	2.79E+14
sp|Q8TBC4	6	63	23.32159	4.42E+15	1.9E+14	########	2.65E+16	1E+16
sp|Q8TBF2	6	63	4.701839	1.02E+14	2.17E+13	########	6.13E+14	1E+15
sp|Q8TBG9	6	63	5.940625	5.33E+14	8.98E+13	########	3.2E+15	5.66E+15
sp|Q8TBX8	6	63	35.56079	2.72E+15	7.64E+13	########	1.63E+16	4.81E+15
sp|Q8TC12	6	63	18.46905	4.11E+14	2.22E+13	########	2.46E+15	1E+15
sp|Q8TCD5	6	63	35.29358	6.27E+14	1.78E+13	########	3.76E+15	1.12E+15
sp|Q8TCU6	6	63	2.478542	6.06E+12	2.45E+12	########	3.64E+13	1.54E+14
sp|Q8TCZ2	6	63	3.697225	3.39E+14	9.16E+13	########	2.03E+15	6E+15
sp|Q8TD22	6	63	27.45977	5.13E+15	1.87E+14	########	3.08E+16	1.18E+16
sp|Q8TDC3	6	63	4.246353	6.14E+13	1.45E+13	########	3.68E+14	9.1E+14
sp|Q8TDJ6	6	63	21.13503	7.83E+16	3.7E+15	########	4.7E+17	2.33E+17
sp|Q8TDQ7	6	63	34.67277	1.33E+15	3.84E+13	########	8E+15	2.42E+15
sp|Q8TDW0	6	63	5.400978	6.39E+14	1.18E+14	########	3.83E+15	7.45E+15
sp|Q8TEA8	6	63	29.61469	5.86E+14	1.98E+13	########	3.52E+15	1.25E+15
sp|Q8TEQ8	6	63	4.557043	7.64E+10	1.68E+10	########	4.59E+11	1.06E+12
sp|Q8TF30	6	63	3.81623	3.83E+14	1E+14	########	2.3E+15	6E+15
sp|Q8TF44	6	63	2.763536	7.74E+12	2.8E+12	########	4.64E+13	1.76E+14
sp|Q8TF61	6	63	4.809724	1.13E+14	2.35E+13	########	6.77E+14	1.48E+15
sp|Q8WU68	6	63	28.91514	1.16E+14	4.01E+12	########	6.96E+14	2.53E+14
sp|Q8WU79	6	63	6.23134	1.3E+14	2.09E+13	########	7.8E+14	1.31E+15
sp|Q8WUD1	6	63	1.238438	1.25E+13	1.01E+13	########	7.52E+13	6.38E+14
sp|Q8WUK0	6	63	10.38132	1.26E+13	1.21E+12	########	7.56E+13	7.65E+13
sp|Q8WUM4	6	63	1.42738	2.83E+15	1.98E+15	########	1.7E+16	1E+17

For Peer Review Only

sp|Q8WUW	6	63	46.96861	1.51E+15	3.22E+13	########	9.07E+15	2.03E+15
sp|Q8WUX	6	63	11.2806	3.51E+12	3.11E+11	########	2.1E+13	1.96E+13
sp|Q8WUY	6	63	3.482071	2.9E+13	8.33E+12	########	1.74E+14	5.25E+14
sp|Q8WUY	6	63	25.4746	3.55E+14	1.39E+13	########	2.13E+15	8.79E+14
sp|Q8WVC	6	63	2.510602	2.28E+12	9.1E+11	########	1.37E+13	5.73E+13
sp|Q8WVM	6	63	6.426334	7.09E+14	1.1E+14	########	4.25E+15	6.95E+15
sp|Q8WVV	6	63	9.775279	2.48E+14	2.54E+13	########	1.49E+15	1.6E+15
sp|Q8WW2	6	63	18.2392	2.19E+14	1.2E+13	########	1.32E+15	7.57E+14
sp|Q8WWI	6	63	1.720022	3.74E+10	2.17E+10	########	2.24E+11	1.37E+12
sp|Q8WWI	6	63	17.27883	1.49E+17	8.64E+15	########	8.96E+17	5.44E+17
sp|Q8WXC	6	63	1.341082	4.25E+12	3.17E+12	########	2.55E+13	2E+14
sp|Q8WXD	6	63	3.8854	1.02E+14	2.62E+13	########	6.1E+14	1.65E+15
sp|Q8WXD	6	63	11.86286	7.93E+14	6.69E+13	########	4.76E+15	4.21E+15
sp|Q8WXE	6	63	10.74273	6.29E+13	5.85E+12	########	3.77E+14	3.69E+14
sp|Q8WXF	6	63	22.66594	2.91E+15	1.28E+14	########	1.75E+16	8.09E+15
sp|Q8WXF	6	63	66.25399	5.75E+16	8.68E+14	########	########	5.47E+16
sp|Q8WXG	6	63	15.51329	4.09E+15	2.64E+14	########	2.46E+16	1.66E+16
sp|Q8WXH	6	63	136.4396	6.74E+15	4.94E+13	########	4.04E+16	3.11E+15
sp|Q8WXI2	6	63	4.23661	9.84E+12	2.32E+12	########	5.9E+13	1.46E+14
sp|Q8WXS	6	63	1.662813	5.16E+11	3.1E+11	########	3.09E+12	1.95E+13
sp|Q8WY22	6	63	2.195999	2.55E+13	1.16E+13	########	1.53E+14	7.33E+14
sp|Q8WZ42	6	63	1.350434	2.78E+14	2.06E+14	########	1.67E+15	1.3E+16
sp|Q8WZA	6	63	13.10903	1.09E+14	8.31E+12	########	6.54E+14	5.24E+14
sp|Q8WZA	6	63	38.49598	1.61E+14	4.19E+12	########	9.67E+14	2.64E+14
sp|Q8WZA	6	63	6.687243	3.2E+14	4.78E+13	########	1.92E+15	3.01E+15
sp|Q92499	6	63	15.28148	3.17E+16	2.08E+15	########	########	1.31E+17
sp|Q92504	6	63	4.555251	2.69E+13	5.91E+12	########	1.61E+14	3.72E+14
sp|Q92520	6	63	########	1.99E+12	3.55E+12	########	1.19E+13	2.24E+14
sp|Q92522	6	63	40.03973	8.91E+14	2.23E+13	########	5.35E+15	1E+15
sp|Q92529	6	63	2.019126	8.03E+12	3.98E+12	########	4.82E+13	2.51E+14
sp|Q92530	6	63	1.730213	2.2E+13	1.27E+13	########	1.32E+14	8E+14
sp|Q92538	6	63	########	2.77E+11	3.29E+11	########	1.66E+12	2.07E+13
sp|Q92542	6	63	21.60787	9.29E+13	4.3E+12	########	5.58E+14	2.71E+14
sp|Q92556	6	63	4.039187	3.74E+14	9.27E+13	########	2.25E+15	5.84E+15
sp|Q92558	6	63	21.73213	1.14E+16	5.25E+14	########	6.84E+16	3.31E+16
sp|Q92561	6	63	42.58633	1.93E+16	4.53E+14	########	1.16E+17	3E+16
sp|Q92572	6	63	2.135092	1.03E+13	4.82E+12	########	6.17E+13	3.04E+14
sp|Q92574	6	63	14.69785	7.79E+13	5.3E+12	########	4.68E+14	3.34E+14
sp|Q92581	6	63	3.938876	1.48E+13	3.76E+12	########	8.88E+13	2.37E+14
sp|Q92597	6	63	13.46257	4.52E+16	3.36E+15	########	2.71E+17	2.12E+17
sp|Q92598	6	63	37.89423	2.32E+17	6.12E+15	########	1.39E+18	########
sp|Q92599	6	63	16.32261	2.38E+17	1.46E+16	########	1E+18	9.19E+17
sp|Q92604	6	63	1.737285	9.84E+12	5.66E+12	########	5.9E+13	3.57E+14
sp|Q92614	6	63	76.26925	2.09E+16	2.74E+14	########	1.25E+17	1.73E+16
sp|Q92616	6	63	14.46088	1.93E+14	1.33E+13	########	1.16E+15	8.4E+14
sp|Q92621	6	63	45.55862	3.78E+13	8.3E+11	########	2.27E+14	5.23E+13
sp|Q92667	6	63	6.172862	1.07E+14	1.73E+13	########	6.39E+14	1.09E+15
sp|Q92673	6	63	9.881134	1.37E+14	1.39E+13	########	8.24E+14	8.76E+14
sp|Q92686	6	63	23.21266	4.24E+17	1.83E+16	########	2.54E+18	1.15E+18
sp|Q92688	6	63	49.94009	2.95E+14	5.9E+12	########	1.77E+15	3.72E+14
sp|Q92696	6	63	########	5.57E+13	6.35E+13	########	3.34E+14	4E+15
sp|Q92734	6	63	103.0653	1.19E+16	1.16E+14	########	7.15E+16	7.29E+15
sp|Q92736	6	63	20.77817	3.99E+15	1.92E+14	########	2.4E+16	1.21E+16
sp|Q92743	6	63	1.349144	1.91E+13	1.42E+13	########	1.15E+14	8.92E+14
sp|Q92747	6	63	4.114236	2.34E+15	5.69E+14	########	1.4E+16	3.58E+16
sp|Q92752	6	63	9.832588	1E+18	1.04E+17	########	6E+18	7E+18

For Peer Review Only

sp|Q92777|	6	63	12.49468	5.94E+17	4.76E+16	########	3.57E+18	########
sp|Q92783|	6	63	5.404771	2.53E+14	4.68E+13	########	1.52E+15	3E+15
sp|Q92796|	6	63	3.580343	1.97E+13	5.49E+12	########	1.18E+14	3.46E+14
sp|Q92820|	6	63	15.90054	2.91E+14	1.83E+13	########	1.75E+15	1E+15
sp|Q92823|	6	63	42.36464	4E+17	9.45E+15	########	2.4E+18	6E+17
sp|Q92841|	6	63	51.24293	1.44E+16	2.8E+14	########	8.62E+16	1.77E+16
sp|Q92845|	6	63	10.43728	3.35E+13	3.21E+12	########	2.01E+14	2.02E+14
sp|Q92854|	6	63	########	8.63E+12	9.49E+12	########	5.18E+13	5.98E+14
sp|Q92859|	6	63	19.69682	2.7E+14	1.37E+13	########	1.62E+15	8.64E+14
sp|Q92882|	6	63	19.06536	6.02E+14	3.16E+13	########	3.61E+15	1.99E+15
sp|Q92896|	6	63	7.463242	5.76E+14	7.72E+13	########	3.46E+15	4.86E+15
sp|Q92900|	6	63	12.36245	3.99E+14	3.23E+13	########	2.39E+15	2.03E+15
sp|Q92905|	6	63	10.54878	6.37E+14	6.04E+13	########	3.82E+15	3.8E+15
sp|Q92930|	6	63	3.336438	3.65E+14	1.09E+14	########	2.19E+15	6.89E+15
sp|Q92932|	6	63	6.154572	4.37E+13	7.11E+12	########	2.62E+14	4.48E+14
sp|Q92945|	6	63	18.81715	1.68E+16	8.92E+14	########	1.01E+17	5.62E+16
sp|Q92947|	6	63	13.31438	1E+14	7.52E+12	########	6.01E+14	4.74E+14
sp|Q92973|	6	63	2.14362	9.96E+13	4.65E+13	########	5.98E+14	2.93E+15
sp|Q92974|	6	63	41.78477	4.61E+15	1.1E+14	########	2.77E+16	6.96E+15
sp|Q92982|	6	63	20.13162	1.01E+13	5.01E+11	########	6.05E+13	3.15E+13
sp|Q92990|	6	63	1.264129	1.57E+13	1.25E+13	########	9.45E+13	7.85E+14
sp|Q93008|	6	63	19.48181	8.71E+14	4.47E+13	########	5.23E+15	2.82E+15
sp|Q93009|	6	63	3.259632	6.89E+14	2.11E+14	########	4.13E+15	1E+16
sp|Q93034|	6	63	34.62363	1.63E+15	4.71E+13	########	9.79E+15	3E+15
sp|Q93050|	6	63	15.27872	1.43E+18	9.37E+16	########	9E+18	6E+18
sp|Q93077|	6	63	7.940865	1.72E+17	2.17E+16	########	1E+18	1.37E+18
sp|Q93100|	6	63	1.829512	1.4E+12	7.67E+11	########	8.42E+12	4.83E+13
sp|Q969E2	6	63	3.691541	4.21E+13	1.14E+13	########	2.53E+14	7.19E+14
sp|Q969E4	6	63	24.54202	7.01E+13	2.85E+12	########	4.2E+14	1.8E+14
sp|Q969G6	6	63	7.535086	1.76E+13	2.34E+12	########	1.06E+14	1.47E+14
sp|Q969H8	6	63	2.24422	6.89E+14	3.07E+14	########	4.13E+15	1.93E+16
sp|Q969L2|	6	63	4.351379	1.16E+15	2.67E+14	########	6.96E+15	1.68E+16
sp|Q969P0	6	63	47.00089	7.77E+17	1.65E+16	########	5E+18	1.04E+18
sp|Q969Q0	6	63	40.5615	1.02E+15	2.51E+13	########	6.1E+15	1.58E+15
sp|Q969S9	6	63	5.242439	5.68E+12	1.08E+12	########	3.41E+13	6.83E+13
sp|Q969T9	6	63	7.485605	1.59E+14	2.13E+13	########	9.56E+14	1E+15
sp|Q969Z0	6	63	10.85209	4.94E+13	4.55E+12	########	2.96E+14	2.87E+14
sp|Q969Z3	6	63	19.37931	3.36E+14	1.73E+13	########	2.02E+15	1.09E+15
sp|Q96A00	6	63	4.553989	4.66E+15	1.02E+15	########	2.8E+16	6.45E+16
sp|Q96A26	6	63	41.5875	7.03E+15	1.69E+14	########	4.22E+16	1.06E+16
sp|Q96A33	6	63	27.80779	1.73E+14	6.21E+12	########	1.04E+15	3.91E+14
sp|Q96A65	6	63	15.74294	4.53E+14	2.88E+13	########	2.72E+15	1.81E+15
sp|Q96AB3	6	63	38.79596	2.55E+16	6.57E+14	########	1.53E+17	4.14E+16
sp|Q96AC1	6	63	22.80207	3.2E+15	1.4E+14	########	1.92E+16	9E+15
sp|Q96AE4	6	63	24.19227	1.86E+14	7.71E+12	########	1.12E+15	4.86E+14
sp|Q96AG3	6	63	24.29308	4.28E+14	1.76E+13	########	2.57E+15	1.11E+15
sp|Q96AG4	6	63	17.98671	1.13E+15	6.3E+13	########	6.79E+15	3.97E+15
sp|Q96AJ9	6	63	########	2.97E+12	4.25E+12	########	1.78E+13	2.68E+14
sp|Q96AM1	6	63	1.725001	2.37E+12	1.37E+12	########	1.42E+13	8.64E+13
sp|Q96AQ6	6	63	35.2946	1.4E+17	3.98E+15	########	8.43E+17	2.51E+17
sp|Q96AT9	6	63	18.29948	4.73E+14	2.59E+13	########	2.84E+15	1.63E+15
sp|Q96AX1	6	63	32.58533	1.75E+14	5.37E+12	########	1.05E+15	3.38E+14
sp|Q96BM9	6	63	46.65352	3.7E+16	7.92E+14	########	########	4.99E+16
sp|Q96BQ5	6	63	11.4411	1.1E+13	9.59E+11	########	6.58E+13	6.04E+13
sp|Q96C19	6	63	35.92645	1.45E+15	4.02E+13	########	8.67E+15	2.53E+15
sp|Q96CM8	6	63	5.065397	1.43E+13	2.83E+12	########	8.59E+13	1.78E+14

For Peer Review Only

sp|Q96CN7	6	63	62.30778	2.8E+15	4.5E+13	########	1.68E+16	2.84E+15
sp|Q96CS3	6	63	17.96349	2.14E+14	1.19E+13	########	1.29E+15	7.52E+14
sp|Q96CT7	6	63	23.43399	1.58E+15	6.73E+13	########	9.47E+15	4.24E+15
sp|Q96CV9	6	63	15.4301	3.41E+14	2.21E+13	########	2.05E+15	1.39E+15
sp|Q96CW	6	63	24.57318	4.21E+17	1.71E+16	########	2.53E+18	1.08E+18
sp|Q96CX2	6	63	8.277446	5.45E+14	6.58E+13	########	3.27E+15	4.14E+15
sp|Q96D09	6	63	5.724338	4.4E+11	7.68E+10	########	2.64E+12	4.84E+12
sp|Q96D71	6	63	1.134574	6.44E+12	5.67E+12	########	3.86E+13	3.57E+14
sp|Q96DA2	6	63	7.49429	1.09E+14	1.46E+13	########	6.56E+14	9.19E+14
sp|Q96DA6	6	63	9.428513	1.15E+13	1.22E+12	########	6.88E+13	7.66E+13
sp|Q96DB2	6	63	4.224785	3.13E+14	7.41E+13	########	1.88E+15	4.67E+15
sp|Q96DD7	6	63	32.67584	2.89E+14	8.86E+12	########	1.74E+15	5.58E+14
sp|Q96DE0	6	63	3.372772	8.44E+12	2.5E+12	########	5.06E+13	1.58E+14
sp|Q96DG6	6	63	37.91764	1.21E+15	3.19E+13	########	7.26E+15	2.01E+15
sp|Q96DZ1	6	63	2.016543	5E+12	2.48E+12	########	3E+13	1.56E+14
sp|Q96DZ9	6	63	2.64631	2.35E+14	8.87E+13	########	1.41E+15	5.59E+15
sp|Q96E17	6	63	8.46294	8.2E+14	9.69E+13	########	4.92E+15	6E+15
sp|Q96EE3	6	63	########	1.57E+13	2.57E+13	########	9.45E+13	1.62E+15
sp|Q96EK5	6	63	5.499592	2.18E+13	3.96E+12	########	1.31E+14	2.5E+14
sp|Q96EQ0	6	63	2.200076	1.33E+14	6.04E+13	########	7.98E+14	4E+15
sp|Q96EY1	6	63	56.20999	1.27E+15	2.25E+13	########	7.6E+15	1.42E+15
sp|Q96EY7	6	63	4.826623	2.25E+12	4.66E+11	########	1.35E+13	2.93E+13
sp|Q96F07	6	63	93.60606	1.1E+17	1.18E+15	########	7E+17	7.42E+16
sp|Q96F24	6	63	3.132218	8.94E+11	2.86E+11	########	5.37E+12	1.8E+13
sp|Q96F85	6	63	16.63152	5.58E+16	3.35E+15	########	3.35E+17	2.11E+17
sp|Q96FC7	6	63	########	2.17E+14	8.17E+14	########	1.3E+15	5.15E+16
sp|Q96FE5	6	63	3.823827	8.11E+13	2.12E+13	########	4.87E+14	1.34E+15
sp|Q96FJ2	6	63	24.33912	2.08E+16	8.54E+14	########	1.25E+17	5E+16
sp|Q96FN4	6	63	########	2.28E+12	6.22E+12	########	1.37E+13	3.92E+14
sp|Q96FQ6	6	63	9.829273	2.35E+14	2.39E+13	########	1.41E+15	1.51E+15
sp|Q96FW1	6	63	7.898477	1.98E+16	2.51E+15	########	1.19E+17	2E+17
sp|Q96FZ7	6	63	1.09872	2.93E+12	2.67E+12	########	1.76E+13	1.68E+14
sp|Q96G03	6	63	98.2961	3.22E+15	3.27E+13	########	1.93E+16	2.06E+15
sp|Q96GA7	6	63	2.756703	3.06E+14	1.11E+14	########	1.83E+15	6.99E+15
sp|Q96GD0	6	63	148.4621	4.56E+16	3.07E+14	########	2.74E+17	1.94E+16
sp|Q96GG9	6	63	6.003935	9.63E+13	1.6E+13	########	5.78E+14	1.01E+15
sp|Q96GK7	6	63	27.59611	2.52E+15	9.12E+13	########	1.51E+16	5.74E+15
sp|Q96GQ5	6	63	1.76567	2.55E+14	1.44E+14	########	1.53E+15	9.09E+15
sp|Q96GR2	6	63	5.192183	7.13E+12	1.37E+12	########	4.28E+13	8.66E+13
sp|Q96GW	6	63	22.67238	4.04E+17	1.78E+16	########	2.42E+18	1.12E+18
sp|Q96HE7	6	63	10.65942	1.91E+14	1.79E+13	########	1.15E+15	1.13E+15
sp|Q96HN2	6	63	40.66874	3.51E+15	8.62E+13	########	2.1E+16	5.43E+15
sp|Q96HR9	6	63	6.587177	8.5E+12	1.29E+12	########	5.1E+13	8.13E+13
sp|Q96HS1	6	63	34.32377	4.27E+14	1.24E+13	########	2.56E+15	7.83E+14
sp|Q96HU8	6	63	12.72461	4.83E+14	3.79E+13	########	2.9E+15	2E+15
sp|Q96HY6	6	63	9.184873	1.18E+14	1.28E+13	########	7.07E+14	8.08E+14
sp|Q96I15|	6	63	10.2126	1.76E+14	1.72E+13	########	1.05E+15	1.08E+15
sp|Q96I23|	6	63	9.756083	3.54E+13	3.63E+12	########	2.12E+14	2.29E+14
sp|Q96I99|	6	63	9.095148	5.51E+14	6.06E+13	########	3.31E+15	4E+15
sp|Q96ID5|	6	63	6.220602	5.4E+14	8.68E+13	########	3.24E+15	5E+15
sp|Q96IU4|	6	63	6.101193	4.49E+14	7.37E+13	########	2.7E+15	4.64E+15
sp|Q96IX5|	6	63	39.35341	6.72E+15	1.71E+14	########	4.03E+16	1.08E+16
sp|Q96JE9	6	63	3.517846	1.38E+16	3.91E+15	########	8.26E+16	2.46E+17
sp|Q96JG6	6	63	10.28856	7.98E+13	7.76E+12	########	4.79E+14	4.89E+14
sp|Q96JH7	6	63	5.037559	3.97E+14	7.89E+13	########	2.38E+15	4.97E+15
sp|Q96JJ3|	6	63	1.279089	4E+13	3.12E+13	########	2.4E+14	1.97E+15

For Peer Review

sp|Q96JJ7|	6	63	2.643086	6.03E+13	2.28E+13	########	3.62E+14	1.44E+15
sp|Q96JQ2	6	63	1.703918	9.7E+11	5.69E+11	########	5.82E+12	3.59E+13
sp|Q96K17	6	63	29.77734	5.94E+15	1.99E+14	########	3.56E+16	1.26E+16
sp|Q96KG9	6	63	11.69693	6.67E+13	5.7E+12	########	4E+14	3.59E+14
sp|Q96KN2	6	63	26.37048	7E+14	2.65E+13	########	4.2E+15	1.67E+15
sp|Q96KP1	6	63	20.44907	1.65E+14	8.06E+12	########	9.89E+14	5.08E+14
sp|Q96KP4	6	63	29.56855	6.53E+17	2.21E+16	########	4E+18	########
sp|Q96L92|	6	63	5.676765	8.42E+13	1.48E+13	########	5.05E+14	9.34E+14
sp|Q96LT7	6	63	1.040785	1.63E+10	1.57E+10	########	9.8E+10	9.88E+11
sp|Q96MM(	6	63	5.607749	9.46E+14	1.69E+14	########	5.68E+15	1.06E+16
sp|Q96MZ0	6	63	76.71235	6.46E+15	8.43E+13	########	3.88E+16	5.31E+15
sp|Q96N66	6	63	5.87857	1.6E+14	2.73E+13	########	9.62E+14	2E+15
sp|Q96NN9	6	63	3.860929	8.73E+12	2.26E+12	########	5.24E+13	1.42E+14
sp|Q96NW7	6	63	3.215206	4.78E+12	1.49E+12	########	2.87E+13	9.37E+13
sp|Q96P47	6	63	14.97308	3.55E+15	2.37E+14	########	2.13E+16	1.49E+16
sp|Q96P70	6	63	27.489	5.99E+14	2.18E+13	########	3.59E+15	1.37E+15
sp|Q96PE3	6	63	16.92959	3.41E+14	2.01E+13	########	2.05E+15	1.27E+15
sp|Q96PE5	6	63	5.204756	1.16E+15	2.24E+14	########	6.99E+15	1.41E+16
sp|Q96PE7	6	63	1.280293	2.23E+12	1.74E+12	########	1.34E+13	1.1E+14
sp|Q96PU5	6	63	2.634244	1.67E+11	6.35E+10	########	1E+12	4E+12
sp|Q96PU8	6	63	8.374634	8.16E+14	9.74E+13	########	4.89E+15	6.14E+15
sp|Q96PV0	6	63	34.48148	5E+16	1.52E+15	########	3E+17	9.6E+16
sp|Q96PY5	6	63	9.428963	1.22E+16	1.29E+15	########	7.32E+16	8.15E+16
sp|Q96Q05	6	63	5.791011	2.73E+13	4.72E+12	########	1.64E+14	2.97E+14
sp|Q96QE2	6	63	4.143383	1.39E+13	3.35E+12	########	8.32E+13	2.11E+14
sp|Q96QK1	6	63	4.504373	2.35E+16	5.23E+15	########	########	3.29E+17
sp|Q96QR8	6	63	1.93555	4.74E+13	2.45E+13	########	2.84E+14	1.54E+15
sp|Q96RD7	6	63	5.441053	3.54E+13	6.5E+12	########	2.12E+14	4.1E+14
sp|Q96RF0	6	63	1.270714	8.39E+12	6.6E+12	########	5.03E+13	4.16E+14
sp|Q96RL7	6	63	10.52762	2.96E+13	2.81E+12	########	1.78E+14	1.77E+14
sp|Q96RP9	6	63	21.82377	1.5E+14	6.85E+12	########	8.97E+14	4.32E+14
sp|Q96RQ3	6	63	15.56635	7.26E+14	4.66E+13	########	4.35E+15	2.94E+15
sp|Q96RR4	6	63	15.57226	9.95E+14	6.39E+13	########	5.97E+15	4.02E+15
sp|Q96RS6	6	63	3.782833	3.9E+12	1.03E+12	########	2.34E+13	6.49E+13
sp|Q96RT1	6	63	2.282143	1.84E+13	8.05E+12	########	1.1E+14	5.07E+14
sp|Q96S52	6	63	6.669492	1.85E+13	2.77E+12	########	1.11E+14	1.74E+14
sp|Q96S66	6	63	1.115204	2.05E+11	1.83E+11	########	1.23E+12	1.16E+13
sp|Q96S97	6	63	2.485681	3.86E+14	1.55E+14	########	2.31E+15	1E+16
sp|Q96SB3	6	63	17.06492	2.66E+14	1.56E+13	########	1.6E+15	9.84E+14
sp|Q96SW2	6	63	4.301523	7.9E+12	1.84E+12	########	4.74E+13	1.16E+14
sp|Q96SZ5	6	63	20.5853	2.44E+14	1.19E+13	########	1.46E+15	7.47E+14
sp|Q96T76	6	63	33.96908	6.67E+14	1.96E+13	########	4E+15	1.24E+15
sp|Q96TA1	6	63	########	3.75E+12	4.02E+12	########	2.25E+13	2.53E+14
sp|Q96TC7	6	63	15.81506	1.37E+15	8.65E+13	########	8.21E+15	5.45E+15
sp|Q99250|	6	63	43.15148	5.87E+15	1.36E+14	########	3.52E+16	9E+15
sp|Q99259|	6	63	8.738407	3.8E+14	4.35E+13	########	2.28E+15	2.74E+15
sp|Q99426|	6	63	3.34934	1.86E+15	5.56E+14	########	1.12E+16	3.5E+16
sp|Q99436|	6	63	54.72841	5.64E+15	1.03E+14	########	3.38E+16	6.49E+15
sp|Q99447|	6	63	12.10114	6.89E+14	5.7E+13	########	4.14E+15	4E+15
sp|Q99460|	6	63	28.88214	3.78E+15	1.31E+14	########	2.27E+16	8.24E+15
sp|Q99471|	6	63	45.1383	9.64E+14	2.13E+13	########	5.78E+15	1.34E+15
sp|Q99490|	6	63	5.127299	1.03E+13	2.01E+12	########	6.17E+13	1.26E+14
sp|Q99497|	6	63	173.4291	6E+18	3.46E+16	########	4E+19	2.18E+18
sp|Q99523|	6	63	6.547015	6.09E+14	9.3E+13	########	3.65E+15	5.86E+15
sp|Q99536|	6	63	14.6685	2.04E+16	1.39E+15	########	1.22E+17	9E+16
sp|Q99569|	6	63	25.42523	9.87E+13	3.88E+12	########	5.92E+14	2.44E+14

For Peer Review Only

sp|Q99572|	6	63	9.420487	1.08E+15	1.14E+14	########	6.45E+15	7E+15
sp|Q99574|	6	63	########	6.62E+11	9.49E+11	########	3.97E+12	5.98E+13
sp|Q99584|	6	63	137.3542	6.51E+16	4.74E+14	########	3.91E+17	2.99E+16
sp|Q99598|	6	63	76.47958	2.91E+15	3.8E+13	########	1.75E+16	2.4E+15
sp|Q99613|	6	63	12.28584	1.57E+15	1.27E+14	########	9.4E+15	8.03E+15
sp|Q99614|	6	63	9.657263	1.28E+14	1.33E+13	########	7.7E+14	8.37E+14
sp|Q99615|	6	63	12.10532	8.19E+13	6.77E+12	########	4.92E+14	4.26E+14
sp|Q99622|	6	63	1.744622	8.75E+12	5.02E+12	########	5.25E+13	3.16E+14
sp|Q99623|	6	63	139.9061	4.16E+17	2.98E+15	########	2.5E+18	1.88E+17
sp|Q99627|	6	63	2.545616	2.51E+14	9.87E+13	########	1.51E+15	6.22E+15
sp|Q99643|	6	63	9.728567	1.93E+14	1.98E+13	########	1.16E+15	1E+15
sp|Q99653|	6	63	5.718346	2.04E+14	3.56E+13	########	1.22E+15	2.24E+15
sp|Q99683|	6	63	1.287106	4.46E+11	3.47E+11	########	2.68E+12	2.18E+13
sp|Q99685|	6	63	7.321304	1.11E+15	1.51E+14	########	6.65E+15	9.53E+15
sp|Q99714|	6	63	45.37265	1.85E+16	4.08E+14	########	1.11E+17	3E+16
sp|Q99719|	6	63	9.585813	4.56E+17	4.76E+16	########	3E+18	3E+18
sp|Q99726|	6	63	6.794267	2.83E+15	4.17E+14	########	1.7E+16	2.63E+16
sp|Q99729|	6	63	8.897012	4.99E+14	5.61E+13	########	3E+15	4E+15
sp|Q99733|	6	63	########	1.04E+14	2.32E+14	########	6.24E+14	1.46E+16
sp|Q99747|	6	63	15.53785	2.63E+16	1.7E+15	########	########	1.07E+17
sp|Q99766|	6	63	14.06795	7.05E+13	5.01E+12	########	4.23E+14	3.16E+14
sp|Q99767|	6	63	1.422347	1.5E+12	1.06E+12	########	9.01E+12	6.65E+13
sp|Q99784|	6	63	5.391357	4.56E+14	8.46E+13	########	2.74E+15	5.33E+15
sp|Q99798|	6	63	39.88899	7E+18	1.71E+17	########	4E+19	1E+19
sp|Q99805|	6	63	4.055342	8.06E+13	1.99E+13	########	4.83E+14	1E+15
sp|Q99807|	6	63	4.497646	2.65E+13	5.88E+12	########	1.59E+14	3.71E+14
sp|Q99816|	6	63	1.849414	1.76E+13	9.49E+12	########	1.05E+14	5.98E+14
sp|Q99829|	6	63	########	7.74E+12	1.28E+13	########	4.65E+13	8.06E+14
sp|Q99832|	6	63	36.75536	5.3E+16	1.44E+15	########	3.18E+17	9.09E+16
sp|Q99873|	6	63	8.371034	3.38E+13	4.04E+12	########	2.03E+14	2.54E+14
sp|Q99879|	6	63	137.8779	8.96E+16	6.5E+14	########	5.37E+17	4.09E+16
sp|Q99884|	6	63	1.304571	2.4E+13	1.84E+13	########	1.44E+14	1.16E+15
sp|Q99943|	6	63	2.08162	1.64E+13	7.86E+12	########	9.82E+13	4.95E+14
sp|Q99961|	6	63	5.007087	4.02E+14	8.03E+13	########	2.41E+15	5.06E+15
sp|Q99962|	6	63	6.043817	1.11E+17	1.83E+16	########	6.65E+17	1.16E+18
sp|Q99963|	6	63	1.153125	2.3E+14	2E+14	########	1E+15	1.26E+16
sp|Q9BPU6	6	63	64.69411	########	6.94E+15	########	2.69E+18	4.37E+17
sp|Q9BPW	6	63	37.30078	1.51E+17	4.05E+15	########	9.07E+17	2.55E+17
sp|Q9BPX5	6	63	3.086216	7.99E+14	2.59E+14	########	4.8E+15	1.63E+16
sp|Q9BQ69	6	63	30.26223	2.94E+15	9.72E+13	########	1.76E+16	6.12E+15
sp|Q9BQ7(	6	63	3.608934	2.43E+13	6.74E+12	########	1.46E+14	4.24E+14
sp|Q9BQE5	6	63	7.297409	1.79E+14	2.46E+13	########	1.08E+15	1.55E+15
sp|Q9BQG	6	63	########	2.2E+12	3.03E+12	########	1.32E+13	1.91E+14
sp|Q9BQI5	6	63	2.596754	5.55E+14	2.14E+14	########	3.33E+15	1.35E+16
sp|Q9BQI7	6	63	4.300408	4.79E+14	1.11E+14	########	2.87E+15	7.02E+15
sp|Q9BQI9	6	63	4.633909	1.06E+13	2.29E+12	########	6.35E+13	1.44E+14
sp|Q9BR01	6	63	39.23142	1.23E+15	3.13E+13	########	7.37E+15	1.97E+15
sp|Q9BR76	6	63	########	9.39E+12	2.74E+13	########	5.63E+13	1.72E+15
sp|Q9BRA2	6	63	8.328346	3.65E+13	4.38E+12	########	2.19E+14	2.76E+14
sp|Q9BRF8	6	63	23.99724	5.44E+15	2.27E+14	########	3.26E+16	1.43E+16
sp|Q9BRK(	6	63	4.017833	1.66E+14	4.12E+13	########	9.94E+14	2.6E+15
sp|Q9BRK4	6	63	########	2.36E+14	2.92E+14	########	1.42E+15	2E+16
sp|Q9BRQ(	6	63	16.23305	5.05E+14	3.11E+13	########	3.03E+15	1.96E+15
sp|Q9BRX8	6	63	43.6267	2.51E+16	5.76E+14	########	1.51E+17	3.63E+16
sp|Q9BS26	6	63	16.63732	5.16E+14	3.1E+13	########	3.09E+15	1.95E+15
sp|Q9BSA4	6	63	4.002008	2.26E+14	5.66E+13	########	1.36E+15	3.56E+15

For Review Only

sp|Q9BSF(	6	63	########	2.7E+12	3.01E+12	########	1.62E+13	1.89E+14
sp|Q9BSH4	6	63	35.90693	1.08E+15	3.01E+13	########	6.48E+15	2E+15
sp|Q9BSH5	6	63	7.749227	1.11E+14	1.44E+13	########	6.67E+14	9.04E+14
sp|Q9BSJ8	6	63	72.67513	1.33E+16	1.83E+14	########	8E+16	1.16E+16
sp|Q9BST9	6	63	1.411317	3.99E+12	2.83E+12	########	2.39E+13	1.78E+14
sp|Q9BSU1	6	63	16.78401	1.91E+14	1.14E+13	########	1.14E+15	7.16E+14
sp|Q9BT78	6	63	4.737352	5.01E+14	1.06E+14	########	3.01E+15	6.66E+15
sp|Q9BTE1	6	63	2.877745	1.43E+13	4.96E+12	########	8.57E+13	3.13E+14
sp|Q9BTE6	6	63	6.298189	2.55E+13	4.05E+12	########	1.53E+14	2.55E+14
sp|Q9BTV4	6	63	5.655757	1.94E+14	3.43E+13	########	1.16E+15	2.16E+15
sp|Q9BTV5	6	63	24.87423	2.88E+15	1.16E+14	########	1.73E+16	7.3E+15
sp|Q9BTW9	6	63	27.15766	1.35E+15	4.96E+13	########	8.09E+15	3.13E+15
sp|Q9BU02	6	63	12.77413	1.67E+14	1.31E+13	########	1E+15	8.23E+14
sp|Q9BUF5	6	63	1.027153	2.77E+15	2.7E+15	########	1.66E+16	########
sp|Q9BUR5	6	63	49.95477	1.13E+16	2.27E+14	########	6.79E+16	1.43E+16
sp|Q9BUT1	6	63	102.0856	7.65E+15	7.49E+13	########	4.59E+16	4.72E+15
sp|Q9BV20	6	63	2.766249	4.09E+13	1.48E+13	########	2.45E+14	9.31E+14
sp|Q9BV23	6	63	1.609384	4.49E+12	2.79E+12	########	2.69E+13	1.76E+14
sp|Q9BV79	6	63	10.20961	1.9E+14	1.86E+13	########	1.14E+15	1.17E+15
sp|Q9BVA0	6	63	########	1.74E+13	4.96E+13	########	1.04E+14	3.13E+15
sp|Q9BVA1	6	63	9.05123	2.69E+16	2.97E+15	########	1.61E+17	2E+17
sp|Q9BVK6	6	63	7.172988	3.06E+13	4.27E+12	########	1.84E+14	2.69E+14
sp|Q9BW30	6	63	18.08253	7.74E+16	4.28E+15	########	4.65E+17	2.7E+17
sp|Q9BW62	6	63	1.141659	4.21E+11	3.69E+11	########	2.52E+12	2.32E+13
sp|Q9BW72	6	63	64.98078	2.3E+15	3.54E+13	########	1.38E+16	2.23E+15
sp|Q9BWD	6	63	82.08447	2.33E+17	2.83E+15	########	1.4E+18	1.78E+17
sp|Q9BWM	6	63	66.99414	7.92E+16	1.18E+15	########	4.75E+17	7.45E+16
sp|Q9BWQ	6	63	4.580651	9.69E+13	2.12E+13	########	5.82E+14	1.33E+15
sp|Q9BWS9	6	63	1.481963	1.78E+13	1.2E+13	########	1.07E+14	7.58E+14
sp|Q9BX66	6	63	10.29388	6.34E+14	6.16E+13	########	3.8E+15	3.88E+15
sp|Q9BX67	6	63	5.267344	6.73E+14	1.28E+14	########	4.04E+15	8E+15
sp|Q9BX68	6	63	34.94565	3.49E+15	9.97E+13	########	2.09E+16	6.28E+15
sp|Q9BXF6	6	63	1.596519	4.52E+12	2.83E+12	########	2.71E+13	1.79E+14
sp|Q9BXJ9	6	63	4.550218	1.56E+14	3.43E+13	########	9.36E+14	2.16E+15
sp|Q9BXK5	6	63	17.59256	4.9E+15	2.79E+14	########	2.94E+16	1.76E+16
sp|Q9BXS0	6	63	1.476136	1.12E+14	7.6E+13	########	6.73E+14	4.79E+15
sp|Q9BXS5	6	63	1.620705	4.14E+13	2.56E+13	########	2.49E+14	2E+15
sp|Q9BXW6	6	63	2.086757	2.81E+14	1.35E+14	########	1.68E+15	8.48E+15
sp|Q9BXW	6	63	6.080561	1.38E+13	2.27E+12	########	8.28E+13	1.43E+14
sp|Q9BY11	6	63	14.31407	1.1E+18	7.7E+16	########	7E+18	5E+18
sp|Q9BY32	6	63	6.52061	3.37E+14	5.16E+13	########	2.02E+15	3E+15
sp|Q9BY67	6	63	2.281063	1.93E+15	8.46E+14	########	1.16E+16	5.33E+16
sp|Q9BYB0	6	63	26.48151	1.39E+15	5.25E+13	########	8.34E+15	3.31E+15
sp|Q9BYD6	6	63	13.28171	7.08E+13	5.33E+12	########	4.25E+14	3.36E+14
sp|Q9BZ29	6	63	6.196883	2.8E+13	4.52E+12	########	1.68E+14	2.85E+14
sp|Q9BZC7	6	63	12.03232	1.28E+14	1.07E+13	########	7.7E+14	6.72E+14
sp|Q9BZE9	6	63	2.579403	1.13E+12	4.4E+11	########	6.81E+12	2.77E+13
sp|Q9BZF1	6	63	3.441595	3.71E+12	1.08E+12	########	2.23E+13	6.79E+13
sp|Q9BZF3	6	63	7.462024	9.27E+12	1.24E+12	########	5.56E+13	7.83E+13
sp|Q9BZV1	6	63	22.83164	7.46E+15	3.27E+14	########	4E+16	2.06E+16
sp|Q9BZZ5	6	63	4.841917	2.14E+13	4.42E+12	########	1.28E+14	2.79E+14
sp|Q9C005	6	63	1.96012	2.75E+13	1.4E+13	########	1.65E+14	8.83E+14
sp|Q9C026	6	63	1.175267	6.66E+11	5.67E+11	########	4E+12	3.57E+13
sp|Q9C040	6	63	1.046681	2.15E+15	2.06E+15	########	1.29E+16	1.3E+17
sp|Q9C0B1	6	63	39.12935	3.28E+14	8.38E+12	########	1.97E+15	5.28E+14
sp|Q9C0C9	6	63	10.89862	2.97E+15	2.73E+14	########	1.78E+16	1.72E+16

For Peer Review Only

sp|Q9C0D9	6	63	3.783628	4.4E+12	1.16E+12	########	2.64E+13	7.33E+13
sp|Q9C0E8	6	63	5.586379	1.12E+13	2E+12	########	6.71E+13	1.26E+14
sp|Q9C0H2	6	63	16.49257	8.1E+13	4.91E+12	########	4.86E+14	3.09E+14
sp|Q9C0H5	6	63	3.123743	1.63E+14	5.22E+13	########	9.78E+14	3.29E+15
sp|Q9C0H9	6	63	51.61738	7.83E+15	1.52E+14	########	4.7E+16	9.56E+15
sp|Q9GZM7	6	63	14.14093	1.95E+16	1.38E+15	########	1.17E+17	8.68E+16
sp|Q9GZM8	6	63	1.946489	1.44E+12	7.41E+11	########	8.65E+12	4.67E+13
sp|Q9GZN7	6	63	7.180202	4.68E+14	6.52E+13	########	2.81E+15	4.11E+15
sp|Q9GZP4	6	63	274.2596	1.54E+16	5.61E+13	########	9.24E+16	3.54E+15
sp|Q9GZQ8	6	63	18.33344	2.75E+16	1.5E+15	########	1.65E+17	9.44E+16
sp|Q9GZS3	6	63	19.80986	3.19E+14	1.61E+13	########	1.92E+15	1.02E+15
sp|Q9GZT3	6	63	17.5089	3.65E+14	2.08E+13	########	2.19E+15	1.31E+15
sp|Q9GZT4	6	63	6.819821	2.03E+14	2.98E+13	########	1.22E+15	1.88E+15
sp|Q9GZT6	6	63	2.674267	1.03E+14	3.87E+13	########	6.2E+14	2.44E+15
sp|Q9GZT8	6	63	36.76363	9.73E+15	2.65E+14	########	5.84E+16	1.67E+16
sp|Q9GZV7	6	63	8.804854	3E+18	2.99E+17	########	2E+19	2E+19
sp|Q9GZY8	6	63	22.13336	4.69E+15	2.12E+14	########	2.81E+16	1.33E+16
sp|Q9GZZ9	6	63	20.15864	8.84E+14	4.38E+13	########	5.3E+15	2.76E+15
sp|Q9H008	6	63	109.8002	7.78E+16	7.08E+14	########	4.67E+17	4.46E+16
sp|Q9H019	6	63	19.1879	1.42E+14	7.42E+12	########	8.54E+14	4.67E+14
sp|Q9H061	6	63	65.64072	7.09E+14	1.08E+13	########	4.25E+15	6.81E+14
sp|Q9H074	6	63	87.23139	7.96E+14	9.12E+12	########	4.78E+15	5.75E+14
sp|Q9H078	6	63	6.632436	4.01E+13	6.05E+12	########	2.41E+14	3.81E+14
sp|Q9H0A8	6	63	7.461011	3.57E+13	4.78E+12	########	2.14E+14	3.01E+14
sp|Q9H0B6	6	63	6.092071	1.7E+13	2.8E+12	########	1.02E+14	1.76E+14
sp|Q9H0E2	6	63	12.88809	2.91E+15	2.26E+14	########	1.75E+16	1.42E+16
sp|Q9H0Q0	6	63	13.17652	1.52E+15	1.15E+14	########	9.1E+15	7.25E+15
sp|Q9H0Q3	6	63	4.574918	5.74E+15	1.26E+15	########	3.45E+16	7.91E+16
sp|Q9H0R4	6	63	68.93396	1.39E+17	2.01E+15	########	8.32E+17	1.27E+17
sp|Q9H0R8	6	63	3.386975	5.15E+13	1.52E+13	########	3.09E+14	9.58E+14
sp|Q9H0U4	6	63	10.80718	1.88E+15	1.74E+14	########	1.13E+16	1.1E+16
sp|Q9H0W9	6	63	140.3148	3.44E+16	2.45E+14	########	########	1.54E+16
sp|Q9H115	6	63	15.91391	3.57E+17	2.24E+16	########	2.14E+18	1.41E+18
sp|Q9H1E5	6	63	9.919958	2.8E+14	2.82E+13	########	1.68E+15	2E+15
sp|Q9H1K0	6	63	2.582592	8.11E+11	3.14E+11	########	4.87E+12	1.98E+13
sp|Q9H1K1	6	63	6.068888	1.33E+14	2.19E+13	########	7.99E+14	1.38E+15
sp|Q9H1K4	6	63	7.248945	2.18E+14	3.01E+13	########	1.31E+15	1.89E+15
sp|Q9H1P3	6	63	4.581215	3.54E+13	7.73E+12	########	2.12E+14	4.87E+14
sp|Q9H1V8	6	63	7.00451	9.38E+14	1.34E+14	########	5.63E+15	8.44E+15
sp|Q9H1Z4	6	63	8.52539	3.4E+14	3.99E+13	########	2.04E+15	2.52E+15
sp|Q9H244	6	63	2.913126	7.65E+12	2.62E+12	########	4.59E+13	1.65E+14
sp|Q9H254	6	63	50.99811	9.84E+14	1.93E+13	########	5.9E+15	1.22E+15
sp|Q9H269	6	63	21.38548	3.92E+14	1.83E+13	########	2.35E+15	1E+15
sp|Q9H270	6	63	15.94683	2.26E+14	1.42E+13	########	1.36E+15	8.92E+14
sp|Q9H299	6	63	42.82525	6.25E+15	1.46E+14	########	3.75E+16	9E+15
sp|Q9H2C0	6	63	########	8.74E+11	1.07E+12	########	5.24E+12	6.76E+13
sp|Q9H2G2	6	63	10.86154	1.26E+14	1.16E+13	########	7.58E+14	7.32E+14
sp|Q9H2H9	6	63	4.545866	1.36E+13	2.98E+12	########	8.13E+13	1.88E+14
sp|Q9H2M9	6	63	19.60938	2.96E+14	1.51E+13	########	1.77E+15	9.5E+14
sp|Q9H2U2	6	63	60.32685	1.69E+15	2.79E+13	########	1.01E+16	1.76E+15
sp|Q9H2W0	6	63	1.677901	1.33E+12	7.9E+11	########	7.96E+12	4.98E+13
sp|Q9H2X9	6	63	22.03804	1.52E+17	6.89E+15	########	9.11E+17	4.34E+17
sp|Q9H305	6	63	3.313402	1.22E+13	3.69E+12	########	7.34E+13	2.33E+14
sp|Q9H313	6	63	82.42525	1.72E+16	2.09E+14	########	########	1.32E+16
sp|Q9H330	6	63	2.552246	2.28E+12	8.95E+11	########	1.37E+13	5.64E+13
sp|Q9H3H3	6	63	14.81804	1.81E+14	1.22E+13	########	1.09E+15	7.69E+14

For Peer Review Only

sp|Q9H3K6	6	63	4.999905	5.08E+13	1.02E+13	########	3.05E+14	6.4E+14
sp|Q9H3N1	6	63	7.934975	8.26E+13	1.04E+13	########	4.96E+14	6.56E+14
sp|Q9H3Q1	6	63	5.549603	1.67E+14	3.01E+13	########	1E+15	2E+15
sp|Q9H3S7	6	63	22.49941	5.79E+14	2.57E+13	########	3.48E+15	1.62E+15
sp|Q9H3Z4	6	63	17.59271	5.12E+16	2.91E+15	########	3.07E+17	1.83E+17
sp|Q9H425	6	63	2.418906	2.47E+13	1.02E+13	########	1.48E+14	6.43E+14
sp|Q9H444	6	63	1.54519	9.37E+13	6.06E+13	########	5.62E+14	3.82E+15
sp|Q9H479	6	63	4.727017	3.36E+14	7.11E+13	########	2.02E+15	4E+15
sp|Q9H490	6	63	23.13687	2.9E+13	1.25E+12	########	1.74E+14	7.9E+13
sp|Q9H492	6	63	6.391131	1.37E+16	2.15E+15	########	8.23E+16	1.35E+17
sp|Q9H4A4	6	63	12.56651	4.92E+14	3.91E+13	########	2.95E+15	2.46E+15
sp|Q9H4A6	6	63	3.67644	3.58E+12	9.73E+11	########	2.15E+13	6.13E+13
sp|Q9H4G0	6	63	58.06321	########	3.86E+15	########	1.35E+18	2.43E+17
sp|Q9H4G4	6	63	6.152052	2.19E+15	3.55E+14	########	1.31E+16	2.24E+16
sp|Q9H4M3	6	63	5.195718	3.94E+13	7.58E+12	########	2.36E+14	4.78E+14
sp|Q9H4M9	6	63	9.801786	6.68E+14	6.82E+13	########	4.01E+15	4.29E+15
sp|Q9H598	6	63	6.257699	2.77E+14	4.43E+13	########	1.66E+15	2.79E+15
sp|Q9H6K4	6	63	8.104664	2.83E+13	3.49E+12	########	1.7E+14	2.2E+14
sp|Q9H6K5	6	63	1.66804	1.82E+12	1.09E+12	########	1.09E+13	6.89E+13
sp|Q9H6L5	6	63	4.264191	4.38E+11	1.03E+11	########	2.63E+12	6.48E+12
sp|Q9H6R3	6	63	9.3206	7.42E+13	7.96E+12	########	4.45E+14	5.02E+14
sp|Q9H6U6	6	63	11.93716	3.57E+13	2.99E+12	########	2.14E+14	1.88E+14
sp|Q9H7D0	6	63	2.069643	7.58E+13	3.66E+13	########	4.55E+14	2.31E+15
sp|Q9H7Z7	6	63	23.14983	5.18E+15	2.24E+14	########	3.11E+16	1.41E+16
sp|Q9H845	6	63	26.85695	2.22E+15	8.28E+13	########	1.33E+16	5.21E+15
sp|Q9H8H3	6	63	52.05213	4.64E+16	8.91E+14	########	2.78E+17	5.61E+16
sp|Q9H902	6	63	1.175922	6.2E+10	5.28E+10	########	3.72E+11	3.32E+12
sp|Q9H936	6	63	70.3665	1.02E+17	1.45E+15	########	6.11E+17	9.11E+16
sp|Q9H993	6	63	5.826603	5.19E+14	8.92E+13	########	3.12E+15	5.62E+15
sp|Q9H9A6	6	63	6.796471	6.31E+13	9.29E+12	########	3.79E+14	5.85E+14
sp|Q9H9B4	6	63	71.48258	1.11E+17	1.55E+15	########	6.66E+17	9.78E+16
sp|Q9H9C1	6	63	3.677384	2.39E+12	6.51E+11	########	1.44E+13	4.1E+13
sp|Q9H9H5	6	63	1.588508	3.55E+14	2.23E+14	########	2.13E+15	1.41E+16
sp|Q9H9J2	6	63	2.517657	4.19E+12	1.67E+12	########	2.52E+13	1.05E+14
sp|Q9H9Q2	6	63	2.99668	1.04E+13	3.46E+12	########	6.22E+13	2.18E+14
sp|Q9H9S3	6	63	11.4198	9.1E+13	7.97E+12	########	5.46E+14	5.02E+14
sp|Q9H9S4	6	63	34.82266	4.11E+14	1.18E+13	########	2.46E+15	7.43E+14
sp|Q9HA64	6	63	70.32749	8.87E+15	1.26E+14	########	5.32E+16	7.94E+15
sp|Q9HA65	6	63	2.99669	8.06E+13	2.69E+13	########	4.84E+14	1.7E+15
sp|Q9HA77	6	63	6.469082	4.6E+13	7.12E+12	########	2.76E+14	4.48E+14
sp|Q9HAR2	6	63	2.15293	1.54E+12	7.17E+11	########	9.26E+12	4.52E+13
sp|Q9HAT2	6	63	25.28063	3.9E+14	1.54E+13	########	2.34E+15	9.71E+14
sp|Q9HAV0	6	63	34.32567	2.82E+16	8.21E+14	########	1.69E+17	5.17E+16
sp|Q9HAV7	6	63	40.15687	5.59E+14	1.39E+13	########	3.35E+15	8.76E+14
sp|Q9HB71	6	63	########	6.07E+14	7.13E+14	########	3.64E+15	4.49E+16
sp|Q9HB90	6	63	6.068322	1.56E+13	2.57E+12	########	9.35E+13	1.62E+14
sp|Q9HBF4	6	63	3.451515	8.53E+14	2.47E+14	########	5.12E+15	2E+16
sp|Q9HBH5	6	63	2.254732	3.53E+12	1.56E+12	########	2.12E+13	9.86E+13
sp|Q9HBI6	6	63	4.289942	5.31E+13	1.24E+13	########	3.18E+14	7.79E+14
sp|Q9HBL0	6	63	7.638459	3.41E+13	4.46E+12	########	2.04E+14	2.81E+14
sp|Q9HBL8	6	63	1.040038	5.96E+11	5.73E+11	########	3.57E+12	3.61E+13
sp|Q9HC38	6	63	168.4282	5.07E+16	3.01E+14	########	########	1.9E+16
sp|Q9HC56	6	63	########	1.26E+12	2E+12	########	7.54E+12	1.26E+14
sp|Q9HCC0	6	63	20.85777	9.11E+14	4.37E+13	########	5.47E+15	2.75E+15
sp|Q9HCD6	6	63	18.96837	1.66E+14	8.77E+12	########	9.99E+14	5.53E+14
sp|Q9HCH3	6	63	3.328549	1.35E+15	4.06E+14	########	8.11E+15	2.56E+16

For Peer Review Only

sp|Q9HCJ1	6	63	3.696416	1.45E+13	3.93E+12	########	8.72E+13	2.48E+14
sp|Q9HCJ6	6	63	28.289	2.72E+16	9.62E+14	########	1.63E+17	6.06E+16
sp|Q9HCM	6	63	14.82538	4.57E+15	3.08E+14	########	2.74E+16	2E+16
sp|Q9HCP6	6	63	14.79029	1.31E+14	8.86E+12	########	7.87E+14	5.58E+14
sp|Q9HD23	6	63	7.875719	6.45E+11	8.19E+10	########	3.87E+12	5.16E+12
sp|Q9HD34	6	63	14.3573	1.71E+14	1.19E+13	########	1.03E+15	7.51E+14
sp|Q9HD42	6	63	1.015602	2.34E+12	2.3E+12	########	1.4E+13	1.45E+14
sp|Q9HDC9	6	63	18.33505	3.33E+15	1.82E+14	########	2E+16	1E+16
sp|Q9NNW	6	63	31.02335	6.2E+14	2E+13	########	3.72E+15	1.26E+15
sp|Q9NP72	6	63	7.818425	1.06E+15	1.36E+14	########	6.37E+15	8.56E+15
sp|Q9NP78	6	63	3.030049	1.7E+13	5.61E+12	########	1.02E+14	3.53E+14
sp|Q9NP79	6	63	14.99917	7.11E+14	4.74E+13	########	4.27E+15	2.99E+15
sp|Q9NP80	6	63	25.68177	8.31E+13	3.23E+12	########	4.98E+14	2.04E+14
sp|Q9NP81	6	63	12.49302	3.09E+14	2.47E+13	########	1.85E+15	1.56E+15
sp|Q9NP97	6	63	1.934958	8.41E+14	4.35E+14	########	5.05E+15	2.74E+16
sp|Q9NPD7	6	63	5.100682	3.08E+14	6.04E+13	########	1.85E+15	3.81E+15
sp|Q9NPF4	6	63	15.32854	6.43E+13	4.19E+12	########	3.86E+14	2.64E+14
sp|Q9NPJ3	6	63	20.32402	3.49E+15	1.72E+14	########	2.09E+16	1.08E+16
sp|Q9NPQ8	6	63	7.122876	3.88E+13	5.45E+12	########	2.33E+14	3.43E+14
sp|Q9NQ48	6	63	########	3.78E+12	8.32E+12	########	2.27E+13	5.24E+14
sp|Q9NQ66	6	63	5.444894	5.48E+16	1.01E+16	########	3.29E+17	6.35E+17
sp|Q9NQ79	6	63	4.671126	1.7E+14	3.64E+13	########	1.02E+15	2.29E+15
sp|Q9NQ88	6	63	10.58696	1.4E+14	1.33E+13	########	8.42E+14	8.35E+14
sp|Q9NQC3	6	63	13.09198	1.64E+17	1.25E+16	########	9.82E+17	8E+17
sp|Q9NQC7	6	63	30.34521	5.12E+13	1.69E+12	########	3.07E+14	1.06E+14
sp|Q9NQE9	6	63	26.32821	8.14E+14	3.09E+13	########	4.88E+15	1.95E+15
sp|Q9NQP4	6	63	6.123223	5.74E+13	9.37E+12	########	3.44E+14	5.9E+14
sp|Q9NQR4	6	63	42.23764	1.38E+16	3.27E+14	########	8.29E+16	2.06E+16
sp|Q9NQW	6	63	10.63188	2.55E+16	2.4E+15	########	1.53E+17	1.51E+17
sp|Q9NQW	6	63	73.20607	1.19E+16	1.62E+14	########	7.12E+16	1.02E+16
sp|Q9NQX3	6	63	27.73523	3.1E+15	1.12E+14	########	1.86E+16	7.04E+15
sp|Q9NQX7	6	63	3.416452	7.57E+13	2.21E+13	########	4.54E+14	1.4E+15
sp|Q9NR28	6	63	29.84741	7.59E+14	2.54E+13	########	4.55E+15	1.6E+15
sp|Q9NR31	6	63	6.085843	1.04E+15	1.71E+14	########	6.23E+15	1.07E+16
sp|Q9NR45	6	63	3.75622	6.06E+14	1.61E+14	########	3.64E+15	1.02E+16
sp|Q9NR46	6	63	5.584155	1.66E+16	2.97E+15	########	9.96E+16	1.87E+17
sp|Q9NR48	6	63	########	7.93E+13	3.23E+14	########	4.76E+14	2E+16
sp|Q9NRA0	6	63	########	1.83E+11	3.16E+11	########	1.1E+12	1.99E+13
sp|Q9NRD9	6	63	1.428347	6.1E+12	4.27E+12	########	3.66E+13	2.69E+14
sp|Q9NRG7	6	63	56.93557	4.94E+15	8.68E+13	########	2.96E+16	5.47E+15
sp|Q9NRN7	6	63	########	7.86E+12	7.97E+12	########	4.72E+13	5.02E+14
sp|Q9NRQ2	6	63	2.259404	4.34E+13	1.92E+13	########	2.6E+14	1.21E+15
sp|Q9NRR3	6	63	1.521203	5.07E+13	3.33E+13	########	3.04E+14	2.1E+15
sp|Q9NRV9	6	63	########	2.47E+14	2.93E+14	########	1.48E+15	1.85E+16
sp|Q9NRW	6	63	94.58572	8.2E+16	8.67E+14	########	4.92E+17	5.46E+16
sp|Q9NRW	6	63	11.08883	6.09E+14	5.5E+13	########	3.66E+15	3.46E+15
sp|Q9NRX4	6	63	2.015809	2.05E+15	1.02E+15	########	1.23E+16	6.42E+16
sp|Q9NRY4	6	63	26.47303	5.26E+14	1.99E+13	########	3.16E+15	1.25E+15
sp|Q9NRY5	6	63	2.539732	5.22E+12	2.05E+12	########	3.13E+13	1.29E+14
sp|Q9NRY6	6	63	4.405207	9.26E+12	2.1E+12	########	5.56E+13	1.32E+14
sp|Q9NRZ7	6	63	4.543676	2.25E+13	4.95E+12	########	1.35E+14	3.12E+14
sp|Q9NS69	6	63	29.04035	1.6E+15	5.5E+13	########	9.59E+15	3.47E+15
sp|Q9NS86	6	63	6.655698	9.95E+14	1.49E+14	########	5.97E+15	9E+15
sp|Q9NSD9	6	63	12.87853	2.05E+17	1.59E+16	########	1.23E+18	1E+18
sp|Q9NSE4	6	63	111.6089	2.33E+16	2.09E+14	########	1.4E+17	1.32E+16
sp|Q9NSK0	6	63	########	1.85E+11	2.2E+11	########	1.11E+12	1.39E+13

For Peer Review Only

sp|Q9NSY(	6	63	8.577217	5.14E+13	5.99E+12	########	3.08E+14	3.77E+14
sp|Q9NT62	6	63	6.713075	2.5E+14	3.73E+13	########	1.5E+15	2.35E+15
sp|Q9NTI5|	6	63	89.66453	5.98E+13	6.67E+11	########	3.59E+14	4.2E+13
sp|Q9NTJ4	6	63	21.88888	1.26E+14	5.74E+12	########	7.54E+14	3.62E+14
sp|Q9NTJ5	6	63	27.33297	1.23E+15	4.51E+13	########	7.4E+15	3E+15
sp|Q9NTK5	6	63	2.562146	2.91E+15	1.14E+15	########	1.75E+16	7E+16
sp|Q9NTX5	6	63	4.052565	1.29E+14	3.18E+13	########	7.74E+14	2.01E+15
sp|Q9NUB1	6	63	21.56319	2.57E+15	1.19E+14	########	1.54E+16	8E+15
sp|Q9NUJ1	6	63	48.02419	1.75E+16	3.64E+14	########	1.05E+17	2.29E+16
sp|Q9NUJ3	6	63	6.210733	1.59E+14	2.56E+13	########	9.53E+14	2E+15
sp|Q9NUM4	6	63	21.64245	2.18E+15	1.01E+14	########	1.31E+16	6E+15
sp|Q9NUP9	6	63	12.52889	3.95E+14	3.15E+13	########	2.37E+15	1.99E+15
sp|Q9NUQ2	6	63	4.62672	2.6E+13	5.63E+12	########	1.56E+14	3.54E+14
sp|Q9NUQ9	6	63	199.3492	6.52E+16	3.27E+14	########	3.91E+17	2.06E+16
sp|Q9NUU7	6	63	3.593425	1.41E+13	3.92E+12	########	8.44E+13	2.47E+14
sp|Q9NV70	6	63	29.46609	5.99E+14	2.03E+13	########	3.6E+15	1.28E+15
sp|Q9NV96	6	63	11.75002	2.53E+14	2.15E+13	########	1.52E+15	1.35E+15
sp|Q9NVA2	6	63	8.904887	1.12E+17	1.25E+16	########	7E+17	7.9E+17
sp|Q9NVE7	6	63	28.95538	2.75E+14	9.5E+12	########	1.65E+15	5.98E+14
sp|Q9NVH1	6	63	39.86284	6.06E+15	1.52E+14	########	3.63E+16	9.57E+15
sp|Q9NVI7|	6	63	61.44821	3.46E+15	5.63E+13	########	2.08E+16	3.55E+15
sp|Q9NVJ2	6	63	14.8167	8.6E+15	5.81E+14	########	5.16E+16	3.66E+16
sp|Q9NVS9	6	63	26.16696	2.43E+16	9.27E+14	########	########	5.84E+16
sp|Q9NVT9	6	63	43.13663	1.78E+15	4.13E+13	########	1.07E+16	2.6E+15
sp|Q9NWS	6	63	14.2975	1.14E+14	7.99E+12	########	6.86E+14	5.03E+14
sp|Q9NWU	6	63	14.50324	9.36E+13	6.46E+12	########	5.62E+14	4.07E+14
sp|Q9NWV	6	63	9.608454	3.52E+14	3.67E+13	########	2.11E+15	2E+15
sp|Q9NX14	6	63	9.424238	8.02E+14	8.5E+13	########	4.81E+15	5.36E+15
sp|Q9NX40	6	63	39.2648	1.49E+15	3.79E+13	########	8.94E+15	2.39E+15
sp|Q9NX63	6	63	42.44535	3.77E+15	8.89E+13	########	2.26E+16	5.6E+15
sp|Q9NXA8	6	63	11.67106	4.63E+13	3.97E+12	########	2.78E+14	2.5E+14
sp|Q9NY33	6	63	20.66765	3.8E+15	1.84E+14	########	2.28E+16	1.16E+16
sp|Q9NY35	6	63	4.938349	4.04E+14	8.17E+13	########	2.42E+15	5E+15
sp|Q9NY47	6	63	1.871245	9.44E+13	5.05E+13	########	5.67E+14	3.18E+15
sp|Q9NY65	6	63	25.44891	2.31E+16	9.07E+14	########	1.38E+17	5.71E+16
sp|Q9NYB0	6	63	8.298696	1.84E+13	2.22E+12	########	1.11E+14	1.4E+14
sp|Q9NYB9	6	63	27.66256	3.68E+15	1.33E+14	########	2.21E+16	8E+15
sp|Q9NYF8	6	63	8.132848	1.79E+13	2.2E+12	########	1.07E+14	1.39E+14
sp|Q9NYI0|	6	63	21.25233	4.53E+16	2.13E+15	########	2.72E+17	1E+17
sp|Q9NYU2	6	63	26.09496	4.35E+15	1.67E+14	########	2.61E+16	1.05E+16
sp|Q9NZ01	6	63	8.939403	1.16E+15	1.3E+14	########	6.96E+15	8.18E+15
sp|Q9NZ32	6	63	14.21216	8.1E+14	5.7E+13	########	4.86E+15	3.59E+15
sp|Q9NZ45	6	63	64.79538	2.21E+17	3.42E+15	########	1.33E+18	2E+17
sp|Q9NZ52	6	63	4.691638	2.3E+13	4.91E+12	########	1.38E+14	3.09E+14
sp|Q9NZ56	6	63	1.852518	7.36E+13	3.97E+13	########	4.42E+14	3E+15
sp|Q9NZB8	6	63	8.940544	2.62E+13	2.94E+12	########	1.57E+14	1.85E+14
sp|Q9NZD2	6	63	########	4.7E+15	5.39E+15	########	2.82E+16	3E+17
sp|Q9NZG7	6	63	7.012039	2.13E+14	3.04E+13	########	1.28E+15	1.92E+15
sp|Q9NZH0	6	63	4.782451	1.94E+14	4.05E+13	########	1.16E+15	3E+15
sp|Q9NZJ4	6	63	5.629292	3.06E+12	5.44E+11	########	1.84E+13	3.43E+13
sp|Q9NZJ6	6	63	2.608966	5.84E+13	2.24E+13	########	3.51E+14	1.41E+15
sp|Q9NZJ7	6	63	66.24353	6.32E+15	9.54E+13	########	3.79E+16	6.01E+15
sp|Q9NZL4	6	63	37.97137	1.83E+14	4.81E+12	########	1.1E+15	3.03E+14
sp|Q9NZL9	6	63	104.1573	9.36E+15	8.99E+13	########	5.62E+16	5.66E+15
sp|Q9NZM1	6	63	1.648392	1.36E+13	8.27E+12	########	8.18E+13	5.21E+14
sp|Q9NZN3	6	63	45.29878	1.69E+17	3.73E+15	########	1.01E+18	2.35E+17

For Peer Review

sp|Q9NZN4	6	63	17.06847	3.07E+15	1.8E+14	########	1.84E+16	1.13E+16
sp|Q9NZQ3	6	63	44.60842	2.22E+15	4.97E+13	########	1.33E+16	3E+15
sp|Q9NZR1	6	63	4.730655	9.53E+15	2.02E+15	########	5.72E+16	1.27E+17
sp|Q9NZW	6	63	10.39008	9.9E+13	9.53E+12	########	5.94E+14	6E+14
sp|Q9NZZ3	6	63	########	2.13E+13	2.15E+13	########	1.28E+14	1E+15
sp|Q9P000	6	63	4.877168	9.5E+13	1.95E+13	########	5.7E+14	1E+15
sp|Q9P032	6	63	8.139987	8.19E+13	1.01E+13	########	4.91E+14	6.34E+14
sp|Q9P035	6	63	28.25808	1.55E+16	5.5E+14	########	9.32E+16	3.46E+16
sp|Q9P0J0	6	63	45.92408	5.11E+16	1.11E+15	########	3.07E+17	7.01E+16
sp|Q9P0J1	6	63	12.30757	3.62E+13	2.94E+12	########	2.17E+14	1.85E+14
sp|Q9P0J7	6	63	22.36427	6.66E+12	2.98E+11	########	4E+13	1.88E+13
sp|Q9P0K1	6	63	13.5297	2.25E+16	1.67E+15	########	1.35E+17	1.05E+17
sp|Q9P0L0	6	63	########	1.27E+14	3.08E+14	########	7.61E+14	1.94E+16
sp|Q9P0L2	6	63	4.746232	2.15E+14	4.53E+13	########	1.29E+15	2.85E+15
sp|Q9P0S9	6	63	14.4547	1.19E+14	8.25E+12	########	7.16E+14	5.2E+14
sp|Q9P0U1	6	63	12.98976	3.61E+13	2.78E+12	########	2.16E+14	1.75E+14
sp|Q9P0V9	6	63	1.051832	1.4E+14	1.33E+14	########	8.42E+14	8.41E+15
sp|Q9P0Z9	6	63	15.18708	5.7E+12	3.76E+11	########	3.42E+13	2.37E+13
sp|Q9P121	6	63	9.646446	3.35E+15	3.47E+14	########	2.01E+16	2.19E+16
sp|Q9P1F3	6	63	87.68827	1.3E+16	1.49E+14	########	7.83E+16	9E+15
sp|Q9P1U1	6	63	1.724783	1.65E+14	9.57E+13	########	9.9E+14	6.03E+15
sp|Q9P1Y5	6	63	10.79614	8.52E+13	7.89E+12	########	5.11E+14	4.97E+14
sp|Q9P253	6	63	8.915015	4.28E+13	4.8E+12	########	2.57E+14	3.02E+14
sp|Q9P260	6	63	24.63976	1.02E+15	4.12E+13	########	6.1E+15	2.6E+15
sp|Q9P265	6	63	17.63984	1.61E+15	9.12E+13	########	9.65E+15	5.75E+15
sp|Q9P2E9	6	63	7.998536	5.47E+13	6.84E+12	########	3.28E+14	4.31E+14
sp|Q9P2G1	6	63	3.846778	2.81E+14	7.3E+13	########	1.69E+15	4.6E+15
sp|Q9P2J5	6	63	16.99139	6.08E+15	3.58E+14	########	3.65E+16	2E+16
sp|Q9P2K5	6	63	32.21074	9.3E+14	2.89E+13	########	5.58E+15	1.82E+15
sp|Q9P2R3	6	63	16.89515	5.46E+15	3.23E+14	########	3.28E+16	2.04E+16
sp|Q9P2R7	6	63	116.5925	3.46E+17	2.97E+15	########	2.07E+18	1.87E+17
sp|Q9P2T1	6	63	143.3595	9.41E+15	6.56E+13	########	5.64E+16	4E+15
sp|Q9P2U7	6	63	13.3485	5.72E+16	4.28E+15	########	3.43E+17	2.7E+17
sp|Q9P2W3	6	63	4.264492	1.16E+14	2.72E+13	########	6.95E+14	1.71E+15
sp|Q9UBB4	6	63	3.997872	9.99E+13	2.5E+13	########	5.99E+14	1.57E+15
sp|Q9UBB6	6	63	6.986031	########	3.74E+16	########	2E+18	2E+18
sp|Q9UBC2	6	63	2.663538	4.14E+14	1.56E+14	########	2.49E+15	1E+16
sp|Q9UBE0	6	63	10.67717	1E+14	9.39E+12	########	6.02E+14	5.92E+14
sp|Q9UBF2	6	63	15.76884	3.01E+13	1.91E+12	########	1.8E+14	1.2E+14
sp|Q9UBI6	6	63	16.5255	3.13E+16	1.89E+15	########	1.88E+17	1.19E+17
sp|Q9UBN7	6	63	8.645769	1.33E+13	1.54E+12	########	7.98E+13	9.69E+13
sp|Q9UBP0	6	63	4.417973	7.61E+12	1.72E+12	########	4.57E+13	1.09E+14
sp|Q9UBP4	6	63	19.80327	3.65E+15	1.85E+14	########	2.19E+16	1.16E+16
sp|Q9UBQ0	6	63	15.41508	3.13E+15	2.03E+14	########	1.88E+16	1.28E+16
sp|Q9UBQ5	6	63	########	1.93E+11	5.39E+11	########	1.16E+12	3.39E+13
sp|Q9UBQ7	6	63	78.56145	1.27E+17	1.62E+15	########	7.62E+17	1.02E+17
sp|Q9UBS5	6	63	12.108	1.07E+15	8.86E+13	########	6.44E+15	6E+15
sp|Q9UBT2	6	63	20.94781	7.87E+14	3.76E+13	########	4.72E+15	2E+15
sp|Q9UBV8	6	63	37.82825	2.53E+15	6.7E+13	########	1.52E+16	4.22E+15
sp|Q9UBW	6	63	4.109535	9.46E+13	2.3E+13	########	5.68E+14	1.45E+15
sp|Q9UBX3	6	63	2.98064	4.47E+13	1.5E+13	########	2.68E+14	9.44E+14
sp|Q9UD71	6	63	11.5494	1.85E+14	1.6E+13	########	1.11E+15	1E+15
sp|Q9UDT6	6	63	12.81129	6.83E+14	5.33E+13	########	4.1E+15	3.36E+15
sp|Q9UDW	6	63	33.57772	3.86E+15	1.15E+14	########	2.32E+16	7.25E+15
sp|Q9UDY2	6	63	3.403872	1.29E+15	3.78E+14	########	7.71E+15	2.38E+16
sp|Q9UDY4	6	63	14.67335	6.77E+14	4.61E+13	########	4.06E+15	2.91E+15

For Peer Review Only

sp|Q9UEW	6	63	35.46423	8.08E+15	2.28E+14	########	4.85E+16	1.44E+16
sp|Q9UEY8	6	63	########	7.05E+14	3.07E+15	########	4.23E+15	1.94E+17
sp|Q9UF11	6	63	7.356111	2.05E+15	2.79E+14	########	1.23E+16	1.76E+16
sp|Q9UFN(	6	63	4.030662	7.16E+13	1.78E+13	########	4.3E+14	1.12E+15
sp|Q9UGC(	6	63	1.780325	1.57E+13	8.83E+12	########	9.43E+13	5.56E+14
sp|Q9UGI6	6	63	5.362611	7.55E+13	1.41E+13	########	4.53E+14	8.87E+14
sp|Q9UGM	6	63	14.51145	3.15E+13	2.17E+12	########	1.89E+14	1.37E+14
sp|Q9UGT4	6	63	3.961998	2.61E+13	6.59E+12	########	1.57E+14	4.15E+14
sp|Q9UGV2	6	63	33.53418	1.24E+16	3.69E+14	########	7.42E+16	2.32E+16
sp|Q9UH03	6	63	9.050158	8.21E+16	9.07E+15	########	4.93E+17	5.71E+17
sp|Q9UH99	6	63	39.99186	1.36E+15	3.41E+13	########	8.19E+15	2.15E+15
sp|Q9UHA4	6	63	16.09446	7.24E+14	4.5E+13	########	4.35E+15	3E+15
sp|Q9UHB9	6	63	4.794272	1.24E+14	2.59E+13	########	7.46E+14	1.63E+15
sp|Q9UHC(	6	63	13.54925	3.84E+15	2.84E+14	########	2.31E+16	1.79E+16
sp|Q9UHD1	6	63	12.95837	1.08E+15	8.3E+13	########	6.45E+15	5.23E+15
sp|Q9UHD2	6	63	16.59233	2.34E+13	1.41E+12	########	1.41E+14	8.9E+13
sp|Q9UHD8	6	63	20.50108	2.63E+16	1.28E+15	########	2E+17	8.08E+16
sp|Q9UHG2	6	63	7.572791	1.09E+16	1.44E+15	########	6.54E+16	9.07E+16
sp|Q9UHG3	6	63	50.44318	7.58E+15	1.5E+14	########	4.55E+16	9.46E+15
sp|Q9UHI5	6	63	2.097797	8.89E+11	4.24E+11	########	5.33E+12	2.67E+13
sp|Q9UHL4	6	63	57.17743	4.87E+15	8.51E+13	########	2.92E+16	5.36E+15
sp|Q9UHQ4	6	63	3.552184	5.14E+13	1.45E+13	########	3.09E+14	9.12E+14
sp|Q9UHQ9	6	63	25.36304	2.27E+15	8.96E+13	########	1.36E+16	6E+15
sp|Q9UHV9	6	63	80.92891	2.47E+15	3.05E+13	########	1.48E+16	1.92E+15
sp|Q9UHX1	6	63	11.65276	2.26E+13	1.94E+12	########	1.36E+14	1.22E+14
sp|Q9UHY1	6	63	29.89202	1.76E+15	5.88E+13	########	1.05E+16	4E+15
sp|Q9UHY7	6	63	19.49005	2.98E+16	1.53E+15	########	1.79E+17	9.63E+16
sp|Q9UI09|	6	63	40.09804	3.01E+16	7.52E+14	########	1.81E+17	4.74E+16
sp|Q9UI12|	6	63	2.686806	2.28E+16	8.49E+15	########	1.37E+17	5.35E+17
sp|Q9UI15|	6	63	73.18394	2.43E+17	3.32E+15	########	1.46E+18	2.09E+17
sp|Q9UI40|	6	63	7.54633	3.58E+14	4.74E+13	########	2.15E+15	2.98E+15
sp|Q9UI47|	6	63	6.450164	3.19E+14	4.94E+13	########	1.91E+15	3.11E+15
sp|Q9UIA9|	6	63	1.013074	3.77E+13	3.72E+13	########	2.26E+14	2.34E+15
sp|Q9UIC8	6	63	25.58689	1.39E+15	5.43E+13	########	8.33E+15	3.42E+15
sp|Q9UID3	6	63	24.68756	7.31E+14	2.96E+13	########	4.38E+15	2E+15
sp|Q9UIG0	6	63	64.2885	6.76E+16	1.05E+15	########	4.06E+17	6.62E+16
sp|Q9UII2|A	6	63	2.115576	2.94E+14	1.39E+14	########	1.76E+15	8.74E+15
sp|Q9UIJ7|	6	63	16.81378	1.6E+15	9.53E+13	########	9.62E+15	6.01E+15
sp|Q9UIQ6	6	63	1.506112	3.03E+13	2.01E+13	########	1.82E+14	1.27E+15
sp|Q9UIW2	6	63	7.103024	1.98E+15	2.79E+14	########	1.19E+16	1.76E+16
sp|Q9UJ14	6	63	10.02405	3.07E+14	3.06E+13	########	1.84E+15	1.93E+15
sp|Q9UJ41	6	63	5.043034	1.19E+13	2.37E+12	########	7.17E+13	1.49E+14
sp|Q9UJ70	6	63	8.690923	1.18E+15	1.35E+14	########	7.06E+15	8.53E+15
sp|Q9UJC5	6	63	1.652158	3.59E+14	2.17E+14	########	2.15E+15	1.37E+16
sp|Q9UJD0	6	63	6.652568	1.54E+13	2.31E+12	########	9.23E+13	1.46E+14
sp|Q9UJQ1	6	63	########	5.25E+11	5.86E+11	########	3.15E+12	3.69E+13
sp|Q9UJS0	6	63	33.75804	6.57E+15	1.95E+14	########	3.94E+16	1.23E+16
sp|Q9UJU6	6	63	6.469527	3.94E+14	6.09E+13	########	2.36E+15	4E+15
sp|Q9UJW0	6	63	14.6631	2.02E+15	1.38E+14	########	1.21E+16	8.67E+15
sp|Q9UJY5	6	63	78.03993	2.98E+14	3.81E+12	########	1.79E+15	2.4E+14
sp|Q9UJZ1	6	63	88.38625	8.1E+15	9.17E+13	########	4.86E+16	5.77E+15
sp|Q9UK22	6	63	9.335532	8.99E+16	9.63E+15	########	5.4E+17	6.07E+17
sp|Q9UK41	6	63	3.693583	2.53E+13	6.86E+12	########	1.52E+14	4.32E+14
sp|Q9UK45	6	63	12.3636	3.48E+13	2.82E+12	########	2.09E+14	1.77E+14
sp|Q9UK76	6	63	34.0764	4.01E+15	1.18E+14	########	2.41E+16	7.42E+15
sp|Q9UKA9	6	63	58.7478	1.23E+15	2.09E+13	########	7.36E+15	1.32E+15

For Peer Review Only

sp|Q9UKG	6	63	1.479303	2.36E+14	1.6E+14	########	1.42E+15	1E+16
sp|Q9UKK	6	63	24.01464	3.86E+15	1.61E+14	########	2.31E+16	1E+16
sp|Q9UKU	6	63	18.29037	1.83E+16	1E+15	########	1.1E+17	6.32E+16
sp|Q9UKU	6	63	2.16811	2.16E+13	9.94E+12	########	1.29E+14	6.26E+14
sp|Q9UL12	6	63	2.703559	1.58E+15	5.84E+14	########	9.48E+15	3.68E+16
sp|Q9UL15	6	63	14.45611	1.39E+14	9.65E+12	########	8.37E+14	6.08E+14
sp|Q9UL25	6	63	7.229361	3.53E+14	4.88E+13	########	2.12E+15	3E+15
sp|Q9UL26	6	63	5.481839	1.05E+14	1.91E+13	########	6.28E+14	1.2E+15
sp|Q9UL46	6	63	65.98046	1.16E+15	1.76E+13	########	6.96E+15	1.11E+15
sp|Q9UL51	6	63	4.818336	5.05E+13	1.05E+13	########	3.03E+14	6.6E+14
sp|Q9ULAC	6	63	35.36765	2.58E+15	7.29E+13	########	1.55E+16	4.6E+15
sp|Q9ULB1	6	63	4.761342	8.43E+13	1.77E+13	########	5.06E+14	1E+15
sp|Q9ULC3	6	63	7.864862	1.96E+14	2.49E+13	########	1.17E+15	1.57E+15
sp|Q9ULC4	6	63	3.479884	8.18E+13	2.35E+13	########	4.91E+14	1.48E+15
sp|Q9ULDC	6	63	66.68074	1.9E+17	2.85E+15	########	1.14E+18	1.8E+17
sp|Q9ULJ8	6	63	8.795906	9.98E+13	1.13E+13	########	5.99E+14	7.15E+14
sp|Q9ULKC	6	63	2.554763	2.07E+12	8.12E+11	########	1.24E+13	5.12E+13
sp|Q9ULN7	6	63	1.989835	1.97E+13	9.88E+12	########	1.18E+14	6.23E+14
sp|Q9ULPC	6	63	17.20416	1.18E+17	6.85E+15	########	7.07E+17	4.31E+17
sp|Q9ULP9	6	63	4.156148	1.65E+15	3.96E+14	########	9.88E+15	2.5E+16
sp|Q9ULR3	6	63	5.105231	1.29E+15	2.52E+14	########	7.72E+15	2E+16
sp|Q9ULT8	6	63	1.911001	1.71E+16	8.94E+15	########	1.02E+17	########
sp|Q9ULU8	6	63	14.35356	2.72E+16	1.89E+15	########	1.63E+17	1.19E+17
sp|Q9ULV4	6	63	8.841489	2.03E+15	2.29E+14	########	1.22E+16	1E+16
sp|Q9UM19	6	63	11.51223	2.5E+15	2.18E+14	########	1.5E+16	1.37E+16
sp|Q9UM22	6	63	7.579335	2.63E+14	3.47E+13	########	1.58E+15	2.19E+15
sp|Q9UM54	6	63	1.638173	9.66E+13	5.9E+13	########	5.79E+14	4E+15
sp|Q9UMFC	6	63	########	1.04E+15	1.42E+15	########	6.22E+15	8.94E+16
sp|Q9UMS4	6	63	9.793837	1.07E+15	1.09E+14	########	6.42E+15	6.88E+15
sp|Q9UMXC	6	63	6.042302	8.55E+13	1.41E+13	########	5.13E+14	8.91E+14
sp|Q9UMX9	6	63	1.883862	9.82E+12	5.21E+12	########	5.89E+13	3.29E+14
sp|Q9UMY4	6	63	########	2.26E+13	3.24E+13	########	1.36E+14	2.04E+15
sp|Q9UN36	6	63	39.73513	2.74E+18	6.89E+16	########	2E+19	4E+18
sp|Q9UN37	6	63	8.647381	1.4E+14	1.61E+13	########	8.38E+14	1.02E+15
sp|Q9UN86	6	63	9.456768	1.16E+15	1.23E+14	########	6.97E+15	7.74E+15
sp|Q9UNA1	6	63	2.013197	9.84E+12	4.89E+12	########	5.9E+13	3.08E+14
sp|Q9UNE7	6	63	9.039461	6.53E+13	7.22E+12	########	3.92E+14	4.55E+14
sp|Q9UNFC	6	63	12.71925	7.14E+14	5.61E+13	########	4.28E+15	3.54E+15
sp|Q9UNH7	6	63	7.560209	2.64E+14	3.5E+13	########	1.59E+15	2.2E+15
sp|Q9UNKC	6	63	2.728418	9.91E+11	3.63E+11	########	5.95E+12	2.29E+13
sp|Q9UNM	6	63	30.1704	1.12E+15	3.72E+13	########	6.73E+15	2.34E+15
sp|Q9UNN9	6	63	1.589862	8.19E+12	5.15E+12	########	4.91E+13	3.24E+14
sp|Q9UNS2	6	63	2.490728	6E+14	2.41E+14	########	3.6E+15	1.52E+16
sp|Q9UNW	6	63	23.55969	4.51E+14	1.91E+13	########	2.7E+15	1.2E+15
sp|Q9UNZ2	6	63	4.594159	4.83E+14	1.05E+14	########	2.9E+15	6.62E+15
sp|Q9UPA5	6	63	28.23665	1.01E+17	3.58E+15	########	6.07E+17	2.26E+17
sp|Q9UPN3	6	63	23.47398	4.7E+15	2E+14	########	2.82E+16	1.26E+16
sp|Q9UPQC	6	63	20.27691	4.31E+14	2.13E+13	########	2.59E+15	1.34E+15
sp|Q9UPQ3	6	63	32.00944	1.66E+14	5.18E+12	########	9.95E+14	3.26E+14
sp|Q9UPRC	6	63	7.287737	2.92E+13	4E+12	########	1.75E+14	2.52E+14
sp|Q9UPR5	6	63	19.3007	2.95E+16	1.53E+15	########	1.77E+17	9.64E+16
sp|Q9UPT5	6	63	5.280158	1E+14	1.9E+13	########	6E+14	1.19E+15
sp|Q9UPT6	6	63	16.89709	7.17E+13	4.24E+12	########	4.3E+14	2.67E+14
sp|Q9UPU5	6	63	13.27239	5.25E+12	3.96E+11	########	3.15E+13	2.49E+13
sp|Q9UPV7	6	63	23.57583	1.25E+16	5.31E+14	########	7.51E+16	3.35E+16
sp|Q9UPW	6	63	17.31687	9.29E+14	5.36E+13	########	5.57E+15	3.38E+15

For Peer Review Only

sp|Q9UPX(	6	63	20.45978	1.08E+15	5.26E+13	########	6.45E+15	3.31E+15
sp|Q9UPX8	6	63	3.310715	8.87E+12	2.68E+12	########	5.32E+13	1.69E+14
sp|Q9UPY5	6	63	22.72472	2.32E+14	1.02E+13	########	1.39E+15	6.43E+14
sp|Q9UPY6	6	63	13.79883	4.34E+14	3.14E+13	########	2.6E+15	1.98E+15
sp|Q9UPY8	6	63	4.377672	7.45E+15	1.7E+15	########	4.47E+16	1E+17
sp|Q9UQ03	6	63	6.765845	8.4E+15	1.24E+15	########	5.04E+16	7.82E+16
sp|Q9UQ16	6	63	74.16357	########	3.21E+15	########	1E+18	2.02E+17
sp|Q9UQ80	6	63	50.57226	3.76E+16	7.44E+14	########	2.26E+17	5E+16
sp|Q9UQ90	6	63	21.43918	8.39E+13	3.91E+12	########	5.04E+14	2.47E+14
sp|Q9UQB3	6	63	30.03461	1.35E+16	4.51E+14	########	8.13E+16	2.84E+16
sp|Q9UQB8	6	63	17.47848	5.16E+15	2.95E+14	########	3.1E+16	2E+16
sp|Q9UQM	6	63	23.08479	1E+19	6.18E+17	########	9E+19	4E+19
sp|Q9Y224	6	63	20.08564	1E+16	4.98E+14	########	6E+16	3.14E+16
sp|Q9Y230	6	63	8.933817	1.45E+14	1.62E+13	########	8.71E+14	1.02E+15
sp|Q9Y241	6	63	34.6678	1.05E+15	3.04E+13	########	6.32E+15	1.91E+15
sp|Q9Y262	6	63	3.558241	1.18E+14	3.31E+13	########	7.07E+14	2.09E+15
sp|Q9Y263	6	63	1.502311	2.97E+12	1.98E+12	########	1.78E+13	1.25E+14
sp|Q9Y265	6	63	23.01197	1.94E+15	8.42E+13	########	1.16E+16	5.3E+15
sp|Q9Y266	6	63	5.052555	1.49E+14	2.95E+13	########	8.94E+14	1.86E+15
sp|Q9Y276	6	63	64.94854	1.09E+15	1.68E+13	########	6.54E+15	1.06E+15
sp|Q9Y277	6	63	41.41633	2.18E+17	5.26E+15	########	1.31E+18	########
sp|Q9Y281	6	63	28.8611	1.22E+16	4.24E+14	########	7.33E+16	2.67E+16
sp|Q9Y285	6	63	14.15389	9.29E+16	6.56E+15	########	########	4.13E+17
sp|Q9Y287	6	63	9.119059	1.64E+14	1.8E+13	########	9.84E+14	1E+15
sp|Q9Y295	6	63	1.166076	2.91E+12	2.49E+12	########	1.74E+13	1.57E+14
sp|Q9Y2A7	6	63	61.12065	9.03E+16	1.48E+15	########	5.42E+17	9E+16
sp|Q9Y2B0	6	63	21.04393	3.85E+14	1.83E+13	########	2.31E+15	1.15E+15
sp|Q9Y2C4	6	63	6.650915	2.67E+13	4.01E+12	########	1.6E+14	2.53E+14
sp|Q9Y2D4	6	63	5.091049	4.52E+13	8.89E+12	########	2.71E+14	5.6E+14
sp|Q9Y2E4	6	63	########	2.39E+13	2.95E+13	########	1.43E+14	1.86E+15
sp|Q9Y2G8	6	63	4.460546	6.12E+12	1.37E+12	########	3.67E+13	8.64E+13
sp|Q9Y2H1	6	63	2.584447	8.63E+13	3.34E+13	########	5.18E+14	2.1E+15
sp|Q9Y2H9	6	63	1.791224	3.73E+11	2.08E+11	########	2.24E+12	1.31E+13
sp|Q9Y2I1|	6	63	1.912316	4.04E+13	2.11E+13	########	2.42E+14	1.33E+15
sp|Q9Y2I8|	6	63	12.17796	1.41E+15	1.15E+14	########	8.43E+15	7.27E+15
sp|Q9Y2J0	6	63	5.529341	1.3E+16	2.34E+15	########	7.77E+16	1.48E+17
sp|Q9Y2J2	6	63	26.51711	1.17E+18	4.43E+16	########	7E+18	3E+18
sp|Q9Y2J8	6	63	11.87271	1.39E+17	1.17E+16	########	8.32E+17	7E+17
sp|Q9Y2K9	6	63	6.327881	2.87E+14	4.53E+13	########	1.72E+15	2.85E+15
sp|Q9Y2Q0	6	63	16.90799	2.68E+16	1.58E+15	########	1.61E+17	9.97E+16
sp|Q9Y2Q3	6	63	86.89825	2.55E+15	2.93E+13	########	1.53E+16	1.85E+15
sp|Q9Y2Q5	6	63	11.03573	1.37E+14	1.25E+13	########	8.25E+14	7.85E+14
sp|Q9Y2S2	6	63	59.83492	3E+16	5.02E+14	########	1.8E+17	3.16E+16
sp|Q9Y2T2	6	63	1.855125	1.55E+12	8.34E+11	########	9.28E+12	5.25E+13
sp|Q9Y2T3	6	63	75.15901	7.73E+17	1.03E+16	########	5E+18	6.48E+17
sp|Q9Y2V2	6	63	18.96828	2.33E+15	1.23E+14	########	1.4E+16	7.75E+15
sp|Q9Y2X3	6	63	23.35779	1.06E+14	4.53E+12	########	6.34E+14	2.85E+14
sp|Q9Y2X7	6	63	98.98531	9.4E+15	9.5E+13	########	5.64E+16	5.98E+15
sp|Q9Y2Z0	6	63	4.39532	2.11E+14	4.79E+13	########	1.26E+15	3.02E+15
sp|Q9Y2Z4	6	63	16.76991	2.54E+14	1.51E+13	########	2E+15	9.54E+14
sp|Q9Y2Z9	6	63	9.200908	1.77E+13	1.92E+12	########	1.06E+14	1.21E+14
sp|Q9Y305	6	63	26.81554	6.21E+15	2.32E+14	########	3.73E+16	1.46E+16
sp|Q9Y320	6	63	9.129068	3.58E+13	3.92E+12	########	2.15E+14	2.47E+14
sp|Q9Y333	6	63	7.49015	4.15E+14	5.54E+13	########	2.49E+15	3.49E+15
sp|Q9Y342	6	63	########	5.15E+12	5.95E+12	########	3.09E+13	3.75E+14
sp|Q9Y371	6	63	10.18544	9.2E+14	9.03E+13	########	5.52E+15	5.69E+15

For Peer Review Only

sp|Q9Y376	6	63	47.96384	8.8E+15	1.84E+14	########	5.28E+16	1.16E+16
sp|Q9Y394	6	63	34.07518	1.23E+16	3.61E+14	########	7.39E+16	2.28E+16
sp|Q9Y3A5	6	63	29.75476	4.74E+15	1.59E+14	########	2.85E+16	1E+16
sp|Q9Y3B3	6	63	4.911159	8.76E+13	1.78E+13	########	5.25E+14	1.12E+15
sp|Q9Y3B8	6	63	9.572081	1.1E+13	1.15E+12	########	6.6E+13	7.24E+13
sp|Q9Y3C4	6	63	4.884013	1.51E+12	3.1E+11	########	9.08E+12	1.95E+13
sp|Q9Y3C6	6	63	7.716326	1.89E+13	2.44E+12	########	1.13E+14	1.54E+14
sp|Q9Y3C8	6	63	2.002493	5.66E+12	2.83E+12	########	3.4E+13	1.78E+14
sp|Q9Y3D6	6	63	26.85219	5.56E+15	2.07E+14	########	3.34E+16	1.3E+16
sp|Q9Y3E1	6	63	80.6584	8.52E+15	1.06E+14	########	5.11E+16	6.66E+15
sp|Q9Y3F4	6	63	6.153699	3.34E+14	5.42E+13	########	2E+15	3E+15
sp|Q9Y3I0|	6	63	11.63766	1.01E+16	8.71E+14	########	6.08E+16	5.49E+16
sp|Q9Y3P9	6	63	2.971658	1.67E+13	5.61E+12	########	1E+14	3.53E+14
sp|Q9Y3U8	6	63	51.19815	9E+15	1.76E+14	########	5.4E+16	1.11E+16
sp|Q9Y3Z3	6	63	34.94252	3.61E+14	1.03E+13	########	2.17E+15	6.51E+14
sp|Q9Y426	6	63	20.20409	7.67E+13	3.8E+12	########	4.6E+14	2.39E+14
sp|Q9Y490	6	63	7.169119	6.63E+15	9.24E+14	########	3.98E+16	5.82E+16
sp|Q9Y496	6	63	15.43668	6.42E+13	4.16E+12	########	3.85E+14	2.62E+14
sp|Q9Y4C0	6	63	3.627357	1.46E+14	4.03E+13	########	8.77E+14	2.54E+15
sp|Q9Y4D1	6	63	4.818706	1.37E+13	2.85E+12	########	8.24E+13	1.8E+14
sp|Q9Y4D8	6	63	10.70339	4.79E+13	4.48E+12	########	2.88E+14	2.82E+14
sp|Q9Y4E1	6	63	3.863596	8.58E+12	2.22E+12	########	5.15E+13	1.4E+14
sp|Q9Y4E6	6	63	10.9535	1.38E+16	1.26E+15	########	8.3E+16	7.96E+16
sp|Q9Y4E8	6	63	6.709513	9.03E+13	1.35E+13	########	5.42E+14	8.47E+14
sp|Q9Y4F1	6	63	15.36698	7.67E+15	4.99E+14	########	4.6E+16	3E+16
sp|Q9Y4F5	6	63	21.57932	2.3E+15	1.07E+14	########	1.38E+16	6.73E+15
sp|Q9Y4G6	6	63	33.745	7.42E+16	2.2E+15	########	4.45E+17	########
sp|Q9Y4G8	6	63	38.41022	3.21E+15	8.36E+13	########	1.93E+16	5.27E+15
sp|Q9Y4I1|	6	63	49.2104	3.77E+17	7.66E+15	########	2.26E+18	4.82E+17
sp|Q9Y4J8	6	63	4.26255	1.65E+14	3.87E+13	########	9.89E+14	2.44E+15
sp|Q9Y4L1	6	63	39.64128	3.75E+16	9.47E+14	########	2.25E+17	5.97E+16
sp|Q9Y4P1	6	63	1.536045	1.14E+13	7.43E+12	########	6.85E+13	4.68E+14
sp|Q9Y4P8	6	63	5.562766	5.69E+14	1.02E+14	########	3.41E+15	6E+15
sp|Q9Y4W6	6	63	54.94795	1.07E+16	1.95E+14	########	6.43E+16	1.23E+16
sp|Q9Y4Y9	6	63	4.087718	9.89E+13	2.42E+13	########	5.93E+14	1.52E+15
sp|Q9Y512	6	63	39.54785	9.21E+15	2.33E+14	########	5.52E+16	1.47E+16
sp|Q9Y536	6	63	1.018372	5.23E+14	5.14E+14	########	3.14E+15	3.24E+16
sp|Q9Y566	6	63	2.614258	5.84E+11	2.23E+11	########	3.5E+12	1.41E+13
sp|Q9Y570	6	63	3.656958	1.75E+15	4.8E+14	########	1.05E+16	3E+16
sp|Q9Y584	6	63	3.063063	1.94E+12	6.33E+11	########	1.16E+13	3.99E+13
sp|Q9Y5J7	6	63	22.8991	1.37E+15	6E+13	########	8.24E+15	3.78E+15
sp|Q9Y5K5	6	63	1.579995	4.58E+12	2.9E+12	########	2.75E+13	1.83E+14
sp|Q9Y5K8	6	63	5.062337	1.51E+16	2.99E+15	########	9.08E+16	1.88E+17
sp|Q9Y5L0	6	63	40.70091	4.25E+14	1.04E+13	########	2.55E+15	6.57E+14
sp|Q9Y5L3	6	63	5.029372	3.38E+15	6.71E+14	########	2.03E+16	4.23E+16
sp|Q9Y5P6	6	63	68.90592	2.93E+14	4.25E+12	########	1.76E+15	2.67E+14
sp|Q9Y5S2	6	63	22.43521	1.03E+15	4.57E+13	########	6.15E+15	2.88E+15
sp|Q9Y5U8	6	63	16.25002	5.77E+14	3.55E+13	########	3.46E+15	2.24E+15
sp|Q9Y5X1	6	63	7.347949	1.37E+14	1.87E+13	########	8.23E+14	1.18E+15
sp|Q9Y5X3	6	63	3.065329	2.93E+13	9.57E+12	########	1.76E+14	6.03E+14
sp|Q9Y5Y2	6	63	1.734183	2.32E+13	1.34E+13	########	1.39E+14	8.42E+14
sp|Q9Y5Z4	6	63	128.6306	7.51E+14	5.84E+12	########	4.5E+15	3.68E+14
sp|Q9Y617	6	63	27.04654	3.25E+17	1.2E+16	########	2E+18	########
sp|Q9Y639	6	63	15.16775	4.06E+16	2.68E+15	########	2.44E+17	1.69E+17
sp|Q9Y653	6	63	########	3.43E+12	4.05E+12	########	2.06E+13	2.55E+14
sp|Q9Y678	6	63	50.50496	1.62E+15	3.2E+13	########	9.7E+15	2.02E+15

For Peer Review Only

sp|Q9Y696	6	63	1.409648	3.31E+15	2.35E+15	########	1.98E+16	1E+17
sp|Q9Y697	6	63	29.57651	8.52E+15	2.88E+14	########	5.11E+16	1.82E+16
sp|Q9Y6B6	6	63	8.645992	1.25E+15	1.45E+14	########	7.52E+15	9.14E+15
sp|Q9Y6C9	6	63	62.84563	5.37E+16	8.55E+14	########	3.22E+17	5.39E+16
sp|Q9Y6D5	6	63	7.533768	1.09E+14	1.44E+13	########	6.53E+14	9.1E+14
sp|Q9Y6D6	6	63	2.5968	8.02E+11	3.09E+11	########	4.81E+12	1.95E+13
sp|Q9Y6E0	6	63	3.323178	3.08E+13	9.26E+12	########	1.85E+14	5.83E+14
sp|Q9Y6G9	6	63	22.38877	2.23E+15	9.94E+13	########	1.34E+16	6.27E+15
sp|Q9Y6I3|	6	63	7.81021	3.28E+15	4.2E+14	########	1.97E+16	2.65E+16
sp|Q9Y6K8	6	63	13.98992	8.76E+15	6.26E+14	########	5.26E+16	4E+16
sp|Q9Y6M9	6	63	47.02851	2E+16	4.25E+14	########	1.2E+17	2.68E+16
sp|Q9Y6R1	6	63	35.53406	########	7.99E+15	########	1.7E+18	#########
sp|Q9Y6R7	6	63	2.377551	8.59E+13	3.61E+13	########	5.16E+14	2E+15
sp|Q9Y6T7	6	63	6.102717	7.78E+14	1.27E+14	########	4.67E+15	8.03E+15
sp|Q9Y6V0	6	63	28.55231	1.11E+15	3.9E+13	########	6.68E+15	2.45E+15
sp|Q9Y6X5	6	63	6.174018	5.08E+13	8.23E+12	########	3.05E+14	5.18E+14

For Review Only

padj	Significant	2-1	3-1	4-1	5-1	6-1	7-1	3-2
########	Yes	NA	NA	########	NA	NA	NA	NA
########	No	NA	NA	NA	NA	NA	NA	NA
########	No	NA	NA	NA	NA	NA	NA	NA
########	Yes	NA	NA	NA	NA	NA	########	NA
########	Yes	NA	NA	NA	NA	NA	########	NA
########	Yes	NA	NA	NA	NA	NA	NA	NA
########	Yes	NA	########	NA	NA	NA	NA	NA
########	No	NA	NA	NA	NA	NA	NA	NA
########	No	NA	NA	NA	NA	NA	NA	NA
########	Yes	NA	NA	NA	########	########	########	NA
########	No	NA	NA	NA	NA	NA	NA	NA
########	Yes	NA	NA	NA	NA	NA	########	NA
########	Yes	NA	NA	NA	NA	NA	########	NA
########	No	NA	NA	NA	NA	NA	NA	NA
########	Yes	NA	NA	NA	NA	NA	NA	NA
########	Yes	NA	NA	NA	NA	NA	NA	NA
########	No	NA	NA	NA	NA	NA	NA	NA
########	No	NA	NA	NA	NA	NA	NA	NA
########	Yes	NA	NA	NA	NA	NA	########	NA
########	No	NA	NA	NA	NA	NA	NA	NA
########	No	NA	NA	NA	NA	NA	NA	NA
########	Yes	########	########	########	########	########	########	NA
########	Yes	NA	NA	NA	NA	########	NA	NA
########	No	NA	NA	NA	NA	NA	NA	NA
########	No	NA	NA	NA	NA	NA	NA	NA
########	Yes	NA	NA	########	NA	NA	NA	NA
########	No	NA	NA	NA	NA	NA	NA	NA
########	No	NA	NA	NA	NA	NA	NA	NA
########	Yes	NA	NA	NA	NA	NA	########	NA
########	Yes	NA	NA	NA	NA	NA	########	NA
########	Yes	########	########	NA	########	########	########	NA
########	Yes	NA	NA	NA	NA	NA	########	NA
########	Yes	NA	NA	NA	########	NA	########	NA
########	Yes	NA	NA	NA	NA	NA	########	NA
########	No	NA	NA	NA	NA	NA	NA	NA
########	No	NA	NA	NA	NA	NA	NA	NA
########	Yes	NA	NA	NA	NA	NA	NA	NA
########	No	NA	NA	NA	NA	NA	NA	NA
########	Yes	NA	NA	NA	########	NA	########	NA
########	Yes	NA	NA	NA	NA	NA	NA	NA
########	Yes	NA	NA	NA	NA	NA	########	NA
########	Yes	NA	NA	NA	NA	NA	NA	NA
########	Yes	NA	NA	NA	NA	########	NA	NA
########	No	NA	NA	NA	NA	NA	NA	NA
########	Yes	NA	########	NA	########	########	########	NA
########	Yes	########	########	NA	NA	NA	NA	########
########	Yes	NA	NA	NA	NA	NA	########	NA
########	Yes	########	########	########	########	########	########	NA
########	Yes	NA	NA	NA	########	########	NA	NA
########	Yes	NA	NA	NA	NA	NA	NA	NA
########	Yes	########	########	########	########	########	########	NA
########	No	NA	NA	NA	NA	NA	NA	NA
########	Yes	NA	NA	NA	NA	NA	########	NA
########	No	NA	NA	NA	NA	NA	NA	NA
########	No	NA	NA	NA	NA	NA	NA	NA

For Review Only

########	Yes	########	########	NA	########	########	########	NA
########	Yes	NA	########	NA	NA	NA	########	NA
########	Yes	########	########	########	########	########	########	NA
########	Yes	NA	NA	NA	NA	NA	########	NA
########	Yes	NA	NA	NA	NA	########	########	NA
########	Yes	NA	NA	NA	NA	NA	NA	NA
########	Yes	NA	NA	NA	NA	NA	########	NA
########	Yes	NA	NA	NA	NA	########	NA	NA
########	Yes	NA	NA	NA	NA	NA	NA	NA
########	Yes	########	########	NA	########	NA	########	NA
########	No	NA	NA	NA	NA	NA	NA	NA
########	Yes	NA	NA	NA	NA	NA	########	NA
########	Yes	NA	NA	NA	NA	########	########	NA
########	Yes	NA	NA	NA	NA	NA	########	NA
########	No	NA	NA	NA	NA	NA	NA	NA
########	Yes	########	########	########	########	########	########	NA
########	No	NA	NA	NA	NA	NA	NA	NA
########	Yes	########	########	########	########	########	########	NA
########	Yes	NA	NA	NA	NA	NA	NA	NA
########	Yes	NA	NA	NA	NA	NA	NA	NA
########	Yes	NA	########	NA	########	########	########	NA
########	Yes	########	########	NA	NA	NA	########	NA
########	No	NA	NA	NA	NA	NA	NA	NA
########	Yes	########	########	########	########	########	########	NA
########	Yes	NA	NA	NA	NA	########	########	NA
########	Yes	NA	NA	NA	########	########	########	NA
########	Yes	NA	NA	NA	NA	########	NA	NA
########	Yes	########	########	########	########	########	########	NA
########	Yes	NA	NA	NA	NA	########	########	NA
########	Yes	NA	NA	NA	NA	NA	########	NA
########	Yes	NA	NA	NA	NA	NA	########	NA
########	Yes	########	########	########	########	########	########	NA
########	Yes	NA	NA	NA	NA	NA	NA	NA
########	Yes	########	########	########	########	########	########	NA
########	Yes	########	NA	NA	NA	########	########	NA
########	Yes	NA	NA	########	########	########	NA	NA
########	Yes	########	########	NA	NA	NA	NA	NA
########	Yes	NA	NA	NA	NA	########	########	NA
########	Yes	NA	NA	NA	NA	########	NA	NA
########	Yes	NA	NA	NA	NA	NA	########	NA
########	Yes	########	########	NA	########	########	########	NA
########	Yes	NA	NA	NA	NA	NA	NA	NA
########	Yes	########	########	NA	########	########	########	NA
########	Yes	########	########	########	########	########	########	NA
########	Yes	########	NA	########	########	########	########	NA
########	Yes	NA	NA	NA	NA	NA	NA	NA
########	Yes	NA	NA	NA	########	########	########	NA
########	Yes	NA	NA	NA	NA	NA	########	NA
########	Yes	########	########	########	########	########	########	NA
########	Yes	NA	NA	NA	NA	NA	NA	NA
########	No	NA	NA	NA	NA	NA	NA	NA
########	Yes	NA	NA	NA	########	########	NA	NA
########	Yes	########	########	########	NA	########	########	NA
########	Yes	NA	NA	NA	NA	########	########	NA
########	Yes	NA	NA	NA	NA	NA	########	NA
########	Yes	NA	NA	NA	NA	NA	NA	NA

For Review Only

######## Yes	NA	NA	NA	########	########	########	NA
######## Yes	NA	NA	NA	NA	NA	NA	NA
######## Yes	NA	NA	NA	NA	NA	########	NA
######## Yes	########	########	########	########	########	########	NA
######## Yes	NA	NA	NA	NA	########	NA	NA
######## Yes	NA	NA	NA	NA	NA	########	NA
######## Yes	NA	NA	NA	########	########	########	NA
######## Yes	NA	NA	NA	NA	########	########	NA
######## Yes	NA	NA	NA	########	########	NA	NA
######## No	NA	NA	NA	NA	NA	NA	NA
######## Yes	########	########	NA	NA	NA	NA	NA
######## Yes	NA	########	NA	########	########	########	NA
######## No	NA	NA	NA	NA	NA	NA	NA
######## Yes	NA	NA	NA	########	########	########	NA
######## Yes	########	########	NA	NA	NA	########	NA
######## Yes	########	########	NA	########	########	########	NA
######## Yes	########	########	########	########	########	########	NA
######## No	NA	NA	NA	NA	NA	NA	NA
######## Yes	NA	NA	NA	########	########	########	NA
######## Yes	NA	NA	NA	NA	########	NA	NA
######## Yes	########	########	########	########	########	########	NA
######## Yes	########	########	########	########	########	########	NA
######## Yes	NA	NA	NA	NA	########	########	NA
######## Yes	########	########	########	########	########	########	NA
######## Yes	NA	NA	NA	NA	NA	########	NA
######## Yes	NA	NA	NA	NA	NA	NA	NA
######## Yes	########	NA	NA	NA	NA	NA	NA
######## Yes	NA	NA	NA	NA	NA	NA	NA
######## Yes	NA	NA	NA	NA	########	########	NA
######## Yes	NA	NA	NA	NA	NA	########	NA
######## Yes	NA	NA	NA	NA	NA	NA	NA
######## Yes	NA	NA	NA	NA	########	NA	NA
######## Yes	########	########	########	########	########	########	NA
######## Yes	NA	NA	NA	########	########	########	NA
######## Yes	########	NA	########	########	########	########	########
######## Yes	NA	NA	########	########	########	NA	NA
######## Yes	NA	NA	NA	NA	NA	########	NA
######## Yes	NA	NA	NA	NA	NA	NA	NA
######## Yes	NA	NA	NA	########	NA	NA	NA
######## Yes	NA	NA	NA	NA	NA	########	NA
######## Yes	NA	NA	########	NA	########	########	NA
######## Yes	########	########	########	########	########	########	NA
######## No	NA	NA	NA	NA	NA	NA	NA
######## Yes	NA	NA	########	NA	NA	########	NA
######## Yes	NA	NA	########	########	########	########	NA
######## Yes	NA	NA	NA	NA	########	NA	NA
######## Yes	NA	NA	NA	NA	########	NA	NA
######## Yes	NA	########	########	########	########	########	NA
######## Yes	NA	NA	########	########	########	NA	NA
######## No	NA	NA	NA	NA	NA	NA	NA
######## Yes	NA	NA	NA	########	########	NA	NA
######## Yes	NA	NA	NA	########	NA	NA	NA
######## No	NA	NA	NA	NA	NA	NA	NA
######## No	NA	NA	NA	NA	NA	NA	NA

For Review Only

######## Yes	NA	NA	NA	NA	NA	########	NA
######## Yes	########	########	########	########	########	########	NA
######## No	NA	NA	NA	NA	NA	NA	NA
######## Yes	NA	NA	########	NA	NA	NA	NA
######## Yes	NA	NA	NA	NA	NA	NA	NA
######## Yes	NA	NA	NA	########	NA	NA	NA
######## Yes	########	########	NA	########	########	########	NA
######## Yes	NA	NA	########	########	########	########	NA
######## Yes	NA	NA	NA	########	########	########	NA
######## Yes	########	########	########	########	########	########	NA
######## Yes	NA	########	########	NA	NA	NA	########
######## Yes	NA	NA	NA	########	########	########	NA
######## Yes	########	########	########	########	########	########	NA
######## Yes	NA	NA	########	########	########	NA	NA
######## Yes	########	########	########	########	########	########	NA
######## Yes	NA	NA	NA	NA	NA	########	NA
######## Yes	NA	NA	NA	NA	########	NA	NA
######## Yes	NA	NA	NA	NA	NA	########	NA
######## Yes	NA	NA	NA	NA	########	########	NA
######## Yes	NA	NA	NA	########	NA	########	NA
######## Yes	########	NA	NA	NA	NA	########	NA
######## Yes	########	NA	NA	NA	NA	########	NA
######## Yes	NA	NA	NA	########	########	########	NA
######## Yes	NA	NA	NA	NA	NA	########	NA
######## Yes	NA	NA	########	########	########	NA	NA
######## No	NA	NA	NA	NA	NA	NA	NA
######## No	NA	NA	NA	NA	NA	NA	NA
######## Yes	NA	NA	NA	NA	NA	########	NA
######## Yes	NA	NA	NA	NA	########	NA	NA
######## Yes	NA	NA	NA	NA	NA	########	NA
######## Yes	########	########	NA	########	########	########	########
######## Yes	########	########	NA	NA	########	########	NA
######## Yes	########	########	NA	NA	########	########	NA
######## Yes	########	########	NA	########	########	########	NA
######## Yes	NA	NA	NA	NA	########	########	NA
######## No	NA	NA	NA	NA	NA	NA	NA
######## Yes	NA	NA	########	########	########	########	NA
######## Yes	NA	NA	NA	NA	NA	########	NA
######## No	NA	NA	NA	NA	NA	NA	NA
######## Yes	NA	NA	NA	NA	NA	########	NA
######## No	NA	NA	NA	NA	NA	NA	NA
######## Yes	NA	NA	NA	NA	########	NA	NA
######## No	NA	NA	NA	NA	NA	NA	NA
######## Yes	NA	NA	NA	NA	########	NA	NA
######## No	NA	NA	NA	NA	NA	NA	NA
######## Yes	NA	NA	NA	NA	NA	########	NA
######## Yes	NA	########	NA	NA	NA	NA	########
######## Yes	NA	NA	NA	NA	NA	########	NA
######## Yes	########	########	########	NA	NA	########	NA
######## Yes	NA	NA	NA	NA	########	NA	NA
######## Yes	########	########	NA	########	########	########	NA
######## Yes	NA	NA	NA	NA	NA	NA	NA
######## No	NA	NA	NA	NA	NA	NA	NA
######## Yes	NA	NA	NA	########	########	########	NA
######## Yes	########	########	NA	########	########	########	NA
######## Yes	########	########	NA	########	########	NA	NA

For Review Only

######## Yes	########	NA	NA	NA	NA	NA	NA
######## No	NA	NA	NA	NA	NA	NA	NA
######## Yes	NA	NA	NA	NA	########	########	NA
######## Yes	NA	NA	NA	NA	########	########	NA
######## Yes	NA	NA	NA	NA	NA	########	NA
######## Yes	NA	NA	NA	NA	NA	NA	NA
######## Yes	NA	########	NA	NA	########	########	NA
######## Yes	NA	NA	NA	NA	NA	NA	NA
######## Yes	########	########	NA	########	########	########	NA
######## Yes	NA	NA	NA	NA	########	########	NA
######## Yes	NA	NA	NA	NA	NA	########	NA
######## Yes	NA	NA	NA	NA	NA	NA	NA
######## Yes	NA	NA	NA	NA	########	########	NA
######## Yes	NA	NA	NA	NA	NA	########	NA
######## Yes	NA	NA	NA	########	########	########	NA
######## Yes	########	########	########	########	NA	NA	NA
######## Yes	NA	NA	NA	########	NA	NA	NA
######## Yes	########	NA	NA	########	########	########	NA
######## Yes	NA	NA	NA	NA	########	########	NA
######## No	NA	NA	NA	NA	NA	NA	NA
######## Yes	NA	NA	NA	NA	NA	########	NA
######## Yes	########	########	########	########	########	########	NA
######## Yes	NA	NA	NA	NA	########	########	NA
######## Yes	NA	NA	NA	NA	NA	########	NA
######## Yes	NA	NA	NA	NA	NA	NA	NA
######## No	NA	NA	NA	NA	NA	NA	NA
######## Yes	NA	NA	NA	NA	########	########	NA
######## Yes	NA	NA	NA	NA	########	########	NA
######## Yes	NA	NA	NA	NA	########	NA	NA
######## Yes	NA	NA	NA	########	NA	########	NA
######## Yes	NA	NA	NA	########	NA	########	NA
######## No	NA	NA	NA	NA	NA	NA	NA
######## Yes	NA	NA	NA	NA	NA	########	NA
######## Yes	NA	NA	NA	NA	########	NA	NA
######## Yes	NA	NA	########	########	########	NA	NA
######## Yes	########	########	########	########	########	########	NA
######## Yes	NA	NA	NA	########	########	########	NA
######## Yes	NA	NA	NA	NA	########	########	NA
######## Yes	NA	NA	NA	NA	NA	########	NA
######## Yes	NA	NA	NA	########	NA	NA	NA
######## Yes	NA	NA	NA	NA	########	NA	NA
######## Yes	NA	NA	NA	NA	NA	NA	NA
######## Yes	NA	NA	NA	NA	NA	NA	NA
######## Yes	NA	NA	NA	NA	########	########	NA
######## Yes	NA	NA	NA	NA	NA	########	NA
######## Yes	NA	NA	NA	NA	NA	########	NA
######## Yes	NA	NA	NA	NA	NA	NA	NA
######## Yes	NA	NA	########	########	NA	########	NA
######## No	NA	NA	NA	NA	NA	NA	NA
######## Yes	########	########	########	########	########	########	NA
######## Yes	NA	NA	########	########	########	########	NA
######## Yes	NA	NA	NA	########	########	########	NA
######## Yes	NA	NA	NA	NA	NA	########	NA
######## Yes	NA	########	########	########	########	########	NA
######## No	NA	NA	NA	NA	NA	NA	NA
######## Yes	NA	########	NA	NA	########	NA	NA

For Review Only

########	Yes	NA	NA	NA	NA	NA	######## NA
########	Yes	########	########	NA	########	########	######## NA
########	Yes	########	########	NA	########	########	######## NA
########	Yes	########	########	NA	NA	NA	######## NA
########	Yes	NA	NA	NA	NA	NA	NA NA
########	Yes	NA	NA	NA	########	########	######## NA
########	Yes	NA	NA	NA	########	NA	######## NA
########	No	NA	NA	NA	NA	NA	NA NA
########	Yes	NA	NA	NA	NA	NA	######## NA
########	Yes	NA	NA	NA	NA	NA	######## NA
########	Yes	NA	NA	NA	NA	########	######## NA
########	Yes	########	########	########	########	########	######## NA
########	Yes	NA	NA	NA	########	NA	######## NA
########	Yes	########	########	NA	########	########	NA NA
########	Yes	NA	NA	NA	NA	NA	######## NA
########	Yes	NA	NA	########	NA	########	######## NA
########	Yes	NA	########	NA	########	########	######## NA
########	Yes	########	########	NA	########	########	NA NA
########	Yes	NA	NA	NA	NA	########	######## NA
########	Yes	########	########	NA	########	########	######## NA
########	Yes	NA	NA	NA	NA	NA	NA NA
########	Yes	NA	########	NA	########	########	######## NA
########	Yes	########	########	NA	NA	NA	######## NA
########	Yes	########	########	########	########	########	######## NA
########	Yes	########	########	NA	########	########	######## NA
########	Yes	########	NA	NA	########	########	######## NA
########	Yes	NA	NA	NA	########	########	######## NA
########	No	NA	NA	NA	NA	NA	NA NA
########	Yes	########	########	########	########	########	######## NA
########	Yes	########	########	########	########	########	######## NA
########	Yes	########	NA	NA	NA	########	######## NA
########	Yes	NA	NA	NA	NA	NA	######## NA
########	Yes	########	########	########	########	########	######## NA
########	Yes	NA	NA	NA	NA	NA	######## NA
########	Yes	########	########	########	########	########	######## NA
########	Yes	NA	NA	NA	NA	NA	######## NA
########	Yes	NA	NA	NA	########	########	######## NA
########	Yes	########	########	NA	########	########	######## NA
########	Yes	NA	NA	NA	NA	########	######## NA
########	Yes	NA	NA	NA	NA	########	NA NA
########	Yes	NA	NA	NA	NA	########	NA NA
########	Yes	NA	NA	NA	NA	NA	######## NA
########	Yes	NA	NA	NA	NA	NA	NA NA
########	Yes	NA	NA	NA	NA	NA	######## NA
########	Yes	NA	NA	NA	########	NA	######## NA
########	Yes	NA	########	NA	NA	NA	NA NA
########	Yes	########	########	NA	NA	NA	NA NA
########	Yes	NA	NA	NA	NA	NA	######## NA
########	Yes	NA	NA	NA	########	########	######## NA
########	No	NA	NA	NA	NA	NA	NA NA
########	Yes	NA	NA	NA	NA	NA	NA NA
########	Yes	NA	NA	NA	NA	NA	######## NA
########	Yes	NA	NA	NA	NA	NA	######## NA
########	No	NA	NA	NA	NA	NA	NA NA
########	Yes	NA	NA	NA	########	########	######## NA

For Review Only

######## No	NA	NA	NA	NA	NA	NA	NA
######## Yes	########	########	########	########	########	########	NA
######## Yes	########	########	NA	########	########	########	NA
######## Yes	NA	NA	NA	########	NA	########	NA
######## Yes	NA	NA	########	########	########	########	########
######## Yes	########	########	NA	########	########	########	NA
######## Yes	NA	NA	NA	NA	NA	########	NA
######## Yes	NA	NA	########	########	NA	NA	NA
######## Yes	NA	NA	NA	NA	########	NA	NA
######## No	NA	NA	NA	NA	NA	NA	NA
######## Yes	NA	NA	NA	NA	NA	########	NA
######## Yes	NA	NA	########	NA	NA	########	NA
######## Yes	NA	NA	########	########	########	########	NA
######## Yes	NA	NA	NA	NA	########	NA	NA
######## No	NA	NA	NA	NA	NA	NA	NA
######## Yes	########	########	########	NA	NA	########	NA
######## Yes	NA	########	NA	NA	NA	########	NA
######## Yes	########	########	NA	########	########	########	NA
######## Yes	NA	NA	NA	########	########	########	NA
######## Yes	NA	NA	NA	NA	########	########	NA
######## Yes	NA	NA	NA	########	########	########	NA
######## Yes	NA	NA	NA	NA	NA	########	NA
######## Yes	NA	NA	########	########	########	########	NA
######## Yes	NA	NA	NA	NA	NA	########	NA
######## Yes	########	########	NA	NA	NA	########	NA
######## Yes	NA	NA	NA	NA	NA	########	NA
######## No	NA	NA	NA	NA	NA	NA	NA
######## Yes	NA	NA	NA	NA	########	########	NA
######## Yes	NA	NA	########	########	########	########	NA
######## Yes	########	########	NA	########	########	########	NA
######## No	NA	NA	NA	NA	NA	NA	NA
######## Yes	NA	NA	########	########	########	########	NA
######## Yes	NA	NA	NA	NA	########	########	NA
######## Yes	NA	NA	NA	NA	########	########	NA
######## Yes	NA	NA	NA	NA	########	########	NA
######## Yes	NA	########	NA	########	########	########	NA
######## Yes	########	########	NA	NA	########	########	NA
######## Yes	########	########	NA	NA	NA	NA	NA
######## Yes	########	########	NA	########	########	########	NA
######## Yes	NA	NA	NA	NA	NA	NA	NA
######## Yes	########	########	NA	########	########	########	NA
######## Yes	NA	NA	NA	NA	########	NA	NA
######## Yes	NA	NA	NA	NA	########	########	NA
######## Yes	########	########	NA	########	########	########	NA
######## Yes	NA	NA	NA	########	########	########	NA
######## Yes	NA	NA	NA	########	########	########	NA
######## Yes	NA	NA	NA	NA	########	########	NA
######## No	NA	NA	NA	NA	NA	NA	NA
######## Yes	NA	NA	NA	NA	NA	NA	NA
######## No	NA	NA	NA	NA	NA	NA	NA
######## Yes	NA	########	NA	NA	########	########	NA
######## Yes	########	########	NA	########	########	########	NA
######## No	NA	NA	NA	NA	NA	NA	NA
######## Yes	NA	NA	NA	########	########	########	NA
######## Yes	NA	NA	NA	NA	NA	########	NA

For Review Only

######## Yes	NA	NA	NA	NA	########	########	NA
######## Yes	NA	NA	NA	NA	########	NA	NA
######## Yes	NA	NA	NA	NA	NA	########	NA
######## Yes	########	########	NA	########	########	########	NA
######## Yes	NA	NA	NA	NA	########	########	NA
######## No	NA	NA	NA	NA	NA	NA	NA
######## Yes	########	########	########	NA	NA	NA	NA
######## Yes	NA	########	NA	NA	NA	NA	NA
######## Yes	NA	NA	NA	NA	NA	########	NA
######## Yes	NA	NA	NA	NA	########	NA	NA
######## No	NA	NA	NA	NA	NA	NA	NA
######## Yes	NA	NA	########	NA	########	########	NA
######## No	NA	NA	NA	NA	NA	NA	NA
######## Yes	NA	NA	NA	NA	NA	NA	NA
######## Yes	NA	NA	NA	NA	########	########	NA
######## Yes	NA	NA	NA	NA	NA	########	NA
######## Yes	NA	NA	NA	NA	NA	########	NA
######## Yes	NA	NA	NA	NA	########	########	NA
######## Yes	########	########	########	########	########	########	NA
######## Yes	NA	NA	NA	NA	NA	########	NA
######## Yes	NA	NA	NA	NA	NA	########	NA
######## Yes	NA	NA	NA	NA	NA	########	NA
######## Yes	########	########	NA	########	########	########	NA
######## Yes	NA	NA	NA	########	NA	NA	NA
######## Yes	########	########	NA	########	########	########	NA
######## Yes	NA	NA	NA	NA	########	NA	NA
######## Yes	NA	NA	NA	NA	NA	########	NA
######## Yes	NA	NA	NA	NA	NA	########	NA
######## Yes	NA	NA	NA	########	########	NA	NA
######## Yes	########	NA	NA	NA	NA	NA	NA
######## Yes	NA	NA	NA	NA	NA	########	NA
######## Yes	NA	NA	NA	NA	NA	########	NA
######## Yes	########	########	NA	########	########	########	NA
######## Yes	NA	NA	########	NA	NA	NA	NA
######## Yes	########	########	NA	NA	NA	NA	NA
######## Yes	NA	NA	########	########	########	########	NA
######## Yes	NA	NA	########	########	########	########	NA
######## Yes	NA	########	NA	########	########	NA	NA
######## Yes	NA	NA	########	########	########	NA	NA
######## Yes	NA	########	NA	NA	NA	########	NA
######## Yes	NA	NA	NA	NA	NA	########	NA
######## Yes	########	########	NA	########	########	########	NA
######## Yes	NA	NA	NA	########	########	########	NA
######## Yes	NA	NA	NA	NA	NA	########	NA
######## Yes	NA	NA	NA	NA	NA	########	NA
######## Yes	NA	NA	NA	NA	NA	########	NA
######## Yes	NA	NA	NA	########	########	########	NA
######## Yes	########	########	NA	########	########	########	NA
######## Yes	NA	NA	########	NA	NA	NA	NA
######## Yes	NA	NA	########	########	########	########	NA
######## No	NA	NA	NA	NA	NA	NA	NA
######## Yes	NA	NA	NA	NA	NA	########	NA
######## Yes	NA	NA	NA	NA	NA	########	NA
######## Yes	NA	NA	NA	NA	NA	########	NA
######## No	NA	NA	NA	NA	NA	NA	NA
######## Yes	NA	NA	NA	NA	NA	########	NA

For Review Only

######## Yes	NA	NA	NA	########	########	########	NA
######## Yes	NA	NA	NA	NA	NA	########	NA
######## Yes	NA	NA	NA	NA	NA	########	NA
######## Yes	NA	NA	NA	NA	NA	########	NA
######## Yes	NA	NA	NA	NA	NA	########	NA
######## Yes	NA	NA	NA	NA	NA	########	NA
######## Yes	NA	NA	NA	NA	NA	########	NA
######## Yes	NA	NA	NA	NA	NA	########	NA
######## Yes	NA	NA	NA	NA	NA	########	NA
######## Yes	NA	NA	########	########	########	########	NA
######## Yes	NA	NA	########	########	########	NA	NA
######## No	NA	NA	NA	NA	NA	NA	NA
######## Yes	NA	NA	NA	NA	NA	NA	NA
######## Yes	NA	NA	NA	NA	NA	########	NA
######## Yes	NA	NA	NA	NA	NA	########	NA
######## Yes	NA	NA	NA	NA	NA	########	NA
######## Yes	NA	NA	NA	NA	########	########	NA
######## Yes	NA	NA	NA	NA	NA	########	NA
######## Yes	NA	NA	NA	NA	########	NA	NA
######## Yes	NA	NA	NA	NA	NA	########	NA
######## Yes	NA	NA	NA	NA	########	NA	NA
######## Yes	NA	NA	NA	NA	NA	########	NA
######## Yes	########	########	########	########	########	########	NA
######## Yes	NA	NA	NA	NA	NA	########	NA
######## Yes	########	########	########	########	########	########	NA
######## Yes	NA	########	NA	NA	NA	NA	########
######## Yes	NA	NA	NA	NA	NA	########	NA
######## Yes	NA	NA	NA	NA	########	NA	NA
######## Yes	NA	NA	NA	NA	NA	########	NA
######## Yes	NA	NA	NA	NA	########	NA	NA
######## Yes	NA	NA	NA	NA	NA	NA	NA
######## Yes	NA	NA	NA	NA	NA	########	NA
######## Yes	NA	NA	NA	NA	NA	########	NA
######## Yes	NA	########	########	########	########	########	NA
######## Yes	NA	NA	########	NA	NA	########	NA
######## Yes	NA	NA	NA	NA	NA	NA	NA
######## Yes	NA	NA	NA	NA	########	NA	NA
######## Yes	NA	NA	NA	NA	########	NA	NA
######## Yes	NA	NA	NA	NA	########	NA	NA
######## No	NA	NA	NA	NA	NA	NA	NA
######## Yes	NA	NA	NA	NA	NA	########	NA
######## Yes	NA	NA	NA	NA	NA	########	NA
######## Yes	NA	NA	NA	NA	NA	########	NA
######## Yes	NA	NA	NA	NA	NA	########	NA
######## Yes	NA	NA	NA	NA	NA	########	NA
######## Yes	NA	NA	NA	NA	NA	########	NA
######## Yes	NA	NA	NA	########	########	NA	NA
######## Yes	NA	NA	NA	NA	NA	########	NA
######## Yes	NA	NA	NA	NA	NA	########	NA
######## Yes	NA	NA	NA	NA	########	NA	NA
######## Yes	NA	NA	NA	NA	########	NA	NA
######## Yes	########	########	NA	NA	NA	NA	NA
######## Yes	########	########	########	NA	NA	NA	NA
######## Yes	########	########	########	NA	NA	NA	NA
######## Yes	########	########	NA	########	########	########	NA
######## Yes	NA	########	NA	NA	NA	NA	NA

For Review Only

######## Yes	NA	NA	NA	NA	########	NA	NA
######## Yes	NA	NA	NA	NA	NA	########	NA
######## Yes	NA	NA	########	########	########	NA	NA
######## Yes	NA	NA	NA	########	########	########	NA
######## Yes	NA	NA	NA	NA	NA	########	NA
######## No	NA	NA	NA	NA	NA	NA	NA
######## Yes	NA	NA	NA	NA	NA	########	NA
######## Yes	NA	NA	NA	NA	NA	########	NA
######## Yes	########	########	NA	NA	NA	########	NA
######## Yes	########	########	NA	NA	########	NA	NA
######## Yes	NA	NA	NA	NA	NA	########	NA
######## Yes	NA	NA	NA	NA	NA	########	NA
######## Yes	NA	NA	NA	NA	NA	NA	NA
######## Yes	NA	NA	NA	NA	NA	########	NA
######## Yes	########	########	########	NA	########	########	NA
######## Yes	NA	NA	########	########	########	########	NA
######## Yes	NA	NA	NA	NA	NA	########	NA
######## Yes	NA	NA	NA	NA	########	########	NA
######## Yes	NA	NA	NA	########	########	NA	NA
######## Yes	########	NA	########	########	NA	########	NA
######## Yes	########	NA	########	########	NA	########	########
######## Yes	NA	NA	########	########	########	########	NA
######## Yes	NA	NA	NA	########	########	########	NA
######## Yes	NA	NA	NA	########	########	########	NA
######## Yes	########	NA	NA	NA	NA	NA	NA
######## No	NA	NA	NA	NA	NA	NA	NA
######## Yes	NA	NA	########	########	########	NA	NA
######## Yes	########	########	NA	NA	NA	########	NA
######## Yes	NA	NA	NA	########	########	NA	NA
######## No	NA	NA	NA	NA	NA	NA	NA
######## Yes	NA	NA	NA	NA	########	########	NA
######## Yes	NA	########	NA	########	########	########	NA
######## Yes	NA	NA	NA	NA	NA	########	NA
######## No	NA	NA	NA	NA	NA	NA	NA
######## Yes	NA	NA	NA	NA	########	NA	NA
######## Yes	NA	NA	NA	NA	########	NA	NA
######## Yes	NA	NA	NA	NA	########	NA	NA
######## Yes	NA	NA	NA	########	NA	########	NA
######## No	NA	NA	NA	NA	NA	NA	NA
######## Yes	########	########	########	########	########	NA	NA
######## Yes	NA	NA	NA	########	########	NA	NA
######## Yes	NA	########	########	########	########	########	NA
######## Yes	NA	NA	NA	NA	NA	########	NA
######## Yes	NA	NA	NA	NA	NA	########	NA
######## Yes	NA	NA	NA	NA	########	########	NA
######## Yes	NA	NA	NA	NA	NA	NA	NA
######## No	NA	NA	NA	NA	NA	NA	NA
######## Yes	########	########	NA	########	########	########	NA
######## Yes	NA	NA	NA	NA	NA	########	NA
######## Yes	NA	NA	NA	NA	NA	########	NA
######## No	NA	NA	NA	NA	NA	NA	NA

For Review Only

######## Yes	NA	NA	NA	########	########	########	NA
######## Yes	NA	NA	NA	NA	NA	########	NA
######## Yes	########	########	########	########	########	########	NA
######## Yes	NA	NA	NA	NA	NA	########	NA
######## Yes	NA	NA	########	########	########	########	NA
######## No	NA	NA	NA	NA	NA	NA	NA
######## Yes	NA	NA	########	########	########	########	NA
######## Yes	NA	NA	NA	NA	NA	########	NA
######## Yes	NA	NA	########	########	NA	########	NA
######## Yes	########	########	########	########	########	########	NA
######## Yes	########	########	########	########	########	########	NA
######## Yes	########	########	NA	NA	NA	########	NA
######## Yes	NA	NA	########	########	NA	NA	NA
######## Yes	########	########	########	########	########	########	NA
######## Yes	NA	NA	NA	NA	NA	########	NA
######## Yes	NA	NA	NA	NA	NA	########	NA
######## Yes	NA	NA	NA	########	########	########	NA
######## Yes	NA	NA	NA	NA	NA	########	NA
######## Yes	########	########	########	########	########	########	NA
######## Yes	NA	########	########	########	NA	NA	########
######## Yes	NA	NA	NA	NA	NA	########	NA
######## Yes	NA	NA	NA	NA	NA	########	NA
######## Yes	NA	NA	NA	NA	NA	########	NA
######## Yes	NA	NA	NA	NA	NA	########	NA
######## Yes	NA	NA	########	########	########	########	NA
######## Yes	NA	NA	NA	NA	NA	########	NA
######## No	NA	NA	NA	NA	NA	NA	NA
######## Yes	########	########	########	########	########	########	NA
######## Yes	########	########	NA	########	########	########	NA
######## Yes	NA	NA	NA	NA	NA	NA	NA
######## Yes	########	########	NA	########	########	########	NA
######## No	NA	NA	NA	NA	NA	NA	NA
######## Yes	NA	NA	########	########	########	########	NA
######## No	NA	NA	NA	NA	NA	NA	NA
######## Yes	NA	NA	NA	NA	NA	NA	NA
######## Yes	NA	NA	########	########	########	########	NA
######## Yes	NA	NA	NA	########	########	########	NA
######## Yes	NA	NA	NA	NA	########	########	NA
######## No	NA	NA	NA	NA	NA	NA	NA
######## No	NA	NA	NA	NA	NA	NA	NA
######## Yes	NA	NA	NA	NA	########	NA	NA
######## No	NA	NA	NA	NA	NA	NA	NA
######## No	NA	NA	NA	NA	NA	NA	NA
######## Yes	########	########	NA	########	NA	########	NA
######## No	NA	NA	NA	NA	NA	NA	NA
######## Yes	########	########	########	########	########	########	NA
######## Yes	########	########	NA	########	########	########	########
######## Yes	NA	NA	NA	NA	NA	NA	NA
######## Yes	NA	NA	NA	NA	########	NA	NA
######## Yes	NA	NA	NA	NA	########	NA	NA
######## Yes	########	########	########	########	NA	NA	NA
######## Yes	NA	NA	NA	NA	########	NA	NA
######## Yes	NA	NA	########	########	########	########	NA
######## Yes	NA	NA	NA	########	########	########	NA
######## Yes	NA	NA	NA	NA	########	NA	NA
######## Yes	NA	NA	NA	NA	########	NA	NA

For Review Only

########	Yes	NA	NA	NA	########	########	########	NA
########	Yes	NA	########	########	NA	########	########	NA
########	Yes	NA	NA	NA	########	########	########	NA
########	Yes	NA	NA	NA	########	########	########	NA
########	Yes	NA	NA	NA	NA	########	########	NA
########	Yes	########	########	NA	NA	NA	########	NA
########	Yes	NA	NA	NA	NA	NA	########	NA
########	Yes	NA	NA	########	########	########	########	NA
########	Yes	NA	NA	NA	NA	NA	########	NA
########	Yes	NA	NA	NA	NA	NA	########	NA
########	Yes	NA	NA	NA	########	########	NA	NA
########	Yes	NA	NA	NA	NA	########	NA	NA
########	Yes	NA	NA	NA	########	########	########	NA
########	Yes	NA	NA	NA	########	########	########	NA
########	Yes	########	########	NA	########	NA	########	NA
########	Yes	########	########	########	########	########	########	NA
########	Yes	NA	########	NA	NA	########	########	NA
########	Yes	########	########	########	########	########	########	NA
########	Yes	NA	NA	NA	NA	NA	########	NA
########	Yes	NA	NA	NA	NA	NA	########	NA
########	Yes	NA	NA	NA	########	########	########	NA
########	No	NA	NA	NA	NA	NA	NA	NA
########	No	NA	NA	NA	NA	NA	NA	NA
########	Yes	NA	NA	NA	NA	NA	NA	NA
########	Yes	NA	NA	NA	NA	NA	########	NA
########	Yes	NA	NA	NA	NA	########	NA	NA
########	Yes	NA	NA	NA	NA	NA	########	NA
########	Yes	NA	NA	NA	NA	NA	########	NA
########	Yes	NA	NA	NA	NA	NA	########	NA
########	Yes	########	########	NA	########	########	########	NA
########	Yes	NA	NA	########	NA	NA	########	NA
########	Yes	NA	NA	NA	NA	NA	########	NA
########	Yes	NA	NA	NA	NA	NA	########	NA
########	Yes	NA	NA	NA	NA	########	########	NA
########	Yes	########	########	########	########	NA	########	NA
########	No	NA	NA	NA	NA	NA	NA	NA
########	Yes	########	########	NA	########	NA	########	NA
########	Yes	NA	NA	NA	NA	NA	########	NA
########	Yes	########	########	NA	########	########	########	NA
########	Yes	NA	NA	NA	NA	########	NA	NA
########	Yes	NA	NA	NA	NA	NA	########	NA
########	Yes	NA	NA	NA	NA	NA	########	NA
########	Yes	NA	NA	NA	########	########	########	NA
########	Yes	########	########	NA	########	########	NA	NA
########	Yes	NA	NA	NA	NA	########	NA	NA
########	Yes	NA	NA	NA	NA	########	NA	NA
########	Yes	NA	NA	########	########	NA	NA	NA
########	Yes	########	########	########	########	########	########	NA
########	Yes	NA	NA	NA	NA	NA	NA	NA
########	Yes	NA	NA	NA	NA	NA	########	NA
########	Yes	########	########	NA	########	########	########	NA
########	Yes	NA	NA	########	########	########	NA	NA
########	Yes	########	########	NA	########	NA	########	NA
########	Yes	########	########	NA	########	########	########	NA
########	Yes	NA	NA	########	########	########	NA	NA

For Review Only

######## Yes	NA	NA	NA	NA	NA	########	NA
######## Yes	NA	NA	NA	NA	NA	NA	NA
######## Yes	NA	NA	NA	########	########	########	NA
######## Yes	NA	NA	NA	NA	########	NA	NA
######## Yes	NA	NA	########	########	NA	########	NA
######## No	NA	NA	NA	NA	NA	NA	NA
######## Yes	NA	NA	########	########	########	########	NA
######## Yes	NA	NA	NA	NA	NA	########	NA
######## Yes	NA	NA	########	########	########	########	NA
######## Yes	########	########	########	########	########	########	########
######## Yes	NA	NA	NA	NA	NA	########	NA
######## Yes	NA	NA	NA	NA	NA	########	NA
######## Yes	NA	NA	########	########	########	########	NA
######## Yes	NA	NA	NA	NA	########	########	NA
######## Yes	########	########	########	########	########	########	NA
######## Yes	########	########	NA	NA	########	########	NA
######## Yes	NA	NA	NA	NA	NA	NA	NA
######## Yes	########	########	NA	NA	NA	########	NA
######## Yes	########	NA	NA	NA	NA	########	NA
######## Yes	########	########	########	########	########	########	NA
######## Yes	########	########	NA	########	########	########	NA
######## Yes	########	########	NA	########	########	########	NA
######## Yes	NA	NA	NA	NA	NA	NA	NA
######## Yes	########	########	########	########	########	NA	NA
######## Yes	NA	########	NA	NA	########	########	NA
######## Yes	NA	NA	NA	NA	NA	########	NA
######## Yes	NA	NA	NA	NA	NA	########	NA
######## No	NA	NA	NA	NA	NA	NA	NA
######## Yes	NA	NA	########	########	########	########	NA
######## Yes	NA	NA	NA	NA	########	########	NA
######## Yes	NA	NA	NA	NA	NA	########	NA
######## Yes	########	########	########	########	########	########	NA
######## Yes	########	########	########	########	########	########	NA
######## Yes	########	########	NA	NA	########	########	NA
######## Yes	NA	NA	########	########	########	NA	NA
######## Yes	NA	NA	NA	NA	NA	NA	NA
######## Yes	########	NA	NA	NA	NA	NA	NA
######## Yes	NA	NA	########	########	########	########	NA
######## Yes	NA	NA	NA	NA	########	NA	NA
######## Yes	NA	NA	NA	########	########	########	NA
######## Yes	NA	NA	NA	NA	########	NA	NA
######## Yes	NA	NA	########	########	NA	NA	NA
######## Yes	NA	NA	NA	NA	NA	########	NA
######## Yes	NA	NA	NA	NA	NA	########	NA
######## Yes	NA	NA	NA	NA	########	NA	NA
######## Yes	NA	NA	NA	NA	NA	########	NA
######## Yes	NA	NA	NA	NA	NA	########	NA
######## Yes	########	########	NA	NA	NA	########	NA
######## Yes	NA	NA	NA	NA	########	NA	NA
######## Yes	NA	NA	NA	########	NA	########	NA
######## Yes	NA	NA	NA	NA	NA	########	NA
######## Yes	NA	NA	NA	########	########	NA	NA
######## Yes	NA	NA	NA	NA	NA	########	NA
######## Yes	NA	NA	NA	NA	NA	########	NA
######## Yes	NA	NA	########	########	########	NA	NA

For Review Only

######## Yes	NA	NA	########	NA	NA	NA	NA
######## Yes	NA	NA	NA	NA	NA	########	NA
######## Yes	NA	NA	NA	NA	NA	NA	NA
######## Yes	########	########	NA	########	########	########	NA
######## Yes	NA	NA	########	########	########	NA	NA
######## Yes	########	########	########	########	########	########	NA
######## No	NA	NA	NA	NA	NA	NA	NA
######## Yes	NA	NA	########	########	########	########	NA
######## Yes	NA	NA	NA	NA	########	########	NA
######## Yes	NA	NA	########	########	NA	########	NA
######## Yes	NA	NA	NA	NA	NA	########	NA
######## Yes	NA	########	NA	########	########	########	NA
######## Yes	########	########	########	########	########	########	NA
######## Yes	NA	NA	NA	NA	NA	NA	NA
######## Yes	NA	NA	########	########	########	########	NA
######## Yes	NA	NA	NA	NA	NA	NA	NA
######## Yes	########	########	NA	########	########	########	NA
######## No	NA	NA	NA	NA	NA	NA	NA
######## Yes	NA	NA	NA	NA	########	########	NA
######## Yes	NA	NA	NA	########	########	########	NA
######## No	NA	NA	NA	NA	NA	NA	NA
######## Yes	NA	NA	NA	NA	NA	########	NA
######## Yes	NA	NA	NA	########	########	########	NA
######## No	NA	NA	NA	NA	NA	NA	NA
######## Yes	########	########	NA	NA	NA	########	NA
######## Yes	NA	NA	NA	NA	NA	########	NA
######## Yes	NA	NA	NA	NA	########	NA	NA
######## Yes	NA	NA	NA	NA	########	########	NA
######## No	NA	NA	NA	NA	NA	NA	NA
######## Yes	NA	########	NA	NA	########	########	NA
######## Yes	NA	NA	NA	NA	########	NA	NA
######## Yes	NA	NA	NA	NA	NA	########	NA
######## Yes	########	########	########	########	########	########	NA
######## Yes	NA	NA	NA	NA	########	NA	NA
######## Yes	NA	NA	NA	########	########	NA	NA
######## No	NA	NA	NA	NA	NA	NA	NA
######## Yes	NA	NA	NA	NA	NA	########	NA
######## Yes	NA	NA	NA	NA	########	########	NA
######## Yes	NA	NA	NA	NA	########	########	NA
######## No	NA	NA	NA	NA	NA	NA	NA
######## Yes	NA	NA	NA	NA	NA	########	NA
######## Yes	NA	NA	NA	########	########	########	NA
######## Yes	NA	NA	NA	NA	NA	########	NA
######## Yes	########	########	########	########	########	########	NA
######## Yes	NA	NA	NA	NA	NA	NA	NA
######## Yes	NA	NA	########	########	########	########	NA
######## Yes	NA	NA	########	########	NA	########	NA
######## No	NA	NA	NA	NA	NA	NA	NA
######## Yes	NA	NA	########	########	########	NA	NA
######## No	NA	NA	NA	NA	NA	NA	NA
######## Yes	NA	NA	NA	NA	NA	NA	NA
######## Yes	NA	NA	NA	########	########	########	NA

For Review Only

########	Yes	NA	NA	NA	########	NA	########	NA
########	Yes	NA	NA	########	NA	NA	########	NA
########	Yes	########	########	########	########	########	########	NA
########	Yes	########	########	########	########	########	########	NA
########	Yes	NA	NA	NA	NA	NA	########	NA
########	Yes	NA	NA	NA	########	########	########	NA
########	Yes	NA	NA	NA	########	########	########	NA
########	Yes	########	########	NA	NA	NA	########	NA
########	Yes	########	########	NA	NA	########	########	NA
########	Yes	NA	NA	NA	NA	########	NA	NA
########	Yes	NA	NA	NA	########	########	########	NA
########	Yes	NA	NA	NA	########	########	NA	NA
########	Yes	########	########	NA	########	########	########	NA
########	Yes	NA	NA	NA	NA	NA	NA	NA
########	Yes	NA	NA	NA	########	########	########	NA
########	Yes	NA	NA	NA	NA	########	NA	NA
########	Yes	NA	NA	NA	NA	NA	########	NA
########	Yes	########	########	NA	########	########	########	NA
########	Yes	########	NA	########	NA	NA	NA	NA
########	Yes	NA	NA	NA	########	########	########	NA
########	Yes	NA	NA	NA	NA	NA	########	NA
########	Yes	########	########	########	########	########	########	NA
########	Yes	########	########	NA	########	########	########	NA
########	Yes	########	########	NA	########	########	########	NA
########	Yes	NA	NA	NA	########	NA	########	NA
########	Yes	NA	NA	NA	NA	NA	########	NA
########	Yes	NA	NA	NA	NA	NA	NA	NA
########	Yes	NA	NA	NA	NA	NA	########	NA
########	Yes	NA	NA	NA	########	NA	########	NA
########	Yes	NA	NA	NA	NA	NA	########	NA
########	Yes	NA	NA	NA	NA	NA	########	NA
########	Yes	NA	NA	NA	########	########	########	NA
########	Yes	NA	NA	NA	NA	########	NA	NA
########	Yes	NA	NA	NA	NA	NA	########	NA
########	Yes	NA	NA	NA	NA	NA	NA	NA
########	Yes	NA	NA	NA	NA	NA	########	NA
########	Yes	NA	NA	NA	NA	NA	########	NA
########	Yes	NA	NA	NA	NA	NA	########	NA
########	Yes	NA	NA	NA	########	########	########	NA
########	Yes	NA	NA	NA	NA	########	NA	NA
########	Yes	########	########	NA	########	########	########	NA
########	Yes	########	########	########	########	########	########	NA
########	No	NA	NA	NA	NA	NA	NA	NA
########	Yes	NA	NA	NA	NA	NA	########	NA
########	Yes	NA	NA	NA	NA	NA	NA	NA
########	Yes	NA	NA	NA	NA	NA	NA	NA
########	No	NA	NA	NA	NA	NA	NA	NA
########	Yes	NA	NA	NA	########	########	NA	NA
########	Yes	NA	NA	NA	NA	NA	########	NA
########	Yes	NA	NA	NA	NA	NA	########	NA
########	Yes	########	########	########	########	NA	NA	NA
########	Yes	NA	NA	NA	########	########	########	NA
########	Yes	NA	########	########	NA	NA	########	########
########	Yes	NA	NA	NA	########	########	########	NA
########	Yes	NA	NA	NA	NA	########	########	NA
########	Yes	NA	NA	NA	########	########	NA	NA

For Review Only

########	Yes	NA	NA	NA	NA	NA	########	NA
########	Yes	########	########	NA	NA	NA	########	NA
########	Yes	NA	NA	NA	NA	NA	NA	NA
########	Yes	########	########	NA	########	########	########	NA
########	Yes	NA	NA	NA	NA	########	NA	NA
########	Yes	NA	########	NA	########	########	NA	########
########	Yes	NA	NA	########	########	NA	########	NA
########	Yes	########	########	########	########	########	########	NA
########	Yes	########	########	NA	NA	NA	NA	########
########	No	NA	NA	NA	NA	NA	NA	NA
########	Yes	NA	NA	NA	NA	NA	########	NA
########	Yes	NA	NA	NA	NA	NA	########	NA
########	Yes	NA	NA	########	########	########	########	NA
########	Yes	NA	NA	########	NA	NA	NA	NA
########	No	NA	NA	NA	NA	NA	NA	NA
########	Yes	########	########	NA	NA	NA	NA	NA
########	Yes	NA	########	NA	NA	NA	NA	NA
########	Yes	########	########	########	########	########	########	NA
########	Yes	NA	NA	NA	NA	NA	########	NA
########	Yes	NA	NA	NA	NA	########	NA	NA
########	Yes	########	########	NA	########	########	########	NA
########	No	NA	NA	NA	NA	NA	NA	NA
########	Yes	NA	NA	NA	NA	NA	NA	NA
########	Yes	NA	########	NA	NA	NA	NA	NA
########	No	NA	NA	NA	NA	NA	NA	NA
########	Yes	NA	NA	NA	NA	NA	########	NA
########	Yes	########	########	########	########	########	########	NA
########	Yes	NA	NA	NA	NA	NA	########	NA
########	Yes	########	NA	NA	NA	NA	########	NA
########	Yes	NA	NA	NA	NA	NA	########	NA
########	Yes	########	########	NA	########	########	########	NA
########	Yes	NA	NA	NA	########	########	########	NA
########	Yes	NA	NA	NA	NA	########	NA	NA
########	Yes	NA	NA	NA	NA	NA	########	NA
########	No	NA	NA	NA	NA	NA	NA	NA
########	Yes	NA	NA	########	########	########	########	NA
########	Yes	NA	NA	NA	########	########	NA	NA
########	Yes	NA	NA	NA	########	########	########	NA
########	Yes	NA	NA	NA	NA	NA	########	NA
########	Yes	NA	NA	NA	NA	########	########	NA
########	Yes	NA	NA	NA	NA	NA	########	NA
########	Yes	NA	NA	########	########	########	########	NA
########	Yes	NA	NA	NA	NA	NA	NA	NA
########	Yes	NA	NA	NA	########	########	########	NA
########	Yes	NA	NA	NA	########	########	NA	NA
########	Yes	NA	########	NA	NA	NA	NA	########
########	Yes	########	########	########	########	########	########	NA
########	No	NA	NA	NA	NA	NA	NA	NA
########	Yes	########	########	NA	########	########	########	NA
########	Yes	NA	NA	NA	NA	########	NA	NA
########	Yes	NA	NA	NA	NA	NA	########	NA
########	Yes	########	########	NA	########	NA	NA	NA
########	Yes	NA	NA	NA	NA	########	NA	NA
########	Yes	NA	NA	NA	NA	NA	########	NA
########	Yes	NA	NA	NA	NA	NA	########	NA

For Review Only

######## Yes	NA	NA	NA	NA	########	NA	NA
######## Yes	NA	NA	NA	NA	########	########	NA
######## Yes	NA	NA	NA	########	########	########	NA
######## Yes	########	########	NA	########	########	########	NA
######## Yes	NA	NA	NA	NA	NA	########	NA
######## Yes	NA	NA	NA	########	########	########	NA
######## Yes	NA	NA	NA	########	########	########	NA
######## Yes	NA	NA	NA	NA	########	########	NA
######## Yes	NA	NA	NA	########	########	########	NA
######## Yes	NA	NA	NA	########	NA	NA	NA
######## Yes	NA	NA	NA	NA	########	########	NA
######## Yes	NA	NA	NA	NA	NA	########	NA
######## Yes	########	NA	NA	########	########	########	NA
######## Yes	NA	########	NA	NA	NA	NA	NA
######## Yes	NA	NA	NA	NA	########	NA	NA
######## Yes	########	########	########	########	########	########	NA
######## Yes	NA	########	NA	NA	NA	########	NA
######## Yes	########	########	########	########	########	########	NA
######## Yes	NA	NA	NA	NA	NA	NA	NA
######## Yes	NA	NA	NA	NA	########	NA	NA
######## Yes	NA	NA	NA	NA	NA	NA	NA
######## Yes	NA	NA	########	NA	########	########	NA
######## Yes	NA	NA	NA	NA	NA	NA	NA
######## Yes	NA	NA	########	########	########	########	NA
######## Yes	########	########	########	NA	########	########	########
######## Yes	NA	NA	NA	NA	NA	NA	NA
######## Yes	NA	NA	NA	NA	########	NA	NA
######## Yes	NA	NA	NA	NA	########	########	NA
######## Yes	NA	NA	NA	NA	########	NA	NA
######## Yes	NA	NA	########	NA	NA	NA	NA
######## Yes	NA	NA	NA	########	########	NA	NA
######## Yes	########	########	########	########	########	########	NA
######## Yes	NA	NA	NA	NA	########	NA	NA
######## Yes	NA	NA	########	########	NA	########	NA
######## Yes	NA	NA	NA	NA	NA	NA	NA
######## Yes	NA	NA	NA	NA	########	########	NA
######## Yes	NA	NA	NA	########	########	########	NA
######## Yes	NA	NA	NA	########	########	########	NA
######## Yes	NA	NA	NA	NA	NA	########	NA
######## Yes	NA	########	########	NA	########	########	########
######## Yes	NA	NA	NA	NA	NA	NA	NA
######## Yes	########	########	NA	########	########	########	NA
######## Yes	NA	NA	NA	NA	NA	########	NA
######## Yes	NA	NA	NA	NA	########	NA	NA
######## Yes	NA	NA	NA	########	########	########	NA
######## Yes	NA	NA	NA	########	########	NA	NA
######## No	NA	NA	NA	NA	NA	NA	NA
######## Yes	NA	NA	NA	NA	########	########	NA
######## Yes	NA	NA	NA	########	########	########	NA
######## Yes	NA	NA	NA	########	########	########	NA
######## Yes	NA	NA	NA	NA	########	NA	NA
######## No	NA	NA	NA	NA	NA	NA	NA
######## Yes	########	########	########	NA	########	NA	NA

For Review Only

######## Yes	NA	NA	########	########	NA	NA	NA
######## Yes	NA	NA	NA	########	########	########	NA
######## Yes	NA	########	########	NA	NA	########	NA
######## Yes	NA	NA	########	########	NA	NA	NA
######## Yes	NA	NA	NA	NA	NA	########	NA
######## No	NA	NA	NA	NA	NA	NA	NA
######## Yes	NA	NA	NA	NA	########	########	NA
######## Yes	NA	NA	NA	########	NA	########	NA
######## Yes	########	########	NA	########	########	########	NA
######## Yes	########	########	NA	########	########	########	NA
######## Yes	NA	NA	NA	NA	########	NA	NA
######## Yes	########	########	NA	########	########	########	NA
######## Yes	NA	########	NA	NA	########	########	NA
######## Yes	NA	NA	NA	########	########	########	NA
######## Yes	NA	NA	########	########	NA	########	NA
######## Yes	NA	NA	NA	NA	########	########	NA
######## Yes	NA	########	NA	NA	NA	NA	########
######## Yes	NA	NA	NA	NA	########	NA	NA
######## Yes	NA	NA	NA	########	########	NA	NA
######## Yes	NA	NA	NA	NA	########	NA	NA
######## Yes	NA	NA	NA	NA	########	########	NA
######## Yes	NA	NA	NA	NA	NA	########	NA
######## Yes	NA	NA	########	########	########	########	NA
######## Yes	NA	NA	########	NA	NA	NA	NA
######## Yes	NA	NA	NA	NA	NA	########	NA
######## Yes	NA	NA	########	########	########	########	NA
######## Yes	NA	NA	NA	NA	NA	########	NA
######## Yes	NA	NA	NA	NA	NA	########	NA
######## Yes	NA	########	NA	NA	NA	NA	NA
######## Yes	NA	NA	NA	NA	NA	NA	NA
######## Yes	########	########	NA	########	########	########	NA
######## No	NA	NA	NA	NA	NA	NA	NA
######## Yes	NA	NA	########	########	########	########	NA
######## Yes	NA	NA	NA	########	NA	########	NA
######## Yes	NA	NA	NA	NA	NA	########	NA
######## Yes	########	########	########	########	########	########	NA
######## Yes	########	########	########	########	########	########	NA
######## Yes	NA	NA	NA	########	########	########	NA
######## No	NA	NA	NA	NA	NA	NA	NA
######## Yes	NA	NA	NA	NA	NA	########	NA
######## Yes	NA	NA	NA	########	########	########	NA
######## Yes	NA	NA	NA	########	NA	NA	NA
######## Yes	NA	NA	########	########	########	########	NA
######## Yes	########	########	NA	NA	NA	NA	NA
######## Yes	NA	NA	NA	NA	NA	########	NA
######## No	NA	NA	NA	NA	NA	NA	NA
######## Yes	NA	NA	NA	NA	NA	########	NA
######## Yes	NA	NA	NA	NA	########	NA	NA
######## Yes	NA	NA	NA	NA	NA	NA	NA
######## No	NA	NA	NA	NA	NA	NA	NA
######## Yes	NA	########	NA	NA	########	NA	NA
######## No	NA	NA	NA	NA	NA	NA	NA

For Review Only

######## Yes	NA	NA	NA	NA	NA	NA	NA
######## Yes	NA	NA	NA	NA	NA	########	NA
######## Yes	NA	NA	########	########	########	########	NA
######## Yes	NA	NA	NA	NA	NA	########	NA
######## Yes	NA	NA	NA	NA	NA	########	NA
######## Yes	NA	NA	NA	NA	########	########	NA
######## Yes	########	########	NA	########	########	########	NA
######## Yes	########	########	NA	########	########	########	NA
######## Yes	NA	NA	NA	NA	NA	########	NA
######## Yes	NA	NA	NA	NA	########	NA	NA
######## No	NA	NA	NA	NA	NA	NA	NA
######## Yes	NA	NA	NA	NA	########	NA	NA
######## Yes	NA	NA	NA	NA	NA	########	NA
######## No	NA	NA	NA	NA	NA	NA	NA
######## Yes	NA	NA	########	########	########	########	NA
######## Yes	NA	NA	NA	NA	########	########	NA
######## Yes	########	########	########	NA	NA	########	NA
######## Yes	NA	########	########	########	########	########	NA
######## Yes	NA	NA	NA	NA	########	########	NA
######## Yes	########	NA	NA	NA	NA	NA	NA
######## Yes	NA	########	########	########	########	NA	NA
######## Yes	NA	########	########	########	########	########	NA
######## Yes	NA	NA	########	########	########	########	NA
######## Yes	########	########	########	########	NA	########	NA
######## Yes	########	########	########	########	########	########	NA
######## Yes	NA	NA	NA	########	########	########	NA
######## Yes	NA	NA	NA	NA	NA	########	NA
######## Yes	########	########	########	NA	NA	########	NA
######## No	NA	NA	NA	NA	NA	NA	NA
######## Yes	NA	NA	NA	NA	########	NA	NA
######## Yes	NA	NA	NA	NA	NA	NA	NA
######## No	NA	NA	NA	NA	NA	NA	NA
######## Yes	########	########	NA	########	########	########	NA
######## Yes	########	########	NA	########	########	########	NA
######## Yes	NA	NA	NA	########	########	NA	NA
######## Yes	NA	NA	NA	NA	########	NA	NA
######## Yes	NA	NA	########	########	########	########	########
######## Yes	NA	NA	NA	NA	########	########	NA
######## Yes	NA	NA	NA	NA	NA	########	NA
######## Yes	NA	NA	NA	NA	NA	########	NA
######## No	NA	NA	NA	NA	NA	NA	NA
######## Yes	########	########	NA	########	########	########	NA
######## Yes	NA	NA	NA	NA	NA	########	NA
######## Yes	NA	NA	NA	NA	NA	########	NA
######## Yes	NA	NA	NA	########	########	########	NA
######## Yes	########	########	NA	NA	NA	########	NA
######## Yes	########	########	########	########	########	########	NA
######## No	NA	NA	NA	NA	NA	NA	NA
######## Yes	NA	NA	NA	NA	########	NA	NA
######## Yes	########	########	NA	########	########	NA	NA
######## Yes	########	########	########	########	########	########	NA
######## Yes	NA	NA	NA	NA	########	NA	NA

For Review Only

######## Yes	NA	NA	NA	NA	########	NA	NA
######## Yes	########	########	########	########	########	########	NA
######## Yes	########	########	NA	NA	########	########	NA
######## Yes	########	NA	########	NA	NA	########	NA
######## Yes	NA	NA	NA	NA	NA	########	NA
######## Yes	NA	NA	NA	NA	NA	########	NA
######## Yes	NA	NA	########	########	########	########	NA
######## No	NA	NA	NA	NA	NA	NA	NA
######## Yes	########	########	NA	NA	########	########	NA
######## Yes	NA	NA	NA	NA	########	NA	NA
######## Yes	########	NA	NA	NA	########	########	NA
######## Yes	########	########	NA	########	########	########	NA
######## No	NA	NA	NA	NA	NA	NA	NA
######## Yes	NA	NA	NA	########	########	########	NA
######## Yes	########	########	NA	########	########	NA	NA
######## Yes	########	########	########	NA	NA	########	########
######## Yes	########	########	NA	NA	########	NA	NA
######## Yes	NA	NA	NA	########	########	########	NA
######## Yes	NA	NA	NA	NA	NA	########	NA
######## Yes	NA	NA	NA	########	########	########	NA
######## No	NA	NA	NA	NA	NA	NA	NA
######## Yes	NA	NA	NA	NA	NA	########	NA
######## Yes	NA	NA	NA	NA	########	NA	NA
######## Yes	NA	NA	NA	NA	########	NA	NA
######## Yes	NA	NA	########	########	NA	NA	NA
######## Yes	NA	NA	NA	NA	NA	NA	NA
######## Yes	NA	NA	NA	########	########	########	NA
######## Yes	########	########	NA	NA	NA	########	NA
######## Yes	########	########	########	########	########	########	NA
######## Yes	NA	NA	NA	NA	NA	########	NA
######## Yes	########	NA	########	########	########	########	NA
######## Yes	########	NA	NA	NA	NA	NA	NA
######## No	NA	NA	NA	NA	NA	NA	NA
######## Yes	NA	NA	NA	NA	NA	NA	NA
######## Yes	NA	NA	NA	NA	NA	NA	NA
######## Yes	NA	NA	NA	NA	NA	########	NA
######## Yes	NA	NA	NA	NA	########	NA	NA
######## Yes	NA	NA	NA	NA	########	NA	NA
######## Yes	########	########	NA	########	########	########	########
######## Yes	NA	########	NA	NA	########	########	NA
######## Yes	NA	NA	NA	########	########	NA	NA
######## Yes	NA	NA	########	########	########	########	NA
######## Yes	NA	NA	NA	########	########	########	NA
######## Yes	NA	NA	########	########	########	########	NA
######## Yes	NA	NA	########	########	########	NA	NA
######## Yes	NA	NA	########	########	########	NA	NA
######## No	NA	NA	NA	NA	NA	NA	NA
######## Yes	NA	NA	NA	NA	########	NA	NA
######## Yes	NA	########	NA	########	NA	########	NA
######## Yes	########	########	########	########	########	########	NA
######## Yes	########	########	########	########	########	########	########
######## No	NA	NA	NA	NA	NA	NA	NA
######## Yes	NA	NA	NA	NA	NA	NA	NA
######## Yes	NA	NA	NA	NA	NA	########	NA
######## Yes	########	########	########	########	NA	########	NA
######## Yes	########	########	NA	########	########	########	NA

For Review Only

######## Yes	NA	NA	NA	NA	NA	########	NA
######## Yes	########	########	########	NA	NA	########	NA
######## Yes	NA	########	NA	NA	NA	NA	NA
######## Yes	########	########	########	########	########	########	NA
######## Yes	########	########	########	########	########	########	NA
######## Yes	########	########	########	########	########	########	NA
######## Yes	NA	NA	NA	NA	########	NA	NA
######## Yes	NA	NA	NA	########	########	########	NA
######## Yes	NA	NA	NA	NA	########	########	NA
######## No	NA	NA	NA	NA	NA	NA	NA
######## Yes	NA	########	########	########	########	########	NA
######## Yes	NA	NA	NA	NA	########	NA	NA
######## Yes	NA	NA	NA	########	########	NA	NA
######## Yes	NA	NA	NA	NA	########	NA	NA
######## Yes	NA	NA	NA	NA	########	NA	NA
######## Yes	NA	NA	NA	NA	########	NA	NA
######## Yes	NA	NA	NA	NA	########	NA	NA
######## Yes	NA	NA	NA	NA	########	NA	NA
######## Yes	NA	NA	########	########	########	########	NA
######## Yes	NA	NA	NA	NA	NA	NA	NA
######## Yes	NA	NA	NA	NA	NA	NA	NA
######## Yes	########	########	########	########	########	########	NA
######## Yes	NA	NA	NA	NA	NA	########	NA
######## Yes	NA	########	########	########	########	########	NA
######## Yes	NA	NA	NA	NA	NA	########	NA
######## Yes	NA	NA	NA	NA	NA	NA	NA
######## Yes	NA	NA	NA	NA	########	########	NA
######## Yes	NA	NA	########	NA	NA	########	NA
######## Yes	NA	NA	NA	NA	########	NA	NA
######## Yes	########	########	NA	########	########	########	NA
######## Yes	########	########	NA	########	########	########	NA
######## Yes	NA	NA	########	########	########	NA	NA
######## Yes	NA	NA	NA	NA	NA	########	NA
######## Yes	NA	NA	########	########	########	########	NA
######## No	NA	NA	NA	NA	NA	NA	NA
######## Yes	NA	NA	NA	NA	NA	########	NA
######## No	NA	NA	NA	NA	NA	NA	NA
######## Yes	NA	NA	NA	NA	NA	########	NA
######## No	NA	NA	NA	NA	NA	NA	NA
######## Yes	NA	NA	NA	########	NA	########	NA
######## Yes	NA	NA	NA	########	########	########	NA
######## Yes	NA	########	NA	NA	NA	########	NA
######## Yes	NA	NA	NA	########	NA	NA	NA
######## Yes	########	########	NA	NA	########	NA	########
######## Yes	NA	NA	NA	NA	NA	########	NA
######## Yes	NA	NA	NA	########	########	NA	NA
######## Yes	NA	NA	NA	NA	NA	########	NA
######## Yes	NA	NA	NA	NA	########	NA	NA
######## Yes	NA	NA	NA	NA	NA	NA	NA
######## Yes	NA	NA	NA	NA	NA	########	NA
######## Yes	NA	NA	NA	NA	NA	########	NA
######## Yes	########	NA	NA	NA	NA	########	NA
######## Yes	NA	NA	NA	NA	########	########	NA
######## Yes	########	########	NA	########	########	########	NA
######## Yes	NA	NA	NA	NA	########	########	NA

For Review Only

########	Yes	NA	########	NA	########	########	########	NA
########	Yes	NA	NA	NA	NA	NA	########	NA
########	Yes	########	########	########	########	########	########	NA
########	Yes	NA	NA	NA	NA	NA	########	NA
########	Yes	NA	NA	NA	########	########	########	NA
########	Yes	NA	NA	NA	NA	NA	NA	NA
########	Yes	NA	NA	NA	NA	########	########	NA
########	Yes	NA	NA	NA	NA	########	########	NA
########	Yes	NA	########	NA	NA	########	########	NA
########	Yes	########	########	########	########	########	########	NA
########	Yes	NA	NA	NA	########	NA	########	NA
########	Yes	NA	NA	NA	NA	NA	########	NA
########	Yes	NA	NA	NA	NA	NA	########	NA
########	Yes	NA	NA	NA	NA	########	########	NA
########	Yes	NA	NA	NA	########	########	########	NA
########	Yes	########	########	NA	########	########	########	NA
########	No	NA	NA	NA	NA	NA	NA	NA
########	Yes	NA	NA	NA	########	########	########	NA
########	Yes	NA	NA	NA	NA	########	########	NA
########	Yes	NA	NA	NA	NA	NA	########	NA
########	Yes	NA	NA	NA	NA	NA	########	NA
########	Yes	NA	NA	NA	NA	NA	########	NA
########	Yes	NA	NA	########	NA	NA	NA	NA
########	Yes	NA	NA	NA	NA	########	########	NA
########	Yes	########	########	NA	########	########	########	NA
########	No	NA	NA	NA	NA	NA	NA	NA
########	Yes	########	########	NA	########	########	########	NA
########	Yes	NA	NA	NA	########	########	########	NA
########	Yes	NA	NA	NA	NA	########	########	NA
########	Yes	NA	NA	########	NA	NA	NA	########
########	Yes	NA	NA	NA	NA	NA	########	NA
########	Yes	NA	NA	NA	NA	NA	########	NA
########	Yes	########	########	NA	NA	########	########	NA
########	Yes	NA	NA	NA	NA	NA	########	NA
########	Yes	NA	NA	NA	########	########	########	NA
########	Yes	NA	NA	NA	NA	########	########	NA
########	No	NA	NA	NA	NA	NA	NA	NA
########	Yes	NA	NA	########	########	NA	########	########
########	Yes	NA	NA	NA	NA	########	NA	NA
########	Yes	########	########	NA	NA	########	########	NA
########	Yes	########	########	NA	NA	NA	########	NA
########	Yes	NA	NA	########	########	########	########	NA
########	Yes	NA	NA	NA	########	########	NA	NA
########	Yes	NA	NA	########	NA	NA	########	NA
########	Yes	NA	########	########	########	########	NA	NA
########	Yes	NA	NA	########	########	########	########	NA
########	Yes	NA	NA	NA	########	NA	########	NA
########	Yes	NA	NA	NA	NA	NA	NA	NA
########	Yes	NA	NA	NA	NA	########	########	NA
########	Yes	########	########	########	NA	NA	########	NA
########	Yes	########	########	NA	########	########	########	NA
########	Yes	NA	NA	NA	NA	NA	NA	NA
########	Yes	NA	NA	NA	NA	NA	NA	NA
########	Yes	NA	NA	NA	NA	########	########	NA
########	Yes	########	########	########	########	########	########	NA
########	Yes	NA	NA	NA	NA	NA	########	NA

For Review Only

######## Yes	NA	NA	########	########	NA	########	NA
######## Yes	########	########	NA	########	########	########	NA
######## Yes	NA	########	########	########	########	########	########
######## Yes	NA	########	NA	########	NA	########	NA
######## Yes	NA	NA	########	########	########	NA	NA
######## Yes	NA	NA	NA	NA	NA	########	NA
######## Yes	########	########	########	########	########	########	NA
######## No	NA	NA	NA	NA	NA	NA	NA
######## Yes	########	########	########	########	########	########	NA
######## Yes	NA	NA	NA	NA	NA	########	NA
######## Yes	NA	NA	NA	NA	NA	NA	NA
######## No	NA	NA	NA	NA	NA	NA	NA
######## Yes	########	########	NA	########	########	########	NA
######## Yes	########	########	########	########	########	########	NA
######## Yes	NA	NA	NA	NA	########	########	NA
######## Yes	########	########	########	########	########	########	NA
######## Yes	########	########	########	########	########	########	NA
######## Yes	NA	NA	NA	NA	########	########	NA
######## Yes	NA	NA	NA	NA	########	########	NA
######## Yes	NA	NA	NA	NA	NA	########	NA
######## Yes	NA	NA	NA	NA	NA	########	NA
######## Yes	NA	NA	NA	NA	NA	NA	NA
######## Yes	########	########	########	########	########	########	NA
######## Yes	NA	NA	NA	########	########	########	NA
######## Yes	NA	NA	NA	NA	########	########	NA
######## Yes	########	########	NA	NA	NA	NA	NA
######## Yes	NA	NA	NA	NA	NA	########	NA
######## Yes	NA	NA	NA	NA	NA	########	NA
######## No	NA	NA	NA	NA	NA	NA	NA
######## Yes	NA	NA	NA	NA	NA	########	NA
######## Yes	########	########	########	NA	NA	NA	NA
######## Yes	NA	NA	NA	NA	NA	########	NA
######## Yes	NA	NA	NA	NA	NA	NA	NA
######## No	NA	NA	NA	NA	NA	NA	NA
######## Yes	NA	NA	NA	NA	NA	########	NA
######## Yes	NA	NA	NA	NA	NA	########	NA
######## Yes	########	########	NA	########	########	########	NA
######## No	NA	NA	NA	NA	NA	NA	NA
######## Yes	########	########	NA	########	########	########	NA
######## Yes	########	NA	NA	NA	########	########	########
######## Yes	NA	NA	NA	NA	NA	########	NA
######## Yes	NA	NA	NA	NA	########	########	NA
######## Yes	########	########	########	########	########	########	NA
######## Yes	NA	NA	########	########	########	NA	NA
######## Yes	NA	########	NA	NA	NA	########	NA
######## Yes	NA	NA	NA	NA	NA	########	NA
######## Yes	NA	NA	NA	NA	########	NA	NA
######## Yes	NA	NA	NA	NA	########	NA	NA
######## Yes	NA	NA	NA	NA	NA	NA	NA
######## Yes	NA	NA	NA	########	########	########	NA
######## No	NA	NA	NA	NA	NA	NA	NA
######## No	NA	NA	NA	NA	NA	NA	NA
######## Yes	########	########	NA	########	########	########	NA
######## Yes	NA	NA	NA	NA	NA	########	NA
######## Yes	NA	NA	NA	NA	NA	########	NA
######## Yes	NA	NA	########	########	########	NA	NA

For Review Only

######## Yes	NA	NA	NA	NA	NA	########	NA
######## Yes	NA	NA	NA	NA	NA	########	NA
######## Yes	NA	NA	NA	NA	########	NA	NA
######## Yes	NA	NA	NA	NA	NA	########	NA
######## No	NA	NA	NA	NA	NA	NA	NA
######## Yes	NA	NA	NA	NA	NA	########	NA
######## No	NA	NA	NA	NA	NA	NA	NA
######## Yes	NA	########	NA	NA	NA	NA	NA
######## Yes	NA	NA	NA	NA	NA	########	NA
######## Yes	NA	NA	NA	NA	########	########	NA
######## Yes	NA	NA	########	########	########	########	NA
######## Yes	########	########	########	########	NA	########	NA
######## Yes	########	########	########	########	########	########	NA
######## Yes	########	########	########	########	########	########	NA
######## Yes	########	########	########	NA	NA	########	########
######## Yes	NA	NA	########	########	########	NA	NA
######## Yes	NA	NA	NA	NA	NA	NA	NA
######## Yes	NA	NA	NA	NA	NA	NA	NA
######## Yes	NA	NA	NA	NA	NA	NA	NA
######## Yes	NA	NA	NA	NA	NA	########	NA
######## Yes	########	########	########	########	########	########	NA
######## Yes	NA	NA	NA	########	########	NA	NA
######## Yes	NA	NA	NA	NA	########	########	NA
######## Yes	########	########	######## NA		########	########	NA
######## Yes	########	########	NA	########	########	########	NA
######## Yes	NA	NA	NA	NA	NA	########	NA
######## Yes	NA	NA	NA	NA	########	NA	NA
######## Yes	NA	NA	NA	NA	NA	########	NA
######## Yes	########	########	NA	########	########	########	NA
######## Yes	########	########	NA	NA	NA	NA	NA
######## Yes	NA	NA	NA	########	########	########	NA
######## Yes	NA	########	NA	NA	NA	NA	NA
######## Yes	########	########	NA	########	########	########	NA
######## Yes	NA	NA	NA	NA	NA	NA	NA
######## Yes	NA	NA	NA	########	########	########	NA
######## Yes	NA	NA	NA	########	########	NA	NA
######## Yes	NA	NA	NA	NA	NA	NA	NA
######## Yes	NA	NA	NA	NA	NA	########	NA
######## Yes	NA	NA	NA	NA	########	########	NA
######## Yes	NA	NA	NA	########	NA	########	NA
######## Yes	NA	NA	NA	########	########	NA	NA
######## No	NA	NA	NA	NA	NA	NA	NA
######## Yes	NA	NA	NA	NA	NA	########	NA
######## Yes	NA	NA	NA	NA	NA	########	NA
######## Yes	NA	NA	NA	NA	########	NA	NA
######## Yes	NA	NA	NA	########	########	########	NA
######## Yes	NA	NA	########	########	########	########	NA
######## Yes	NA	NA	NA	NA	########	NA	NA
######## Yes	NA	NA	NA	NA	NA	########	NA
######## Yes	########	########	########	########	NA	########	NA
######## No	NA	NA	NA	NA	NA	NA	NA
######## Yes	NA	NA	NA	NA	NA	########	NA
######## Yes	NA	NA	NA	NA	########	NA	NA
######## Yes	NA	NA	NA	########	########	########	NA

For Review Only

######## Yes	NA	NA	NA	NA	########	########	NA
######## Yes	NA	NA	NA	NA	########	########	NA
######## Yes	NA	NA	NA	NA	########	NA	NA
######## Yes	NA	NA	########	########	########	########	NA
######## Yes	NA	########	########	NA	########	########	NA
######## Yes	NA	NA	NA	NA	NA	########	NA
######## No	NA	NA	NA	NA	NA	NA	NA
######## Yes	NA	NA	NA	NA	NA	NA	NA
######## Yes	NA	NA	NA	########	########	########	NA
######## No	NA	NA	NA	NA	NA	NA	NA
######## Yes	NA	########	NA	########	########	########	NA
######## Yes	NA	NA	NA	NA	NA	NA	NA
######## Yes	NA	NA	NA	NA	NA	########	NA
######## Yes	NA	NA	NA	NA	NA	########	NA
######## Yes	NA	NA	NA	NA	NA	########	NA
######## Yes	########	NA	NA	########	########	########	NA
######## Yes	NA	NA	NA	NA	NA	########	NA
######## Yes	NA	NA	NA	NA	NA	NA	NA
######## Yes	NA	NA	########	########	########	########	NA
######## Yes	NA	NA	NA	NA	NA	NA	NA
######## No	NA	NA	NA	NA	NA	NA	NA
######## Yes	NA	NA	NA	NA	########	########	NA
######## Yes	NA	NA	########	NA	########	########	NA
######## Yes	NA	NA	NA	NA	########	NA	NA
######## Yes	NA	NA	NA	NA	########	NA	NA
######## Yes	NA	NA	NA	NA	########	NA	NA
######## Yes	NA	NA	NA	NA	NA	########	NA
######## Yes	NA	NA	NA	NA	########	NA	NA
######## Yes	NA	NA	NA	NA	NA	########	NA
######## Yes	NA	NA	NA	NA	########	NA	NA
######## Yes	NA	NA	NA	########	########	########	NA
######## Yes	NA	NA	NA	NA	NA	########	NA
######## Yes	NA	NA	NA	NA	########	########	NA
######## Yes	########	########	NA	NA	########	########	NA
######## Yes	NA	NA	NA	NA	NA	NA	NA
######## Yes	NA	NA	NA	NA	########	########	NA
######## Yes	NA	NA	NA	NA	########	########	NA
######## Yes	NA	NA	NA	NA	NA	NA	NA
######## Yes	NA	NA	NA	NA	NA	########	NA
######## Yes	########	NA	NA	NA	NA	########	NA
######## Yes	NA	NA	NA	NA	NA	########	NA
######## Yes	NA	NA	NA	########	########	########	NA
######## Yes	NA	NA	NA	########	########	########	NA
######## Yes	########	########	########	########	########	########	NA
######## Yes	NA	NA	NA	NA	########	########	NA
######## Yes	NA	NA	NA	NA	NA	NA	NA
######## No	NA	NA	NA	NA	NA	NA	NA
######## Yes	NA	NA	NA	NA	########	NA	NA
######## Yes	NA	NA	NA	NA	########	########	NA
######## Yes	NA	NA	NA	########	########	########	NA
######## Yes	########	########	NA	NA	NA	########	NA
######## Yes	########	########	########	NA	NA	########	NA
######## Yes	NA	NA	NA	NA	########	NA	NA

For Review Only

########	Yes	NA	NA	NA	NA	########	NA	NA
########	Yes	NA	NA	NA	NA	########	NA	NA
########	Yes	NA	NA	NA	########	########	NA	NA
########	Yes	NA	NA	NA	NA	########	NA	NA
########	Yes	NA	NA	NA	########	########	NA	NA
########	Yes	NA	NA	NA	NA	########	NA	NA
########	Yes	NA	NA	NA	NA	########	NA	NA
########	Yes	NA	NA	NA	NA	########	NA	NA
########	Yes	NA	NA	NA	NA	########	NA	NA
########	Yes	NA	NA	NA	NA	########	NA	NA
########	Yes	NA	NA	NA	NA	NA	NA	NA
########	Yes	NA	NA	NA	NA	########	NA	NA
########	Yes	NA	NA	NA	NA	NA	########	NA
########	Yes	########	########	NA	NA	########	NA	NA
########	Yes	NA	NA	NA	NA	########	NA	NA
########	Yes	NA	NA	NA	NA	########	NA	NA
########	Yes	NA	NA	NA	NA	########	########	NA
########	Yes	NA	NA	NA	########	########	########	NA
########	Yes	########	########	########	########	NA	########	NA
########	Yes	NA	NA	NA	NA	########	NA	NA
########	Yes	NA	NA	NA	NA	########	NA	NA
########	Yes	NA	NA	NA	NA	########	NA	NA
########	Yes	NA	NA	########	########	########	########	NA
########	Yes	NA	NA	NA	NA	########	NA	NA
########	Yes	NA	NA	NA	NA	########	NA	NA
########	Yes	NA	NA	NA	NA	NA	########	NA
########	Yes	########	########	########	########	########	########	NA
########	Yes	########	NA	########	########	########	NA	NA
########	Yes	NA	NA	NA	NA	NA	########	NA
########	Yes	NA	NA	NA	NA	########	NA	NA
########	Yes	NA	NA	NA	NA	########	########	NA
########	Yes	NA	NA	NA	NA	NA	########	NA
########	Yes	NA	NA	NA	########	########	########	NA
########	Yes	NA	NA	NA	NA	########	NA	NA
########	Yes	NA	NA	NA	NA	########	NA	NA
########	Yes	NA	NA	NA	NA	########	NA	NA
########	Yes	NA	NA	NA	NA	########	NA	NA
########	Yes	NA	NA	NA	NA	########	NA	NA
########	Yes	NA	NA	########	########	########	########	NA
########	Yes	NA	NA	NA	########	NA	########	NA
########	Yes	NA	NA	NA	NA	########	NA	NA
########	Yes	NA	NA	NA	NA	########	NA	NA
########	Yes	NA	NA	NA	NA	########	NA	NA
########	Yes	NA	NA	NA	NA	########	NA	NA
########	Yes	NA	NA	NA	NA	########	NA	NA
########	Yes	NA	NA	NA	########	########	NA	NA
########	Yes	NA	NA	NA	NA	NA	########	NA
########	Yes	NA	NA	NA	NA	NA	########	NA
########	No	NA	NA	NA	NA	NA	NA	NA
########	Yes	NA	NA	NA	NA	NA	########	NA
########	Yes	########	########	########	########	########	########	NA
########	Yes	NA	NA	NA	########	########	NA	NA
########	Yes	NA	NA	NA	NA	########	NA	NA
########	Yes	NA	NA	NA	########	########	NA	NA
########	Yes	########	########	########	########	########	########	NA
########	Yes	NA	NA	########	########	########	########	NA

For Review Only

######## Yes	NA	NA	NA	NA	NA	########	NA
######## Yes	NA	NA	NA	NA	########	########	NA
######## Yes	NA	NA	NA	NA	########	NA	NA
######## Yes	NA	NA	NA	NA	########	NA	NA
######## Yes	NA	NA	NA	NA	NA	########	NA
######## Yes	NA	NA	NA	NA	NA	NA	NA
######## Yes	NA	NA	NA	########	########	NA	NA
######## Yes	NA	NA	NA	NA	NA	########	NA
######## Yes	NA	NA	NA	NA	NA	########	NA
######## Yes	NA	NA	NA	########	########	NA	NA
######## Yes	NA	NA	NA	NA	########	NA	NA
######## No	NA	NA	NA	NA	NA	NA	NA
######## Yes	NA	NA	NA	########	########	NA	NA
######## Yes	NA	########	########	########	########	########	NA
######## Yes	NA	NA	NA	########	########	########	NA
######## Yes	NA	NA	NA	########	########	NA	NA
######## Yes	NA	NA	NA	NA	NA	########	NA
######## Yes	NA	NA	NA	NA	NA	########	NA
######## No	NA	NA	NA	NA	NA	NA	NA
######## Yes	NA	NA	NA	########	########	########	NA
######## Yes	NA	NA	NA	NA	NA	########	NA
######## Yes	########	########	########	########	########	########	NA
######## Yes	NA	########	########	########	########	########	NA
######## Yes	########	########	########	########	########	########	NA
######## Yes	NA	########	########	NA	NA	########	NA
######## Yes	NA	NA	NA	NA	NA	########	NA
######## Yes	########	########	########	########	########	########	NA
######## Yes	NA	NA	NA	NA	NA	########	NA
######## Yes	########	NA	########	NA	########	########	########
######## No	NA	NA	NA	NA	NA	NA	NA
######## Yes	NA	NA	NA	NA	NA	########	NA
######## Yes	NA	NA	NA	NA	NA	########	NA
######## Yes	########	NA	NA	########	########	########	NA
######## Yes	########	########	########	########	########	########	NA
######## Yes	########	NA	########	########	########	########	########
######## Yes	NA	NA	NA	NA	########	########	NA
######## Yes	########	NA	NA	NA	NA	########	NA
######## Yes	NA	NA	NA	NA	########	NA	NA
######## No	NA	NA	NA	NA	NA	NA	NA
######## Yes	NA	NA	NA	NA	########	########	NA
######## Yes	NA	NA	NA	########	########	########	NA
######## Yes	NA	NA	NA	########	NA	NA	NA
######## Yes	########	########	########	########	########	########	NA
######## Yes	NA	NA	NA	NA	########	NA	NA
######## Yes	########	########	NA	NA	########	########	NA
######## Yes	NA	NA	NA	########	########	NA	NA
######## Yes	########	########	NA	NA	NA	NA	NA
######## Yes	NA	NA	NA	NA	########	NA	NA
######## Yes	NA	NA	NA	NA	NA	########	NA
######## Yes	NA	NA	NA	NA	NA	########	NA
######## Yes	########	########	NA	NA	NA	########	NA
######## No	NA	NA	NA	NA	NA	NA	NA
######## Yes	NA	NA	NA	NA	NA	########	NA
######## Yes	NA	NA	NA	NA	NA	########	NA
######## Yes	NA	NA	NA	NA	########	NA	NA
######## Yes	########	########	NA	NA	########	########	NA

For Review Only

######## Yes	########	########	########	########	########	########	NA
######## Yes	########	########	########	########	########	########	NA
######## No	NA	NA	NA	NA	NA	NA	NA
######## No	NA	NA	NA	NA	NA	NA	NA
######## Yes	NA	NA	########	########	########	########	NA
######## Yes	NA	NA	NA	########	########	########	NA
######## Yes	NA	NA	########	########	########	########	NA
######## Yes	########	NA	NA	########	NA	########	NA
######## Yes	########	########	NA	########	########	########	NA
######## Yes	NA	NA	########	NA	NA	########	NA
######## Yes	########	########	########	########	########	########	NA
######## Yes	NA	NA	NA	NA	NA	########	NA
######## No	NA	NA	NA	NA	NA	NA	NA
######## Yes	NA	NA	NA	########	########	NA	NA
######## Yes	NA	NA	NA	NA	########	NA	NA
######## Yes	NA	NA	NA	NA	########	NA	NA
######## Yes	NA	NA	NA	NA	NA	########	NA
######## Yes	########	########	########	########	########	########	NA
######## Yes	NA	########	########	########	########	########	NA
######## Yes	########	########	########	########	########	########	NA
######## Yes	NA	NA	NA	NA	NA	########	NA
######## Yes	NA	NA	NA	NA	########	NA	NA
######## No	NA	NA	NA	NA	NA	NA	NA
######## Yes	NA	NA	NA	NA	NA	########	NA
######## Yes	NA	NA	NA	NA	NA	########	NA
######## Yes	NA	NA	NA	########	########	########	NA
######## Yes	NA	NA	NA	NA	NA	########	NA
######## Yes	NA	NA	NA	NA	NA	NA	NA
######## Yes	########	########	NA	########	########	########	NA
######## Yes	NA	NA	########	########	########	########	NA
######## No	NA	NA	NA	NA	NA	NA	NA
######## Yes	NA	NA	NA	NA	NA	########	NA
######## No	NA	NA	NA	NA	NA	NA	NA
######## Yes	NA	NA	NA	NA	########	NA	NA
######## Yes	NA	NA	NA	NA	########	NA	NA
######## Yes	NA	########	########	########	########	########	NA
######## No	NA	NA	NA	NA	NA	NA	NA
######## Yes	NA	NA	########	########	NA	NA	NA
######## Yes	NA	NA	NA	NA	########	NA	NA
######## Yes	########	########	########	########	NA	########	NA
######## Yes	########	########	########	########	########	########	NA
######## Yes	########	########	NA	########	########	########	########
######## Yes	NA	NA	NA	NA	########	########	NA
######## No	NA	NA	NA	NA	NA	NA	NA
######## Yes	NA	NA	NA	NA	########	NA	NA
######## Yes	NA	NA	NA	NA	NA	########	NA
######## Yes	########	########	########	########	########	########	NA
######## No	NA	NA	NA	NA	NA	NA	NA
######## Yes	NA	NA	NA	NA	NA	########	NA
######## Yes	NA	NA	NA	NA	NA	########	NA
######## Yes	NA	NA	NA	NA	########	NA	NA

For Review Only

######## Yes	NA	NA	NA	NA	NA	NA	NA
######## Yes	NA	NA	NA	########	########	########	NA
######## Yes	NA	NA	NA	NA	NA	########	NA
######## Yes	########	########	NA	########	########	NA	NA
######## Yes	NA	NA	NA	########	########	########	NA
######## Yes	NA	NA	NA	########	########	########	NA
######## Yes	NA	NA	NA	NA	NA	########	NA
######## Yes	NA	NA	NA	NA	NA	########	NA
######## Yes	NA	NA	NA	NA	NA	NA	NA
######## Yes	NA	NA	NA	NA	NA	########	NA
######## Yes	NA	NA	NA	NA	NA	########	NA
######## Yes	NA	NA	NA	NA	########	NA	NA
######## Yes	NA	NA	NA	########	########	########	NA
######## No	NA	NA	NA	NA	NA	NA	NA
######## Yes	NA	NA	NA	########	NA	########	NA
######## Yes	NA	NA	NA	NA	########	########	NA
######## Yes	NA	NA	NA	NA	########	NA	NA
######## Yes	########	########	NA	########	########	########	NA
######## Yes	NA	NA	########	########	NA	NA	NA
######## Yes	NA	NA	NA	########	########	########	NA
######## Yes	########	########	########	########	########	########	NA
######## Yes	NA	NA	NA	########	########	########	NA
######## Yes	NA	NA	########	########	########	NA	NA
######## Yes	########	########	########	NA	########	########	NA
######## Yes	NA	NA	NA	########	########	########	NA
######## Yes	NA	NA	NA	NA	NA	NA	NA
######## Yes	NA	NA	NA	NA	NA	########	NA
######## Yes	########	########	########	########	########	########	NA
######## Yes	NA	NA	NA	NA	NA	NA	NA
######## Yes	NA	NA	NA	NA	########	########	NA
######## Yes	NA	NA	NA	NA	########	NA	NA
######## Yes	########	########	NA	NA	########	########	NA
######## Yes	NA	########	########	########	########	########	NA
######## No	NA	NA	NA	NA	NA	NA	NA
######## Yes	NA	NA	NA	NA	NA	########	NA
######## Yes	NA	NA	NA	########	NA	########	NA
######## Yes	NA	NA	NA	NA	NA	########	NA
######## Yes	NA	NA	NA	NA	NA	NA	NA
######## Yes	NA	NA	NA	########	NA	########	NA
######## No	NA	NA	NA	NA	NA	NA	NA
######## Yes	NA	NA	NA	NA	NA	########	NA
######## Yes	########	########	########	########	########	########	NA
######## Yes	NA	NA	NA	NA	NA	NA	NA
######## Yes	NA	NA	NA	NA	NA	########	NA
######## Yes	NA	NA	NA	NA	NA	NA	NA
######## Yes	########	########	NA	NA	NA	NA	NA
######## Yes	########	########	########	########	########	########	NA
######## Yes	NA	NA	NA	NA	NA	########	NA
######## Yes	NA	NA	NA	NA	NA	########	NA
######## Yes	NA	NA	NA	NA	NA	########	########
######## No	NA	NA	NA	NA	NA	NA	NA
######## Yes	NA	NA	########	########	NA	NA	NA
######## Yes	NA	NA	NA	NA	NA	NA	NA
######## Yes	NA	########	NA	########	########	########	########
######## Yes	NA	NA	NA	########	########	########	NA
######## Yes	########	NA	NA	NA	NA	NA	NA

For Review Only

######## Yes	NA	NA	NA	NA	########	NA	NA
######## Yes	########	########	########	########	NA	########	NA
######## Yes	########	########	########	########	NA	NA	NA
######## Yes	NA	NA	NA	########	NA	NA	NA
######## Yes	########	########	NA	########	########	########	NA
######## Yes	########	########	########	########	########	########	NA
######## Yes	NA	NA	NA	NA	NA	########	NA
######## Yes	NA	NA	########	NA	NA	NA	NA
######## Yes	NA	NA	NA	NA	########	########	NA
######## Yes	NA	NA	NA	########	########	########	NA
######## Yes	NA	########	NA	NA	NA	NA	NA
######## No	NA	NA	NA	NA	NA	NA	NA
######## Yes	NA	NA	NA	NA	NA	########	NA
######## Yes	NA	NA	NA	NA	########	########	NA
######## Yes	NA	NA	NA	NA	########	########	NA
######## Yes	NA	NA	NA	NA	NA	########	NA
######## No	NA	NA	NA	NA	NA	NA	NA
######## No	NA	NA	NA	NA	NA	NA	NA
######## Yes	NA	NA	NA	NA	NA	########	NA
######## Yes	########	########	NA	########	########	########	NA
######## Yes	########	########	########	########	########	########	NA
######## Yes	########	########	########	########	########	########	NA
######## Yes	NA	NA	NA	NA	NA	########	NA
######## Yes	NA	NA	########	########	########	NA	NA
######## Yes	NA	NA	########	NA	NA	NA	NA
######## Yes	NA	NA	NA	NA	NA	########	NA
######## Yes	########	########	########	########	########	NA	NA
######## Yes	########	########	########	########	########	########	NA
######## Yes	NA	NA	########	NA	NA	NA	NA
######## Yes	########	NA	NA	NA	NA	NA	NA
######## Yes	NA	NA	NA	NA	NA	########	NA
######## No	NA	NA	NA	NA	NA	NA	NA
######## Yes	NA	NA	NA	NA	NA	NA	NA
######## Yes	########	########	########	########	########	########	NA
######## Yes	NA	NA	NA	NA	NA	########	NA
######## Yes	########	NA	NA	NA	########	########	NA
######## Yes	NA	NA	########	########	########	NA	NA
######## Yes	NA	NA	NA	NA	########	NA	NA
######## Yes	NA	NA	NA	########	########	########	NA
######## Yes	########	########	########	########	########	########	NA
######## Yes	########	########	NA	########	########	########	NA
######## Yes	NA	NA	########	########	NA	########	NA
######## Yes	NA	NA	NA	NA	########	########	NA
######## Yes	NA	NA	NA	########	########	########	NA
######## Yes	NA	NA	NA	########	########	NA	NA
######## Yes	NA	NA	NA	NA	########	########	NA
######## Yes	NA	NA	NA	NA	NA	NA	NA
######## Yes	NA	########	NA	NA	NA	NA	NA
######## No	NA	NA	NA	NA	NA	NA	NA
######## Yes	NA	NA	NA	NA	NA	NA	NA
######## Yes	########	########	########	########	########	########	NA
######## Yes	NA	NA	NA	########	########	########	NA

For Review Only

######## Yes	NA	NA	NA	NA	########	########	NA
######## Yes	NA	NA	NA	NA	########	########	NA
######## Yes	NA	NA	NA	########	########	########	NA
######## Yes	NA	NA	NA	NA	NA	########	NA
######## No	NA	NA	NA	NA	NA	NA	NA
######## Yes	NA	NA	NA	NA	NA	NA	NA
######## Yes	NA	NA	NA	NA	NA	########	NA
######## Yes	NA	########	########	########	########	########	NA
######## Yes	NA	NA	NA	NA	NA	NA	NA
######## Yes	NA	NA	NA	NA	NA	NA	NA
######## Yes	NA	NA	NA	NA	########	########	NA
######## Yes	NA	NA	NA	NA	NA	########	NA
######## Yes	NA	NA	NA	NA	NA	########	NA
######## Yes	NA	NA	NA	NA	NA	NA	NA
######## Yes	NA	NA	NA	NA	########	NA	NA
######## Yes	########	NA	NA	NA	########	########	NA
######## Yes	NA	NA	NA	########	########	NA	NA
######## Yes	########	########	########	########	########	########	NA
######## Yes	NA	########	NA	########	########	########	NA
######## Yes	########	########	########	########	########	########	NA
######## Yes	NA	NA	NA	NA	NA	########	NA
######## Yes	NA	NA	NA	########	########	########	NA
######## Yes	NA	NA	NA	NA	NA	NA	NA
######## Yes	########	########	########	########	########	########	NA
######## Yes	NA	NA	NA	NA	NA	########	NA
######## No	NA	NA	NA	NA	NA	NA	NA
######## Yes	########	########	########	########	########	########	NA
######## Yes	########	NA	########	NA	NA	NA	NA
######## Yes	NA	NA	NA	########	########	########	NA
######## Yes	########	########	########	########	########	NA	NA
######## Yes	NA	NA	########	########	########	########	NA
######## Yes	########	########	########	########	########	########	NA
######## Yes	NA	NA	NA	NA	NA	########	NA
######## Yes	NA	NA	NA	NA	NA	########	NA
######## Yes	NA	NA	NA	NA	########	########	NA
######## Yes	NA	NA	NA	########	NA	NA	NA
######## Yes	NA	NA	NA	NA	########	NA	NA
######## Yes	NA	NA	NA	########	########	########	NA
######## Yes	NA	NA	NA	########	########	########	NA
######## Yes	NA	NA	NA	########	########	NA	NA
######## Yes	NA	NA	NA	NA	########	########	NA
######## No	NA	NA	NA	NA	NA	NA	NA
######## Yes	NA	NA	########	########	NA	NA	NA
######## Yes	NA	NA	NA	NA	NA	NA	NA
######## Yes	NA	NA	NA	########	########	########	NA
######## Yes	NA	NA	NA	NA	NA	NA	NA
######## Yes	NA	NA	NA	NA	NA	########	NA
######## Yes	NA	NA	NA	########	########	########	NA
######## Yes	NA	########	NA	########	########	########	NA
######## Yes	########	########	########	########	NA	########	NA
######## Yes	NA	NA	NA	NA	NA	########	NA
######## No	NA	NA	NA	NA	NA	NA	NA
######## Yes	NA	NA	NA	########	########	NA	NA
######## Yes	NA	NA	NA	NA	NA	########	NA
######## Yes	NA	NA	########	NA	NA	NA	NA
######## Yes	NA	########	########	########	########	########	NA

For Review Only

######## No	NA	NA	NA	NA	NA	NA	NA
######## Yes	NA	NA	NA	NA	########	########	NA
######## Yes	NA	NA	########	########	NA	########	NA
######## Yes	NA	NA	NA	NA	NA	########	NA
######## Yes	NA	NA	NA	NA	NA	########	NA
######## No	NA	NA	NA	NA	NA	NA	NA
######## Yes	NA	NA	NA	########	########	########	NA
######## Yes	NA	NA	NA	NA	NA	########	NA
######## Yes	NA	NA	NA	NA	########	NA	NA
######## Yes	NA	NA	########	########	########	NA	NA
######## Yes	NA	NA	NA	NA	NA	########	NA
######## Yes	NA	NA	NA	########	########	########	NA
######## Yes	NA	########	NA	########	########	########	NA
######## Yes	NA	NA	NA	NA	########	NA	NA
######## Yes	########	########	########	########	########	########	NA
######## Yes	NA	NA	########	########	NA	NA	NA
######## Yes	NA	NA	########	########	NA	########	NA
######## Yes	NA	NA	NA	NA	NA	NA	NA
######## No	NA	NA	NA	NA	NA	NA	NA
######## Yes	NA	########	########	########	########	########	NA
######## Yes	NA	NA	NA	NA	########	########	NA
######## Yes	NA	NA	NA	NA	NA	NA	NA
######## Yes	NA	NA	NA	NA	NA	########	NA
######## Yes	NA	NA	########	########	NA	########	NA
######## Yes	NA	NA	NA	NA	NA	########	NA
######## Yes	NA	NA	NA	NA	########	NA	NA
######## Yes	NA	NA	NA	########	NA	########	NA
######## Yes	NA	NA	NA	########	########	########	NA
######## Yes	NA	NA	NA	NA	NA	NA	NA
######## Yes	########	########	########	########	########	########	NA
######## Yes	NA	NA	NA	########	NA	########	NA
######## No	NA	NA	NA	NA	NA	NA	NA
######## Yes	NA	NA	NA	NA	########	NA	NA
######## Yes	########	########	########	NA	NA	########	NA
######## Yes	NA	NA	NA	########	NA	NA	NA
######## Yes	########	########	########	########	########	########	NA
######## Yes	########	########	########	NA	NA	NA	########
######## Yes	NA	NA	NA	NA	########	########	NA
######## Yes	NA	NA	########	########	########	########	NA
######## Yes	NA	NA	NA	NA	NA	########	NA
######## Yes	NA	NA	NA	NA	NA	NA	NA
######## Yes	########	########	########	########	########	########	NA
######## Yes	NA	########	########	NA	NA	NA	NA
######## Yes	NA	NA	NA	NA	NA	########	NA
######## Yes	NA	########	########	########	NA	NA	########
######## No	NA	NA	NA	NA	NA	NA	NA
######## No	NA	NA	NA	NA	NA	NA	NA
######## Yes	########	########	NA	NA	NA	NA	NA
######## Yes	########	########	NA	########	########	########	NA
######## Yes	NA	NA	NA	NA	NA	########	NA
######## Yes	NA	NA	NA	NA	NA	########	NA
######## Yes	########	########	########	########	NA	########	NA
######## Yes	NA	NA	########	########	########	NA	NA
######## Yes	NA	NA	NA	########	NA	########	NA
######## Yes	NA	NA	NA	########	########	########	NA
######## No	NA	NA	NA	NA	NA	NA	NA

For Review Only

######## Yes	########	########	########	########	########	########	NA
######## No	NA	NA	NA	NA	NA	NA	NA
######## Yes	NA	NA	NA	NA	NA	########	NA
######## Yes	NA	NA	NA	NA	########	########	NA
######## Yes	NA	NA	NA	NA	NA	########	NA
######## Yes	NA	NA	########	########	NA	NA	NA
######## Yes	NA	NA	NA	NA	NA	########	NA
######## Yes	NA	NA	NA	NA	NA	########	NA
######## No	NA	NA	NA	NA	NA	NA	NA
######## Yes	########	########	########	########	########	########	NA
######## Yes	########	########	########	NA	########	########	NA
######## Yes	########	########	NA	########	########	########	NA
######## Yes	########	########	########	########	########	########	NA
######## Yes	NA	NA	NA	NA	NA	########	NA
######## Yes	NA	NA	NA	NA	NA	NA	NA
######## Yes	########	########	NA	NA	########	NA	NA
######## No	NA	NA	NA	NA	NA	NA	NA
######## Yes	NA	NA	NA	########	NA	########	NA
######## Yes	NA	NA	NA	NA	########	########	NA
######## Yes	NA	NA	NA	NA	NA	########	NA
######## Yes	########	########	########	########	########	########	NA
######## No	NA	NA	NA	NA	NA	NA	NA
######## Yes	NA	NA	NA	NA	########	NA	NA
######## Yes	NA	NA	NA	########	NA	########	NA
######## Yes	NA	NA	NA	NA	NA	########	NA
######## Yes	NA	NA	########	########	########	NA	NA
######## Yes	NA	NA	NA	NA	NA	########	NA
######## Yes	NA	NA	NA	NA	NA	NA	NA
######## Yes	NA	NA	NA	########	########	NA	NA
######## No	NA	NA	NA	NA	NA	NA	NA
######## Yes	NA	NA	NA	NA	NA	NA	NA
######## Yes	NA	NA	NA	NA	NA	########	NA
######## Yes	NA	NA	NA	NA	########	########	NA
######## Yes	NA	NA	NA	NA	NA	########	NA
######## No	NA	NA	NA	NA	NA	NA	NA
######## Yes	########	NA	NA	NA	NA	NA	NA
######## Yes	NA	NA	########	NA	########	########	NA
######## Yes	NA	NA	NA	NA	NA	########	NA
######## Yes	NA	NA	NA	NA	NA	########	NA
######## No	NA	NA	NA	NA	NA	NA	NA
######## Yes	NA	NA	NA	########	########	########	NA
######## Yes	NA	NA	NA	NA	NA	########	NA
######## Yes	########	########	########	NA	NA	NA	########
######## Yes	NA	NA	NA	########	########	########	NA
######## Yes	NA	NA	NA	########	NA	########	NA
######## Yes	NA	NA	NA	NA	########	########	NA
######## Yes	NA	NA	NA	NA	NA	########	NA
######## Yes	########	########	########	########	########	########	NA
######## Yes	########	########	NA	NA	########	########	NA
######## Yes	NA	NA	NA	NA	NA	########	NA
######## Yes	NA	NA	NA	########	########	NA	NA
######## Yes	NA	NA	NA	NA	NA	########	NA
######## Yes	NA	NA	########	########	########	########	NA
######## Yes	NA	NA	NA	NA	########	########	NA
######## No	NA	NA	NA	NA	NA	NA	NA
######## Yes	NA	NA	NA	NA	NA	########	NA

For Review Only

######## Yes	########	########	########	########	NA	########	NA
######## Yes	NA	NA	NA	NA	########	NA	NA
######## Yes	NA	NA	NA	NA	NA	NA	NA
######## Yes	NA	NA	NA	NA	NA	########	NA
######## Yes	NA	NA	NA	NA	NA	########	NA
######## Yes	NA	########	NA	NA	########	########	NA
######## Yes	########	########	NA	########	########	########	NA
######## Yes	NA	NA	NA	########	NA	########	NA
######## Yes	NA	NA	NA	NA	NA	########	NA
######## Yes	NA	NA	NA	NA	NA	########	NA
######## Yes	NA	NA	NA	########	########	########	NA
######## Yes	NA	NA	NA	########	NA	########	NA
######## Yes	########	########	########	########	########	########	NA
######## Yes	########	########	########	########	########	########	NA
######## Yes	########	########	NA	########	########	########	NA
######## Yes	########	########	NA	########	########	NA	NA
######## Yes	NA	NA	NA	NA	NA	NA	NA
######## Yes	########	########	NA	NA	NA	########	NA
######## Yes	NA	NA	NA	NA	NA	NA	NA
######## Yes	NA	NA	NA	########	########	########	NA
######## Yes	NA	NA	NA	NA	########	NA	NA
######## Yes	NA	NA	NA	NA	NA	NA	NA
######## Yes	NA	NA	NA	NA	########	########	NA
######## Yes	NA	NA	NA	NA	NA	########	NA
######## Yes	########	########	NA	########	########	########	NA
######## Yes	########	########	########	########	########	########	NA
######## Yes	########	########	########	########	########	########	NA
######## Yes	NA	NA	NA	NA	########	########	NA
######## Yes	NA	NA	NA	NA	########	NA	NA
######## Yes	NA	NA	NA	NA	NA	########	NA
######## Yes	NA	NA	NA	NA	NA	NA	NA
######## Yes	NA	NA	NA	########	NA	########	NA
######## No	NA	NA	NA	NA	NA	NA	NA
######## Yes	NA	NA	NA	NA	########	NA	NA
######## No	NA	NA	NA	NA	NA	NA	NA
######## Yes	NA	NA	NA	NA	NA	########	NA
######## Yes	########	########	NA	########	########	########	NA
######## Yes	NA	NA	NA	NA	########	########	NA
######## Yes	NA	NA	########	########	########	########	NA
######## Yes	########	########	NA	########	########	########	NA
######## Yes	NA	NA	NA	NA	NA	########	NA
######## Yes	########	########	########	########	########	########	NA
######## Yes	NA	NA	NA	NA	NA	NA	NA
######## Yes	NA	########	NA	NA	NA	########	NA
######## Yes	NA	NA	NA	NA	NA	########	NA
######## Yes	NA	########	NA	NA	NA	NA	NA
######## Yes	NA	NA	NA	NA	NA	########	NA
######## Yes	NA	NA	NA	########	########	########	NA
######## Yes	NA	NA	NA	########	########	NA	NA
######## Yes	NA	NA	########	########	########	########	NA
######## Yes	NA	NA	NA	NA	NA	NA	NA
######## No	NA	NA	NA	NA	NA	NA	NA
######## Yes	########	########	########	########	########	########	NA
######## Yes	NA	NA	########	########	########	########	NA
######## Yes	NA	########	NA	########	########	########	NA
######## No	NA	NA	NA	NA	NA	NA	NA

For Review Only

######## No	NA	NA	NA	NA	NA	NA	NA
######## No	NA	NA	NA	NA	NA	NA	NA
######## Yes	NA	NA	NA	NA	########	NA	NA
######## No	NA	NA	NA	NA	NA	NA	NA
######## Yes	NA	NA	NA	NA	NA	########	NA
######## No	NA	NA	NA	NA	NA	NA	NA
######## Yes	NA	########	NA	NA	NA	NA	NA
######## Yes	NA	########	NA	NA	NA	NA	NA
######## Yes	NA	########	NA	########	NA	NA	NA
######## Yes	NA	NA	NA	NA	NA	########	NA
######## Yes	NA	NA	NA	NA	NA	NA	NA
######## Yes	NA	NA	NA	NA	NA	########	NA
######## Yes	NA	NA	NA	NA	NA	NA	NA
######## Yes	########	NA	########	########	########	########	########
######## Yes	NA	########	NA	NA	NA	NA	NA
######## Yes	########	########	########	########	########	########	NA
######## No	NA	NA	NA	NA	NA	NA	NA
######## Yes	########	########	########	########	NA	########	NA
######## Yes	NA	NA	NA	NA	NA	########	NA
######## Yes	NA	NA	NA	NA	NA	NA	NA
######## Yes	NA	########	NA	########	########	########	NA
######## Yes	NA	NA	NA	########	########	########	NA
######## Yes	NA	NA	NA	NA	NA	########	NA
######## Yes	NA	NA	NA	NA	NA	NA	NA
######## Yes	NA	NA	NA	NA	NA	NA	NA
######## Yes	NA	NA	NA	NA	NA	NA	NA
######## Yes	NA	NA	NA	NA	NA	NA	NA
######## Yes	NA	########	########	########	########	########	NA
######## Yes	NA	NA	NA	NA	NA	########	NA
######## Yes	########	NA	NA	NA	NA	NA	NA
######## Yes	NA	NA	########	NA	########	########	NA
######## No	NA	NA	NA	NA	NA	NA	NA
######## Yes	NA	########	NA	NA	########	########	NA
######## Yes	NA	NA	NA	NA	########	########	NA
######## Yes	NA	NA	NA	NA	########	########	NA
######## Yes	NA	NA	NA	NA	NA	########	NA
######## Yes	NA	NA	NA	NA	NA	NA	NA
######## No	NA	NA	NA	NA	NA	NA	NA
######## No	NA	NA	NA	NA	NA	NA	NA
######## No	NA	NA	NA	NA	NA	NA	NA
######## Yes	NA	NA	NA	NA	NA	########	NA
######## Yes	NA	NA	NA	NA	NA	NA	NA
######## Yes	NA	########	NA	NA	NA	NA	NA
######## Yes	########	########	########	########	########	########	NA
######## No	NA	NA	NA	NA	NA	NA	NA
######## Yes	NA	NA	NA	NA	NA	NA	NA
######## Yes	NA	########	NA	########	########	########	NA
######## Yes	NA	NA	NA	NA	NA	########	NA
######## Yes	NA	########	NA	NA	########	########	NA
######## Yes	NA	NA	NA	NA	NA	########	NA
######## Yes	NA	NA	NA	NA	########	########	NA
######## Yes	NA	NA	NA	########	########	########	NA
######## No	NA	NA	NA	NA	NA	NA	NA
######## Yes	NA	NA	NA	NA	NA	########	NA
######## Yes	NA	NA	NA	NA	NA	NA	NA
######## No	NA	NA	NA	NA	NA	NA	NA

For Review Only

######## Yes	NA	NA	NA	NA	NA	########	NA
######## Yes	NA	NA	NA	NA	NA	NA	NA
######## Yes	NA	NA	########	########	########	########	NA
######## Yes	########	########	########	########	########	########	NA
######## Yes	NA	NA	NA	NA	NA	########	NA
######## Yes	NA	########	########	########	########	########	NA
######## Yes	NA	NA	########	########	########	NA	NA
######## Yes	NA	NA	NA	NA	NA	########	NA
######## Yes	NA	NA	NA	########	########	########	NA
######## Yes	NA	NA	NA	NA	NA	NA	NA
######## No	NA	NA	NA	NA	NA	NA	NA
######## Yes	########	########	NA	########	########	########	NA
######## Yes	NA	NA	NA	NA	########	########	NA
######## Yes	########	########	NA	########	########	########	########
######## No	NA	NA	NA	NA	NA	NA	NA
######## Yes	########	########	NA	NA	NA	########	NA
######## Yes	NA	NA	NA	NA	NA	########	NA
######## Yes	########	########	########	########	########	########	NA
######## Yes	NA	NA	NA	########	########	########	NA
######## Yes	NA	NA	NA	NA	NA	NA	NA
######## Yes	########	########	########	########	########	########	NA
######## Yes	NA	NA	NA	NA	########	NA	NA
######## Yes	NA	########	NA	NA	NA	NA	NA
######## Yes	NA	NA	NA	NA	NA	########	NA
######## Yes	NA	NA	NA	NA	NA	NA	NA
######## Yes	NA	NA	NA	NA	########	NA	NA
######## Yes	NA	NA	########	NA	NA	NA	NA
######## Yes	NA	NA	NA	NA	NA	########	NA
######## Yes	NA	NA	NA	NA	########	########	NA
######## No	NA	NA	NA	NA	NA	NA	NA
######## Yes	########	########	NA	NA	NA	NA	NA
######## Yes	########	########	NA	NA	########	########	NA
######## No	NA	NA	NA	NA	NA	NA	NA
######## Yes	NA	NA	NA	NA	########	########	NA
######## No	NA	NA	NA	NA	NA	NA	NA
######## Yes	NA	NA	NA	NA	########	########	NA
######## Yes	NA	NA	NA	NA	NA	########	NA
######## Yes	NA	NA	NA	NA	########	########	NA
######## Yes	NA	NA	NA	NA	NA	########	NA
######## Yes	NA	NA	NA	########	########	NA	NA
######## No	NA	NA	NA	NA	NA	NA	NA
######## Yes	NA	########	NA	NA	NA	NA	NA
######## Yes	NA	NA	NA	NA	NA	########	NA
######## Yes	NA	NA	NA	NA	NA	NA	NA
######## Yes	NA	NA	NA	NA	NA	########	NA
######## Yes	NA	NA	NA	NA	NA	########	NA
######## Yes	########	########	########	NA	NA	NA	########
######## Yes	NA	NA	NA	NA	NA	NA	NA
######## Yes	NA	NA	NA	NA	NA	NA	NA
######## Yes	NA	NA	NA	########	########	NA	NA
######## Yes	NA	NA	NA	########	########	########	NA
######## Yes	NA	NA	NA	NA	NA	NA	NA
######## Yes	NA	NA	NA	NA	NA	NA	NA
######## Yes	NA	NA	NA	NA	NA	########	NA
######## Yes	########	########	########	########	########	########	NA
######## Yes	NA	NA	NA	NA	NA	########	NA

For Review Only

########	Yes	########	########	########	########	########	########	NA
########	Yes	NA	NA	NA	NA	########	NA	NA
########	Yes	NA	NA	NA	NA	NA	########	NA
########	Yes	NA	NA	NA	NA	NA	########	NA
########	No	NA	NA	NA	NA	NA	NA	NA
########	Yes	########	########	########	########	########	########	NA
########	Yes	NA	NA	NA	NA	NA	########	NA
########	Yes	########	########	NA	NA	NA	########	NA
########	No	NA	NA	NA	NA	NA	NA	NA
########	Yes	NA	########	NA	NA	NA	NA	NA
########	Yes	NA	NA	NA	########	########	########	NA
########	Yes	NA	NA	NA	NA	NA	########	NA
########	Yes	NA	NA	NA	NA	########	NA	NA
########	Yes	NA	########	NA	NA	NA	NA	NA
########	Yes	NA	NA	NA	########	NA	NA	NA
########	Yes	NA	NA	NA	NA	########	########	NA
########	Yes	NA	NA	NA	NA	NA	NA	NA
########	No	NA	NA	NA	NA	NA	NA	NA
########	Yes	########	########	########	########	########	########	NA
########	Yes	NA	NA	NA	NA	NA	########	NA
########	Yes	NA	NA	NA	NA	NA	NA	NA
########	Yes	NA	NA	NA	NA	NA	########	NA
########	Yes	NA	########	NA	NA	NA	NA	NA
########	Yes	NA	NA	NA	NA	########	########	NA
########	Yes	NA	NA	NA	NA	NA	########	NA
########	Yes	NA	NA	NA	NA	########	########	NA
########	Yes	########	########	########	########	########	########	NA
########	Yes	NA	NA	NA	NA	NA	########	NA
########	Yes	########	########	########	########	########	########	NA
########	Yes	########	########	NA	NA	NA	########	NA
########	No	NA	NA	NA	NA	NA	NA	NA
########	Yes	########	NA	########	########	########	########	NA
########	Yes	NA	NA	NA	########	NA	NA	NA
########	Yes	########	########	NA	########	########	########	NA
########	Yes	NA	NA	NA	NA	NA	########	NA
########	Yes	NA	########	NA	NA	NA	NA	NA
########	Yes	NA	NA	NA	NA	NA	########	NA
########	Yes	NA	NA	NA	NA	NA	NA	NA
########	Yes	########	########	NA	NA	NA	NA	NA
########	No	NA	NA	NA	NA	NA	NA	NA
########	Yes	NA	NA	NA	NA	NA	########	NA
########	Yes	NA	NA	NA	NA	########	########	NA
########	Yes	NA	NA	NA	########	########	########	NA
########	Yes	########	########	########	NA	NA	########	NA
########	Yes	NA	NA	NA	NA	NA	NA	NA
########	No	NA	NA	NA	NA	NA	NA	NA
########	Yes	NA	NA	NA	NA	########	########	NA
########	Yes	NA	NA	NA	NA	NA	NA	NA
########	Yes	NA	NA	NA	########	########	########	NA
########	Yes	########	NA	NA	NA	NA	NA	NA
########	Yes	########	########	########	########	########	########	NA
########	Yes	NA	NA	NA	NA	NA	########	NA
########	Yes	NA	NA	########	########	########	########	NA
########	Yes	NA	NA	NA	########	########	NA	NA
########	Yes	NA	NA	NA	NA	NA	########	NA
########	Yes	########	########	########	########	########	########	NA

For Review Only

######## Yes	NA	########	NA	NA	NA	NA	NA
######## Yes	########	NA	NA	NA	########	NA	NA
######## Yes	NA	########	NA	NA	NA	NA	########
######## Yes	NA	NA	NA	NA	NA	########	NA
######## No	NA	NA	NA	NA	NA	NA	NA
######## Yes	NA	NA	NA	########	########	########	NA
######## Yes	NA	NA	NA	########	########	########	NA
######## Yes	NA	########	NA	NA	########	########	NA
######## Yes	NA	NA	########	########	########	NA	NA
######## Yes	########	NA	NA	NA	########	NA	########
######## Yes	NA	NA	NA	NA	NA	NA	NA
######## No	NA	NA	NA	NA	NA	NA	NA
######## No	NA	NA	NA	NA	NA	NA	NA
######## Yes	NA	########	########	########	########	########	NA
######## Yes	NA	NA	NA	NA	NA	########	NA
######## Yes	########	########	########	########	########	########	NA
######## No	NA	NA	NA	NA	NA	NA	NA
######## Yes	NA	NA	NA	NA	NA	########	NA
######## Yes	NA	NA	NA	NA	########	NA	NA
######## No	NA	NA	NA	NA	NA	NA	NA
######## Yes	NA	NA	########	########	########	########	NA
######## Yes	NA	NA	NA	NA	NA	NA	NA
######## Yes	NA	NA	NA	NA	NA	NA	NA
######## Yes	NA	########	NA	NA	NA	########	NA
######## Yes	NA	NA	########	########	########	NA	NA
######## Yes	NA	NA	NA	NA	NA	NA	NA
######## Yes	NA	NA	########	########	########	########	NA
######## Yes	NA	NA	########	########	NA	########	NA
######## Yes	########	NA	NA	########	########	########	NA
######## Yes	NA	NA	NA	NA	NA	########	NA
######## Yes	NA	NA	NA	NA	NA	NA	NA
######## Yes	########	NA	NA	NA	########	########	NA
######## Yes	NA	NA	NA	NA	NA	########	NA
######## Yes	NA	NA	########	########	########	########	NA
######## Yes	NA	NA	NA	NA	########	NA	NA
######## Yes	NA	NA	NA	NA	NA	########	NA
######## No	NA	NA	NA	NA	NA	NA	NA
######## Yes	NA	NA	NA	NA	NA	NA	NA
######## Yes	NA	NA	NA	NA	########	NA	NA
######## Yes	NA	NA	########	########	########	########	NA
######## Yes	########	########	########	NA	NA	NA	NA
######## Yes	NA	NA	NA	NA	########	########	NA
######## Yes	NA	NA	NA	NA	NA	########	NA
######## Yes	NA	NA	NA	NA	NA	########	NA
######## Yes	########	########	########	NA	NA	########	NA
######## Yes	########	########	########	########	########	########	NA
######## Yes	NA	########	NA	NA	########	########	NA
######## Yes	########	########	########	########	########	########	NA
######## Yes	NA	NA	NA	NA	NA	########	NA
######## Yes	########	########	########	########	########	########	NA
######## Yes	NA	NA	NA	NA	NA	########	NA
######## Yes	NA	NA	########	NA	NA	########	NA

For Review Only

########	Yes	NA	NA	NA	NA	########	########	NA
########	Yes	NA	NA	NA	NA	NA	########	NA
########	Yes	NA	NA	NA	NA	NA	########	NA
########	Yes	NA	NA	########	########	NA	NA	NA
########	Yes	NA	NA	########	########	########	########	NA
########	Yes	NA	NA	########	########	########	########	NA
########	Yes	NA	NA	NA	NA	########	########	NA
########	Yes	########	NA	NA	NA	NA	NA	NA
########	Yes	NA	NA	NA	NA	NA	NA	NA
########	Yes	NA	NA	NA	NA	NA	########	NA
########	No	NA	NA	NA	NA	NA	NA	NA
########	No	NA	NA	NA	NA	NA	NA	NA
########	Yes	NA	NA	NA	NA	########	########	NA
########	No	NA	NA	NA	NA	NA	NA	NA
########	Yes	NA	########	NA	NA	NA	NA	NA
########	Yes	NA	NA	NA	NA	NA	NA	NA
########	Yes	NA	NA	NA	NA	NA	########	NA
########	Yes	NA	NA	NA	NA	NA	########	NA
########	No	NA	NA	NA	NA	NA	NA	NA
########	No	NA	NA	NA	NA	NA	NA	NA
########	No	NA	NA	NA	NA	NA	NA	NA
########	No	NA	NA	NA	NA	NA	NA	NA
########	Yes	NA	########	########	########	########	########	NA
########	Yes	NA	NA	NA	NA	########	########	NA
########	Yes	NA	NA	NA	NA	NA	########	NA
########	Yes	NA	NA	NA	NA	NA	NA	NA
########	Yes	########	########	########	########	NA	########	NA
########	Yes	NA	NA	NA	NA	########	NA	NA
########	Yes	NA	NA	NA	NA	NA	########	NA
########	Yes	NA	NA	NA	NA	NA	########	NA
########	Yes	NA	########	NA	NA	NA	NA	NA
########	Yes	NA	NA	NA	NA	########	########	NA
########	Yes	NA	NA	NA	NA	NA	NA	NA
########	Yes	NA	NA	########	########	NA	########	NA
########	Yes	NA	NA	NA	NA	NA	########	NA
########	Yes	NA	NA	NA	NA	NA	NA	NA
########	No	NA	NA	NA	NA	NA	NA	NA
########	Yes	NA	NA	NA	NA	NA	NA	NA
########	Yes	NA	NA	NA	NA	NA	########	NA
########	Yes	NA	NA	NA	NA	NA	NA	NA
########	Yes	NA	NA	NA	NA	########	########	NA
########	Yes	NA	NA	NA	NA	NA	########	NA
########	Yes	NA	NA	########	NA	NA	########	NA
########	Yes	NA	NA	NA	NA	NA	########	NA
########	Yes	NA	NA	NA	NA	########	########	NA
########	Yes	NA	########	########	NA	NA	########	NA
########	Yes	NA	NA	NA	NA	########	NA	NA
########	Yes	NA	NA	NA	NA	########	NA	NA
########	Yes	NA	NA	NA	NA	NA	NA	NA
########	Yes	NA	NA	NA	########	########	########	NA
########	Yes	NA	NA	NA	########	NA	NA	NA

For Review Only

########	Yes	NA	########	########	########	NA	########	NA
########	No	NA	NA	NA	NA	NA	NA	NA
########	No	NA	NA	NA	NA	NA	NA	NA
########	Yes	NA	NA	NA	NA	NA	########	NA
########	Yes	NA	NA	NA	NA	NA	NA	NA
########	Yes	NA	NA	########	NA	NA	NA	NA
########	Yes	NA	########	NA	NA	NA	NA	########
########	Yes	NA	NA	NA	########	NA	NA	NA
########	Yes	NA	NA	NA	NA	NA	NA	NA
########	Yes	NA	NA	NA	NA	NA	NA	NA
########	Yes	NA	NA	########	########	NA	NA	NA
########	Yes	NA	NA	NA	NA	NA	NA	NA
########	Yes	NA	NA	########	NA	########	########	NA
########	Yes	NA	########	NA	NA	NA	NA	########
########	Yes	NA	########	########	########	########	########	NA
########	Yes	NA	NA	NA	NA	NA	NA	NA
########	Yes	########	########	NA	NA	NA	NA	NA
########	Yes	########	########	########	########	########	########	NA
########	Yes	NA	NA	NA	########	########	########	NA
########	Yes	########	NA	NA	NA	NA	NA	NA
########	Yes	NA	NA	NA	NA	NA	########	NA
########	Yes	NA	NA	NA	NA	NA	NA	NA
########	No	NA	NA	NA	NA	NA	NA	NA
########	No	NA	NA	NA	NA	NA	NA	NA
########	Yes	NA	NA	NA	NA	NA	NA	NA
########	No	NA	NA	NA	NA	NA	NA	NA
########	Yes	NA	NA	NA	NA	########	NA	NA
########	Yes	NA	NA	NA	NA	NA	NA	NA
########	No	NA	NA	NA	NA	NA	NA	NA
########	No	NA	NA	NA	NA	NA	NA	NA
########	Yes	########	########	NA	########	########	########	NA
########	Yes	NA	NA	NA	NA	NA	NA	NA
########	No	NA	NA	NA	NA	NA	NA	NA
########	Yes	NA	NA	NA	NA	NA	########	NA
########	Yes	NA	NA	NA	NA	NA	########	NA
########	Yes	NA	NA	NA	NA	########	NA	NA
########	Yes	########	########	########	########	########	########	NA
########	Yes	NA	NA	NA	NA	########	########	NA
########	Yes	NA	NA	NA	NA	NA	########	NA
########	Yes	NA	NA	NA	NA	NA	NA	NA
########	Yes	NA	NA	NA	NA	NA	NA	NA
########	Yes	########	########	########	NA	NA	NA	########
########	Yes	NA	NA	NA	NA	NA	########	NA
########	Yes	NA	NA	NA	NA	NA	########	NA
########	Yes	NA	NA	NA	NA	NA	########	NA
########	Yes	NA	NA	NA	NA	NA	########	NA
########	Yes	NA	NA	NA	NA	NA	########	NA
########	Yes	NA	NA	NA	########	NA	NA	NA
########	Yes	NA	NA	NA	NA	NA	########	NA
########	Yes	NA	NA	NA	NA	NA	NA	NA
########	Yes	NA	NA	NA	NA	########	########	NA
########	Yes	########	########	########	########	########	########	NA
########	Yes	NA	NA	NA	NA	########	NA	NA
########	No	NA	NA	NA	NA	NA	NA	NA
########	Yes	NA	########	NA	NA	NA	NA	NA
########	No	NA	NA	NA	NA	NA	NA	NA

For Review Only

########	Yes	NA	NA	NA	NA	########	########	NA
########	Yes	NA	NA	NA	########	########	########	NA
########	Yes	NA	NA	NA	NA	NA	NA	NA
########	Yes	########	NA	NA	########	########	########	NA
########	Yes	NA	NA	NA	NA	NA	NA	NA
########	Yes	NA	NA	NA	NA	########	########	NA
########	Yes	########	########	########	########	########	########	NA
########	Yes	########	########	########	########	########	########	NA
########	No	NA	NA	NA	NA	NA	NA	NA
########	Yes	NA	NA	NA	NA	########	NA	NA
########	No	NA	NA	NA	NA	NA	NA	NA
########	Yes	NA	NA	NA	NA	NA	NA	NA
########	Yes	NA	NA	NA	NA	########	########	NA
########	Yes	NA	NA	NA	NA	########	NA	NA
########	Yes	########	########	########	########	########	########	NA
########	Yes	NA	NA	NA	########	########	########	NA
########	Yes	NA	NA	NA	NA	########	########	NA
########	Yes	NA	NA	NA	NA	########	########	NA
########	Yes	NA	NA	NA	NA	NA	NA	NA
########	No	NA	NA	NA	NA	NA	NA	NA
########	No	NA	NA	NA	NA	NA	NA	NA
########	No	NA	NA	NA	NA	NA	NA	NA
########	Yes	NA	NA	NA	NA	NA	########	NA
########	Yes	########	NA	NA	########	########	########	NA
########	Yes	NA	NA	NA	NA	NA	########	NA
########	Yes	########	########	########	########	NA	########	NA
########	Yes	NA	NA	########	NA	NA	NA	NA
########	No	NA	NA	NA	NA	NA	NA	NA
########	Yes	########	########	########	########	NA	########	NA
########	No	NA	NA	NA	NA	NA	NA	NA
########	No	NA	NA	NA	NA	NA	NA	NA
########	No	NA	NA	NA	NA	NA	NA	NA
########	Yes	NA	NA	########	########	########	NA	NA
########	Yes	NA	NA	NA	NA	NA	NA	NA
########	Yes	NA	NA	NA	NA	########	########	NA
########	Yes	NA	########	########	########	########	########	NA
########	No	NA	NA	NA	NA	NA	NA	NA
########	Yes	NA	NA	NA	NA	########	########	NA
########	Yes	NA	NA	NA	NA	NA	NA	NA
########	Yes	NA	NA	NA	########	########	NA	NA
########	Yes	NA	NA	NA	NA	NA	########	NA
########	Yes	NA	NA	NA	NA	########	########	NA
########	No	NA	NA	NA	NA	NA	NA	NA
########	Yes	NA	NA	NA	########	########	########	NA
########	Yes	NA	NA	########	NA	NA	########	NA
########	Yes	########	########	########	########	########	########	NA
########	Yes	NA	NA	NA	NA	NA	########	NA
########	Yes	NA	NA	########	########	NA	NA	NA
########	Yes	NA	NA	NA	NA	NA	########	NA
########	Yes	NA	NA	NA	NA	NA	########	NA
########	No	NA	NA	NA	NA	NA	NA	NA
########	Yes	NA	NA	NA	NA	NA	########	NA
########	Yes	########	########	NA	########	########	########	NA
########	No	NA	NA	NA	NA	NA	NA	NA
########	Yes	NA	NA	NA	NA	NA	NA	NA
########	Yes	NA	NA	NA	########	########	NA	NA

For Review Only

######## Yes	NA	NA	NA	NA	########	########	NA
######## Yes	NA	NA	NA	NA	NA	########	NA
######## Yes	NA	NA	NA	NA	NA	########	NA
######## Yes	NA	NA	NA	NA	NA	########	NA
######## Yes	NA	NA	########	########	########	########	NA
######## Yes	########	########	########	########	########	########	NA
######## Yes	NA	########	########	########	########	########	NA
######## No	NA	NA	NA	NA	NA	NA	NA
######## Yes	########	NA	########	########	########	########	NA
######## Yes	########	########	########	########	########	########	NA
######## Yes	NA	NA	NA	########	NA	NA	NA
######## Yes	NA	NA	NA	NA	NA	########	NA
######## Yes	NA	NA	NA	NA	NA	########	NA
######## Yes	NA	NA	NA	NA	NA	NA	NA
######## Yes	NA	NA	NA	NA	NA	NA	NA
######## Yes	NA	NA	NA	NA	NA	########	NA
######## Yes	NA	NA	NA	NA	NA	########	NA
######## No	NA	NA	NA	NA	NA	NA	NA
######## Yes	########	########	########	########	########	########	NA
######## Yes	NA	NA	########	########	########	NA	NA
######## No	NA	NA	NA	NA	NA	NA	NA
######## Yes	NA	NA	NA	NA	########	########	NA
######## Yes	NA	NA	NA	########	########	NA	NA
######## Yes	NA	NA	NA	NA	NA	########	NA
######## Yes	NA	NA	NA	NA	########	########	NA
######## Yes	########	########	########	########	########	########	NA
######## No	NA	NA	NA	NA	NA	NA	NA
######## Yes	NA	NA	########	NA	########	NA	NA
######## Yes	NA	NA	NA	NA	NA	########	NA
######## Yes	NA	NA	NA	NA	########	########	NA
######## No	NA	NA	NA	NA	NA	NA	NA
######## Yes	NA	NA	NA	########	NA	NA	NA
######## Yes	NA	NA	########	########	########	########	NA
######## Yes	NA	NA	NA	########	########	NA	NA
######## Yes	NA	########	NA	NA	NA	NA	NA
######## Yes	NA	NA	########	NA	########	NA	NA
######## Yes	NA	########	NA	NA	NA	NA	NA
######## Yes	NA	########	########	NA	NA	NA	########
######## Yes	NA	NA	NA	NA	NA	########	NA
######## Yes	########	########	########	NA	########	########	########
######## Yes	NA	NA	########	NA	NA	########	NA
######## Yes	NA	NA	NA	########	########	########	NA
######## Yes	########	########	NA	########	########	NA	NA
######## Yes	NA	NA	NA	########	########	########	NA
######## Yes	NA	NA	NA	NA	NA	########	NA
######## Yes	NA	NA	NA	########	########	########	NA
######## Yes	########	NA	NA	NA	########	########	NA
######## No	NA	NA	NA	NA	NA	NA	NA
######## No	NA	NA	NA	NA	NA	NA	NA
######## Yes	NA	NA	########	########	########	NA	NA
######## Yes	NA	NA	NA	NA	NA	########	NA
######## Yes	NA	NA	NA	NA	########	########	NA
######## Yes	NA	NA	NA	NA	NA	########	NA
######## Yes	NA	########	NA	NA	NA	NA	NA
######## Yes	NA	NA	NA	NA	NA	########	NA
######## Yes	NA	########	NA	NA	NA	NA	########

For Review Only

######## Yes	NA	NA	NA	NA	NA	########	NA
######## Yes	NA	NA	NA	NA	########	########	NA
######## Yes	NA	NA	NA	NA	########	NA	NA
######## Yes	########	########	########	NA	########	########	NA
######## Yes	NA	NA	NA	NA	########	########	NA
######## Yes	NA	NA	NA	########	########	NA	NA
######## Yes	NA	NA	NA	NA	NA	NA	NA
######## No	NA	NA	NA	NA	NA	NA	NA
######## Yes	NA	NA	NA	########	########	########	NA
######## Yes	NA	########	NA	NA	NA	NA	NA
######## Yes	NA	NA	NA	NA	NA	########	NA
######## Yes	NA	NA	NA	########	########	NA	NA
######## Yes	NA	NA	NA	NA	NA	NA	NA
######## Yes	NA	NA	NA	NA	NA	########	NA
######## No	NA	NA	NA	NA	NA	NA	NA
######## Yes	NA	NA	NA	NA	NA	NA	NA
######## Yes	NA	NA	NA	NA	NA	########	NA
######## No	NA	NA	NA	NA	NA	NA	NA
######## Yes	NA	NA	NA	NA	NA	NA	NA
######## Yes	########	########	NA	########	########	########	NA
######## Yes	NA	########	NA	NA	NA	NA	NA
######## Yes	NA	NA	NA	NA	########	########	NA
######## Yes	NA	NA	NA	NA	NA	NA	NA
######## Yes	NA	NA	NA	NA	NA	########	NA
######## No	NA	NA	NA	NA	NA	NA	NA
######## Yes	NA	NA	NA	NA	NA	NA	NA
######## Yes	NA	NA	########	NA	########	########	NA
######## No	NA	NA	NA	NA	NA	NA	NA
######## Yes	NA	NA	NA	NA	NA	########	NA
######## Yes	NA	########	########	########	########	########	NA
######## No	NA	NA	NA	NA	NA	NA	NA
######## Yes	NA	NA	NA	NA	NA	########	NA
######## Yes	NA	NA	NA	NA	NA	NA	NA
######## Yes	NA	NA	NA	########	NA	########	NA
######## Yes	NA	NA	NA	NA	NA	########	NA
######## Yes	NA	########	NA	NA	NA	########	NA
######## No	NA	NA	NA	NA	NA	NA	NA
######## Yes	NA	NA	########	########	NA	NA	NA
######## Yes	NA	NA	NA	NA	########	########	NA
######## Yes	NA	NA	NA	NA	########	########	NA
######## Yes	NA	NA	########	########	########	########	NA
######## Yes	NA	NA	NA	NA	########	NA	NA
######## Yes	########	########	NA	########	########	########	NA
######## Yes	NA	NA	NA	NA	########	########	NA
######## Yes	NA	NA	########	NA	NA	########	NA
######## Yes	NA	NA	NA	NA	NA	########	NA
######## Yes	########	########	NA	NA	NA	NA	NA
######## Yes	NA	########	NA	NA	NA	NA	########
######## Yes	NA	NA	NA	NA	NA	NA	NA
######## Yes	NA	NA	NA	NA	NA	########	NA
######## Yes	NA	NA	NA	########	########	########	NA
######## Yes	NA	NA	NA	NA	NA	NA	NA
######## Yes	NA	NA	NA	NA	########	########	NA
######## Yes	NA	NA	NA	NA	########	NA	NA
######## No	NA	NA	NA	NA	NA	NA	NA

For Review Only

######## Yes	NA	NA	NA	NA	NA	NA	NA
######## No	NA	NA	NA	NA	NA	NA	NA
######## Yes	NA	NA	NA	NA	########	NA	NA
######## Yes	########	NA	########	########	########	########	NA
######## Yes	NA	NA	NA	NA	NA	########	NA
######## Yes	NA	NA	NA	########	########	########	NA
######## Yes	NA	NA	NA	NA	NA	########	NA
######## Yes	NA	NA	NA	NA	NA	########	NA
######## No	NA	NA	NA	NA	NA	NA	NA
######## Yes	########	########	########	########	########	########	NA
######## Yes	NA	NA	########	########	########	########	NA
######## Yes	NA	NA	NA	NA	NA	########	NA
######## Yes	NA	NA	NA	NA	NA	########	NA
######## Yes	NA	NA	NA	NA	NA	NA	NA
######## Yes	NA	NA	########	########	########	########	NA
######## Yes	########	########	########	########	########	########	NA
######## Yes	NA	########	########	########	########	########	NA
######## Yes	NA	NA	NA	NA	NA	########	NA
######## No	NA	NA	NA	NA	NA	NA	NA
######## Yes	NA	NA	NA	NA	NA	NA	NA
######## Yes	NA	NA	NA	NA	NA	########	NA
######## Yes	########	NA	########	########	########	########	########
######## Yes	NA	NA	NA	NA	NA	########	NA
######## Yes	NA	NA	NA	NA	NA	########	NA
######## Yes	NA	NA	NA	NA	NA	NA	NA
######## Yes	NA	NA	NA	NA	NA	NA	NA
######## No	NA	NA	NA	NA	NA	NA	NA
######## Yes	NA	NA	NA	########	NA	NA	NA
######## No	NA	NA	NA	NA	NA	NA	NA
######## Yes	NA	NA	NA	########	NA	########	NA
######## Yes	########	########	NA	NA	NA	NA	NA
######## Yes	NA	########	NA	NA	NA	NA	########
######## Yes	########	########	########	########	########	########	NA
######## Yes	########	########	########	########	########	########	NA
######## No	NA	NA	NA	NA	NA	NA	NA
######## Yes	NA	NA	NA	NA	NA	########	NA
######## No	NA	NA	NA	NA	NA	NA	NA
######## Yes	NA	NA	NA	NA	NA	NA	NA
######## Yes	########	NA	NA	NA	########	########	########
######## Yes	NA	NA	NA	NA	########	########	NA
######## Yes	NA	NA	NA	NA	NA	########	NA
######## Yes	NA	NA	NA	########	########	########	NA
######## No	NA	NA	NA	NA	NA	NA	NA
######## Yes	########	########	########	NA	NA	########	NA
######## Yes	NA	NA	NA	########	########	########	NA
######## Yes	NA	NA	NA	NA	NA	########	NA
######## Yes	NA	NA	NA	NA	NA	########	NA
######## Yes	NA	NA	NA	########	########	########	NA
######## Yes	NA	NA	NA	NA	NA	########	NA
######## Yes	########	########	########	########	NA	########	NA
######## Yes	NA	NA	NA	NA	########	########	NA
######## Yes	NA	NA	########	########	########	########	NA
######## Yes	NA	NA	NA	########	########	########	NA
######## Yes	NA	NA	########	NA	NA	NA	NA
######## Yes	NA	NA	NA	########	########	NA	NA
######## Yes	########	NA	NA	########	########	########	NA

For Review Only

######## Yes	NA	NA	NA	NA	########	NA	NA
######## No	NA	NA	NA	NA	NA	NA	NA
######## Yes	NA	NA	NA	########	########	########	NA
######## Yes	NA	NA	NA	########	########	########	NA
######## Yes	NA	NA	NA	NA	########	NA	NA
######## Yes	NA	NA	NA	NA	NA	########	NA
######## Yes	########	########	########	########	########	########	NA
######## No	NA	NA	NA	NA	NA	NA	NA
######## Yes	########	########	NA	########	########	########	NA
######## Yes	NA	NA	NA	NA	########	NA	NA
######## Yes	NA	########	########	NA	NA	NA	NA
######## Yes	NA	NA	NA	NA	NA	########	NA
######## No	NA	NA	NA	NA	NA	NA	NA
######## Yes	NA	NA	NA	NA	NA	NA	NA
######## Yes	########	########	NA	########	########	NA	NA
######## Yes	NA	NA	NA	NA	NA	########	NA
######## Yes	NA	NA	NA	NA	########	NA	NA
######## Yes	NA	NA	NA	NA	NA	########	NA
######## No	NA	NA	NA	NA	NA	NA	NA
######## Yes	NA	NA	NA	########	NA	########	NA
######## Yes	NA	########	NA	NA	NA	NA	NA
######## No	NA	NA	NA	NA	NA	NA	NA
######## Yes	NA	NA	NA	NA	NA	NA	NA
######## Yes	########	########	NA	########	########	########	NA
######## Yes	NA	NA	########	NA	NA	NA	NA
######## Yes	########	NA	NA	NA	NA	NA	NA
######## No	NA	NA	NA	NA	NA	NA	NA
######## No	NA	NA	NA	NA	NA	NA	NA
######## Yes	NA	NA	NA	NA	########	########	NA
######## Yes	NA	NA	NA	NA	NA	########	NA
######## Yes	########	########	########	########	########	########	NA
######## No	NA	NA	NA	NA	NA	NA	NA
######## No	NA	NA	NA	NA	NA	NA	NA
######## Yes	NA	NA	NA	NA	NA	NA	NA
######## Yes	NA	NA	NA	NA	NA	########	NA
######## No	NA	NA	NA	NA	NA	NA	NA
######## Yes	NA	NA	NA	########	########	########	NA
######## Yes	########	########	NA	########	NA	########	NA
######## Yes	NA	NA	NA	NA	NA	NA	NA
######## Yes	########	########	NA	########	########	########	NA
######## Yes	NA	NA	NA	NA	NA	NA	NA
######## Yes	NA	NA	NA	NA	########	########	NA
######## No	NA	NA	NA	NA	NA	NA	NA
######## Yes	NA	NA	NA	NA	########	NA	NA
######## Yes	NA	NA	NA	NA	NA	NA	NA
######## Yes	NA	NA	NA	NA	NA	########	NA
######## Yes	NA	NA	NA	NA	NA	########	NA
######## No	NA	NA	NA	NA	NA	NA	NA
######## Yes	NA	NA	NA	NA	########	NA	NA
######## Yes	NA	NA	NA	NA	NA	########	NA
######## Yes	NA	NA	NA	NA	NA	########	NA
######## Yes	NA	NA	NA	NA	NA	########	NA
######## No	NA	NA	NA	NA	NA	NA	NA
######## Yes	NA	########	NA	########	########	NA	NA
######## Yes	NA	NA	########	########	########	########	NA
######## Yes	NA	NA	########	########	NA	########	NA
######## Yes	NA	NA	NA	NA	########	NA	NA

For Review Only

######## No	NA	NA	NA	NA	NA	NA	NA
######## Yes	########	########	NA	NA	########	NA	NA
######## Yes	NA	NA	NA	NA	NA	########	NA
######## Yes	########	########	NA	NA	########	########	NA
######## No	NA	NA	NA	NA	NA	NA	NA
######## Yes	NA	NA	NA	NA	########	########	NA
######## Yes	NA	NA	NA	NA	NA	NA	NA
######## Yes	NA	NA	NA	########	NA	NA	NA
######## Yes	NA	NA	NA	NA	########	########	NA
######## Yes	NA	NA	NA	########	NA	NA	NA
######## Yes	########	########	########	########	########	########	NA
######## Yes	NA	NA	NA	NA	########	########	NA
######## Yes	NA	NA	NA	NA	NA	########	NA
######## No	NA	NA	NA	NA	NA	NA	NA
######## Yes	########	########	NA	########	########	########	NA
######## Yes	NA	NA	NA	########	########	########	NA
######## Yes	NA	NA	NA	NA	NA	NA	NA
######## No	NA	NA	NA	NA	NA	NA	NA
######## Yes	########	NA	NA	NA	NA	NA	NA
######## No	NA	NA	NA	NA	NA	NA	NA
######## Yes	NA	NA	NA	NA	NA	########	NA
######## Yes	NA	NA	NA	NA	NA	########	NA
######## Yes	NA	NA	NA	NA	NA	########	NA
######## No	NA	NA	NA	NA	NA	NA	NA
######## Yes	########	########	NA	########	########	########	NA
######## Yes	NA	NA	NA	########	NA	########	NA
######## Yes	########	########	NA	########	########	########	NA
######## Yes	NA	NA	NA	NA	NA	NA	NA
######## No	NA	NA	NA	NA	NA	NA	NA
######## Yes	NA	NA	########	########	########	########	NA
######## Yes	NA	NA	NA	NA	########	NA	NA
######## Yes	########	########	NA	NA	NA	########	NA
######## No	NA	NA	NA	NA	NA	NA	NA
######## Yes	NA	NA	NA	NA	########	NA	NA
######## Yes	NA	NA	NA	NA	NA	########	NA
######## No	NA	NA	NA	NA	NA	NA	NA
######## No	NA	NA	NA	NA	NA	NA	NA
######## No	NA	NA	NA	NA	NA	NA	NA
######## Yes	NA	########	NA	NA	NA	NA	NA
######## Yes	NA	NA	NA	NA	########	########	NA
######## Yes	NA	NA	NA	NA	NA	########	NA
######## No	NA	NA	NA	NA	NA	NA	NA
######## Yes	########	NA	########	########	########	########	########
######## Yes	########	########	NA	NA	NA	NA	NA
######## Yes	NA	NA	NA	########	########	########	NA
######## Yes	NA	NA	########	NA	NA	NA	NA
######## Yes	NA	NA	NA	NA	NA	NA	NA
######## Yes	NA	NA	NA	NA	NA	NA	NA
######## Yes	NA	NA	NA	NA	########	########	NA
######## Yes	NA	NA	NA	NA	########	########	NA
######## Yes	########	########	NA	NA	NA	NA	NA
######## No	NA	NA	NA	NA	NA	NA	NA
######## No	NA	NA	NA	NA	NA	NA	NA
######## No	NA	NA	NA	NA	NA	NA	NA
######## Yes	NA	NA	NA	NA	NA	########	NA
######## Yes	NA	NA	NA	NA	NA	########	NA

For Review Only

######## Yes	NA	NA	########	NA	NA	NA	NA
######## Yes	NA	########	NA	########	NA	########	NA
######## Yes	NA	NA	NA	NA	########	NA	NA
######## Yes	NA	NA	NA	NA	NA	NA	NA
######## Yes	NA	NA	########	########	########	########	NA
######## Yes	########	########	########	########	########	########	NA
######## No	NA	NA	NA	NA	NA	NA	NA
######## Yes	NA	NA	NA	NA	NA	########	NA
######## Yes	NA	NA	NA	NA	NA	########	NA
######## Yes	NA	NA	NA	NA	########	NA	NA
######## Yes	########	########	NA	NA	########	########	NA
######## Yes	########	########	NA	NA	NA	NA	NA
######## Yes	NA	NA	NA	########	NA	NA	NA
######## Yes	NA	NA	NA	NA	NA	NA	NA
######## Yes	NA	NA	NA	NA	NA	########	NA
######## Yes	NA	NA	NA	NA	########	NA	NA
######## Yes	NA	NA	NA	NA	########	########	NA
######## Yes	########	########	########	########	########	########	NA
######## Yes	NA	NA	NA	########	########	########	NA
######## Yes	NA	NA	########	########	########	########	NA
######## Yes	########	########	NA	########	########	########	NA
######## Yes	NA	NA	NA	NA	NA	########	NA
######## Yes	NA	########	NA	NA	NA	NA	NA
######## Yes	NA	NA	########	NA	NA	########	NA
######## Yes	NA	NA	NA	NA	NA	########	NA
######## Yes	NA	NA	NA	NA	NA	########	NA
######## Yes	NA	NA	NA	########	########	########	NA
######## Yes	NA	NA	NA	NA	NA	NA	NA
######## Yes	NA	NA	NA	NA	NA	########	NA
######## Yes	NA	NA	NA	NA	NA	########	NA
######## Yes	NA	NA	NA	NA	NA	########	NA
######## Yes	NA	NA	NA	NA	NA	########	NA
######## Yes	NA	NA	NA	NA	NA	########	NA
######## Yes	NA	NA	NA	NA	NA	########	NA
######## Yes	NA	NA	NA	NA	NA	NA	NA
######## Yes	NA	NA	NA	NA	NA	NA	NA
######## Yes	NA	########	########	NA	NA	NA	NA
######## Yes	NA	NA	NA	NA	NA	########	NA
######## Yes	########	NA	NA	NA	NA	NA	NA
######## Yes	NA	NA	NA	NA	########	########	NA
######## Yes	NA	NA	NA	NA	NA	NA	NA
######## Yes	########	########	########	########	########	########	NA
######## Yes	NA	NA	########	NA	########	########	NA
######## Yes	NA	NA	NA	NA	########	########	NA
######## Yes	NA	NA	NA	NA	NA	########	NA
######## No	NA	NA	NA	NA	NA	NA	NA
######## Yes	NA	NA	NA	NA	NA	########	NA
######## Yes	NA	NA	########	NA	NA	NA	NA
######## Yes	########	########	NA	########	########	########	NA
######## Yes	NA	NA	NA	NA	NA	########	NA
######## No	NA	NA	NA	NA	NA	NA	NA
######## Yes	NA	NA	NA	NA	########	########	NA
######## Yes	NA	NA	NA	NA	########	NA	NA
######## Yes	NA	NA	########	########	########	########	NA
######## Yes	NA	NA	NA	NA	NA	NA	NA
######## Yes	NA	NA	NA	NA	NA	########	NA

For Review Only

######## Yes	NA	NA	NA	NA	NA	########	NA
######## Yes	NA	NA	NA	########	########	NA	NA
######## Yes	NA	NA	NA	NA	NA	########	NA
######## Yes	NA	NA	NA	########	########	########	NA
######## Yes	NA	NA	NA	NA	NA	########	NA
######## Yes	NA	NA	NA	NA	NA	NA	NA
######## No	NA	NA	NA	NA	NA	NA	NA
######## Yes	NA	########	NA	NA	NA	NA	NA
######## Yes	########	########	NA	########	########	########	NA
######## Yes	NA	NA	NA	NA	########	NA	NA
######## Yes	NA	NA	NA	NA	NA	########	NA
######## Yes	NA	NA	NA	NA	NA	########	NA
######## Yes	NA	NA	NA	########	########	########	NA
######## Yes	NA	NA	NA	NA	NA	########	NA
######## Yes	NA	########	NA	########	########	########	NA
######## Yes	NA	NA	NA	NA	NA	########	NA
######## Yes	NA	NA	########	########	########	NA	NA
######## Yes	NA	########	NA	NA	NA	NA	NA
######## No	NA	NA	NA	NA	NA	NA	NA
######## Yes	NA	NA	NA	NA	NA	NA	NA
######## Yes	NA	########	NA	NA	NA	########	NA
######## Yes	NA	NA	NA	NA	########	########	NA
######## No	NA	NA	NA	NA	NA	NA	NA
######## Yes	########	NA	NA	########	########	########	NA
######## Yes	########	########	NA	NA	NA	########	NA
######## Yes	NA	NA	########	########	########	NA	NA
######## No	NA	NA	NA	NA	NA	NA	NA
######## Yes	########	########	NA	########	########	########	NA
######## Yes	NA	NA	NA	NA	NA	########	NA
######## Yes	NA	NA	NA	########	########	########	NA
######## Yes	########	########	NA	########	########	########	NA
######## Yes	NA	NA	NA	NA	NA	NA	NA
######## No	NA	NA	NA	NA	NA	NA	NA
######## Yes	NA	NA	NA	NA	NA	NA	NA
######## Yes	NA	NA	NA	NA	NA	NA	NA
######## Yes	NA	NA	########	NA	NA	NA	NA
######## Yes	NA	NA	NA	NA	NA	########	NA
######## Yes	NA	NA	NA	NA	NA	########	NA
######## Yes	NA	NA	NA	NA	NA	NA	NA
######## Yes	########	NA	NA	NA	NA	NA	NA
######## No	NA	NA	NA	NA	NA	NA	NA
######## Yes	NA	NA	NA	NA	NA	########	NA
######## Yes	NA	NA	########	########	########	NA	NA
######## Yes	########	########	NA	########	########	########	NA
######## No	NA	NA	NA	NA	NA	NA	NA
######## Yes	NA	NA	########	########	NA	NA	NA
######## Yes	NA	NA	NA	NA	NA	########	NA
######## No	NA	NA	NA	NA	NA	NA	NA
######## Yes	NA	NA	########	NA	NA	NA	NA
######## Yes	########	########	########	########	########	########	NA
######## No	NA	NA	NA	NA	NA	NA	NA
######## Yes	NA	NA	NA	########	########	########	NA
######## No	NA	NA	NA	NA	NA	NA	NA
######## Yes	########	########	NA	NA	NA	########	NA
######## Yes	NA	NA	NA	########	########	########	NA
######## Yes	NA	NA	NA	NA	NA	########	NA

For Review Only

######## Yes	NA	NA	NA	NA	NA	NA	NA
######## Yes	NA	NA	NA	NA	NA	########	NA
######## Yes	NA	NA	NA	NA	NA	########	NA
######## Yes	NA	NA	########	########	########	NA	NA
######## Yes	########	NA	NA	NA	NA	NA	NA
######## Yes	########	########	NA	NA	NA	NA	NA
######## No	NA	NA	NA	NA	NA	NA	NA
######## Yes	NA	NA	NA	NA	NA	########	NA
######## Yes	########	########	NA	NA	NA	NA	NA
######## Yes	NA	NA	NA	NA	NA	########	NA
######## Yes	NA	NA	NA	NA	NA	NA	NA
######## Yes	NA	NA	NA	NA	########	########	NA
######## Yes	NA	########	########	NA	NA	NA	########
######## Yes	########	########	NA	NA	NA	NA	NA
######## No	NA	NA	NA	NA	NA	NA	NA
######## Yes	NA	NA	NA	NA	NA	NA	NA
######## Yes	NA	NA	NA	NA	NA	########	NA
######## Yes	########	NA	NA	########	########	########	NA
######## Yes	########	########	NA	NA	########	########	NA
######## No	NA	NA	NA	NA	NA	NA	NA
######## Yes	NA	NA	NA	NA	########	NA	NA
######## Yes	NA	NA	NA	NA	########	NA	NA
######## Yes	NA	NA	NA	NA	NA	########	NA
######## Yes	NA	NA	NA	NA	NA	########	NA
######## Yes	########	NA	########	########	########	########	########
######## Yes	NA	NA	NA	NA	########	########	NA
######## Yes	NA	NA	NA	NA	NA	########	NA
######## Yes	NA	NA	NA	NA	NA	########	NA
######## Yes	NA	NA	########	########	########	########	NA
######## Yes	NA	NA	NA	########	NA	########	NA
######## Yes	NA	NA	NA	NA	NA	########	NA
######## Yes	NA	NA	NA	NA	NA	NA	NA
######## Yes	NA	NA	NA	NA	NA	########	NA
######## Yes	NA	NA	NA	NA	NA	########	NA
######## No	NA	NA	NA	NA	NA	NA	NA
######## No	NA	NA	NA	NA	NA	NA	NA
######## No	NA	NA	NA	NA	NA	NA	NA
######## Yes	########	########	NA	########	########	########	NA
######## No	NA	NA	NA	NA	NA	NA	NA
######## No	NA	NA	NA	NA	NA	NA	NA
######## No	NA	NA	NA	NA	NA	NA	NA
######## No	NA	NA	NA	NA	NA	NA	NA
######## Yes	NA	NA	NA	NA	NA	########	NA
######## Yes	NA	NA	NA	NA	########	########	NA
######## No	NA	NA	NA	NA	NA	NA	NA
######## Yes	NA	NA	NA	########	########	########	NA
######## Yes	NA	NA	NA	NA	NA	NA	NA
######## Yes	NA	NA	NA	NA	########	NA	NA
######## Yes	NA	NA	NA	NA	########	NA	NA
######## Yes	########	########	NA	########	########	########	NA
######## Yes	NA	NA	NA	NA	########	NA	NA
######## Yes	NA	NA	NA	NA	########	NA	NA
######## Yes	########	########	NA	########	########	########	########
######## No	NA	NA	NA	NA	NA	NA	NA

For Review Only

########	Yes	NA	NA	NA	########	########	########	NA
########	Yes	NA	########	NA	NA	########	########	NA
########	Yes	########	########	########	########	########	########	NA
########	Yes	NA	NA	NA	NA	NA	########	NA
########	Yes	NA	NA	NA	NA	########	########	NA
########	Yes	NA	NA	NA	NA	NA	NA	NA
########	Yes	NA	NA	NA	NA	########	NA	NA
########	Yes	########	########	########	NA	NA	########	########
########	Yes	########	########	NA	########	########	NA	NA
########	Yes	NA	NA	NA	########	########	########	NA
########	Yes	NA	NA	NA	NA	########	NA	NA
########	Yes	NA	NA	NA	NA	NA	########	NA
########	Yes	NA	NA	NA	NA	NA	NA	NA
########	Yes	NA	NA	NA	########	########	########	NA
########	Yes	NA	NA	NA	NA	NA	########	NA
########	Yes	NA	NA	NA	########	########	########	NA
########	Yes	########	NA	########	########	NA	NA	NA
########	Yes	NA	NA	########	########	NA	NA	NA
########	Yes	NA	NA	NA	########	########	########	NA
########	Yes	########	########	NA	########	########	########	NA
########	Yes	########	########	NA	########	########	########	NA
########	Yes	NA	NA	NA	########	NA	########	NA
########	Yes	NA	NA	NA	NA	NA	########	NA
########	Yes	NA	NA	NA	########	########	########	NA
########	Yes	NA	########	NA	NA	NA	NA	NA
########	Yes	NA	NA	NA	NA	NA	########	NA
########	Yes	NA	NA	NA	NA	NA	########	NA
########	Yes	NA	########	NA	NA	NA	NA	NA
########	Yes	NA	########	NA	########	########	########	NA
########	Yes	NA	########	NA	########	########	########	NA
########	Yes	########	########	NA	NA	NA	NA	NA
########	Yes	NA	NA	NA	NA	NA	########	NA
########	Yes	NA	NA	NA	NA	########	NA	NA
########	No	NA	NA	NA	NA	NA	NA	NA
########	Yes	########	########	########	########	########	########	NA
########	Yes	########	########	########	########	########	########	NA
########	Yes	NA	NA	NA	NA	########	########	NA
########	Yes	########	########	########	########	########	########	NA
########	Yes	NA	NA	NA	NA	########	########	NA
########	Yes	NA	NA	########	NA	NA	########	NA
########	Yes	NA	NA	NA	NA	NA	########	NA
########	Yes	NA	NA	NA	########	########	########	NA
########	Yes	########	########	NA	########	########	########	NA
########	Yes	NA	NA	NA	NA	########	NA	NA
########	No	NA	NA	NA	NA	NA	NA	NA
########	Yes	NA	########	NA	NA	NA	NA	NA
########	No	NA	NA	NA	NA	NA	NA	NA
########	Yes	NA	NA	NA	########	NA	########	NA
########	Yes	NA	NA	NA	NA	########	NA	NA
########	Yes	NA	########	########	########	########	########	NA
########	Yes	NA	NA	NA	NA	NA	NA	NA
########	Yes	NA	########	NA	########	########	########	NA
########	Yes	NA	NA	NA	NA	NA	########	NA
########	Yes	NA	NA	NA	NA	NA	########	NA
########	No	NA	NA	NA	NA	NA	NA	NA
########	Yes	NA	NA	NA	NA	########	########	NA

For Review Only

######## Yes	########	########	########	########	########	########	NA
######## Yes	NA	NA	########	########	########	########	NA
######## Yes	NA	NA	NA	########	NA	########	NA
######## Yes	NA	NA	NA	NA	NA	########	NA
######## No	NA	NA	NA	NA	NA	NA	NA
######## Yes	NA	NA	NA	NA	NA	########	NA
######## Yes	########	NA	NA	NA	NA	NA	NA
######## Yes	NA	NA	NA	NA	NA	########	NA
######## Yes	########	########	NA	########	########	########	NA
######## Yes	########	########	NA	NA	NA	NA	NA
######## Yes	########	########	########	########	########	########	NA
######## Yes	NA	NA	NA	NA	NA	########	NA
######## No	NA	NA	NA	NA	NA	NA	NA
######## Yes	NA	NA	NA	NA	NA	########	NA
######## Yes	NA	########	########	NA	NA	NA	NA
######## Yes	NA	########	NA	NA	NA	NA	NA
######## No	NA	NA	NA	NA	NA	NA	NA
######## Yes	NA	NA	########	NA	NA	NA	NA
######## Yes	NA	NA	########	########	########	NA	NA
######## Yes	NA	NA	NA	NA	NA	########	NA
######## No	NA	NA	NA	NA	NA	NA	NA
######## Yes	NA	NA	NA	########	########	########	NA
######## Yes	NA	NA	NA	NA	NA	########	NA
######## Yes	NA	NA	NA	NA	########	########	NA
######## Yes	NA	NA	NA	NA	NA	########	NA
######## Yes	NA	NA	NA	NA	########	NA	NA
######## Yes	NA	NA	NA	NA	NA	NA	NA
######## Yes	NA	NA	########	########	########	NA	NA
######## Yes	########	########	########	########	########	########	NA
######## Yes	NA	NA	########	NA	NA	########	NA
######## Yes	########	########	NA	########	########	########	NA
######## Yes	NA	NA	NA	NA	NA	########	NA
######## Yes	NA	NA	NA	NA	########	########	NA
######## Yes	NA	NA	NA	########	NA	NA	NA
######## Yes	NA	NA	NA	NA	NA	########	NA
######## Yes	NA	NA	NA	NA	NA	########	NA
######## Yes	NA	NA	NA	NA	NA	NA	NA
######## Yes	########	########	########	########	########	########	NA
######## Yes	NA	NA	NA	NA	########	########	NA
######## Yes	NA	NA	NA	########	########	NA	NA
######## Yes	NA	NA	NA	NA	NA	########	NA
######## Yes	NA	NA	NA	NA	NA	NA	NA
######## Yes	NA	NA	NA	NA	########	########	NA
######## Yes	NA	NA	NA	NA	########	########	NA
######## No	NA	NA	NA	NA	NA	NA	NA
######## Yes	NA	NA	NA	NA	NA	########	NA
######## Yes	NA	NA	NA	NA	NA	########	NA
######## Yes	########	########	########	########	########	########	NA
######## Yes	NA	NA	NA	NA	########	########	NA
######## Yes	NA	NA	NA	NA	NA	NA	NA
######## Yes	NA	NA	NA	NA	NA	NA	NA
######## Yes	NA	NA	NA	NA	NA	########	NA
######## Yes	NA	NA	NA	NA	NA	########	NA
######## Yes	NA	########	NA	########	########	########	NA
######## Yes	NA	NA	NA	NA	NA	NA	NA
######## Yes	########	NA	########	########	########	########	NA

For Review Only

######## Yes	NA	NA	NA	########	########	########	NA
######## No	NA	NA	NA	NA	NA	NA	NA
######## Yes	NA	NA	########	########	########	########	NA
######## Yes	########	NA	########	NA	NA	NA	NA
######## No	NA	NA	NA	NA	NA	NA	NA
######## Yes	NA	NA	NA	########	########	NA	NA
######## Yes	########	########	NA	NA	NA	NA	NA
######## Yes	NA	NA	NA	NA	########	NA	NA
######## Yes	NA	NA	########	########	########	########	NA
######## Yes	NA	NA	NA	########	NA	NA	NA
######## Yes	########	########	########	########	########	########	NA
######## Yes	NA	NA	########	########	########	NA	NA
######## Yes	NA	NA	NA	NA	NA	########	NA
######## Yes	NA	NA	NA	NA	NA	########	NA
######## Yes	NA	NA	NA	NA	NA	########	NA
######## Yes	NA	NA	NA	########	########	########	NA
######## Yes	NA	NA	NA	NA	########	########	NA
######## Yes	NA	NA	NA	NA	########	########	NA
######## Yes	NA	NA	NA	NA	NA	########	NA
######## No	NA	NA	NA	NA	NA	NA	NA
######## Yes	NA	NA	NA	NA	NA	########	NA
######## Yes	NA	NA	NA	NA	NA	NA	NA
######## Yes	########	########	########	########	NA	########	NA
######## Yes	NA	NA	NA	NA	NA	########	NA
######## Yes	########	########	########	########	########	########	NA
######## Yes	########	########	########	########	########	########	NA
######## Yes	NA	NA	NA	########	NA	########	NA
######## Yes	########	########	NA	########	########	########	NA
######## Yes	NA	NA	NA	NA	########	NA	NA
######## Yes	NA	NA	NA	NA	NA	########	NA
######## Yes	NA	NA	NA	########	########	NA	NA
######## Yes	NA	NA	NA	NA	NA	########	NA
######## No	NA	NA	NA	NA	NA	NA	NA
######## Yes	NA	NA	NA	NA	NA	########	NA
######## Yes	NA	NA	NA	NA	########	########	NA
######## Yes	NA	NA	NA	NA	NA	########	NA
######## No	NA	NA	NA	NA	NA	NA	NA
######## Yes	########	########	NA	NA	NA	########	NA
######## No	NA	NA	NA	NA	NA	NA	NA
######## Yes	NA	NA	NA	NA	NA	########	NA
######## Yes	NA	NA	NA	NA	NA	########	NA
######## Yes	NA	NA	NA	NA	NA	NA	NA
######## Yes	NA	NA	NA	NA	NA	########	NA
######## No	NA	NA	NA	NA	NA	NA	NA
######## Yes	NA	NA	NA	########	########	########	NA
######## No	NA	NA	NA	NA	NA	NA	NA
######## Yes	########	########	NA	NA	########	########	NA
######## Yes	NA	NA	NA	NA	NA	NA	NA
######## Yes	NA	NA	NA	########	########	########	NA
######## Yes	NA	NA	NA	NA	NA	########	NA
######## Yes	########	########	NA	########	########	########	########
######## Yes	NA	NA	NA	NA	NA	########	NA
######## Yes	NA	NA	NA	NA	NA	NA	NA
######## Yes	NA	NA	NA	NA	NA	########	NA
######## Yes	NA	NA	NA	NA	NA	########	NA
######## Yes	########	########	########	########	########	########	NA

For Review Only

######## No	NA	NA	NA	NA	NA	NA	NA
######## Yes	NA	NA	NA	NA	NA	########	NA
######## Yes	NA	NA	NA	NA	NA	########	NA
######## No	NA	NA	NA	NA	NA	NA	NA
######## Yes	NA	NA	NA	NA	NA	NA	NA
######## Yes	########	########	########	########	########	########	NA
######## Yes	NA	NA	########	########	########	NA	NA
######## Yes	NA	NA	NA	NA	NA	NA	NA
######## Yes	NA	NA	########	########	########	########	NA
######## Yes	NA	NA	NA	NA	NA	NA	NA
######## Yes	NA	NA	NA	NA	########	########	NA
######## Yes	NA	NA	NA	NA	NA	NA	NA
######## Yes	NA	NA	NA	NA	NA	NA	NA
######## Yes	########	NA	NA	NA	NA	NA	NA
######## Yes	########	########	NA	########	########	########	NA
######## Yes	NA	NA	NA	NA	NA	NA	NA
######## Yes	NA	NA	NA	NA	NA	NA	NA
######## No	NA	NA	NA	NA	NA	NA	NA
######## Yes	NA	NA	NA	########	########	########	NA
######## Yes	NA	NA	NA	NA	NA	NA	NA
######## Yes	NA	NA	NA	NA	########	NA	NA
######## No	NA	NA	NA	NA	NA	NA	NA
######## Yes	NA	NA	NA	NA	########	########	NA
######## Yes	NA	NA	NA	NA	########	NA	NA
######## Yes	NA	NA	NA	NA	NA	########	NA
######## Yes	NA	NA	########	########	NA	NA	NA
######## No	NA	NA	NA	NA	NA	NA	NA
######## No	NA	NA	NA	NA	NA	NA	NA
######## Yes	########	########	########	########	########	########	NA
######## Yes	NA	NA	NA	########	########	NA	NA
######## No	NA	NA	NA	NA	NA	NA	NA
######## No	NA	NA	NA	NA	NA	NA	NA
######## Yes	NA	NA	########	########	########	NA	NA
######## Yes	########	NA	########	########	########	########	NA
######## Yes	NA	NA	NA	NA	NA	########	NA
######## No	NA	NA	NA	NA	NA	NA	NA
######## Yes	NA	NA	NA	NA	NA	########	NA
######## Yes	NA	NA	NA	NA	########	NA	NA
######## Yes	NA	NA	NA	########	########	NA	NA
######## Yes	NA	NA	NA	NA	NA	NA	NA
######## Yes	########	########	########	########	NA	########	NA
######## No	NA	NA	NA	NA	NA	NA	NA
######## Yes	NA	NA	NA	NA	NA	NA	NA
######## Yes	########	########	########	########	########	########	NA
######## Yes	NA	NA	NA	NA	NA	NA	NA
######## Yes	########	NA	########	########	########	NA	########
######## Yes	NA	########	NA	########	########	########	NA
######## Yes	NA	NA	NA	########	########	########	NA
######## Yes	########	########	########	########	########	########	NA
######## Yes	NA	NA	NA	########	########	########	NA
######## Yes	NA	NA	NA	NA	NA	########	NA
######## Yes	NA	NA	NA	NA	NA	########	NA
######## Yes	NA	NA	NA	NA	########	########	NA
######## Yes	NA	NA	NA	########	########	########	NA
######## Yes	NA	NA	NA	NA	########	########	NA
######## Yes	########	NA	NA	NA	########	########	NA

For Review Only

######## Yes	########	NA	NA	########	########	########	NA
######## Yes	NA	NA	NA	NA	NA	NA	NA
######## Yes	NA	NA	########	########	########	NA	NA
######## Yes	NA	NA	NA	NA	########	########	NA
######## Yes	NA	NA	NA	NA	########	NA	NA
######## Yes	NA	NA	NA	NA	NA	########	NA
######## Yes	NA	NA	NA	########	########	########	NA
######## Yes	NA	NA	NA	NA	########	NA	NA
######## Yes	########	########	NA	NA	NA	NA	NA
######## Yes	NA	NA	NA	NA	########	########	NA
######## Yes	NA	NA	########	########	########	########	NA
######## Yes	NA	NA	NA	########	########	########	NA
######## Yes	########	########	########	NA	NA	########	NA
######## Yes	NA	NA	NA	NA	NA	########	NA
######## Yes	NA	########	NA	NA	########	########	NA
######## Yes	NA	NA	NA	NA	########	NA	NA
######## No	NA	NA	NA	NA	NA	NA	NA
######## Yes	NA	NA	NA	NA	########	########	NA
######## Yes	NA	NA	NA	NA	NA	########	NA
######## Yes	NA	########	NA	########	########	########	NA
######## Yes	########	########	########	NA	NA	########	NA
######## Yes	NA	NA	NA	NA	NA	########	NA
######## Yes	NA	NA	NA	NA	########	NA	NA
######## Yes	NA	NA	########	NA	NA	########	NA
######## No	NA	NA	NA	NA	NA	NA	NA
######## Yes	NA	NA	NA	NA	########	########	NA
######## Yes	########	########	NA	NA	########	########	NA
######## Yes	NA	########	NA	NA	NA	NA	########
######## Yes	NA	NA	NA	NA	NA	########	NA
######## No	NA	NA	NA	NA	NA	NA	NA
######## Yes	NA	NA	########	NA	NA	NA	NA
######## Yes	NA	NA	NA	NA	NA	NA	NA
######## No	NA	NA	NA	NA	NA	NA	NA
######## No	NA	NA	NA	NA	NA	NA	NA
######## Yes	NA	NA	NA	NA	NA	########	NA
######## Yes	NA	NA	NA	NA	NA	########	NA
######## Yes	########	########	########	########	########	########	NA
######## Yes	NA	NA	NA	NA	NA	########	NA
######## Yes	NA	NA	NA	NA	NA	########	NA
######## Yes	########	NA	NA	NA	########	########	NA
######## Yes	########	########	########	########	########	########	########
######## Yes	NA	NA	########	########	NA	NA	NA
######## Yes	NA	NA	NA	########	########	########	NA
######## No	NA	NA	NA	NA	NA	NA	NA
######## Yes	NA	NA	NA	########	NA	########	NA
######## Yes	NA	NA	NA	NA	NA	########	NA
######## Yes	########	########	########	########	########	########	NA
######## Yes	NA	########	########	########	########	########	NA
######## Yes	NA	NA	NA	NA	########	NA	NA
######## Yes	########	########	NA	NA	NA	NA	NA
######## Yes	NA	########	NA	NA	NA	NA	NA
######## Yes	NA	NA	NA	########	########	########	NA
######## Yes	NA	NA	########	NA	NA	NA	NA
######## Yes	NA	NA	NA	NA	NA	########	NA
######## No	NA	NA	NA	NA	NA	NA	NA
######## Yes	NA	NA	NA	########	########	NA	NA

For Review Only

########	Yes	NA	NA	NA	NA	NA	########	NA
########	Yes	NA	NA	NA	NA	########	########	NA
########	Yes	NA	NA	NA	NA	########	NA	NA
########	Yes	NA	NA	NA	NA	NA	NA	NA
########	Yes	NA	NA	NA	NA	NA	########	NA
########	Yes	NA	NA	NA	NA	NA	########	NA
########	Yes	########	NA	NA	########	########	########	NA
########	No	NA	NA	NA	NA	NA	NA	NA
########	Yes	NA	NA	NA	NA	NA	########	NA
########	Yes	########	########	########	########	########	########	NA
########	Yes	NA	NA	########	########	########	########	NA
########	Yes	########	########	NA	NA	NA	########	NA
########	Yes	NA	NA	NA	NA	NA	NA	NA
########	Yes	NA	NA	NA	NA	########	NA	NA
########	Yes	########	########	########	########	########	########	NA
########	Yes	########	########	NA	########	########	########	NA
########	Yes	########	########	NA	NA	NA	NA	NA
########	Yes	NA	NA	NA	NA	########	########	NA
########	Yes	NA	NA	NA	NA	NA	NA	NA
########	Yes	NA	NA	NA	NA	NA	########	NA
########	Yes	NA	NA	NA	########	########	########	NA
########	Yes	NA	NA	NA	NA	NA	NA	NA
########	Yes	NA	NA	NA	NA	NA	########	NA
########	Yes	NA	NA	NA	NA	NA	########	NA
########	Yes	NA	NA	########	NA	NA	########	NA
########	Yes	########	########	########	########	########	########	NA
########	Yes	NA	NA	NA	########	########	########	NA
########	Yes	NA	########	########	########	########	########	NA
########	Yes	########	########	########	########	########	########	NA
########	Yes	NA	NA	NA	NA	NA	########	NA
########	Yes	NA	NA	########	NA	NA	########	NA
########	No	NA	NA	NA	NA	NA	NA	NA
########	Yes	NA	NA	NA	NA	########	########	NA
########	Yes	########	########	NA	########	########	########	NA
########	Yes	NA	NA	NA	NA	NA	########	NA
########	Yes	########	########	########	NA	########	########	NA
########	No	NA	NA	NA	NA	NA	NA	NA
########	Yes	NA	NA	NA	NA	NA	NA	NA
########	Yes	NA	NA	NA	NA	########	########	NA
########	Yes	NA	NA	NA	NA	NA	NA	NA
########	Yes	########	########	NA	########	########	########	NA
########	No	NA	NA	NA	NA	NA	NA	NA
########	Yes	NA	NA	NA	########	########	NA	NA
########	Yes	NA	NA	########	########	########	########	NA
########	Yes	NA	NA	NA	NA	########	NA	NA
########	Yes	NA	NA	NA	NA	NA	########	NA
########	Yes	NA	NA	NA	########	########	########	NA
########	Yes	NA	########	########	NA	NA	NA	NA
########	Yes	NA	NA	NA	########	########	########	NA
########	Yes	NA	NA	NA	NA	NA	NA	NA
########	No	NA	NA	NA	NA	NA	NA	NA
########	Yes	NA	NA	NA	NA	NA	########	NA
########	Yes	NA	NA	NA	NA	NA	########	NA
########	Yes	NA	NA	NA	########	NA	########	NA
########	No	NA	NA	NA	NA	NA	NA	NA
########	Yes	########	NA	NA	NA	########	########	NA

For Review Only

######## No	NA	NA	NA	NA	NA	NA	NA
######## Yes	########	########	NA	########	########	########	NA
######## Yes	NA	NA	NA	NA	NA	########	NA
######## Yes	########	########	NA	########	########	########	NA
######## Yes	NA	NA	NA	NA	NA	########	NA
######## Yes	NA	NA	NA	NA	NA	NA	NA
######## Yes	NA	NA	NA	NA	NA	NA	NA
######## Yes	NA	########	########	########	########	########	NA
######## Yes	NA	NA	NA	NA	NA	########	NA
######## Yes	NA	NA	NA	NA	########	########	NA
######## Yes	########	########	NA	########	########	########	NA
######## Yes	NA	NA	########	########	########	########	NA
######## Yes	NA	NA	NA	NA	NA	NA	NA
######## Yes	NA	NA	NA	NA	NA	########	NA
######## Yes	########	NA	NA	########	########	########	NA
######## Yes	NA	NA	NA	########	########	NA	NA

For Review Only

4-2	5-2	6-2	7-2	4-3	5-3	6-3	7-3	5-4
NA	NA	NA	NA	NA	NA	NA	NA	NA
NA	NA	NA	NA	NA	NA	NA	NA	NA
NA	NA	NA	NA	NA	NA	NA	NA	NA
NA	NA	NA	########	NA	NA	NA	########	NA
NA	NA	NA	########	NA	NA	NA	########	NA
NA	NA	NA	NA	NA	NA	########	NA	NA
NA	########	########	########	########	########	########	########	NA
NA	NA	NA	NA	NA	NA	NA	NA	NA
NA	NA	NA	NA	NA	NA	NA	NA	NA
NA	########	########	########	NA	########	########	########	NA
NA	NA	NA	NA	NA	NA	NA	NA	NA
NA	NA	NA	########	NA	NA	NA	########	NA
NA	NA	NA	########	NA	NA	NA	########	NA
NA	NA	NA	NA	NA	NA	NA	NA	NA
NA	NA	NA	NA	NA	NA	NA	########	NA
NA	NA	NA	NA	NA	NA	NA	NA	NA
NA	NA	NA	NA	NA	NA	NA	NA	NA
NA	NA	NA	########	NA	NA	NA	########	NA
NA	NA	NA	NA	NA	NA	NA	NA	NA
NA	NA	NA	NA	NA	NA	NA	NA	NA
NA	NA	NA	NA	NA	NA	NA	NA	NA
NA	NA	########	NA	NA	NA	NA	NA	NA
NA	NA	NA	NA	NA	NA	NA	NA	NA
NA	NA	NA	NA	NA	NA	NA	NA	NA
NA	NA	NA	NA	NA	NA	NA	NA	NA
NA	NA	NA	NA	NA	NA	NA	NA	NA
NA	NA	NA	NA	NA	NA	NA	NA	NA
NA	NA	NA	########	NA	NA	NA	NA	NA
NA	NA	NA	########	NA	NA	NA	########	NA
NA	NA	########	########	NA	NA	NA	########	NA
NA	NA	NA	########	NA	NA	NA	########	NA
########	########	########	########	########	########	NA	########	NA
NA	NA	NA	NA	NA	NA	NA	########	NA
NA	NA	NA	NA	NA	NA	NA	NA	NA
NA	NA	NA	NA	NA	NA	NA	NA	NA
NA	NA	NA	NA	NA	NA	NA	NA	NA
NA	NA	NA	NA	NA	NA	NA	NA	NA
NA	NA	NA	########	NA	NA	NA	########	NA
NA	NA	NA	NA	NA	NA	NA	NA	NA
NA	NA	NA	########	NA	NA	NA	########	NA
########	########	########	########	NA	########	########	########	NA
NA	NA	########	NA	NA	NA	########	NA	NA
NA	NA	NA	NA	NA	NA	NA	NA	NA
NA	NA	########	########	NA	NA	########	########	NA
########	########	########	########	########	########	########	########	NA
NA	NA	NA	########	NA	NA	NA	########	NA
NA	NA	NA	NA	NA	NA	NA	NA	NA
NA	NA	########	NA	NA	########	########	NA	NA
NA	NA	NA	NA	NA	NA	NA	NA	NA
NA	NA	NA	NA	NA	NA	NA	########	NA
NA	NA	NA	NA	NA	NA	NA	NA	NA
NA	NA	NA	########	NA	NA	NA	########	NA
NA	NA	NA	NA	NA	NA	NA	NA	NA
NA	NA	NA	NA	NA	NA	NA	NA	NA

For Review Only

########	########	########	########	########	########	########	########	########
NA	NA	########	########	NA	NA	########	########	NA
NA	NA	NA	########	NA	NA	NA	########	NA
NA	NA	NA	########	NA	NA	NA	########	NA
NA	NA	NA	########	NA	NA	NA	########	NA
NA	NA	NA	NA	NA	NA	NA	NA	NA
NA	NA	NA	########	NA	NA	NA	########	NA
NA	NA	########	NA	NA	NA	########	NA	NA
########	########	########	########	NA	NA	NA	########	NA
########	########	########	########	########	########	########	########	########
NA	NA	NA	NA	NA	NA	NA	NA	NA
NA	NA	########	########	NA	NA	########	########	NA
NA	########	########	########	NA	########	########	########	NA
NA	NA	NA	########	NA	NA	NA	########	NA
NA	NA	NA	NA	NA	NA	NA	NA	NA
NA	NA	NA	########	NA	NA	NA	########	NA
NA	NA	NA	NA	NA	NA	NA	NA	NA
NA	NA	NA	NA	NA	NA	NA	NA	NA
NA	NA	NA	NA	NA	NA	NA	########	NA
NA	NA	NA	########	NA	NA	NA	NA	NA
NA	########	########	########	########	########	########	########	########
NA	########	NA	########	NA	########	NA	########	NA
NA	NA	NA	NA	NA	NA	NA	NA	NA
NA	NA	########	NA	NA	NA	NA	NA	NA
NA	NA	NA	########	NA	NA	NA	NA	NA
NA	NA	NA	########	NA	NA	########	########	NA
NA	NA	NA	########	NA	NA	NA	########	NA
NA	NA	NA	########	NA	NA	NA	########	NA
NA	NA	########	NA	NA	NA	NA	NA	NA
NA	NA	NA	NA	NA	NA	NA	NA	NA
NA	NA	########	########	NA	NA	NA	########	NA
########	########	########	########	########	########	########	########	NA
########	########	########	NA	########	########	########	########	NA
NA	########	########	########	########	########	########	########	NA
NA	NA	NA	########	NA	NA	NA	########	NA
NA	########	########	########	NA	########	########	########	########
NA	NA	NA	########	NA	NA	NA	########	NA
########	########	########	########	########	########	########	########	########
NA	NA	NA	NA	NA	########	NA	NA	NA
########	########	########	########	########	########	########	########	########
NA	NA	NA	########	NA	NA	NA	NA	NA
########	########	########	########	########	########	########	########	NA
NA	NA	NA	NA	NA	NA	NA	NA	NA
########	########	########	########	########	########	########	########	########
NA	NA	NA	NA	NA	NA	NA	NA	NA
NA	########	########	########	NA	########	########	########	NA
NA	NA	NA	########	NA	NA	NA	########	NA
NA	NA	NA	NA	NA	NA	NA	NA	NA
########	NA	NA	NA	########	NA	NA	NA	########
NA	NA	NA	NA	NA	NA	NA	NA	NA
NA	NA	########	NA	NA	NA	########	NA	NA
########	########	########	########	NA	########	########	########	########
########	NA	NA	########	########	NA	NA	########	########
NA	NA	NA	########	NA	NA	NA	########	NA
NA	NA	NA	########	NA	NA	NA	NA	NA

For Review Only

NA	########	########	########	NA	########	########	########	NA
NA	NA	NA	########	NA	NA	NA	########	NA
NA	NA	NA	########	NA	NA	NA	########	NA
NA	NA	########	########	NA	NA	########	########	NA
NA	NA	NA	########	NA	NA	NA	########	NA
NA	NA	NA	########	NA	NA	NA	########	NA
NA	NA	########	########	NA	NA	########	########	NA
NA	NA	########	########	NA	NA	NA	########	NA
NA	NA	NA	NA	NA	NA	########	NA	NA
NA	NA	NA	NA	NA	NA	NA	NA	NA
NA	########	########	########	########	########	########	########	########
NA	########	########	########	NA	########	########	########	########
NA	NA	NA	NA	NA	NA	NA	NA	NA
NA	########	########	########	NA	NA	NA	########	NA
########	########	########	########	NA	########	########	########	NA
########	########	########	########	########	########	########	########	########
NA	########	NA	########	NA	NA	NA	########	########
NA	NA	NA	NA	NA	NA	NA	NA	NA
NA	NA	NA	########	NA	NA	NA	########	NA
NA	NA	########	NA	NA	NA	########	NA	NA
NA	NA	NA	NA	NA	NA	NA	NA	NA
NA	NA	########	########	NA	NA	########	########	########
########	NA	########	########	NA	NA	########	########	NA
########	########	########	########	NA	NA	NA	########	NA
NA	NA	########	NA	NA	NA	NA	########	NA
NA	NA	NA	NA	NA	NA	NA	NA	NA
########	########	########	########	########	########	########	########	NA
NA	NA	########	NA	NA	NA	########	NA	NA
NA	NA	NA	########	NA	NA	NA	########	NA
NA	NA	NA	########	NA	NA	NA	########	NA
NA	########	########	########	########	########	########	########	NA
NA	NA	########	NA	NA	NA	########	NA	NA
NA	NA	########	########	NA	NA	NA	########	NA
NA	########	########	########	########	########	########	########	NA
########	########	########	########	########	########	########	########	NA
########	########	########	NA	########	########	########	NA	NA
NA	NA	NA	NA	NA	NA	NA	########	NA
NA	NA	NA	NA	NA	NA	NA	NA	NA
NA	########	NA	NA	NA	########	NA	NA	########
NA	NA	NA	########	NA	NA	NA	########	NA
NA	NA	NA	NA	NA	NA	NA	NA	NA
NA	NA	NA	########	NA	NA	NA	########	NA
########	NA	########	########	########	NA	########	########	########
NA	NA	NA	NA	NA	NA	NA	NA	NA
NA	NA	NA	NA	NA	NA	NA	NA	NA
########	NA	NA	NA	########	NA	NA	########	########
########	########	########	########	NA	########	########	########	NA
NA	NA	########	NA	NA	NA	########	NA	NA
NA	NA	########	NA	NA	NA	########	NA	NA
NA	########	########	########	NA	########	########	########	NA
NA	NA	NA	NA	NA	NA	NA	NA	NA
NA	NA	NA	NA	NA	NA	NA	NA	NA
NA	########	########	NA	NA	########	########	NA	NA
NA	NA	NA	NA	NA	NA	NA	NA	NA
NA	NA	NA	NA	NA	NA	NA	NA	NA
NA	NA	NA	NA	NA	NA	NA	NA	NA

For Review Only

NA	NA	NA	########	NA	NA	NA	########	NA
NA	NA	NA	NA	NA	NA	NA	NA	NA
NA	NA	NA	NA	NA	NA	NA	NA	NA
########	NA	NA	NA	NA	NA	NA	NA	NA
NA	NA	NA	NA	NA	NA	NA	NA	NA
NA	NA	NA	NA	NA	NA	NA	NA	NA
########	########	########	########	########	########	########	########	########
NA	########	########	########	NA	########	########	########	NA
NA	NA	NA	########	NA	NA	NA	########	NA
NA	NA	NA	########	NA	NA	NA	########	NA
########	NA	NA	NA	NA	########	########	########	########
NA	########	########	########	NA	########	########	########	########
NA	NA	########	########	NA	NA	NA	########	NA
########	########	########	NA	########	########	########	NA	NA
NA	NA	########	NA	NA	NA	########	NA	NA
NA	NA	NA	########	NA	NA	NA	########	NA
NA	NA	########	NA	NA	NA	########	NA	NA
NA	NA	NA	########	NA	NA	NA	########	NA
NA	NA	########	########	NA	NA	########	########	NA
NA	########	NA	########	NA	########	NA	########	NA
NA	NA	NA	########	NA	NA	NA	########	NA
NA	########	########	NA	NA	NA	NA	NA	NA
NA	NA	NA	########	NA	NA	NA	########	NA
NA	NA	NA	########	NA	NA	NA	########	NA
########	########	########	NA	########	########	########	NA	NA
NA	NA	NA	NA	NA	NA	NA	NA	NA
NA	NA	NA	NA	NA	NA	NA	NA	NA
NA	NA	NA	########	NA	NA	NA	########	NA
NA	NA	NA	NA	NA	NA	NA	NA	NA
NA	NA	NA	########	NA	NA	NA	########	NA
########	########	########	########	########	########	########	########	########
########	########	########	########	########	########	########	########	########
NA	########	########	########	########	########	########	########	########
########	########	########	########	########	########	########	########	########
NA	NA	NA	########	NA	NA	NA	########	NA
NA	NA	NA	NA	NA	NA	NA	NA	NA
########	########	########	########	NA	########	########	########	NA
NA	NA	NA	########	NA	NA	NA	########	NA
NA	NA	NA	NA	NA	NA	NA	NA	NA
NA	NA	NA	########	NA	NA	NA	########	NA
NA	NA	NA	########	NA	NA	########	########	NA
NA	NA	NA	NA	NA	NA	NA	NA	NA
NA	NA	########	########	NA	NA	########	NA	NA
NA	NA	NA	NA	NA	NA	NA	NA	NA
NA	NA	NA	########	NA	NA	NA	########	NA
NA	NA	NA	NA	NA	########	########	########	NA
NA	NA	NA	########	NA	NA	NA	########	NA
NA	########	########	NA	NA	########	########	NA	NA
NA	NA	NA	NA	NA	NA	########	NA	NA
########	########	########	########	########	########	########	########	########
NA	NA	NA	NA	NA	NA	NA	NA	NA
NA	NA	NA	NA	NA	NA	NA	NA	NA
NA	########	########	########	NA	########	########	########	########
########	########	########	########	########	########	########	########	########
########	########	########	########	########	########	########	########	NA

For Review Only

########	########	########	########	NA	########	########	########	NA
NA	NA	NA	NA	NA	NA	NA	NA	NA
NA	NA	########	########	NA	NA	########	########	NA
NA	########	########	########	NA	NA	NA	########	NA
NA	NA	########	########	NA	NA	NA	########	NA
NA	NA	NA	########	NA	NA	NA	NA	NA
NA	NA	NA	########	NA	NA	NA	########	NA
NA	NA	NA	NA	NA	NA	NA	NA	NA
########	########	########	########	########	########	########	########	########
NA	NA	########	########	NA	NA	########	########	NA
NA	NA	NA	NA	NA	NA	NA	NA	NA
########	########	########	########	NA	NA	NA	NA	NA
NA	NA	########	########	NA	NA	########	########	NA
NA	NA	NA	NA	NA	NA	NA	########	NA
NA	NA	NA	########	NA	NA	NA	########	NA
NA	NA	########	########	NA	NA	########	########	NA
NA	########	NA	NA	NA	########	NA	NA	NA
NA	NA	########	########	NA	NA	########	########	NA
NA	NA	########	########	NA	NA	########	########	NA
NA	NA	NA	NA	NA	NA	NA	NA	NA
NA	NA	NA	########	NA	NA	NA	########	NA
########	########	########	########	NA	########	########	########	########
NA	########	########	########	NA	########	########	########	NA
NA	NA	NA	NA	NA	NA	########	NA	NA
NA	NA	NA	NA	NA	########	NA	NA	NA
NA	NA	NA	NA	NA	NA	NA	NA	NA
NA	NA	NA	########	NA	NA	NA	########	NA
NA	NA	NA	########	NA	NA	NA	########	NA
NA	NA	NA	NA	NA	NA	NA	NA	NA
NA	########	NA	NA	NA	########	NA	NA	NA
########	########	########	########	########	########	########	########	NA
NA	NA	NA	NA	NA	NA	NA	NA	NA
NA	NA	NA	########	NA	NA	NA	########	NA
NA	NA	########	NA	NA	NA	########	NA	NA
########	########	########	########	########	########	########	########	NA
NA	NA	########	########	NA	NA	NA	########	NA
NA	NA	NA	########	NA	NA	NA	########	NA
NA	NA	NA	########	NA	NA	NA	########	NA
NA	NA	NA	########	NA	NA	NA	########	NA
NA	NA	NA	########	NA	########	NA	NA	NA
NA	NA	NA	NA	NA	NA	NA	NA	NA
NA	NA	NA	NA	NA	NA	NA	NA	########
NA	NA	NA	########	NA	NA	NA	########	NA
NA	NA	########	########	NA	NA	########	NA	NA
NA	NA	NA	########	NA	NA	NA	########	NA
NA	NA	NA	NA	NA	NA	NA	########	NA
NA	NA	NA	########	NA	NA	NA	########	NA
NA	########	NA	########	NA	NA	NA	########	NA
NA	NA	NA	NA	NA	NA	NA	NA	NA
NA	########	########	########	NA	########	########	########	########
NA	########	########	########	NA	########	########	########	NA
########	########	########	########	########	########	########	########	NA
NA	NA	NA	########	NA	NA	NA	########	NA
NA	NA	########	########	NA	NA	########	########	NA
NA	NA	NA	NA	NA	NA	NA	NA	NA
########	########	########	########	########	########	########	########	NA

For Review Only

NA	NA	NA	########	NA	NA	NA	########	NA
########	########	########	########	########	########	########	########	########
########	########	########	########	########	########	########	########	########
NA	########	########	########	########	########	########	########	NA
NA	########	########	########	########	########	########	########	NA
NA	########	########	########	NA	########	########	########	########
NA	NA	NA	########	NA	########	NA	########	NA
NA	NA	NA	NA	NA	NA	NA	NA	NA
NA	NA	NA	########	NA	NA	NA	NA	NA
########	NA	NA	NA	########	NA	NA	NA	########
NA	NA	########	########	NA	NA	########	NA	NA
NA	NA	NA	NA	NA	NA	NA	NA	NA
NA	NA	NA	########	NA	NA	NA	########	NA
NA	NA	NA	########	NA	NA	NA	########	NA
NA	########	########	########	NA	########	########	########	NA
NA	NA	########	########	NA	NA	########	########	########
NA	########	########	########	NA	NA	########	########	NA
########	########	########	########	########	########	########	########	########
########	NA	NA	########	NA	NA	NA	########	NA
########	########	########	########	########	########	########	########	########
NA	NA	########	########	NA	NA	NA	NA	NA
########	########	########	########	########	########	########	########	########
NA	########	########	NA	NA	########	########	NA	NA
NA	NA	NA	NA	NA	NA	NA	NA	NA
########	########	########	########	########	########	########	########	########
########	########	########	########	NA	########	########	########	########
NA	########	########	########	########	########	########	########	NA
NA	NA	NA	NA	NA	NA	NA	NA	NA
NA	NA	NA	NA	NA	NA	NA	NA	NA
NA	NA	########	NA	NA	NA	########	NA	NA
NA	NA	NA	NA	NA	NA	NA	########	NA
########	########	NA	########	NA	NA	NA	########	NA
NA	NA	NA	NA	NA	NA	NA	NA	NA
NA	NA	NA	########	NA	NA	NA	########	NA
NA	NA	NA	NA	NA	NA	NA	NA	NA
NA	NA	NA	########	NA	NA	NA	########	NA
NA	NA	NA	########	NA	NA	NA	########	NA
NA	NA	NA	NA	NA	NA	NA	NA	NA
########	########	########	########	########	########	########	########	########
NA	NA	NA	########	NA	NA	NA	########	NA
NA	NA	########	NA	NA	NA	########	NA	NA
NA	NA	########	NA	NA	NA	########	NA	NA
NA	NA	NA	########	NA	NA	NA	########	NA
NA	NA	NA	NA	NA	NA	NA	NA	NA
########	########	NA	NA	########	########	NA	NA	NA
NA	NA	NA	########	NA	NA	NA	########	NA
NA	########	########	########	########	########	########	########	NA
NA	########	########	########	NA	########	########	########	########
NA	NA	NA	########	NA	NA	NA	########	NA
NA	NA	NA	NA	NA	NA	NA	NA	NA
NA	NA	NA	NA	NA	NA	NA	NA	NA
NA	NA	NA	########	NA	NA	NA	########	NA
NA	NA	NA	########	NA	NA	NA	########	NA
NA	NA	NA	########	NA	NA	NA	########	NA
NA	NA	NA	NA	NA	NA	NA	NA	NA
NA	NA	NA	########	NA	NA	NA	NA	NA

For Review Only

NA	NA	NA	NA	NA	NA	NA	NA	NA
NA	NA	NA	NA	NA	NA	NA	NA	NA
########	########	########	########	########	########	########	########	########
NA	NA	NA	########	NA	NA	NA	########	NA
########	########	########	########	NA	########	########	########	NA
########	########	########	########	########	########	########	########	########
NA	NA	NA	########	NA	NA	NA	########	NA
########	########	########	NA	########	########	NA	NA	NA
NA	########	########	NA	NA	NA	########	NA	NA
NA	NA	NA	NA	NA	NA	NA	NA	NA
NA	NA	NA	########	NA	NA	NA	########	NA
NA	NA	NA	########	NA	NA	NA	########	NA
NA	########	NA	########	NA	########	NA	########	NA
NA	NA	NA	########	NA	NA	NA	########	NA
NA	NA	NA	NA	NA	NA	NA	NA	NA
NA	NA	NA	########	NA	NA	NA	########	NA
NA	NA	NA	########	NA	NA	NA	########	NA
########	########	########	########	########	########	########	########	########
########	########	########	########	NA	########	########	########	NA
NA	NA	NA	########	NA	NA	########	########	NA
NA	########	########	########	NA	########	########	########	NA
########	NA	NA	NA	NA	NA	NA	NA	NA
NA	NA	NA	NA	NA	NA	NA	NA	NA
NA	NA	NA	########	NA	NA	NA	########	NA
########	########	########	NA	########	########	NA	NA	NA
NA	NA	NA	########	NA	NA	NA	########	NA
NA	NA	NA	NA	NA	NA	NA	NA	NA
NA	NA	NA	########	NA	NA	NA	########	NA
########	########	########	########	NA	NA	NA	########	NA
########	########	########	########	########	########	########	########	########
NA	NA	NA	NA	NA	NA	NA	NA	NA
NA	########	########	########	NA	########	########	########	########
NA	NA	########	########	NA	NA	########	NA	NA
NA	NA	NA	########	NA	NA	NA	########	NA
NA	NA	NA	########	NA	NA	NA	########	NA
NA	NA	NA	########	NA	NA	NA	########	NA
########	########	########	########	########	########	########	########	########
NA	########	########	########	NA	########	########	########	########
########	########	########	########	########	########	########	########	NA
########	########	########	########	########	########	########	########	########
NA	########	########	########	NA	########	########	########	NA
########	########	########	########	########	########	########	########	########
NA	NA	NA	NA	NA	NA	NA	NA	NA
NA	NA	########	NA	NA	NA	########	NA	NA
########	########	########	########	########	########	########	########	########
NA	########	########	########	NA	########	########	########	NA
NA	########	########	########	NA	########	########	########	NA
NA	NA	########	########	NA	NA	########	########	NA
NA	NA	NA	NA	NA	NA	NA	NA	NA
NA	NA	NA	########	NA	NA	NA	########	NA
NA	NA	NA	NA	NA	NA	NA	NA	NA
NA	########	########	########	########	########	########	########	########
########	########	########	########	########	########	########	########	########
NA	NA	NA	NA	NA	NA	NA	NA	NA
NA	NA	NA	########	NA	NA	NA	########	NA
NA	NA	NA	########	NA	NA	NA	########	NA

For Review Only

NA	NA	########	########	NA	NA	########	########	NA
NA	NA	########	NA	NA	NA	NA	NA	NA
NA	NA	NA	########	NA	NA	NA	########	NA
NA	NA	NA	NA	NA	NA	NA	NA	NA
NA	NA	NA	########	NA	NA	########	########	########
NA	NA	NA	NA	NA	NA	NA	NA	NA
NA	########	########	########	NA	########	########	########	########
NA	NA	NA	NA	########	########	########	NA	NA
########	########	NA	########	NA	NA	NA	########	NA
NA	NA	NA	NA	NA	NA	NA	NA	NA
NA	NA	NA	NA	NA	NA	NA	NA	NA
########	NA	########	########	########	NA	########	########	########
NA	NA	NA	NA	NA	NA	NA	NA	NA
NA	NA	NA	NA	NA	NA	NA	NA	NA
NA	NA	NA	########	NA	NA	NA	########	NA
NA	NA	NA	########	NA	NA	NA	########	NA
NA	NA	NA	########	NA	NA	NA	########	NA
NA	NA	########	########	NA	NA	########	NA	NA
NA	NA	NA	NA	NA	NA	NA	NA	NA
NA	NA	NA	########	NA	NA	NA	########	NA
NA	NA	NA	########	NA	NA	NA	########	NA
NA	NA	NA	########	NA	NA	NA	########	NA
########	########	########	########	########	########	########	########	########
NA	########	########	NA	NA	########	########	NA	########
########	########	########	########	########	########	########	########	########
NA	NA	########	NA	NA	NA	NA	NA	NA
NA	NA	NA	########	NA	NA	NA	########	NA
NA	NA	NA	########	NA	NA	NA	########	NA
NA	########	########	NA	NA	NA	NA	NA	NA
########	########	########	########	NA	NA	NA	NA	NA
NA	NA	NA	########	NA	NA	NA	########	NA
NA	NA	NA	NA	NA	NA	NA	NA	########
NA	########	########	########	NA	########	########	########	########
NA	NA	NA	########	NA	NA	NA	########	NA
NA	########	########	########	NA	########	########	########	NA
NA	NA	########	########	########	NA	########	########	NA
########	########	########	NA	NA	########	########	NA	NA
NA	NA	NA	########	NA	NA	########	########	NA
NA	########	########	########	NA	########	########	########	########
NA	########	########	########	########	########	########	########	########
NA	########	NA	########	NA	########	NA	########	NA
NA	NA	NA	########	NA	NA	NA	########	NA
NA	NA	NA	########	NA	NA	NA	########	NA
NA	NA	NA	########	NA	NA	NA	########	NA
NA	########	NA	########	NA	NA	NA	########	NA
########	########	########	NA	########	########	########	NA	NA
########	NA	NA	NA	NA	NA	NA	NA	NA
NA	########	########	########	NA	########	########	########	NA
NA	NA	NA	NA	NA	NA	NA	NA	NA
NA	NA	NA	########	NA	NA	NA	########	NA
NA	NA	NA	########	NA	NA	NA	########	NA
NA	NA	NA	########	NA	NA	NA	########	NA
NA	NA	NA	NA	NA	NA	NA	NA	NA
NA	NA	NA	########	NA	NA	NA	########	NA

For Review Only

NA	NA	NA	########	NA	NA	NA	########	NA
NA	NA	NA	########	NA	NA	NA	########	NA
NA	NA	NA	########	NA	NA	NA	########	NA
NA	NA	NA	########	NA	NA	NA	########	NA
NA	NA	NA	########	NA	NA	NA	########	NA
NA	NA	NA	########	NA	NA	NA	########	NA
NA	NA	NA	########	NA	NA	NA	########	NA
NA	NA	NA	########	NA	NA	NA	########	NA
NA	NA	NA	########	NA	NA	NA	########	NA
NA	NA	########	########	NA	NA	########	########	NA
NA	########	########	########	NA	########	########	########	NA
NA	NA	NA	NA	NA	NA	NA	NA	NA
NA	NA	NA	NA	NA	NA	NA	NA	NA
NA	NA	NA	########	NA	NA	NA	########	NA
NA	NA	NA	########	NA	NA	NA	########	NA
NA	NA	NA	########	NA	NA	NA	########	NA
NA	NA	########	########	NA	NA	########	########	NA
NA	NA	NA	########	NA	NA	NA	########	NA
NA	NA	########	NA	NA	NA	########	NA	NA
NA	NA	NA	########	NA	NA	NA	########	NA
NA	NA	########	NA	NA	NA	########	NA	NA
NA	NA	NA	########	NA	NA	NA	########	NA
NA	NA	NA	NA	NA	NA	NA	NA	NA
NA	NA	NA	########	NA	NA	NA	########	NA
NA	NA	NA	NA	NA	NA	NA	########	NA
NA	NA	NA	NA	########	########	########	########	NA
NA	NA	NA	########	NA	NA	NA	########	NA
NA	NA	########	NA	NA	NA	########	NA	NA
NA	NA	NA	########	NA	NA	NA	########	NA
NA	NA	########	NA	NA	NA	########	NA	NA
NA	NA	NA	########	NA	NA	NA	########	NA
NA	NA	NA	########	NA	NA	NA	########	NA
NA	NA	NA	########	NA	NA	NA	########	NA
NA	NA	NA	########	NA	NA	NA	########	NA
NA	NA	NA	NA	NA	NA	NA	NA	NA
NA	NA	NA	NA	NA	NA	NA	########	NA
NA	NA	########	NA	NA	NA	########	NA	NA
NA	NA	########	NA	NA	NA	########	NA	NA
NA	NA	########	NA	NA	NA	########	NA	NA
NA	NA	NA	NA	NA	NA	NA	NA	NA
NA	NA	NA	########	NA	NA	NA	########	NA
NA	NA	NA	########	NA	NA	NA	########	NA
NA	NA	NA	########	NA	NA	NA	########	NA
NA	NA	NA	########	NA	NA	NA	########	NA
NA	NA	NA	########	NA	NA	NA	########	NA
NA	NA	NA	########	NA	NA	NA	########	NA
NA	########	########	NA	NA	########	########	NA	########
NA	NA	NA	########	NA	NA	NA	########	NA
NA	NA	NA	########	NA	NA	NA	########	NA
NA	NA	########	NA	NA	NA	########	NA	NA
NA	NA	########	NA	NA	NA	########	NA	NA
NA	########	########	########	########	########	########	########	########
NA	########	########	########	NA	########	########	########	########
NA	########	########	########	NA	########	########	########	########
NA	########	########	########	########	########	########	########	########
NA	NA	NA	NA	NA	########	########	########	NA

For Review Only

NA	NA	########	NA	NA	NA	########	NA	NA
NA	NA	NA	########	NA	NA	NA	########	NA
########	########	########	NA	########	########	NA	NA	NA
NA	########	########	########	NA	########	########	########	########
NA	NA	NA	########	NA	NA	NA	########	NA
NA	NA	NA	NA	NA	NA	NA	NA	NA
NA	NA	NA	########	NA	NA	NA	########	NA
NA	NA	NA	########	NA	NA	NA	########	########
########	########	########	NA	NA	########	########	NA	NA
NA	########	########	########	########	########	########	########	########
NA	NA	NA	########	NA	NA	NA	########	NA
NA	NA	NA	########	NA	NA	NA	########	NA
NA	NA	NA	########	NA	NA	NA	########	NA
NA	NA	NA	########	NA	NA	NA	########	NA
NA	NA	########	########	NA	NA	########	########	NA
NA	########	########	########	NA	########	########	########	########
NA	NA	NA	########	NA	NA	NA	########	NA
########	########	########	NA	NA	########	########	########	NA
NA	########	########	NA	NA	########	NA	NA	NA
########	########	########	########	########	########	NA	########	NA
########	########	NA	NA	########	########	NA	########	########
########	########	########	########	########	########	########	########	NA
NA	########	########	########	NA	########	########	########	########
NA	########	########	########	NA	########	########	########	NA
NA	########	########	NA	NA	NA	NA	NA	NA
NA	NA	NA	NA	NA	NA	NA	NA	NA
NA	########	########	NA	NA	########	########	NA	NA
########	########	########	########	########	########	########	########	NA
NA	########	########	NA	NA	########	########	NA	########
NA	NA	NA	NA	NA	NA	NA	NA	NA
NA	NA	########	########	NA	NA	NA	########	NA
NA	########	########	########	########	########	########	########	########
NA	NA	NA	########	NA	NA	NA	########	NA
NA	NA	NA	NA	NA	NA	NA	NA	NA
########	########	########	########	########	########	########	########	NA
########	########	########	########	########	########	########	########	########
NA	########	########	NA	NA	NA	########	NA	NA
NA	NA	NA	########	NA	NA	NA	########	NA
NA	NA	NA	NA	NA	NA	NA	NA	NA
NA	########	########	NA	NA	NA	########	NA	NA
NA	NA	########	NA	NA	NA	########	NA	NA
NA	NA	########	NA	NA	NA	########	NA	NA
NA	NA	NA	########	NA	NA	NA	########	NA
NA	NA	NA	NA	NA	NA	NA	NA	NA
NA	NA	NA	########	NA	NA	NA	########	NA
NA	########	########	NA	NA	########	########	NA	NA
NA	NA	########	########	NA	NA	########	########	NA
NA	NA	NA	########	NA	NA	NA	########	NA
NA	NA	NA	########	NA	NA	NA	########	NA
NA	NA	########	########	NA	NA	########	########	NA
NA	NA	########	########	NA	NA	########	########	NA
NA	NA	NA	NA	NA	NA	NA	NA	NA
########	########	########	########	########	########	########	########	########
NA	NA	NA	########	NA	NA	NA	########	NA
NA	NA	NA	########	NA	NA	NA	########	NA
NA	NA	NA	NA	NA	NA	NA	NA	NA

For Review Only

NA	########	########	########	NA	########	NA	########	NA
NA	NA	NA	########	NA	NA	NA	########	NA
NA	NA	########	########	NA	NA	########	########	NA
NA	NA	########	########	NA	NA	NA	########	NA
########	########	########	########	########	########	########	########	NA
NA	NA	NA	NA	NA	NA	NA	NA	NA
########	########	########	NA	########	########	########	########	NA
NA	NA	NA	########	NA	NA	NA	########	NA
NA	NA	NA	########	NA	NA	NA	########	NA
NA	NA	NA	NA	NA	NA	NA	NA	NA
NA	NA	NA	NA	NA	NA	NA	NA	NA
NA	NA	NA	########	NA	NA	NA	########	NA
########	########	NA	NA	########	########	NA	NA	NA
NA	NA	########	NA	NA	NA	########	NA	NA
NA	NA	NA	########	NA	NA	NA	########	NA
NA	NA	NA	########	NA	NA	NA	########	NA
NA	########	########	########	NA	########	########	########	NA
NA	NA	NA	########	NA	NA	NA	########	NA
NA	NA	########	########	NA	NA	########	########	NA
########	########	NA	NA	NA	NA	NA	########	NA
NA	NA	NA	########	NA	NA	NA	########	NA
NA	NA	NA	########	NA	NA	NA	########	NA
NA	NA	NA	########	NA	NA	NA	########	NA
NA	NA	NA	########	NA	NA	NA	########	NA
########	########	########	########	NA	########	########	########	NA
NA	NA	NA	########	NA	NA	NA	########	NA
NA	NA	NA	NA	NA	NA	NA	NA	NA
NA	NA	NA	NA	NA	NA	NA	NA	NA
########	########	########	########	########	########	########	########	########
NA	NA	NA	NA	NA	NA	NA	########	NA
########	########	########	NA	########	########	########	NA	########
NA	NA	NA	NA	NA	NA	NA	NA	NA
NA	########	########	########	NA	########	########	########	########
NA	NA	NA	NA	NA	NA	NA	NA	NA
########	########	########	NA	########	########	########	NA	########
NA	NA	NA	NA	NA	NA	NA	NA	NA
NA	########	########	########	NA	########	########	########	########
NA	NA	NA	########	NA	NA	NA	########	NA
NA	NA	NA	NA	NA	NA	NA	NA	NA
NA	NA	NA	NA	NA	NA	NA	NA	NA
########	########	########	########	########	########	########	########	########
NA	NA	NA	NA	NA	NA	NA	NA	NA
NA	NA	NA	NA	NA	NA	NA	NA	NA
NA	########	########	########	########	########	########	########	########
NA	NA	NA	NA	NA	NA	NA	NA	NA
NA	NA	########	NA	NA	NA	########	NA	NA
NA	NA	########	NA	NA	NA	########	NA	NA
NA	NA	NA	########	NA	NA	NA	########	NA
NA	########	########	NA	NA	########	########	NA	NA
########	########	########	########	########	########	########	########	NA
NA	########	########	########	NA	########	########	########	NA
NA	########	########	NA	NA	NA	########	NA	NA
NA	NA	########	NA	NA	NA	########	NA	NA

For Review Only

NA	NA	NA	########	NA	NA	NA	########	NA
########	NA	########	########	########	NA	########	########	########
NA	NA	NA	########	NA	NA	NA	########	NA
NA	########	########	########	NA	########	########	########	NA
NA	NA	NA	########	NA	NA	NA	########	NA
NA	NA	NA	########	NA	NA	NA	########	NA
NA	NA	NA	########	NA	NA	NA	########	NA
NA	########	########	########	NA	########	########	########	NA
NA	NA	NA	########	NA	NA	NA	########	NA
NA	NA	NA	########	NA	NA	NA	########	NA
NA	########	########	NA	NA	########	########	NA	########
NA	NA	########	NA	NA	NA	########	NA	NA
NA	########	########	########	NA	########	########	########	########
NA	NA	########	########	NA	NA	########	########	NA
########	########	########	########	########	########	########	########	########
NA	NA	NA	########	NA	NA	NA	########	NA
NA	########	########	########	########	########	########	########	########
NA	NA	NA	NA	NA	NA	NA	NA	NA
NA	NA	NA	########	NA	NA	NA	########	NA
NA	NA	NA	########	NA	NA	NA	########	NA
NA	########	########	########	NA	########	########	########	NA
NA	NA	NA	NA	NA	NA	NA	NA	NA
NA	NA	NA	NA	NA	NA	NA	NA	NA
NA	NA	NA	NA	NA	NA	NA	NA	NA
NA	NA	NA	########	NA	NA	NA	########	NA
NA	NA	########	NA	NA	NA	########	NA	NA
NA	NA	NA	########	NA	NA	NA	########	NA
NA	NA	NA	########	NA	NA	NA	########	NA
NA	NA	NA	########	NA	NA	NA	########	NA
########	########	########	########	########	########	########	########	########
NA	NA	NA	########	NA	NA	NA	########	NA
NA	NA	NA	########	NA	NA	NA	########	NA
NA	NA	NA	########	NA	NA	NA	########	NA
NA	########	########	NA	NA	########	########	NA	NA
NA	NA	########	NA	NA	NA	########	NA	NA
NA	NA	NA	NA	NA	NA	NA	NA	NA
########	########	NA	########	########	########	########	########	########
NA	NA	NA	########	NA	NA	NA	########	NA
########	########	########	########	########	########	########	########	########
########	NA	NA	NA	NA	NA	NA	NA	########
NA	NA	########	NA	NA	NA	########	NA	NA
NA	NA	NA	########	NA	NA	NA	########	NA
NA	NA	NA	NA	NA	NA	NA	NA	NA
NA	NA	NA	########	NA	NA	NA	########	NA
########	########	########	########	########	########	########	########	########
NA	NA	########	NA	NA	NA	########	NA	NA
NA	NA	########	NA	NA	NA	########	NA	NA
########	########	NA	NA	NA	########	NA	########	NA
NA	NA	NA	NA	NA	NA	NA	NA	NA
NA	NA	NA	NA	NA	NA	NA	########	NA
NA	NA	NA	########	NA	NA	NA	########	NA
########	########	########	NA	NA	########	########	NA	########
########	########	########	########	NA	########	########	########	NA
########	########	########	########	########	########	########	########	########
########	########	########	########	########	########	########	########	########
NA	########	########	NA	NA	########	########	NA	########

For Review Only

NA	NA	NA	########	NA	NA	NA	########	NA
NA	NA	NA	NA	NA	NA	NA	NA	NA
NA	########	########	########	NA	########	########	########	NA
NA	NA	########	NA	NA	NA	########	NA	NA
NA	NA	NA	NA	NA	NA	NA	########	NA
NA	NA	NA	NA	NA	NA	NA	NA	NA
########	########	########	########	########	########	########	########	NA
NA	NA	NA	########	NA	NA	NA	########	NA
NA	########	########	########	NA	########	NA	########	NA
NA	########	########	########	########	########	########	########	########
########	########	NA	NA	NA	NA	NA	########	NA
########	########	NA	########	NA	########	NA	########	NA
NA	NA	NA	########	NA	NA	NA	########	NA
NA	########	########	########	NA	NA	NA	########	NA
NA	NA	NA	NA	NA	NA	NA	NA	NA
########	########	########	########	########	########	########	########	NA
NA	NA	NA	NA	NA	NA	NA	NA	NA
########	########	########	NA	########	NA	NA	NA	NA
NA	NA	NA	NA	NA	NA	NA	NA	NA
NA	########	NA	NA	NA	NA	NA	NA	NA
########	########	########	########	########	########	########	########	########
########	########	########	########	########	########	########	########	########
NA	NA	NA	########	NA	NA	NA	NA	NA
NA	NA	NA	NA	NA	NA	NA	NA	NA
NA	NA	########	########	NA	NA	########	########	NA
NA	NA	NA	########	NA	NA	NA	########	NA
########	########	########	########	########	########	########	########	NA
NA	NA	NA	NA	NA	NA	NA	NA	NA
########	########	########	########	########	########	########	########	NA
NA	########	########	########	NA	########	########	########	NA
NA	NA	NA	########	NA	NA	NA	########	NA
NA	NA	NA	NA	NA	NA	NA	NA	NA
NA	NA	NA	NA	NA	NA	NA	NA	NA
NA	########	########	########	########	########	########	########	########
########	########	########	NA	########	NA	NA	NA	NA
NA	NA	NA	NA	NA	NA	NA	NA	NA
NA	NA	########	########	NA	NA	NA	########	NA
NA	NA	########	########	NA	########	########	########	NA
NA	NA	########	NA	NA	NA	########	NA	NA
NA	########	########	########	NA	########	########	########	########
NA	NA	########	NA	NA	NA	########	NA	NA
########	########	NA	NA	########	########	NA	NA	NA
NA	NA	NA	########	NA	NA	NA	########	NA
NA	NA	NA	########	NA	NA	NA	########	NA
NA	NA	########	NA	NA	NA	########	NA	NA
NA	NA	NA	########	NA	NA	NA	########	NA
NA	NA	NA	########	NA	NA	NA	########	NA
NA	NA	NA	########	NA	NA	NA	########	NA
########	########	########	########	########	########	########	########	NA
NA	NA	########	NA	NA	NA	########	NA	NA
NA	NA	NA	########	NA	NA	NA	########	NA
NA	NA	NA	########	NA	NA	NA	########	NA
NA	NA	########	########	NA	NA	########	########	NA
NA	NA	NA	########	NA	NA	NA	########	NA
NA	NA	NA	########	NA	NA	NA	########	NA
NA	########	########	NA	NA	########	########	NA	NA

For Review Only

########	NA	NA	NA	NA	NA	NA	NA	NA
NA	NA	NA	########	NA	NA	NA	########	NA
NA	NA	########	NA	NA	NA	NA	NA	NA
########	########	########	########	########	########	########	########	########
########	########	########	########	########	########	########	########	########
NA	NA	NA	NA	NA	NA	NA	NA	NA
NA	NA	NA	NA	NA	NA	NA	NA	NA
NA	########	########	########	NA	########	########	########	NA
NA	NA	########	########	NA	NA	########	########	NA
########	########	NA	########	########	########	NA	########	NA
NA	NA	NA	########	NA	NA	NA	NA	NA
########	########	########	########	########	########	########	########	########
NA	NA	NA	NA	NA	NA	NA	NA	NA
NA	########	########	########	NA	########	########	########	NA
########	########	########	NA	########	########	########	NA	NA
NA	NA	NA	########	NA	NA	########	########	########
########	########	########	########	########	########	########	########	########
NA	NA	NA	NA	NA	NA	NA	NA	NA
NA	NA	NA	########	NA	NA	NA	########	NA
NA	########	NA	########	NA	########	NA	########	NA
NA	NA	NA	NA	NA	NA	NA	NA	NA
NA	NA	NA	########	NA	NA	NA	########	NA
NA	########	########	NA	NA	NA	########	NA	########
NA	NA	NA	NA	NA	NA	NA	NA	NA
NA	NA	########	########	NA	########	########	NA	NA
NA	NA	NA	########	NA	NA	NA	########	NA
NA	NA	########	NA	NA	NA	########	NA	NA
NA	########	########	########	NA	########	########	########	########
NA	NA	########	NA	NA	NA	NA	NA	NA
NA	NA	NA	########	NA	NA	NA	########	NA
NA	NA	NA	NA	NA	NA	NA	NA	NA
NA	NA	NA	NA	########	########	########	########	NA
NA	NA	NA	########	NA	########	########	NA	NA
NA	NA	NA	########	NA	NA	NA	########	NA
NA	NA	NA	NA	NA	NA	NA	NA	NA
NA	NA	########	NA	NA	NA	########	NA	NA
########	NA	NA	########	########	NA	NA	########	NA
NA	NA	########	########	NA	NA	########	########	NA
NA	NA	NA	NA	NA	NA	NA	NA	NA
NA	NA	NA	########	NA	NA	NA	########	NA
NA	########	NA	########	NA	NA	NA	########	NA
NA	NA	NA	########	NA	NA	NA	########	NA
NA	NA	NA	NA	NA	NA	NA	NA	NA
NA	NA	NA	NA	NA	NA	NA	NA	NA
NA	########	NA	########	NA	########	NA	########	NA
########	########	NA	########	########	########	NA	########	NA
NA	NA	NA	NA	NA	NA	NA	NA	NA
########	########	########	NA	########	########	NA	NA	NA
NA	NA	NA	NA	NA	NA	NA	NA	NA
NA	NA	NA	NA	NA	NA	NA	NA	NA
NA	########	########	########	NA	########	########	########	NA

For Review Only

NA	NA	NA	########	NA	NA	NA	########	NA
NA	NA	NA	########	NA	NA	NA	########	NA
NA	########	########	########	NA	NA	########	########	NA
NA	NA	NA	NA	NA	NA	NA	NA	NA
NA	NA	NA	########	NA	NA	NA	########	NA
NA	NA	NA	########	NA	NA	########	########	NA
NA	NA	########	########	NA	NA	########	########	NA
NA	NA	########	########	NA	NA	########	########	NA
NA	########	########	########	NA	########	########	########	NA
NA	NA	########	NA	NA	NA	########	NA	NA
NA	########	########	########	NA	########	########	########	########
NA	########	########	NA	NA	NA	########	NA	NA
NA	NA	NA	########	NA	NA	NA	########	NA
NA	NA	NA	########	NA	NA	NA	########	NA
########	########	########	########	NA	########	########	########	NA
NA	########	########	NA	NA	NA	########	NA	NA
NA	NA	NA	########	NA	NA	NA	########	NA
########	########	########	########	########	########	########	########	########
NA	NA	NA	########	NA	NA	NA	########	NA
NA	########	########	########	NA	########	########	########	########
NA	NA	NA	########	NA	NA	NA	########	NA
NA	NA	NA	########	NA	NA	NA	########	NA
########	########	########	########	########	########	########	########	########
########	########	########	########	########	########	########	########	########
########	########	NA	NA	NA	########	NA	########	NA
NA	NA	NA	########	NA	NA	NA	########	NA
NA	NA	NA	NA	NA	NA	NA	NA	NA
NA	NA	NA	########	NA	NA	NA	########	NA
NA	NA	NA	NA	NA	NA	NA	NA	NA
NA	NA	NA	########	NA	NA	NA	########	NA
NA	NA	NA	########	NA	NA	NA	########	NA
NA	########	########	########	NA	########	########	NA	########
NA	NA	########	NA	NA	NA	########	NA	NA
NA	NA	NA	########	NA	NA	NA	########	NA
NA	NA	NA	########	NA	NA	NA	########	NA
NA	NA	NA	########	NA	NA	NA	########	NA
NA	NA	NA	########	NA	NA	NA	NA	NA
NA	########	NA	########	NA	########	NA	########	NA
NA	########	########	########	NA	NA	########	########	NA
NA	NA	########	NA	NA	NA	########	NA	NA
########	########	########	########	########	########	########	########	########
NA	NA	NA	NA	NA	NA	NA	NA	NA
NA	NA	NA	NA	NA	NA	NA	NA	NA
NA	NA	NA	########	NA	NA	NA	########	NA
NA	NA	NA	NA	NA	NA	NA	NA	NA
NA	NA	########	NA	NA	NA	NA	NA	NA
NA	NA	NA	NA	NA	NA	NA	NA	NA
NA	NA	NA	NA	NA	NA	########	NA	NA
NA	NA	NA	########	NA	NA	NA	########	NA
NA	NA	NA	########	NA	NA	NA	########	NA
NA	NA	NA	NA	NA	NA	NA	NA	NA
NA	NA	########	########	NA	NA	########	########	########
NA	NA	NA	########	NA	########	########	########	NA
NA	NA	########	########	NA	NA	NA	########	NA
NA	NA	NA	########	NA	NA	NA	########	NA
NA	########	########	NA	NA	########	########	NA	########

For Review Only

NA	NA	NA	########	NA	NA	NA	########	NA
NA	########	########	########	NA	########	########	########	########
NA	NA	########	NA	NA	NA	########	NA	NA
########	########	########	########	########	########	########	########	########
NA	NA	########	NA	NA	NA	########	NA	NA
NA	########	########	########	########	########	########	########	########
########	########	########	########	NA	########	NA	########	NA
NA	NA	NA	NA	NA	NA	NA	NA	NA
########	########	########	########	########	########	########	########	NA
NA	NA	NA	NA	NA	NA	NA	NA	NA
NA	NA	NA	########	NA	NA	NA	########	NA
NA	NA	NA	NA	NA	NA	NA	NA	NA
NA	########	########	########	NA	NA	########	########	NA
########	NA	NA	NA	########	NA	########	NA	########
NA	NA	NA	NA	NA	NA	NA	NA	NA
NA	########	########	NA	########	########	########	########	NA
NA	########	########	NA	########	########	########	NA	NA
NA	NA	NA	########	NA	NA	NA	########	NA
NA	NA	NA	########	NA	NA	NA	########	NA
NA	NA	########	NA	NA	NA	NA	NA	NA
########	########	########	########	########	########	########	########	########
NA	NA	NA	NA	NA	NA	NA	NA	NA
NA	NA	NA	NA	NA	NA	NA	NA	NA
NA	NA	NA	########	########	########	########	########	NA
NA	NA	NA	NA	NA	NA	NA	NA	NA
NA	NA	NA	########	NA	NA	NA	########	NA
NA	NA	########	NA	NA	NA	NA	NA	NA
NA	NA	NA	########	NA	NA	########	########	NA
########	########	NA	########	########	NA	NA	########	NA
NA	NA	NA	########	NA	NA	NA	########	NA
########	########	########	NA	########	########	########	NA	########
NA	NA	NA	########	NA	NA	########	########	NA
NA	NA	########	NA	NA	NA	########	NA	NA
NA	NA	NA	########	NA	NA	NA	########	NA
NA	########	########	########	NA	########	########	########	NA
NA	NA	NA	NA	NA	NA	NA	NA	NA
NA	########	########	########	NA	########	########	########	NA
NA	########	########	NA	NA	NA	########	########	NA
NA	NA	NA	########	NA	NA	NA	########	NA
NA	NA	NA	########	NA	NA	NA	########	NA
NA	NA	########	########	NA	NA	########	########	NA
NA	NA	NA	########	NA	NA	NA	########	NA
NA	########	########	########	NA	########	########	########	########
NA	########	NA	########	NA	NA	NA	NA	NA
NA	NA	NA	NA	NA	NA	NA	NA	NA
NA	NA	########	NA	NA	NA	########	NA	NA
NA	NA	NA	NA	NA	########	########	########	NA
NA	NA	NA	NA	NA	NA	NA	NA	NA
NA	NA	NA	NA	NA	NA	NA	NA	NA
########	########	########	########	########	########	########	########	########
########	########	########	########	NA	NA	########	########	NA
NA	NA	NA	########	NA	NA	NA	########	NA
########	########	########	########	########	########	########	########	NA
NA	NA	NA	NA	NA	NA	NA	NA	NA
NA	NA	NA	########	NA	NA	NA	########	NA
NA	NA	NA	########	NA	NA	NA	########	NA

For Review Only

NA	NA	########	NA	NA	NA	########	NA	NA
NA	NA	########	########	NA	NA	########	########	NA
NA	########	########	########	NA	########	########	########	########
########	########	########	########	########	########	########	########	########
NA	NA	NA	########	NA	NA	NA	########	NA
NA	########	########	########	NA	NA	########	########	NA
NA	########	########	########	NA	########	########	########	########
NA	NA	########	########	NA	NA	########	########	NA
NA	NA	########	########	NA	NA	########	########	NA
NA	NA	NA	NA	NA	########	NA	NA	NA
NA	NA	########	########	NA	NA	########	########	NA
NA	NA	NA	########	NA	NA	NA	########	NA
########	########	########	########	NA	########	########	########	########
NA	NA	########	########	NA	NA	########	########	NA
NA	########	########	NA	NA	NA	########	NA	NA
NA	NA	NA	NA	NA	NA	NA	NA	NA
NA	NA	NA	########	NA	########	########	NA	NA
NA	NA	NA	NA	NA	NA	NA	NA	NA
NA	NA	NA	########	NA	NA	NA	########	NA
########	NA	########	NA	NA	NA	NA	NA	NA
NA	NA	########	NA	NA	NA	########	NA	NA
########	NA	########	########	########	NA	########	########	########
NA	NA	NA	NA	NA	NA	NA	NA	NA
########	########	########	########	########	########	########	########	NA
NA	########	########	########	########	########	########	########	########
NA	NA	NA	NA	NA	NA	NA	########	NA
NA	NA	########	NA	NA	NA	NA	NA	NA
NA	NA	NA	NA	NA	NA	NA	NA	NA
NA	NA	########	NA	NA	NA	########	NA	NA
NA	NA	NA	NA	NA	NA	NA	NA	NA
NA	########	########	NA	NA	NA	########	NA	NA
NA	NA	########	NA	NA	NA	########	NA	NA
NA	NA	########	NA	NA	NA	########	NA	NA
########	########	NA	########	########	########	NA	########	NA
NA	NA	########	NA	NA	NA	########	NA	NA
########	########	########	########	########	########	NA	########	NA
NA	NA	NA	########	NA	NA	NA	NA	NA
NA	########	########	########	NA	########	########	########	NA
NA	NA	########	########	NA	NA	########	########	NA
NA	NA	########	########	NA	NA	########	########	NA
NA	NA	########	########	NA	NA	########	########	NA
NA	NA	NA	########	NA	NA	NA	########	NA
########	NA	NA	########	########	NA	########	########	########
########	########	########	NA	NA	NA	NA	NA	NA
########	########	########	########	########	########	########	########	########
NA	NA	NA	########	NA	NA	NA	########	NA
NA	NA	########	NA	NA	NA	########	NA	NA
NA	NA	########	########	NA	NA	########	########	NA
NA	########	########	NA	NA	NA	########	NA	NA
NA	NA	NA	NA	NA	NA	NA	NA	NA
NA	NA	########	########	NA	NA	########	########	NA
NA	NA	NA	########	NA	NA	NA	########	NA
NA	########	########	########	NA	########	########	########	NA
NA	########	########	NA	NA	NA	########	NA	NA
NA	NA	NA	NA	NA	NA	NA	NA	NA
NA	NA	NA	NA	NA	NA	NA	NA	########

For Review Only

########	########	NA	NA	NA	########	NA	NA	NA
NA	########	########	########	NA	########	########	########	########
NA	NA	NA	########	NA	########	########	########	NA
########	########	NA	NA	NA	########	NA	NA	NA
NA	NA	NA	########	NA	NA	NA	########	NA
NA	NA	NA	NA	NA	NA	NA	NA	NA
NA	########	########	NA	########	########	########	NA	NA
NA	NA	NA	########	NA	NA	NA	########	NA
########	########	########	NA	########	########	########	########	########
########	########	########	########	########	########	########	########	########
NA	NA	########	NA	NA	NA	########	NA	NA
########	########	########	NA	########	########	########	NA	########
NA	NA	NA	########	NA	NA	NA	########	NA
NA	NA	NA	########	NA	NA	NA	########	NA
########	########	########	########	########	########	NA	########	NA
NA	NA	########	########	NA	NA	NA	NA	NA
NA	NA	NA	NA	########	########	########	########	NA
NA	NA	########	NA	NA	NA	########	NA	NA
NA	########	########	NA	NA	########	########	NA	NA
NA	NA	########	NA	NA	NA	########	NA	NA
NA	NA	NA	########	NA	NA	NA	########	NA
NA	NA	NA	########	NA	NA	NA	########	NA
NA	########	########	########	NA	########	########	########	NA
NA	NA	NA	NA	NA	NA	NA	NA	NA
NA	NA	NA	########	NA	NA	NA	########	NA
NA	########	########	########	NA	########	########	########	########
NA	NA	NA	########	NA	NA	NA	########	NA
NA	NA	NA	########	NA	NA	NA	########	NA
########	########	########	########	########	########	########	########	NA
NA	NA	NA	NA	NA	NA	NA	NA	NA
########	########	########	########	########	########	########	########	########
NA	NA	NA	NA	NA	NA	NA	NA	NA
NA	NA	NA	########	NA	########	NA	########	NA
NA	NA	NA	########	NA	NA	NA	########	NA
NA	NA	NA	########	NA	NA	NA	########	NA
NA	NA	NA	########	NA	NA	NA	########	NA
########	########	########	########	########	########	########	########	########
########	########	########	########	########	########	########	########	NA
NA	NA	NA	########	NA	NA	NA	########	NA
NA	NA	NA	NA	NA	NA	NA	NA	NA
NA	NA	NA	NA	NA	NA	NA	NA	NA
NA	########	########	########	NA	########	########	########	########
NA	NA	NA	NA	NA	NA	NA	NA	NA
NA	NA	NA	########	NA	NA	NA	########	NA
NA	########	########	########	NA	NA	NA	########	NA
NA	NA	NA	########	NA	NA	NA	########	NA
NA	########	NA	########	NA	########	NA	########	NA
########	########	########	########	NA	########	########	########	NA
NA	NA	########	########	NA	NA	########	########	NA
NA	NA	NA	NA	NA	NA	NA	NA	NA
NA	NA	NA	########	NA	NA	NA	########	NA
NA	NA	########	NA	NA	NA	########	NA	NA
NA	NA	NA	NA	NA	NA	NA	########	NA
NA	NA	NA	NA	NA	NA	NA	NA	NA
########	########	########	NA	########	########	########	########	NA
NA	NA	NA	NA	NA	NA	NA	NA	NA

For Review Only

NA	NA	NA	NA	NA	########	########	########	NA
NA	NA	NA	########	NA	NA	NA	########	NA
NA	NA	NA	########	NA	########	NA	########	NA
NA	NA	NA	########	NA	NA	NA	########	NA
NA	NA	NA	########	NA	NA	NA	########	NA
NA	NA	NA	NA	NA	NA	NA	NA	NA
########	########	########	########	########	########	########	########	########
########	########	########	########	########	########	########	########	########
NA	NA	NA	########	NA	NA	NA	########	NA
NA	NA	########	NA	NA	NA	########	NA	NA
NA	NA	NA	NA	NA	NA	NA	NA	NA
NA	NA	########	NA	NA	NA	########	NA	NA
NA	NA	NA	########	NA	NA	NA	########	NA
NA	NA	NA	NA	NA	NA	NA	NA	NA
NA	########	########	########	NA	NA	NA	########	NA
NA	NA	########	########	NA	NA	########	########	NA
NA	########	########	NA	NA	########	########	NA	NA
NA	########	########	########	NA	NA	########	########	NA
########	NA	NA	########	########	NA	NA	########	NA
########	########	########	########	########	########	########	########	NA
NA	########	########	NA	NA	########	########	NA	########
########	########	########	########	NA	########	########	NA	########
########	########	########	########	########	########	########	########	########
NA	NA	NA	NA	NA	NA	NA	NA	NA
NA	NA	NA	NA	NA	NA	NA	NA	NA
NA	NA	NA	########	NA	NA	NA	########	NA
NA	NA	NA	########	NA	NA	NA	########	NA
NA	########	########	########	NA	########	########	########	NA
NA	NA	NA	NA	NA	NA	NA	NA	NA
NA	########	########	NA	NA	########	########	NA	NA
NA	NA	NA	########	NA	NA	########	########	NA
NA	NA	NA	NA	NA	NA	NA	NA	NA
NA	NA	NA	########	NA	NA	NA	########	NA
########	########	########	########	########	########	########	########	########
NA	NA	########	NA	NA	NA	########	NA	NA
NA	########	########	NA	NA	NA	########	NA	NA
########	########	########	########	########	########	########	########	########
NA	########	NA	########	NA	NA	NA	########	NA
NA	NA	NA	NA	NA	NA	NA	NA	NA
NA	NA	NA	########	NA	NA	########	########	NA
NA	NA	NA	NA	NA	NA	NA	NA	NA
########	########	########	########	########	########	########	########	########
NA	NA	NA	########	NA	NA	NA	########	NA
NA	########	########	NA	NA	NA	########	########	NA
NA	########	########	NA	NA	NA	########	NA	NA
NA	NA	NA	NA	NA	NA	NA	NA	NA
NA	NA	NA	########	NA	NA	NA	########	NA
NA	NA	NA	########	NA	NA	NA	########	NA
NA	NA	NA	########	NA	NA	NA	########	NA
########	########	########	########	########	########	########	########	NA
NA	NA	NA	NA	NA	NA	NA	NA	NA
NA	NA	NA	NA	NA	NA	NA	NA	NA
NA	NA	NA	NA	NA	NA	########	NA	NA
########	########	########	########	########	########	########	########	########
NA	NA	NA	NA	NA	NA	NA	NA	NA
NA	########	########	NA	NA	NA	########	NA	NA

For review only

NA	########	########	NA	NA	NA	########	NA	NA
NA	NA	NA	NA	NA	NA	NA	NA	NA
NA	########	########	########	########	########	########	########	########
########	########	NA	NA	########	########	NA	########	NA
NA	NA	NA	########	NA	NA	NA	########	NA
NA	NA	NA	########	NA	NA	NA	########	NA
NA	NA	########	########	NA	NA	########	########	NA
NA	NA	NA	NA	NA	NA	NA	NA	NA
NA	########	########	########	NA	########	########	########	NA
NA	NA	########	NA	NA	NA	########	NA	NA
########	NA	NA	########	NA	NA	NA	########	NA
########	NA	NA	NA	########	NA	NA	########	########
NA	NA	NA	NA	NA	NA	NA	NA	NA
NA	NA	NA	########	NA	NA	NA	########	NA
########	########	########	########	########	########	########	########	NA
NA	########	########	########	########	########	########	########	########
NA	NA	########	NA	NA	NA	########	NA	NA
NA	NA	########	########	NA	NA	NA	NA	NA
NA	NA	NA	########	NA	NA	NA	########	NA
NA	########	########	########	NA	NA	########	########	NA
NA	NA	NA	NA	NA	NA	NA	NA	NA
NA	NA	NA	########	NA	NA	NA	########	NA
########	########	########	NA	NA	NA	########	NA	NA
NA	NA	########	NA	NA	NA	########	NA	NA
NA	########	NA	NA	NA	########	NA	NA	NA
NA	NA	NA	NA	NA	NA	NA	NA	NA
NA	NA	NA	########	NA	NA	NA	NA	NA
NA	########	########	########	########	########	########	########	NA
NA	NA	NA	NA	NA	NA	NA	NA	NA
NA	NA	NA	########	NA	NA	NA	########	NA
########	########	########	########	########	########	########	########	NA
########	########	########	########	########	########	########	########	NA
NA	NA	NA	NA	NA	NA	NA	NA	NA
########	NA	NA	########	NA	NA	########	########	########
NA	########	########	########	NA	########	########	########	########
NA	########	NA	NA	NA	########	NA	NA	NA
NA	NA	NA	########	NA	NA	NA	########	NA
NA	NA	########	NA	NA	NA	########	NA	NA
########	########	########	########	########	########	########	########	NA
NA	NA	NA	########	NA	NA	NA	########	NA
NA	########	########	NA	NA	########	########	NA	NA
########	########	NA	########	NA	########	NA	########	NA
NA	########	########	########	NA	NA	########	########	NA
########	########	########	########	########	########	########	########	NA
########	########	########	########	########	########	########	########	########
########	########	########	########	########	########	########	########	NA
NA	NA	NA	NA	NA	NA	NA	NA	NA
NA	########	########	NA	NA	NA	########	NA	NA
########	########	########	########	########	########	########	########	########
NA	NA	NA	NA	NA	NA	NA	NA	NA
NA	########	########	########	########	########	########	########	########
NA	NA	NA	NA	NA	NA	NA	NA	NA
NA	NA	NA	NA	NA	NA	########	########	NA
NA	NA	NA	NA	NA	NA	NA	NA	NA
NA	NA	########	########	NA	NA	########	########	NA
########	########	########	########	########	########	########	########	########

For Review Only

NA	NA	NA	########	NA	NA	NA	########	NA
NA	########	########	########	NA	########	########	########	########
NA	########	########	NA	NA	########	########	NA	NA
NA	NA	########	########	NA	NA	########	########	NA
NA	NA	########	########	NA	NA	########	########	NA
NA	NA	NA	########	NA	NA	NA	NA	NA
NA	NA	NA	NA	NA	NA	########	NA	NA
NA	NA	########	NA	NA	########	########	########	NA
NA	NA	NA	########	NA	NA	NA	########	NA
NA	NA	NA	NA	NA	NA	NA	NA	NA
NA	NA	########	########	NA	NA	########	########	NA
NA	NA	########	NA	NA	NA	########	NA	NA
NA	########	########	NA	NA	NA	########	NA	NA
NA	NA	########	NA	NA	NA	########	NA	NA
NA	NA	########	NA	NA	NA	########	NA	NA
NA	NA	########	NA	NA	NA	########	NA	NA
NA	########	########	NA	NA	########	########	NA	NA
NA	NA	########	NA	NA	NA	########	NA	NA
NA	NA	########	NA	NA	NA	########	NA	NA
NA	NA	NA	########	NA	NA	NA	########	NA
NA	NA	NA	NA	NA	NA	NA	NA	NA
NA	NA	NA	########	NA	NA	NA	########	NA
NA	NA	NA	NA	NA	NA	NA	NA	NA
NA	NA	NA	########	NA	NA	NA	########	NA
NA	NA	########	########	NA	NA	########	########	NA
NA	NA	NA	########	NA	NA	NA	########	NA
NA	NA	NA	NA	NA	NA	NA	########	NA
########	########	########	########	NA	########	########	########	NA
########	NA	NA	########	########	NA	NA	########	########
NA	NA	########	NA	NA	NA	########	NA	NA
########	########	########	########	########	########	########	########	########
########	########	########	########	########	########	########	########	########
########	########	NA	NA	NA	########	NA	NA	NA
NA	NA	NA	########	NA	NA	NA	########	NA
NA	NA	NA	########	NA	NA	NA	########	NA
NA	NA	NA	NA	NA	NA	NA	NA	NA
NA	NA	NA	NA	NA	NA	NA	########	NA
NA	NA	NA	NA	NA	NA	NA	NA	NA
NA	NA	NA	########	NA	NA	NA	########	NA
NA	NA	NA	NA	NA	NA	NA	NA	NA
NA	NA	NA	########	NA	NA	NA	########	NA
NA	########	NA	########	NA	########	NA	########	NA
NA	NA	########	########	NA	NA	########	########	NA
NA	########	NA	NA	NA	########	NA	NA	########
NA	########	########	########	########	########	########	########	########
NA	NA	NA	########	NA	NA	NA	########	NA
NA	NA	########	NA	NA	NA	########	NA	NA
NA	NA	NA	########	NA	NA	NA	########	NA
NA	NA	########	NA	NA	NA	########	NA	NA
NA	NA	NA	NA	NA	NA	NA	NA	NA
NA	NA	NA	########	NA	NA	NA	########	NA
NA	NA	NA	NA	NA	NA	NA	NA	NA
NA	NA	########	########	NA	NA	########	########	NA
NA	NA	NA	########	NA	NA	########	########	NA
########	########	########	########	########	########	########	########	########
NA	NA	NA	NA	NA	NA	NA	########	NA

For Review Only

NA	NA	NA	########	NA	NA	NA	########	NA
NA	NA	NA	########	NA	NA	NA	########	NA
NA	NA	########	########	NA	NA	NA	########	NA
NA	NA	NA	########	NA	NA	NA	########	NA
NA	########	########	########	NA	########	########	########	########
NA	NA	NA	NA	NA	NA	NA	########	NA
NA	NA	########	########	NA	NA	########	########	NA
NA	NA	########	########	NA	NA	########	########	NA
NA	NA	########	########	########	########	########	########	NA
NA	NA	NA	NA	NA	NA	NA	NA	NA
########	########	########	NA	########	########	NA	########	NA
NA	NA	NA	########	NA	NA	NA	########	NA
NA	NA	NA	########	NA	NA	NA	########	NA
NA	########	########	########	NA	########	########	########	NA
NA	########	########	########	NA	########	########	########	########
########	########	########	########	########	########	########	########	########
NA	NA	NA	NA	NA	NA	NA	NA	NA
NA	NA	########	########	NA	NA	NA	########	NA
NA	NA	NA	########	NA	NA	NA	########	NA
NA	NA	NA	########	NA	NA	NA	########	NA
NA	NA	NA	########	NA	NA	NA	########	NA
NA	NA	NA	########	NA	NA	NA	########	NA
NA	NA	NA	NA	NA	NA	NA	########	NA
NA	NA	########	NA	NA	NA	########	########	NA
########	########	########	########	########	########	########	########	########
NA	NA	NA	NA	NA	NA	NA	NA	NA
########	########	########	########	########	########	########	########	NA
NA	NA	NA	########	NA	NA	NA	########	NA
NA	NA	NA	NA	NA	NA	NA	NA	NA
########	########	NA	NA	NA	NA	NA	########	NA
NA	NA	NA	########	NA	NA	NA	########	NA
NA	NA	NA	########	NA	NA	NA	########	NA
NA	########	NA	NA	NA	########	NA	NA	NA
NA	NA	NA	########	NA	NA	NA	########	NA
NA	NA	########	########	NA	########	########	########	########
NA	NA	NA	########	NA	NA	NA	########	NA
NA	NA	NA	NA	NA	NA	NA	NA	NA
########	########	########	########	NA	NA	NA	########	NA
NA	########	########	NA	NA	NA	########	NA	NA
NA	NA	########	NA	NA	NA	########	NA	NA
NA	NA	########	########	NA	NA	########	########	NA
########	########	########	########	########	########	########	########	########
NA	########	########	NA	NA	########	########	NA	NA
########	########	NA	########	########	########	NA	########	NA
NA	NA	NA	NA	NA	NA	NA	NA	NA
NA	NA	NA	########	NA	NA	NA	########	NA
NA	NA	NA	########	NA	NA	NA	########	NA
NA	NA	NA	NA	NA	NA	NA	NA	NA
NA	NA	NA	NA	NA	NA	NA	NA	NA
NA	NA	NA	NA	NA	NA	NA	NA	NA
########	########	########	########	########	########	########	########	########
NA	NA	NA	NA	NA	NA	NA	NA	NA
NA	NA	NA	NA	NA	NA	NA	NA	NA
NA	NA	########	NA	NA	NA	########	NA	NA
NA	NA	NA	NA	NA	NA	NA	NA	NA
NA	NA	NA	NA	NA	NA	NA	NA	NA

For Review Only

########	########	NA	NA	########	########	NA	########	NA
########	########	########	NA	########	########	########	NA	########
########	NA	########	########	########	NA	########	########	########
NA	NA	NA	########	NA	NA	NA	########	NA
NA	########	########	########	########	########	########	########	NA
NA	NA	NA	########	NA	NA	NA	########	NA
NA	NA	NA	NA	NA	NA	NA	NA	NA
NA	NA	NA	NA	NA	NA	NA	NA	NA
NA	NA	NA	########	NA	NA	NA	########	########
NA	NA	NA	########	NA	NA	NA	########	NA
NA	NA	########	NA	NA	NA	########	NA	NA
NA	NA	NA	NA	NA	NA	NA	NA	NA
########	########	########	########	########	########	########	########	########
NA	NA	NA	NA	NA	NA	NA	NA	NA
NA	NA	NA	########	NA	NA	NA	########	NA
NA	NA	NA	NA	NA	NA	NA	NA	NA
NA	########	########	########	NA	########	########	########	NA
NA	NA	NA	NA	NA	NA	NA	NA	NA
NA	NA	NA	########	NA	NA	NA	########	NA
NA	NA	NA	########	NA	NA	NA	########	NA
NA	NA	NA	NA	NA	NA	NA	NA	NA
NA	NA	NA	NA	NA	NA	NA	NA	NA
NA	NA	NA	########	NA	NA	NA	########	NA
NA	NA	NA	########	NA	NA	NA	NA	NA
########	########	########	########	########	########	########	########	NA
NA	NA	NA	########	NA	NA	NA	########	NA
NA	NA	NA	########	NA	NA	NA	########	NA
NA	NA	NA	NA	NA	NA	NA	NA	NA
NA	NA	NA	########	NA	NA	NA	########	NA
NA	########	########	########	NA	########	########	########	########
NA	NA	NA	########	NA	NA	NA	########	NA
########	NA	NA	########	NA	NA	NA	NA	NA
NA	NA	NA	NA	NA	NA	NA	NA	NA
NA	NA	NA	NA	NA	NA	NA	NA	NA
NA	NA	NA	########	NA	NA	NA	########	NA
########	########	########	########	########	########	########	########	########
NA	NA	NA	NA	NA	NA	NA	NA	NA
########	NA	########	########	NA	NA	########	########	########
########	NA	NA	########	NA	NA	########	########	########
NA	NA	NA	########	NA	NA	NA	########	NA
NA	NA	########	########	NA	NA	########	########	NA
NA	########	########	########	NA	########	########	########	NA
########	########	########	NA	########	########	########	NA	NA
NA	NA	NA	NA	NA	NA	NA	NA	NA
NA	NA	NA	########	NA	NA	NA	########	NA
NA	########	########	NA	NA	########	########	NA	NA
NA	NA	########	NA	NA	NA	########	NA	NA
NA	NA	NA	########	NA	NA	NA	NA	NA
NA	NA	########	########	NA	NA	########	########	NA
NA	NA	NA	NA	NA	NA	NA	NA	NA
NA	NA	NA	NA	NA	NA	NA	NA	NA
NA	########	########	########	NA	########	########	########	########
NA	NA	NA	########	NA	NA	NA	########	NA
NA	NA	NA	########	NA	NA	NA	########	NA
########	########	########	NA	########	########	########	########	########

For Review Only

NA	NA	NA	########	NA	NA	NA	########	NA
NA	NA	NA	########	NA	NA	NA	########	NA
NA	NA	NA	NA	NA	NA	NA	NA	NA
NA	NA	NA	########	NA	NA	NA	########	NA
NA	NA	NA	NA	NA	NA	NA	NA	NA
NA	NA	NA	########	NA	NA	NA	########	NA
NA	NA	NA	NA	NA	NA	NA	NA	NA
NA	########	########	########	########	########	########	########	NA
NA	NA	NA	########	NA	NA	NA	########	NA
NA	NA	NA	########	NA	NA	NA	########	NA
NA	NA	########	########	NA	NA	########	########	NA
NA	NA	########	########	NA	NA	########	########	NA
NA	NA	########	########	NA	NA	########	########	########
NA	NA	########	########	NA	NA	########	########	NA
NA	########	########	########	########	########	########	########	########
NA	########	########	NA	NA	########	NA	NA	NA
NA	NA	NA	NA	NA	NA	########	########	NA
NA	NA	NA	NA	NA	NA	NA	NA	NA
NA	NA	NA	NA	NA	NA	NA	NA	NA
NA	NA	NA	########	NA	NA	NA	########	NA
NA	NA	NA	NA	NA	NA	NA	NA	NA
NA	NA	NA	########	NA	NA	NA	########	NA
NA	NA	########	########	NA	NA	########	########	NA
NA	NA	########	NA	NA	NA	NA	NA	NA
NA	NA	NA	NA	NA	NA	NA	NA	NA
NA	NA	NA	########	NA	NA	NA	########	NA
NA	NA	NA	NA	NA	NA	NA	NA	NA
########	########	########	########	########	########	########	########	########
NA	NA	NA	########	NA	NA	NA	########	NA
NA	NA	########	NA	NA	NA	########	NA	NA
NA	NA	NA	########	NA	NA	NA	########	NA
########	########	########	########	########	########	########	########	########
########	########	########	########	########	########	########	########	NA
########	########	########	########	########	########	########	########	NA
NA	########	########	########	########	########	########	########	NA
########	########	########	########	########	########	########	########	########
NA	NA	NA	NA	NA	NA	########	NA	NA
########	########	########	########	########	########	########	########	########
NA	########	########	NA	NA	########	########	NA	NA
NA	NA	NA	NA	NA	NA	NA	NA	NA
NA	NA	NA	########	NA	NA	########	########	NA
NA	NA	NA	########	NA	NA	NA	########	NA
NA	########	########	NA	NA	########	########	NA	########
NA	NA	NA	NA	NA	NA	NA	NA	NA
NA	NA	NA	########	NA	NA	NA	########	NA
NA	NA	NA	########	NA	NA	NA	########	NA
NA	NA	########	NA	NA	NA	########	NA	NA
NA	########	########	########	NA	########	########	########	########
NA	NA	NA	########	NA	NA	NA	########	NA
NA	NA	########	NA	NA	NA	########	NA	NA
NA	NA	NA	########	NA	NA	NA	########	NA
NA	NA	########	########	NA	NA	########	########	NA
NA	NA	NA	NA	NA	NA	NA	NA	NA
########	########	NA	NA	NA	NA	NA	########	NA
NA	NA	########	NA	NA	NA	########	NA	NA
NA	########	########	########	NA	########	########	########	########

For Review Only

NA	NA	########	########	NA	NA	########	########	NA
NA	NA	########	########	NA	NA	########	########	NA
NA	########	########	########	NA	NA	########	########	NA
NA	NA	########	########	NA	NA	########	########	NA
NA	NA	NA	########	NA	NA	NA	NA	NA
NA	NA	NA	########	NA	NA	NA	########	NA
NA	NA	NA	NA	NA	NA	NA	NA	NA
NA	NA	NA	NA	NA	NA	NA	NA	NA
NA	NA	NA	########	NA	NA	NA	########	NA
NA	NA	NA	NA	NA	NA	NA	NA	NA
NA	NA	NA	NA	NA	NA	NA	NA	NA
NA	NA	NA	########	NA	NA	NA	########	NA
NA	NA	NA	########	NA	NA	NA	########	NA
NA	NA	NA	########	NA	NA	NA	########	NA
NA	NA	NA	########	NA	NA	NA	########	NA
NA	NA	NA	########	NA	NA	NA	########	NA
NA	NA	NA	########	NA	NA	NA	########	NA
NA	NA	NA	########	NA	NA	########	########	NA
########	########	NA	NA	NA	NA	NA	NA	NA
NA	########	NA	########	NA	NA	NA	########	NA
NA	NA	NA	NA	NA	NA	NA	########	NA
NA	NA	NA	NA	NA	NA	NA	NA	NA
NA	NA	########	NA	NA	NA	########	NA	NA
NA	NA	NA	########	NA	NA	NA	########	NA
NA	NA	NA	NA	NA	NA	NA	NA	NA
NA	########	########	NA	NA	NA	########	NA	NA
NA	NA	########	NA	NA	NA	########	NA	NA
NA	NA	NA	########	NA	NA	NA	########	NA
NA	NA	########	NA	NA	NA	########	NA	NA
NA	NA	########	########	NA	NA	########	########	NA
NA	########	########	NA	NA	NA	########	NA	NA
NA	NA	########	NA	NA	NA	########	NA	NA
########	########	########	NA	########	########	########	NA	NA
NA	NA	NA	NA	NA	NA	NA	NA	NA
NA	NA	########	########	NA	NA	NA	########	NA
NA	NA	########	########	NA	NA	NA	########	NA
NA	########	########	########	NA	########	########	NA	NA
NA	NA	NA	########	NA	NA	NA	########	NA
NA	NA	NA	NA	NA	NA	NA	NA	NA
NA	NA	NA	########	NA	NA	NA	########	NA
NA	########	########	########	NA	########	########	########	########
NA	NA	NA	########	NA	NA	NA	########	NA
NA	NA	NA	########	NA	NA	NA	########	NA
NA	NA	########	########	NA	NA	########	########	NA
NA	NA	NA	########	NA	NA	NA	NA	NA
NA	NA	NA	NA	NA	NA	NA	NA	NA
NA	########	########	NA	NA	NA	########	NA	NA
NA	NA	########	########	NA	NA	########	########	NA
NA	########	########	########	NA	NA	NA	########	NA
NA	NA	NA	########	NA	NA	NA	########	NA
NA	########	########	########	NA	########	########	########	NA
NA	NA	########	NA	NA	NA	########	NA	NA

For Review Only

NA	NA	########	NA	NA	NA	########	NA	NA
NA	########	########	NA	NA	NA	########	NA	NA
NA	NA	########	NA	NA	NA	########	NA	NA
NA	NA	########	NA	NA	NA	########	NA	NA
NA	########	########	NA	NA	########	########	NA	NA
NA	########	########	NA	NA	NA	########	NA	NA
NA	NA	########	NA	NA	NA	########	NA	NA
NA	########	########	NA	NA	NA	########	NA	NA
NA	NA	########	NA	NA	NA	########	NA	NA
NA	NA	########	########	NA	NA	########	########	NA
NA	NA	########	NA	NA	NA	NA	NA	NA
NA	NA	########	NA	NA	NA	########	NA	NA
NA	NA	NA	########	NA	NA	NA	########	NA
NA	########	########	NA	NA	########	########	NA	NA
NA	NA	########	NA	NA	NA	########	NA	NA
NA	NA	########	NA	NA	NA	########	NA	NA
NA	NA	NA	########	NA	NA	NA	########	NA
NA	########	########	########	NA	########	########	########	########
NA	NA	NA	########	NA	NA	NA	########	NA
NA	NA	########	NA	NA	NA	########	NA	NA
NA	NA	########	NA	NA	NA	########	NA	NA
NA	########	########	NA	NA	NA	########	NA	NA
NA	NA	########	########	NA	NA	########	########	NA
NA	NA	########	NA	NA	NA	########	NA	NA
NA	NA	########	NA	NA	NA	########	NA	NA
NA	NA	NA	########	NA	NA	NA	########	NA
NA	NA	NA	NA	NA	NA	NA	NA	NA
NA	NA	NA	NA	NA	NA	NA	NA	NA
NA	NA	NA	########	NA	NA	NA	########	NA
NA	NA	########	NA	NA	NA	########	NA	NA
NA	NA	########	########	NA	NA	########	########	NA
NA	NA	NA	########	NA	NA	NA	########	NA
NA	########	########	NA	NA	########	########	NA	NA
NA	NA	########	NA	NA	NA	########	NA	NA
NA	NA	########	NA	NA	NA	########	NA	NA
NA	NA	########	NA	NA	NA	########	NA	NA
NA	NA	########	NA	NA	NA	########	NA	NA
NA	NA	########	NA	NA	NA	########	NA	NA
NA	########	########	########	NA	########	########	########	########
NA	NA	NA	########	NA	NA	NA	########	NA
NA	########	########	NA	NA	NA	########	NA	NA
NA	########	########	NA	NA	NA	########	NA	NA
NA	NA	########	NA	NA	NA	########	NA	NA
NA	NA	########	NA	NA	NA	########	NA	NA
NA	NA	########	NA	NA	NA	########	NA	NA
NA	########	########	NA	NA	########	########	NA	NA
NA	NA	NA	########	NA	NA	NA	########	NA
NA	NA	NA	########	NA	NA	NA	########	NA
NA	NA	NA	NA	NA	NA	NA	NA	NA
NA	NA	NA	########	NA	NA	NA	########	NA
NA	NA	NA	NA	NA	NA	NA	NA	NA
NA	########	########	NA	NA	########	########	NA	########
NA	NA	########	NA	NA	NA	########	NA	NA
NA	NA	########	########	NA	NA	########	########	NA
NA	NA	NA	########	NA	NA	NA	########	NA
NA	########	########	########	NA	########	########	########	########

For Review Only

NA	NA	NA	########	NA	NA	NA	########	NA
NA	NA	NA	########	NA	NA	NA	########	NA
NA	NA	NA	NA	NA	NA	NA	NA	NA
NA	NA	########	NA	NA	NA	########	NA	NA
NA	NA	NA	NA	NA	NA	NA	########	NA
NA	NA	NA	NA	NA	NA	NA	########	NA
NA	########	########	NA	NA	NA	########	NA	NA
NA	NA	NA	########	NA	NA	NA	########	NA
NA	NA	NA	########	NA	NA	NA	########	NA
NA	########	########	NA	NA	########	########	NA	########
NA	########	########	NA	NA	########	########	NA	NA
NA	NA	NA	NA	NA	NA	NA	NA	NA
NA	########	########	NA	NA	########	########	NA	NA
########	NA	NA	########	NA	NA	NA	########	NA
NA	########	########	########	NA	########	########	########	########
NA	NA	########	NA	NA	NA	########	NA	NA
NA	NA	NA	########	NA	NA	NA	NA	NA
NA	NA	NA	########	NA	NA	NA	########	NA
NA	NA	NA	NA	NA	NA	NA	NA	NA
NA	NA	NA	########	NA	NA	NA	########	NA
########	########	########	########	NA	NA	NA	########	NA
NA	NA	########	########	NA	NA	########	########	NA
NA	NA	########	########	NA	NA	NA	########	NA
NA	NA	########	########	NA	NA	NA	########	NA
NA	NA	NA	NA	NA	NA	NA	NA	NA
NA	NA	NA	########	NA	NA	NA	########	NA
NA	NA	NA	NA	NA	NA	NA	NA	NA
NA	NA	NA	########	NA	NA	NA	########	NA
########	NA	NA	NA	########	NA	########	########	########
NA	NA	NA	NA	NA	NA	NA	NA	NA
NA	NA	NA	########	NA	NA	NA	########	NA
NA	NA	NA	########	NA	NA	NA	########	NA
NA	########	########	########	NA	########	########	########	########
NA	NA	NA	NA	NA	NA	NA	NA	NA
########	########	########	########	########	########	########	########	NA
NA	NA	NA	########	NA	NA	NA	########	NA
NA	NA	NA	########	NA	NA	NA	########	NA
NA	NA	########	NA	NA	NA	########	NA	NA
NA	NA	NA	NA	NA	NA	NA	NA	NA
NA	NA	########	########	NA	NA	########	########	NA
NA	########	NA	########	NA	########	NA	########	NA
NA	NA	NA	NA	NA	NA	NA	NA	NA
NA	NA	NA	NA	NA	NA	NA	NA	NA
NA	NA	########	########	NA	NA	########	########	NA
########	########	########	########	########	########	########	########	NA
NA	########	########	NA	NA	########	########	NA	NA
########	########	########	########	########	########	########	########	NA
NA	########	########	NA	NA	NA	########	NA	NA
NA	NA	NA	########	NA	NA	NA	########	NA
NA	NA	NA	########	NA	NA	NA	########	NA
NA	NA	NA	########	NA	NA	NA	########	NA
NA	NA	NA	NA	NA	NA	NA	NA	NA
NA	NA	NA	########	NA	NA	NA	########	NA
NA	NA	NA	########	NA	NA	NA	########	NA
NA	NA	########	NA	NA	NA	########	NA	NA
NA	########	########	NA	NA	########	########	NA	NA

For Review Only

NA	NA	NA	NA	NA	NA	NA	NA	NA
NA	NA	NA	NA	NA	NA	NA	NA	NA
NA	NA	NA	NA	NA	NA	NA	NA	NA
NA	NA	NA	NA	NA	NA	NA	NA	NA
NA	########	NA	########	NA	########	NA	########	NA
NA	NA	########	########	NA	NA	########	########	NA
########	########	########	########	########	########	########	########	NA
NA	NA	NA	########	NA	NA	NA	########	NA
########	########	########	########	########	########	########	########	########
########	NA	NA	########	########	NA	NA	########	NA
NA	NA	########	########	NA	NA	NA	########	NA
NA	NA	NA	NA	NA	NA	NA	NA	NA
NA	NA	NA	NA	NA	NA	NA	NA	NA
NA	NA	########	NA	NA	NA	########	NA	########
NA	NA	########	NA	NA	NA	########	NA	NA
NA	NA	NA	NA	NA	NA	NA	NA	NA
NA	NA	NA	########	NA	NA	NA	########	NA
NA	NA	NA	NA	NA	NA	NA	NA	NA
########	########	########	########	NA	NA	########	########	NA
########	########	########	########	NA	########	########	########	########
NA	NA	NA	########	NA	NA	NA	########	NA
NA	########	########	NA	NA	########	########	NA	NA
NA	NA	NA	NA	NA	NA	NA	NA	NA
NA	NA	NA	########	NA	NA	NA	########	NA
NA	NA	NA	########	NA	NA	NA	########	NA
NA	########	########	########	NA	########	########	########	########
NA	NA	NA	########	NA	NA	NA	########	NA
NA	########	########	NA	NA	########	########	NA	NA
NA	NA	NA	########	NA	NA	NA	########	NA
NA	########	NA	########	NA	########	NA	########	NA
NA	NA	NA	NA	NA	NA	NA	NA	NA
NA	NA	NA	########	NA	NA	NA	########	NA
NA	NA	NA	NA	NA	NA	NA	NA	NA
NA	########	########	NA	NA	NA	########	NA	NA
NA	NA	########	########	NA	NA	########	########	NA
########	########	########	########	########	########	########	########	########
NA	NA	NA	NA	NA	NA	NA	NA	NA
NA	########	########	NA	########	########	########	NA	########
########	########	########	########	########	########	########	########	########
NA	########	########	NA	NA	NA	########	NA	NA
NA	########	########	########	NA	NA	########	########	NA
NA	NA	NA	NA	NA	NA	NA	NA	NA
########	########	NA	NA	########	NA	NA	NA	NA
NA	########	########	NA	NA	NA	########	NA	NA
NA	NA	########	NA	NA	NA	########	NA	NA
NA	NA	NA	########	NA	NA	NA	########	NA
########	########	########	########	########	########	########	########	########
NA	NA	NA	########	NA	NA	NA	########	NA
NA	NA	NA	NA	NA	NA	NA	NA	NA
NA	NA	########	NA	NA	NA	########	NA	NA
NA	NA	NA	########	NA	NA	NA	########	NA
NA	NA	NA	NA	NA	NA	NA	NA	NA
NA	NA	NA	NA	NA	NA	NA	NA	NA
NA	NA	NA	########	NA	NA	NA	########	NA
NA	NA	NA	########	NA	NA	NA	########	NA
NA	NA	NA	NA	NA	NA	NA	NA	NA

For Review Only

########	NA	NA	NA	NA	NA	NA	NA	NA
NA	########	NA	########	NA	########	NA	########	NA
NA	########	NA	NA	NA	########	NA	NA	NA
########	########	########	########	########	########	########	########	########
NA	NA	########	########	NA	NA	########	########	NA
NA	########	########	########	NA	NA	########	########	NA
NA	########	########	########	NA	########	########	########	NA
NA	NA	NA	########	NA	NA	NA	########	NA
########	########	########	########	########	########	########	########	NA
NA	NA	NA	########	NA	NA	NA	NA	NA
NA	NA	NA	########	NA	NA	NA	########	NA
NA	########	########	NA	NA	NA	########	NA	NA
NA	NA	########	########	NA	NA	########	########	NA
NA	NA	NA	NA	NA	NA	NA	NA	NA
NA	########	NA	########	NA	NA	NA	########	NA
NA	NA	########	########	NA	NA	########	########	NA
NA	NA	########	NA	NA	NA	########	NA	NA
########	########	########	########	########	########	########	########	########
########	########	NA	NA	########	NA	NA	########	########
NA	NA	NA	NA	NA	NA	NA	########	NA
NA	NA	NA	NA	NA	NA	NA	NA	NA
NA	NA	NA	########	NA	NA	NA	########	NA
NA	########	########	NA	NA	########	########	NA	NA
NA	########	########	NA	NA	########	########	NA	########
NA	########	########	########	NA	########	########	########	########
NA	NA	NA	########	NA	NA	NA	########	NA
NA	NA	NA	########	NA	NA	NA	########	NA
NA	NA	NA	NA	NA	NA	NA	NA	NA
NA	NA	NA	NA	NA	########	NA	NA	NA
NA	NA	########	########	NA	NA	NA	########	NA
NA	NA	########	NA	NA	NA	########	NA	NA
NA	########	########	########	NA	########	########	########	########
########	########	########	########	NA	NA	NA	NA	NA
NA	NA	NA	NA	NA	NA	NA	NA	NA
NA	NA	NA	########	NA	NA	NA	########	NA
NA	NA	NA	########	NA	NA	NA	########	NA
NA	NA	NA	########	NA	NA	NA	########	NA
NA	NA	NA	NA	NA	NA	NA	NA	NA
NA	NA	NA	NA	NA	NA	NA	NA	NA
NA	NA	NA	NA	NA	NA	NA	NA	NA
NA	NA	NA	########	NA	########	NA	########	########
NA	NA	NA	NA	NA	NA	NA	NA	NA
NA	########	########	########	NA	NA	########	########	NA
NA	NA	NA	########	NA	NA	NA	########	NA
NA	NA	NA	NA	NA	NA	NA	NA	NA
########	########	########	########	########	########	########	########	NA
NA	NA	########	########	NA	NA	########	########	NA
NA	NA	NA	########	NA	NA	NA	########	NA
NA	NA	NA	########	NA	NA	NA	########	########
NA	########	########	########	NA	NA	NA	########	NA
NA	NA	NA	NA	NA	NA	NA	NA	NA
########	########	########	NA	########	########	NA	########	NA
NA	NA	NA	NA	NA	NA	NA	NA	NA
NA	########	########	########	########	########	########	########	########
NA	NA	########	########	NA	########	########	########	NA
########	########	########	########	NA	########	NA	########	NA

For Review Only

NA	########	########	NA	NA	NA	########	NA	NA
NA	NA	NA	########	NA	NA	NA	########	NA
NA	NA	NA	########	NA	NA	NA	########	NA
NA	########	########	NA	NA	########	NA	NA	########
########	########	########	########	########	########	########	########	########
########	########	########	########	NA	########	########	########	########
NA	NA	NA	NA	NA	NA	NA	########	NA
########	NA	NA	NA	########	NA	NA	NA	########
NA	NA	NA	########	NA	NA	NA	########	NA
NA	NA	########	########	NA	NA	########	########	NA
NA	########	########	NA	########	########	########	NA	NA
NA	NA	NA	NA	NA	NA	NA	NA	NA
NA	NA	NA	NA	NA	NA	NA	########	NA
NA	NA	########	########	NA	NA	NA	NA	NA
NA	NA	########	########	NA	########	########	########	NA
NA	NA	NA	NA	NA	NA	NA	########	NA
NA	NA	NA	NA	NA	NA	NA	NA	NA
NA	NA	NA	NA	NA	NA	NA	NA	NA
NA	NA	NA	########	NA	NA	NA	########	NA
NA	NA	########	########	NA	NA	########	########	NA
NA	NA	NA	########	NA	NA	NA	########	NA
NA	NA	NA	NA	NA	NA	NA	NA	NA
NA	NA	NA	########	NA	NA	NA	########	NA
########	########	########	NA	########	########	########	NA	NA
########	NA	NA	NA	NA	NA	NA	NA	########
NA	NA	NA	########	NA	NA	NA	########	NA
NA	NA	NA	NA	NA	NA	NA	########	NA
NA	NA	NA	########	NA	NA	NA	########	NA
########	NA	NA	NA	########	NA	NA	NA	NA
########	########	########	########	NA	NA	NA	NA	NA
NA	NA	NA	########	NA	NA	NA	########	NA
NA	NA	NA	NA	NA	NA	NA	NA	NA
NA	NA	########	NA	NA	NA	########	NA	NA
NA	NA	NA	NA	NA	NA	NA	NA	NA
NA	NA	NA	########	NA	NA	NA	########	########
########	########	########	########	NA	########	########	########	NA
########	########	########	NA	########	########	########	NA	########
NA	NA	########	NA	NA	NA	########	NA	NA
NA	########	########	########	NA	########	########	########	########
########	########	########	########	NA	NA	########	########	NA
NA	NA	NA	########	NA	NA	NA	########	NA
NA	NA	NA	########	NA	NA	NA	########	NA
NA	########	########	NA	NA	########	########	NA	NA
########	########	########	NA	NA	########	NA	NA	NA
########	########	########	########	########	########	########	########	########
########	########	NA	########	########	########	NA	########	NA
NA	NA	########	########	NA	NA	########	########	NA
NA	NA	########	########	NA	NA	########	########	NA
NA	########	########	########	NA	########	########	########	NA
NA	NA	########	########	NA	NA	########	########	NA
NA	NA	NA	NA	NA	NA	NA	NA	NA
NA	########	########	########	########	########	########	########	NA
NA	NA	NA	NA	NA	NA	NA	NA	NA
NA	NA	NA	NA	NA	NA	NA	NA	NA
NA	NA	########	########	NA	NA	########	########	NA
NA	########	########	########	NA	########	########	########	NA

For Review Only

NA	NA	########	########	NA	NA	########	########	NA
NA	NA	########	########	NA	NA	########	########	NA
NA	NA	NA	########	NA	NA	NA	########	NA
NA	NA	NA	########	NA	NA	NA	########	NA
NA	NA	NA	NA	NA	NA	NA	NA	NA
NA	NA	NA	NA	NA	NA	NA	NA	NA
NA	NA	NA	########	NA	NA	########	########	NA
NA	NA	########	########	NA	NA	########	########	NA
NA	NA	NA	########	NA	NA	NA	########	NA
NA	NA	NA	########	NA	NA	NA	########	NA
NA	NA	########	########	NA	NA	########	########	NA
NA	NA	NA	########	NA	NA	NA	########	NA
NA	NA	NA	########	NA	NA	NA	########	NA
NA	NA	NA	########	NA	NA	NA	########	NA
NA	########	########	NA	NA	NA	########	NA	NA
########	########	NA	NA	NA	NA	NA	########	NA
NA	########	########	NA	NA	########	########	NA	NA
########	########	########	########	NA	########	########	########	########
########	########	########	########	########	########	########	########	########
NA	NA	NA	NA	NA	NA	NA	NA	NA
NA	NA	NA	########	NA	NA	NA	########	NA
NA	NA	NA	NA	NA	NA	NA	NA	NA
NA	NA	NA	NA	NA	NA	NA	NA	NA
NA	NA	########	########	NA	NA	NA	########	NA
NA	NA	NA	########	NA	NA	NA	########	NA
NA	NA	NA	NA	NA	NA	NA	NA	NA
NA	########	########	########	NA	NA	########	########	########
NA	NA	NA	NA	NA	NA	NA	NA	NA
NA	NA	NA	########	NA	NA	NA	########	NA
NA	NA	NA	########	NA	NA	NA	########	NA
########	########	########	########	########	########	########	########	NA
NA	NA	NA	NA	NA	NA	NA	NA	NA
NA	NA	NA	########	NA	NA	NA	########	NA
NA	NA	NA	NA	NA	NA	NA	NA	NA
NA	NA	########	########	NA	NA	NA	########	NA
NA	NA	NA	NA	NA	NA	NA	########	NA
NA	NA	########	NA	NA	NA	########	NA	NA
NA	########	########	########	NA	########	########	########	########
NA	########	########	########	NA	########	########	########	########
NA	########	########	NA	NA	NA	NA	NA	NA
NA	########	########	########	NA	NA	########	########	########
NA	NA	NA	NA	NA	NA	NA	NA	NA
########	########	NA	NA	NA	########	NA	NA	NA
NA	NA	NA	NA	NA	NA	NA	NA	NA
########	########	########	########	########	########	########	########	NA
NA	NA	NA	NA	NA	NA	NA	NA	########
NA	NA	NA	########	NA	NA	NA	########	NA
NA	NA	NA	########	NA	NA	NA	########	NA
NA	NA	NA	########	NA	NA	NA	########	NA
NA	NA	NA	########	NA	NA	########	########	NA
NA	NA	NA	########	NA	NA	NA	########	NA
NA	NA	NA	NA	NA	NA	NA	NA	NA
NA	########	########	NA	NA	########	########	NA	NA
NA	NA	NA	########	NA	NA	NA	########	NA
########	########	NA	NA	NA	NA	########	########	NA
NA	NA	NA	########	NA	NA	NA	########	NA

For Review Only

NA	NA	NA	NA	NA	NA	NA	NA	NA
########	NA	########	########	NA	NA	########	########	NA
########	########	NA	########	NA	########	NA	########	NA
NA	NA	NA	########	NA	NA	NA	########	NA
NA	NA	NA	NA	NA	NA	NA	########	NA
NA	NA	NA	NA	NA	NA	NA	NA	NA
########	########	########	NA	NA	########	########	########	NA
NA	NA	NA	########	NA	NA	NA	########	NA
NA	NA	########	NA	NA	NA	########	NA	NA
########	########	########	NA	NA	########	########	NA	NA
NA	NA	NA	########	NA	NA	NA	########	NA
NA	NA	NA	########	NA	NA	NA	########	NA
NA	NA	########	########	NA	NA	########	########	########
NA	NA	########	NA	NA	NA	########	NA	NA
NA	NA	NA	NA	NA	NA	NA	NA	NA
########	########	NA	NA	########	########	NA	NA	NA
########	########	NA	########	########	########	NA	########	NA
NA	NA	NA	NA	NA	NA	NA	########	NA
NA	NA	NA	NA	NA	NA	NA	NA	NA
NA	########	########	########	NA	########	########	########	########
NA	NA	########	NA	NA	NA	########	NA	NA
NA	NA	NA	NA	NA	########	NA	NA	NA
NA	NA	NA	########	NA	NA	NA	########	NA
NA	NA	NA	########	NA	NA	NA	########	NA
NA	NA	NA	########	NA	NA	NA	########	NA
NA	NA	########	NA	NA	NA	########	NA	NA
NA	########	NA	########	NA	########	NA	########	########
NA	NA	########	########	NA	NA	NA	########	NA
NA	NA	########	NA	NA	NA	########	NA	NA
NA	NA	NA	NA	NA	NA	NA	NA	NA
NA	NA	NA	########	NA	NA	NA	########	NA
NA	NA	NA	NA	NA	NA	NA	NA	NA
NA	NA	NA	########	NA	NA	NA	########	NA
NA	NA	NA	NA	NA	NA	NA	NA	NA
NA	########	NA	NA	NA	NA	NA	NA	NA
NA	NA	NA	NA	NA	NA	NA	NA	NA
NA	########	########	########	########	########	########	########	########
NA	NA	########	########	NA	NA	########	NA	NA
NA	NA	########	########	NA	NA	########	########	NA
NA	NA	NA	########	NA	NA	NA	########	NA
NA	NA	NA	########	NA	NA	NA	########	NA
NA	NA	########	########	NA	########	########	########	NA
########	NA	########	########	########	NA	########	########	########
NA	NA	NA	########	NA	NA	NA	########	NA
########	########	NA	NA	NA	NA	########	########	NA
NA	NA	NA	NA	NA	NA	NA	NA	NA
NA	NA	NA	NA	NA	NA	NA	NA	NA
########	########	########	########	########	########	########	########	########
########	########	########	########	########	########	########	########	########
NA	NA	NA	NA	NA	NA	NA	NA	NA
NA	NA	NA	########	NA	NA	NA	########	NA
NA	NA	NA	NA	NA	NA	NA	NA	NA
########	########	########	NA	########	########	########	########	NA
NA	########	NA	########	NA	NA	NA	########	NA
NA	NA	########	########	NA	########	########	########	NA
NA	NA	NA	NA	NA	NA	NA	NA	NA

For Review Only

NA	NA	NA	NA	NA	NA	NA	NA	NA
NA	NA	NA	NA	NA	NA	NA	NA	NA
NA	NA	NA	########	NA	NA	NA	########	NA
NA	NA	NA	########	NA	NA	NA	########	NA
NA	NA	NA	########	NA	NA	NA	########	NA
########	########	NA	NA	########	########	NA	NA	NA
NA	NA	NA	########	NA	NA	NA	########	NA
NA	NA	NA	########	NA	NA	NA	########	NA
NA	NA	NA	NA	NA	NA	NA	NA	NA
NA	NA	NA	NA	NA	NA	NA	NA	NA
NA	NA	NA	########	NA	########	NA	NA	NA
NA	NA	NA	########	NA	NA	NA	########	NA
NA	NA	########	########	NA	NA	NA	########	NA
NA	########	########	########	NA	NA	########	########	NA
NA	########	########	########	NA	########	########	########	########
NA	NA	NA	########	NA	NA	NA	########	NA
NA	NA	NA	NA	NA	NA	NA	NA	NA
NA	NA	NA	########	NA	NA	NA	########	NA
NA	NA	########	########	NA	NA	NA	########	NA
NA	NA	NA	########	NA	NA	NA	########	NA
NA	NA	########	########	NA	NA	########	########	NA
NA	NA	NA	NA	NA	NA	NA	NA	NA
NA	NA	########	NA	NA	NA	########	NA	NA
NA	NA	NA	########	NA	NA	NA	########	NA
NA	NA	NA	########	NA	NA	NA	########	NA
########	########	########	########	########	########	########	NA	NA
NA	NA	NA	########	NA	NA	NA	########	NA
NA	NA	NA	NA	NA	NA	NA	NA	NA
NA	########	########	NA	NA	########	########	NA	########
NA	NA	NA	NA	NA	NA	NA	NA	NA
NA	NA	NA	NA	NA	NA	NA	NA	NA
NA	NA	NA	########	NA	NA	NA	NA	NA
NA	NA	########	########	NA	NA	NA	########	NA
NA	NA	NA	NA	NA	NA	NA	NA	NA
NA	NA	NA	NA	NA	NA	NA	NA	NA
########	########	########	########	NA	NA	NA	########	NA
NA	NA	NA	########	NA	NA	NA	########	NA
NA	NA	NA	########	NA	NA	NA	########	NA
NA	NA	NA	########	NA	NA	NA	########	NA
NA	NA	NA	NA	NA	NA	NA	NA	NA
NA	NA	NA	########	NA	NA	NA	########	NA
NA	NA	NA	########	NA	NA	NA	########	NA
NA	########	########	########	########	########	########	########	########
NA	NA	NA	########	NA	NA	NA	########	NA
NA	NA	NA	########	NA	NA	NA	########	NA
NA	NA	NA	########	NA	NA	NA	NA	NA
NA	NA	NA	########	NA	NA	NA	########	NA
NA	NA	NA	NA	NA	NA	NA	NA	NA
NA	NA	NA	NA	NA	NA	NA	########	NA
NA	NA	NA	########	NA	NA	NA	########	NA
NA	########	########	NA	NA	########	########	NA	NA
NA	NA	NA	########	NA	NA	NA	########	NA
NA	NA	NA	########	NA	NA	NA	########	NA
NA	NA	NA	NA	NA	NA	NA	NA	NA
NA	NA	NA	NA	NA	NA	NA	NA	NA
NA	NA	NA	########	NA	NA	NA	########	NA

For Review Only

NA	NA	########	NA	NA	NA	########	NA	NA
NA	########	########	NA	NA	########	########	NA	NA
########	########	########	########	########	########	NA	NA	NA
NA	NA	NA	########	NA	NA	NA	########	NA
NA	NA	NA	########	NA	NA	NA	########	NA
NA	NA	########	########	NA	NA	########	########	NA
########	########	########	########	########	########	########	########	########
NA	NA	NA	########	NA	NA	NA	########	NA
NA	NA	NA	########	NA	NA	NA	########	NA
NA	NA	NA	########	NA	NA	NA	########	NA
NA	########	########	########	NA	########	########	########	NA
NA	NA	NA	########	NA	NA	NA	########	NA
NA	NA	NA	NA	NA	NA	NA	NA	NA
NA	NA	NA	NA	NA	NA	NA	NA	NA
########	########	########	########	########	########	########	########	########
########	########	########	########	########	########	########	########	########
NA	NA	NA	NA	NA	NA	NA	NA	NA
########	########	########	########	########	########	########	########	NA
NA	NA	NA	########	NA	NA	NA	########	NA
NA	########	########	########	NA	########	########	########	NA
NA	NA	########	NA	NA	NA	########	NA	NA
NA	NA	NA	########	NA	NA	NA	########	NA
NA	NA	########	########	NA	NA	########	########	NA
NA	NA	NA	########	NA	NA	NA	########	NA
NA	NA	NA	NA	NA	NA	NA	NA	NA
NA	NA	########	NA	NA	NA	########	NA	NA
NA	NA	NA	NA	NA	NA	NA	NA	NA
NA	NA	NA	########	NA	NA	NA	########	NA
########	########	########	########	########	########	########	########	########
NA	NA	########	########	NA	NA	########	########	NA
NA	NA	NA	########	NA	NA	NA	########	NA
########	########	########	########	########	########	########	########	########
NA	NA	NA	########	NA	NA	NA	########	NA
NA	NA	NA	NA	NA	NA	NA	NA	NA
NA	NA	########	NA	NA	NA	NA	NA	NA
NA	########	########	########	NA	########	########	########	NA
NA	NA	NA	NA	NA	NA	NA	NA	NA
NA	########	########	########	########	########	########	########	NA
NA	NA	NA	########	NA	NA	NA	########	NA
NA	NA	########	########	NA	NA	########	########	NA
NA	########	########	NA	NA	########	########	NA	NA
########	########	########	########	NA	########	########	########	NA
NA	NA	NA	NA	NA	NA	NA	NA	NA
NA	NA	NA	NA	NA	NA	NA	NA	NA
NA	NA	########	NA	NA	NA	NA	NA	NA
########	########	########	########	########	########	########	########	NA
NA	########	########	########	########	########	########	########	########
NA	NA	NA	NA	NA	NA	NA	NA	NA

For Review Only

NA	NA	NA	NA	NA	NA	NA	NA	NA
NA	NA	NA	NA	NA	NA	NA	NA	NA
NA	NA	########	NA	NA	NA	########	NA	NA
NA	NA	NA	NA	NA	NA	NA	NA	NA
NA	NA	NA	########	NA	NA	NA	########	NA
NA	NA	NA	NA	NA	NA	NA	NA	NA
NA	NA	NA	NA	########	########	########	########	NA
NA	NA	NA	NA	########	########	########	########	NA
NA	########	########	########	########	########	########	########	NA
NA	NA	NA	########	NA	NA	NA	########	NA
NA	NA	########	########	NA	NA	NA	NA	NA
NA	NA	NA	########	NA	NA	NA	########	NA
NA	NA	NA	NA	########	########	########	########	NA
########	########	########	########	########	########	########	########	NA
NA	NA	NA	NA	NA	########	########	########	NA
NA	########	########	########	NA	NA	########	########	########
NA	NA	NA	NA	NA	NA	NA	NA	NA
NA	NA	########	NA	NA	NA	########	NA	NA
NA	NA	NA	########	NA	NA	NA	########	NA
NA	NA	NA	########	NA	NA	NA	########	NA
NA	########	########	########	########	########	########	########	NA
NA	NA	########	########	NA	NA	########	########	########
NA	NA	NA	########	NA	NA	NA	########	NA
NA	NA	########	########	NA	NA	########	########	NA
NA	NA	NA	########	NA	NA	NA	########	NA
########	########	########	########	########	########	########	########	NA
NA	NA	NA	NA	NA	NA	NA	NA	NA
NA	NA	########	########	NA	NA	NA	########	NA
NA	NA	NA	########	NA	NA	NA	########	NA
NA	########	########	########	NA	########	########	########	NA
########	NA	########	########	NA	NA	########	########	########
NA	NA	NA	NA	NA	NA	NA	NA	NA
NA	NA	########	########	NA	NA	NA	NA	NA
NA	NA	########	########	NA	NA	########	########	NA
NA	########	########	########	NA	########	########	########	########
NA	NA	########	########	NA	NA	########	########	NA
NA	NA	NA	NA	NA	NA	NA	NA	########
NA	NA	NA	NA	NA	NA	NA	NA	NA
NA	NA	NA	NA	NA	NA	NA	NA	NA
NA	NA	NA	NA	NA	NA	NA	NA	NA
NA	NA	########	########	NA	########	########	########	NA
NA	########	########	########	NA	########	########	########	NA
NA	NA	########	NA	NA	########	########	NA	########
NA	NA	NA	########	NA	NA	NA	########	NA
NA	NA	NA	NA	NA	NA	NA	NA	NA
NA	NA	NA	NA	NA	NA	NA	NA	NA
########	########	########	########	########	########	########	########	########
NA	NA	NA	########	NA	NA	NA	########	NA
NA	NA	NA	########	NA	NA	NA	########	NA
########	########	NA	########	########	########	NA	########	NA
NA	NA	########	########	NA	NA	NA	########	NA
NA	########	########	########	NA	########	########	########	NA
NA	NA	NA	NA	NA	NA	NA	NA	NA
NA	NA	NA	########	NA	NA	NA	########	NA
NA	NA	NA	########	NA	NA	NA	NA	NA
NA	NA	NA	NA	NA	NA	NA	NA	NA

For Review Only

NA	NA	NA	########	NA	NA	NA	########	NA
NA	NA	NA	NA	NA	NA	NA	NA	NA
########	########	########	########	########	########	########	########	########
NA	NA	NA	NA	NA	NA	NA	NA	NA
NA	NA	NA	########	NA	NA	NA	########	NA
########	########	########	########	NA	NA	########	########	NA
########	########	########	NA	########	########	########	NA	NA
########	NA	NA	NA	########	NA	NA	NA	########
NA	NA	########	########	NA	NA	NA	########	NA
NA	NA	NA	########	NA	NA	NA	########	NA
NA	NA	NA	NA	NA	NA	NA	NA	NA
NA	########	########	########	########	########	########	########	########
NA	NA	########	########	NA	NA	########	########	NA
NA	########	########	########	########	########	########	########	########
NA	NA	NA	NA	NA	NA	NA	NA	NA
NA	########	########	NA	########	########	########	NA	########
NA	NA	NA	########	NA	NA	NA	########	NA
NA	NA	########	NA	NA	NA	########	NA	NA
########	########	########	########	NA	NA	NA	NA	NA
NA	NA	NA	NA	NA	NA	NA	NA	NA
NA	NA	NA	NA	NA	NA	NA	NA	NA
NA	NA	########	NA	NA	NA	########	NA	NA
NA	########	########	########	NA	########	########	########	########
NA	########	NA	########	NA	########	########	NA	NA
NA	NA	########	########	NA	NA	NA	NA	NA
NA	NA	NA	NA	NA	NA	NA	NA	NA
########	########	NA	NA	NA	NA	NA	########	NA
NA	NA	NA	########	NA	NA	NA	########	NA
NA	NA	NA	########	NA	NA	NA	########	NA
NA	NA	NA	NA	NA	NA	NA	NA	NA
NA	########	########	########	NA	########	########	########	########
NA	NA	NA	########	NA	NA	NA	########	NA
NA	NA	NA	NA	NA	NA	NA	NA	NA
NA	NA	NA	########	NA	NA	NA	########	NA
NA	NA	NA	NA	NA	NA	NA	NA	NA
NA	NA	########	########	NA	NA	NA	########	NA
########	########	NA	NA	########	########	NA	NA	NA
NA	NA	NA	########	NA	NA	NA	########	NA
NA	NA	NA	########	NA	NA	NA	########	NA
NA	########	########	NA	NA	NA	########	NA	NA
NA	NA	NA	NA	NA	NA	NA	NA	NA
NA	########	########	########	########	########	########	########	########
NA	NA	########	########	NA	NA	NA	########	NA
NA	NA	NA	NA	NA	NA	NA	NA	NA
NA	NA	NA	########	NA	NA	NA	########	NA
NA	NA	NA	NA	NA	NA	NA	########	NA
########	NA	NA	NA	NA	########	########	########	########
NA	########	########	########	NA	########	########	########	NA
NA	NA	NA	NA	NA	NA	NA	NA	NA
NA	NA	NA	NA	NA	NA	########	NA	NA
NA	NA	NA	NA	NA	NA	NA	NA	NA
NA	NA	NA	########	NA	NA	NA	NA	NA
NA	NA	NA	NA	NA	NA	NA	NA	NA
NA	########	########	########	NA	########	NA	########	########
NA	NA	NA	NA	NA	NA	NA	NA	NA
NA	NA	NA	########	NA	NA	NA	########	NA

For Review Only

NA	NA	NA	NA	NA	NA	NA	NA	NA
NA	NA	########	NA	NA	NA	########	NA	NA
NA	NA	########	########	NA	NA	########	########	NA
########	NA	NA	########	NA	NA	NA	########	NA
NA	NA	NA	NA	NA	NA	NA	NA	NA
NA	NA	########	########	NA	NA	NA	########	NA
NA	NA	NA	########	NA	NA	NA	########	NA
NA	NA	NA	########	NA	NA	NA	########	NA
NA	NA	NA	NA	NA	NA	NA	NA	NA
NA	NA	########	########	NA	########	########	########	NA
NA	NA	NA	########	NA	NA	NA	########	NA
########	NA	NA	NA	NA	NA	NA	########	NA
NA	NA	########	NA	NA	NA	########	NA	NA
NA	NA	NA	NA	########	########	########	########	NA
NA	########	NA	NA	NA	NA	NA	NA	NA
########	NA	########	NA	NA	NA	########	########	NA
NA	NA	NA	########	NA	NA	NA	########	NA
NA	NA	NA	NA	NA	NA	NA	NA	NA
NA	NA	NA	NA	NA	NA	NA	NA	NA
NA	NA	NA	########	NA	NA	NA	########	NA
NA	NA	NA	NA	NA	NA	NA	NA	NA
NA	NA	NA	########	NA	NA	NA	########	NA
NA	########	########	NA	NA	########	########	########	NA
NA	NA	NA	########	NA	NA	########	########	########
NA	NA	NA	NA	NA	NA	NA	########	NA
NA	NA	########	########	NA	NA	########	########	NA
NA	NA	NA	########	NA	NA	NA	########	NA
NA	NA	NA	########	NA	NA	NA	########	NA
NA	NA	########	########	NA	NA	########	########	NA
NA	########	########	NA	########	########	########	NA	NA
NA	NA	NA	NA	NA	NA	NA	NA	NA
########	########	########	########	########	########	########	########	NA
NA	NA	NA	NA	NA	NA	NA	NA	NA
NA	NA	NA	########	NA	NA	NA	########	NA
NA	NA	NA	NA	NA	NA	NA	NA	NA
NA	NA	NA	NA	########	########	########	########	NA
NA	NA	NA	########	NA	NA	NA	########	NA
NA	NA	NA	NA	NA	NA	NA	NA	NA
########	########	########	########	########	########	########	########	NA
NA	NA	NA	NA	NA	NA	NA	NA	NA
NA	NA	NA	########	NA	NA	########	########	NA
NA	NA	########	########	NA	NA	########	########	NA
NA	########	########	########	NA	########	########	########	########
NA	########	########	########	########	########	########	########	########
########	NA	NA	NA	NA	NA	NA	NA	NA
NA	NA	NA	NA	NA	NA	NA	NA	NA
########	########	########	########	NA	NA	NA	########	NA
NA	NA	NA	NA	NA	NA	NA	NA	NA
NA	NA	########	########	NA	########	########	########	NA
########	########	########	########	########	########	########	########	NA
NA	NA	NA	NA	NA	NA	NA	NA	NA
NA	NA	NA	########	NA	NA	NA	########	NA
NA	NA	########	########	NA	NA	########	########	NA
NA	NA	NA	NA	NA	NA	NA	NA	NA
NA	########	########	########	NA	########	########	########	NA
NA	NA	NA	########	NA	NA	NA	########	NA

For Review Only

NA	NA	NA	NA	NA	########	########	########	NA
########	########	########	########	NA	########	########	########	NA
NA	NA	NA	NA	########	########	########	########	NA
NA	NA	########	########	NA	NA	NA	########	NA
NA	NA	NA	NA	NA	NA	NA	NA	NA
NA	########	########	########	NA	########	########	########	########
NA	########	########	########	NA	########	########	########	NA
NA	########	########	########	########	########	########	########	########
NA	########	########	NA	NA	NA	NA	NA	NA
########	########	########	########	########	########	########	########	NA
########	########	NA	NA	NA	########	NA	NA	NA
NA	NA	NA	NA	NA	NA	NA	NA	NA
NA	NA	NA	NA	NA	NA	NA	NA	NA
NA	########	########	########	NA	########	########	########	NA
NA	NA	NA	NA	NA	NA	NA	########	NA
NA	NA	NA	########	NA	NA	NA	########	########
NA	NA	NA	NA	NA	NA	NA	NA	NA
NA	NA	########	########	NA	NA	NA	########	NA
NA	########	########	NA	NA	NA	NA	NA	NA
NA	NA	NA	NA	NA	NA	NA	NA	NA
NA	NA	########	NA	NA	NA	NA	NA	NA
NA	NA	NA	########	NA	NA	NA	########	NA
NA	NA	NA	NA	NA	NA	NA	########	NA
NA	NA	NA	########	NA	NA	NA	########	NA
########	########	########	NA	########	########	########	NA	NA
NA	NA	NA	NA	NA	NA	NA	NA	NA
NA	########	########	########	NA	########	########	########	NA
########	########	NA	########	########	########	NA	########	NA
NA	########	########	########	NA	########	########	########	########
NA	NA	NA	########	NA	NA	NA	########	NA
NA	########	########	########	NA	NA	NA	########	NA
########	########	NA	NA	########	########	NA	########	NA
NA	NA	NA	NA	NA	NA	NA	NA	NA
NA	NA	NA	########	NA	NA	NA	########	NA
NA	NA	########	NA	NA	NA	########	NA	NA
NA	NA	NA	########	NA	NA	NA	########	NA
NA	NA	NA	NA	NA	NA	NA	NA	NA
NA	NA	NA	NA	########	########	########	########	NA
NA	NA	########	NA	NA	NA	########	NA	NA
NA	########	########	########	NA	########	########	########	NA
NA	########	########	########	NA	########	########	########	NA
NA	NA	NA	NA	NA	NA	NA	NA	NA
NA	NA	NA	NA	NA	NA	NA	NA	NA
NA	NA	NA	########	NA	NA	########	########	NA
NA	########	########	########	NA	########	########	########	########
NA	NA	NA	NA	NA	NA	NA	NA	NA
NA	NA	NA	########	NA	NA	NA	########	NA
NA	NA	NA	########	NA	NA	NA	########	NA
NA	NA	NA	########	NA	NA	NA	NA	NA
NA	NA	NA	NA	NA	NA	NA	NA	NA
NA	########	########	########	########	########	########	########	NA
NA	NA	NA	NA	NA	NA	NA	NA	NA
NA	NA	NA	########	NA	NA	NA	########	NA
NA	NA	NA	NA	NA	NA	NA	NA	NA
NA	NA	NA	########	NA	NA	NA	########	NA
NA	NA	########	########	NA	NA	########	########	########

For Review Only

NA	########	########	########	NA	########	########	########	NA
########	NA	NA	########	NA	NA	NA	########	NA
NA	NA	NA	########	NA	NA	NA	########	NA
########	########	NA	########	########	########	NA	########	NA
NA	NA	########	NA	NA	NA	########	NA	NA
NA	NA	NA	########	NA	NA	NA	########	NA
NA	NA	########	########	NA	NA	########	########	########
########	########	########	########	########	########	########	NA	NA
NA	NA	NA	NA	NA	NA	NA	NA	NA
NA	NA	NA	########	NA	NA	NA	########	NA
NA	NA	NA	NA	NA	NA	NA	NA	NA
NA	NA	NA	NA	NA	NA	NA	NA	NA
NA	NA	NA	########	NA	NA	########	########	NA
NA	NA	NA	NA	NA	NA	NA	NA	NA
NA	########	########	########	########	########	########	########	NA
NA	NA	NA	NA	NA	NA	NA	NA	NA
NA	NA	NA	########	NA	NA	NA	########	NA
NA	NA	NA	########	NA	NA	NA	########	NA
NA	NA	NA	NA	NA	NA	NA	NA	NA
NA	NA	NA	NA	NA	NA	NA	NA	NA
NA	NA	NA	NA	NA	NA	NA	NA	NA
NA	NA	NA	NA	NA	NA	NA	NA	NA
NA	NA	########	########	NA	NA	########	########	NA
NA	NA	NA	########	NA	NA	NA	########	NA
NA	NA	NA	########	NA	NA	NA	########	NA
NA	NA	NA	NA	NA	NA	NA	NA	NA
NA	NA	########	NA	NA	NA	########	NA	NA
NA	NA	########	NA	NA	NA	########	NA	NA
NA	NA	NA	NA	NA	NA	NA	NA	NA
NA	NA	NA	########	NA	NA	NA	########	NA
NA	NA	NA	########	NA	NA	########	########	NA
########	NA	NA	########	########	NA	NA	########	NA
NA	NA	NA	########	NA	NA	NA	########	NA
NA	NA	NA	########	NA	NA	NA	########	NA
NA	NA	NA	########	NA	NA	NA	########	NA
NA	NA	NA	########	NA	NA	NA	########	NA
NA	NA	NA	NA	NA	NA	NA	NA	NA
NA	NA	NA	NA	NA	NA	NA	NA	########
NA	NA	NA	NA	NA	NA	NA	########	NA
NA	NA	NA	NA	NA	NA	NA	NA	NA
NA	########	########	NA	NA	NA	########	########	########
########	########	########	########	NA	########	########	########	NA
NA	NA	NA	########	NA	NA	NA	NA	NA
NA	NA	NA	NA	NA	NA	NA	########	NA
NA	NA	NA	NA	NA	NA	NA	NA	NA
NA	NA	NA	########	NA	NA	NA	########	NA
NA	########	########	########	########	########	########	########	########
########	NA	NA	NA	NA	NA	NA	########	NA
########	########	NA	NA	NA	########	NA	########	NA
NA	NA	########	########	NA	########	########	########	########
NA	NA	########	########	NA	########	########	########	########
NA	NA	########	NA	NA	NA	########	NA	NA
NA	NA	########	NA	NA	NA	########	NA	NA
NA	NA	NA	NA	NA	NA	NA	########	NA
########	########	########	########	NA	NA	########	########	NA
NA	########	NA	NA	NA	########	NA	NA	NA

For Review Only

NA	NA	NA	########	NA	NA	NA	########	NA
NA	NA	NA	NA	NA	NA	NA	NA	NA
NA	NA	NA	NA	NA	NA	NA	NA	NA
NA	NA	NA	########	NA	NA	NA	########	NA
NA	NA	########	NA	NA	NA	########	NA	NA
########	NA	NA	NA	NA	NA	NA	NA	NA
NA	NA	NA	NA	NA	########	########	########	NA
NA	########	NA	NA	NA	########	NA	NA	NA
NA	NA	NA	NA	NA	NA	NA	NA	NA
NA	NA	NA	NA	NA	NA	NA	########	NA
########	########	NA	NA	NA	NA	NA	NA	NA
NA	########	NA	NA	NA	NA	NA	NA	NA
########	NA	########	########	########	NA	NA	########	########
NA	NA	NA	NA	########	########	########	########	NA
NA	########	NA	########	NA	NA	NA	########	NA
NA	NA	NA	NA	NA	NA	NA	NA	NA
NA	########	########	########	########	########	########	########	NA
NA	NA	########	NA	NA	NA	########	NA	NA
NA	NA	########	########	NA	NA	########	########	NA
NA	########	########	########	NA	NA	NA	NA	NA
NA	NA	NA	########	NA	NA	NA	########	NA
NA	NA	NA	########	NA	NA	NA	NA	NA
NA	NA	NA	NA	NA	NA	NA	NA	NA
NA	NA	NA	NA	NA	NA	NA	NA	NA
NA	NA	NA	NA	NA	NA	NA	NA	NA
NA	NA	NA	NA	NA	NA	NA	NA	NA
NA	NA	NA	########	NA	NA	NA	########	NA
NA	NA	NA	NA	NA	NA	NA	########	NA
NA	NA	NA	NA	NA	NA	NA	NA	NA
NA	NA	NA	NA	NA	NA	NA	NA	NA
########	########	########	########	########	########	########	########	########
NA	NA	NA	NA	NA	NA	########	########	NA
NA	NA	NA	NA	NA	NA	NA	NA	NA
NA	NA	NA	########	NA	NA	NA	########	NA
NA	NA	NA	########	NA	NA	NA	########	NA
NA	NA	NA	NA	NA	NA	########	NA	NA
NA	NA	########	########	NA	NA	########	########	NA
########	NA	NA	########	########	NA	NA	########	NA
NA	NA	NA	########	NA	NA	NA	########	NA
NA	NA	NA	NA	NA	NA	NA	NA	NA
NA	NA	NA	NA	NA	NA	NA	NA	NA
NA	########	########	########	NA	########	########	########	########
NA	NA	########	########	NA	NA	NA	NA	NA
NA	########	########	########	NA	NA	########	########	NA
NA	NA	NA	########	NA	NA	NA	########	NA
NA	NA	NA	########	NA	NA	NA	########	NA
NA	NA	########	########	NA	NA	########	########	NA
NA	########	NA	NA	NA	########	NA	NA	NA
NA	NA	NA	########	NA	NA	NA	########	NA
NA	NA	NA	NA	NA	NA	NA	NA	NA
NA	NA	########	########	NA	NA	NA	NA	NA
NA	NA	NA	NA	NA	NA	NA	NA	NA
NA	NA	########	NA	NA	NA	########	NA	NA
NA	NA	NA	NA	NA	NA	NA	NA	NA
NA	NA	NA	########	NA	########	########	########	NA
NA	NA	NA	NA	NA	NA	NA	NA	NA

For Review Only

NA	########	########	########	NA	NA	########	########	NA
NA	NA	########	########	NA	########	########	########	########
NA	NA	NA	NA	NA	NA	NA	NA	NA
########	########	########	########	########	########	########	########	NA
NA	NA	NA	NA	NA	NA	NA	NA	NA
NA	NA	NA	NA	NA	NA	NA	NA	NA
NA	NA	NA	NA	NA	NA	NA	NA	NA
NA	########	########	########	NA	NA	########	########	NA
NA	NA	NA	NA	NA	NA	NA	NA	NA
NA	NA	########	NA	NA	NA	########	NA	NA
NA	NA	NA	NA	NA	NA	NA	NA	NA
NA	NA	########	NA	NA	NA	########	NA	NA
NA	########	########	########	NA	########	########	########	NA
NA	NA	########	NA	NA	NA	########	NA	NA
NA	NA	NA	NA	NA	NA	NA	NA	NA
NA	########	########	########	NA	########	########	########	########
NA	########	########	########	NA	NA	########	########	NA
NA	NA	########	########	NA	NA	########	########	NA
NA	########	########	########	NA	NA	NA	NA	NA
NA	NA	NA	NA	NA	NA	NA	NA	NA
NA	NA	NA	NA	NA	NA	NA	NA	NA
NA	NA	NA	NA	NA	NA	NA	NA	NA
NA	NA	NA	########	NA	NA	NA	########	NA
########	########	########	########	########	########	########	########	NA
NA	NA	NA	########	NA	NA	NA	########	NA
NA	NA	########	NA	NA	NA	########	NA	NA
########	NA	NA	NA	########	NA	NA	NA	########
NA	NA	NA	NA	NA	NA	NA	NA	NA
NA	NA	########	NA	NA	NA	########	NA	NA
NA	NA	NA	NA	NA	NA	NA	NA	NA
NA	NA	NA	NA	NA	NA	NA	NA	NA
NA	NA	NA	NA	NA	NA	NA	NA	NA
########	########	########	NA	NA	########	########	NA	########
NA	NA	NA	########	NA	NA	NA	########	NA
NA	NA	########	########	NA	NA	########	########	NA
NA	########	########	########	NA	NA	########	########	NA
NA	NA	NA	NA	NA	NA	NA	NA	NA
NA	NA	########	########	NA	NA	########	########	NA
NA	NA	NA	NA	NA	NA	NA	NA	NA
NA	########	########	########	NA	########	########	########	########
########	NA	NA	########	########	NA	NA	########	########
NA	NA	NA	NA	NA	NA	NA	NA	NA
NA	NA	NA	########	NA	NA	NA	########	NA
########	########	########	NA	NA	########	NA	NA	NA
NA	NA	NA	########	NA	NA	NA	########	NA
NA	NA	NA	########	NA	NA	NA	########	NA
NA	NA	NA	NA	NA	NA	NA	NA	NA
NA	NA	NA	########	NA	NA	NA	########	NA
NA	NA	########	########	NA	NA	########	########	NA
NA	NA	NA	NA	NA	NA	NA	NA	NA
NA	NA	NA	NA	NA	NA	NA	########	NA
NA	NA	########	NA	NA	NA	########	NA	NA

For Review Only

NA	########	########	########	NA	NA	########	########	NA
NA	NA	NA	########	NA	NA	NA	########	NA
NA	NA	NA	NA	NA	NA	NA	NA	NA
NA	NA	NA	########	NA	NA	NA	########	NA
NA	NA	NA	########	NA	NA	NA	########	NA
NA	NA	NA	NA	NA	NA	NA	NA	NA
NA	NA	########	########	NA	NA	NA	########	NA
NA	NA	NA	NA	NA	NA	NA	NA	NA
NA	NA	NA	########	NA	NA	NA	########	NA
NA	NA	########	########	NA	NA	NA	########	NA
NA	########	NA	NA	NA	########	NA	NA	NA
########	NA	NA	########	NA	NA	NA	########	NA
NA	NA	NA	########	NA	NA	NA	########	NA
NA	NA	NA	NA	NA	########	NA	NA	NA
NA	NA	########	########	NA	NA	NA	########	NA
NA	NA	NA	########	NA	NA	NA	########	NA
NA	NA	NA	########	NA	NA	NA	########	NA
NA	NA	NA	NA	NA	NA	NA	NA	NA
NA	NA	########	########	NA	NA	########	########	NA
########	########	########	NA	########	########	########	NA	NA
NA	NA	NA	NA	NA	NA	NA	NA	NA
NA	NA	########	########	NA	NA	########	########	NA
NA	NA	NA	NA	NA	NA	NA	NA	NA
NA	NA	NA	########	NA	NA	NA	########	NA
NA	NA	NA	########	NA	NA	NA	########	NA
NA	NA	NA	NA	NA	NA	NA	NA	NA
NA	NA	NA	NA	NA	NA	NA	NA	NA
NA	NA	NA	NA	NA	NA	NA	NA	NA
NA	NA	NA	########	NA	NA	NA	########	NA
NA	NA	########	########	NA	NA	########	########	########
NA	NA	NA	NA	NA	NA	NA	NA	NA
NA	NA	NA	NA	NA	########	NA	NA	NA
NA	########	########	########	NA	########	########	########	NA
NA	########	########	NA	NA	########	########	NA	NA
NA	NA	NA	NA	########	########	########	########	NA
########	NA	NA	NA	########	NA	NA	NA	NA
NA	########	########	########	########	########	########	########	NA
NA	NA	NA	########	########	########	########	########	NA
NA	NA	NA	########	NA	NA	NA	########	NA
NA	########	########	########	########	########	########	########	########
########	########	NA	NA	########	NA	NA	########	########
NA	NA	########	########	NA	NA	########	########	NA
########	########	########	########	########	########	########	########	########
########	########	########	########	NA	########	########	########	NA
NA	NA	NA	########	NA	NA	NA	########	NA
NA	########	########	########	NA	########	########	########	########
NA	NA	NA	########	NA	NA	NA	########	NA
NA	NA	NA	NA	NA	NA	NA	NA	NA
NA	NA	NA	NA	NA	NA	NA	NA	NA
########	########	########	NA	########	########	########	NA	########
NA	NA	NA	########	NA	NA	NA	########	NA
NA	NA	########	########	NA	NA	########	########	NA
NA	NA	########	########	NA	NA	########	########	NA
NA	########	########	########	NA	########	########	########	########
NA	NA	NA	########	NA	NA	NA	########	NA
NA	NA	NA	NA	NA	########	########	########	NA

For Review Only

NA	NA	NA	########	NA	NA	NA	########	NA
NA	NA	########	########	NA	NA	########	########	NA
NA	NA	########	NA	NA	NA	########	NA	NA
NA	NA	NA	########	NA	NA	NA	########	NA
NA	NA	########	########	NA	NA	########	########	NA
NA	########	########	NA	NA	########	########	NA	NA
########	NA	NA	NA	NA	NA	NA	########	NA
NA	NA	NA	NA	NA	NA	NA	NA	NA
NA	NA	########	########	NA	NA	########	########	NA
########	########	########	########	########	########	########	########	NA
NA	NA	NA	NA	NA	NA	NA	NA	NA
NA	NA	########	NA	NA	NA	########	NA	NA
NA	NA	NA	########	NA	NA	NA	########	NA
NA	NA	NA	########	NA	NA	NA	########	NA
NA	NA	NA	NA	NA	NA	NA	NA	NA
NA	NA	NA	NA	NA	NA	NA	########	NA
NA	NA	########	########	NA	NA	########	########	NA
NA	NA	NA	NA	NA	NA	NA	NA	NA
NA	########	NA	NA	NA	########	NA	NA	########
NA	NA	NA	NA	NA	NA	NA	NA	NA
########	########	########	########	########	########	########	########	########
NA	NA	NA	NA	NA	########	########	########	NA
########	########	########	########	########	########	########	########	NA
NA	NA	NA	NA	NA	NA	NA	NA	NA
NA	NA	NA	########	NA	NA	NA	########	NA
NA	NA	NA	NA	NA	NA	NA	NA	NA
NA	NA	NA	########	NA	NA	NA	########	NA
NA	NA	NA	########	NA	NA	NA	########	NA
NA	NA	NA	NA	NA	NA	NA	NA	NA
NA	NA	NA	########	NA	NA	NA	########	NA
NA	NA	NA	########	NA	NA	NA	NA	NA
NA	NA	NA	NA	NA	NA	NA	NA	NA
NA	NA	NA	########	NA	NA	NA	########	NA
NA	NA	NA	NA	NA	NA	NA	NA	NA
NA	NA	NA	########	NA	NA	NA	########	NA
NA	NA	NA	########	NA	NA	NA	########	NA
NA	########	########	########	########	########	########	########	NA
NA	NA	NA	NA	NA	NA	NA	NA	NA
########	########	NA	NA	########	########	NA	NA	NA
NA	NA	########	########	NA	NA	########	########	NA
NA	NA	########	NA	NA	NA	########	########	########
NA	########	NA	########	NA	########	########	########	NA
NA	NA	########	NA	NA	NA	########	NA	NA
########	########	########	########	########	########	########	########	########
NA	NA	########	########	NA	NA	########	########	NA
########	NA	NA	NA	NA	NA	NA	########	NA
NA	NA	NA	########	NA	NA	########	########	NA
NA	NA	NA	NA	NA	########	########	########	NA
NA	NA	NA	########	NA	NA	NA	########	NA
NA	NA	NA	########	NA	NA	NA	########	NA
NA	########	########	########	########	########	########	########	########
NA	NA	########	NA	NA	NA	########	NA	NA
NA	NA	NA	########	NA	NA	########	########	NA
NA	########	########	NA	NA	########	########	NA	NA
NA	NA	NA	NA	NA	NA	NA	NA	NA

For Review Only

NA	NA	NA	NA	NA	NA	NA	NA	NA
NA	NA	NA	NA	NA	NA	NA	NA	NA
NA	NA	########	NA	NA	NA	########	NA	NA
NA	NA	NA	########	NA	NA	NA	########	NA
NA	NA	NA	########	NA	NA	NA	########	NA
NA	NA	########	########	NA	NA	########	########	########
NA	NA	NA	########	NA	NA	NA	########	NA
NA	NA	NA	########	NA	NA	NA	########	NA
NA	NA	NA	NA	NA	NA	NA	NA	NA
NA	NA	NA	NA	NA	NA	NA	NA	NA
########	########	########	########	########	########	########	########	########
NA	NA	NA	########	NA	NA	NA	########	NA
NA	NA	NA	NA	NA	NA	NA	NA	NA
########	########	########	########	NA	NA	NA	NA	NA
NA	########	########	########	NA	NA	########	########	NA
NA	NA	########	########	NA	NA	########	########	NA
NA	NA	########	########	NA	NA	NA	########	NA
NA	NA	NA	########	NA	NA	NA	########	NA
NA	NA	NA	NA	NA	NA	NA	NA	NA
NA	NA	########	########	NA	NA	NA	NA	NA
NA	########	NA	NA	NA	NA	NA	########	NA
########	########	########	########	########	########	########	########	NA
NA	NA	NA	########	NA	NA	NA	########	NA
NA	NA	########	########	NA	NA	NA	########	NA
NA	NA	NA	NA	NA	NA	NA	NA	NA
NA	NA	########	########	NA	NA	NA	NA	NA
NA	NA	NA	NA	NA	NA	NA	NA	NA
NA	########	NA	NA	NA	########	NA	NA	NA
NA	NA	NA	NA	NA	NA	NA	NA	NA
NA	########	NA	########	NA	NA	NA	########	NA
########	########	########	########	########	########	########	########	NA
NA	########	########	########	########	########	########	########	NA
NA	NA	########	########	NA	NA	########	########	NA
NA	NA	NA	NA	NA	NA	NA	NA	NA
NA	NA	NA	NA	NA	NA	NA	NA	NA
########	NA	NA	NA	NA	NA	NA	NA	########
NA	NA	NA	NA	NA	NA	NA	NA	NA
NA	NA	NA	########	NA	NA	NA	########	NA
########	########	########	########	NA	########	########	########	NA
NA	NA	NA	########	NA	NA	NA	NA	NA
NA	NA	NA	########	NA	NA	NA	########	NA
NA	NA	########	########	NA	NA	########	########	########
NA	NA	NA	NA	NA	NA	NA	NA	NA
NA	########	########	NA	NA	########	########	NA	########
NA	########	########	########	NA	########	########	########	########
NA	NA	########	########	NA	NA	NA	########	NA
NA	NA	NA	NA	NA	NA	NA	NA	NA
NA	NA	########	########	NA	NA	########	########	NA
NA	NA	NA	########	NA	NA	NA	########	NA
NA	NA	########	########	NA	NA	########	########	NA
NA	NA	########	########	NA	NA	NA	########	NA
NA	NA	NA	NA	NA	NA	NA	NA	NA
NA	NA	NA	########	NA	NA	NA	########	NA
NA	NA	NA	########	NA	NA	NA	########	NA
NA	########	########	NA	NA	NA	########	NA	NA
########	########	########	########	########	########	########	########	NA

For Review Only

NA	NA	########	NA	NA	NA	########	########	NA
NA	NA	NA	NA	NA	NA	NA	NA	NA
NA	NA	NA	########	NA	NA	NA	########	NA
NA	NA	NA	########	NA	NA	NA	########	NA
NA	NA	########	NA	NA	NA	########	NA	NA
NA	NA	NA	########	NA	NA	NA	########	NA
NA	NA	NA	########	NA	NA	NA	########	NA
NA	NA	NA	NA	NA	NA	NA	NA	NA
########	########	########	########	########	########	########	########	########
NA	NA	NA	NA	NA	NA	NA	NA	NA
NA	########	########	########	NA	########	########	########	########
NA	NA	NA	########	NA	NA	NA	########	NA
NA	NA	NA	NA	NA	NA	NA	NA	NA
NA	########	########	########	NA	########	########	########	NA
NA	########	########	########	########	########	########	########	########
NA	NA	NA	########	NA	NA	NA	########	NA
NA	NA	NA	########	NA	NA	########	NA	NA
NA	NA	########	########	NA	NA	NA	########	NA
NA	NA	NA	NA	NA	NA	NA	NA	NA
NA	NA	NA	########	NA	NA	NA	########	NA
########	########	########	########	########	########	########	########	NA
NA	NA	NA	NA	NA	NA	NA	NA	NA
NA	NA	########	########	NA	NA	NA	########	NA
########	########	########	NA	########	########	########	NA	NA
########	NA	NA	NA	NA	NA	NA	NA	NA
NA	########	########	########	NA	NA	NA	NA	NA
NA	NA	NA	NA	NA	NA	NA	NA	NA
NA	NA	NA	NA	NA	NA	NA	NA	NA
NA	NA	########	########	NA	########	########	########	NA
NA	NA	NA	########	NA	NA	NA	########	NA
NA	NA	NA	NA	NA	NA	NA	NA	NA
NA	NA	NA	NA	NA	NA	NA	NA	NA
NA	NA	NA	NA	NA	NA	NA	NA	NA
NA	########	########	NA	NA	NA	########	NA	NA
NA	NA	NA	NA	NA	NA	NA	########	NA
NA	NA	NA	NA	NA	NA	NA	NA	NA
NA	NA	########	########	NA	NA	NA	########	NA
########	########	########	########	########	########	########	########	########
NA	########	NA	NA	NA	NA	NA	NA	NA
########	########	########	########	########	########	########	########	########
NA	NA	NA	NA	NA	NA	NA	########	NA
NA	NA	########	########	NA	NA	NA	########	NA
NA	NA	NA	NA	NA	NA	NA	NA	NA
NA	NA	NA	NA	NA	NA	NA	NA	NA
########	NA	NA	NA	NA	NA	NA	NA	NA
NA	NA	NA	########	NA	NA	NA	########	NA
NA	NA	NA	########	NA	NA	NA	########	NA
NA	NA	NA	NA	NA	NA	NA	NA	NA
NA	NA	NA	########	NA	NA	NA	########	NA
NA	NA	NA	########	NA	NA	NA	########	NA
NA	NA	NA	########	NA	NA	NA	NA	NA
NA	NA	NA	NA	NA	NA	NA	NA	NA
########	########	########	########	########	########	########	########	NA
########	########	########	########	########	########	NA	########	NA
########	########	NA	########	########	########	NA	########	NA
NA	NA	NA	NA	NA	NA	NA	NA	NA

For Review Only

NA	NA	NA	NA	NA	NA	NA	NA	NA
########	########	########	########	########	########	########	########	NA
NA	########	########	NA	NA	NA	NA	NA	NA
########	NA	NA	########	########	NA	NA	########	NA
NA	NA	NA	NA	NA	NA	NA	NA	NA
NA	########	########	########	NA	########	########	########	NA
NA	NA	NA	########	NA	NA	NA	########	NA
NA	NA	NA	NA	NA	########	NA	NA	########
NA	NA	########	########	NA	NA	NA	NA	NA
NA	########	########	NA	NA	########	NA	NA	NA
NA	NA	########	########	NA	NA	NA	########	NA
########	NA	NA	########	########	NA	NA	########	########
NA	NA	NA	########	NA	NA	NA	########	NA
NA	NA	NA	NA	NA	NA	NA	NA	NA
########	########	########	########	########	########	########	########	########
NA	########	########	########	NA	########	########	########	NA
NA	NA	NA	NA	NA	NA	NA	NA	NA
NA	NA	NA	NA	NA	NA	NA	NA	NA
########	########	########	########	########	########	########	########	NA
NA	NA	NA	NA	NA	NA	NA	NA	NA
NA	NA	NA	########	NA	NA	NA	########	NA
########	########	NA	NA	NA	NA	NA	NA	NA
NA	NA	########	########	NA	NA	NA	########	NA
NA	NA	NA	NA	NA	NA	NA	NA	NA
NA	########	########	########	########	########	########	########	########
NA	NA	NA	########	NA	NA	NA	########	NA
########	########	########	########	########	########	########	########	########
NA	########	########	NA	NA	########	########	NA	########
NA	NA	NA	NA	NA	NA	NA	NA	NA
NA	########	########	########	NA	NA	########	########	NA
NA	NA	########	NA	NA	NA	########	NA	NA
########	########	########	NA	########	########	########	NA	NA
NA	NA	NA	NA	NA	NA	NA	NA	NA
NA	NA	########	NA	NA	NA	########	NA	NA
NA	NA	NA	########	NA	NA	NA	NA	NA
NA	NA	NA	NA	NA	NA	NA	NA	NA
NA	NA	NA	NA	NA	NA	NA	NA	NA
NA	NA	NA	NA	NA	NA	NA	NA	NA
NA	NA	NA	NA	NA	########	########	########	NA
NA	NA	########	########	NA	NA	########	########	NA
NA	NA	NA	########	NA	NA	NA	########	NA
NA	NA	NA	NA	NA	NA	NA	NA	NA
########	########	########	########	########	########	########	########	NA
########	########	########	########	########	########	########	########	NA
NA	NA	NA	########	NA	NA	NA	NA	NA
########	NA	NA	########	NA	NA	########	########	########
NA	NA	NA	NA	NA	NA	NA	NA	NA
NA	NA	NA	NA	NA	NA	NA	NA	NA
NA	NA	NA	########	NA	NA	NA	########	NA
NA	NA	########	########	NA	NA	########	########	NA
NA	NA	########	NA	NA	NA	########	NA	NA
NA	NA	NA	NA	NA	NA	NA	NA	NA
NA	NA	NA	NA	NA	NA	NA	NA	NA
NA	NA	NA	NA	NA	NA	NA	NA	NA
NA	NA	NA	########	NA	NA	NA	########	NA
NA	NA	NA	########	NA	NA	NA	########	NA

For Review Only

NA	NA	NA	NA	NA	NA	NA	NA	NA
NA	NA	NA	NA	NA	NA	NA	NA	NA
NA	NA	########	NA	NA	NA	########	NA	NA
NA	########	########	########	NA	NA	NA	NA	NA
########	########	########	########	########	########	########	########	########
NA	NA	NA	NA	NA	NA	NA	NA	NA
NA	NA	NA	NA	NA	NA	NA	NA	NA
NA	########	NA	########	NA	NA	NA	########	NA
NA	NA	NA	########	NA	NA	NA	########	NA
NA	NA	########	NA	NA	NA	########	NA	NA
NA	NA	NA	########	NA	NA	NA	########	NA
########	########	########	########	########	########	########	########	NA
NA	########	NA	NA	NA	NA	NA	NA	NA
NA	NA	NA	NA	NA	NA	NA	NA	NA
NA	NA	NA	########	NA	NA	NA	########	NA
NA	NA	########	NA	NA	NA	########	NA	NA
NA	########	########	########	NA	########	########	########	NA
NA	NA	########	########	NA	NA	########	########	NA
NA	NA	NA	########	NA	NA	NA	########	NA
########	########	########	########	NA	########	########	########	NA
NA	########	########	########	########	########	########	########	########
NA	NA	NA	########	NA	NA	NA	########	NA
NA	NA	NA	########	########	########	########	########	NA
########	NA	NA	NA	NA	NA	NA	########	NA
NA	NA	NA	########	NA	NA	NA	########	NA
NA	NA	NA	########	NA	NA	NA	########	NA
NA	########	########	########	NA	NA	########	########	NA
NA	NA	NA	NA	NA	NA	NA	########	NA
NA	NA	NA	########	NA	NA	NA	########	NA
NA	NA	NA	########	NA	NA	NA	NA	NA
NA	NA	NA	########	NA	NA	NA	########	NA
NA	NA	NA	########	NA	NA	NA	########	NA
NA	NA	NA	########	NA	NA	NA	########	NA
NA	NA	NA	########	NA	NA	NA	########	NA
NA	NA	NA	NA	NA	NA	NA	NA	NA
########	########	########	NA	NA	########	########	NA	NA
NA	NA	NA	NA	NA	NA	########	########	NA
NA	NA	NA	########	NA	NA	NA	########	NA
NA	NA	NA	########	NA	NA	NA	########	NA
NA	########	########	########	NA	NA	########	########	NA
NA	NA	NA	NA	NA	NA	NA	NA	NA
########	########	########	########	NA	########	########	########	NA
NA	NA	########	########	NA	NA	########	########	########
NA	NA	########	########	NA	NA	########	########	NA
NA	NA	NA	########	NA	NA	NA	########	NA
NA	NA	NA	NA	NA	NA	NA	NA	NA
NA	NA	NA	########	NA	NA	NA	########	NA
NA	NA	NA	NA	NA	NA	NA	NA	NA
NA	NA	########	########	NA	NA	########	########	NA
NA	NA	NA	########	NA	########	########	########	NA
NA	NA	NA	NA	NA	NA	NA	NA	NA
########	########	########	########	NA	NA	########	########	NA
NA	NA	########	NA	NA	NA	########	NA	NA
########	########	########	########	NA	########	########	########	########
########	NA	NA	NA	NA	NA	NA	NA	NA
NA	NA	NA	########	NA	NA	NA	########	NA

For Review Only

NA	NA	NA	########	NA	NA	NA	########	NA
NA	########	########	NA	NA	########	########	NA	NA
NA	NA	NA	########	NA	NA	NA	########	NA
NA	########	########	########	NA	########	########	########	########
NA	NA	NA	########	NA	NA	NA	########	NA
NA	NA	NA	NA	NA	NA	NA	NA	NA
NA	NA	NA	NA	NA	NA	NA	NA	NA
NA	NA	########	########	NA	NA	########	########	NA
########	NA	NA	########	########	NA	########	########	########
NA	NA	########	NA	NA	NA	########	NA	NA
NA	NA	NA	########	NA	NA	NA	########	NA
NA	NA	NA	########	NA	NA	NA	########	NA
########	########	########	########	NA	########	########	########	NA
NA	NA	NA	########	NA	NA	NA	########	NA
NA	NA	NA	NA	NA	NA	NA	NA	NA
NA	NA	NA	########	NA	NA	NA	########	NA
NA	NA	NA	NA	NA	NA	NA	NA	NA
NA	########	########	########	########	########	########	########	NA
NA	NA	NA	NA	NA	NA	NA	NA	NA
NA	NA	NA	NA	NA	NA	NA	NA	########
NA	NA	NA	########	NA	########	########	NA	NA
NA	NA	########	########	NA	NA	########	########	NA
NA	NA	NA	NA	NA	NA	NA	NA	NA
########	########	########	########	########	########	########	########	NA
########	########	########	########	########	########	########	########	########
########	########	########	NA	########	########	########	NA	########
NA	NA	NA	NA	NA	NA	NA	NA	NA
########	########	########	########	########	########	########	########	########
NA	NA	NA	########	NA	NA	NA	########	NA
NA	########	NA	########	NA	NA	NA	########	NA
########	########	########	########	########	########	########	########	########
NA	NA	NA	NA	NA	NA	NA	NA	NA
NA	NA	NA	NA	NA	NA	NA	NA	NA
NA	NA	NA	NA	NA	NA	NA	NA	NA
NA	NA	NA	NA	NA	NA	NA	NA	NA
########	NA	NA	NA	########	NA	########	########	########
NA	NA	NA	########	NA	NA	NA	########	NA
NA	NA	NA	########	NA	NA	NA	########	NA
NA	NA	NA	########	NA	NA	NA	NA	NA
NA	########	########	########	NA	########	########	NA	NA
NA	NA	NA	NA	NA	NA	NA	NA	NA
########	########	########	########	NA	########	########	########	NA
########	########	########	########	########	########	########	########	########
NA	NA	NA	NA	NA	NA	NA	NA	NA
########	########	NA	NA	NA	NA	NA	NA	NA
NA	NA	NA	########	NA	NA	NA	########	NA
NA	NA	NA	NA	NA	NA	NA	NA	NA
NA	NA	NA	NA	NA	NA	NA	NA	NA
NA	NA	NA	NA	NA	NA	NA	NA	NA
NA	NA	NA	NA	NA	NA	NA	NA	NA
NA	NA	NA	########	NA	NA	NA	########	NA
NA	NA	NA	NA	NA	NA	NA	NA	NA
NA	########	########	########	########	########	########	NA	NA
########	########	########	########	NA	########	########	########	NA
NA	NA	NA	NA	NA	NA	NA	NA	NA

For Review Only

NA	NA	########	NA	NA	NA	NA	NA	NA
NA	NA	NA	########	NA	NA	NA	########	NA
NA	NA	NA	########	NA	NA	NA	########	NA
########	########	########	NA	########	########	NA	NA	NA
########	########	########	########	NA	########	########	########	NA
########	########	########	########	########	########	########	########	NA
NA	NA	NA	NA	NA	NA	NA	NA	NA
########	########	NA	########	########	########	NA	########	NA
########	########	########	########	########	########	########	########	########
NA	NA	NA	########	NA	NA	NA	########	NA
NA	NA	NA	NA	NA	NA	NA	NA	NA
NA	NA	NA	########	NA	NA	NA	########	NA
NA	########	########	########	########	########	########	########	########
NA	########	########	########	########	########	########	########	NA
NA	NA	NA	NA	NA	NA	NA	NA	NA
NA	NA	NA	########	NA	NA	NA	NA	NA
NA	NA	NA	########	NA	NA	NA	########	NA
########	########	########	########	########	########	########	########	NA
NA	NA	NA	NA	NA	NA	NA	NA	NA
NA	NA	NA	NA	NA	NA	NA	NA	NA
NA	NA	########	NA	NA	NA	########	NA	NA
NA	NA	########	NA	NA	NA	########	NA	NA
NA	NA	NA	########	NA	NA	NA	########	NA
NA	NA	NA	########	NA	NA	NA	########	NA
########	########	########	########	########	########	########	########	NA
NA	NA	########	########	NA	NA	########	########	NA
NA	NA	NA	########	NA	NA	NA	########	NA
NA	NA	NA	########	NA	NA	NA	########	NA
NA	########	########	########	NA	########	########	########	NA
NA	NA	NA	########	NA	NA	NA	########	NA
NA	########	########	########	NA	NA	NA	########	NA
NA	NA	NA	NA	NA	NA	NA	NA	NA
NA	NA	NA	########	NA	NA	NA	########	NA
NA	NA	NA	########	NA	NA	NA	########	NA
NA	NA	NA	########	NA	NA	NA	NA	NA
NA	NA	NA	NA	NA	NA	NA	NA	NA
NA	NA	NA	NA	NA	NA	NA	NA	NA
NA	NA	NA	NA	NA	NA	NA	NA	NA
########	########	########	########	########	########	########	########	########
NA	NA	NA	NA	NA	NA	NA	NA	NA
NA	NA	NA	NA	NA	NA	NA	NA	NA
NA	NA	NA	NA	NA	NA	NA	NA	NA
NA	NA	NA	NA	NA	NA	NA	NA	NA
NA	NA	NA	########	NA	NA	NA	########	NA
NA	NA	NA	########	NA	NA	########	########	NA
NA	NA	NA	NA	NA	NA	NA	NA	NA
NA	NA	########	########	NA	NA	########	########	NA
NA	NA	NA	NA	NA	NA	NA	NA	NA
NA	NA	########	NA	NA	NA	########	NA	NA
NA	NA	########	NA	NA	NA	########	NA	NA
########	########	########	########	########	########	########	########	########
NA	NA	########	NA	NA	NA	NA	NA	NA
NA	########	########	NA	NA	NA	########	NA	NA
########	########	########	########	########	########	########	########	########
NA	NA	NA	NA	NA	NA	NA	NA	NA

For Review Only

NA	NA	NA	########	NA	########	########	########	########
NA	NA	NA	NA	NA	NA	NA	NA	NA
NA	NA	NA	NA	NA	NA	NA	NA	NA
NA	NA	NA	########	NA	NA	NA	########	NA
########	NA	NA	########	NA	NA	########	########	########
NA	NA	NA	NA	NA	NA	NA	NA	NA
NA	NA	########	NA	NA	NA	NA	NA	NA
NA	########	########	NA	########	########	########	########	########
########	########	########	NA	########	########	########	NA	########
NA	NA	NA	########	NA	NA	NA	NA	NA
NA	NA	########	NA	NA	NA	########	NA	NA
NA	NA	########	########	NA	NA	NA	########	NA
NA	NA	NA	NA	NA	NA	########	########	NA
NA	NA	NA	########	NA	NA	########	########	NA
NA	NA	NA	NA	NA	NA	NA	NA	NA
NA	NA	########	########	NA	NA	########	########	NA
NA	NA	NA	########	NA	NA	NA	########	NA
NA	########	NA	NA	NA	NA	NA	########	NA
NA	########	########	########	NA	########	########	########	########
########	########	########	########	########	########	########	########	########
########	########	########	########	########	########	########	########	########
NA	NA	NA	########	NA	########	NA	########	NA
NA	NA	NA	########	NA	NA	NA	########	NA
########	########	########	########	########	########	########	########	########
NA	########	########	########	########	########	########	########	NA
NA	NA	NA	########	NA	NA	NA	########	NA
NA	NA	NA	########	NA	NA	NA	########	NA
NA	########	########	########	########	########	########	########	NA
NA	########	########	########	NA	########	########	########	########
########	########	########	########	########	########	########	########	########
########	########	########	########	########	########	########	########	NA
NA	NA	NA	########	NA	NA	NA	########	NA
NA	NA	########	NA	NA	NA	NA	NA	NA
NA	NA	NA	NA	NA	NA	NA	NA	NA
NA	NA	NA	########	NA	NA	NA	########	NA
NA	NA	NA	NA	NA	NA	NA	NA	NA
NA	NA	########	########	NA	########	########	########	NA
NA	NA	NA	NA	NA	NA	NA	NA	NA
NA	########	########	########	NA	NA	########	########	NA
########	NA	NA	########	########	NA	########	########	NA
########	########	NA	NA	NA	NA	NA	NA	NA
NA	########	########	########	NA	########	########	########	NA
########	########	########	########	########	########	########	########	########
NA	NA	########	NA	NA	NA	########	NA	NA
NA	NA	NA	NA	NA	NA	NA	NA	NA
NA	########	########	########	NA	########	########	########	NA
NA	NA	NA	NA	NA	NA	NA	NA	NA
NA	NA	NA	########	NA	NA	NA	########	NA
NA	NA	########	NA	NA	NA	########	NA	NA
NA	NA	NA	NA	NA	NA	NA	NA	NA
NA	NA	NA	NA	NA	NA	NA	NA	NA
NA	########	########	########	########	########	########	########	########
NA	NA	NA	########	NA	NA	NA	########	NA
NA	NA	NA	########	NA	NA	NA	########	NA
NA	NA	NA	NA	NA	NA	NA	NA	NA
NA	########	########	########	NA	NA	########	########	NA

For Review Only

NA	NA	########	NA	NA	NA	NA	NA	NA
########	########	########	########	########	########	########	########	NA
NA	NA	NA	########	NA	NA	NA	NA	NA
NA	NA	########	########	NA	NA	########	########	NA
NA	NA	NA	NA	NA	NA	NA	NA	NA
NA	NA	NA	########	NA	NA	NA	########	NA
########	########	########	########	NA	NA	NA	NA	NA
########	########	NA	########	########	NA	NA	########	NA
########	########	########	########	########	########	########	########	########
NA	########	########	########	########	########	########	########	NA
NA	NA	NA	########	NA	NA	NA	########	NA
NA	NA	NA	########	NA	NA	NA	########	NA
NA	NA	NA	NA	NA	NA	NA	NA	NA
NA	NA	NA	NA	NA	NA	NA	NA	NA
########	NA	########	########	NA	########	########	########	########
NA	########	########	########	NA	########	########	########	########
NA	NA	NA	NA	NA	NA	NA	NA	NA
########	NA	NA	NA	########	NA	NA	NA	########
NA	NA	########	NA	NA	NA	NA	NA	NA
NA	NA	NA	########	NA	NA	NA	########	NA
NA	NA	NA	NA	NA	NA	NA	NA	NA
NA	########	########	########	NA	NA	########	########	NA
NA	NA	NA	########	NA	NA	########	########	NA
NA	########	########	########	NA	NA	########	########	NA
NA	NA	########	########	NA	NA	########	########	NA
NA	NA	########	NA	NA	NA	########	NA	NA
NA	NA	NA	NA	NA	NA	########	########	NA
########	########	########	NA	NA	########	########	########	NA
NA	NA	NA	NA	NA	NA	NA	NA	NA
NA	NA	NA	########	NA	NA	NA	########	NA
########	########	########	########	########	########	########	########	########
NA	NA	NA	########	NA	NA	NA	########	NA
NA	NA	NA	########	NA	NA	NA	########	NA
NA	NA	NA	NA	NA	NA	NA	NA	NA
NA	NA	NA	########	NA	NA	NA	########	NA
NA	NA	NA	########	NA	NA	NA	########	NA
NA	NA	NA	########	NA	NA	NA	########	NA
NA	NA	NA	NA	NA	NA	NA	NA	NA
########	NA	NA	########	########	NA	NA	########	NA
NA	########	########	NA	NA	########	########	NA	NA
NA	NA	NA	########	NA	NA	NA	########	NA
NA	########	NA	NA	NA	NA	NA	NA	NA
NA	NA	NA	########	NA	NA	NA	########	NA
NA	NA	########	########	NA	NA	NA	########	NA
NA	NA	NA	NA	NA	NA	NA	NA	NA
NA	NA	NA	########	NA	NA	NA	########	NA
NA	NA	########	########	NA	NA	########	########	NA
NA	NA	NA	NA	NA	NA	NA	NA	NA
NA	NA	########	########	NA	NA	########	########	NA
NA	NA	NA	########	NA	NA	NA	########	NA
NA	NA	NA	NA	NA	########	########	########	NA
NA	NA	NA	########	NA	NA	NA	########	NA
NA	NA	NA	########	NA	NA	NA	########	NA
########	########	########	########	########	########	########	########	########
NA	NA	NA	NA	NA	NA	NA	NA	NA
NA	NA	########	########	NA	NA	########	########	NA

For Review Only

NA	########	########	########	NA	########	########	########	########
NA	NA	NA	NA	NA	NA	NA	NA	NA
NA	NA	NA	########	NA	########	########	########	NA
NA	NA	NA	NA	NA	NA	NA	NA	NA
NA	NA	NA	NA	NA	NA	NA	NA	NA
NA	########	NA	NA	NA	########	NA	NA	NA
NA	########	########	########	########	########	########	########	NA
NA	NA	########	NA	NA	NA	########	NA	NA
NA	########	########	########	########	########	########	########	########
NA	NA	NA	########	NA	NA	NA	########	NA
NA	NA	NA	NA	NA	NA	NA	NA	NA
########	########	########	NA	########	########	########	NA	NA
########	NA	NA	NA	NA	NA	NA	NA	NA
NA	NA	NA	########	NA	NA	NA	########	NA
NA	NA	NA	########	NA	NA	NA	########	NA
NA	########	########	########	NA	########	########	########	########
NA	NA	########	########	NA	NA	########	########	NA
NA	NA	########	########	NA	NA	NA	########	NA
########	########	NA	########	########	########	NA	########	NA
NA	NA	NA	NA	NA	NA	NA	NA	NA
NA	NA	NA	########	NA	NA	NA	########	NA
NA	NA	NA	NA	NA	NA	NA	NA	NA
NA	NA	NA	########	NA	NA	########	########	NA
NA	NA	NA	########	NA	NA	NA	########	NA
NA	NA	NA	NA	NA	NA	NA	NA	NA
NA	NA	NA	########	NA	NA	NA	########	NA
NA	NA	NA	########	NA	NA	NA	########	NA
########	########	########	########	########	########	########	########	########
NA	NA	NA	NA	NA	NA	NA	NA	NA
NA	NA	NA	########	NA	NA	NA	########	NA
NA	NA	NA	NA	NA	NA	########	NA	NA
NA	NA	########	########	NA	NA	########	########	NA
NA	NA	NA	NA	NA	NA	NA	NA	NA
NA	NA	NA	########	NA	NA	NA	########	NA
NA	NA	########	########	NA	NA	########	########	NA
NA	NA	NA	########	NA	NA	NA	########	NA
NA	NA	NA	NA	NA	NA	NA	NA	NA
NA	########	########	NA	NA	########	########	NA	NA
NA	NA	NA	NA	NA	NA	NA	NA	NA
NA	NA	NA	########	NA	NA	NA	########	NA
NA	NA	########	########	NA	NA	NA	NA	NA
NA	NA	NA	########	NA	NA	NA	########	NA
NA	NA	NA	NA	NA	NA	NA	NA	NA
NA	NA	########	########	NA	NA	NA	NA	NA
NA	NA	NA	NA	NA	NA	NA	NA	NA
NA	########	########	########	########	########	########	########	########
########	########	########	########	NA	NA	########	NA	NA
NA	NA	NA	########	NA	NA	NA	########	NA
NA	NA	NA	########	NA	NA	NA	########	NA
########	########	########	########	########	########	########	########	########
NA	NA	NA	########	NA	NA	NA	########	NA
NA	NA	NA	NA	NA	########	NA	########	NA
NA	NA	NA	########	NA	NA	NA	########	NA
NA	NA	NA	########	NA	NA	NA	########	NA
NA	NA	NA	NA	NA	NA	NA	NA	NA

For Review Only

NA	NA	NA	NA	NA	NA	NA	NA	NA
NA	NA	NA	########	NA	NA	NA	########	NA
NA	NA	NA	########	NA	NA	NA	########	NA
NA	NA	NA	NA	NA	NA	NA	NA	NA
NA	NA	NA	NA	NA	NA	NA	NA	NA
NA	########	########	########	NA	NA	NA	########	NA
NA	NA	########	NA	NA	NA	########	NA	NA
NA	NA	NA	########	NA	NA	NA	NA	NA
NA	NA	########	########	NA	NA	########	########	NA
NA	NA	########	NA	NA	NA	########	NA	NA
NA	NA	########	########	NA	NA	########	########	NA
NA	NA	NA	########	NA	NA	NA	########	NA
NA	NA	NA	########	NA	NA	NA	########	NA
NA	NA	NA	NA	NA	NA	NA	NA	NA
########	########	########	########	########	########	########	########	########
NA	########	NA	########	NA	########	NA	########	NA
NA	NA	NA	NA	NA	NA	NA	NA	NA
NA	NA	NA	NA	NA	NA	NA	NA	NA
NA	########	########	########	NA	########	########	########	NA
NA	NA	NA	########	NA	NA	NA	########	NA
NA	NA	NA	NA	NA	NA	########	NA	NA
NA	NA	NA	NA	NA	NA	NA	NA	NA
NA	########	########	########	NA	NA	########	########	NA
NA	NA	########	NA	NA	NA	########	NA	NA
NA	NA	NA	########	NA	NA	NA	########	NA
NA	########	NA	NA	NA	########	NA	NA	NA
NA	NA	NA	NA	NA	NA	NA	NA	NA
NA	NA	NA	NA	NA	NA	NA	NA	NA
NA	NA	NA	NA	NA	NA	NA	NA	NA
NA	NA	NA	NA	NA	NA	NA	NA	NA
NA	NA	NA	NA	NA	NA	NA	NA	NA
NA	NA	NA	NA	NA	NA	NA	NA	NA
########	########	########	NA	########	########	########	NA	########
NA	NA	NA	########	NA	NA	NA	########	NA
########	########	NA	NA	NA	########	NA	NA	NA
NA	NA	NA	NA	NA	NA	NA	NA	NA
NA	NA	NA	########	NA	NA	NA	########	NA
NA	NA	########	NA	NA	NA	########	NA	NA
NA	NA	NA	########	NA	NA	NA	NA	NA
NA	NA	NA	NA	NA	NA	NA	NA	NA
NA	NA	NA	NA	NA	NA	NA	NA	NA
NA	NA	########	########	NA	NA	########	########	NA
NA	NA	NA	NA	NA	NA	NA	NA	NA
NA	NA	NA	NA	NA	NA	NA	NA	NA
NA	NA	########	NA	NA	NA	########	NA	NA
NA	NA	NA	NA	NA	NA	NA	########	NA
########	########	########	########	########	########	########	########	NA
NA	NA	########	########	NA	NA	NA	########	NA
NA	########	########	########	NA	########	########	########	########
NA	########	########	########	NA	########	########	########	NA
NA	NA	########	########	NA	NA	########	########	NA
NA	NA	NA	########	NA	NA	NA	########	NA
NA	NA	NA	########	NA	NA	NA	NA	########
NA	NA	########	########	NA	NA	########	########	NA
NA	NA	########	########	NA	NA	########	########	NA
NA	NA	########	########	NA	NA	########	########	NA
########	########	########	########	########	########	########	########	NA

For Review Only

########	########	########	########	########	########	########	########	NA
########	########	########	########	NA	NA	NA	NA	NA
########	########	########	NA	########	########	########	NA	########
NA	NA	########	########	NA	NA	########	########	NA
NA	NA	########	NA	NA	NA	NA	NA	NA
NA	NA	NA	########	NA	NA	NA	########	NA
NA	########	########	########	NA	########	########	########	########
NA	NA	########	NA	NA	NA	########	########	NA
########	########	########	########	########	########	########	########	########
NA	########	########	########	NA	########	########	########	NA
########	########	########	########	NA	########	########	########	NA
NA	NA	########	########	NA	NA	########	########	NA
NA	########	########	NA	NA	########	########	NA	NA
NA	NA	########	NA	NA	NA	########	NA	NA
NA	########	########	########	########	########	########	########	########
NA	NA	########	NA	NA	NA	########	NA	NA
NA	NA	NA	NA	NA	NA	NA	NA	NA
NA	########	########	NA	NA	########	########	NA	NA
NA	NA	NA	########	NA	NA	NA	########	NA
########	########	########	########	########	########	########	########	########
NA	########	########	########	########	########	########	########	########
NA	NA	NA	########	NA	NA	NA	########	NA
NA	########	########	NA	NA	NA	########	NA	NA
NA	NA	NA	########	NA	NA	NA	########	########
NA	NA	NA	NA	NA	NA	NA	NA	NA
NA	########	########	########	NA	########	########	########	NA
########	########	NA	########	NA	NA	NA	########	NA
NA	NA	NA	NA	########	########	########	########	NA
NA	NA	NA	########	NA	NA	NA	########	NA
NA	NA	NA	NA	NA	NA	NA	NA	NA
########	NA	NA	NA	########	NA	NA	NA	########
NA	NA	NA	NA	NA	NA	NA	NA	NA
NA	NA	NA	NA	NA	NA	NA	NA	NA
NA	NA	NA	NA	NA	NA	NA	NA	NA
NA	NA	########	########	NA	NA	########	########	NA
NA	NA	########	########	NA	NA	########	########	NA
NA	NA	########	########	NA	NA	NA	########	NA
NA	NA	NA	########	NA	NA	NA	########	NA
NA	NA	NA	########	NA	NA	NA	########	NA
NA	NA	NA	########	NA	NA	NA	########	NA
NA	########	########	########	########	########	########	########	########
########	########	NA	NA	########	########	NA	NA	NA
NA	NA	NA	########	NA	NA	NA	########	NA
NA	NA	NA	NA	NA	NA	NA	NA	NA
NA	NA	NA	########	NA	NA	NA	########	NA
NA	NA	NA	########	NA	NA	NA	########	NA
NA	NA	NA	NA	NA	NA	NA	NA	NA
########	########	########	########	NA	########	########	########	########
NA	NA	NA	NA	NA	NA	NA	NA	NA
########	########	########	########	########	########	########	########	NA
NA	########	########	########	########	########	########	########	NA
########	########	########	########	########	########	########	########	NA
########	NA	NA	NA	########	NA	NA	NA	NA
NA	NA	NA	########	NA	NA	NA	########	NA
NA	NA	NA	NA	NA	NA	NA	NA	NA
NA	NA	########	########	NA	NA	########	########	NA

For Review Only

NA	NA	NA	########	NA	NA	NA	########	NA
########	########	########	########	########	########	########	########	NA
NA	NA	########	NA	NA	########	########	NA	NA
NA	########	NA	NA	NA	########	NA	NA	NA
NA	NA	NA	########	NA	NA	NA	########	NA
NA	NA	NA	########	NA	NA	NA	########	NA
NA	NA	NA	NA	NA	NA	NA	########	NA
NA	NA	NA	NA	NA	NA	NA	NA	NA
NA	########	########	########	NA	########	########	########	NA
NA	NA	NA	NA	NA	NA	NA	NA	NA
NA	NA	NA	NA	NA	NA	NA	NA	NA
NA	NA	########	NA	NA	NA	########	NA	NA
NA	NA	NA	NA	NA	NA	NA	########	NA
NA	NA	########	NA	NA	NA	########	NA	NA
NA	NA	NA	NA	NA	NA	NA	NA	NA
NA	NA	########	########	NA	NA	########	########	########
NA	NA	NA	########	NA	NA	NA	########	NA
NA	NA	########	########	NA	NA	NA	########	NA
NA	NA	NA	NA	NA	NA	NA	NA	NA
NA	NA	NA	########	NA	NA	NA	########	NA
NA	NA	########	########	NA	NA	NA	########	NA
NA	NA	NA	NA	NA	NA	NA	########	NA
NA	NA	NA	########	NA	NA	NA	########	NA
NA	NA	NA	########	NA	NA	NA	########	NA
NA	NA	NA	########	NA	NA	NA	########	NA
NA	NA	########	########	NA	NA	########	########	NA
########	########	########	########	NA	########	########	########	NA
NA	########	########	########	NA	########	########	########	########
NA	NA	########	########	NA	NA	NA	########	NA
NA	NA	NA	########	NA	NA	NA	########	NA
########	########	NA	########	########	NA	########	########	NA
NA	NA	NA	NA	NA	NA	NA	NA	NA
NA	NA	########	########	NA	NA	NA	########	NA
########	########	########	########	########	########	########	########	########
NA	NA	NA	########	NA	NA	NA	########	NA
NA	########	########	########	########	########	########	########	########
NA	NA	NA	NA	NA	NA	NA	NA	NA
NA	NA	NA	NA	NA	NA	NA	NA	NA
NA	NA	NA	NA	NA	NA	NA	NA	NA
NA	NA	NA	NA	NA	NA	NA	NA	NA
########	########	########	########	########	########	########	########	########
NA	NA	NA	NA	NA	NA	NA	NA	NA
NA	NA	NA	NA	NA	NA	########	NA	NA
NA	########	########	########	NA	########	########	########	########
NA	NA	########	NA	NA	NA	########	NA	NA
NA	NA	NA	########	NA	NA	NA	########	NA
NA	########	########	########	NA	NA	########	########	########
NA	########	########	########	NA	########	########	########	########
NA	########	########	########	NA	########	########	########	NA
NA	NA	NA	NA	NA	NA	NA	NA	NA
NA	NA	NA	NA	NA	NA	NA	NA	NA
NA	NA	NA	########	NA	NA	NA	########	NA
NA	NA	NA	########	NA	NA	NA	########	NA
NA	########	NA	########	NA	NA	NA	########	NA
NA	NA	NA	NA	NA	NA	NA	NA	NA
########	NA	NA	########	########	NA	########	########	########

For Review Only

NA	NA	NA	NA	NA	NA	NA	NA	NA
########	########	########	########	########	########	########	########	NA
NA	NA	NA	########	NA	NA	########	########	NA
NA	########	########	########	########	########	########	########	########
NA	NA	NA	########	NA	NA	NA	########	NA
NA	NA	NA	NA	NA	NA	NA	NA	NA
NA	NA	NA	NA	NA	NA	NA	NA	NA
NA	NA	NA	########	NA	NA	NA	########	NA
NA	NA	NA	########	NA	NA	NA	########	NA
NA	NA	########	########	NA	NA	########	########	NA
########	########	########	########	########	########	########	########	########
########	########	########	########	########	########	########	########	NA
NA	NA	NA	NA	NA	NA	NA	NA	NA
NA	NA	########	########	NA	NA	NA	########	NA
########	########	########	########	########	########	########	########	NA
NA	########	########	NA	NA	NA	NA	NA	NA

For Review Only

6-4	7-4	6-5	7-5	7-6
NA	NA	NA	NA	NA
NA	NA	NA	NA	NA
NA	NA	NA	NA	NA
NA	########	NA	########	########
NA	########	NA	########	########
NA	NA	NA	NA	########
NA	NA	NA	NA	NA
NA	NA	NA	NA	NA
NA	NA	NA	NA	NA
NA	NA	NA	NA	NA
NA	NA	NA	NA	NA
NA	########	NA	########	########
NA	########	NA	########	########
NA	NA	NA	NA	NA
NA	NA	NA	NA	NA
NA	NA	NA	NA	NA
NA	NA	NA	NA	NA
NA	NA	NA	NA	NA
NA	########	NA	########	########
NA	NA	NA	NA	NA
NA	NA	NA	NA	NA
NA	NA	NA	NA	NA
########	NA	########	NA	########
NA	NA	NA	NA	NA
NA	NA	NA	NA	NA
NA	########	NA	NA	NA
NA	NA	NA	NA	NA
NA	NA	NA	NA	NA
NA	########	NA	########	NA
########	########	NA	########	########
########	########	NA	########	NA
NA	########	NA	########	NA
NA	########	NA	########	########
NA	########	NA	########	NA
NA	NA	NA	NA	NA
NA	NA	NA	NA	NA
NA	NA	NA	NA	NA
NA	NA	NA	NA	NA
NA	########	NA	########	########
NA	NA	NA	NA	NA
NA	########	NA	########	########
NA	NA	NA	NA	NA
########	NA	########	NA	########
NA	NA	NA	NA	NA
########	########	########	########	########
NA	NA	NA	NA	NA
NA	########	NA	########	########
NA	NA	NA	NA	NA
NA	########	NA	########	########
NA	NA	NA	NA	NA
NA	NA	NA	NA	NA
NA	NA	NA	NA	NA
########	########	NA	########	NA
NA	NA	NA	NA	NA
NA	NA	NA	NA	NA

For Review Only

########	########	NA	NA	NA
########	########	NA	########	########
NA	########	NA	########	########
NA	########	NA	########	########
########	########	NA	########	########
NA	NA	NA	########	########
NA	########	NA	########	########
########	########	########	########	########
NA	NA	NA	NA	NA
NA	########	########	NA	########
NA	NA	NA	NA	NA
NA	########	NA	########	########
########	########	NA	########	NA
NA	########	NA	########	########
NA	NA	NA	NA	NA
NA	########	NA	########	########
NA	NA	NA	NA	NA
NA	NA	NA	NA	NA
NA	NA	NA	NA	NA
NA	########	NA	NA	NA
########	########	NA	NA	NA
NA	########	NA	########	########
NA	NA	NA	NA	NA
NA	NA	NA	NA	NA
########	########	NA	########	########
########	########	NA	########	########
########	########	########	########	########
NA	NA	NA	NA	NA
########	########	########	########	########
NA	########	NA	########	########
NA	########	NA	########	########
NA	NA	NA	NA	NA
NA	NA	NA	########	NA
########	########	########	########	########
NA	NA	NA	NA	NA
NA	########	NA	########	########
NA	NA	NA	NA	NA
########	########	NA	NA	NA
NA	########	NA	########	NA
NA	NA	NA	NA	NA
NA	########	NA	########	NA
########	NA	########	NA	########
NA	########	NA	########	########
NA	NA	NA	NA	NA
########	########	NA	NA	NA
NA	NA	NA	NA	NA
########	########	########	########	########
########	########	########	########	NA
########	########	NA	########	NA
NA	########	NA	########	########
NA	NA	NA	########	########

For Review Only

########	########	########	NA	########
NA	########	NA	NA	NA
NA	########	NA	NA	NA
########	########	########	########	NA
NA	########	NA	########	########
NA	########	NA	########	########
########	########	########	########	########
########	########	NA	########	NA
NA	NA	NA	########	########
NA	NA	NA	NA	NA
########	########	NA	NA	NA
########	########	NA	NA	NA
NA	NA	NA	NA	NA
NA	########	NA	NA	NA
########	########	NA	########	NA
########	########	NA	NA	NA
NA	########	NA	########	########
NA	NA	NA	NA	NA
NA	NA	NA	NA	NA
########	NA	########	########	########
NA	NA	NA	NA	NA
########	########	########	########	########
NA	########	NA	########	NA
NA	########	NA	########	########
NA	########	NA	########	########
NA	########	NA	########	NA
NA	NA	NA	NA	NA
########	NA	NA	NA	########
NA	########	NA	########	NA
NA	########	NA	########	########
NA	NA	NA	NA	NA
########	NA	########	NA	########
NA	########	NA	########	########
########	########	NA	NA	NA
NA	NA	NA	NA	NA
NA	########	NA	########	########
NA	NA	NA	NA	NA
NA	NA	NA	NA	NA
NA	NA	########	########	NA
NA	########	NA	########	########
NA	########	NA	NA	NA
NA	########	NA	########	########
########	########	NA	########	NA
NA	NA	NA	NA	NA
NA	NA	NA	NA	NA
########	########	NA	########	########
########	########	NA	NA	NA
########	NA	########	NA	########
########	NA	########	NA	########
########	########	NA	########	########
NA	NA	NA	NA	NA
NA	NA	NA	NA	NA
########	########	NA	########	########
NA	########	NA	########	########
NA	NA	NA	NA	NA
NA	NA	NA	NA	NA

For Review Only

NA	########	NA	########	########
NA	NA	NA	NA	NA
NA	NA	NA	NA	NA
NA	########	NA	########	########
NA	########	NA	########	########
NA	NA	NA	########	NA
########	########	NA	NA	NA
########	########	NA	########	NA
NA	########	NA	########	########
NA	########	NA	########	########
########	########	NA	NA	NA
########	########	NA	########	NA
########	########	NA	########	########
########	########	NA	########	########
NA	NA	NA	NA	NA
NA	########	NA	########	########
########	NA	########	NA	########
NA	########	NA	########	########
########	########	########	########	NA
NA	########	NA	NA	NA
NA	########	NA	########	########
NA	########	NA	########	########
NA	########	NA	########	########
NA	########	NA	########	########
NA	NA	NA	########	########
NA	NA	NA	NA	NA
NA	NA	NA	NA	NA
NA	########	NA	########	########
########	NA	NA	NA	NA
NA	########	NA	########	########
########	########	NA	NA	NA
########	########	NA	NA	NA
########	########	NA	NA	NA
########	########	NA	NA	NA
NA	########	NA	########	########
NA	NA	NA	NA	NA
########	########	NA	########	########
NA	########	NA	########	########
NA	NA	NA	NA	NA
NA	########	NA	########	########
########	########	NA	NA	NA
NA	NA	NA	NA	NA
########	NA	########	########	########
NA	NA	NA	NA	NA
NA	########	NA	########	########
NA	NA	NA	NA	NA
NA	########	NA	########	########
########	NA	########	NA	########
NA	NA	NA	NA	NA
########	########	NA	NA	NA
NA	########	NA	########	########
NA	NA	NA	NA	NA
NA	########	NA	########	########
########	########	NA	NA	NA
NA	NA	NA	NA	NA

For Review Only

NA	NA	NA	NA	NA
NA	NA	NA	NA	NA
########	########	########	########	NA
NA	########	NA	########	########
NA	########	NA	NA	NA
NA	########	NA	########	########
NA	########	########	########	NA
NA	NA	NA	NA	NA
########	########	NA	NA	NA
########	########	NA	########	NA
NA	NA	NA	NA	NA
NA	NA	NA	NA	NA
NA	########	NA	########	########
NA	NA	NA	NA	########
NA	NA	NA	NA	NA
########	########	########	NA	NA
NA	NA	NA	########	NA
########	########	########	########	########
########	########	NA	########	NA
NA	NA	NA	NA	NA
NA	########	NA	########	########
########	########	NA	########	########
NA	########	NA	NA	NA
NA	NA	NA	NA	########
NA	########	NA	########	########
NA	NA	NA	NA	NA
NA	########	NA	########	NA
########	########	NA	########	NA
NA	NA	NA	NA	NA
NA	########	NA	########	########
NA	########	NA	########	########
NA	NA	NA	NA	NA
NA	########	NA	########	########
########	NA	########	NA	########
########	########	########	########	########
NA	########	NA	########	########
NA	########	NA	########	########
NA	########	NA	########	########
NA	########	NA	########	########
NA	########	NA	########	########
NA	NA	NA	NA	NA
NA	########	NA	NA	NA
NA	########	NA	NA	NA
########	NA	########	########	########
NA	########	NA	########	########
NA	########	NA	NA	NA
NA	########	NA	########	########
NA	########	NA	########	########
NA	NA	NA	NA	NA
########	########	NA	########	########
########	NA	NA	NA	NA
########	########	NA	########	NA
NA	########	NA	########	########
NA	########	NA	NA	NA
NA	NA	NA	NA	NA
NA	NA	NA	NA	NA

For Review Only

NA	########	NA	########	########
########	########	NA	NA	NA
########	########	NA	NA	NA
NA	########	NA	########	########
NA	NA	NA	NA	NA
########	########	########	NA	########
NA	########	NA	########	########
NA	NA	NA	NA	NA
NA	########	NA	NA	NA
########	########	NA	########	NA
########	########	NA	########	########
NA	NA	NA	NA	NA
NA	########	NA	########	########
NA	########	NA	########	########
NA	########	NA	NA	NA
########	########	########	########	NA
########	########	########	########	NA
NA	NA	NA	NA	NA
########	########	########	########	########
########	########	NA	########	NA
NA	NA	NA	NA	NA
########	########	NA	NA	NA
NA	NA	NA	########	########
NA	NA	NA	NA	NA
########	########	NA	########	NA
########	########	NA	NA	NA
NA	########	NA	NA	NA
NA	NA	NA	NA	NA
NA	NA	NA	NA	NA
########	NA	########	NA	########
NA	########	NA	########	NA
NA	########	NA	########	########
NA	NA	NA	NA	NA
NA	########	NA	########	########
NA	NA	NA	NA	NA
NA	########	NA	########	########
NA	########	NA	########	NA
NA	NA	NA	NA	NA
########	########	NA	NA	NA
NA	########	NA	########	########
########	NA	########	NA	########
########	NA	########	NA	########
NA	########	NA	########	########
NA	########	NA	########	NA
NA	########	NA	########	########
NA	########	NA	########	########
NA	NA	NA	NA	NA
########	########	NA	NA	NA
NA	########	NA	########	########
NA	NA	NA	NA	NA
NA	NA	NA	NA	NA
NA	########	NA	########	NA
NA	########	NA	########	########
NA	########	NA	########	########
NA	NA	NA	NA	NA
NA	NA	NA	NA	NA

For Review Only

NA	NA	NA	NA	NA
NA	NA	NA	NA	NA
########	########	NA	########	NA
NA	########	NA	########	########
########	########	NA	NA	NA
########	########	NA	NA	NA
NA	########	NA	########	########
NA	########	NA	########	########
########	NA	NA	########	########
NA	NA	NA	NA	NA
NA	########	NA	########	########
NA	########	NA	########	########
NA	########	NA	########	########
NA	########	NA	########	########
NA	NA	NA	NA	NA
NA	########	NA	########	########
NA	########	NA	########	########
########	########	NA	########	########
NA	NA	NA	NA	NA
NA	NA	NA	NA	NA
NA	########	NA	########	########
########	########	NA	########	NA
NA	NA	NA	NA	NA
NA	########	NA	########	########
########	########	NA	########	########
NA	########	NA	########	NA
NA	NA	NA	NA	NA
NA	########	NA	########	NA
NA	########	NA	########	########
########	########	NA	NA	NA
NA	NA	NA	NA	NA
########	########	NA	########	NA
########	NA	########	########	########
########	########	NA	########	########
NA	########	NA	########	########
NA	########	NA	########	NA
########	########	NA	NA	NA
########	########	NA	########	########
NA	NA	NA	NA	NA
########	########	NA	NA	NA
NA	NA	NA	NA	NA
########	########	NA	NA	NA
NA	########	NA	NA	########
NA	########	NA	########	########
########	########	NA	NA	NA
########	########	NA	NA	NA
########	########	NA	########	NA
########	########	NA	########	NA
NA	NA	NA	NA	NA
NA	########	NA	NA	NA
NA	NA	NA	NA	NA
########	########	NA	NA	NA
########	########	NA	NA	NA
NA	NA	NA	NA	NA
NA	########	NA	########	########
NA	########	NA	########	########

For Review Only

########	########	########	########	NA
NA	NA	NA	NA	NA
NA	########	NA	########	########
NA	NA	NA	NA	NA
########	########	NA	########	########
NA	NA	NA	NA	NA
########	########	NA	NA	NA
NA	NA	NA	NA	NA
NA	########	NA	########	########
NA	NA	NA	NA	########
NA	NA	NA	NA	NA
########	########	########	########	########
NA	NA	NA	NA	NA
NA	########	NA	########	NA
########	########	NA	########	NA
NA	NA	NA	NA	NA
NA	########	NA	########	########
########	########	########	########	########
NA	NA	NA	NA	NA
NA	########	NA	########	########
NA	########	NA	########	########
NA	########	NA	########	########
########	########	NA	NA	NA
NA	NA	NA	########	########
########	########	NA	NA	NA
NA	NA	NA	NA	########
NA	########	NA	########	########
NA	########	NA	########	########
NA	NA	NA	NA	NA
NA	NA	NA	NA	NA
NA	########	NA	########	########
NA	########	NA	########	########
########	########	NA	NA	NA
########	########	NA	NA	NA
########	########	NA	NA	NA
NA	########	NA	########	########
NA	########	NA	########	########
########	########	########	########	########
NA	########	NA	########	########
NA	########	NA	########	########
########	########	NA	NA	NA
########	########	NA	########	NA
NA	########	NA	########	########
NA	########	NA	########	########
NA	NA	NA	NA	NA
NA	########	NA	########	########
NA	########	NA	########	########
########	########	NA	########	########
NA	########	NA	########	NA
NA	########	NA	########	########
NA	NA	NA	NA	NA
NA	########	NA	########	########
NA	########	NA	########	########
NA	########	NA	########	########
NA	NA	NA	NA	NA
NA	########	NA	########	########

For Peer Review Only

NA	########	NA	########	########
NA	########	NA	########	########
NA	########	NA	########	########
NA	########	NA	########	########
NA	########	NA	########	########
NA	########	NA	########	########
NA	########	NA	########	########
NA	########	NA	########	########
NA	########	NA	NA	########
NA	########	NA	########	########
########	########	NA	########	########
NA	NA	NA	NA	NA
NA	NA	NA	NA	NA
NA	########	NA	########	########
NA	########	NA	########	########
NA	########	NA	########	########
NA	########	NA	NA	NA
NA	########	NA	########	########
########	NA	NA	NA	NA
NA	########	NA	########	########
NA	NA	NA	NA	########
NA	########	NA	########	########
NA	NA	NA	NA	NA
NA	########	NA	########	########
NA	########	NA	NA	NA
NA	NA	NA	NA	NA
NA	########	NA	########	########
NA	NA	NA	NA	NA
NA	########	NA	########	########
########	NA	########	NA	########
NA	########	NA	########	########
NA	########	NA	########	########
NA	########	NA	########	########
NA	########	NA	########	########
NA	NA	NA	NA	NA
NA	NA	NA	NA	########
########	NA	NA	NA	########
########	NA	########	NA	########
########	NA	NA	NA	########
NA	NA	NA	NA	NA
NA	########	NA	########	########
NA	########	NA	########	########
NA	########	NA	########	########
NA	########	NA	########	########
NA	########	NA	########	########
NA	########	NA	########	########
########	########	NA	########	########
NA	########	NA	########	########
NA	########	NA	########	########
########	NA	########	NA	########
########	NA	########	NA	########
########	########	NA	NA	NA
########	########	NA	NA	NA
########	########	NA	NA	NA
########	########	NA	NA	NA
NA	NA	NA	NA	NA

For Review Only

########	NA	NA	NA	########
NA	########	NA	########	########
NA	########	NA	########	########
########	########	NA	########	########
NA	########	NA	########	########
NA	NA	NA	NA	NA
NA	########	NA	########	NA
NA	########	########	########	########
NA	########	NA	########	########
########	########	NA	NA	NA
NA	########	NA	########	########
NA	NA	NA	NA	NA
NA	########	NA	NA	NA
NA	########	NA	########	########
########	########	########	########	########
########	########	########	########	NA
NA	########	NA	########	########
NA	########	NA	########	########
NA	NA	NA	########	########
########	########	########	########	########
########	########	########	########	########
NA	########	NA	########	########
NA	########	NA	########	########
########	########	NA	########	########
NA	NA	NA	NA	NA
NA	NA	NA	NA	NA
########	########	########	########	########
NA	########	NA	########	########
########	NA	NA	########	########
NA	NA	NA	NA	NA
NA	########	NA	NA	NA
########	########	NA	########	NA
NA	NA	NA	NA	NA
NA	NA	NA	NA	NA
NA	NA	NA	########	########
NA	NA	NA	########	NA
########	NA	########	NA	########
NA	########	NA	########	########
NA	NA	NA	NA	NA
########	NA	########	########	########
########	NA	########	########	########
########	NA	########	########	########
NA	########	NA	########	########
NA	NA	NA	NA	NA
NA	########	NA	########	########
########	NA	########	########	########
########	########	########	########	########
NA	########	NA	########	########
NA	########	NA	########	########
########	########	########	########	########
NA	########	NA	NA	NA
NA	NA	NA	NA	NA
########	########	NA	NA	NA
NA	########	NA	########	########
NA	########	NA	########	########
NA	NA	NA	NA	NA

For Review Only

NA	########	NA	########	########
NA	########	NA	########	########
########	########	########	NA	NA
NA	########	NA	########	########
NA	########	NA	########	########
NA	NA	NA	NA	NA
########	########	########	########	########
NA	########	NA	########	########
NA	########	NA	########	########
NA	NA	NA	NA	NA
NA	NA	NA	NA	NA
NA	########	NA	########	########
########	########	########	########	NA
########	NA	########	NA	########
NA	########	NA	########	########
NA	########	NA	########	########
########	NA	NA	NA	NA
NA	########	NA	########	########
NA	########	NA	########	########
NA	########	NA	########	########
NA	########	NA	########	########
NA	########	NA	########	########
NA	########	NA	########	########
NA	########	NA	NA	########
########	NA	NA	NA	NA
NA	########	NA	########	########
NA	NA	NA	NA	NA
NA	NA	NA	NA	NA
########	########	NA	########	NA
########	########	NA	NA	NA
########	########	NA	########	########
NA	NA	NA	NA	NA
########	########	NA	NA	NA
NA	NA	NA	NA	NA
########	NA	NA	########	########
########	########	NA	########	########
########	########	########	########	########
NA	########	NA	########	NA
NA	NA	NA	NA	NA
NA	NA	NA	NA	NA
########	########	NA	########	########
NA	NA	NA	NA	NA
NA	NA	NA	NA	NA
NA	########	########	NA	########
NA	NA	NA	NA	NA
NA	NA	NA	NA	NA
########	########	NA	########	NA
NA	NA	NA	NA	NA
########	NA	########	NA	########
########	NA	########	NA	########
NA	########	NA	########	########
########	NA	########	########	########
NA	########	NA	########	########
NA	########	NA	NA	NA
########	NA	########	NA	########
########	NA	########	NA	########

For Review Only

NA	########	NA	########	########
########	########	########	########	NA
NA	########	NA	########	########
NA	########	NA	########	########
NA	########	NA	########	########
NA	########	NA	########	########
NA	########	NA	########	########
NA	########	NA	########	########
NA	########	NA	########	########
NA	########	NA	########	########
########	NA	########	NA	########
########	NA	########	NA	########
########	########	########	NA	NA
NA	########	NA	########	########
NA	NA	NA	NA	NA
NA	########	NA	########	########
########	########	NA	NA	NA
NA	NA	NA	NA	NA
NA	########	NA	########	########
NA	########	NA	########	########
########	########	NA	########	########
NA	NA	NA	NA	NA
NA	NA	NA	NA	NA
NA	NA	NA	########	NA
NA	########	NA	########	########
NA	NA	NA	NA	NA
NA	########	NA	########	########
NA	########	NA	########	########
NA	########	NA	########	########
########	########	NA	NA	NA
NA	########	NA	########	########
NA	########	NA	########	########
NA	########	NA	########	########
########	########	NA	########	########
########	NA	########	NA	########
NA	NA	NA	NA	NA
NA	########	########	NA	########
NA	########	NA	########	########
########	########	NA	NA	NA
########	########	NA	NA	NA
########	NA	NA	NA	########
NA	########	NA	########	########
NA	NA	NA	NA	########
NA	########	NA	########	########
########	NA	NA	########	########
NA	NA	NA	########	########
########	NA	########	NA	########
########	########	########	########	NA
NA	NA	NA	NA	NA
NA	########	NA	NA	NA
NA	########	NA	########	########
########	########	NA	########	########
NA	########	NA	########	########
NA	########	########	NA	########
########	########	NA	NA	NA
########	########	NA	########	########

For Review Only

NA	########	NA	########	########
NA	NA	NA	NA	NA
########	########	########	########	########
########	NA	NA	NA	########
NA	NA	NA	NA	NA
NA	NA	NA	NA	NA
########	########	########	########	########
NA	########	NA	########	########
NA	########	NA	########	########
########	########	NA	NA	NA
########	########	########	########	NA
########	########	########	########	########
NA	########	NA	########	########
NA	########	NA	########	########
NA	NA	NA	NA	NA
########	########	NA	NA	NA
NA	NA	NA	NA	NA
NA	########	NA	########	########
NA	NA	NA	NA	NA
NA	########	NA	########	########
########	########	NA	########	NA
########	########	NA	########	NA
NA	########	NA	NA	NA
NA	NA	NA	NA	NA
########	########	########	########	########
NA	########	NA	########	########
NA	########	NA	########	########
NA	NA	NA	NA	NA
NA	########	NA	########	########
########	########	NA	NA	NA
NA	########	NA	########	########
NA	NA	NA	NA	NA
NA	NA	NA	NA	NA
########	########	NA	NA	NA
NA	########	NA	########	########
NA	NA	NA	NA	########
NA	########	NA	########	NA
NA	########	NA	########	########
########	NA	########	########	########
NA	########	NA	########	########
########	NA	########	NA	########
########	########	NA	########	NA
NA	########	NA	########	########
NA	########	NA	########	########
########	########	########	########	########
NA	########	NA	########	########
NA	########	NA	########	########
NA	########	NA	########	########
NA	########	NA	########	########
########	NA	NA	NA	########
NA	########	NA	NA	########
NA	########	NA	########	########
########	########	NA	########	########
NA	########	NA	########	NA
NA	########	NA	########	########
NA	########	NA	########	########

For Review Only

########	########	NA	########	NA
NA	########	NA	########	########
NA	NA	NA	########	########
########	########	NA	########	NA
########	########	NA	########	########
NA	NA	NA	NA	NA
NA	NA	NA	NA	NA
NA	########	NA	########	########
NA	########	NA	########	########
########	########	########	########	########
NA	########	NA	NA	NA
########	########	NA	NA	NA
NA	NA	NA	NA	NA
NA	NA	NA	NA	NA
NA	########	NA	########	########
########	########	NA	NA	NA
########	########	NA	NA	NA
NA	NA	NA	NA	NA
NA	########	NA	########	########
NA	########	NA	########	########
NA	NA	NA	NA	NA
NA	########	NA	NA	########
########	NA	########	NA	########
NA	NA	NA	NA	NA
NA	########	NA	########	########
NA	########	NA	########	########
########	NA	########	########	########
NA	NA	NA	NA	NA
NA	NA	NA	NA	NA
########	########	NA	NA	NA
NA	NA	NA	NA	########
NA	########	NA	########	########
NA	NA	NA	NA	NA
########	NA	########	NA	########
########	NA	NA	########	########
NA	NA	NA	NA	NA
NA	NA	NA	NA	NA
NA	########	NA	########	########
NA	########	NA	########	########
NA	NA	NA	########	########
########	NA	########	NA	########
########	########	########	########	########
########	########	NA	########	########
NA	NA	NA	NA	NA
NA	########	NA	########	########
NA	########	NA	########	########
NA	########	NA	########	########
NA	NA	NA	NA	NA
NA	NA	NA	NA	NA
NA	########	NA	########	########
NA	########	########	########	########
NA	NA	NA	NA	NA
########	########	########	########	########
NA	NA	NA	NA	NA
NA	NA	NA	NA	NA
########	########	NA	########	########

For Review Only

NA	########	NA	########	########
NA	NA	NA	NA	NA
########	########	NA	########	########
NA	NA	NA	NA	NA
NA	########	NA	########	########
NA	########	NA	########	########
########	########	NA	NA	NA
NA	########	NA	########	########
########	########	########	########	########
########	NA	########	NA	########
########	########	NA	########	########
########	NA	########	########	########
NA	########	NA	########	########
NA	########	NA	########	########
########	########	NA	########	NA
########	NA	########	########	########
NA	########	NA	########	########
########	########	NA	NA	NA
NA	########	NA	########	########
########	########	########	########	NA
NA	########	NA	########	########
NA	########	NA	########	########
########	########	NA	NA	########
########	########	NA	NA	NA
NA	########	########	########	########
NA	########	NA	########	########
NA	NA	NA	########	########
NA	########	NA	########	########
NA	NA	NA	NA	NA
NA	########	NA	########	########
NA	########	NA	########	########
########	NA	########	########	########
########	NA	########	NA	########
NA	########	NA	########	########
NA	########	NA	NA	NA
NA	########	NA	########	########
NA	NA	NA	NA	NA
NA	########	NA	########	########
########	########	########	########	########
NA	NA	NA	NA	########
########	########	NA	NA	NA
NA	NA	NA	NA	NA
NA	NA	NA	NA	NA
NA	########	NA	########	########
NA	########	NA	########	########
NA	NA	NA	NA	NA
NA	NA	NA	NA	NA
########	NA	NA	NA	########
NA	########	NA	########	########
NA	########	NA	########	########
NA	NA	NA	NA	NA
########	########	NA	NA	NA
NA	########	NA	########	########
NA	########	NA	########	########
NA	########	NA	########	########
########	########	########	########	########

For Review Only

NA	########	NA	########	########
########	########	NA	########	NA
NA	NA	NA	NA	########
########	########	NA	########	NA
########	NA	########	########	########
########	NA	NA	NA	NA
NA	########	NA	########	########
NA	NA	NA	NA	NA
NA	NA	NA	NA	NA
NA	NA	NA	NA	NA
NA	########	NA	########	########
NA	########	NA	########	NA
NA	########	NA	########	########
########	########	########	NA	NA
NA	NA	NA	NA	NA
NA	NA	NA	NA	NA
NA	NA	NA	NA	########
NA	########	NA	########	########
NA	########	NA	########	########
NA	NA	NA	NA	NA
########	########	NA	########	NA
NA	NA	NA	NA	NA
NA	NA	NA	NA	########
NA	NA	NA	NA	NA
NA	NA	NA	NA	NA
NA	########	NA	########	########
NA	NA	NA	NA	NA
NA	NA	NA	NA	NA
NA	########	NA	########	########
NA	########	NA	########	########
########	########	NA	########	########
NA	########	NA	########	########
########	NA	########	NA	########
NA	########	NA	########	########
########	########	NA	########	NA
NA	NA	NA	NA	NA
NA	########	NA	########	########
########	########	NA	########	########
NA	########	NA	########	########
NA	########	NA	########	########
########	########	########	########	########
NA	NA	NA	NA	NA
NA	########	NA	########	########
NA	NA	NA	NA	NA
NA	NA	NA	NA	NA
########	########	NA	########	########
NA	########	NA	NA	NA
NA	NA	NA	NA	NA
NA	NA	NA	NA	NA
########	########	NA	NA	NA
NA	NA	NA	NA	NA
NA	########	NA	########	########
NA	NA	########	NA	NA
NA	NA	NA	NA	NA
NA	########	NA	########	########
NA	########	NA	########	########

For Review Only

########	NA	########	NA	########
########	########	NA	########	########
########	########	NA	########	NA
########	########	NA	########	NA
NA	########	NA	########	########
########	########	########	########	########
########	########	########	########	########
########	########	########	########	########
########	########	########	########	########
NA	NA	NA	########	########
########	########	NA	########	########
NA	########	NA	########	########
########	########	########	########	NA
########	########	########	########	NA
########	NA	########	NA	########
NA	NA	NA	NA	NA
NA	########	NA	########	########
NA	NA	NA	NA	NA
NA	########	NA	########	########
NA	########	NA	########	########
NA	NA	NA	NA	NA
########	########	########	########	########
NA	########	NA	########	########
########	########	NA	########	########
########	########	NA	NA	NA
NA	########	NA	NA	NA
NA	NA	NA	NA	########
NA	NA	NA	NA	NA
NA	NA	NA	########	########
NA	########	NA	NA	NA
########	NA	########	########	########
########	NA	NA	NA	########
########	NA	########	NA	########
########	########	########	########	########
NA	NA	NA	NA	NA
########	########	########	########	########
NA	########	NA	NA	NA
########	########	########	########	########
########	########	########	########	########
########	########	########	########	########
########	########	########	########	########
NA	########	NA	########	########
########	########	########	########	NA
NA	NA	NA	NA	NA
########	########	NA	NA	NA
NA	########	NA	########	########
########	NA	NA	NA	########
NA	########	NA	########	########
NA	NA	NA	########	########
NA	NA	NA	NA	NA
########	########	########	########	########
NA	########	NA	########	########
NA	########	NA	########	########
########	NA	NA	NA	NA
NA	NA	NA	NA	NA
NA	########	NA	NA	NA

For Review Only

NA	########	NA	########	NA
########	########	NA	########	########
NA	########	NA	########	########
########	########	########	########	NA
NA	########	NA	########	########
NA	NA	NA	NA	NA
NA	########	NA	########	########
NA	########	NA	########	########
########	########	NA	########	########
########	########	NA	NA	NA
########	NA	########	########	########
########	########	NA	########	########
NA	########	NA	########	########
NA	########	NA	########	########
NA	########	########	########	########
NA	NA	NA	NA	NA
NA	NA	NA	NA	NA
########	NA	########	NA	########
NA	NA	NA	########	########
########	NA	NA	NA	########
NA	########	########	########	########
NA	########	NA	########	########
NA	########	NA	########	########
NA	########	NA	NA	NA
NA	########	NA	########	########
########	NA	########	NA	########
NA	########	NA	########	########
NA	########	NA	########	########
NA	NA	NA	NA	NA
NA	NA	NA	########	NA
########	########	NA	NA	NA
NA	NA	NA	NA	NA
NA	########	NA	########	########
NA	########	NA	########	########
NA	########	NA	########	########
NA	########	NA	########	NA
NA	########	NA	########	########
########	########	NA	NA	NA
NA	NA	NA	NA	NA
NA	########	NA	########	########
NA	########	NA	########	########
NA	########	NA	########	########
NA	########	NA	########	########
NA	NA	NA	NA	NA
########	########	NA	########	NA
NA	NA	NA	NA	NA
NA	########	NA	########	########
########	NA	########	########	########
NA	########	NA	NA	NA
NA	NA	NA	NA	NA
NA	NA	NA	NA	########
NA	NA	NA	NA	NA

For Review Only

NA	NA	NA	NA	NA
NA	########	NA	########	########
NA	########	NA	########	########
NA	########	NA	########	########
NA	########	NA	########	########
NA	NA	NA	NA	NA
########	########	########	########	NA
########	########	NA	########	NA
NA	########	NA	########	########
########	NA	NA	NA	NA
NA	NA	NA	NA	NA
########	NA	########	########	########
NA	########	NA	########	########
NA	NA	NA	NA	NA
NA	########	NA	########	########
########	########	NA	########	########
########	########	NA	########	########
NA	########	NA	########	########
########	########	NA	########	NA
NA	NA	NA	NA	NA
########	NA	NA	########	########
########	NA	NA	########	########
########	########	NA	########	########
NA	NA	NA	NA	NA
NA	NA	NA	NA	NA
NA	########	NA	########	########
NA	########	NA	########	########
NA	########	NA	########	########
NA	NA	NA	NA	NA
########	NA	########	########	########
NA	NA	NA	NA	NA
NA	NA	NA	NA	NA
########	########	NA	########	NA
########	########	NA	NA	NA
########	NA	NA	########	########
########	NA	########	NA	########
########	########	NA	NA	NA
NA	########	NA	########	########
NA	NA	NA	########	########
########	########	NA	########	########
NA	NA	NA	NA	NA
NA	########	NA	NA	NA
NA	########	NA	########	########
########	########	NA	########	########
########	NA	########	NA	########
NA	NA	NA	NA	NA
NA	########	NA	########	########
NA	########	NA	########	########
NA	########	NA	########	########
NA	########	NA	########	########
NA	NA	NA	NA	NA
NA	NA	NA	NA	NA
NA	NA	NA	NA	########
########	NA	NA	########	########
NA	NA	NA	NA	NA
########	NA	########	########	########

For Review Only

########	NA	########	########	########
NA	NA	NA	NA	NA
########	########	NA	NA	NA
########	########	########	########	NA
NA	########	NA	########	########
NA	########	NA	########	########
NA	########	NA	########	########
NA	NA	NA	NA	NA
########	########	########	########	########
########	NA	########	NA	########
########	########	NA	########	NA
########	########	NA	########	NA
NA	NA	NA	NA	NA
NA	########	NA	########	########
NA	NA	NA	########	########
########	########	NA	########	########
########	NA	########	NA	########
NA	NA	NA	NA	NA
NA	########	NA	########	########
########	########	NA	########	########
NA	NA	NA	NA	NA
NA	########	NA	########	########
NA	########	NA	########	########
########	NA	########	########	########
NA	########	NA	########	NA
NA	NA	NA	NA	########
NA	NA	NA	NA	NA
NA	########	NA	########	########
NA	NA	NA	NA	NA
NA	########	NA	########	########
########	########	NA	NA	NA
NA	NA	NA	NA	NA
NA	NA	NA	NA	NA
########	########	NA	########	########
########	########	########	########	########
NA	NA	NA	NA	NA
NA	########	NA	########	########
########	NA	########	NA	########
########	########	NA	NA	NA
NA	########	NA	########	########
########	NA	########	########	########
NA	########	NA	########	########
########	########	NA	NA	NA
NA	########	NA	########	########
NA	########	NA	########	########
NA	########	NA	########	########
NA	NA	NA	NA	NA
########	NA	########	########	########
NA	########	NA	NA	NA
NA	NA	NA	NA	NA
########	########	NA	########	NA
NA	NA	NA	NA	NA
NA	NA	NA	NA	NA
NA	NA	NA	NA	NA
########	########	########	########	########
########	########	NA	NA	NA

For Review Only

NA	########	NA	########	########
########	########	NA	########	NA
NA	NA	NA	########	########
########	########	########	########	########
########	########	NA	########	NA
NA	NA	NA	NA	NA
NA	########	NA	########	########
########	NA	NA	NA	NA
NA	########	NA	########	########
NA	NA	NA	NA	NA
########	########	########	########	########
########	NA	########	NA	########
########	NA	########	########	########
########	NA	########	NA	########
########	NA	########	NA	########
########	NA	########	NA	########
########	NA	########	NA	########
########	NA	########	NA	########
NA	########	NA	########	########
NA	NA	NA	########	NA
NA	########	NA	########	########
NA	NA	NA	NA	NA
NA	########	NA	########	########
########	########	########	########	########
NA	########	NA	########	########
NA	########	NA	########	########
NA	NA	NA	NA	NA
########	########	NA	########	########
########	NA	########	NA	########
########	########	NA	NA	NA
########	########	NA	########	NA
NA	########	NA	########	########
NA	########	NA	########	########
NA	NA	NA	NA	NA
NA	########	NA	########	########
NA	NA	NA	NA	NA
NA	########	NA	########	########
NA	########	NA	########	########
########	########	########	########	########
NA	NA	########	########	NA
########	########	NA	NA	NA
NA	########	NA	########	########
NA	NA	NA	########	########
NA	########	NA	########	########
########	NA	########	NA	########
NA	########	NA	NA	NA
NA	########	NA	########	NA
NA	NA	NA	NA	NA
########	########	NA	########	########
NA	########	NA	########	########
########	########	NA	NA	NA
NA	NA	NA	NA	NA

For Review Only

NA	########	NA	########	########
NA	########	NA	########	########
########	########	NA	########	########
NA	########	NA	########	########
########	########	NA	########	########
NA	NA	NA	NA	########
########	########	########	########	########
########	########	########	########	########
########	########	########	########	########
NA	NA	NA	NA	NA
NA	########	NA	########	########
NA	########	NA	########	NA
NA	########	NA	########	########
########	########	NA	########	NA
########	########	NA	########	NA
########	########	NA	NA	NA
NA	NA	NA	NA	NA
NA	########	NA	########	NA
NA	########	NA	########	########
NA	########	NA	########	########
NA	########	NA	########	########
NA	########	NA	########	########
########	########	NA	NA	NA
########	########	NA	########	########
########	########	NA	NA	########
NA	NA	NA	NA	NA
NA	NA	NA	NA	NA
NA	########	NA	########	########
NA	NA	NA	NA	NA
########	########	NA	########	########
NA	########	NA	########	########
NA	########	NA	########	########
NA	########	NA	########	NA
NA	########	NA	########	########
########	########	NA	########	########
NA	########	NA	########	########
NA	NA	NA	NA	NA
NA	########	NA	########	########
########	NA	########	########	########
########	NA	########	########	########
########	########	########	########	########
NA	########	NA	########	########
########	NA	########	########	########
########	########	########	########	########
NA	NA	NA	NA	NA
NA	NA	NA	NA	NA
NA	########	NA	########	########
NA	NA	NA	NA	NA
NA	NA	NA	NA	NA
NA	NA	NA	NA	NA
########	########	NA	NA	NA
NA	########	NA	########	NA
NA	########	NA	########	NA
########	########	########	########	########
NA	NA	NA	NA	NA
NA	NA	NA	NA	NA

For Review Only

NA	########	########	########	########
########	########	NA	########	########
########	########	########	########	########
NA	########	NA	########	########
NA	########	NA	########	########
NA	########	NA	########	########
NA	NA	NA	NA	NA
NA	NA	NA	NA	NA
########	########	NA	########	NA
NA	########	NA	########	########
NA	NA	NA	NA	########
NA	NA	NA	NA	NA
########	########	NA	NA	NA
NA	NA	NA	########	NA
NA	########	NA	########	########
NA	NA	NA	NA	NA
NA	########	NA	NA	NA
NA	NA	NA	NA	NA
NA	########	NA	NA	NA
NA	########	NA	########	########
NA	########	NA	########	########
########	NA	########	NA	########
NA	NA	NA	NA	NA
NA	########	NA	########	########
NA	NA	NA	NA	NA
NA	NA	NA	NA	NA
NA	########	NA	########	########
NA	########	NA	########	########
NA	NA	NA	NA	NA
NA	########	NA	########	########
########	########	NA	NA	NA
NA	########	NA	########	########
NA	NA	NA	NA	########
NA	NA	NA	NA	NA
NA	NA	NA	NA	NA
NA	########	NA	NA	########
########	########	NA	NA	NA
NA	NA	NA	NA	NA
########	########	########	########	NA
########	########	########	########	########
NA	########	NA	########	########
########	########	########	########	########
########	########	NA	########	########
NA	########	NA	########	########
NA	NA	NA	NA	NA
NA	########	NA	########	########
########	NA	########	NA	########
NA	########	NA	########	########
NA	NA	NA	NA	NA
NA	########	NA	########	########
NA	NA	NA	NA	NA
NA	NA	NA	NA	NA
########	########	NA	########	NA
NA	NA	NA	NA	NA
NA	########	NA	########	########
########	########	NA	########	########

For Review Only

NA	########	NA	########	########
NA	########	NA	########	########
NA	NA	NA	NA	NA
NA	########	NA	########	NA
NA	NA	NA	NA	NA
NA	########	NA	########	########
NA	NA	NA	NA	NA
NA	NA	NA	NA	NA
NA	########	NA	########	########
NA	########	NA	########	########
NA	########	NA	NA	NA
########	########	########	########	########
########	########	NA	########	########
########	########	NA	########	########
########	########	NA	########	########
NA	NA	NA	########	########
NA	NA	NA	NA	NA
NA	NA	NA	########	########
NA	########	NA	########	NA
NA	########	NA	########	########
NA	NA	NA	NA	NA
NA	########	NA	########	########
########	########	########	########	########
NA	NA	NA	########	########
NA	NA	NA	NA	NA
NA	########	NA	########	########
NA	########	NA	NA	NA
########	NA	NA	########	########
NA	########	NA	########	########
########	NA	########	NA	########
NA	########	NA	########	########
########	########	NA	NA	NA
NA	NA	NA	NA	NA
########	########	NA	########	########
NA	NA	NA	NA	NA
########	########	NA	NA	NA
NA	NA	########	NA	NA
########	########	NA	NA	NA
NA	NA	NA	NA	NA
NA	NA	NA	########	NA
########	########	NA	########	NA
NA	########	NA	########	########
########	NA	NA	########	########
NA	NA	NA	NA	NA
NA	########	NA	########	########
########	########	NA	########	NA
########	########	########	########	########
NA	########	NA	########	########
NA	########	NA	########	########
########	NA	########	NA	########
NA	########	NA	########	########
########	########	NA	########	########
NA	NA	NA	NA	NA
NA	########	NA	########	########
########	NA	########	NA	########
NA	########	NA	########	########

For Review Only

########	########	########	########	########
########	########	########	########	########
########	NA	NA	NA	NA
NA	########	NA	########	########
NA	########	NA	########	NA
NA	########	NA	########	########
NA	NA	NA	NA	NA
NA	########	NA	########	NA
########	########	NA	########	########
NA	NA	NA	NA	NA
NA	NA	NA	NA	NA
NA	########	NA	########	########
NA	########	NA	########	########
NA	########	NA	########	########
NA	########	NA	########	########
NA	########	NA	########	########
NA	########	NA	########	########
NA	########	########	########	NA
NA	NA	########	########	NA
NA	########	NA	NA	########
NA	NA	NA	NA	########
NA	NA	NA	NA	NA
########	NA	########	NA	########
NA	########	NA	########	########
NA	########	NA	########	########
########	NA	########	NA	########
########	NA	########	NA	########
NA	########	NA	########	########
########	NA	########	NA	########
NA	########	NA	########	NA
########	NA	########	########	########
NA	########	NA	########	########
NA	########	NA	########	########
########	NA	########	NA	########
NA	########	NA	########	########
NA	NA	NA	########	########
NA	########	NA	########	########
NA	NA	NA	NA	NA
NA	########	NA	########	########
NA	########	NA	########	########
NA	NA	NA	NA	NA
NA	########	NA	########	########
NA	NA	NA	NA	NA
NA	########	NA	########	########
NA	########	NA	########	########
NA	########	NA	########	########
NA	########	NA	########	########
########	########	########	########	NA
NA	NA	NA	NA	NA
NA	NA	NA	NA	NA
########	NA	########	########	########
########	########	NA	NA	NA
NA	########	NA	NA	NA
NA	########	NA	########	########
########	########	NA	########	########
########	NA	########	NA	########

For Review Only

########	NA	########	NA	########
########	NA	########	########	########
########	NA	NA	########	########
########	NA	########	NA	########
########	NA	########	########	########
########	NA	########	NA	########
########	NA	########	NA	########
########	NA	########	NA	########
########	NA	########	NA	########
########	NA	########	NA	########
########	NA	NA	NA	########
########	NA	########	NA	########
NA	########	NA	########	########
########	NA	########	NA	########
########	NA	########	NA	########
########	NA	########	NA	########
NA	########	NA	########	########
########	########	NA	NA	NA
NA	########	NA	########	########
########	NA	########	########	########
########	NA	NA	NA	########
########	NA	########	########	########
NA	########	NA	########	########
########	NA	########	NA	########
########	NA	########	NA	########
NA	########	NA	########	########
NA	NA	NA	NA	NA
NA	NA	NA	NA	NA
NA	########	NA	########	########
########	NA	########	NA	########
########	########	NA	########	########
NA	########	NA	########	########
########	NA	########	########	########
########	NA	########	NA	########
########	NA	########	NA	########
########	NA	########	NA	########
########	NA	########	NA	########
########	NA	########	NA	########
########	########	NA	########	########
NA	########	NA	########	########
########	NA	########	########	########
########	NA	########	########	########
########	NA	########	NA	########
########	NA	########	NA	########
########	NA	########	########	########
########	NA	########	########	########
NA	########	NA	########	########
NA	########	NA	########	########
NA	NA	NA	NA	NA
NA	########	NA	########	########
NA	NA	NA	NA	NA
########	NA	NA	########	########
########	NA	########	NA	########
########	########	NA	########	########
NA	########	NA	########	########
########	########	NA	########	########

For Review Only

NA	########	NA	########	########
NA	########	NA	########	########
NA	NA	NA	NA	NA
########	NA	########	NA	########
NA	NA	NA	NA	NA
NA	########	NA	########	########
########	NA	########	########	########
NA	########	NA	########	########
NA	########	NA	########	########
########	NA	NA	########	########
########	NA	########	########	########
NA	NA	NA	NA	NA
########	########	########	########	########
NA	########	NA	########	########
NA	########	NA	########	########
NA	NA	NA	NA	NA
########	NA	NA	NA	########
NA	########	NA	########	########
NA	NA	NA	NA	NA
NA	########	NA	########	########
NA	########	NA	########	########
########	########	NA	########	########
NA	########	NA	########	########
NA	########	NA	########	########
########	NA	NA	########	########
NA	########	NA	########	########
NA	NA	NA	NA	NA
NA	########	NA	########	########
########	########	########	########	NA
NA	NA	NA	NA	NA
NA	########	NA	########	########
NA	########	NA	########	########
########	########	NA	########	NA
NA	NA	NA	NA	NA
NA	NA	NA	NA	NA
NA	########	NA	########	########
NA	########	NA	########	########
########	NA	########	NA	########
NA	NA	NA	NA	NA
########	########	########	########	########
NA	########	NA	########	########
NA	NA	NA	########	NA
NA	NA	NA	NA	NA
########	########	########	NA	########
########	########	NA	########	########
########	########	NA	########	########
NA	NA	NA	NA	NA
########	NA	########	########	########
NA	########	NA	########	########
NA	########	NA	########	########
NA	########	NA	########	########
NA	NA	NA	NA	NA
NA	########	NA	########	########
NA	########	NA	########	########
########	NA	########	NA	########
########	NA	########	NA	########

For Review Only

NA	NA	NA	NA	NA
NA	NA	NA	NA	NA
NA	NA	NA	NA	NA
NA	NA	NA	NA	NA
NA	NA	NA	NA	########
########	########	NA	########	NA
NA	NA	NA	NA	NA
NA	########	NA	########	########
########	########	NA	########	NA
########	########	########	########	########
NA	########	NA	########	########
NA	########	NA	NA	########
NA	NA	NA	NA	NA
########	NA	########	NA	########
########	NA	########	########	########
NA	NA	NA	NA	NA
NA	########	NA	########	########
NA	NA	NA	NA	NA
NA	########	NA	########	NA
########	NA	NA	NA	NA
NA	########	NA	########	########
########	NA	########	NA	########
NA	NA	NA	NA	NA
NA	NA	NA	########	NA
NA	########	NA	########	########
########	########	########	########	########
NA	########	NA	########	########
NA	########	NA	########	########
NA	########	NA	########	########
NA	########	NA	########	########
NA	NA	NA	NA	NA
NA	########	NA	########	########
NA	NA	NA	NA	NA
########	NA	NA	########	########
NA	########	########	########	########
########	########	NA	NA	########
NA	NA	NA	########	########
########	NA	NA	########	########
########	########	NA	########	NA
########	NA	########	NA	########
########	########	NA	########	NA
NA	NA	NA	NA	NA
########	########	########	########	NA
########	########	########	########	########
########	NA	########	NA	########
NA	########	NA	########	########
########	########	NA	########	NA
########	########	NA	########	NA
NA	NA	NA	NA	NA
########	NA	########	NA	########
NA	########	NA	########	########
NA	NA	NA	NA	NA
NA	NA	NA	NA	NA
NA	########	NA	########	########
NA	########	NA	########	NA
NA	NA	NA	NA	########

For Review Only

NA	########	NA	NA	########
NA	########	NA	########	########
NA	########	NA	########	########
########	NA	NA	########	########
########	########	NA	NA	NA
########	########	########	########	########
########	########	NA	NA	NA
NA	########	NA	NA	NA
NA	NA	NA	NA	NA
NA	########	NA	NA	########
NA	########	NA	########	########
NA	NA	NA	########	########
NA	########	NA	########	########
NA	NA	NA	NA	NA
NA	########	NA	########	########
NA	########	NA	NA	NA
########	NA	########	NA	########
########	########	NA	NA	NA
########	########	########	########	NA
NA	NA	NA	NA	NA
NA	NA	NA	NA	NA
NA	########	NA	########	NA
NA	########	NA	########	########
########	NA	########	########	########
########	########	NA	########	########
NA	########	NA	NA	NA
NA	########	NA	########	########
NA	NA	NA	NA	NA
NA	########	NA	########	########
NA	########	NA	########	########
NA	NA	NA	NA	########
########	########	NA	########	NA
NA	NA	NA	NA	NA
NA	NA	NA	NA	NA
NA	########	NA	########	########
NA	########	NA	########	########
NA	NA	NA	NA	NA
NA	########	NA	NA	NA
NA	NA	NA	NA	NA
NA	NA	NA	NA	NA
NA	########	########	########	########
NA	NA	NA	NA	NA
NA	########	NA	NA	NA
NA	########	NA	########	########
NA	NA	########	NA	NA
NA	NA	NA	NA	NA
########	########	NA	########	NA
NA	########	NA	########	########
########	########	NA	########	########
NA	########	NA	########	NA
NA	NA	NA	NA	NA
########	########	########	########	########
########	########	########	########	NA
########	########	NA	########	NA
NA	########	NA	########	########
NA	NA	NA	NA	NA

For Review Only

NA	NA	NA	########	########
NA	########	NA	########	########
NA	########	NA	########	########
NA	NA	NA	########	########
########	########	NA	NA	NA
########	########	NA	########	NA
NA	########	NA	########	########
########	########	NA	NA	NA
NA	########	NA	NA	NA
########	########	########	########	########
NA	NA	NA	########	########
NA	NA	NA	NA	NA
NA	NA	NA	NA	NA
NA	NA	NA	NA	NA
########	########	NA	########	########
########	########	NA	NA	NA
NA	NA	NA	NA	NA
NA	NA	NA	NA	NA
NA	########	NA	########	########
########	########	NA	########	NA
NA	########	NA	########	########
NA	NA	NA	NA	NA
NA	########	NA	########	########
NA	########	NA	########	########
########	########	NA	NA	NA
NA	########	NA	########	########
NA	NA	NA	NA	NA
NA	########	NA	########	########
NA	NA	NA	NA	NA
########	NA	########	NA	########
NA	NA	NA	NA	NA
NA	########	NA	########	########
########	########	NA	########	NA
NA	########	NA	########	########
########	NA	########	NA	########
########	########	NA	NA	NA
NA	########	NA	########	########
NA	########	NA	########	########
NA	########	NA	########	########
NA	########	NA	########	########
NA	########	NA	########	########
########	########	NA	########	NA
NA	########	NA	########	########
########	########	########	########	########
NA	########	NA	########	########
########	########	########	########	########
########	########	NA	########	########
NA	########	NA	########	NA
########	########	NA	NA	NA
NA	NA	NA	NA	NA
NA	NA	NA	########	NA
########	########	NA	########	########
########	########	NA	########	NA

For Review Only

########	########	########	########	########
########	########	NA	########	########
NA	NA	NA	NA	NA
NA	########	NA	########	########
NA	NA	NA	NA	NA
NA	########	NA	########	NA
NA	########	NA	########	########
NA	########	NA	########	NA
NA	NA	NA	########	########
NA	########	NA	########	########
NA	########	########	########	########
NA	########	NA	########	########
NA	########	NA	########	########
NA	NA	NA	NA	NA
########	NA	########	########	########
########	########	########	########	NA
########	########	########	########	########
########	NA	NA	NA	########
NA	########	NA	########	########
NA	NA	NA	NA	NA
NA	########	NA	########	########
NA	NA	NA	NA	NA
NA	NA	NA	########	########
NA	########	NA	########	########
NA	########	NA	########	########
NA	NA	NA	NA	NA
########	########	NA	########	########
NA	NA	NA	NA	NA
NA	########	NA	########	########
NA	########	NA	########	NA
########	########	########	########	########
NA	NA	NA	NA	NA
NA	########	NA	########	########
NA	NA	NA	NA	NA
########	########	########	########	NA
NA	########	NA	########	########
########	NA	########	NA	########
########	########	########	########	NA
########	########	NA	NA	NA
NA	NA	NA	########	########
########	########	NA	NA	NA
NA	NA	NA	NA	NA
NA	########	NA	########	NA
NA	NA	NA	NA	NA
########	########	NA	########	NA
########	########	NA	NA	NA
NA	########	NA	########	########
NA	########	NA	########	########
NA	########	NA	########	########
NA	########	NA	########	########
NA	########	NA	########	########
NA	NA	NA	NA	NA
NA	########	NA	########	########
NA	########	NA	########	########
########	########	########	########	NA
NA	NA	NA	########	NA

For Review Only

NA	NA	NA	NA	NA
########	########	########	########	########
NA	########	########	########	########
NA	########	NA	########	########
NA	NA	NA	NA	########
NA	NA	NA	NA	NA
NA	########	NA	########	########
NA	########	NA	NA	########
########	NA	########	NA	########
NA	########	NA	########	########
NA	########	NA	########	########
NA	########	NA	########	########
########	########	NA	########	NA
########	NA	########	NA	########
NA	NA	NA	NA	NA
NA	########	########	########	NA
########	########	########	########	########
NA	NA	NA	NA	NA
NA	NA	NA	NA	NA
########	########	########	########	########
########	NA	########	########	########
NA	NA	NA	########	########
NA	########	NA	########	########
NA	########	NA	########	########
NA	########	NA	########	########
NA	NA	NA	NA	########
NA	########	########	########	########
########	########	NA	NA	NA
########	NA	########	NA	NA
NA	NA	NA	NA	NA
NA	########	NA	########	########
NA	NA	NA	NA	NA
NA	########	NA	########	########
NA	NA	NA	########	########
NA	NA	NA	########	NA
NA	NA	NA	NA	NA
########	########	NA	NA	NA
NA	########	NA	########	########
########	########	########	########	NA
NA	########	NA	########	########
NA	########	NA	########	########
########	########	NA	########	########
########	########	########	########	NA
NA	########	NA	########	########
########	########	########	########	NA
NA	NA	NA	NA	NA
NA	NA	NA	NA	NA
########	########	NA	NA	NA
########	########	NA	NA	NA
NA	NA	NA	########	########
NA	########	NA	########	########
NA	NA	NA	NA	NA
NA	########	NA	########	########
NA	########	NA	########	########
########	########	NA	########	########
NA	NA	NA	NA	NA

For Review Only

NA	NA	NA	NA	NA
NA	NA	NA	NA	NA
NA	########	NA	########	########
NA	########	NA	########	NA
NA	########	NA	########	########
NA	########	NA	########	########
NA	########	NA	########	########
NA	########	NA	########	########
NA	NA	NA	NA	NA
NA	NA	NA	NA	NA
NA	########	NA	########	########
########	########	########	########	NA
NA	########	NA	########	########
########	########	NA	NA	NA
########	########	NA	NA	NA
NA	NA	NA	NA	########
NA	NA	NA	NA	NA
NA	########	NA	########	########
########	########	NA	########	NA
NA	########	NA	########	########
########	########	########	########	########
NA	NA	NA	NA	NA
########	NA	########	NA	########
NA	########	NA	########	########
NA	########	NA	########	########
NA	NA	NA	########	########
NA	########	NA	########	########
NA	########	NA	NA	NA
########	NA	NA	########	########
NA	NA	NA	NA	NA
NA	########	NA	########	NA
NA	########	NA	########	########
NA	########	NA	########	NA
NA	########	NA	########	########
NA	NA	NA	NA	NA
NA	NA	NA	NA	NA
NA	########	NA	########	########
NA	########	NA	########	########
NA	########	NA	########	########
NA	NA	NA	NA	NA
NA	########	NA	########	########
NA	########	NA	########	########
########	########	NA	NA	NA
NA	########	NA	########	########
NA	########	########	########	########
NA	NA	NA	NA	NA
NA	########	NA	########	########
NA	NA	NA	NA	NA
NA	########	NA	########	NA
NA	########	NA	########	########
########	NA	NA	########	########
NA	########	NA	########	########
NA	########	NA	########	########
NA	NA	NA	NA	NA
NA	NA	NA	NA	NA
NA	########	NA	########	########

For Review Only

########	NA	########	NA	########
########	NA	########	NA	########
NA	NA	NA	NA	NA
NA	########	NA	########	########
NA	########	NA	########	########
########	########	########	########	########
########	########	NA	NA	NA
NA	########	NA	########	########
NA	########	NA	########	########
NA	########	NA	########	########
########	########	NA	########	########
NA	########	NA	########	########
NA	NA	NA	NA	NA
NA	NA	NA	NA	NA
########	########	NA	NA	NA
########	NA	NA	########	########
NA	NA	NA	########	########
NA	########	NA	########	########
NA	########	NA	########	NA
########	########	NA	########	########
########	NA	########	NA	########
NA	NA	NA	NA	NA
########	########	NA	NA	NA
NA	########	NA	########	########
########	########	NA	########	NA
########	########	########	########	NA
NA	NA	NA	NA	NA
########	########	NA	########	NA
########	NA	########	NA	########
NA	########	NA	########	########
NA	########	NA	NA	NA
NA	########	NA	########	########
NA	NA	NA	NA	NA
########	NA	########	NA	########
NA	NA	NA	NA	NA
NA	########	NA	########	########
########	########	NA	NA	NA
NA	########	NA	NA	NA
NA	########	NA	########	########
NA	NA	NA	NA	NA
NA	NA	NA	NA	NA
NA	########	NA	########	########
NA	NA	NA	NA	########
NA	NA	NA	NA	NA
NA	########	NA	########	########
NA	########	NA	NA	NA
NA	NA	NA	########	########
########	########	NA	########	NA
NA	NA	NA	NA	########
NA	NA	NA	NA	NA
NA	NA	NA	NA	########
NA	########	NA	########	########
########	########	NA	NA	NA
NA	NA	NA	NA	NA

For Review Only

NA	NA	NA	NA	NA
NA	NA	NA	NA	NA
########	NA	########	NA	########
NA	NA	NA	NA	NA
NA	########	NA	########	########
NA	NA	NA	NA	NA
NA	NA	NA	NA	NA
NA	NA	NA	NA	NA
NA	NA	NA	NA	NA
NA	########	NA	########	########
NA	NA	NA	NA	NA
NA	########	NA	########	########
NA	NA	NA	NA	NA
NA	NA	NA	NA	NA
NA	NA	NA	NA	NA
########	########	NA	########	NA
NA	NA	NA	NA	NA
########	NA	########	NA	########
NA	########	NA	########	NA
########	NA	NA	NA	########
NA	NA	NA	NA	NA
########	########	NA	########	########
NA	########	NA	########	########
NA	NA	NA	NA	NA
NA	########	NA	########	NA
NA	NA	NA	NA	NA
NA	NA	NA	NA	NA
NA	########	NA	########	########
NA	########	NA	########	########
NA	NA	NA	NA	NA
########	########	########	########	########
NA	NA	NA	NA	NA
NA	NA	NA	NA	NA
########	########	NA	########	########
########	########	NA	NA	NA
########	########	NA	NA	NA
########	########	NA	NA	NA
NA	NA	NA	NA	NA
NA	NA	NA	NA	NA
NA	NA	NA	NA	NA
NA	########	NA	NA	NA
NA	NA	NA	NA	NA
########	NA	NA	NA	NA
NA	########	NA	########	########
NA	NA	NA	NA	NA
NA	########	NA	NA	NA
########	########	NA	NA	NA
NA	########	NA	########	########
########	########	NA	########	NA
########	########	########	########	NA
########	########	NA	########	########
########	########	NA	NA	NA
NA	NA	NA	NA	NA
NA	########	NA	########	########
NA	########	NA	########	########
NA	NA	NA	NA	NA

For Review Only

NA	########	NA	########	########
NA	########	NA	########	########
########	########	NA	########	########
NA	NA	NA	NA	NA
NA	########	NA	########	########
NA	########	NA	NA	NA
NA	########	NA	########	########
########	########	NA	NA	NA
NA	########	NA	NA	NA
NA	NA	NA	NA	NA
NA	NA	NA	NA	NA
########	########	NA	NA	NA
########	NA	NA	NA	NA
########	########	NA	NA	NA
NA	NA	NA	NA	NA
########	########	NA	########	########
NA	########	NA	########	########
NA	NA	NA	NA	########
NA	NA	NA	NA	NA
NA	NA	NA	NA	NA
NA	NA	NA	NA	NA
########	NA	########	NA	########
########	########	NA	NA	NA
NA	########	NA	########	########
NA	NA	NA	NA	NA
NA	NA	NA	NA	########
NA	########	NA	########	NA
NA	########	NA	########	########
NA	########	NA	########	NA
NA	NA	NA	NA	NA
########	########	NA	NA	NA
########	########	########	########	########
NA	NA	NA	NA	NA
########	########	NA	########	########
NA	NA	NA	NA	NA
NA	########	NA	########	NA
NA	########	NA	########	########
NA	########	NA	########	NA
NA	########	NA	########	########
NA	########	NA	########	########
NA	NA	NA	NA	NA
########	########	NA	NA	NA
NA	########	NA	########	NA
NA	NA	NA	NA	NA
NA	########	NA	########	########
NA	########	NA	########	NA
########	########	NA	NA	NA
########	NA	NA	NA	NA
NA	NA	NA	########	NA
NA	NA	NA	########	########
NA	NA	NA	NA	NA
NA	########	NA	NA	NA
NA	NA	NA	NA	########
NA	########	NA	########	########
NA	NA	NA	NA	NA
NA	########	NA	########	########

For Review Only

NA	NA	NA	NA	NA
########	NA	########	NA	########
NA	########	NA	########	########
NA	########	NA	########	########
NA	NA	NA	NA	NA
NA	########	########	########	########
NA	########	NA	########	########
NA	########	NA	########	########
NA	NA	NA	NA	NA
NA	NA	NA	NA	NA
NA	########	NA	########	########
NA	########	NA	########	########
NA	NA	########	NA	########
NA	NA	NA	NA	NA
NA	########	NA	########	########
NA	########	NA	########	########
NA	########	NA	########	########
NA	NA	NA	NA	NA
NA	NA	NA	NA	NA
NA	########	NA	########	########
NA	########	NA	NA	NA
NA	########	NA	########	########
NA	NA	NA	NA	NA
########	########	NA	########	########
NA	########	NA	NA	NA
########	########	NA	########	NA
NA	########	NA	NA	NA
NA	########	NA	########	########
########	########	NA	########	########
NA	########	NA	########	########
NA	NA	NA	NA	NA
NA	NA	NA	NA	NA
NA	########	NA	########	########
NA	########	NA	########	NA
NA	NA	NA	########	NA
NA	NA	NA	NA	NA
NA	NA	NA	NA	NA
########	########	NA	########	NA
########	########	NA	########	NA
########	########	NA	NA	NA
########	########	NA	########	########
NA	NA	NA	NA	NA
NA	NA	NA	NA	NA
NA	NA	NA	NA	NA
NA	NA	NA	NA	########
########	########	NA	NA	NA
NA	NA	NA	NA	NA
NA	NA	NA	NA	NA
NA	########	NA	########	########
NA	########	NA	########	########
NA	NA	NA	NA	NA
NA	########	NA	NA	NA
NA	########	NA	NA	NA

For Review Only

NA	NA	NA	NA	NA
NA	NA	NA	NA	NA
NA	NA	NA	NA	NA
NA	########	NA	########	NA
NA	NA	NA	NA	NA
########	########	NA	NA	NA
NA	NA	NA	NA	NA
########	########	NA	NA	NA
NA	NA	NA	########	########
NA	NA	NA	NA	NA
NA	########	NA	########	NA
NA	NA	NA	NA	NA
NA	NA	NA	NA	NA
########	########	NA	########	NA
NA	########	NA	########	########
########	########	NA	NA	NA
NA	NA	NA	NA	NA
NA	########	NA	########	NA
NA	NA	NA	########	########
NA	NA	NA	NA	NA
NA	NA	NA	NA	NA
NA	NA	NA	########	########
NA	NA	NA	NA	NA
NA	########	NA	########	########
########	########	########	########	########
NA	NA	NA	NA	########
NA	########	NA	########	########
########	########	########	########	########
########	########	NA	########	########
NA	########	NA	########	########
NA	NA	NA	NA	NA
########	########	########	########	NA
NA	########	NA	NA	NA
NA	########	NA	########	NA
########	NA	########	########	########
NA	########	NA	########	NA
NA	NA	NA	NA	NA
NA	NA	NA	NA	NA
########	NA	########	NA	########
NA	NA	NA	NA	NA
########	########	########	########	########
NA	########	NA	########	########
NA	NA	NA	NA	NA
########	########	NA	########	NA
########	########	NA	NA	NA
########	########	NA	NA	NA
NA	########	NA	########	########
NA	########	NA	########	########
NA	NA	NA	########	########
NA	NA	NA	NA	NA
########	########	NA	NA	NA
NA	NA	NA	NA	NA
NA	########	NA	########	########
NA	NA	NA	NA	NA
NA	########	NA	########	########
########	########	NA	########	NA

For Review Only

########	########	NA	########	NA
########	########	########	########	########
NA	########	NA	########	########
NA	########	NA	########	########
NA	NA	NA	NA	NA
NA	########	NA	########	########
########	########	NA	NA	NA
NA	NA	NA	NA	NA
NA	NA	NA	NA	NA
NA	########	NA	########	########
NA	NA	NA	NA	NA
NA	NA	NA	NA	NA
########	########	NA	########	########
NA	NA	NA	NA	NA
NA	NA	NA	NA	NA
NA	########	NA	NA	NA
NA	########	NA	########	########
NA	########	NA	########	########
NA	NA	NA	NA	NA
NA	NA	NA	NA	NA
NA	NA	NA	NA	NA
NA	NA	NA	NA	NA
########	########	########	########	########
NA	NA	NA	NA	NA
NA	########	NA	########	########
NA	########	NA	NA	NA
########	NA	NA	NA	NA
NA	NA	NA	NA	########
NA	########	NA	########	NA
NA	########	NA	########	########
NA	NA	NA	NA	NA
########	########	########	########	NA
NA	NA	NA	########	########
NA	########	NA	########	########
NA	########	NA	########	########
NA	########	NA	NA	########
NA	NA	NA	NA	NA
########	########	NA	NA	NA
NA	########	NA	########	NA
NA	########	NA	########	########
########	NA	NA	########	########
NA	########	NA	NA	NA
NA	NA	NA	NA	NA
NA	NA	NA	########	########
NA	NA	NA	NA	NA
NA	########	NA	########	NA
########	########	NA	NA	NA
NA	########	NA	########	########
NA	########	########	########	NA
########	########	########	########	NA
########	########	NA	########	########
########	NA	########	NA	########
########	NA	########	NA	########
NA	########	NA	NA	NA
########	########	NA	########	NA
NA	########	NA	########	########

For Review Only

NA	########	NA	########	########
NA	NA	NA	NA	NA
NA	NA	NA	NA	NA
NA	########	NA	########	########
NA	NA	NA	NA	NA
########	########	NA	NA	NA
########	########	NA	NA	NA
NA	########	NA	########	########
NA	NA	NA	NA	NA
NA	NA	NA	NA	NA
NA	########	NA	########	########
NA	########	NA	########	NA
NA	NA	########	########	NA
NA	NA	NA	NA	NA
NA	########	NA	NA	########
NA	NA	NA	########	########
########	########	NA	NA	NA
########	NA	########	NA	########
########	########	NA	########	NA
NA	NA	NA	NA	NA
NA	########	NA	########	########
NA	NA	NA	NA	NA
NA	NA	NA	NA	NA
NA	NA	NA	NA	NA
########	########	NA	NA	NA
NA	NA	NA	NA	NA
NA	########	NA	########	########
NA	NA	NA	NA	########
NA	NA	NA	NA	NA
NA	NA	NA	NA	NA
########	########	NA	########	########
########	########	########	########	NA
NA	NA	NA	NA	NA
NA	########	NA	########	########
NA	########	NA	########	NA
NA	########	NA	########	########
########	########	########	########	########
########	########	########	########	########
NA	########	NA	########	########
NA	########	NA	NA	NA
NA	########	NA	########	########
########	########	NA	########	NA
NA	NA	NA	NA	NA
NA	########	NA	########	########
NA	########	NA	########	########
NA	########	NA	########	########
NA	########	NA	########	########
NA	NA	########	########	NA
NA	########	NA	########	########
NA	NA	NA	NA	NA
NA	NA	NA	NA	NA
NA	NA	NA	NA	NA
########	NA	########	NA	########
NA	NA	NA	NA	NA
NA	########	NA	NA	NA
NA	NA	NA	NA	NA

For Review Only

########	########	########	########	########
########	########	NA	NA	NA
NA	########	NA	NA	########
########	########	NA	NA	NA
NA	########	NA	NA	NA
NA	########	NA	########	NA
NA	NA	NA	NA	NA
########	########	NA	NA	NA
NA	NA	NA	NA	NA
########	########	########	########	########
NA	NA	NA	NA	NA
########	NA	NA	NA	NA
########	########	NA	NA	NA
########	NA	########	NA	########
NA	NA	NA	NA	NA
########	########	NA	########	########
NA	########	NA	########	NA
########	########	NA	########	########
NA	NA	NA	NA	NA
NA	NA	NA	NA	NA
NA	NA	NA	NA	NA
NA	NA	NA	NA	NA
NA	########	NA	########	########
########	########	NA	NA	NA
NA	########	NA	########	########
NA	########	NA	########	########
########	########	NA	NA	NA
NA	NA	NA	NA	NA
########	NA	########	NA	########
NA	NA	NA	NA	NA
NA	NA	NA	NA	NA
NA	NA	NA	NA	NA
NA	########	NA	########	########
NA	########	NA	NA	NA
########	########	########	########	########
########	########	NA	########	########
NA	NA	NA	NA	NA
########	########	########	########	NA
########	########	NA	NA	NA
NA	########	NA	########	########
NA	########	NA	########	########
########	########	NA	########	########
NA	NA	NA	NA	NA
########	########	########	########	########
########	########	NA	########	########
NA	NA	NA	NA	NA
NA	########	NA	########	########
NA	########	NA	########	########
NA	########	NA	########	########
NA	########	NA	########	########
NA	NA	NA	NA	NA
NA	########	NA	########	########
########	########	NA	########	NA
NA	NA	NA	NA	NA
NA	########	NA	########	NA
########	NA	NA	NA	########

For Review Only

NA	########	NA	########	NA
NA	########	NA	NA	########
NA	NA	NA	NA	########
NA	########	NA	########	########
NA	########	NA	########	########
NA	########	NA	########	NA
NA	########	NA	########	NA
NA	NA	NA	NA	NA
NA	########	NA	########	########
NA	########	NA	########	########
NA	########	NA	########	########
NA	########	NA	########	########
NA	########	NA	########	########
NA	NA	NA	########	NA
NA	########	NA	########	NA
NA	########	NA	########	########
NA	########	NA	########	########
NA	NA	NA	NA	NA
########	########	########	########	########
NA	########	NA	########	########
NA	NA	NA	NA	NA
########	########	NA	########	NA
NA	NA	NA	NA	NA
NA	########	NA	########	########
NA	########	NA	########	########
NA	NA	NA	NA	NA
NA	NA	NA	NA	NA
NA	NA	NA	NA	########
NA	########	NA	########	########
########	########	NA	NA	NA
NA	NA	NA	NA	NA
NA	NA	NA	########	NA
########	########	NA	########	########
########	NA	########	########	########
NA	NA	NA	NA	NA
NA	########	NA	NA	########
NA	NA	NA	NA	NA
########	########	NA	########	NA
NA	########	NA	########	########
########	########	NA	NA	NA
########	########	########	########	NA
########	########	NA	########	NA
########	NA	NA	########	########
NA	########	NA	########	########
NA	########	NA	########	########
########	########	NA	NA	NA
########	########	NA	########	########
NA	NA	NA	NA	NA
NA	NA	NA	NA	NA
########	########	NA	########	########
NA	########	NA	########	########
########	########	########	########	########
########	########	########	########	########
########	########	NA	NA	NA
NA	########	NA	########	########
NA	NA	NA	NA	NA

For Review Only

NA	########	NA	########	########
########	########	########	########	NA
########	NA	########	NA	########
NA	########	NA	########	NA
########	########	########	########	########
########	NA	NA	NA	########
########	########	NA	NA	NA
NA	NA	NA	NA	NA
NA	########	NA	NA	NA
NA	NA	NA	NA	NA
NA	########	NA	########	########
########	NA	########	########	########
NA	########	NA	########	NA
NA	########	NA	########	########
NA	NA	NA	NA	NA
NA	NA	NA	NA	NA
NA	########	NA	NA	NA
NA	NA	NA	NA	NA
NA	NA	NA	########	NA
NA	NA	NA	NA	NA
########	########	NA	NA	NA
NA	NA	NA	NA	NA
########	########	########	########	########
NA	NA	NA	NA	NA
NA	########	NA	########	########
NA	NA	NA	NA	NA
NA	NA	NA	NA	NA
NA	########	NA	########	########
NA	NA	NA	NA	NA
NA	########	NA	########	########
NA	NA	NA	NA	NA
NA	NA	NA	NA	NA
NA	########	NA	########	########
NA	########	NA	NA	NA
NA	########	NA	########	########
NA	########	NA	########	########
NA	NA	NA	NA	NA
NA	########	NA	########	NA
########	########	########	########	NA
########	########	NA	NA	NA
NA	########	NA	########	########
########	NA	########	NA	########
########	########	NA	NA	NA
########	########	NA	########	NA
########	########	NA	########	NA
NA	########	NA	########	########
NA	NA	NA	NA	NA
NA	NA	NA	NA	NA
NA	########	NA	########	########
NA	########	NA	NA	########
########	########	NA	NA	NA
NA	NA	NA	NA	NA
NA	########	NA	########	NA
NA	NA	NA	NA	NA
NA	NA	NA	NA	NA

For Review Only

NA	########	NA	NA	NA
NA	NA	NA	NA	NA
########	NA	########	NA	########
NA	########	NA	########	NA
NA	########	NA	########	########
########	########	NA	########	########
NA	########	NA	########	########
NA	########	NA	NA	NA
NA	NA	NA	NA	NA
NA	NA	NA	NA	NA
########	########	NA	########	NA
########	########	NA	########	NA
NA	NA	NA	NA	NA
NA	NA	NA	NA	NA
NA	########	NA	NA	NA
########	########	NA	########	########
NA	########	NA	########	########
NA	########	NA	########	NA
NA	NA	NA	NA	NA
NA	NA	NA	NA	NA
NA	########	NA	########	########
NA	NA	NA	NA	NA
NA	########	NA	########	########
########	########	NA	NA	NA
NA	########	NA	########	########
########	NA	NA	NA	NA
NA	NA	NA	NA	NA
NA	########	NA	########	########
NA	NA	NA	NA	NA
NA	########	NA	NA	########
NA	NA	NA	NA	NA
NA	NA	NA	NA	NA
########	########	NA	NA	NA
NA	NA	NA	NA	NA
NA	NA	NA	NA	NA
########	########	NA	NA	NA
NA	NA	NA	NA	NA
NA	NA	NA	NA	NA
NA	NA	NA	NA	NA
NA	NA	NA	NA	NA
NA	########	NA	########	########
########	########	########	########	########
NA	NA	NA	NA	NA
########	NA	NA	########	########
########	########	########	########	########
NA	########	NA	########	########
NA	########	NA	NA	NA
########	########	########	########	########
NA	########	NA	########	########
########	########	NA	########	########
NA	########	NA	########	########
NA	NA	NA	NA	NA
NA	########	NA	########	########
########	########	NA	########	NA
########	########	########	########	########
NA	NA	NA	NA	NA

For Review Only

NA	########	NA	########	########
NA	NA	NA	NA	NA
NA	########	NA	########	########
NA	########	NA	########	########
########	NA	########	NA	########
NA	########	NA	########	########
NA	########	NA	NA	NA
NA	NA	NA	NA	NA
########	########	NA	########	NA
NA	NA	NA	NA	########
########	########	NA	NA	NA
NA	########	NA	########	########
NA	NA	NA	NA	NA
NA	NA	NA	NA	NA
########	########	NA	NA	NA
NA	########	NA	########	########
NA	########	NA	########	########
NA	########	NA	########	NA
NA	NA	NA	NA	NA
NA	########	NA	########	########
NA	NA	NA	NA	NA
NA	NA	NA	NA	NA
NA	########	NA	NA	NA
########	########	NA	########	########
NA	########	NA	NA	NA
NA	NA	NA	NA	NA
NA	NA	NA	NA	NA
NA	NA	NA	NA	NA
########	########	########	########	########
NA	########	NA	########	########
NA	NA	NA	NA	NA
NA	NA	NA	NA	NA
NA	NA	NA	NA	NA
NA	NA	NA	NA	NA
NA	########	NA	########	########
NA	NA	NA	NA	NA
NA	########	NA	########	########
########	########	NA	########	########
NA	NA	NA	########	NA
########	########	NA	NA	NA
NA	########	NA	########	NA
NA	########	NA	########	NA
NA	NA	NA	NA	NA
NA	NA	NA	NA	NA
NA	########	NA	########	NA
NA	########	NA	NA	########
NA	########	NA	########	########
NA	NA	NA	NA	NA
NA	########	NA	########	########
NA	########	NA	########	########
NA	########	NA	NA	NA
NA	NA	NA	NA	NA
NA	NA	NA	NA	NA
NA	########	NA	########	########
########	########	########	########	########
NA	NA	NA	NA	########

For Review Only

NA	NA	NA	NA	NA
NA	NA	NA	NA	NA
NA	########	NA	########	########
########	########	########	########	########
NA	NA	NA	NA	NA
########	########	NA	########	NA
NA	########	NA	########	########
NA	NA	NA	NA	NA
########	########	NA	NA	NA
NA	NA	NA	########	########
NA	########	NA	########	########
########	########	NA	########	########
NA	########	NA	########	########
NA	NA	NA	NA	NA
########	########	NA	NA	NA
NA	########	NA	########	########
NA	########	NA	########	NA
NA	NA	NA	NA	NA
NA	NA	NA	NA	NA
NA	NA	NA	NA	NA
NA	########	NA	########	########
NA	########	NA	########	########
NA	########	NA	########	########
NA	NA	NA	NA	NA
########	########	NA	NA	NA
NA	########	NA	########	########
########	########	NA	########	NA
NA	NA	NA	NA	NA
NA	NA	NA	NA	NA
NA	NA	NA	NA	NA
########	NA	NA	NA	########
########	########	NA	########	########
NA	NA	NA	NA	NA
########	NA	########	NA	########
NA	########	NA	########	########
NA	NA	NA	NA	NA
NA	NA	NA	NA	NA
NA	NA	NA	NA	NA
NA	NA	NA	NA	NA
########	########	NA	########	NA
NA	########	NA	########	########
NA	NA	NA	NA	NA
NA	NA	NA	NA	NA
NA	NA	NA	NA	NA
########	########	NA	NA	NA
########	########	NA	########	NA
NA	NA	NA	########	########
NA	NA	NA	NA	NA
NA	########	NA	########	NA
########	########	########	########	NA
NA	NA	NA	NA	NA
NA	NA	NA	NA	NA
NA	NA	NA	NA	NA
NA	NA	NA	NA	NA
NA	########	NA	########	########
NA	########	NA	########	########

For Review Only

########	########	NA	NA	NA
NA	########	NA	NA	NA
########	########	########	########	########
NA	NA	NA	NA	NA
########	########	NA	NA	NA
NA	NA	NA	NA	NA
NA	NA	NA	NA	NA
NA	########	NA	NA	########
NA	########	NA	########	########
########	NA	########	NA	########
########	########	NA	########	########
NA	NA	NA	NA	NA
NA	########	NA	########	########
NA	NA	NA	NA	NA
NA	########	NA	########	########
NA	########	NA	########	########
########	########	########	########	NA
NA	########	NA	########	NA
NA	########	NA	########	########
NA	########	NA	NA	NA
########	########	NA	NA	NA
NA	########	NA	########	########
NA	NA	NA	NA	NA
NA	########	NA	########	########
########	########	NA	NA	NA
NA	########	NA	########	########
########	########	NA	NA	NA
NA	########	NA	########	########
NA	########	NA	########	########
NA	NA	NA	########	NA
NA	########	NA	########	########
NA	########	NA	########	########
NA	########	NA	########	########
NA	########	NA	########	########
NA	NA	NA	NA	NA
NA	NA	NA	NA	NA
########	########	NA	NA	NA
NA	########	NA	NA	NA
NA	########	NA	########	########
########	########	NA	NA	NA
NA	NA	NA	NA	NA
########	########	NA	NA	NA
########	########	########	########	NA
########	########	########	########	NA
NA	########	NA	########	########
NA	NA	NA	NA	NA
NA	########	NA	########	########
NA	########	NA	NA	NA
########	########	########	########	NA
NA	########	NA	########	########
NA	NA	NA	NA	NA
NA	########	NA	########	########
NA	NA	NA	NA	########
########	########	########	########	########
########	########	NA	NA	NA
NA	########	NA	########	########

For Review Only

NA	########	NA	########	########
NA	NA	NA	########	########
NA	########	NA	########	########
########	########	NA	########	NA
NA	########	NA	########	########
NA	NA	NA	NA	NA
NA	NA	NA	NA	NA
NA	NA	NA	NA	NA
########	########	NA	########	NA
########	NA	########	NA	########
NA	########	NA	########	########
NA	########	NA	########	NA
########	########	########	########	########
NA	########	NA	########	########
NA	NA	NA	NA	NA
NA	########	NA	########	########
NA	########	NA	########	########
NA	NA	NA	NA	NA
NA	NA	NA	NA	NA
########	########	NA	NA	NA
NA	########	NA	########	########
NA	########	NA	########	NA
NA	NA	NA	NA	NA
NA	NA	NA	NA	NA
########	########	NA	NA	NA
########	########	NA	########	########
NA	NA	NA	NA	NA
########	########	NA	NA	NA
NA	########	NA	########	########
NA	########	NA	NA	NA
########	########	NA	########	NA
NA	NA	NA	########	NA
NA	NA	NA	NA	NA
NA	NA	NA	NA	NA
NA	NA	NA	########	########
########	########	NA	NA	NA
NA	########	NA	########	########
NA	########	NA	########	########
NA	NA	NA	NA	NA
NA	NA	NA	NA	NA
NA	NA	NA	NA	NA
NA	########	NA	########	########
NA	########	NA	########	########
########	########	NA	NA	NA
NA	NA	NA	NA	NA
NA	########	NA	########	NA
NA	NA	NA	NA	NA
NA	NA	NA	NA	NA
NA	########	NA	NA	NA
NA	NA	NA	NA	NA
NA	NA	NA	NA	NA
NA	########	NA	########	########
NA	NA	NA	NA	NA
NA	########	NA	########	########
########	########	NA	NA	NA
NA	NA	NA	NA	NA

For Review Only

NA	NA	NA	NA	########
NA	########	NA	########	########
NA	########	NA	########	########
NA	########	NA	########	########
NA	NA	NA	NA	NA
NA	NA	NA	NA	NA
NA	NA	NA	NA	NA
########	########	########	########	########
########	########	NA	NA	NA
NA	########	NA	########	########
NA	NA	NA	NA	NA
NA	########	NA	########	########
########	########	NA	NA	NA
NA	NA	NA	NA	NA
NA	NA	NA	NA	NA
NA	NA	NA	########	########
NA	########	NA	########	########
########	NA	NA	NA	NA
NA	########	NA	NA	NA
NA	NA	NA	NA	NA
########	NA	NA	NA	########
NA	NA	NA	NA	########
NA	########	NA	########	########
########	########	NA	########	########
NA	NA	NA	NA	NA
########	########	########	########	########
NA	########	NA	########	########
NA	########	NA	########	########
NA	NA	NA	NA	NA
NA	########	NA	########	########
NA	########	NA	########	########
NA	########	NA	########	NA
NA	########	NA	########	########
NA	########	NA	########	########
NA	########	NA	NA	########
########	NA	NA	########	########
NA	NA	NA	NA	NA
NA	NA	NA	NA	NA
NA	NA	NA	NA	NA
########	########	NA	NA	NA
NA	NA	NA	NA	NA
NA	NA	NA	NA	NA
NA	NA	NA	NA	NA
NA	NA	NA	NA	NA
NA	########	NA	########	########
########	########	NA	########	NA
NA	NA	NA	NA	NA
########	########	########	########	NA
NA	NA	NA	NA	NA
########	NA	NA	NA	########
NA	NA	NA	NA	########
########	########	NA	NA	NA
NA	NA	NA	########	########
########	NA	########	########	########
########	########	NA	NA	NA
NA	NA	NA	NA	NA

For Review Only

########	########	NA	NA	NA
NA	########	NA	NA	NA
NA	NA	NA	NA	NA
NA	########	NA	########	########
########	########	NA	########	########
NA	NA	NA	NA	########
########	NA	########	NA	NA
########	NA	NA	########	########
########	########	NA	########	########
NA	NA	NA	NA	NA
########	NA	########	NA	########
NA	########	NA	########	########
########	########	NA	NA	NA
NA	########	NA	########	########
NA	NA	NA	########	NA
########	########	NA	########	########
NA	########	NA	########	########
NA	########	NA	########	########
########	########	NA	########	NA
########	########	NA	NA	NA
########	########	NA	NA	NA
NA	########	NA	########	########
NA	########	NA	########	########
########	########	NA	NA	NA
NA	NA	NA	NA	NA
NA	########	NA	########	########
NA	########	NA	########	########
NA	NA	NA	NA	NA
########	########	NA	########	NA
########	########	NA	NA	NA
NA	NA	NA	NA	NA
NA	########	NA	########	########
NA	########	NA	NA	########
NA	NA	NA	NA	NA
NA	########	NA	########	########
NA	NA	NA	NA	NA
########	########	########	########	########
NA	NA	NA	NA	NA
########	########	NA	########	########
########	########	########	########	########
########	########	########	########	NA
NA	########	NA	NA	NA
########	########	NA	########	NA
########	NA	NA	NA	NA
NA	NA	NA	NA	NA
NA	NA	NA	NA	NA
NA	NA	NA	NA	NA
NA	########	NA	NA	########
NA	NA	NA	NA	########
NA	NA	NA	NA	NA
NA	########	NA	########	########
########	########	NA	########	NA
NA	########	NA	########	########
NA	########	NA	########	########
NA	NA	NA	NA	NA
########	########	########	########	########

For Review Only

NA	NA	NA	NA	########
########	########	########	########	NA
NA	NA	NA	NA	NA
NA	########	NA	########	NA
NA	NA	NA	NA	NA
NA	########	NA	########	NA
NA	NA	NA	NA	NA
########	########	NA	########	########
########	########	NA	NA	NA
NA	NA	NA	NA	NA
########	########	NA	########	########
NA	########	NA	########	########
NA	NA	NA	NA	NA
NA	########	NA	########	NA
########	########	NA	NA	NA
########	########	NA	NA	NA
NA	NA	NA	NA	NA
########	########	NA	NA	NA
NA	########	NA	########	########
NA	########	NA	########	########
NA	NA	NA	NA	NA
NA	########	NA	NA	NA
########	########	########	########	NA
########	########	NA	########	########
NA	########	NA	########	########
########	NA	########	NA	########
########	########	NA	NA	NA
NA	########	NA	########	########
NA	NA	NA	NA	NA
########	########	########	########	########
########	########	NA	NA	NA
NA	########	NA	########	########
NA	########	NA	########	########
NA	NA	NA	########	NA
NA	########	NA	########	########
NA	########	NA	########	########
NA	NA	NA	NA	NA
NA	NA	NA	NA	NA
########	########	########	########	NA
########	########	NA	########	########
########	########	NA	########	NA
NA	########	NA	########	NA
NA	########	NA	########	########
NA	########	NA	########	########
NA	NA	NA	NA	NA
NA	########	NA	########	########
NA	########	NA	########	########
NA	NA	NA	NA	NA
########	########	########	########	NA
NA	########	NA	########	########
NA	NA	NA	NA	NA
NA	########	NA	########	########
NA	########	NA	########	########
########	########	NA	NA	NA
NA	########	NA	########	NA
NA	########	NA	########	NA

For Review Only

########	########	NA	########	########
NA	NA	NA	NA	NA
NA	NA	NA	NA	NA
NA	########	NA	NA	NA
NA	NA	NA	NA	NA
NA	NA	NA	########	########
NA	NA	NA	NA	NA
########	NA	NA	NA	########
########	########	NA	########	########
NA	########	NA	########	########
NA	NA	NA	NA	NA
NA	########	NA	########	########
NA	########	NA	########	NA
NA	########	NA	########	########
NA	########	NA	########	########
########	########	NA	NA	NA
########	########	########	########	########
NA	########	NA	########	NA
########	########	########	########	########
NA	NA	NA	NA	NA
NA	########	NA	########	########
NA	NA	NA	NA	########
########	########	NA	########	########
NA	########	NA	########	########
NA	NA	NA	NA	NA
NA	########	NA	########	########
########	########	NA	NA	NA
NA	NA	NA	NA	########
NA	########	NA	########	########
NA	########	NA	########	########
########	########	NA	NA	NA
NA	NA	NA	NA	NA
NA	########	NA	########	########
########	########	NA	########	########
NA	########	NA	########	########
NA	NA	NA	NA	NA
NA	########	NA	########	########
NA	NA	NA	NA	NA
NA	########	NA	########	NA
########	########	NA	########	NA
########	########	NA	NA	NA
NA	########	NA	########	########
NA	NA	NA	NA	NA
NA	NA	NA	NA	NA
NA	NA	NA	NA	NA
########	########	NA	NA	NA
NA	NA	NA	NA	NA
NA	########	NA	########	########
NA	########	NA	########	########
########	########	NA	########	NA
NA	########	NA	########	########
NA	NA	NA	NA	NA
NA	########	NA	########	########
NA	########	NA	########	########
NA	NA	NA	NA	NA

For Review Only

NA	NA	NA	NA	NA
NA	########	NA	########	########
########	########	NA	########	########
NA	NA	NA	NA	NA
NA	NA	NA	NA	NA
NA	########	NA	NA	NA
NA	NA	NA	NA	NA
NA	########	NA	########	NA
NA	########	NA	########	########
NA	NA	NA	########	########
NA	########	########	########	########
NA	NA	NA	########	NA
NA	########	NA	########	NA
NA	NA	NA	NA	NA
########	########	NA	NA	NA
NA	NA	NA	NA	########
NA	########	NA	NA	NA
NA	NA	NA	NA	NA
NA	########	NA	########	########
NA	NA	NA	NA	########
########	NA	NA	NA	########
NA	NA	NA	NA	NA
########	########	NA	########	NA
########	NA	########	NA	NA
NA	########	NA	########	########
NA	########	NA	########	NA
NA	NA	NA	NA	NA
NA	NA	NA	NA	NA
NA	NA	NA	NA	NA
NA	NA	NA	########	########
NA	NA	NA	NA	NA
NA	NA	NA	NA	NA
NA	########	NA	########	########
NA	########	NA	NA	NA
NA	########	NA	########	########
NA	NA	NA	NA	NA
NA	########	NA	########	########
########	NA	########	NA	########
NA	NA	NA	########	########
NA	NA	NA	NA	NA
NA	########	########	########	########
NA	NA	NA	NA	NA
NA	NA	NA	NA	NA
NA	########	NA	########	########
NA	########	NA	########	########
NA	NA	NA	NA	NA
########	########	NA	########	########
########	########	NA	NA	NA
########	########	NA	########	NA
NA	########	NA	NA	NA
NA	########	NA	########	########
########	########	NA	NA	NA
########	########	########	########	NA
########	########	NA	NA	NA
########	########	########	########	NA
NA	NA	NA	NA	NA

For Review Only

NA	NA	NA	NA	NA
NA	NA	NA	NA	NA
NA	########	NA	########	########
########	########	NA	########	########
NA	NA	NA	NA	########
NA	########	NA	########	########
########	########	########	########	########
########	NA	########	########	########
########	########	NA	NA	NA
########	########	NA	########	########
NA	########	NA	NA	NA
########	########	########	########	NA
########	NA	NA	########	########
NA	########	NA	########	########
########	########	########	########	NA
NA	NA	########	NA	########
NA	NA	NA	NA	NA
########	########	########	########	########
NA	########	NA	########	########
########	########	NA	NA	NA
########	########	NA	########	NA
NA	########	NA	########	########
########	########	########	########	########
########	########	NA	########	########
NA	NA	NA	NA	NA
########	########	########	########	########
########	########	########	########	########
NA	NA	NA	NA	NA
NA	########	NA	NA	NA
NA	NA	NA	NA	NA
########	########	NA	NA	NA
NA	NA	NA	NA	NA
NA	NA	NA	NA	NA
NA	NA	NA	NA	NA
########	########	NA	########	NA
NA	NA	NA	NA	NA
NA	########	NA	########	########
NA	########	NA	########	########
NA	########	NA	########	########
NA	########	NA	########	########
########	########	########	########	NA
NA	########	NA	########	NA
NA	########	NA	########	########
NA	NA	NA	NA	NA
NA	########	NA	########	########
NA	########	NA	########	########
NA	NA	NA	NA	NA
########	########	########	########	########
NA	NA	NA	NA	########
NA	NA	NA	NA	NA
NA	NA	NA	NA	NA
NA	########	NA	NA	NA
NA	########	NA	########	########
NA	########	NA	########	########
NA	NA	NA	NA	NA
NA	########	NA	########	########

For Review Only

NA	########	NA	########	########
NA	########	NA	########	########
########	NA	########	########	########
NA	NA	########	########	NA
NA	########	NA	########	########
NA	########	NA	NA	NA
########	########	NA	NA	NA
NA	NA	NA	NA	NA
NA	########	NA	########	########
NA	NA	NA	NA	NA
NA	NA	NA	NA	NA
########	NA	NA	########	########
NA	########	NA	NA	NA
########	NA	########	NA	########
NA	NA	NA	NA	NA
########	########	NA	########	NA
NA	########	NA	########	########
########	########	NA	########	NA
NA	NA	NA	########	########
NA	########	NA	########	NA
########	########	NA	########	NA
NA	########	NA	########	NA
NA	########	NA	########	########
NA	########	NA	########	########
NA	########	NA	########	########
########	########	NA	########	NA
########	########	########	########	NA
########	########	NA	########	NA
NA	########	NA	########	########
NA	########	NA	########	NA
########	########	########	########	########
NA	NA	NA	NA	NA
NA	NA	NA	NA	NA
########	########	NA	NA	NA
NA	########	NA	########	########
########	########	NA	NA	NA
NA	NA	NA	NA	NA
NA	NA	NA	NA	NA
NA	NA	NA	NA	NA
NA	NA	NA	NA	NA
########	NA	NA	NA	NA
NA	NA	NA	NA	NA
NA	NA	NA	NA	########
########	########	NA	########	NA
NA	NA	NA	########	########
NA	########	NA	########	########
########	########	NA	########	NA
########	########	NA	NA	NA
NA	########	NA	NA	NA
NA	########	NA	NA	########
NA	NA	NA	NA	NA
NA	########	NA	########	########
NA	########	NA	########	########
NA	########	NA	########	########
NA	NA	NA	NA	NA
########	########	########	########	########

For Review Only

NA	NA	NA	NA	NA
########	########	NA	NA	NA
########	########	NA	########	NA
########	########	NA	########	########
NA	########	NA	########	NA
NA	NA	NA	NA	NA
NA	NA	########	########	NA
NA	########	NA	########	########
NA	########	NA	########	########
NA	########	NA	########	NA
########	########	NA	NA	NA
NA	########	########	########	########
NA	NA	NA	NA	NA
NA	NA	NA	NA	NA
NA	NA	NA	NA	NA
NA	NA	NA	########	########

For Review Only

Var1	Location	Confidence
A0FGR8	Endoplasm	High
A2RRP1	Endoplasm	High
A6NDG6	Cytoplasm	High
A6NHX0	Cytoplasm	High
C9J069	Mitochondr	High
O00142	Mitochondr	High
O00154	Cytoskeletc	High
O00161	Endoplasm	High
O00299	Cytoplasm	High
O00303	Presynapse	High
O00410	Cytoskeletc	High
O00429	Cytoskeletc	High
O00442	Endoplasm	High
O00445	Endoplasm	High
O00468	Nucleus	High
O00483	Cytoskeletc	High
O00499	Cytoskeletc	High
O00505	Cytoskeletc	High
O00519	Endoplasm	High
O00533	Cytoskeletc	High
O00560	Presynapse	High
O00584	Cytoplasm	High
O00743	Endoplasm	High
O00764	Cytoskeletc	High
O14521	Mitochondr	High
O14523	Endoplasm	High
O14531	Cytoskeletc	High
O14548	Postsynaps	High
O14576	Cytoskeletc	High
O14578	Postsynaps	High
O14594	Cytoskeletc	High
O14617	Cytoskeletc	High
O14639	Cytoskeletc	High
O14672	Presynapse	High
O14734	Endoplasm	High
O14735	Endoplasm	High
O14737	Cytoplasm	High
O14745	Cytoskeletc	High
O14773	Cytoskeletc	High
O14818	Cytoskeletc	High
O14827	Endoplasm	High
O14874	Mitochondr	High
O14880	Cytoskeletc	High
O14949	Mitochondr	High
O15020	Cytoskeletc	High
O15027	Endoplasm	High
O15061	Nucleus	High
O15067	Cytoplasm	High
O15075	Cytoskeletc	High
O15083	Postsynaps	High
O15126	Cytoskeletc	High
O15228	Endoplasm	High
O15230	Nucleus	High
O15260	Endoplasm	High
O15394	Plasma me	High

For Review Only

O15530	Mitochondr	High
O15540	Cytoplasm	High
O43143	Nucleus	High
O43175	Cytoskeletc	High
O43181	Postsynaps	High
O43236	Cytoskeletc	High
O43237	Cytoskeletc	High
O43301	Cytoskeletc	High
O43324	Endoplasm	High
O43390	Cytoskeletc	High
O43396	Cytoskeletc	High
O43414	Cytoplasm	High
O43426	Cytoskeletc	High
O43432	Endoplasm	High
O43488	Cytoskeletc	High
O43491	Cytoskeletc	High
O43598	Cytoplasm	High
O43615	Postsynaps	High
O43674	Mitochondr	High
O43676	Mitochondr	High
O43707	Cytoskeletc	High
O43761	Presynapse	High
O43768	Cytoplasm	High
O43772	Mitochondr	High
O43776	Cytoskeletc	High
O43813	Cytoskeletc	High
O43837	Cytoskeletc	High
O43852	Endoplasm	High
O43865	Cytoskeletc	High
O43920	Mitochondr	High
O60256	Cytoskeletc	High
O60282	Cytoskeletc	High
O60313	Cytoskeletc	High
O60502	Cytoskeletc	High
O60506	Cytoskeletc	High
O60641	Cytoskeletc	High
O60645	Endoplasm	High
O60664	Cytoskeletc	High
O60741	Endoplasm	High
O60763	Endoplasm	High
O60825	Endoplasm	High
O60831	Endoplasm	High
O60841	Endoplasm	High
O60888	Cytoskeletc	High
O60936	Cytoplasm	High
O75061	Cytoskeletc	High
O75078	Endoplasm	High
O75083	Cytoskeletc	High
O75116	Cytoskeletc	High
O75223	Cytoplasm	High
O75306	Cytoskeletc	High
O75363	Plasma me	High
O75367	Nucleus	High
O75368	Cytoskeletc	High
O75381	Endoplasm	High
O75390	Cytoskeletc	High

For Review Only

O75396	Plasma me	High
O75439	Mitochondr	High
O75489	Cytoskeletc	High
O75534	Endoplasm	High
O75608	Cytoplasm	High
O75643	Nucleus	High
O75663	Cytoplasm	High
O75746	Cytoskeletc	High
O75781	Cytoskeletc	High
O75828	Cytoplasm	High
O75832	Endoplasm	High
O75844	Endoplasm	High
O75879	Mitochondr	High
O75880	Mitochondr	High
O75884	Cytoplasm	High
O75891	Cytoskeletc	High
O75914	Cytoplasm	High
O75915	Plasma me	High
O75947	Cytoskeletc	High
O75955	Plasma me	High
O76024	Endoplasm	High
O76070	Cytoskeletc	High
O94760	Cytoskeletc	High
O94811	Cytoskeletc	High
O94819	Cytoskeletc	High
O94826	Cytoskeletc	High
O94874	Endoplasm	High
O94910	Plasma me	High
O94925	Cytoskeletc	High
O94973	Cytoskeletc	High
O94979	Endoplasm	High
O94985	Cytoplasm	High
O95057	Endoplasm	High
O95169	Presynapse	High
O95197	Cytoskeletc	High
O95202	Postsynaps	High
O95248	Cytoskeletc	High
O95278	Cytoplasm	High
O95292	Cytoskeletc	High
O95299	Cytoskeletc	High
O95336	Cytoskeletc	High
O95352	Cytoplasm	High
O95372	Endoplasm	High
O95373	Cytoskeletc	High
O95394	Cytoplasm	High
O95433	Cytoskeletc	High
O95487	Endoplasm	High
O95571	Mitochondr	High
O95670	Cytoskeletc	High
O95747	Endoplasm	High
O95757	Cytoskeletc	High
O95777	Cytoplasm	High
O95782	Cytoskeletc	High
O95810	Nucleus	High
O95817	Cytoplasm	High
O95822	Cytoplasm	High

For Review Only

O95831	Mitochondr	High
O95861	Cytoplasm	High
O95865	Cytoskeletc	High
O96000	Cytoskeletc	High
O96005	Endoplasm	High
P00338	Cytoskeletc	High
P00352	Cytoskeletc	High
P00367	Cytoskeletc	High
P00387	Cytoskeletc	High
P00390	Cytoskeletc	High
P00395	Cytoskeletc	High
P00403	Cytoskeletc	High
P00441	Cytoskeletc	High
P00450	Cytoplasm	High
P00491	Cytoplasm	High
P00492	Cytoskeletc	High
P00505	Cytoskeletc	High
P00533	Presynapse	High
P00558	Cytoskeletc	High
P00568	Cytoskeletc	High
P00738	Cytoskeletc	High
P00751	Cytoplasm	High
P00813	Cytoplasm	High
P00915	Cytoskeletc	High
P00918	Cytoskeletc	High
P01008	Cytoplasm	High
P01009	Cytoskeletc	High
P01011	Cytoplasm	High
P01019	Cytoplasm	High
P01023	Cytoskeletc	High
P01024	Cytoskeletc	High
P01034	Cytoskeletc	High
P01112	Cytoskeletc	High
P01834	Cytoskeletc	High
P01857	Cytoskeletc	High
P02042	Cytoskeletc	High
P02462	Nucleus	High
P02511	Cytoskeletc	High
P02545	Nucleus	High
P02549	Mitochondr	High
P02647	Cytoskeletc	High
P02649	Cytoskeletc	High
P02652	Cytoplasm	High
P02671	Cytoskeletc	High
P02675	Cytoskeletc	High
P02679	Cytoskeletc	High
P02686	Cytoskeletc	High
P02689	Cytoskeletc	High
P02750	Cytoplasm	High
P02763	Cytoplasm	High
P02765	Cytoplasm	High
P02766	Cytoplasm	High
P02768	Cytoskeletc	High
P02774	Cytoplasm	High
P02786	Endoplasm	High
P02787	Cytoskeletc	High

For Review Only

P02792	Cytoskeletc	High
P02794	Cytoskeletc	High
P03928	Mitochondr	High
P04062	Endoplasm	High
P04075	Cytoskeletc	High
P04080	Cytoskeletc	High
P04083	Cytoskeletc	High
P04156	Plasma me	High
P04179	Cytoskeletc	High
P04181	Mitochondr	High
P04216	Cytoskeletc	High
P04271	Cytoskeletc	High
P04406	Cytoskeletc	High
P04632	Cytoskeletc	High
P04792	Cytoskeletc	High
P04839	Endoplasm	High
P04899	Cytoskeletc	High
P05067	Cytoskeletc	High
P05090	Cytoskeletc	High
P05091	Cytoskeletc	High
P05109	Nucleus	High
P05141	Cytoskeletc	High
P05230	Cytoplasm	High
P05386	Cytoskeletc	High
P05387	Cytoskeletc	High
P05388	Cytoskeletc	High
P05413	Cytoskeletc	High
P05771	Cytoskeletc	High
P05937	Cytoplasm	High
P06396	Cytoskeletc	High
P06576	Cytoskeletc	High
P06702	Cytoskeletc	High
P06703	Cytoskeletc	High
P06733	Cytoskeletc	High
P06744	Cytoskeletc	High
P06748	Cytoskeletc	High
P07099	Cytoskeletc	High
P07108	Cytoskeletc	High
P07195	Cytoskeletc	High
P07196	Cytoskeletc	High
P07197	Cytoskeletc	High
P07203	Cytoskeletc	High
P07237	Cytoskeletc	High
P07311	Cytoplasm	High
P07339	Cytoskeletc	High
P07355	Cytoskeletc	High
P07384	Cytoskeletc	High
P07437	Cytoskeletc	High
P07602	Cytoskeletc	High
P07686	Cytoplasm	High
P07741	Cytoskeletc	High
P07814	Cytoskeletc	High
P07900	Cytoskeletc	High
P07910	Cytoskeletc	High
P07919	Cytoskeletc	High
P07954	Cytoskeletc	High

For Review Only

P08133	Cytoskeletc	High
P08134	Endoplasm	High
P08195	Cytoskeletc	High
P08237	Cytoskeletc	High
P08238	Cytoskeletc	High
P08559	Cytoskeletc	High
P08572	Nucleus	High
P08574	Cytoskeletc	High
P08670	Cytoskeletc	High
P08708	Cytoskeletc	High
P08758	Cytoskeletc	High
P08865	Cytoskeletc	High
P09104	Cytoskeletc	High
P09110	Plasma me	High
P09211	Cytoskeletc	High
P09382	Cytoskeletc	High
P09417	Cytoskeletc	High
P09429	Cytoskeletc	High
P09455	Cytoplasm	High
P09471	Cytoskeletc	High
P09488	Cytoskeletc	High
P09496	Cytoskeletc	High
P09497	Cytoskeletc	High
P09525	Presynapse	High
P09543	Cytoskeletc	High
P09622	Cytoskeletc	High
P09651	Cytoskeletc	High
P09669	Cytoskeletc	High
P09936	Cytoskeletc	High
P09960	Cytoplasm	High
P09972	Cytoskeletc	High
P0C0L5	Cytoskeletc	High
P0DMV9	Cytoskeletc	High
P0DOY3	Cytoskeletc	High
P0DP25	Cytoskeletc	High
P0DPI2	Cytoskeletc	High
P10114	Plasma me	High
P10155	Cytoplasm	High
P10412	Cytoskeletc	High
P10451	Cytoplasm	High
P10515	Cytoskeletc	High
P10599	Cytoskeletc	High
P10636	Cytoskeletc	High
P10645	Cytoskeletc	High
P10768	Cytoskeletc	High
P10809	Cytoskeletc	High
P10909	Cytoskeletc	High
P10915	Cytoskeletc	High
P11021	Cytoskeletc	High
P11047	Nucleus	High
P11137	Cytoskeletc	High
P11142	Cytoskeletc	High
P11177	Cytoskeletc	High
P11182	Mitochondr	High
P11215	Endoplasm	High
P11216	Cytoskeletc	High

For Review Only

P11217	Cytoskeletc	High
P11233	Cytoskeletc	High
P11279	Plasma me	High
P11498	Cytoskeletc	High
P11532	Endoplasm	High
P11766	Cytoskeletc	High
P11940	Cytoskeletc	High
P12036	Cytoskeletc	High
P12074	Cytoskeletc	High
P12235	Cytoskeletc	High
P12236	Cytoskeletc	High
P12277	Cytoskeletc	High
P12429	Cytoplasm	High
P12532	Cytoskeletc	High
P13010	Cytoskeletc	High
P13073	Cytoskeletc	High
P13489	Cytoskeletc	High
P13611	Cytoskeletc	High
P13637	Cytoskeletc	High
P13639	Cytoskeletc	High
P13716	Cytoskeletc	High
P13796	Cytoplasm	High
P13804	Cytoskeletc	High
P13861	Cytoskeletc	High
P13929	Cytoplasm	High
P14136	Cytoskeletc	High
P14174	Cytoskeletc	High
P14314	Endoplasm	High
P14324	Cytoplasm	High
P14406	Cytoskeletc	High
P14415	Cytoskeletc	High
P14543	Nucleus	High
P14550	Cytoskeletc	High
P14618	Cytoskeletc	High
P14625	Cytoskeletc	High
P14649	Endoplasm	High
P14854	Cytoskeletc	High
P14866	Cytoskeletc	High
P14923	Endoplasm	High
P14927	Cytoskeletc	High
P15104	Cytoskeletc	High
P15121	Cytoskeletc	High
P15259	Cytoplasm	High
P15311	Cytoskeletc	High
P15531	Cytoskeletc	High
P15559	Cytoplasm	High
P15880	Cytoskeletc	High
P16144	Endoplasm	High
P16152	Cytoskeletc	High
P16219	Cytoskeletc	High
P16435	Endoplasm	High
P16615	Cytoskeletc	High
P16870	Endoplasm	High
P16930	Cytoplasm	High
P16949	Cytoskeletc	High
P17066	Cytoskeletc	High

For Review Only

P17174	Cytoskeletc	High
P17301	Cytoskeletc	High
P17302	Cytoskeletc	High
P17655	Cytoskeletc	High
P17677	Cytoskeletc	High
P17812	Endoplasm	High
P17858	Cytoskeletc	High
P17987	Cytoskeletc	High
P18077	Plasma me	High
P18124	Cytoskeletc	High
P18206	Cytoskeletc	High
P18621	Cytoskeletc	High
P18669	Cytoskeletc	High
P18859	Cytoskeletc	High
P19086	Cytoskeletc	High
P19174	Cytoplasm	High
P19338	Cytoskeletc	High
P19367	Cytoskeletc	High
P19525	Endoplasm	High
P19623	Cytoplasm	High
P19634	Endoplasm	High
P19652	Cytoplasm	High
P19971	Cytoplasm	High
P20073	Cytoskeletc	High
P20336	Cytoskeletc	High
P20340	Cytoplasm	High
P20618	Cytoskeletc	High
P20645	Endoplasm	High
P20674	Cytoskeletc	High
P20700	Nucleus	High
P20810	Cytoplasm	High
P20916	Cytoskeletc	High
P21266	Cytoskeletc	High
P21281	Cytoskeletc	High
P21283	Cytoskeletc	High
P21291	Cytoskeletc	High
P21333	Cytoskeletc	High
P21359	Endoplasm	High
P21399	Cytoplasm	High
P21796	Cytoskeletc	High
P21926	Cytoskeletc	High
P21953	Postsynaps	High
P21964	Plasma me	High
P21980	Nucleus	High
P22033	Mitochondr	High
P22061	Cytoskeletc	High
P22234	Cytoskeletc	High
P22307	Endoplasm	High
P22314	Cytoskeletc	High
P22392	Cytoskeletc	High
P22570	Cytoskeletc	High
P22626	Cytoskeletc	High
P22694	Cytoskeletc	High
P22695	Cytoskeletc	High
P22732	Endoplasm	High
P22748	Endoplasm	High

For Review Only

P23142	Cytoplasm	High
P23246	Cytoskeletc	High
P23284	Cytoskeletc	High
P23297	Cytoskeletc	High
P23368	Cytoskeletc	High
P23381	Cytoskeletc	High
P23396	Cytoskeletc	High
P23471	Cytoskeletc	High
P23515	Cytoskeletc	High
P23526	Cytoskeletc	High
P23634	Cytoskeletc	High
P23763	Cytoskeletc	High
P24043	Nucleus	High
P24534	Cytoskeletc	High
P24539	Cytoskeletc	High
P24666	Cytoplasm	High
P24752	Cytoskeletc	High
P24821	Cytoskeletc	High
P25098	Endoplasm	High
P25325	Cytoplasm	High
P25398	Cytoskeletc	High
P25686	Endoplasm	High
P25705	Cytoskeletc	High
P25786	Cytoskeletc	High
P25787	Cytoskeletc	High
P25789	Cytoskeletc	High
P26038	Cytoskeletc	High
P26232	Cytoskeletc	High
P26373	Cytoskeletc	High
P26440	Cytoskeletc	High
P26639	Cytoskeletc	High
P26640	Cytoskeletc	High
P26641	Cytoskeletc	High
P27144	Mitochondr	High
P27338	Cytoskeletc	High
P27348	Cytoskeletc	High
P27361	Cytoskeletc	High
P27449	Presynapse	High
P27635	Cytoskeletc	High
P27797	Cytoskeletc	High
P27816	Cytoskeletc	High
P27824	Cytoskeletc	High
P28066	Cytoskeletc	High
P28070	Cytoplasm	High
P28074	Cytoplasm	High
P28161	Cytoskeletc	High
P28288	Endoplasm	High
P28482	Cytoskeletc	High
P28838	Cytoskeletc	High
P29401	Cytoskeletc	High
P29972	Cytoskeletc	High
P29992	Plasma me	High
P30038	Cytoskeletc	High
P30040	Cytoskeletc	High
P30041	Cytoskeletc	High
P30043	Cytoskeletc	High

For Review Only

P30044	Cytoskeletc	High
P30046	Cytoskeletc	High
P30048	Cytoskeletc	High
P30049	Cytoskeletc	High
P30050	Cytoskeletc	High
P30084	Cytoskeletc	High
P30085	Cytoskeletc	High
P30086	Cytoskeletc	High
P30101	Cytoskeletc	High
P30153	Cytoskeletc	High
P30536	Endoplasm	High
P30626	Cytoskeletc	High
P30740	Cytoplasm	High
P30837	Mitochondr	High
P31040	Cytoskeletc	High
P31146	Cytoskeletc	High
P31150	Cytoskeletc	High
P31323	Cytoskeletc	High
P31930	Cytoskeletc	High
P31942	Nucleus	High
P31946	Cytoskeletc	High
P31948	Cytoskeletc	High
P31949	Cytoplasm	High
P32119	Cytoskeletc	High
P32455	Cytoplasm	High
P32969	Cytoskeletc	High
P34897	Mitochondr	High
P34932	Cytoskeletc	High
P35080	Cytoskeletc	High
P35222	Cytoskeletc	High
P35232	Cytoskeletc	High
P35237	Cytoplasm	High
P35241	Cytoskeletc	High
P35244	Cytoskeletc	High
P35268	Cytoskeletc	High
P35270	Cytoskeletc	High
P35573	Cytoskeletc	High
P35579	Cytoskeletc	High
P35580	Cytoskeletc	High
P35606	Endoplasm	High
P35609	Cytoskeletc	High
P35611	Cytoskeletc	High
P35612	Cytoskeletc	High
P35749	Nucleus	High
P36405	Cytoskeletc	High
P36507	Endoplasm	High
P36542	Cytoskeletc	High
P36543	Cytoskeletc	High
P36578	Cytoskeletc	High
P36776	Cytoskeletc	High
P36871	Cytoskeletc	High
P36955	Endoplasm	High
P36957	Cytoskeletc	High
P36959	Cytoplasm	High
P37802	Cytoplasm	High
P37837	Cytoskeletc	High

For Review Only

P37840	Cytoskeletc	High
P38117	Cytoskeletc	High
P38159	Nucleus	High
P38606	Cytoskeletc	High
P38646	Cytoskeletc	High
P38919	Nucleus	High
P39023	Cytoskeletc	High
P39060	Nucleus	High
P39210	Postsynaps	High
P39656	Endoplasm	High
P40123	Cytoskeletc	High
P40227	Cytoskeletc	High
P40616	Endoplasm	High
P40763	Endoplasm	High
P40925	Cytoskeletc	High
P40926	Cytoskeletc	High
P40939	Cytoskeletc	High
P41222	Cytoskeletc	High
P41240	Endoplasm	High
P41250	Cytoskeletc	High
P41586	Endoplasm	High
P41732	Endoplasm	High
P42025	Cytoskeletc	High
P42126	Cytoplasm	High
P42167	Nucleus	High
P42356	Plasma me	High
P42658	Cytoskeletc	High
P42704	Cytoskeletc	High
P42765	Cytoskeletc	High
P42858	Endoplasm	High
P43003	Cytoskeletc	High
P43004	Cytoskeletc	High
P43034	Cytoskeletc	High
P43243	Cytoskeletc	High
P43304	Cytoskeletc	High
P43490	Cytoskeletc	High
P43897	Postsynaps	High
P45880	Cytoskeletc	High
P45954	Mitochondr	High
P45974	Cytoskeletc	High
P46109	Cytoskeletc	High
P46379	Endoplasm	High
P46459	Cytoskeletc	High
P46776	Cytoskeletc	High
P46777	Cytoskeletc	High
P46781	Cytoskeletc	High
P46782	Cytoskeletc	High
P46783	Cytoskeletc	High
P46821	Cytoskeletc	High
P46926	Cytoskeletc	High
P46976	Cytoplasm	High
P47897	Endoplasm	High
P47985	Cytoskeletc	High
P48047	Cytoskeletc	High
P48066	Endoplasm	High
P48147	Cytoskeletc	High

For Review Only

P48163	Cytoskeletc	High
P48539	Cytoplasm	High
P48553	Endoplasm	High
P48643	Cytoskeletc	High
P48735	Cytoskeletc	High
P48739	Cytoplasm	High
P49189	Cytoskeletc	High
P49257	Endoplasm	High
P49327	Cytoskeletc	High
P49368	Cytoskeletc	High
P49407	Cytoskeletc	High
P49411	Cytoskeletc	High
P49418	Cytoskeletc	High
P49419	Cytoskeletc	High
P49588	Cytoskeletc	High
P49589	Cytoplasm	High
P49591	Cytoskeletc	High
P49720	Cytoskeletc	High
P49748	Cytoskeletc	High
P49755	Endoplasm	High
P49773	Cytoskeletc	High
P49802	Plasma me	High
P49821	Cytoskeletc	High
P49841	Endoplasm	High
P49902	Cytoplasm	High
P50135	Cytoplasm	High
P50148	Cytoskeletc	High
P50213	Cytoskeletc	High
P50336	Mitochondr	High
P50395	Cytoskeletc	High
P50416	Endoplasm	High
P50440	Cytoplasm	High
P50453	Cytoplasm	High
P50502	Cytoskeletc	High
P50570	Cytoskeletc	High
P50897	Cytoskeletc	High
P50990	Cytoskeletc	High
P50991	Cytoskeletc	High
P50993	Cytoskeletc	High
P50995	Cytoskeletc	High
P51114	Endoplasm	High
P51148	Cytoskeletc	High
P51149	Cytoskeletc	High
P51178	Cytoskeletc	High
P51452	Cytoskeletc	High
P51513	Endoplasm	High
P51553	Cytoskeletc	High
P51570	Endoplasm	High
P51608	Nucleus	High
P51649	Cytoskeletc	High
P51659	Cytoskeletc	High
P51693	Cytoplasm	High
P51858	Cytoplasm	High
P51911	Nucleus	High
P51970	Cytoskeletc	High
P51991	Cytoskeletc	High

For Review Only

P52209	Cytoskeletc	High
P52306	Cytoskeletc	High
P52565	Cytoskeletc	High
P52597	Nucleus	High
P52758	Cytoskeletc	High
P52888	Cytoplasm	High
P52943	Cytoskeletc	High
P53041	Cytoplasm	High
P53396	Cytoskeletc	High
P53618	Endoplasm	High
P53621	Endoplasm	High
P54289	Plasma me	High
P54577	Cytoskeletc	High
P54578	Cytoskeletc	High
P54619	Endoplasm	High
P54652	Cytoskeletc	High
P54687	Cytoplasm	High
P54727	Cytoplasm	High
P54750	Cytoskeletc	High
P54819	Cytoplasm	High
P54920	Cytoskeletc	High
P55010	Endoplasm	High
P55060	Cytoskeletc	High
P55072	Cytoskeletc	High
P55084	Cytoskeletc	High
P55087	Cytoskeletc	High
P55196	Cytoskeletc	High
P55209	Cytoskeletc	High
P55263	Cytoplasm	High
P55268	Nucleus	High
P55290	Plasma me	High
P55327	Cytoskeletc	High
P55786	Cytoskeletc	High
P55795	Cytoskeletc	High
P55809	Cytoskeletc	High
P55957	Cytoplasm	High
P56134	Cytoskeletc	High
P56181	Mitochondr	High
P56192	Presynapse	High
P56537	Endoplasm	High
P57087	Endoplasm	High
P58546	Cytoskeletc	High
P59768	Cytoskeletc	High
P60033	Cytoskeletc	High
P60174	Cytoskeletc	High
P60201	Cytoskeletc	High
P60520	Cytoskeletc	High
P60660	Cytoskeletc	High
P60709	Cytoskeletc	High
P60880	Cytoskeletc	High
P60891	Cytoskeletc	High
P60900	Cytoskeletc	High
P60953	Cytoskeletc	High
P60981	Cytoskeletc	High
P60983	Cytoskeletc	High
P61011	Endoplasm	High

For Review Only

P61018	Cytoskeletc	High
P61019	Cytoskeletc	High
P61020	Cytoskeletc	High
P61026	Plasma me	High
P61077	Cytoskeletc	High
P61086	Cytoplasm	High
P61088	Cytoskeletc	High
P61106	Cytoskeletc	High
P61163	Cytoskeletc	High
P61201	Cytoskeletc	High
P61247	Cytoskeletc	High
P61266	Cytoskeletc	High
P61353	Cytoskeletc	High
P61421	Cytoskeletc	High
P61586	Cytoskeletc	High
P61604	Cytoskeletc	High
P61619	Endoplasm	High
P61758	Cytoskeletc	High
P61764	Cytoskeletc	High
P61769	Cytoskeletc	High
P61916	Cytoplasm	High
P61925	Cytoplasm	High
P61970	Cytoskeletc	High
P61978	Cytoskeletc	High
P61981	Cytoskeletc	High
P62081	Cytoskeletc	High
P62140	Cytoskeletc	High
P62191	Endoplasm	High
P62241	Cytoskeletc	High
P62249	Cytoskeletc	High
P62258	Cytoskeletc	High
P62266	Plasma me	High
P62269	Cytoskeletc	High
P62277	Cytoskeletc	High
P62280	Cytoskeletc	High
P62310	Cytoplasm	High
P62314	Plasma me	High
P62328	Cytoskeletc	High
P62424	Cytoskeletc	High
P62701	Cytoskeletc	High
P62745	Cytoskeletc	High
P62750	Cytoskeletc	High
P62753	Cytoskeletc	High
P62760	Cytoskeletc	High
P62820	Cytoskeletc	High
P62826	Cytoskeletc	High
P62829	Cytoskeletc	High
P62834	Cytoskeletc	High
P62873	Cytoskeletc	High
P62879	Cytoskeletc	High
P62888	Cytoskeletc	High
P62906	Cytoskeletc	High
P62917	Cytoskeletc	High
P62937	Cytoskeletc	High
P62942	Cytoskeletc	High
P62979	Cytoskeletc	High

For Review Only

P62993	Cytoplasm	High
P62995	Nucleus	High
P63000	Cytoskeletc	High
P63010	Cytoskeletc	High
P63027	Cytoskeletc	High
P63096	Cytoskeletc	High
P63098	Cytoskeletc	High
P63104	Cytoskeletc	High
P63151	Cytoskeletc	High
P63172	Endoplasm	High
P63208	Cytoskeletc	High
P63241	Cytoskeletc	High
P63244	Cytoskeletc	High
P63313	Cytoskeletc	High
P67775	Cytoskeletc	High
P68032	Cytoskeletc	High
P68036	Cytoskeletc	High
P68104	Cytoskeletc	High
P68363	Cytoskeletc	High
P68366	Cytoskeletc	High
P68371	Cytoskeletc	High
P68400	Cytoskeletc	High
P68402	Cytoskeletc	High
P68431	Nucleus	High
P68871	Cytoskeletc	High
P69849	Endoplasm	High
P69891	Cytoplasm	High
P69905	Cytoskeletc	High
P78324	Cytoskeletc	High
P78344	Endoplasm	High
P78352	Cytoskeletc	High
P78357	Cytoskeletc	High
P78371	Cytoskeletc	High
P78417	Cytoskeletc	High
P78508	Endoplasm	High
P78559	Cytoskeletc	High
P80404	Cytoskeletc	High
P80723	Cytoskeletc	High
P83916	Cytoplasm	High
P84074	Cytoskeletc	High
P84077	Cytoskeletc	High
P84090	Cytoplasm	High
P84243	Cytoskeletc	High
P98160	Nucleus	High
P99999	Cytoskeletc	High
Q00013	Endoplasm	High
Q00169	Cytoskeletc	High
Q00325	Cytoskeletc	High
Q00341	Endoplasm	High
Q00535	Cytoskeletc	High
Q00577	Cytoskeletc	High
Q00610	Cytoskeletc	High
Q01082	Cytoskeletc	High
Q01105	Cytoskeletc	High
Q01130	Plasma me	High
Q01432	Cytoplasm	High

For Review Only

Q01469	Cytoskeletc	High
Q01484	Cytoskeletc	High
Q01813	Cytoskeletc	High
Q01995	Cytoskeletc	High
Q02218	Cytoskeletc	High
Q02252	Cytoskeletc	High
Q02750	Cytoskeletc	High
Q02790	Cytoskeletc	High
Q02818	Endoplasm	High
Q02878	Cytoskeletc	High
Q02952	Cytoskeletc	High
Q02978	Cytoskeletc	High
Q03001	Cytoskeletc	High
Q03154	Cytoplasm	High
Q04323	Cytoskeletc	High
Q04760	Cytoskeletc	High
Q04917	Cytoskeletc	High
Q05193	Cytoskeletc	High
Q05586	Mitochondr	High
Q05639	Cytoskeletc	High
Q06124	Cytoskeletc	High
Q06136	Plasma me	High
Q06323	Cytoplasm	High
Q06830	Cytoskeletc	High
Q07020	Cytoskeletc	High
Q07021	Cytoskeletc	High
Q07065	Endoplasm	High
Q07666	Nucleus	High
Q07954	Presynapse	High
Q07955	Plasma me	High
Q08209	Cytoskeletc	High
Q08257	Cytoskeletc	High
Q08495	Cytoskeletc	High
Q08623	Cytoplasm	High
Q09666	Cytoskeletc	High
Q0VDG4	Cytoplasm	High
Q10567	Cytoskeletc	High
Q12756	Endoplasm	High
Q12765	Cytoskeletc	High
Q12797	Endoplasm	High
Q12846	Endoplasm	High
Q12860	Cytoskeletc	High
Q12879	Mitochondr	High
Q12905	Cytoskeletc	High
Q12906	Cytoskeletc	High
Q12907	Endoplasm	High
Q12955	Cytoskeletc	High
Q12959	Cytoskeletc	High
Q12974	Endoplasm	High
Q12979	Plasma me	High
Q13011	Cytoskeletc	High
Q13126	Cytoplasm	High
Q13153	Cytoskeletc	High
Q13162	Endoplasm	High
Q13177	Cytoplasm	High
Q13200	Cytoskeletc	High

For Review Only

Q13224	Mitochondr	High
Q13228	Cytoskeletc	High
Q13303	Cytoskeletc	High
Q13336	Endoplasm	High
Q13363	Cytoskeletc	High
Q13404	Cytoplasm	High
Q13418	Endoplasm	High
Q13423	Cytoskeletc	High
Q13449	Plasma me	High
Q13509	Cytoskeletc	High
Q13510	Cytoskeletc	High
Q13526	Cytoskeletc	High
Q13554	Cytoskeletc	High
Q13555	Cytoskeletc	High
Q13557	Cytoskeletc	High
Q13561	Cytoskeletc	High
Q13620	Cytoplasm	High
Q13630	Cytoplasm	High
Q13724	Endoplasm	High
Q13813	Cytoskeletc	High
Q13838	Cytoskeletc	High
Q13867	Cytoplasm	High
Q13884	Endoplasm	High
Q13885	Cytoskeletc	High
Q13907	Cytoplasm	High
Q13938	Cytoplasm	High
Q14008	Plasma me	High
Q14019	Cytoskeletc	High
Q14103	Cytoskeletc	High
Q14108	Cytoskeletc	High
Q14112	Nucleus	High
Q14155	Plasma me	High
Q14157	Endoplasm	High
Q14165	Endoplasm	High
Q14194	Cytoskeletc	High
Q14195	Cytoskeletc	High
Q14203	Cytoskeletc	High
Q14204	Cytoskeletc	High
Q14232	Cytoplasm	High
Q14240	Cytoskeletc	High
Q14247	Cytoskeletc	High
Q14318	Endoplasm	High
Q14353	Cytoplasm	High
Q14515	Cytoskeletc	High
Q14624	Cytoplasm	High
Q14643	Endoplasm	High
Q14677	Endoplasm	High
Q14683	Nucleus	High
Q14697	Cytoskeletc	High
Q14764	Cytoskeletc	High
Q14894	Cytoskeletc	High
Q14914	Cytoplasm	High
Q14974	Cytoskeletc	High
Q14980	Nucleus	High
Q14982	Cytoskeletc	High
Q14CZ8	Cytoskeletc	High

For Review Only

Q15019	Cytoskeletc	High
Q15031	Mitochondr	High
Q15042	Endoplasm	High
Q15046	Endoplasm	High
Q15056	Plasma me	High
Q15102	Cytoskeletc	High
Q15119	Mitochondr	High
Q15120	Postsynaps	High
Q15121	Cytoskeletc	High
Q15126	Endoplasm	High
Q15149	Cytoskeletc	High
Q15165	Cytoskeletc	High
Q15181	Cytoskeletc	High
Q15257	Cytoskeletc	High
Q15274	Cytoplasm	High
Q15276	Endoplasm	High
Q15349	Endoplasm	High
Q15365	Cytoskeletc	High
Q15366	Cytoskeletc	High
Q15369	Endoplasm	High
Q15393	Cytoplasm	High
Q15435	Cytoskeletc	High
Q15493	Cytoplasm	High
Q15555	Cytoskeletc	High
Q15631	Cytoplasm	High
Q15691	Cytoskeletc	High
Q15700	Cytoskeletc	High
Q15714	Cytoskeletc	High
Q15738	Endoplasm	High
Q15746	Endoplasm	High
Q15811	Endoplasm	High
Q15813	Cytoplasm	High
Q15818	Plasma me	High
Q15819	Cytoskeletc	High
Q15843	Cytoskeletc	High
Q15847	Cytoplasm	High
Q15907	Cytoskeletc	High
Q15942	Cytoplasm	High
Q16143	Cytoskeletc	High
Q16181	Cytoskeletc	High
Q16222	Cytoplasm	High
Q16352	Cytoskeletc	High
Q16531	Cytoskeletc	High
Q16537	Cytoskeletc	High
Q16543	Cytoskeletc	High
Q16555	Cytoskeletc	High
Q16643	Cytoskeletc	High
Q16653	Cytoskeletc	High
Q16698	Cytoskeletc	High
Q16718	Cytoskeletc	High
Q16762	Cytoplasm	High
Q16773	Cytoskeletc	High
Q16775	Cytoskeletc	High
Q16777	Cytoskeletc	High
Q16778	Cytoskeletc	High
Q16795	Cytoskeletc	High

For Review Only

Q16798	Cytoskeletc	High
Q16799	Cytoskeletc	High
Q16836	Cytoskeletc	High
Q16849	Plasma me	High
Q16851	Cytoskeletc	High
Q16864	Cytoskeletc	High
Q16880	Endoplasm	High
Q16891	Cytoskeletc	High
Q17R89	Endoplasm	High
Q2M2I8	Cytoskeletc	High
Q2M389	Endoplasm	High
Q2NKQ1	Endoplasm	High
Q2TAA2	Cytoplasm	High
Q330K2	Mitochondr	High
Q3LXA3	Cytoplasm	High
Q3SXM5	Postsynaps	High
Q3ZCM7	Cytoskeletc	High
Q4G0N4	Cytoplasm	High
Q4G176	Mitochondr	High
Q4J6C6	Cytoskeletc	High
Q4KMQ2	Endoplasm	High
Q504Y0	Endoplasm	High
Q53GQ0	Cytoskeletc	High
Q53H12	Cytoskeletc	High
Q5H9L2	Cytoplasm	High
Q5HYK3	Mitochondr	High
Q5JTJ3	Cytoplasm	High
Q5JTZ9	Mitochondr	High
Q5JU85	Postsynaps	High
Q5SQI0	Endoplasm	High
Q5SSJ5	Cytoskeletc	High
Q5T440	Mitochondr	High
Q5T4S7	Plasma me	High
Q5T5P2	Mitochondr	High
Q5TF21	Endoplasm	High
Q5TFE4	Cytoplasm	High
Q5U4P2	Endoplasm	High
Q5VIR6	Endoplasm	High
Q5VST6	Endoplasm	High
Q5VWQ8	Mitochondr	High
Q5VYK3	Endoplasm	High
Q643R3	Endoplasm	High
Q66K14	Endoplasm	High
Q66K74	Plasma me	High
Q68DH5	Endoplasm	High
Q6BCY4	Cytoplasm	High
Q6DN90	Plasma me	High
Q6IAA8	Plasma me	High
Q6IBS0	Cytoskeletc	High
Q6NUK1	Mitochondr	High
Q6NXS1	Cytoplasm	High
Q6NZI2	Nucleus	High
Q6P2Q9	Nucleus	High
Q6P4A7	Mitochondr	High
Q6P587	Cytoskeletc	High
Q6P9B6	Endoplasm	High

For Review Only

Q6PCB7	Endoplasm	High
Q6PCE3	Cytoplasm	High
Q6PI78	Mitochondr	High
Q6PKG0	Endoplasm	High
Q6PUV4	Cytoskeletc	High
Q6U841	Plasma me	High
Q6UW68	Endoplasm	High
Q6UWE0	Endoplasm	High
Q6UWP2	Cytoplasm	High
Q6UWR7	Cytoskeletc	High
Q6XQN6	Cytoplasm	High
Q6Y7W6	Endoplasm	High
Q6YN16	Plasma me	High
Q6ZVM7	Cytoskeletc	High
Q71DI3	Nucleus	High
Q71U36	Cytoskeletc	High
Q71UM5	Endoplasm	High
Q7KZN9	Mitochondr	High
Q7L099	Cytoskeletc	High
Q7L0J3	Cytoskeletc	High
Q7L2E3	Endoplasm	High
Q7L3T8	Mitochondr	High
Q7L523	Endoplasm	High
Q7L775	Nucleus	High
Q7Z3J2	Endoplasm	High
Q7Z4H3	Cytoplasm	High
Q7Z4S6	Cytoskeletc	High
Q7Z4W1	Cytoskeletc	High
Q7Z6G3	Cytoskeletc	High
Q7Z6L0	Cytoskeletc	High
Q7Z6Z7	Cytoskeletc	High
Q7Z7A4	Endoplasm	High
Q7Z7H8	Mitochondr	High
Q86SK9	Endoplasm	High
Q86TV6	Mitochondr	High
Q86TX2	Cytoskeletc	High
Q86VP6	Cytoskeletc	High
Q86VU5	Mitochondr	High
Q86WA6	Mitochondr	High
Q86WU2	Mitochondr	High
Q86X24	Cytoskeletc	High
Q86Y82	Plasma me	High
Q86YN1	Endoplasm	High
Q8IUR0	Endoplasm	High
Q8IW45	Cytoskeletc	High
Q8IWA5	Presynapse	High
Q8IWQ3	Endoplasm	High
Q8IXJ6	Cytoskeletc	High
Q8IY17	Endoplasm	High
Q8IYQ7	Mitochondr	High
Q8IZP0	Presynapse	High
Q8N145	Cytoskeletc	High
Q8N163	Nucleus	High
Q8N183	Postsynaps	High
Q8N1A0	Nucleus	High
Q8N1B4	Endoplasm	High

For Review Only

Q8N2K0	Endoplasm	High
Q8N335	Cytoskeletc	High
Q8N3F0	Cytoskeletc	High
Q8N3J6	Cytoskeletc	High
Q8N461	Cytoplasm	High
Q8N573	Cytoskeletc	High
Q8N5S9	Endoplasm	High
Q8N5V2	Endoplasm	High
Q8N6C5	Endoplasm	High
Q8N6M0	Endoplasm	High
Q8N766	Endoplasm	High
Q8N7J2	Endoplasm	High
Q8N8N7	Cytoplasm	High
Q8N8S7	Cytoplasm	High
Q8NBM8	Endoplasm	High
Q8NBQ5	Endoplasm	High
Q8NBU5	Endoplasm	High
Q8NBX0	Cytoskeletc	High
Q8NCB2	Cytoskeletc	High
Q8NE62	Postsynaps	High
Q8NE86	Mitochondr	High
Q8NEU8	Endoplasm	High
Q8NF91	Cytoskeletc	High
Q8NFV4	Postsynaps	High
Q8NFX7	Endoplasm	High
Q8NFZ8	Cytoskeletc	High
Q8NHG7	Endoplasm	High
Q8TAM6	Cytoskeletc	High
Q8TB36	Cytoskeletc	High
Q8TBC4	Cytoplasm	High
Q8TC12	Endoplasm	High
Q8TCD5	Cytoplasm	High
Q8TCZ2	Presynapse	High
Q8TD22	Presynapse	High
Q8TDJ6	Cytoskeletc	High
Q8TDQ7	Cytoplasm	High
Q8TDW7	Cytoplasm	High
Q8WUK0	Mitochondr	High
Q8WUM4	Cytoskeletc	High
Q8WUY1	Endoplasm	High
Q8WWI5	Cytoskeletc	High
Q8WXF1	Nucleus	High
Q8WXF7	Cytoskeletc	High
Q8WXG6	Plasma me	High
Q8WXH0	Cytoplasm	High
Q8WXS5	Mitochondr	High
Q8WYP5	Cytoskeletc	High
Q8WZA0	Cytoplasm	High
Q8WZA9	Cytoplasm	High
Q92499	Cytoskeletc	High
Q92542	Endoplasm	High
Q92561	Cytoskeletc	High
Q92574	Endoplasm	High
Q92597	Cytoskeletc	High
Q92598	Cytoskeletc	High
Q92599	Cytoskeletc	High

For Review Only

Q92604	Endoplasm	High
Q92614	Cytoskelet	High
Q92616	Endoplasm	High
Q92667	Cytoplasm	High
Q92673	Endoplasm	High
Q92686	Cytoskelet	High
Q92688	Cytoplasm	High
Q92736	Plasma me	High
Q92752	Cytoskelet	High
Q92777	Cytoskelet	High
Q92823	Cytoskelet	High
Q92841	Cytoskelet	High
Q92854	Endoplasm	High
Q92896	Endoplasm	High
Q92900	Endoplasm	High
Q92945	Cytoplasm	High
Q92947	Cytoplasm	High
Q92974	Plasma me	High
Q93009	Cytoskelet	High
Q93050	Cytoskelet	High
Q93077	Cytoskelet	High
Q969E2	Endoplasm	High
Q969E4	Cytoplasm	High
Q969P0	Cytoskelet	High
Q969S9	Mitochondr	High
Q969T9	Endoplasm	High
Q969Z0	Mitochondr	High
Q96A00	Cytoskelet	High
Q96A33	Endoplasm	High
Q96A65	Endoplasm	High
Q96AB3	Cytoskelet	High
Q96AC1	Cytoskelet	High
Q96AE4	Cytoplasm	High
Q96AG4	Endoplasm	High
Q96AQ6	Cytoskelet	High
Q96AX1	Endoplasm	High
Q96BQ5	Mitochondr	High
Q96CN7	Cytoplasm	High
Q96CS3	Endoplasm	High
Q96CV9	Endoplasm	High
Q96CW1	Cytoskelet	High
Q96DA6	Mitochondr	High
Q96DG6	Cytoplasm	High
Q96EY1	Postsynaps	High
Q96F07	Cytoskelet	High
Q96F85	Cytoskelet	High
Q96FJ2	Cytoskelet	High
Q96FW1	Cytoskelet	High
Q96G03	Cytoplasm	High
Q96GG9	Cytoplasm	High
Q96GR2	Endoplasm	High
Q96GW7	Cytoskelet	High
Q96HN2	Cytoskelet	High
Q96HY6	Endoplasm	High
Q96I15	Cytoplasm	High
Q96I23	Mitochondr	High

For Review Only

Q96JE9	Cytoskeletc	High
Q96JG6	Endoplasm	High
Q96K17	Plasma me	High
Q96KG9	Endoplasm	High
Q96KN2	Cytoplasm	High
Q96KP1	Endoplasm	High
Q96KP4	Cytoskeletc	High
Q96MM6	Nucleus	High
Q96MZ0	Plasma me	High
Q96N66	Endoplasm	High
Q96NN9	Endoplasm	High
Q96PE7	Mitochondr	High
Q96PV0	Cytoskeletc	High
Q96QE2	Endoplasm	High
Q96QK1	Cytoskeletc	High
Q96RP9	Mitochondr	High
Q96RQ3	Postsynaps	High
Q96RT1	Endoplasm	High
Q96RW7	Cytoskeletc	High
Q96S97	Endoplasm	High
Q96SB3	Postsynaps	High
Q96SZ5	Cytoplasm	High
Q96T76	Endoplasm	High
Q96TC7	Cytoskeletc	High
Q99250	Plasma me	High
Q99436	Cytoskeletc	High
Q99471	Cytoskeletc	High
Q99497	Cytoskeletc	High
Q99536	Cytoskeletc	High
Q99574	Endoplasm	High
Q99584	Cytoskeletc	High
Q99598	Cytoplasm	High
Q99614	Cytoplasm	High
Q99615	Endoplasm	High
Q99623	Cytoskeletc	High
Q99643	Mitochondr	High
Q99714	Cytoskeletc	High
Q99719	Cytoskeletc	High
Q99733	Cytoskeletc	High
Q99766	Mitochondr	High
Q99798	Cytoskeletc	High
Q99805	Endoplasm	High
Q99807	Postsynaps	High
Q99816	Endoplasm	High
Q99832	Cytoskeletc	High
Q99873	Cytoplasm	High
Q99879	Nucleus	High
Q99962	Cytoskeletc	High
Q99963	Cytoskeletc	High
Q9BPU6	Cytoskeletc	High
Q9BPW8	Cytoskeletc	High
Q9BQ70	Endoplasm	High
Q9BR01	Cytoplasm	High
Q9BSH4	Postsynaps	High
Q9BT78	Cytoskeletc	High
Q9BTV4	Endoplasm	High

For Review Only

Q9BTW9	Endoplasm	High
Q9BU02	Cytoplasm	High
Q9BUF5	Cytoskeletc	High
Q9BUT1	Cytoplasm	High
Q9BV20	Endoplasm	High
Q9BVA1	Cytoskeletc	High
Q9BVK6	Nucleus	High
Q9BW30	Cytoskeletc	High
Q9BWD1	Cytoskeletc	High
Q9BWM7	Cytoskeletc	High
Q9BX68	Cytoskeletc	High
Q9BXK5	Cytoskeletc	High
Q9BXW7	Mitochondr	High
Q9BY11	Cytoskeletc	High
Q9BY67	Cytoskeletc	High
Q9BYB0	Postsynaps	High
Q9BYD6	Nucleus	High
Q9BZV1	Plasma me	High
Q9C040	Cytoskeletc	High
Q9C0B1	Cytoplasm	High
Q9C0C9	Cytoskeletc	High
Q9GZM7	Nucleus	High
Q9GZP4	Cytoplasm	High
Q9GZQ8	Cytoskeletc	High
Q9GZS3	Endoplasm	High
Q9GZV7	Cytoskeletc	High
Q9H008	Cytoskeletc	High
Q9H074	Cytoplasm	High
Q9H0A8	Endoplasm	High
Q9H0E2	Cytoskeletc	High
Q9H0R8	Endoplasm	High
Q9H0U4	Plasma me	High
Q9H115	Cytoskeletc	High
Q9H1E5	Endoplasm	High
Q9H1K1	Mitochondr	High
Q9H1P3	Endoplasm	High
Q9H269	Endoplasm	High
Q9H2H9	Endoplasm	High
Q9H2M9	Endoplasm	High
Q9H2U2	Cytoplasm	High
Q9H2X9	Cytoskeletc	High
Q9H3H3	Cytoplasm	High
Q9H3N1	Nucleus	High
Q9H3S7	Endoplasm	High
Q9H479	Cytoskeletc	High
Q9H492	Cytoskeletc	High
Q9H4A4	Cytoplasm	High
Q9H4A6	Endoplasm	High
Q9H4G0	Cytoskeletc	High
Q9H6K4	Postsynaps	High
Q9H6R3	Cytoplasm	High
Q9H6U6	Endoplasm	High
Q9H7D0	Endoplasm	High
Q9H936	Cytoskeletc	High
Q9H993	Cytoplasm	High
Q9H9B4	Cytoskeletc	High

For Review Only

Q9H9S4	Cytoplasm	High
Q9HA64	Cytoplasm	High
Q9HAR2	Endoplasm	High
Q9HAT2	Cytoplasm	High
Q9HAV7	Mitochondr	High
Q9HB71	Cytoskelet	High
Q9HB90	Endoplasm	High
Q9HBL0	Nucleus	High
Q9HCH3	Cytoskelet	High
Q9HCJ6	Cytoskelet	High
Q9HCM2	Plasma me	High
Q9HCP6	Endoplasm	High
Q9HD23	Mitochondr	High
Q9HDC9	Plasma me	High
Q9NNW7	Mitochondr	High
Q9NP79	Endoplasm	High
Q9NP80	Mitochondr	High
Q9NPF4	Cytoplasm	High
Q9NPQ8	Endoplasm	High
Q9NQ66	Cytoskelet	High
Q9NQ79	Presynapse	High
Q9NQ88	Cytoplasm	High
Q9NQC3	Cytoskelet	High
Q9NQW6	Cytoskelet	High
Q9NQX3	Cytoskelet	High
Q9NQX7	Endoplasm	High
Q9NR46	Cytoskelet	High
Q9NRV9	Cytoskelet	High
Q9NRW1	Cytoskelet	High
Q9NS86	Cytoskelet	High
Q9NSD9	Cytoskelet	High
Q9NSE4	Mitochondr	High
Q9NT62	Endoplasm	High
Q9NTJ4	Cytoplasm	High
Q9NTJ5	Endoplasm	High
Q9NTK5	Cytoskelet	High
Q9NUJ1	Cytoskelet	High
Q9NUQ2	Mitochondr	High
Q9NUQ9	Cytoskelet	High
Q9NV70	Endoplasm	High
Q9NVA2	Cytoskelet	High
Q9NVS9	Cytoplasm	High
Q9NWS8	Mitochondr	High
Q9NWV4	Cytoplasm	High
Q9NX14	Mitochondr	High
Q9NX63	Mitochondr	High
Q9NXA8	Mitochondr	High
Q9NY65	Cytoskelet	High
Q9NYB0	Nucleus	High
Q9NYB9	Presynapse	High
Q9NYI0	Cytoskelet	High
Q9NZ45	Cytoskelet	High
Q9NZB8	Mitochondr	High
Q9NZD2	Cytoskelet	High
Q9NZG7	Endoplasm	High
Q9NZL4	Cytoplasm	High

For Review Only

Q9NZL9	Cytoplasm	High
Q9NZM1	Endoplasm	High
Q9NZN3	Cytoskeletc	High
Q9P032	Postsynaps	High
Q9P0J1	Mitochondr	High
Q9P0J7	Endoplasm	High
Q9P0L0	Cytoskeletc	High
Q9P0S9	Endoplasm	High
Q9P0U1	Mitochondr	High
Q9P1F3	Cytoplasm	High
Q9P1U1	Cytoskeletc	High
Q9P253	Endoplasm	High
Q9P2J5	Presynapse	High
Q9P2R7	Cytoskeletc	High
Q9P2U7	Cytoskeletc	High
Q9P2W3	Endoplasm	High
Q9UBB6	Cytoskeletc	High
Q9UBC2	Cytoskeletc	High
Q9UBF2	Endoplasm	High
Q9UBI6	Presynapse	High
Q9UBN7	Endoplasm	High
Q9UBP0	Endoplasm	High
Q9UBP4	Cytoskeletc	High
Q9UBQ7	Cytoskeletc	High
Q9UBS5	Endoplasm	High
Q9UD71	Cytoplasm	High
Q9UDT6	Endoplasm	High
Q9UDY2	Endoplasm	High
Q9UEY8	Cytoskeletc	High
Q9UGM6	Mitochondr	High
Q9UH03	Cytoskeletc	High
Q9UH99	Nucleus	High
Q9UHA4	Presynapse	High
Q9UHD1	Cytoskeletc	High
Q9UHD8	Cytoskeletc	High
Q9UHL4	Cytoplasm	High
Q9UHV9	Cytoplasm	High
Q9UHX1	Nucleus	High
Q9UHY1	Endoplasm	High
Q9UHY7	Cytoskeletc	High
Q9UI12	Cytoskeletc	High
Q9UI15	Cytoskeletc	High
Q9UI47	Endoplasm	High
Q9UIC8	Cytoplasm	High
Q9UIG0	Cytoskeletc	High
Q9UJC5	Cytoskeletc	High
Q9UJU6	Cytoskeletc	High
Q9UJY5	Cytoplasm	High
Q9UJZ1	Mitochondr	High
Q9UK22	Cytoskeletc	High
Q9UK45	Cytoplasm	High
Q9UK76	Cytoplasm	High
Q9UKA9	Nucleus	High
Q9UKG1	Cytoskeletc	High
Q9UKK9	Cytoplasm	High
Q9UL12	Cytoskeletc	High

For Review Only

Q9UL15	Endoplasm	High
Q9UL46	Cytoplasm	High
Q9ULA0	Cytoplasm	High
Q9ULC4	Cytoskelet	High
Q9ULD0	Cytoskelet	High
Q9ULN7	Endoplasm	High
Q9ULR3	Cytoskelet	High
Q9ULU8	Cytoskelet	High
Q9ULV4	Cytoskelet	High
Q9UM19	Cytoskelet	High
Q9UN36	Cytoskelet	High
Q9UNE7	Endoplasm	High
Q9UNS2	Cytoskelet	High
Q9UNZ2	Cytoskelet	High
Q9UPA5	Cytoskelet	High
Q9UPN3	Cytoskelet	High
Q9UPT5	Endoplasm	High
Q9UPT6	Endoplasm	High
Q9UPX0	Postsynaps	High
Q9UPX8	Nucleus	High
Q9UPY5	Endoplasm	High
Q9UPY8	Cytoskelet	High
Q9UQ16	Cytoskelet	High
Q9UQ90	Mitochondr	High
Q9UQB8	Plasma me	High
Q9UQM7	Cytoskelet	High
Q9Y277	Cytoskelet	High
Q9Y285	Cytoskelet	High
Q9Y287	Endoplasm	High
Q9Y2A7	Cytoskelet	High
Q9Y2B0	Endoplasm	High
Q9Y2D4	Endoplasm	High
Q9Y2J0	Cytoskelet	High
Q9Y2J8	Cytoskelet	High
Q9Y2K9	Endoplasm	High
Q9Y2S2	Cytoskelet	High
Q9Y2T2	Endoplasm	High
Q9Y2T3	Cytoskelet	High
Q9Y2V2	Cytoplasm	High
Q9Y2X7	Plasma me	High
Q9Y2Z4	Mitochondr	High
Q9Y2Z9	Mitochondr	High
Q9Y320	Endoplasm	High
Q9Y371	Endoplasm	High
Q9Y3B3	Endoplasm	High
Q9Y3B8	Cytoplasm	High
Q9Y3C6	Endoplasm	High
Q9Y3D6	Plasma me	High
Q9Y3E1	Nucleus	High
Q9Y426	Endoplasm	High
Q9Y490	Cytoskelet	High
Q9Y4D1	Endoplasm	High
Q9Y4E1	Endoplasm	High
Q9Y4E6	Cytoskelet	High
Q9Y4F1	Plasma me	High
Q9Y4I1	Cytoskelet	High

For Review Only

Q9Y4L1	Cytoskeletc	High
Q9Y5K8	Cytoskeletc	High
Q9Y5P6	Cytoplasm	High
Q9Y5S2	Endoplasm	High
Q9Y5U8	Mitochondr	High
Q9Y5X3	Endoplasm	High
Q9Y5Z4	Cytoplasm	High
Q9Y617	Cytoskeletc	High
Q9Y678	Endoplasm	High
Q9Y696	Cytoskeletc	High
Q9Y697	Cytoskeletc	High
Q9Y6C9	Cytoskeletc	High
Q9Y6D6	Mitochondr	High
Q9Y6I3	Cytoskeletc	High
Q9Y6M9	Mitochondr	High
Q9Y6R1	Cytoskeletc	High
Q9Y6V0	Postsynaps	High
Q9Y6X5	Endoplasm	High
O00116	Cytoplasm	Low
O00192	Cytoplasm_	Low
O14744	Cytoplasm_	Low
O14841	Cytoplasm	Low
O14907	Cytoplasm_	Low
O43708	Cytoplasm_	Low
O43760	Cytoplasm_	Low
O60658	Cytoplasm	Low
O60749	Cytoplasm	Low
O60925	Cytoplasm_	Low
O75936	Cytoplasm_	Low
O95295	Cytoplasm	Low
P01591	Cytoplasm_	Low
P04114	Cytoplasm_	Low
P04217	Cytoplasm_	Low
P05155	Cytoplasm_	Low
P05452	Cytoplasm_	Low
P06730	Cytoplasm	Low
P07738	Cytoplasm_	Low
P08397	Cytoplasm_	Low
P09012	Cytoplasm	Low
P0C0L4	Cytoplasm	Low
P0DJI8	Cytoplasm_	Low
P10620	Cytoplasm_	Low
P20936	Cytoplasm_	Low
P22059	Cytoplasm	Low
P28072	Cytoplasm_	Low
P30876	Cytoplasm	Low
P32929	Cytoplasm_	Low
P34913	Cytoplasm_	Low
P34949	Cytoplasm_	Low
P37235	Cytoplasm_	Low
P40145	Cytoplasm_	Low
P40855	Cytoplasm_	Low
P42566	Cytoplasm_	Low
P48506	Cytoplasm_	Low
P49593	Cytoplasm_	Low
P49721	Cytoplasm_	Low

For Review Only

P50226	Cytoplasm	Low
P50579	Cytoplasm	Low
P53367	Cytoplasm	Low
P53582	Cytoplasm	Low
P61081	Cytoplasm	Low
P61204	Cytoplasm	Low
P61328	Cytoplasm	Low
P62495	Cytoplasm	Low
Q06481	Cytoplasm	Low
Q13617	Cytoplasm	Low
Q14978	Cytoplasm	Low
Q15363	Cytoplasm	Low
Q15417	Cytoplasm	Low
Q16881	Cytoplasm	Low
Q4G0P3	Cytoplasm	Low
Q52LD8	Cytoplasm	Low
Q562R1	Cytoplasm	Low
Q5H9R7	Cytoplasm	Low
Q5JSH3	Cytoplasm	Low
Q5VT25	Cytoplasm	Low
Q5VWZ2	Cytoplasm	Low
Q6IQ22	Cytoplasm	Low
Q7L2H7	Cytoplasm	Low
Q7Z7L7	Cytoplasm	Low
Q86VS8	Cytoplasm	Low
Q8ND24	Cytoplasm	Low
Q8NFU3	Cytoplasm	Low
Q8TEA8	Cytoplasm	Low
Q8TEQ8	Cytoplasm	Low
Q8TF30	Cytoplasm	Low
Q8WWI1	Cytoplasm	Low
Q8WXC6	Cytoplasm	Low
Q92696	Cytoplasm	Low
Q92905	Cytoplasm	Low
Q92990	Cytoplasm	Low
Q96AJ9	Cytoplasm	Low
Q96DZ1	Cytoplasm	Low
Q96IU4	Cytoplasm	Low
Q96JQ2	Cytoplasm	Low
Q96S66	Cytoplasm	Low
Q9BSH5	Cytoplasm	Low
Q9BTE1	Cytoplasm	Low
Q9BW62	Cytoplasm	Low
Q9BXS0	Cytoplasm	Low
Q9BXS5	Cytoplasm	Low
Q9C0D9	Cytoplasm	Low
Q9H2C0	Cytoplasm	Low
Q9H2W6	Cytoplasm	Low
Q9H330	Cytoplasm	Low
Q9NQ48	Cytoplasm	Low
Q9NRY5	Cytoplasm	Low
Q9NRY6	Cytoplasm	Low
Q9P2D7	Cytoplasm	Low
Q9UBB4	Cytoplasm	Low
Q9Y295	Cytoplasm	Low
Q9Y2H9	Cytoplasm	Low

For Review Only

Q9Y3C8	Cytoplasm_	Low
Q9Y3U8	Cytoplasm_	Low
Q9Y4E8	Cytoplasm	Low
Q9Y4F4	Cytoplasm_	Low
Q9Y6R7	Cytoplasm_	Low
E9PAV3	Cytoskeletc	Low
O00401	Cytoskeletc	Low
O00487	Cytoskeletc	Low
O14561	Cytoskeletc	Low
O14775	Cytoskeletc	Low
O14807	Cytoskeletc	Low
O15173	Cytoskeletc	Low
O43399	Cytoskeletc	Low
O43687	Cytoskeletc	Low
O60268	Cytoskeletc	Low
O75131	Cytoskeletc	Low
O75380	Cytoskeletc	Low
O75506	Cytoskeletc	Low
O76054	Cytoskeletc	Low
O94905	Cytoskeletc	Low
O94919	Cytoskeletc	Low
O95716	Cytoskeletc	Low
P01860	Cytoskeletc	Low
P02730	Cytoskeletc	Low
P05166	Cytoskeletc	Low
P13521	Cytoskeletc	Low
P16112	Cytoskeletc	Low
P19784	Cytoskeletc	Low
P20339	Cytoskeletc	Low
P21810	Cytoskeletc	Low
P21912	Cytoskeletc	Low
P26378	Cytoskeletc	Low
P26599	Cytoskeletc	Low
P30711	Cytoskeletc	Low
P31689	Cytoskeletc	Low
P33176	Cytoskeletc	Low
P33402	Cytoskeletc	Low
P35998	Cytoskeletc	Low
P36915	Cytoskeletc	Low
P42224	Cytoskeletc	Low
P43007	Cytoskeletc	Low
P43155	Cytoskeletc	Low
P49758	Cytoskeletc	Low
P50454	Cytoskeletc	Low
P51575	Cytoskeletc	Low
P52788	Cytoskeletc	Low
P52815	Cytoskeletc	Low
P53602	Cytoskeletc	Low
P56385	Cytoskeletc	Low
P61254	Cytoskeletc	Low
P61313	Cytoskeletc	Low
P62333	Cytoskeletc	Low
P62851	Cytoskeletc	Low
P83731	Cytoskeletc	Low
P84085	Cytoskeletc	Low
Q00765	Cytoskeletc	Low

For Review Only

Q02153	Cytoskeletc	Low
Q02156	Cytoskeletc	Low
Q04837	Cytoskeletc	Low
Q08722	Cytoskeletc	Low
Q13596	Cytoskeletc	Low
Q13616	Cytoskeletc	Low
Q13825	Cytoskeletc	Low
Q14289	Cytoskeletc	Low
Q14738	Cytoskeletc	Low
Q15814	Cytoskeletc	Low
Q16401	Cytoskeletc	Low
Q16620	Cytoskeletc	Low
Q27J81	Cytoskeletc	Low
Q58FF8	Cytoskeletc	Low
Q5JRX3	Cytoskeletc	Low
Q684P5	Cytoskeletc	Low
Q6H8Q1	Cytoskeletc	Low
Q709C8	Cytoskeletc	Low
Q7Z460	Cytoskeletc	Low
Q86VW0	Cytoskeletc	Low
Q8IZD9	Cytoskeletc	Low
Q8N1G4	Cytoskeletc	Low
Q8N568	Cytoskeletc	Low
Q8N6T3	Cytoskeletc	Low
Q8N8Y2	Cytoskeletc	Low
Q8NB37	Cytoskeletc	Low
Q8WXD2	Cytoskeletc	Low
Q92530	Cytoskeletc	Low
Q92783	Cytoskeletc	Low
Q92882	Cytoskeletc	Low
Q92973	Cytoskeletc	Low
Q969H8	Cytoskeletc	Low
Q96CX2	Cytoskeletc	Low
Q96EE3	Cytoskeletc	Low
Q96HU8	Cytoskeletc	Low
Q99685	Cytoskeletc	Low
Q99747	Cytoskeletc	Low
Q99829	Cytoskeletc	Low
Q99961	Cytoskeletc	Low
Q9BTV5	Cytoskeletc	Low
Q9C0H9	Cytoskeletc	Low
Q9GZY8	Cytoskeletc	Low
Q9H254	Cytoskeletc	Low
Q9H4M3	Cytoskeletc	Low
Q9NP97	Cytoskeletc	Low
Q9NRN7	Cytoskeletc	Low
Q9NTX5	Cytoskeletc	Low
Q9NVJ2	Cytoskeletc	Low
Q9UBQ0	Cytoskeletc	Low
Q9UBV8	Cytoskeletc	Low
Q9UIA9	Cytoskeletc	Low
Q9UL25	Cytoskeletc	Low
Q9UNM6	Cytoskeletc	Low
Q9Y305	Cytoskeletc	Low
Q9Y4C0	Cytoskeletc	Low
O00178	Endoplasm	Low

For Review Only

O00194	Endoplasm Low
O00231	Endoplasm Low
O00232	Endoplasm Low
O00291	Endoplasm Low
O00478	Endoplasm Low
O00629	Endoplasm Low
O14558	Endoplasm Low
O14653	Endoplasm Low
O14662	Endoplasm Low
O14936	Endoplasm Low
O14974	Endoplasm Low
O15118	Endoplasm Low
O15127	Endoplasm Low
O15372	Endoplasm Low
O15498	Endoplasm Low
O43592	Endoplasm Low
O43854	Endoplasm Low
O60331	Endoplasm Low
O60493	Endoplasm Low
O60613	Endoplasm Low
O60716	Endoplasm Low
O60739	Endoplasm Low
O75110	Endoplasm Low
O75155	Endoplasm Low
O75348	Endoplasm Low
O75351	Endoplasm Low
O75896	Endoplasm Low
O94911	Endoplasm Low
O95219	Endoplasm Low
O95671	Endoplasm Low
O95674	Endoplasm Low
O95825	Endoplasm Low
O95837	Endoplasm Low
O95881	Endoplasm Low
P01903	Endoplasm Low
P02656	Endoplasm Low
P02746	Endoplasm Low
P02747	Endoplasm Low
P02749	Endoplasm Low
P04003	Endoplasm Low
P04844	Endoplasm Low
P05362	Endoplasm Low
P06241	Endoplasm Low
P07947	Endoplasm Low
P08123	Endoplasm Low
P08240	Endoplasm Low
P08575	Endoplasm Low
P0C7M8	Endoplasm Low
P14209	Endoplasm Low
P15374	Endoplasm Low
P15882	Endoplasm Low
P17540	Endoplasm Low
P17900	Endoplasm Low
P18507	Endoplasm Low
P20337	Endoplasm Low
P20774	Endoplasm Low

For Review Only

	P23141	Endoplasm Low
	P23258	Endoplasm Low
	P25685	Endoplasm Low
	P26012	Endoplasm Low
	P27701	Endoplasm Low
	P28907	Endoplasm Low
	P30453	Endoplasm Low
	P35637	Endoplasm Low
	P36269	Endoplasm Low
	P41208	Endoplasm Low
	P41567	Endoplasm Low
	P42330	Endoplasm Low
	P43307	Endoplasm Low
	P46060	Endoplasm Low
	P48723	Endoplasm Low
	P49006	Endoplasm Low
	P50150	Endoplasm Low
	P50583	Endoplasm Low
	P51572	Endoplasm Low
	P51784	Endoplasm Low
	P51790	Endoplasm Low
	P52294	Endoplasm Low
	P53007	Endoplasm Low
	P53365	Endoplasm Low
	P54284	Endoplasm Low
	P55011	Endoplasm Low
	P55160	Endoplasm Low
	P61221	Endoplasm Low
	P61513	Endoplasm Low
	P62068	Endoplasm Low
	P62166	Endoplasm Low
	P62273	Endoplasm Low
	P62308	Endoplasm Low
	P67870	Endoplasm Low
	P98194	Endoplasm Low
	Q01064	Endoplasm Low
	Q01433	Endoplasm Low
	Q03135	Endoplasm Low
	Q04609	Endoplasm Low
	Q05469	Endoplasm Low
	Q08462	Endoplasm Low
	Q12768	Endoplasm Low
	Q12904	Endoplasm Low
	Q12965	Endoplasm Low
	Q13045	Endoplasm Low
	Q13057	Endoplasm Low
	Q13099	Endoplasm Low
	Q13155	Endoplasm Low
	Q13505	Endoplasm Low
	Q13586	Endoplasm Low
	Q13875	Endoplasm Low
	Q14160	Endoplasm Low
	Q14185	Endoplasm Low
	Q14315	Endoplasm Low
	Q14416	Endoplasm Low
	Q14571	Endoplasm Low

For Review Only

Q14696	Endoplasm	Low
Q14721	Endoplasm	Low
Q14956	Endoplasm	Low
Q15049	Endoplasm	Low
Q15067	Endoplasm	Low
Q15293	Endoplasm	Low
Q15599	Endoplasm	Low
Q15773	Endoplasm	Low
Q3KQU3	Endoplasm	Low
Q4KMP7	Endoplasm	Low
Q5T5C0	Endoplasm	Low
Q5TCZ1	Endoplasm	Low
Q5VUB5	Endoplasm	Low
Q5VW32	Endoplasm	Low
Q5VWJ9	Endoplasm	Low
Q5VZK9	Endoplasm	Low
Q6P2E9	Endoplasm	Low
Q6P995	Endoplasm	Low
Q6PIU2	Endoplasm	Low
Q6YP21	Endoplasm	Low
Q6ZSS7	Endoplasm	Low
Q6ZT12	Endoplasm	Low
Q7L5N7	Endoplasm	Low
Q7Z392	Endoplasm	Low
Q7Z406	Endoplasm	Low
Q86SZ2	Endoplasm	Low
Q86TP1	Endoplasm	Low
Q86V81	Endoplasm	Low
Q86WG3	Endoplasm	Low
Q86YQ8	Endoplasm	Low
Q8IUR7	Endoplasm	Low
Q8IV01	Endoplasm	Low
Q8IV38	Endoplasm	Low
Q8IVD9	Endoplasm	Low
Q8IWT6	Endoplasm	Low
Q8IXI2	Endoplasm	Low
Q8IYI6	Endoplasm	Low
Q8IZ83	Endoplasm	Low
Q8IZS8	Endoplasm	Low
Q8N0X7	Endoplasm	Low
Q8N2F6	Endoplasm	Low
Q8N3E9	Endoplasm	Low
Q8N468	Endoplasm	Low
Q8N4V2	Endoplasm	Low
Q8N5K1	Endoplasm	Low
Q8NBF6	Endoplasm	Low
Q8NC51	Endoplasm	Low
Q8NCA5	Endoplasm	Low
Q8NE01	Endoplasm	Low
Q8NF37	Endoplasm	Low
Q8NFP9	Endoplasm	Low
Q8TAF3	Endoplasm	Low
Q8TBB6	Endoplasm	Low
Q8TBG9	Endoplasm	Low
Q8WU79	Endoplasm	Low
Q8WVC6	Endoplasm	Low

For Review Only

Q8WW22	Endoplasm	Low
Q8WXE9	Endoplasm	Low
Q92520	Endoplasm	Low
Q92556	Endoplasm	Low
Q92932	Endoplasm	Low
Q93100	Endoplasm	Low
Q969G6	Endoplasm	Low
Q969Z3	Endoplasm	Low
Q96AG3	Endoplasm	Low
Q96AM1	Endoplasm	Low
Q96CT7	Endoplasm	Low
Q96DB2	Endoplasm	Low
Q96DD7	Endoplasm	Low
Q96E17	Endoplasm	Low
Q96FE5	Endoplasm	Low
Q96FZ7	Endoplasm	Low
Q96RL7	Endoplasm	Low
Q96RR4	Endoplasm	Low
Q96S52	Endoplasm	Low
Q99622	Endoplasm	Low
Q99683	Endoplasm	Low
Q99784	Endoplasm	Low
Q9BQI7	Endoplasm	Low
Q9BRK0	Endoplasm	Low
Q9BSA4	Endoplasm	Low
Q9BWS9	Endoplasm	Low
Q9BX66	Endoplasm	Low
Q9BX67	Endoplasm	Low
Q9BZ29	Endoplasm	Low
Q9BZF1	Endoplasm	Low
Q9H019	Endoplasm	Low
Q9H1K0	Endoplasm	Low
Q9H305	Endoplasm	Low
Q9H4G4	Endoplasm	Low
Q9H598	Endoplasm	Low
Q9HA65	Endoplasm	Low
Q9HBH5	Endoplasm	Low
Q9HBI6	Endoplasm	Low
Q9HD42	Endoplasm	Low
Q9NP72	Endoplasm	Low
Q9NPD7	Endoplasm	Low
Q9NSK0	Endoplasm	Low
Q9NX40	Endoplasm	Low
Q9NY35	Endoplasm	Low
Q9NZ01	Endoplasm	Low
Q9NZJ4	Endoplasm	Low
Q9NZQ3	Endoplasm	Low
Q9P0L2	Endoplasm	Low
Q9P1Y5	Endoplasm	Low
Q9P265	Endoplasm	Low
Q9UGI6	Endoplasm	Low
Q9UHB9	Endoplasm	Low
Q9UHI5	Endoplasm	Low
Q9UID3	Endoplasm	Low
Q9UJ41	Endoplasm	Low
Q9UJQ1	Endoplasm	Low

For Review Only

Q9ULC3	Endoplasm	Low
Q9UM22	Endoplasm	Low
Q9UM54	Endoplasm	Low
Q9UMX5	Endoplasm	Low
Q9UMY4	Endoplasm	Low
Q9UNA1	Endoplasm	Low
Q9UNH7	Endoplasm	Low
Q9UNK0	Endoplasm	Low
Q9UPQ0	Endoplasm	Low
Q9UPQ3	Endoplasm	Low
Q9UPY6	Endoplasm	Low
Q9Y265	Endoplasm	Low
Q9Y2E4	Endoplasm	Low
Q9Y2H1	Endoplasm	Low
Q9Y4P8	Endoplasm	Low
Q9Y5K5	Endoplasm	Low
Q9Y5Y2	Endoplasm	Low
Q9Y6B6	Endoplasm	Low
Q9Y6E0	Endoplasm	Low
Q9Y6K8	Endoplasm	Low
A2RU67	Mitochondr	Low
O00165	Mitochondr	Low
O00305	Mitochondr	Low
O00471	Mitochondr	Low
O14490	Mitochondr	Low
O15034	Mitochondr	Low
O15068	Mitochondr	Low
O15294	Mitochondr	Low
O43678	Mitochondr	Low
O43739	Mitochondr	Low
O60503	Mitochondr	Low
O75521	Mitochondr	Low
O75962	Mitochondr	Low
O94830	Mitochondr	Low
O94966	Mitochondr	Low
O95139	Mitochondr	Low
O95167	Mitochondr	Low
O95886	Mitochondr	Low
O96008	Mitochondr	Low
P00846	Mitochondr	Low
P03891	Mitochondr	Low
P11274	Mitochondr	Low
P17152	Mitochondr	Low
P22830	Mitochondr	Low
P23677	Mitochondr	Low
P24588	Mitochondr	Low
P35498	Mitochondr	Low
P36404	Mitochondr	Low
P43487	Mitochondr	Low
P50402	Mitochondr	Low
P52594	Mitochondr	Low
P54803	Mitochondr	Low
P56378	Mitochondr	Low
P78369	Mitochondr	Low
P82909	Mitochondr	Low
Q08499	Mitochondr	Low

For Review Only

Q08AE8	Mitochondr	Low
Q12792	Mitochondr	Low
Q12849	Mitochondr	Low
Q12931	Mitochondr	Low
Q13015	Mitochondr	Low
Q13232	Mitochondr	Low
Q13362	Mitochondr	Low
Q14161	Mitochondr	Low
Q14C86	Mitochondr	Low
Q15582	Mitochondr	Low
Q5RI15	Mitochondr	Low
Q5SRE7	Mitochondr	Low
Q5TC12	Mitochondr	Low
Q6FI81	Mitochondr	Low
Q6PGP7	Mitochondr	Low
Q6ZMI0	Mitochondr	Low
Q86UW7	Mitochondr	Low
Q86XE3	Mitochondr	Low
Q8IZU2	Mitochondr	Low
Q8NFJ9	Mitochondr	Low
Q8NFZ4	Mitochondr	Low
Q8TDC3	Mitochondr	Low
Q8WXI2	Mitochondr	Low
Q92529	Mitochondr	Low
Q92538	Mitochondr	Low
Q96A26	Mitochondr	Low
Q96IX5	Mitochondr	Low
Q96JJ7	Mitochondr	Low
Q96LT7	Mitochondr	Low
Q96RF0	Mitochondr	Low
Q96RS6	Mitochondr	Low
Q96SW2	Mitochondr	Low
Q96TA1	Mitochondr	Low
Q99490	Mitochondr	Low
Q9BQG1	Mitochondr	Low
Q9BRK4	Mitochondr	Low
Q9BW72	Mitochondr	Low
Q9C026	Mitochondr	Low
Q9HBL8	Mitochondr	Low
Q9HC56	Mitochondr	Low
Q9HD34	Mitochondr	Low
Q9NR48	Mitochondr	Low
Q9NRA0	Mitochondr	Low
Q9NS69	Mitochondr	Low
Q9NVH1	Mitochondr	Low
Q9NZJ7	Mitochondr	Low
Q9UDW1	Mitochondr	Low
Q9UIQ6	Mitochondr	Low
Q9Y2G8	Mitochondr	Low
Q9Y4P1	Mitochondr	Low
Q9Y4W6	Mitochondr	Low
Q9Y653	Mitochondr	Low
O15523	Nucleus	Low
O75592	Nucleus	Low
O75923	Nucleus	Low
P01861	Nucleus	Low

For Review Only

P05164	Nucleus	Low
P07305	Nucleus	Low
P08311	Nucleus	Low
P16157	Nucleus	Low
P16403	Nucleus_Pl	Low
P30484	Nucleus	Low
Q02880	Nucleus	Low
Q03426	Nucleus	Low
Q08431	Nucleus	Low
Q13243	Nucleus	Low
Q14011	Nucleus	Low
Q16853	Nucleus	Low
Q5VZ66	Nucleus	Low
Q68DU8	Nucleus	Low
Q6NUQ4	Nucleus_P(	Low
Q6P1X6	Nucleus	Low
Q86YS7	Nucleus	Low
Q8N5R6	Nucleus	Low
Q8NHP6	Nucleus	Low
Q8TAC2	Nucleus	Low
Q92522	Nucleus_Pl	Low
Q96D09	Nucleus	Low
Q9BSF0	Nucleus	Low
Q9BST9	Nucleus	Low
Q9H902	Nucleus	Low
Q9NRR3	Nucleus	Low
Q9UBQ5	Nucleus	Low
Q9UJD0	Nucleus_P(	Low
O14508	Plasma me	Low
O15400	Plasma me	Low
O75077	Plasma me	Low
O75569	Plasma me	Low
O75822	Plasma me	Low
P08621	Plasma me	Low
P16389	Plasma me	Low
P17980	Plasma me	Low
P40429	Plasma me	Low
P42677	Plasma me	Low
P42766	Plasma me	Low
P49207	Plasma me	Low
P53999	Plasma me	Low
P54764	Plasma me	Low
P62316	Plasma me	Low
P63162	Plasma me	Low
Q05329	Plasma me	Low
Q13347	Plasma me	Low
Q13409	Plasma me	Low
Q13442	Plasma me	Low
Q13492	Plasma me	Low
Q14444	Plasma me	Low
Q15286	Plasma me	Low
Q15334	Plasma me	Low
Q8NFW8	Plasma me	Low
Q8NHM4	Plasma me	Low
Q92859	Plasma me	Low
Q96ID5	Plasma me	Low

For Review Only

Q99613	Plasma me	Low
Q9H4M9	Plasma me	Low
Q9NUJ3	Plasma me	Low
Q9UHC6	Plasma me	Low
Q9UHQ9	Plasma me	Low
Q9UN86	Plasma me	Low
Q9Y3A5	Plasma me	Low
Q9Y536	Plasma me	Low
P03905	Postsynaps	Low
P14867	Postsynaps	Low
P15056	Postsynaps	Low
P17568	Postsynaps	Low
P54829	Postsynaps	Low
Q07002	Postsynaps	Low
Q16134	Postsynaps	Low
Q9BVA0	Postsynaps	Low
Q9C0H5	Postsynaps	Low
Q9ULJ8	Postsynaps	Low
Q9Y241	Postsynaps	Low
Q9Y2Q3	Postsynaps	Low
Q9Y512	Postsynaps	Low
O43272	Presynapse	Low
O75145	Presynapse	Low
O75146	Presynapse	Low
O75821	Presynapse	Low
O95502	Presynapse	Low
O95573	Presynapse	Low
P17931	Presynapse	Low
Q13085	Presynapse	Low
Q14832	Presynapse	Low
Q5FWE3	Presynapse	Low
Q7KZF4	Presynapse	Low
Q86T65	Presynapse	Low
Q8N4P6	Presynapse	Low
Q8N9I0	Presynapse	Low
Q8ND76	Presynapse	Low
Q8TB96	Presynapse	Low
Q92930	Presynapse	Low
Q9NUM4	Presynapse	Low
Q9UN37	Presynapse	Low
A0A024RB	Endoplasm	Medium
A0AVT1	Endoplasm	Medium
A1L0T0	Plasma me	Medium
A2RTX5	Endoplasm	Medium
A5PKW4	Mitochondr	Medium
A5YM72	Cytoskelet	Medium
A6NE02	Endoplasm	Medium
O00139	Plasma me	Medium
O00159	Nucleus	Medium
O00170	Endoplasm	Medium
O00186	Endoplasm	Medium
O00217	Postsynaps	Medium
O00264	Plasma me	Medium
O00330	Cytoskelet	Medium
O00399	Cytoplasm	Medium
O00408	Cytoskelet	Medium

For Review Only

O00571	Endoplasm	Medium
O00625	Cytoplasm	Medium
O14495	Presynapse	Medium
O14525	Endoplasm	Medium
O14579	Endoplasm	Medium
O14787	Endoplasm	Medium
O14810	Cytoskelet	Medium
O14828	Endoplasm	Medium
O14910	Endoplasm	Medium
O14964	Cytoskelet	Medium
O14976	Presynapse	Medium
O14979	Nucleus	Medium
O14980	Cytoskelet	Medium
O14994	Cytoskelet	Medium
O15066	Endoplasm	Medium
O15069	Endoplasm	Medium
O15079	Postsynaps	Medium
O15212	Cytoplasm	Medium
O15240	Plasma me	Medium
O15371	Plasma me	Medium
O15439	Nucleus	Medium
O15484	Plasma me	Medium
O43149	Mitochondr	Medium
O43157	Endoplasm	Medium
O43169	Presynapse	Medium
O43242	Cytoskelet	Medium
O43295	Endoplasm	Medium
O43427	Endoplasm	Medium
O43464	Mitochondr	Medium
O43504	Plasma me	Medium
O43505	Endoplasm	Medium
O43581	Presynapse	Medium
O43681	Cytoskelet	Medium
O43747	Cytoskelet	Medium
O43752	Endoplasm	Medium
O43759	Presynapse	Medium
O43765	Cytoskelet	Medium
O43809	Plasma me	Medium
O60220	Postsynaps	Medium
O60245	Endoplasm	Medium
O60262	Plasma me	Medium
O60307	Mitochondr	Medium
O60346	Cytoskelet	Medium
O60684	Cytoskelet	Medium
O60762	Endoplasm	Medium
O60784	Cytoskelet	Medium
O60861	Presynapse	Medium
O60869	Endoplasm	Medium
O60883	Presynapse	Medium
O60884	Plasma me	Medium
O60939	Endoplasm	Medium
O75027	Mitochondr	Medium
O75044	Mitochondr	Medium
O75051	Endoplasm	Medium
O75122	Plasma me	Medium
O75170	Endoplasm	Medium

For Review Only

O75208	Mitochondr	Medium
O75251	Mitochondr	Medium
O75323	Cytoskeletc	Medium
O75340	Cytoskeletc	Medium
O75352	Endoplasm	Medium
O75382	Endoplasm	Medium
O75431	Mitochondr	Medium
O75438	Mitochondr	Medium
O75508	Cytoskeletc	Medium
O75531	Cytoskeletc	Medium
O75689	Endoplasm	Medium
O75874	Cytoskeletc	Medium
O75899	Presynapse	Medium
O75935	Cytoskeletc	Medium
O75964	Postsynaps	Medium
O76003	Endoplasm	Medium
O76041	Cytoskeletc	Medium
O76094	Endoplasm	Medium
O94772	Presynapse	Medium
O94805	Nucleus	Medium
O94832	Cytoskeletc	Medium
O94875	Mitochondr	Medium
O94903	Endoplasm	Medium
O94915	Cytoskeletc	Medium
O94967	Endoplasm	Medium
O95140	Presynapse	Medium
O95168	Postsynaps	Medium
O95182	Mitochondr	Medium
O95196	Endoplasm	Medium
O95294	Endoplasm	Medium
O95298	Mitochondr	Medium
O95319	Endoplasm	Medium
O95563	Mitochondr	Medium
O95741	Plasma me	Medium
O95847	Mitochondr	Medium
O95858	Endoplasm	Medium
O95970	Presynapse	Medium
P00488	Cytoplasm	Medium
P00966	Cytoplasm	Medium
P01116	Presynapse	Medium
P01303	Endoplasm	Medium
P01859	Cytoplasm	Medium
P01871	Endoplasm	Medium
P01876	Cytoskeletc	Medium
P02745	Endoplasm	Medium
P02790	Cytoskeletc	Medium
P03886	Mitochondr	Medium
P03915	Mitochondr	Medium
P04040	Cytoplasm	Medium
P04264	Cytoskeletc	Medium
P04843	Presynapse	Medium
P05060	Mitochondr	Medium
P05107	Endoplasm	Medium
P05129	Cytoskeletc	Medium
P05165	Mitochondr	Medium
P05198	Presynapse	Medium

For Review Only

P05455	Cytoplasm	Medium
P06756	Plasma me	Medium
P07093	Endoplasm	Medium
P07858	Cytoplasm	Medium
P07951	Cytoplasm	Medium
P08183	Endoplasm	Medium
P08246	Nucleus	Medium
P08571	Cytoplasm	Medium
P0CG30	Cytoplasm	Medium
P10253	Cytoplasm	Medium
P10301	Plasma me	Medium
P10644	Endoplasm	Medium
P11166	Presynapse	Medium
P11277	Mitochondr	Medium
P11310	Cytoplasm	Medium
P11413	Cytoskelet	Medium
P11586	Cytoskelet	Medium
P12081	Cytoskelet	Medium
P12109	Endoplasm	Medium
P12110	Endoplasm	Medium
P12111	Plasma me	Medium
P12268	Endoplasm	Medium
P12270	Nucleus	Medium
P12931	Endoplasm	Medium
P12955	Cytoplasm	Medium
P12956	Cytoskelet	Medium
P13667	Endoplasm	Medium
P13693	Cytoskelet	Medium
P13726	Presynapse	Medium
P13807	Endoplasm	Medium
P13987	Plasma me	Medium
P14621	Cytoplasm	Medium
P14868	Presynapse	Medium
P15170	Cytoskelet	Medium
P15927	Endoplasm	Medium
P15954	Mitochondr	Medium
P16070	Plasma me	Medium
P16083	Cytoplasm	Medium
P16104	Cytoskelet	Medium
P16298	Cytoplasm	Medium
P17252	Cytoskelet	Medium
P18085	Cytoskelet	Medium
P18433	Endoplasm	Medium
P19404	Cytoskelet	Medium
P20290	Plasma me	Medium
P20338	Cytoskelet	Medium
P21397	Plasma me	Medium
P21695	Cytoplasm	Medium
P22102	Endoplasm	Medium
P22676	Cytoplasm	Medium
P23434	Cytoskelet	Medium
P23468	Endoplasm	Medium
P23469	Endoplasm	Medium
P23588	Cytoplasm	Medium
P23610	Endoplasm	Medium
P23743	Mitochondr	Medium

For Review Only

P23786	Mitochondr	Medium
P25713	Cytoplasm	Medium
P25788	Cytoplasm	Medium
P26196	Endoplasm	Medium
P26368	Cytoskelet	Medium
P27544	Endoplasm	Medium
P27695	Cytoskelet	Medium
P27986	Endoplasm	Medium
P28676	Endoplasm	Medium
P29120	Mitochondr	Medium
P29144	Cytoskelet	Medium
P29218	Cytoskelet	Medium
P29762	Cytoplasm	Medium
P30405	Mitochondr	Medium
P30419	Plasma me	Medium
P30519	Plasma me	Medium
P30520	Cytoplasm	Medium
P31153	Cytoplasm	Medium
P31937	Cytoskelet	Medium
P31939	Cytoskelet	Medium
P31943	Cytoskelet	Medium
P31947	Cytoplasm	Medium
P32189	Mitochondr	Medium
P33316	Mitochondr	Medium
P35520	Cytoskelet	Medium
P35613	Plasma me	Medium
P35754	Cytoplasm	Medium
P36222	Cytoplasm	Medium
P36873	Endoplasm	Medium
P36969	Plasma me	Medium
P37108	Cytoskelet	Medium
P38405	Mitochondr	Medium
P39019	Cytoskelet	Medium
P39687	Cytoskelet	Medium
P40121	Cytoplasm	Medium
P41091	Plasma me	Medium
P41217	Plasma me	Medium
P41252	Presynapse	Medium
P42262	Postsynaps	Medium
P42263	Mitochondr	Medium
P42345	Endoplasm	Medium
P42785	Cytoplasm	Medium
P43121	Presynapse	Medium
P43686	Cytoskelet	Medium
P45381	Cytoskelet	Medium
P45985	Cytoskelet	Medium
P46108	Cytoskelet	Medium
P46439	Cytoplasm	Medium
P46778	Cytoskelet	Medium
P46779	Plasma me	Medium
P46939	Endoplasm	Medium
P46940	Cytoskelet	Medium
P46952	Cytoskelet	Medium
P47736	Endoplasm	Medium
P47870	Postsynaps	Medium
P47914	Plasma me	Medium

For Review Only

P48426	Cytoskeletc	Medium
P48444	Endoplasm	Medium
P48556	Endoplasm	Medium
P48637	Cytoplasm	Medium
P49354	Endoplasm	Medium
P49441	Cytoskeletc	Medium
P49815	Endoplasm	Medium
P49840	Endoplasm	Medium
P49915	Cytoskeletc	Medium
P50151	Plasma me	Medium
P50914	Plasma me	Medium
P51398	Mitochondr	Medium
P51610	Nucleus	Medium
P51648	Endoplasm	Medium
P51665	Cytoskeletc	Medium
P51808	Cytoskeletc	Medium
P51888	Plasma me	Medium
P52429	Endoplasm	Medium
P53004	Cytoskeletc	Medium
P53597	Cytoskeletc	Medium
P53677	Presynapse	Medium
P53680	Cytoskeletc	Medium
P53779	Cytoskeletc	Medium
P53990	Endoplasm	Medium
P53992	Endoplasm	Medium
P54136	Presynapse	Medium
P54725	Cytoplasm	Medium
P55036	Endoplasm	Medium
P55735	Cytoskeletc	Medium
P55769	Nucleus	Medium
P55854	Cytoplasm	Medium
P55884	Presynapse	Medium
P56556	Postsynaps	Medium
P57737	Endoplasm	Medium
P58549	Presynapse	Medium
P60228	Endoplasm	Medium
P60842	Endoplasm	Medium
P60866	Plasma me	Medium
P60903	Presynapse	Medium
P61006	Endoplasm	Medium
P61224	Plasma me	Medium
P61225	Presynapse	Medium
P61457	Cytoplasm	Medium
P61601	Cytoskeletc	Medium
P61923	Endoplasm	Medium
P61960	Cytoplasm	Medium
P62070	Mitochondr	Medium
P62195	Cytoskeletc	Medium
P62244	Plasma me	Medium
P62263	Cytoskeletc	Medium
P62304	Plasma me	Medium
P62306	Nucleus	Medium
P62318	Plasma me	Medium
P62330	Mitochondr	Medium
P62837	Cytoplasm	Medium
P62841	Plasma me	Medium

For Review Only

P62847	Cytoskeletc	Medium
P62854	Plasma me	Medium
P62857	Plasma me	Medium
P62861	Plasma me	Medium
P62899	Plasma me	Medium
P62910	Plasma me	Medium
P62913	Cytoskeletc	Medium
P63167	Cytoskeletc	Medium
P63173	Plasma me	Medium
P63215	Cytoskeletc	Medium
P63218	Endoplasm	Medium
P63220	Plasma me	Medium
P78356	Cytoskeletc	Medium
P78362	Plasma me	Medium
P78527	Cytoskeletc	Medium
P84098	Endoplasm	Medium
P84103	Cytoskeletc	Medium
P98179	Nucleus	Medium
P98196	Cytoskeletc	Medium
Q00059	Postsynaps	Medium
Q00587	Nucleus	Medium
Q00688	Presynapse	Medium
Q01650	Endoplasm	Medium
Q01844	Nucleus	Medium
Q02108	Cytoplasm	Medium
Q02224	Mitochondr	Medium
Q02246	Cytoskeletc	Medium
Q02338	Postsynaps	Medium
Q02543	Presynapse	Medium
Q03013	Cytoplasm	Medium
Q04446	Endoplasm	Medium
Q04637	Endoplasm	Medium
Q04828	Endoplasm	Medium
Q07157	Cytoskeletc	Medium
Q07866	Cytoskeletc	Medium
Q07960	Cytoskeletc	Medium
Q08174	Plasma me	Medium
Q08380	Cytoskeletc	Medium
Q08752	Endoplasm	Medium
Q08828	Endoplasm	Medium
Q08AM6	Presynapse	Medium
Q09028	Nucleus	Medium
Q10713	Mitochondr	Medium
Q12791	Endoplasm	Medium
Q13017	Endoplasm	Medium
Q13098	Cytoskeletc	Medium
Q13131	Endoplasm	Medium
Q13148	Endoplasm	Medium
Q13185	Cytoplasm	Medium
Q13332	Endoplasm	Medium
Q13367	Cytoskeletc	Medium
Q13424	Plasma me	Medium
Q13496	Endoplasm	Medium
Q13564	Cytoplasm	Medium
Q13574	Nucleus	Medium
Q13613	Endoplasm	Medium

For Review Only

Q13618	Cytoplasm	Medium
Q13642	Cytoplasm	Medium
Q13683	Endoplasm	Medium
Q14118	Cytoplasm	Medium
Q14123	Endoplasm	Medium
Q14139	Endoplasm	Medium
Q14141	Cytoskelet	Medium
Q14152	Plasma me	Medium
Q14156	Endoplasm	Medium
Q14168	Cytoskelet	Medium
Q14257	Endoplasm	Medium
Q14344	Plasma me	Medium
Q14376	Cytoplasm	Medium
Q14558	Cytoskelet	Medium
Q14642	Presynapse	Medium
Q14699	Mitochondr	Medium
Q14964	Cytoplasm	Medium
Q15008	Endoplasm	Medium
Q15041	Presynapse	Medium
Q15075	Cytoplasm	Medium
Q15084	Plasma me	Medium
Q15111	Endoplasm	Medium
Q15185	Plasma me	Medium
Q15223	Endoplasm	Medium
Q15370	Cytoskelet	Medium
Q15382	Endoplasm	Medium
Q15388	Postsynaps	Medium
Q15404	Cytoskelet	Medium
Q15436	Endoplasm	Medium
Q15750	Endoplasm	Medium
Q15751	Endoplasm	Medium
Q16204	Cytoskelet	Medium
Q16566	Endoplasm	Medium
Q16629	Plasma me	Medium
Q16630	Plasma me	Medium
Q16774	Cytoskelet	Medium
Q3KQV9	Cytoplasm	Medium
Q3YEC7	Endoplasm	Medium
Q3ZCQ8	Mitochondr	Medium
Q3ZCW2	Cytoskelet	Medium
Q49A26	Nucleus	Medium
Q4G0F5	Cytoskelet	Medium
Q4V328	Endoplasm	Medium
Q52LJ0	Nucleus	Medium
Q53HC9	Endoplasm	Medium
Q59EK9	Endoplasm	Medium
Q5JPH6	Mitochondr	Medium
Q5JTD7	Endoplasm	Medium
Q5SYC1	Endoplasm	Medium
Q5T0D9	Plasma me	Medium
Q5T848	Endoplasm	Medium
Q5TGZ0	Mitochondr	Medium
Q5TH69	Endoplasm	Medium
Q5TZA2	Cytoskelet	Medium
Q5VSL9	Mitochondr	Medium
Q5VT66	Cytoplasm	Medium

For Review Only

Q5XKP0	Mitochondr	Medium
Q68D91	Endoplasm	Medium
Q6GMV3	Endoplasm	Medium
Q6IAN0	Endoplasm	Medium
Q6IQ20	Endoplasm	Medium
Q6KCM7	Mitochondr	Medium
Q6L8Q7	Mitochondr	Medium
Q6NV74	Mitochondr	Medium
Q6NVY1	Cytoskeletc	Medium
Q6P3W7	Endoplasm	Medium
Q6PFW1	Endoplasm	Medium
Q6PJW8	Endoplasm	Medium
Q6UXD5	Mitochondr	Medium
Q6VY07	Endoplasm	Medium
Q6WCQ1	Postsynaps	Medium
Q6ZVL6	Endoplasm	Medium
Q71UI9	Cytoskeletc	Medium
Q765P7	Endoplasm	Medium
Q7L1I2	Cytoskeletc	Medium
Q7L1Q6	Endoplasm	Medium
Q7L1S5	Endoplasm	Medium
Q7L266	Cytoplasm	Medium
Q7L576	Endoplasm	Medium
Q7L5N1	Cytoplasm	Medium
Q7Z2K8	Cytoplasm	Medium
Q7Z3D6	Mitochondr	Medium
Q7Z3U7	Endoplasm	Medium
Q86SX6	Mitochondr	Medium
Q86UP2	Cytoskeletc	Medium
Q86UR5	Mitochondr	Medium
Q86UW8	Presynapse	Medium
Q86UX6	Endoplasm	Medium
Q86UY8	Mitochondr	Medium
Q86V88	Cytoplasm	Medium
Q86X10	Endoplasm	Medium
Q86X55	Endoplasm	Medium
Q86X76	Cytoskeletc	Medium
Q86Y39	Cytoskeletc	Medium
Q86YM7	Postsynaps	Medium
Q8IUS5	Mitochondr	Medium
Q8IV08	Endoplasm	Medium
Q8IVF2	Cytoskeletc	Medium
Q8IVP5	Mitochondr	Medium
Q8IWB7	Endoplasm	Medium
Q8IXS8	Endoplasm	Medium
Q8IY22	Endoplasm	Medium
Q8IYB4	Endoplasm	Medium
Q8IYB5	Presynapse	Medium
Q8IYJ1	Mitochondr	Medium
Q8N111	Cytoskeletc	Medium
Q8N122	Endoplasm	Medium
Q8N135	Endoplasm	Medium
Q8N1F7	Nucleus	Medium
Q8N1I0	Endoplasm	Medium
Q8N3P4	Endoplasm	Medium
Q8N3V7	Postsynaps	Medium

For Review Only

Q8N414	Mitochondr	Medium
Q8N465	Mitochondr	Medium
Q8N4C8	Endoplasm	Medium
Q8N4Q0	Mitochondr	Medium
Q8N5H7	Mitochondr	Medium
Q8N5J2	Endoplasm	Medium
Q8N668	Endoplasm	Medium
Q8N684	Nucleus	Medium
Q8N6N7	Cytoplasm	Medium
Q8N987	Cytoskelet	Medium
Q8N9F0	Mitochondr	Medium
Q8N9F7	Endoplasm	Medium
Q8N9N7	Plasma me	Medium
Q8N9R8	Cytoskelet	Medium
Q8NBF2	Cytoplasm	Medium
Q8NBN7	Mitochondr	Medium
Q8NBS9	Endoplasm	Medium
Q8NC96	Cytoskelet	Medium
Q8NCG7	Plasma me	Medium
Q8NCW5	Cytoplasm	Medium
Q8NDA8	Endoplasm	Medium
Q8NDH3	Cytoplasm	Medium
Q8NE71	Endoplasm	Medium
Q8NEB9	Mitochondr	Medium
Q8NHH9	Endoplasm	Medium
Q8TAT6	Endoplasm	Medium
Q8TBF2	Endoplasm	Medium
Q8TBX8	Cytoskelet	Medium
Q8TCU6	Endoplasm	Medium
Q8TF44	Mitochondr	Medium
Q8WUD1	Endoplasm	Medium
Q8WUW1	Presynapse	Medium
Q8WUX9	Mitochondr	Medium
Q8WUY3	Mitochondr	Medium
Q8WVM8	Endoplasm	Medium
Q8WXD9	Endoplasm	Medium
Q8WY22	Mitochondr	Medium
Q8WZ42	Cytoskelet	Medium
Q92558	Cytoskelet	Medium
Q92572	Endoplasm	Medium
Q92581	Endoplasm	Medium
Q92734	Cytoplasm	Medium
Q92743	Endoplasm	Medium
Q92796	Mitochondr	Medium
Q92820	Cytoplasm	Medium
Q92845	Endoplasm	Medium
Q92982	Endoplasm	Medium
Q93008	Endoplasm	Medium
Q93034	Cytoplasm	Medium
Q969Q0	Plasma me	Medium
Q96BM9	Cytoskelet	Medium
Q96C19	Cytoplasm	Medium
Q96CM8	Mitochondr	Medium
Q96DA2	Endoplasm	Medium
Q96DE0	Nucleus	Medium
Q96DZ9	Endoplasm	Medium

For Review Only

Q96EK5	Endoplasm	Medium
Q96EQ0	Cytoskeletc	Medium
Q96F24	Mitochondr	Medium
Q96FC7	Cytoskeletc	Medium
Q96FN4	Endoplasm	Medium
Q96FQ6	Cytoplasm	Medium
Q96GA7	Endoplasm	Medium
Q96GD0	Cytoskeletc	Medium
Q96GK7	Cytoplasm	Medium
Q96GQ5	Endoplasm	Medium
Q96HE7	Endoplasm	Medium
Q96HR9	Endoplasm	Medium
Q96HS1	Mitochondr	Medium
Q96JH7	Cytoskeletc	Medium
Q96L92	Endoplasm	Medium
Q96NW7	Mitochondr	Medium
Q96P47	Plasma me	Medium
Q96P70	Endoplasm	Medium
Q96PE3	Endoplasm	Medium
Q96PE5	Endoplasm	Medium
Q96PU5	Mitochondr	Medium
Q96PU8	Endoplasm	Medium
Q96PY5	Plasma me	Medium
Q96Q05	Endoplasm	Medium
Q96QR8	Endoplasm	Medium
Q99259	Endoplasm	Medium
Q99426	Cytoskeletc	Medium
Q99447	Cytoplasm	Medium
Q99460	Cytoskeletc	Medium
Q99523	Endoplasm	Medium
Q99569	Mitochondr	Medium
Q99572	Presynapse	Medium
Q99627	Cytoskeletc	Medium
Q99653	Presynapse	Medium
Q99729	Cytoplasm	Medium
Q99767	Mitochondr	Medium
Q99884	Endoplasm	Medium
Q99943	Endoplasm	Medium
Q99996	Plasma me	Medium
Q9BQ69	Mitochondr	Medium
Q9BQE5	Cytoskeletc	Medium
Q9BQI5	Cytoskeletc	Medium
Q9BQI9	Cytoplasm	Medium
Q9BR76	Cytoskeletc	Medium
Q9BRX8	Plasma me	Medium
Q9BSJ8	Cytoskeletc	Medium
Q9BSU1	Endoplasm	Medium
Q9BTE6	Endoplasm	Medium
Q9BV23	Endoplasm	Medium
Q9BV79	Mitochondr	Medium
Q9BXF6	Mitochondr	Medium
Q9BXJ9	Endoplasm	Medium
Q9BXW6	Cytoskeletc	Medium
Q9BZC7	Endoplasm	Medium
Q9BZE9	Cytoplasm	Medium
Q9BZF3	Endoplasm	Medium

For Review Only

Q9BZZ5	Endoplasm	Medium
Q9C0E8	Endoplasm	Medium
Q9C0G6	Endoplasm	Medium
Q9GZM8	Nucleus	Medium
Q9GZN7	Presynapse	Medium
Q9GZT3	Mitochondr	Medium
Q9GZT4	Endoplasm	Medium
Q9GZT6	Mitochondr	Medium
Q9GZT8	Cytoskelet	Medium
Q9GZZ9	Cytoskelet	Medium
Q9H061	Mitochondr	Medium
Q9H078	Mitochondr	Medium
Q9H0B6	Endoplasm	Medium
Q9H0Q0	Cytoskelet	Medium
Q9H0Q3	Cytoskelet	Medium
Q9H0R4	Cytoskelet	Medium
Q9H0W9	Cytoplasm	Medium
Q9H1K4	Mitochondr	Medium
Q9H1Z4	Postsynaps	Medium
Q9H244	Endoplasm	Medium
Q9H270	Endoplasm	Medium
Q9H299	Cytoplasm	Medium
Q9H2G2	Endoplasm	Medium
Q9H313	Presynapse	Medium
Q9H3K6	Cytoplasm	Medium
Q9H425	Endoplasm	Medium
Q9H444	Endoplasm	Medium
Q9H6K5	Endoplasm	Medium
Q9H6L5	Mitochondr	Medium
Q9H7Z7	Cytoskelet	Medium
Q9H845	Mitochondr	Medium
Q9H8H3	Plasma me	Medium
Q9H9A6	Endoplasm	Medium
Q9H9C1	Endoplasm	Medium
Q9H9H5	Endoplasm	Medium
Q9H9J2	Mitochondr	Medium
Q9H9Q2	Cytoplasm	Medium
Q9H9S3	Endoplasm	Medium
Q9HA77	Mitochondr	Medium
Q9HAV0	Cytoskelet	Medium
Q9HBF4	Presynapse	Medium
Q9HC38	Cytoskelet	Medium
Q9HCC0	Cytoskelet	Medium
Q9HCD6	Postsynaps	Medium
Q9NP78	Nucleus	Medium
Q9NP81	Mitochondr	Medium
Q9NPJ3	Cytoskelet	Medium
Q9NQC7	Mitochondr	Medium
Q9NQE9	Presynapse	Medium
Q9NQP4	Cytoplasm	Medium
Q9NQR4	Cytoplasm	Medium
Q9NQW7	Cytoplasm	Medium
Q9NR28	Cytoplasm	Medium
Q9NR31	Endoplasm	Medium
Q9NR45	Cytoplasm	Medium
Q9NRD5	Nucleus	Medium

For Review Only

Q9NRG7	Mitochondr	Medium
Q9NRQ2	Endoplasm	Medium
Q9NRW7	Endoplasm	Medium
Q9NRX4	Plasma me	Medium
Q9NRY4	Endoplasm	Medium
Q9NRZ7	Endoplasm	Medium
Q9NSY0	Endoplasm	Medium
Q9NUB1	Cytoskelet	Medium
Q9NUP9	Endoplasm	Medium
Q9NUU7	Mitochondr	Medium
Q9NV96	Endoplasm	Medium
Q9NVE7	Endoplasm	Medium
Q9NVI7	Mitochondr	Medium
Q9NVT9	Mitochondr	Medium
Q9NWU2	Endoplasm	Medium
Q9NY47	Presynapse	Medium
Q9NYU2	Endoplasm	Medium
Q9NZ52	Endoplasm	Medium
Q9NZ56	Endoplasm	Medium
Q9NZH0	Endoplasm	Medium
Q9NZJ6	Mitochondr	Medium
Q9NZN4	Nucleus	Medium
Q9NZW5	Endoplasm	Medium
Q9P000	Endoplasm	Medium
Q9P035	Cytoskelet	Medium
Q9P0J0	Cytoskelet	Medium
Q9P0K1	Plasma me	Medium
Q9P0Z9	Endoplasm	Medium
Q9P260	Endoplasm	Medium
Q9P2E9	Endoplasm	Medium
Q9P2R3	Plasma me	Medium
Q9P2T1	Cytoplasm	Medium
Q9UBE0	Endoplasm	Medium
Q9UBW8	Cytoskelet	Medium
Q9UBX3	Mitochondr	Medium
Q9UDY4	Endoplasm	Medium
Q9UEW8	Cytoskelet	Medium
Q9UF11	Postsynaps	Medium
Q9UFN0	Endoplasm	Medium
Q9UGC6	Endoplasm	Medium
Q9UGT4	Endoplasm	Medium
Q9UHD2	Mitochondr	Medium
Q9UHG2	Cytoskelet	Medium
Q9UHG3	Presynapse	Medium
Q9UHQ4	Endoplasm	Medium
Q9UI09	Postsynaps	Medium
Q9UI40	Endoplasm	Medium
Q9UII2	Mitochondr	Medium
Q9UIJ7	Cytoskelet	Medium
Q9UJ70	Cytoskelet	Medium
Q9UJA5	Endoplasm	Medium
Q9UJS0	Mitochondr	Medium
Q9UK41	Endoplasm	Medium
Q9UKU0	Cytoskelet	Medium
Q9UKU6	Endoplasm	Medium
Q9UL26	Endoplasm	Medium

For Review Only

Q9UL51	Endoplasm	Medium
Q9ULB1	Endoplasm	Medium
Q9ULK0	Endoplasm	Medium
Q9ULP0	Cytoskelet	Medium
Q9ULP9	Plasma me	Medium
Q9ULT8	Nucleus	Medium
Q9UMF0	Cytoskelet	Medium
Q9UMS4	Nucleus	Medium
Q9UNF0	Cytoskelet	Medium
Q9UNN5	Mitochondr	Medium
Q9UNW9	Endoplasm	Medium
Q9UPR0	Endoplasm	Medium
Q9UPR5	Plasma me	Medium
Q9UPU5	Mitochondr	Medium
Q9UPV7	Cytoskelet	Medium
Q9UQ03	Cytoskelet	Medium
Q9UQ80	Cytoskelet	Medium
Q9UQB3	Plasma me	Medium
Q9Y224	Cytoskelet	Medium
Q9Y230	Endoplasm	Medium
Q9Y262	Endoplasm	Medium
Q9Y263	Endoplasm	Medium
Q9Y266	Cytoskelet	Medium
Q9Y276	Postsynaps	Medium
Q9Y2C4	Mitochondr	Medium
Q9Y2I1	Cytoplasm	Medium
Q9Y2I8	Endoplasm	Medium
Q9Y2Q0	Plasma me	Medium
Q9Y2Q5	Endoplasm	Medium
Q9Y2Z0	Cytoskelet	Medium
Q9Y333	Cytoplasm	Medium
Q9Y342	Nucleus	Medium
Q9Y376	Cytoskelet	Medium
Q9Y394	Plasma me	Medium
Q9Y3C4	Cytoplasm	Medium
Q9Y3F4	Cytoskelet	Medium
Q9Y3I0	Cytoskelet	Medium
Q9Y3P9	Endoplasm	Medium
Q9Y496	Endoplasm	Medium
Q9Y4D8	Endoplasm	Medium
Q9Y4F5	Cytoskelet	Medium
Q9Y4G8	Cytoskelet	Medium
Q9Y4J8	Endoplasm	Medium
Q9Y4Y9	Cytoplasm	Medium
Q9Y566	Mitochondr	Medium
Q9Y570	Cytoskelet	Medium
Q9Y584	Mitochondr	Medium
Q9Y5J7	Postsynaps	Medium
Q9Y5L0	Endoplasm	Medium
Q9Y5X1	Endoplasm	Medium
Q9Y6D5	Endoplasm	Medium
Q9Y6G9	Plasma me	Medium
O43572	NA	None
O75334	NA	None
O75436	NA	None
O75937	NA	None

For Review Only

P08603	NA	None
P12694	NA	None
P13645	NA	None
P29692	NA	None
P57088	NA	None
P59666	NA	None
Q14166	NA	None
Q15904	NA	None
Q63HM9	NA	None
Q68CZ2	NA	None
Q6PL24	NA	None
Q8IU85	NA	None
Q8TF61	NA	None
Q92504	NA	None
Q96D71	NA	None
Q96EY7	NA	None
Q96I99	NA	None
Q96JJ3	NA	None
Q9BWQ8	NA	None
Q9C005	NA	None
Q9HCJ1	NA	None
Q9NZZ3	NA	None
Q9P0V9	NA	None
Q9P2G1	NA	None
Q9UMX0	NA	None
Q9Y6T7	NA	None

For Review Only
