# Supplementary material for: Proteomic characterization of post-mortem human brain tissue following ultracentrifugation-based subcellular fractionation
Source: Brain Commun. 2022 Apr 21;4(3):fcac103. doi: 10.1093/braincomms/fcac103 (PMC9123841; doi:10.1093/braincomms/fcac103)
Supplement: fcac103_Supplementary_Data [file fcac103_supplementary_data.zip › Supplementary Figures.pdf]

Supplementary Figure 1

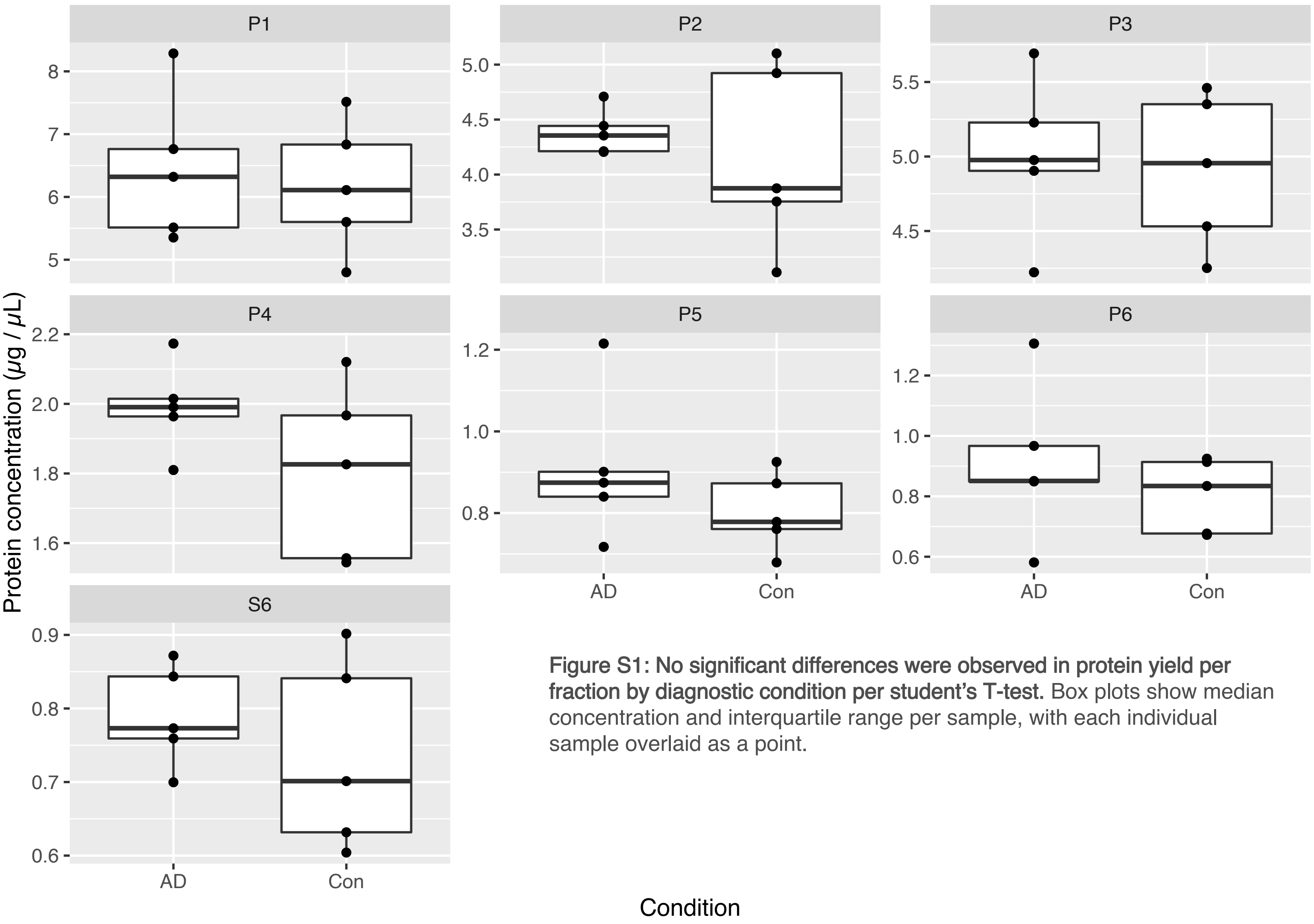

Figure S1: No significant differences were observed in protein yield per fraction by diagnostic condition per student's T-test. Box plots show median concentration and interquartile range per sample, with each individual sample overlaid as a point.

Supplementary Figure 2

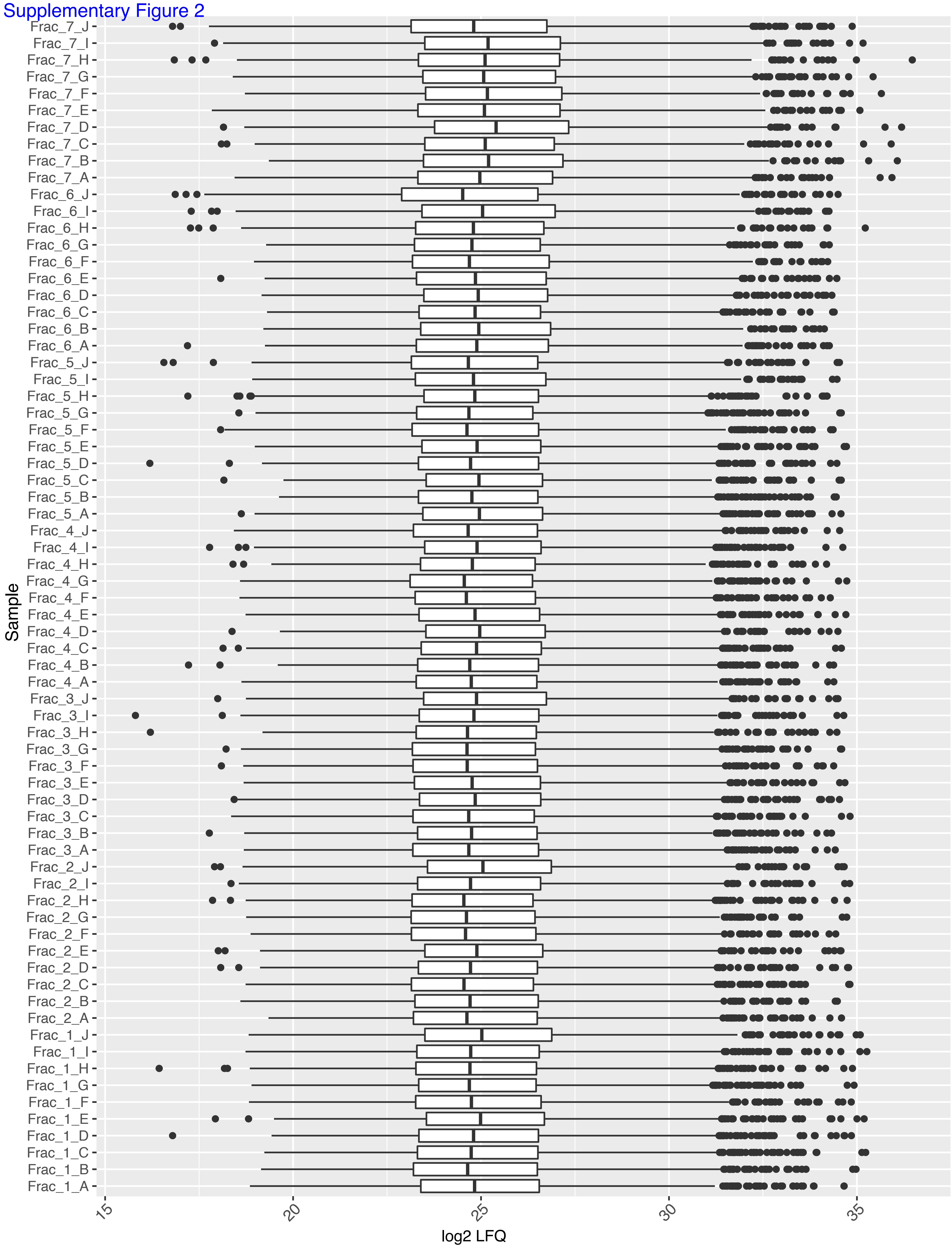

Figure S2: Per sample LFQ values show consistent LC-MS/MS results through the experiment. No further normalization was performed on these values.

Supplementary Figure 3

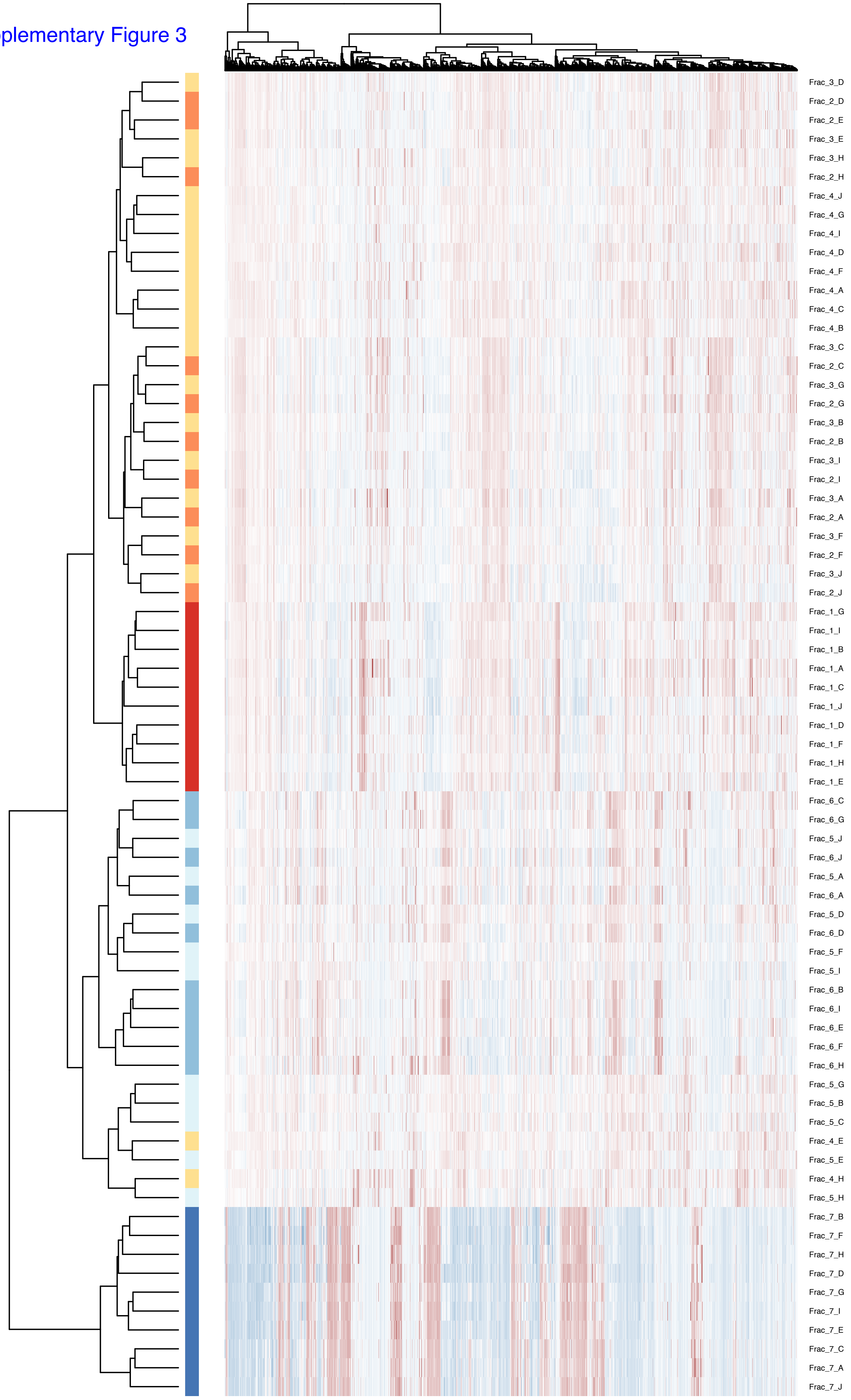

Figure S3: All protein clustering shows reasonable clustering of samples by fraction. In fractions 2 & 3 and 5 & 6 samples are clustered by subject within the larger fraction cluster. Subject is denoted by the letter at the end of the sample name.

Supplementary Figure 4

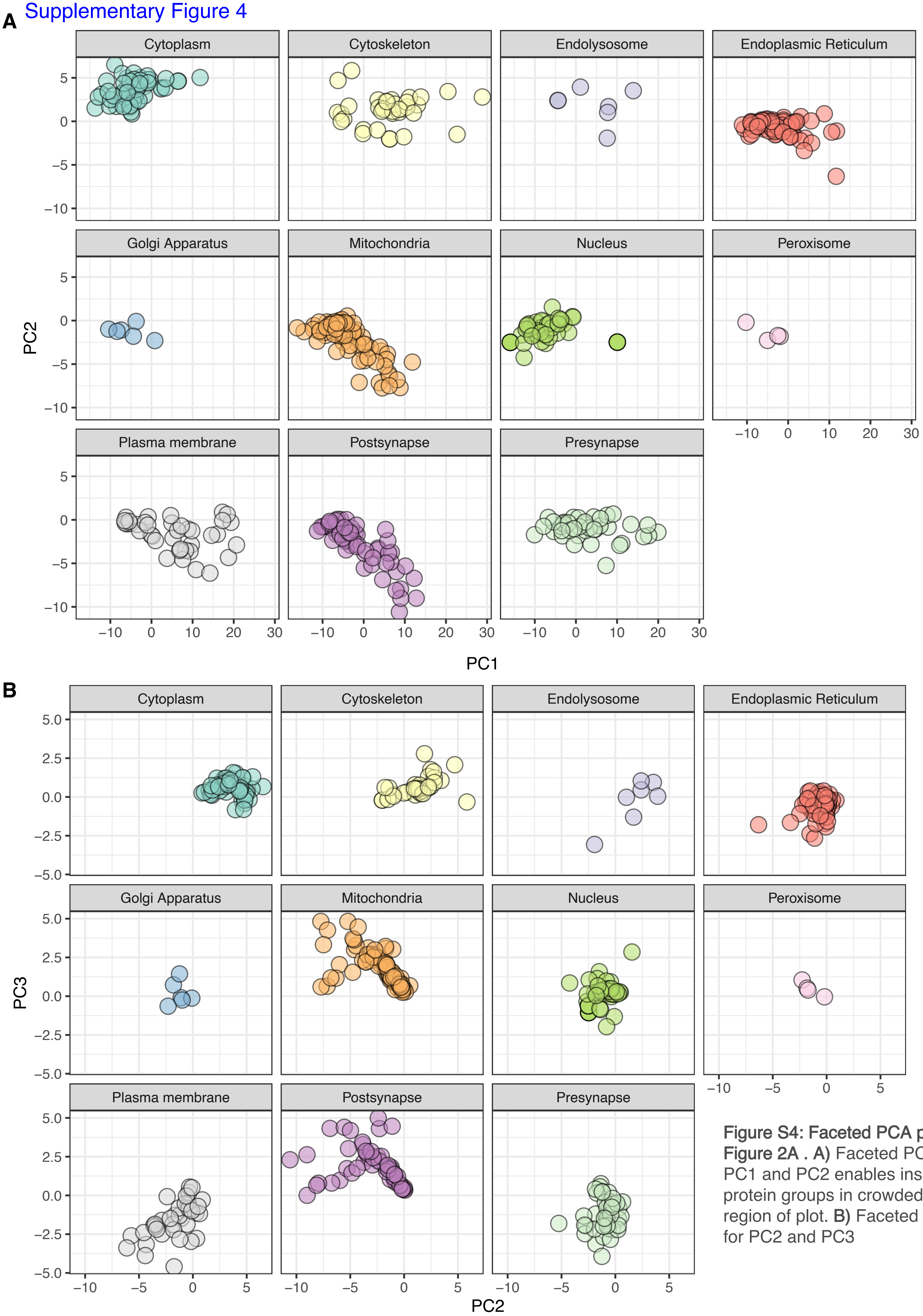

Figure S4: Faceted PCA plots from Figure 2A . A) Faceted PCA plots for PC1 and PC2 enables inspection of protein groups in crowded central region of plot. **B)** Faceted PCA plots for PC2 and PC3

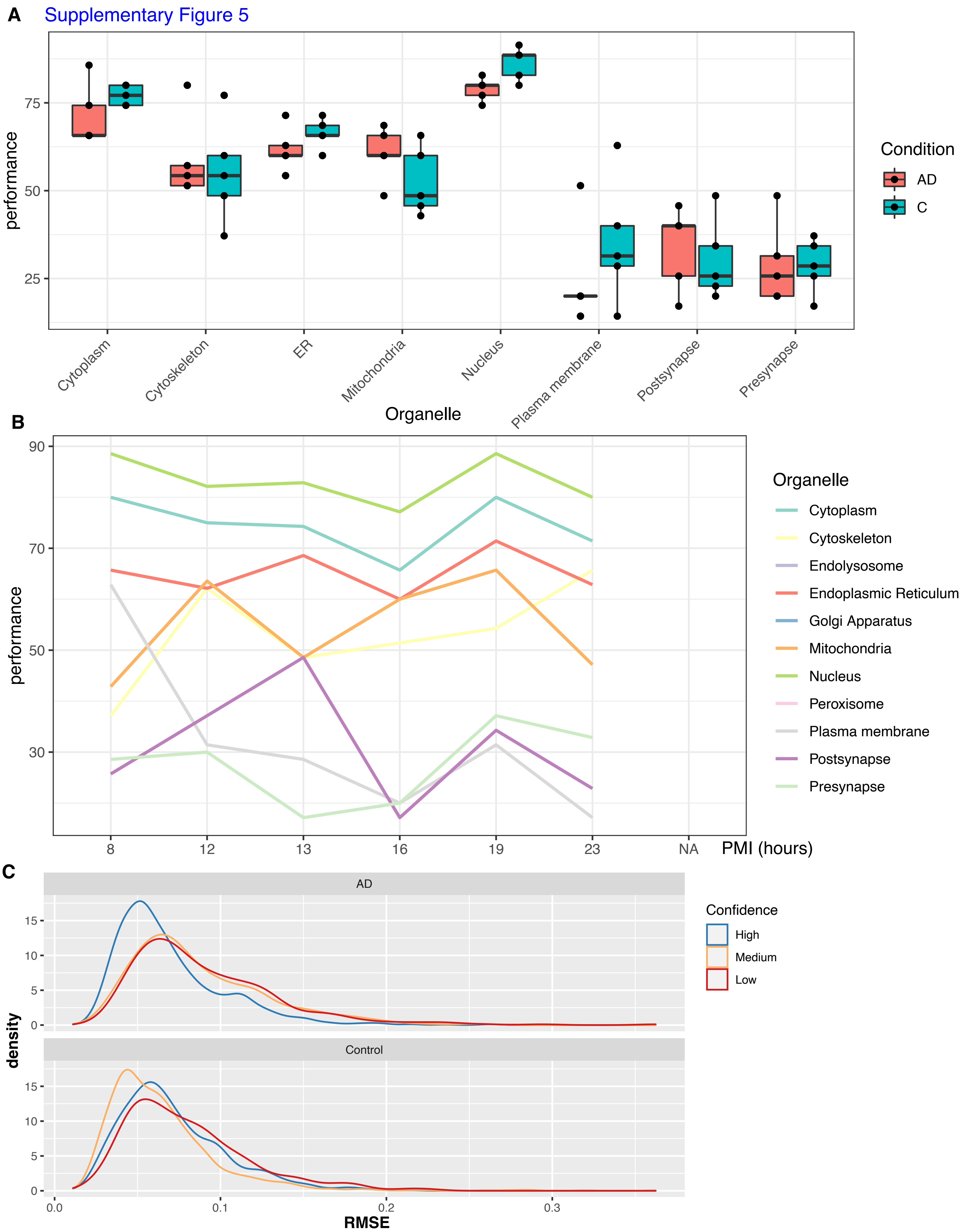

Figure S5: SVM performance is not affected by sample demographics. A) Boxplots showing performance (% of proteins assigned to correct organelle) of the SVM model for each organelle, split by AD and control samples. **B)** SVM performance does not decrease as post-mortem interval increases. The contribution of diagnostic condition and PMI variable to SVM performance were assessed by linear model, no significant associations were found. **C)** Proteins segregate reproducibly regardless of diagnosticcondition, as the RMSE distribution is centered at 0.05 (a 5% variation from the mean segregation pattern for that protein).

Supplementary Table legends

Supplementary Table 1: Table showing the protein yield of each fraction from each subject. Measurements include the final concentration of each lysate in μg/μL (Final Conc), the volume of each sample used for digestion in μL (Sample μL), and the total protein used for digestion in μg (Total protein).

Supplementary Table 2: Standard Maxquant output table includes LFQ data from all detected proteins prior to data filtering.

Supplementary Table 3: ANOVA output table includes ANOVA statistics for each protein and Tukey Honest Significant test p values for proteins that are significantly differentially abundant between fractions. The columns beginning with Frac indicate the significant fraction to fraction comparison, NA is included when the comparison is not significant.

Supplementary Table 4: Organellar marker list

Supplementary Table 5: SVM results table. Table shows the protein IDs, predicted location and prediction confidence for the control and AD diagnostic condition separately.

Supplementary Table 6: Localisation differences results table. Table shows Protein ID, Student's t-test results (Fraction being tested, n per diagnostic group, t-test T statistic, t-test p value, Benjamini-Hochberg adjusted p value, Significance

column for easy sorting) and entropy differences (Mean entropy value across all fractions for controls, mean entropy value for AD, difference between mean entropy values). Note that one entropy value is generated per subject, so this column is duplicate for all rows with the same Protein ID.
