# Supplementary material for: Proteomic characterization of post-mortem human brain tissue following ultracentrifugation-based subcellular fractionation
Source: Brain Commun. 2022 Apr 21;4(3):fcac103. doi: 10.1093/braincomms/fcac103 (PMC9123841; doi:10.1093/braincomms/fcac103)
Supplement: fcac103_Supplementary_Data [file Original_Submission_-_Author's_Response_(1) (1).pdf › Original_Submission_-_Author's_Response_(1) (1).pdf]

Dear Editor,

We thank the reviewers for their time spent with our manuscript and for their clear, constructive feedback. We have endeavored to answer all of their comments in line below. Additions to the text have been shown with track changes. Please note that the extra experiments involved in this rebuttal mean that we have added a further author to the paper, EC Ethier. All co-authors have agreed with her addition as a co-author.

Yours sincerely,

Becky Carlyle

Reviewer: 1

Comments to the Author
The manuscript by Kandigian et al. reported a proteomics study of human brain tissue. This study used density gradient-based ultracentrifugation and label-free LC-MS method to analyze frozen post-mortem human brain tissues and compared the differences in protein subcellular localization between brain tissue from individuals with Alzheimer's disease and control individuals. The goal of this study is to identify Alzheimer's disease-induced changes in protein subcellular location and provide insights into disease mechanisms and changing signaling pathways. An SVM model was developed to predict the subcellular locations of identified proteins. In addition, 85 proteins that had significant differences between AD and control in at least one fraction were identified.

Comments:
1. This manuscript reported a proof-of-concept study. It lacks independent validation of key findings obtained from the proteomics study. Confirmation of the disease-associated changes in protein localization with methods such as immunohistochemistry will significantly strengthen this manuscript.

To address this point we screened a number of antibodies for the proteins highlighted in Figure 3 for use in immunohistochemistry on human tissue sections. The most successful antibody that we screened was for GSK3β. We stained 5 sections from the angular gyrus of individuals with AD, and 5 age-matched controls, and analysed the overlap of the GSK3β signal with the DAPI nuclear signal. This quantification showed a strong trend towards increased signal overlap with the nucleus in control versus AD sections, agreeing with the mass-spectrometry that controls likely have a greater proportion of nuclear GSK3β. We have also mentioned in the results that subjectively, the control sections had more nuclear speckles of GSK3β than AD samples. The power of mass-spectrometry approaches lies in the ability to generate hypotheses about proteins that may not have good tools available for their study, such as strong specific antibodies, and so we believe that this first step towards validation of the GSK3β

finding, plus the expected re-distribution of Tau protein, should be sufficient for a proof of concept paper.

2. The data analysis of the proteomics study needs to be cleaned up. For example, the proteins identified from the decoy database should be removed because they are false identifications. Also, according to Table S1, 3861 proteins were identified with at least two unique peptides, not 4367 proteins as reported by the authors.

We thank the reviewer for bringing this important fact to our attention, this was an oversight in moving from initial data exploration in Perseus to coding our analysis reproducibly in R. The reverse decoy and potential contaminant proteins have now been removed at the beginning of the analysis, and the number have been updated to accurately reflect Table S1 (now Table S2). This sentence now reads: "3843 proteins with at least 2 unique peptides were identified in at least one sample, and 850 proteins were identified in every sample (Table S2)." All figures, tables and following analysis have been redone to account for these changes.

3. Although the imputation of missing values for modeling was described, it is not clear how the missing values were handled in the statistical analysis, such as ANOVA and within-fraction t-tests were?

We have added the following clarifying sentence to the methods section: "Proteins with over 60% missing values were excluded from further analysis, with missing values set as NAs (Figure S2), zero (Support Vector Machine and entropy/within fraction t-tests), or set as the minimum LFQ detected for that protein (Principal Component Analysis, Heatmaps, ANOVA and Tukey Honest Significant Difference test) . "

4. For the PCA analysis in Fig 1B, which loadings provide the best separation power of the fractions? Were these proteins overlapped with the marker proteins used for generating the organelle marker map?

We have not provided a formal analysis of the loadings for individual proteins in this manuscript, although this was something that we looked at in our exploratory analysis. One of the issues we came across in trying to formalize this approach was deciding where to place thresholds on the loading values to define whether they contributed strongly to each PC or not. We therefore made the decision not to include this form of analysis in the manuscript, but these loading values can be easily obtained by extracting the rotations from the PCA object in the R script provided with it. As an example, 43 of the 308 proteins found in the bottom 5% and top 5% of the eigenvector distribution for PC1 were also annotated marker proteins used in generating the organellar marker map.

In mentioning the organellar map the reviewer touches on one of the biggest issues that we faced in analysing this data – that the commonly used organellar references are not generated from complex tissues such as brain, but generally generated in cell culture systems. In this case we chose to rely on lists used by other investigators who used similar fractionation techniques, with the addition of well-annotated synaptic proteins, but further refinement of marker lists may be a future use for this technique in fresh tissue.

5. The headings of Table S2 are confusing. Why the headings of columns L to AF are dates (1-Feb, 1-Mar…)? Again, the proteins identified from the decoy database should be removed before the statistical analysis. It is not clear how the ANOVA analysis was conducted. If ANOVA was conducted separately for the AD and control groups, would it improve the subject clustering in PCA and heatmap?

We apologise for the appearance of the column names in this format – in responding to this comment we discovered that they are incorrectly interpreted if the .csv file is opened directly in Microsoft Excel. To resolve this issue we have re-imported this table into Excel and saved with the appropriate column names, which indicate the two fractions being contrasted for Tukey HSD testing. We have also written a supplementary table legend for all tables, to make use of these table easier.

The ANOVA was performed without knowledge of the diagnostic condition of the sample. Given the greatest separation in PC1 is between fractions, most clearly the cytosolic fraction and the membranous fractions, we do not think it is surprising that there is no clear subject-based clustering of AD from Controls here (shown below with diagnostic condition colored instead of Fraction). These effects are more subtle, and thus would not be reflected in the top principal components or the hierarchical clustering (which will also generally reflect the patterns observed in PC1). The ANOVA is being used purely to assess the efficacy of the fractionation technique, which appears to work very well. Fractions 2 and 3 are not well separated in PCs 1 or 2, hence their interspersion in the hierarchical clustering, but there is more separation between these fractions in PC4.

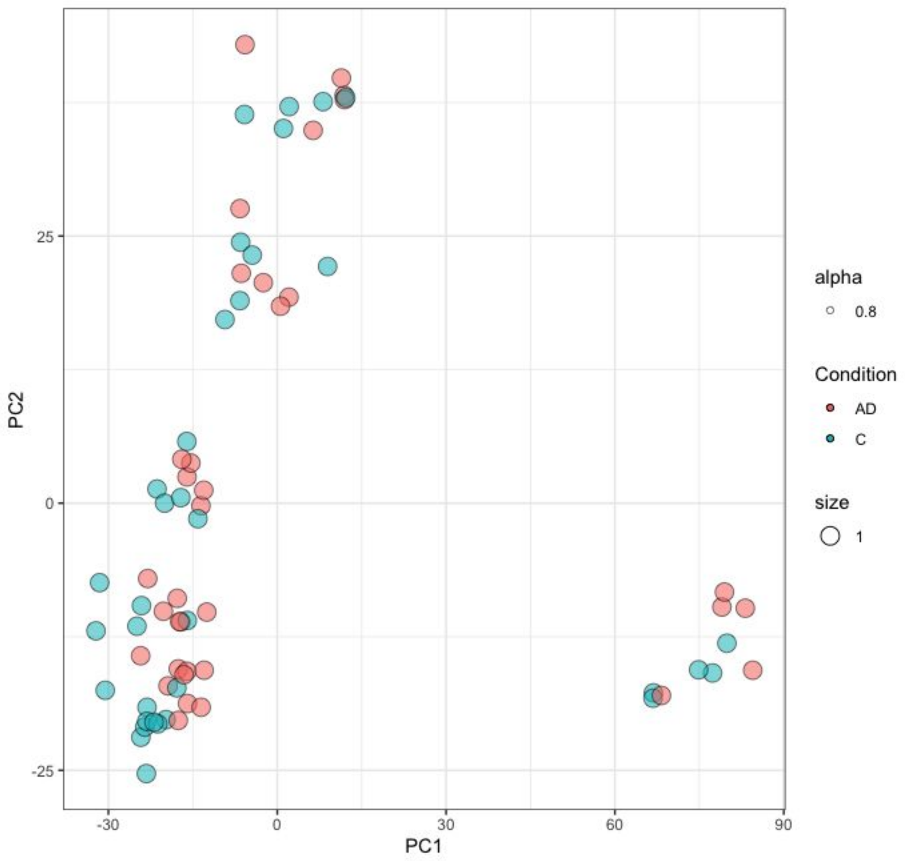

6. What are the marker proteins used in generating the organelle map? The list of proteins selected for organelle markers should be provided as a supplement file.

The marker list was available previously in the connected R Project, we have now brought it into the main paper as supplementary Table S4.

7. Has SVM modeling been performed for each sample? Do the box plots in Fig. S4A represent the SVM performance for each AD or Control sample? If this is the case, the SVM results should be provided as supplemental data. Why Table S3 has only one column on the confidence level of SVM prediction? It seems that only data from the control samples were used for determining the confidence level; why were AD samples not used?

Yes, SVM was performed on each sample individually, as per the Ithzak papers listed in the references, and the points on the boxplot represents each individual sample. We have added a small clarifying statement to the results to affirm this: "The SVM process was repeated ten times, once for each experimental subject."

We have altered Table S3 (now Table S5) so that it now shows the organelle assignment and the confidence for both diagnostic conditions, and now includes the

more descriptive Uniprot name so that it is easier to look up individual proteins without the use of Uniprot.

8. It is not clear how SVM modeling contributes to identifying the disease-associated alterations in subcellular localization. Can the changes in the confidence level of SVM prediction for each protein be used for identifying the proteins whose subcellular locations were changed due to AD?

We made the decision that due to the imperfect nature of the references used to define organellar localization for brain tissue, the similar fractionation patterns of mitochondria and postsynaptic density, and the small number of markers present from the smaller membranous fraction that prevented us from including them in the SVM, that we would use a method to look at movement of proteins that did not require a formal assignment of likely organellar localization. I have added an explanatory section to the methods section that explains this: "Due to the limitations inherent in the organellar marker lists for brain tissue, the small number of markers observed in some categories that prevented them from being included in the SVM, and the overlapping patterns of segregation of some organelles, we chose a method of identifying differences between diagnostic condition that was agnostic to the SVM classifications." The addition of confidence and localization for both the Control and AD groups in Table S3 now allows for the reader to compare the SVM results between condition for themselves.

9. The list of 85 disease-associated proteins and the statistical analysis results should be provided as supplemental data.

This list has now been included as Table S6, and includes ttest and entropy results.

Reviewer: 2

Comments to the Author
Protein localization is an essential aspect of proteome function that is difficult to assess in human postmortem brain tissue, yet almost certainly provides insight into cellular dysfunction that cannot be observed by transcriptomics or whole tissue proteomics. Here the Kandigian et al. apply spatial proteomics to human postmortem brain tissue, first describing the approaches utility to map proteins across cellular compartments and then demonstrating its ability to detect shifts in these localizations in a case control study (AD vs control). This manuscript will be of great interest to both human and mouse/rat neuroscientists. Several suggestions are provided bellow to increase reader accessibility (for those who wish to replicate this approach in their own studies) and better discuss the strengths and weaknesses of spatial proteomics in a complex organ such as the brain.

General/Abstract Notes:
In the abstract the author's state:
"Density gradient-based ultracentrifugation methods allow for subcellular fractionation and have
been utilized in cell lines and fresh mouse brain tissue to predict the subcellular location of
thousands of proteins, but to our knowledge have not been used in frozen human brain tissue."

This statement is not quite accurate. Sucrose density gradient centrifugation coupled with mass spectrometry has been applied to frozen postmortem brain tissue by several groups (Hahn, Cotter, Grant, MacDonald… and Arnold). The distinction is that while those studies focused on parsing synaptic microdomains, here the authors seek to expand this type of inquiry to separate out other overlooked organelles (nucleus, mitochondria, ER, golgi, ect) and utilize spatial proteomics machine learning approaches to categorize organelle localization. This does not diminish from the novelty or importance of the present study, but it would be better to place them in the correct context.

Indeed, the reviewer is absolutely correct. We have changed this statement in the abstract to read: "Density gradient-based ultracentrifugation methods allow for subcellular fractionation and have been utilized in cell lines, mouse, and human brain tissue to quantify thousands of proteins in specific enriched organelles such as the pre- and post-synapse. Serial ultra-centrifugation methods allow for the analysis of multiple cellular organelles from the same biological sample, and to our knowledge have not been previously applied to frozen human brain tissue."

"The use of frozen human tissue for tissue fractionation faces two major challenges, the post mortem interval, during which proteins may leach from their usual location into the cytosol, and freezing, which results in membrane breakdown."
This reviewer would argue that there is a third, larger challenge, in applying this type of spatial proteomics to brain tissue: the complexity of the organ at hand. This type of spatial proteomics (utilizing advances mass spectrometers and machine learning approaches) has, with a few exceptions, been applied to cultured cells, and appears to have only been applied to any type of brain tissue once prior to this study (Courtland et al. PMID: 33749590). Simply comparing the protein marker PCAs between the present study (human brain) and Courtland (mouse brain) to the PCAs from Hela cells in Itzhak et al suggests that the approach fairs better in culture. Again, while this does not diminish from the impact of this work, it needs to be addressed in the text. It is also worth noting the differences in centrifugation schemes in the references above (Courtland et al. appear to have collapsed the lower speeds and spread out the higher ones), and that, perhaps different fractionation cuts are better suited for brain tissue.

We thank the reviewer for bringing our attention to the Courtland paper, which we were not aware of, and have added a specific reference to it in the introduction, and changed the wording of that paragraph as a result: "Serial density gradient-based ultracentrifugation methods allow for subcellular fractionation and have been utilized in cell lines and primary cortical neurons to predict the subcellular location of thousands of proteins [13,14]. Spatial proteomic analysis of brains from ten-month old mice with a disease-relevant mutation in the SWIP gene showed disruption of the WASH complex resulting in altered abundance of hundreds of proteins across multiple subcellar fractions[15]. To our knowledge these serial fractionation techniques have not been used in frozen human tissue. The use of frozen human tissue for tissue fractionation faces three major challenges, the post-mortem interval, during which proteins may leach from their usual location into the cytosol, the complexity of the tissue which harbors hundreds of cell-types, and freezing, which results in membrane breakdown."

While it is definitely true (and fully expected) that this technique works less well in brain tissue than cells, directly comparing the PCA plots with the ones we show is not quite fair to our analysis. All these papers exclude cytosolic fractions from these plots, whereas we include them. Given a large proportion of the variance in our dataset is coming from the many differences between the cytosolic and other fractions, the differences between the other markers are compressed in these major PCs. Regardless, we have added to both the first and second paragraphs of the discussion to attempt to better contextualize these results given the current literature.

Methods:
Overall, the study design, sample preparation, mass spectrometry, and statistical analyses are rigorous and there is little to quibble with. It is likely that other groups will want to use these approaches in mouse/rat/human brain tissue. Additional methodological details would aid them:

1. A supplementary table should report the total protein yields (ug as determined by BCA) in each pellet from each spin. How much did yields differ between pellets from different spins? Did any of the average yields from each spin differ significantly between the AD and control groups?

We have added a supplementary table that shows protein yields and concentrations for each of the individual sample fractions. This table is referred to in the methods where we describe the sample used for digestion "For samples with protein contents below this threshold, 50 μl of the straight sample was used. Table S1 shows protein yields for each individual sample fraction," and in the results; "Post-mortem frozen cortical tissue from 10 individuals was subcellularly fractionated by progressive ultracentrifugation into 6 membranous organellar fractions and a final cytosolic supernatant fractions (Figure 1A). Student's T-test showed no significant differences in protein yield between the control and AD samples in any fraction (Table S1, plotted in Figure S1)." Figure S1 shows a potential trend towards a difference in yield in fraction P4 between AD and control, but

this is not significantly different (student's T-test, p value = 0.19, padj = 0.783). We have not included the t-test outcomes in the manuscript, but they can be generated in the accompanying r-script.

2. "For samples with protein contents below this threshold, 50 μl of the straight sample was used." If un-even amounts of protein were digested how was the amount of peptide load onto the column/analyzed by the mass spec normalized prior to injection? The raw file TICs appear to be decently uniform across different fractions from the same subject.

Indeed, the reviewer is correct. Digested peptides were normalized for loading following Nanodrop quantification. We have added this section to the methods to reflect this: "Eluted peptides were speed-vacced dry and dissolved in MS loading buffer (2% acetonitrile, 0.2% trifluoroacetic acid). A nanodrop (Thermo Scientific Nanodrop 2000 UV-Vis Spectrophotometer) was used to determine protein concentrations (A260/A280). Each sample was then further diluted with MS loading buffer to 0.08µg/µl, with 0.4ug (5µl) injected for LC-MS/MS analysis"

3. The inclusion of a range of PMIs in the samples is encouraging, but we are left to ask how protein levels across the different fractions shift between 0-8 hours. Ideally, comparison experiments using fresh mouse tissue (or better yet a time series modeling PMI) would be used to address this question. However, the reviewer realizes that such experiments are extremely onerous, and would not require them for publication. But if any existing data from preliminary experiments exists, or some comparison to the data from Courtland et al could be conducted, it would be helpful.

We agree that this would be helpful, and are hoping to follow up this work using low PMI tissue in this range. Unfortunately given the difference in centrifugation speeds and the extended fractionation scheme, I do not believe that a direct comparison of Courtland to our data would be appropriate, as segregation patterns will be quite different. They also focus on the area of the fraction profile where we observed many fewer known marker proteins, so a balanced SVM comparison between the two would be tricky.

Impact and discussion:
These data suggest that spatial proteomic techniques can be used to confidently assign a subcellular location to the majority of robustly quantified proteins in human post-mortem brain tissue". This statement seems to overstate the findings as only 50% of the quantified proteins could be assigned to an organelle with high confidence. Nevertheless, the data leaves the reviewer with a high degree of confidence that the method can detect case-control differences in protein distribution across fractions, but the interpretation of these findings is ultimately at the mercy of how well define these proteins were in the control samples.

We have changed this sentence to read, "These data suggest that spatial proteomic techniques can be used to assign a high-confidence subcellular location to approximately one-third of the robustly quantified proteins in human post-mortem brain tissue, a medium-confidence location to a further 40 percent, and that it can be used to highlight changes in localization profiles in response to a change in disease condition." Further, we appreciate that the reviewer believes this is a valuable technique for detecting potential differences between conditions, but that we find ourselves in a circular argument when it comes to assigning specific proteins to specific organelles. Are the references for marker proteins accurate? How can we build better ones without cross-species confounds or PMI artefacts?
