# Supplementary material for: Proteomic characterization of post-mortem human brain tissue following ultracentrifugation-based subcellular fractionation
Source: Brain Commun. 2022 Apr 21;4(3):fcac103. doi: 10.1093/braincomms/fcac103 (PMC9123841; doi:10.1093/braincomms/fcac103)
Supplement: fcac103_Supplementary_Data [file Original_Submission_-_Decision_Letter.pdf › Original_Submission_-_Decision_Letter.pdf]

Original Submission – Decision Letter

29-Sep-2021

Dear Dr. Carlyle,

Thank you for your recent submission BRAINCOM-2021-281 entitled "Proteomic Characterization of Post-Mortem Human Brain Tissue Following Ultracentrifugation-based Subcellular Fractionation" to Brain Communications.

Your manuscript has been reviewed, and while the reviewers and editorial team appreciate many aspects of the paper, they raise some concerns that you will need to address in order for the paper to be considered for publication.

If you can address these concerns, I invite you to submit a revised manuscript and a response to each of the reviewers' comments. The reviewer(s)' comments are included at the bottom of this message.

In addition to the reviewers' comments, please also address our Scientific Editor's comments added below to comply with our requirements for presentation of scientific data at Brain Communications.

Please format your revised manuscript according to our Instructions to Authors https://academic.oup.com/braincomms/pages/General_Instructions – a summary of key points is attached to help with this.

To revise your manuscript, click the link below or log into https://mc.manuscriptcentral.com/braincom and enter your Author Centre, where you will find your manuscript title listed under "Manuscripts with Decisions." Under "Actions," click on "Create a Revision." Your manuscript number will be appended to denote a revision.

*** PLEASE NOTE: This is a two-step process. After clicking on the link, you will be directed to a webpage to confirm. ***

https://mc.manuscriptcentral.com/braincom?URL_MASK=84afe50e8c0143f698c7ae2b8781dc82

Please upload a revised manuscript with changes tracked with track changes or in coloured text.

When submitting your revised manuscript, you will be asked to respond to the reviewer(s) comments. Please be as specific as possible in your response to all of the comments.

The editorial team at Brain Communications aims to increase transparency in scientific publishing and to demonstrate the value that peer review brings to the scientific process. Please therefore consider whether you are willing to have reviewer(s)' comments on your paper and your response published as supplementary data with the paper if it is accepted. This is entirely optional and will only be done if both the authors and reviewers agree. While submitting your revision there will be an opportunity to review or update the answer you gave previously, if necessary.

IMPORTANT: Your original files are available to you when you upload your revised manuscript. Please delete any redundant files before completing the submission.

Because we are trying to facilitate timely publication of manuscripts submitted to Brain Communications, we would appreciate your revised manuscript as soon as possible. If it is not possible for you to resubmit within 3 months, the option to submit a revision will expire, and we would then consider your revised paper as a new submission. However, if you need a little more time, please get in touch and we can extend this deadline for you.

Once again, thank you for submitting your manuscript to Brain Communications and I look forward to receiving your revision.

Best wishes

Dr. Rakez Kayed
Associate Editor, Brain Communications

Professor Tara Spires-Jones
Founding Editor, Brain Communications

Reviewer(s)' Comments to Author:

Reviewer: 1

Comments to the Author
The manuscript by Kandigian et al. reported a proteomics study of human brain tissue. This study used density gradient-based ultracentrifugation and label-free LC-MS method to analyze frozen post-mortem human brain tissues and compared the differences in protein subcellular localization between brain tissue from individuals with Alzheimer's disease and control individuals. The goal of this study is to identify Alzheimer's disease-induced changes in protein subcellular location and provide insights into disease mechanisms and changing signaling pathways. An SVM model was developed to predict the subcellular locations of identified proteins. In addition, 85 proteins that had significant differences between AD and control in at least one fraction were identified.

Comments:
1. This manuscript reported a proof-of-concept study. It lacks independent validation of key findings obtained from the proteomics study. Confirmation of the disease-associated changes in protein localization with methods such as immunohistochemistry will significantly strengthen this manuscript.
2. The data analysis of the proteomics study needs to be cleaned up. For example, the proteins identified from the decoy database should be removed because they are false identifications. Also, according to Table S1, 3861 proteins were identified with at least two unique peptides, not 4367 proteins as reported by the authors.
3. Although the imputation of missing values for modeling was described, it is not clear how the missing values were handled in the statistical analysis, such as ANOVA and within-fraction t-tests were?
4. For the PCA analysis in Fig 1B, which loadings provide the best separation power of the fractions? Were these proteins overlapped with the marker proteins used for generating the organelle marker map?
5. The headings of Table S2 are confusing. Why the headings of columns L to AF are dates (1-Feb, 1-Mar…)? Again, the proteins identified from the decoy database should be removed before the statistical analysis. It is not clear how the ANOVA analysis was conducted. If ANOVA was conducted separately for the AD and control groups, would it improve the subject clustering in PCA and heatmap?
6. What are the marker proteins used in generating the organelle map? The list of proteins selected for organelle markers should be provided as a supplement file.
7. Has SVM modeling been performed for each sample? Do the box plots in Fig. S4A represent the SVM performance for each AD or Control sample? If this is the case, the SVM results should be provided as supplemental data. Why Table S3 has only one column on the confidence level of SVM prediction? It seems that only data from the control samples were used for determining the confidence level; why were AD samples not used?
8. It is not clear how SVM modeling contributes to identifying the disease-associated alterations in subcellular localization. Can the changes in the confidence level of SVM prediction for each protein be used for identifying the proteins whose subcellular locations were changed due to AD?
9. The list of 85 disease-associated proteins and the statistical analysis results should be provided as supplemental data.

Reviewer: 2

Comments to the Author
Protein localization is an essential aspect of proteome function that is difficult to assess in human postmortem brain tissue, yet almost certainly provides insight into cellular dysfunction that cannot be observed by transcriptomics or whole tissue proteomics. Here the Kandigian et al. apply spatial proteomics to human postmortem brain tissue, first describing the approaches utility to map proteins across cellular compartments and then demonstrating its ability to detect shifts in these localizations in a case control study (AD vs control). This manuscript will be of great interest to both human and mouse/rat neuroscientists. Several suggestions are provided bellow to increase reader accessibility (for those who wish to replicate this approach in their own studies) and better discuss the strengths and weaknesses of spatial proteomics in a complex organ such as the brain.

General/Abstract Notes:
In the abstract the author's state:
"Density gradient-based ultracentrifugation methods allow for subcellular fractionation and have been utilized in cell lines and fresh mouse brain tissue to predict the subcellular location of thousands of proteins, but to our knowledge have not been used in frozen human brain tissue."

This statement is not quite accurate. Sucrose density gradient centrifugation coupled with mass spectrometry has been applied to frozen postmortem brain tissue by several groups (Hahn, Cotter, Grant, MacDonald… and Arnold). The distinction is that while those studies focused on parsing synaptic microdomains, here the authors seek to expand this type of inquiry to separate out other overlooked organelles (nucleus, mitochondria, ER, golgi, ect) and utilize spatial proteomics machine learning approaches to categorize organelle localization. This does not diminish from the novelty or importance of the present study, but it would be better to place them in the correct context.

"The use of frozen human tissue for tissue fractionation faces two major challenges, the post mortem interval, during which proteins may leach from their usual location into the cytosol, and freezing, which results in membrane breakdown."

This reviewer would argue that there is a third, larger challenge, in applying this type of spatial proteomics to brain tissue: the complexity of the organ at hand. This type of spatial proteomics (utilizing advances mass spectrometers and machine learning approaches) has, with a few exceptions, been applied to cultured cells, and appears to have only been applied to any type of brain tissue once prior to this study (Courtland et al. PMID: 33749590). Simply comparing the protein marker PCAs between the present study (human brain) and Courtland (mouse brain) to the PCAs from Hela cells in Itzhak et al suggests that the approach fairs better in culture. Again, while this does not diminish from the impact of this work, it needs to be addressed in the text. It is also worth noting the differences in centrifugation schemes in the references above (Courtland et al. appear to have collapsed the lower speeds and spread out the higher ones), and that, perhaps different fractionation cuts are better suited for brain tissue.

Methods:

Overall, the study design, sample preparation, mass spectrometry, and statistical analyses are rigorous and there is little to quibble with. It is likely that other groups will want to use these approaches in mouse/rat/human brain tissue. Additional methodological details would aid them:

1. A supplementary table should report the total protein yields (ug as determined by BCA) in each pellet from each spin. How much did yields differ between pellets from different spins? Did any of the average yields from each spin differ significantly between the AD and control groups?
2. "For samples with protein contents below this threshold, 50 μl of the straight sample was used." If un-even amounts of protein were digested how was the amount of peptide load onto the column/analyzed by the mass spec normalized prior to injection? The raw file TICs appear to be decently uniform across different fractions from the same subject.
3. The inclusion of a range of PMIs in the samples is encouraging, but we are left to ask how protein levels across the different fractions shift between 0-8 hours. Ideally, comparison experiments using fresh mouse tissue (or better yet a time series modeling PMI) would be used to address this question. However, the reviewer realizes that such experiments are extremely onerous, and would not require them for publication. But if any existing data from preliminary experiments exists, or some comparison to the data from Courtland et al could be conducted, it would be helpful.

Impact and discussion:

These data suggest that spatial proteomic techniques can be used to confidently assign a subcellular location to the majority of robustly quantified proteins in human post-mortem brain tissue". This statement seems to overstate the findings as only 50% of the quantified proteins could be assigned to an organelle with high confidence. Nevertheless, the data leaves the reviewer with a high degree of confidence that the method can detect case-control differences in protein distribution across fractions, but the interpretation of these findings is ultimately at the mercy of how well define these proteins were in the control samples.

Comments from the Scientific Editor:

-For all the plots in the figures, please, report the test used, relevant test statistic values alongside p values, what each data point represents in the figure legend and if the n<10, please, show the single data points. Please see instructions for authors for more details.

-Please supply a graphical abstract. A graphical abstract is a visual representation of the central finding or methodology of the paper. It will be displayed in the table of contents. Authors should strive to make the graphical abstract informative, interesting, visually appealing, and straightforward (no caption needed, no abbreviations except gene names). Please upload as a separate high resolution image file (tiff preferred). The technical requirements for the graphical abstracts are:
 --size of the submitted image: 1200 pixels square at 300dpi
 --font of the text: Arial 12-16 point (smaller fonts will not be legible online)

-Please add an Abbreviated Summary (up to 50 words) of your paper that captures the main purpose and conclusions of the work. The summary should state the main results written in the third person ('[1st author surname] et al. report that …') and conclusion. This summary will be used in the contents list online and should not contain any abbreviations other than those on the accepted abbreviations list

and accepted gene/protein names, making it accessible to the non-specialist. Please upload it as a Word document. Allowed abbreviations: AIDS; ANOVA; AMPA; ATP; A,T,C,G; CNS; CSF; CT; DNA; ECG; EEG; EMG; GABA; HIV; MRI; NMDA; PET; PCR; RNA.

- To comply with our ethical standards, the appropriate checklist must be included (see Reporting Guideline section within 'Instructions to authors').

-Please include a paragraph about data availability in the 'Materials and Methods' section. We strongly encourage data sharing either as supplementary information or uploads to repositories which you can link in the paper.
